# Associations of device-measured physical activity across adolescence with metabolic traits: Prospective cohort study

**DOI:** 10.1371/journal.pmed.1002649

**Published:** 2018-09-11

**Authors:** Joshua A. Bell, Mark Hamer, Rebecca C. Richmond, Nicholas J. Timpson, David Carslake, George Davey Smith

**Affiliations:** 1 MRC Integrative Epidemiology Unit at the University of Bristol, Bristol, United Kingdom; 2 Population Health Sciences, Bristol Medical School, University of Bristol, Bristol, United Kingdom; 3 School of Sport, Exercise & Health Sciences, Loughborough University, Leicestershire, United Kingdom; Stanford University, UNITED STATES

## Abstract

**Background:**

Multiple occasions of device-measured physical activity have not been previously examined in relation to metabolic traits. We described associations of total activity, moderate-to-vigorous physical activity (MVPA), and sedentary time from three accelerometry measures taken across adolescence with detailed traits related to systemic metabolism.

**Methods and findings:**

There were 1,826 male and female participants recruited at birth in 1991–1992 via mothers into the Avon Longitudinal Study of Parents and Children offspring cohort who attended clinics in 2003–2005, 2005–2006, and 2006–2008 who were included in ≥1 analysis. Waist-worn uniaxial accelerometers measured total activity (counts/min), MVPA (min/d), and sedentary time (min/d) over ≥3 d at mean age 12y, 14y, and 15y. Current activity (at age 15y), mean activity across occasions, interaction by previous activity, and change in activity were examined in relation to systolic and diastolic blood pressure, insulin, C-reactive protein, and 230 traits from targeted metabolomics (nuclear magnetic resonance spectroscopy), including lipoprotein cholesterol and triglycerides, amino and fatty acids, glycoprotein acetyls, and others, at age 15y. Mean current total activity was 477.5 counts/min (SD = 164.0) while mean MVPA and sedentary time durations were 23.6 min/d (SD = 17.9) and 522.1 min/d (SD = 66.0), respectively. Mean body mass index at age 15y was 21.4 kg/m^2^ (SD = 3.5). Correlations between first and last activity measurement occasions were low (e.g., *r* = 0.40 for counts/min). Current activity was most strongly associated with cholesterol and triglycerides in high-density lipoprotein (HDL) and very low-density lipoprotein (VLDL) particles (e.g., −0.002 mmol/l or −0.18 SD units; 95% CI −0.24–−0.11 for triglycerides in chylomicrons and extremely large very low-density lipoprotein [XL VLDL]) and with glycoprotein acetyls (−0.02 mmol/l or −0.16 SD units; 95% CI −0.22–−0.10), among others. Associations were similar for mean activity across 3 occasions. Attenuations were modest with adjustment for fat mass index based on dual-energy X-ray absorptiometry (DXA). In mutually adjusted models, higher MVPA and sedentary time were oppositely associated with cholesterol and triglycerides in VLDL and HDL particles (MVPA more strongly with glycoprotein acetyls and sedentary time more strongly with amino acids). Associations appeared less consistent for sedentary time than for MVPA based on longer-term measures and were weak for change in all activity types from age 12y–15y. Evidence was also weak for interaction between activity types at age 15y and previous activity measures in relation to most traits (minimum *P* = 0.003; median *P* = 0.26 for counts/min) with interaction coefficients mostly positive. Study limitations include modest sample sizes and relatively short durations of accelerometry measurement on each occasion (3–7 d) and of time lengths between first and last accelerometry occasions (<4 years), which can obscure patterns from chance variation and limit description of activity trajectories. Activity was also recorded using uniaxial accelerometers which predated more sensitive triaxial devices.

**Conclusions:**

Our results support associations of physical activity with metabolic traits that are small in magnitude and more robust for higher MVPA than lower sedentary time. Activity fluctuates over time, but associations of current activity with most metabolic traits do not differ by previous activity. This suggests that the metabolic effects of physical activity, if causal, depend on most recent engagement.

## Abstract 2

Using repeat measurements in adolescents, Joshua A Bell and colleagues study the relationship between engagement in physical activity and metabolism over time in early life.

## Author summary

**Why was this study done?:**

- Much of what is known about the benefits of physical activity for population health comes from studies using one-off measures of activity. Activity fluctuates over time, however, and such measures may not capture longer-term exposure or describe its true health effects.
- Previous studies examining associations of physical activity and sedentary time as recorded by an accelerometer device with metabolism have not only lacked repeated activity measures but have also been limited in the scope and detail of metabolic traits described. Deeper phenotyping could reveal specific pathways through which physical activity may protect against type 2 diabetes and heart disease.

**What did the researchers do and find?:**

- We examined relations of physical activity with metabolism by integrating data from three repeat measures of accelerometry taken on separate occasions across adolescence with data from targeted metabolomics. This allowed us to compare associations of current activity, longer-term activity, and change in activity with detailed traits related to systemic metabolism. This also allowed us to see whether associations of current activity with metabolic traits depend on previous activity (e.g., whether effects are cumulative or acute).
- We found higher total activity to associate widely across metabolic traits including higher cholesterol in high-density lipoprotein (HDL) particles, lower cholesterol and triglycerides in very low-density lipoprotein (VLDL) particles, and lower inflammatory glycoprotein acetyls, among others. Associations were small in magnitude and more robust for higher moderate-to-vigorous activity than lower sedentary time. We also found that activity levels fluctuate over time but that associations of current activity with most metabolic traits do not differ by previous activity.

**What do these findings mean?:**

- Beneficial effects of higher physical activity on metabolic health, if causal and not just reflective of unmeasured aspects of body size, may be driven more by time spent intensely active than by less time spent sitting down. These effects are probably small and short lived, meaning that physical activity would need to be engaged in regularly to prevent disease.

## Introduction

Cardiometabolic diseases contribute the most to non-violent death rates worldwide [1]. Those chiefly responsible for this contribution are type 2 diabetes, coronary heart disease (CHD), and stroke [1], all of which have origins in dysfunctional metabolism [2]. Decades of observational studies have established dose–response associations of higher duration and intensity of physical activity with reduced risk of cardiometabolic diseases and their precursors [3–5]. Physical activity is now commonly measured using accelerometry devices which track intensity and duration of body movement—an advance over self-report methods, which are prone to measurement error from imprecise and biased recall and can thereby mask the true magnitude of associations [6,7]. Accelerometer data are often collected over several days to estimate mean activity level. Such measures, however, still only describe a single measurement occasion in the lifespan of a cohort and may only provide a snapshot of longer-term activity.

The few studies that have repeat accelerometry data report considerable instability over time [8–11], but repeat accelerometry has not been examined in relation to metabolic traits beyond adiposity [8] and fatty liver [11]. It is therefore unclear how longer-term activity and change in activity are associated with metabolic traits. This also has implications for understanding the dynamic nature of physical activity effects—whether they are acute or cumulative. One-off accelerometry measures of sedentary time are also associated with metabolic traits [12,13] and mortality risk [14], although repeated measures are also scarce. Importantly, there are limited data on physical activity and sedentary time in relation to detailed metabolic traits derived from novel metabolomics platforms. These could help clarify mechanisms underlying potential activity benefits by revealing specific components of summary traits such as serum cholesterol that track alongside activity. One recent study examined accelerometry at a single time point in relation to metabolites from untargeted mass spectrometry [15,16], while one examined repeated activity with self-report in relation to more clinically relevant metabolomic traits from targeted nuclear magnetic resonance (NMR) [17].

The extent to which adiposity confounds associations of physical activity with metabolic traits is also unclear [18]. Adverse effects of higher adiposity on metabolic trait levels are strongly supported by prospective observational [19], instrumental variable [20], and surgical reversal [21] studies, while recent instrumental variable studies [22–24] also suggest that higher adiposity reduces activity and lengthens sedentary time. Alternatively, physical activity may help mediate effects of adiposity on metabolic functioning if activity is truly causal for such traits.

This study integrated data from three repeat measures of accelerometry taken on separate occasions across adolescence with data from targeted NMR metabolomics to better describe associations of current and longer-term physical activity and sedentary time with detailed traits related to systemic metabolism. Interaction by previous activity and change in activity were also investigated in relation to these traits. Confounding by adiposity was examined in each instance with adjustment for dual-energy X-ray absorptiometry (DXA)-assessed fat mass index.

## Methods

A prospective analysis plan written in March 2017 before analyses were conducted is included as S1 Protocol. Analyses were not changed following peer review. This study is reported according to the Strengthening the Reporting of Observational Studies in Epidemiology (STROBE) guidelines (checklist included as S1 STROBE Checklist).

### Study population

Data were drawn from offspring participants of the Avon Longitudinal Study of Parents and Children (ALSPAC), a population-based birth cohort study in which 14,541 pregnant women with an expected delivery date between 1 April 1991 and 31 December 1992 were recruited from the former county of Avon in southwest England. Offspring who were alive at 1 year (*n* = 13,988) have since been followed with multiple assessments [25], with an additional 713 children enrolled over the course of the study. Clinical data for present analyses were collected in 2003–2005, 2005–2006, and 2006–2008. Ethical approval was obtained from the ALSPAC Law and Ethics Committee and the Local Research Ethics Committees. Cohort details and data descriptions are available at www.bristol.ac.uk/alspac/researchers/access.

### Assessment of physical activity and sedentary time

Physical activity was assessed with a waist-worn uniaxial ActiGraph device (AM7164 2.2, ActiGraph LLC, Fort Walton Beach, FL, United States of America) on three occasions at approximate mean ages of 12y, 14y, and 15y. On each occasion, the device was worn during waking hours for up to 7 d, during which participants recorded device wear time and timing of activities such as swimming or cycling. Nonwear periods were identified as 10 or more minutes of consecutive zero counts and were deleted from each processing file [26]. A day of accelerometry was considered valid if at least 600 minutes of activity were recorded. Participants were included if they had at least 3 valid days of accelerometry on occasions relevant to that analysis. Data are recorded as counts that result from summing postfiltered accelerometer values (raw data at 30 Hz) into epoch units. Count values vary based on raw acceleration frequency and intensity and the filtering process by which counts are produced are specific to ActiGraph. Total physical activity was expressed as the mean number of counts per minute (CPM) on valid days. Duration of moderate-to-vigorous physical activity (MVPA) was expressed as the mean number of daily minutes spent at or above 3,600 CPM on valid days. Duration of sedentary time was expressed as the mean number of daily minutes spent below 199 CPM on valid days. Activity derivations are further described elsewhere [26,27]. The general term ‘activity’ is used here to describe total movement intensity (based on counts/min), and terms ‘MVPA’ and ‘sedentary time’ are used to describe the amount of time spent in the higher and lower end of the movement intensity spectrum, respectively.

### Assessment of metabolic traits

As part of a clinical assessment at approximately age 15y, systolic and diastolic blood pressure (SBP and DBP, respectively) were examined twice in succession while seated with the arm supported using an appropriately-sized cuff and a DINAMAP 9301 device. Mean values were used to estimate resting levels. Fasting blood samples were drawn, from which circulating insulin (mu/l) and C-reactive protein (mg/l) were quantified. Proton NMR spectroscopy was performed as part of a targeted metabolomics platform [28] to quantify 230 metabolic traits (150 concentrations plus 80 ratios) including the concentration and size of lipoproteins and their cholesterol and triglyceride content, lipid and glycaemic precursors including fatty acids and amino acids, inflammatory factors, and others.

### Assessment of covariates

Basic demographic traits considered were sex, age (in months) at the time of physical activity assessment, ethnicity (‘white’ versus ‘non-white’), and the highest level of education attained by the participant’s mother as reported near the time of delivery (‘Certificate of Secondary Education’; ‘vocational’; ‘O-level’; ‘A-level’; or ‘degree’ using English standards) as an indicator of socioeconomic position at birth. Maternal education has shown similar association patterns as paternal education and head of household occupational social class in ALSPAC with regards to offspring general health as indicated by length at birth and growth through childhood [29]. At approximately age 15y, smoking behaviour was estimated via questionnaire and grouped as ‘never smoked’, ‘has smoked a whole cigarette but does not smoke weekly’, and ‘smokes at least once per week’. Alcohol consumption was estimated via questionnaire at age 15y and grouped as ‘never consumed’, ‘consumed once/twice or occasionally in the past’, ‘consumes less than weekly’, and ‘consumes at least once per week’. These questionnaires were part of repeat self-reported behaviour assessments starting from age 10y [25,30]. At age 12y, 14y, and 15y, participants underwent body scanning with DXA using a Lunar Prodigy narrow fan beam densitometer, from which estimates of total body fat mass (in kg, excluding lean and bone mass) were obtained. Scans were screened for anomalies, motion, and material artefacts and were realigned when necessary as detailed elsewhere [31]. Height was measured while in light clothing without shoes, recorded to the nearest 0.1 cm using a Harpenden stadiometer. Fat mass index (FMI) was calculated by dividing total fat mass by the square of height (in meters). Body mass index (BMI) was also calculated using weight (as kg/m^2^) for descriptive purposes. Accelerometer wear time was based on the total number of minutes recorded on valid days. The month in which the accelerometer was worn was also considered to account for seasonal variation in activity.

### Statistical approach

All activity and metabolic traits were converted into standardised (z-score) units for analyses (mean = 0.0; SD = 1.0). Pearson correlation coefficients were examined between each activity trait at age 12y (first recorded measure) and 15y (last recorded measure) (e.g., CPM at 12y with CPM at 15y). This was used to estimate the relative stability of each activity trait over time.

In the first set of analyses, associations of total activity (CPM), MVPA duration, and sedentary duration at age 15y were examined in relation to metabolic traits at age 15y using separate linear regression models with robust standard errors to accommodate skewed outcome distributions in replace of log transformations. Models of CPM were adjusted for covariates in two cumulative stages: 1) for age at the time of CPM assessment, sex, ethnicity, maternal education, smoking, alcohol, and accelerometer wear time and wear month; and 2) for FMI at the time of CPM assessment to examine effect size attenuations. Models of MVPA and sedentary time were adjusted for covariates in three cumulative stages: 1) for age at the time of exposure assessment, sex, ethnicity, maternal education, and accelerometer wear time and wear month; 2) for the alternative activity measure (MVPA or sedentary time); and 3) for FMI at the time of MVPA or sedentary time assessment to examine effect size attenuations.

In the second set of analyses, associations were examined between levels of each activity trait calculated as the mean of three standardised measurement occasion values (at age 12y, 14y, and 15y) representing a current measure plus two historical measures in relation to metabolic traits at age 15y using separate linear models. Covariate adjustments were arranged as previous except that adjustments for MVPA or sedentary time, accelerometer wear time, and FMI were all based on the mean of three measures (at age 12y, 14y, and 15y). Linear models were also constructed to examine interaction terms of each activity measure at 15y with the mean of its two historical measures (e.g., CPM at age 15y with mean of CPM at 14y and 12y) in relation to metabolic traits at age 15y. *P*-values from these interaction terms and coefficient directions were used as evidence of whether associations of current activity with metabolic traits differ according to historical activity.

In the third set of analyses, associations of change in each activity trait from age 12y–15y (calculated as standardised trait at 15y minus standardised trait at 12y) were examined in relation to metabolic traits at age 15y. Adjustment for age was based on age at 12y. Smoking and alcohol measures were excluded as these did not precede initial exposure assessment. Adjustment for MVPA/sedentary time, accelerometer wear time, and FMI were each based on change values from age 12y–15y.

### Supplementary analyses

To better examine directionality of associations of activity with metabolic traits, the most-adjusted models above were further adjusted for metabolic trait values measured using the same blood pressure and NMR assessment techniques at an available pre-baseline occasion, at approximately age 8y. For example, total activity at 15y in relation to glycoprotein acetyls at 15y was examined with adjustment for glycoprotein acetyls at 8y. Insulin and C-reactive protein were excluded from this analysis as they were not measured at this 8y pre-baseline occasion.

For comparison with metabolic trait outcomes, we additionally examined associations of each activity exposure in relation to FMI at age 15y as an outcome (rather than a confounder). Models were otherwise adjusted for the same set of covariates described previously and were then examined with further adjustment for FMI measured using DXA at a pre-baseline occasion, at approximately age 10y to better address directionality of associations.

All above analyses were performed on an unrestricted sample of participants with data for each separate outcome allowing sample sizes to vary. Analyses were repeated on a complete-case sample of participants who had data for all variables (each activity trait on all three occasions, plus all covariates, plus all metabolic traits at age 15y) to examine the potential for inconsistent sample size from nonrandom missing data to bias results.

Seventeen principal components explained 95% of the variance in these highly correlated metabolic traits in previous ALSPAC analyses [32], approximating the number of independent tests for use in Bonferroni multiple testing corrections. Here, we focus on effect size and precision and interpret *P* values as continuous indicators of evidence strength [33,34]. Analyses were conducted using Stata 15.1 (StataCorp, College Station, Texas, USA).

## Results

### Sample characteristics

**Fig 1:**
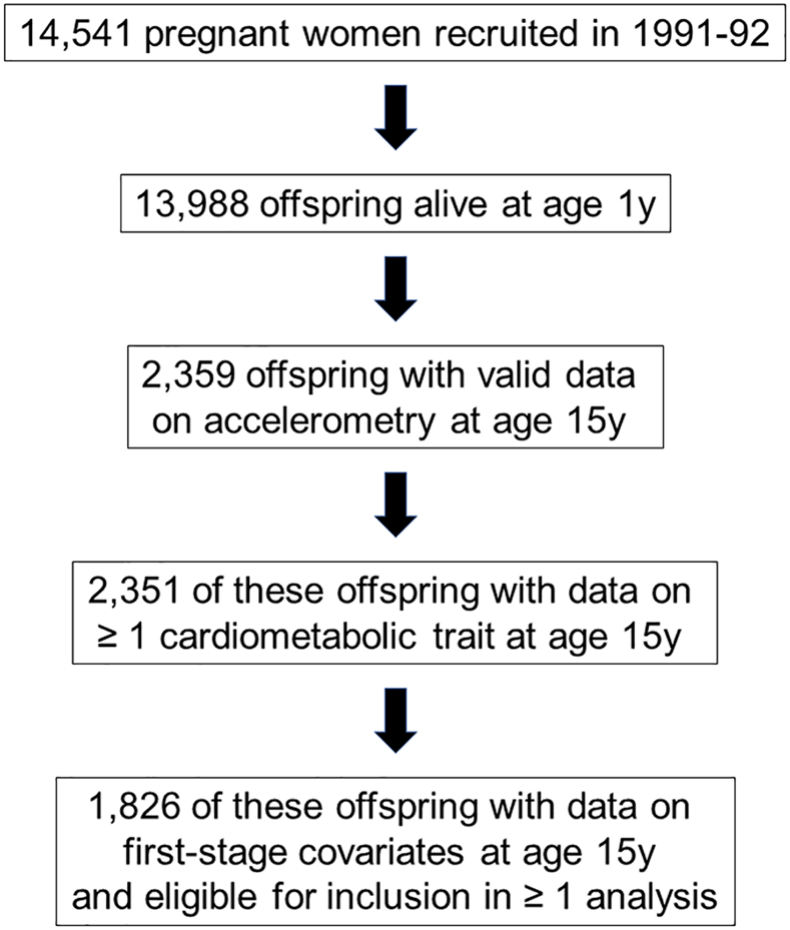
Selection of participants from the ALSPAC offspring cohort eligible for ≥ 1 analysis. ALSPAC, Avon Longitudinal Study of Parents and Children.

There were 1,826 participants included in at least one analysis (i.e., had at least 3 valid days of accelerometry data at age 15y, plus data on at least one metabolic trait at age 15y, plus data on all step-one covariates). Selection of eligible participants is illustrated in Fig 1. Mean age was 15.4 (SD = 0.2) y and mean time between age 12y and 15y measurement occasions was 3.7 (SD = 0.3) y. A small majority of included participants was female (55.6%), while a minority was of a nonwhite ethnicity (3.6%). Weekly smoking and weekly drinking were rare (4.9% and 15.3%, respectively). At age 15y, mean total physical activity was 477.5 (SD = 164.0) CPM, mean MVPA duration was 23.6 (SD = 17.9) min/d, and mean sedentary time was 522.1 (SD = 66.0) min/d. Among participants with at least 3 valid days of accelerometry at age 15y, 72.2% had at least 5 valid days, while 22.0% had 7 valid days. Across ages 12y, 14y, and 15y, total activity decreased, MVPA was relatively stable, and sedentary time increased. Other characteristics are shown in Table 1.

**Table 1:** Characteristics of eligible participants from the ALSPAC offspring cohort.

	Participants included in ≥ 1 analysis (*n* = 1,826)
**Demographics**	
Exact age (years) at 15y clinic—mean (SD)	15.4 (0.2)
Female—percentage (*n*)	55.6 (1,015)
Nonwhite ethnicity—percentage (*n*)	3.6 (66)
Low maternal education—percentage (*n*)	50.5 (922)
Smokes at least weekly at age 15y—percentage (*n*)	4.9 (89)
Consumes alcohol at least weekly at age 15y—percentage (*n*)	15.3 (280)
Fat mass index (kg/m^2^) at age 15y—mean (SD)	5.6 (3.3)
Body mass index (kg/m^2^) at age 15y—mean (SD)	21.4 (3.5)
Has obesity* at age 15y—percentage (*n*)	4.2 (75)
**Activity traits at age 12y**	
Total (counts/min)—mean (SD)	586.0 (168.3)
Duration of moderate-to-vigorous (min/d)—mean (SD)	21.7 (14.0)
Duration of sedentary (min/d)—mean (SD)	434.5 (64.7)
**Activity traits at age 14y**	
Total (counts/min)—mean (SD)	528.1 (177.9)
Duration of moderate-to-vigorous (min/d)—mean (SD)	23.3 (16.2)
Duration of sedentary (min/d)—mean (SD)	489.4 (68.1)
**Activity traits at age 15y**	
Total (counts/min)—mean (SD)	477.5 (164.0)
Duration of moderate-to-vigorous (min/d)—mean (SD)	23.6 (17.9)
Duration of sedentary (min/d)—mean (SD)	522.1 (66.0)
**Summary metabolic traits at age 15y**	
SBP (mmHg)—mean (SD)	123.1 (10.4)
DBP (mmHg)—mean (SD)	67.5 (8.5)
Total cholesterol (mmol/l)—mean (SD)	3.6 (0.6)
LDL cholesterol (mmol/l)—mean (SD)	1.1 (0.3)
HDL cholesterol (mmol/l)—mean (SD)	1.4 (0.2)
Triglycerides (mmol/l)—mean (SD)	0.9 (0.3)
Insulin (mu/l)—median (range)	8.9 (1.2–39.6)
Glucose (mmol/l)—mean (SD)	4.3 (0.3)
Glycoprotein acetyls (mmol/l)—mean (SD)	1.2 (0.1)
C-reactive protein (mg/l)—median (range)	0.38 (0.1–72.6)

Participants described are those with data on accelerometry at age 15y, data on at least one metabolic trait at 15y, and data on step-one covariates. ‘Low maternal education’ defined as highest level of education attained being Certificate of Secondary Education, vocational, or O level (not A level or degree).

*Based on a BMI threshold for obesity (≥ 28.56 kg/m^2^ for males and ≥ 29.19 kg/m^2^ for females) at mean age 15.4y equivalent to ≥ 30.00 kg/m^2^ at age 18y [35].

**Abbreviations:** ALSPAC, Avon Longitudinal Study of Parents and Children; BMI, body mass index; DBP, diastolic blood pressure; HDL, high-density lipoprotein; LDL, low-density lipoprotein; SBP, systolic blood pressure.

Compared with included participants, excluded participants were slightly older, more likely to be male and of a nonwhite ethnicity, more likely to have low maternal education, and more likely to smoke and consume alcohol weekly at age 15y (all in S1 Table). FMI did not differ at age 15y between included and excluded participants. Excluded participants had slightly more adverse insulin and inflammatory profiles, and slightly higher total activity yet lower sedentary time at age 12y, 14y, and 15y.

### Correlations between repeated measures of physical activity and sedentary time

Among 1,826 eligible participants, CPM measured at age 12y was low-to-moderately correlated with CPM measured at age 15y (Pearson *r* = 0.40), likewise when separated by sex (*r* = 0.34 for males, *r* = 0.36 for females). MVPA at age 12y was also low-to-moderately correlated with its measure at age 15y (*r* = 0.39 among all participants; 0.30 versus 0.36 among males and females, respectively). Sedentary time at age 12y was correlated with its measure at age 15y at 0.32 among all participants; this was higher among males than females (0.38 versus 0.25). Correlations were slightly larger between the earlier two measures (age 12y and 14y) at 0.45 for CPM, 0.41 for MVPA, and 0.41 for sedentary time with a slighter sex disparity for sedentary time (0.42 for males, 0.38 for females). Magnitudes were similar for the latter two measures (age 14y and 15y) at 0.43 for CPM, 0.45 for MVPA, and 0.38 for sedentary time with a higher sex disparity for both MVPA (0.38 for males, 0.44 for females) and sedentary time (0.42 for males, 0.32 for females). All coefficients were *P* < 0.001.

### Current physical activity and sedentary time (at age 15y) in relation to metabolic traits at age 15y

**Fig 2:**
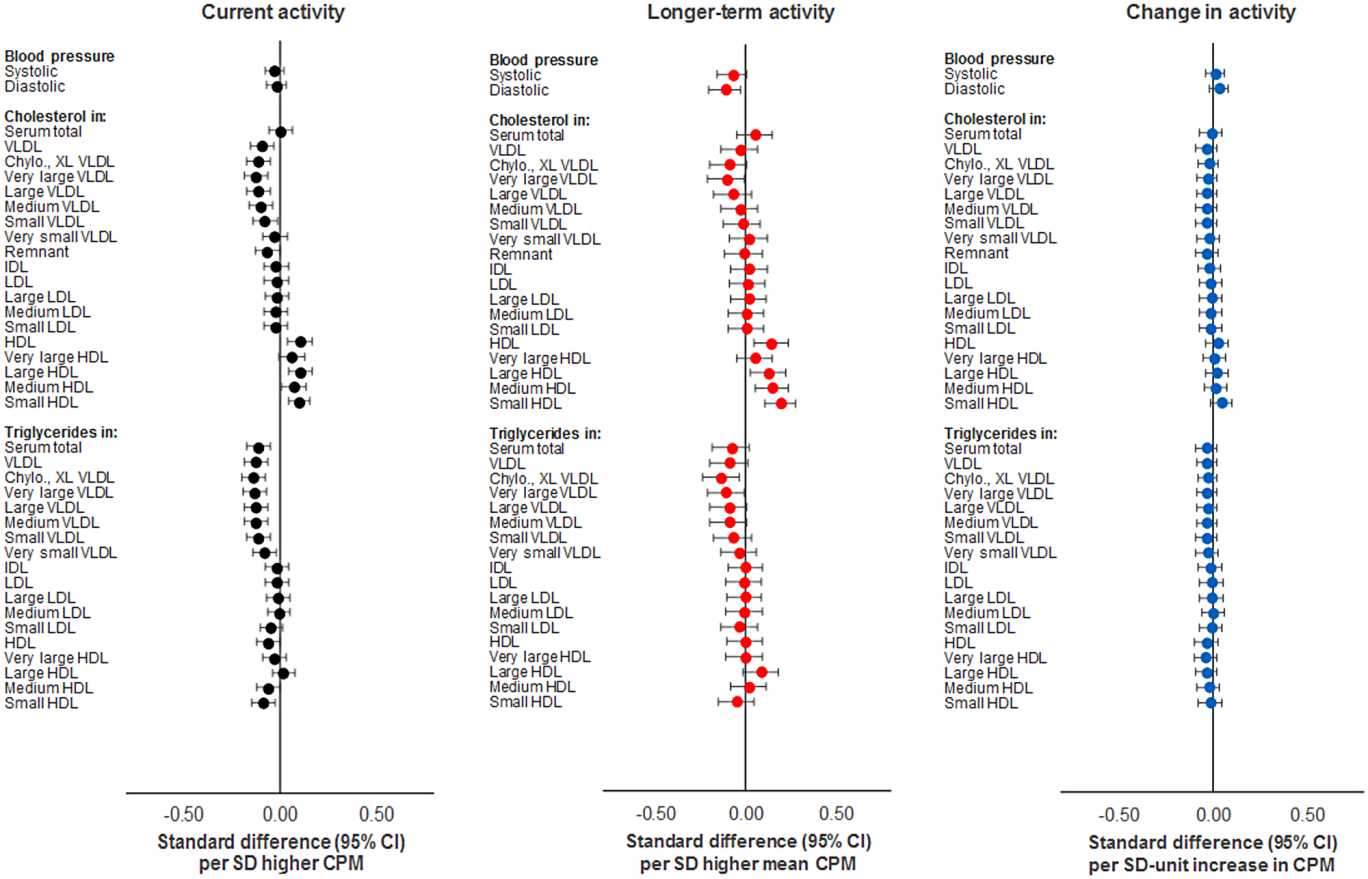
Associations of total activity (CPM) with blood pressure, cholesterol, and triglycerides at age 15y in ALSPAC. Metabolic traits are in standardised units. **Current activity** defined as measure at age 15y. **Longer-term activity** defined as mean of 3 measures (at age 12y, 14y, and 15y). **Change in activity** defined as difference between measures at age 12y and 15y. Models are adjusted for age, sex, ethnicity, maternal education, device wear time and wear month, smoking and alcohol (for current activity), and fat mass index (based on current, mean, or change values) across measures at age 12y, 14y, and 15y. ALSPAC, Avon Longitudinal Study of Parents and Children; CPM, counts per minute.

**Fig 3:**
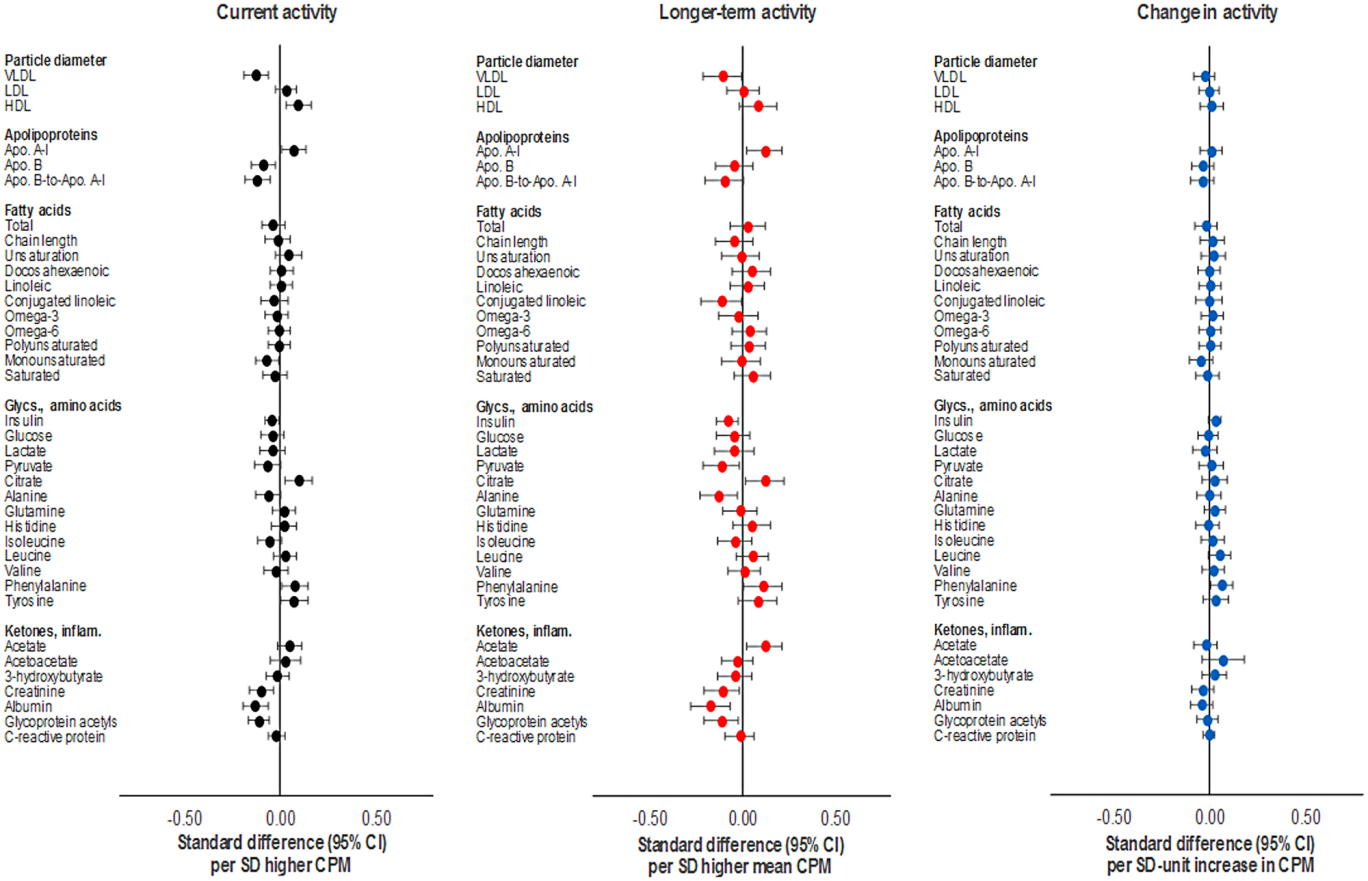
Associations of total activity (CPM) with lipoprotein particle diameter, apolipoproteins, fatty acids, glycolysis factors, amino acids, ketones, and inflammatory factors at age 15y in ALSPAC. Metabolic traits are in standardised units. **Current activity** defined as measure at age 15y. **Longer-term activity** defined as mean of 3 measures (at age 12y, 14y, and 15y). **Change in activity** defined as difference between measures at age 12y and 15y. Models are adjusted for age, sex, ethnicity, maternal education, device wear time and wear month, smoking and alcohol (for current activity), and fat mass index (based on current, mean, or change values) across measures at age 12y, 14y, and 15y. ALSPAC, Avon Longitudinal Study of Parents and Children; CPM, counts per minute.

Total activity (per SD [164 CPM] higher) was associated with numerous metabolic traits at age 15y (Figs 2 and 3; full results in S2 Table). Associations were weak with SBP and DBP but stronger with very low-density lipoprotein (VLDL) particle concentrations and lipid fractions (e.g., −1.19 × 10^−11^ mmol/l or −0.17 SD units; 95% CI = −0.24, −0.11; *P* = 1.78 × 10^−7^ for concentration of chylomicrons and extremely large very low-density lipoprotein [XL VLDL] particles). Associations were weaker with intermediate-density lipoprotein (IDL) and low-density lipoprotein (LDL) but stronger with high-density lipoprotein (HDL) particles, particularly large HDL. Higher total activity was associated with lower total VLDL cholesterol (−0.02 mmol/l or −0.13 SD units; 95% CI = −0.20, −0.07; *P* = 5.13 × 10^−5^) and higher total HDL cholesterol (0.03 mmol/l or 0.13 SD units; 95% CI = 0.07, 0.20; *P* = 3.80 × 10^−5^) but was unassociated with total LDL cholesterol (−0.007 mmol/l or −0.02 SD units; 95% CI = −0.09, 0.04; *P* = 0.44). This pattern was observed for VLDL, HDL, and LDL particle diameter. Lower overall triglyceride concentrations were observed with higher total activity, as well as triglycerides within VLDL and HDL but not LDL particles. Strong associations were also observed with apolipoprotein B, with apolipoprotein B to A1 ratio, with omega 6 to total and polyunsaturated to total fatty acid ratios, and with glycoprotein acetyls. Effect size attenuations across traits were modest upon adjustment for FMI (e.g., from −0.16 to −0.11 SD units for glycoprotein acetyls).

**Fig 4:**
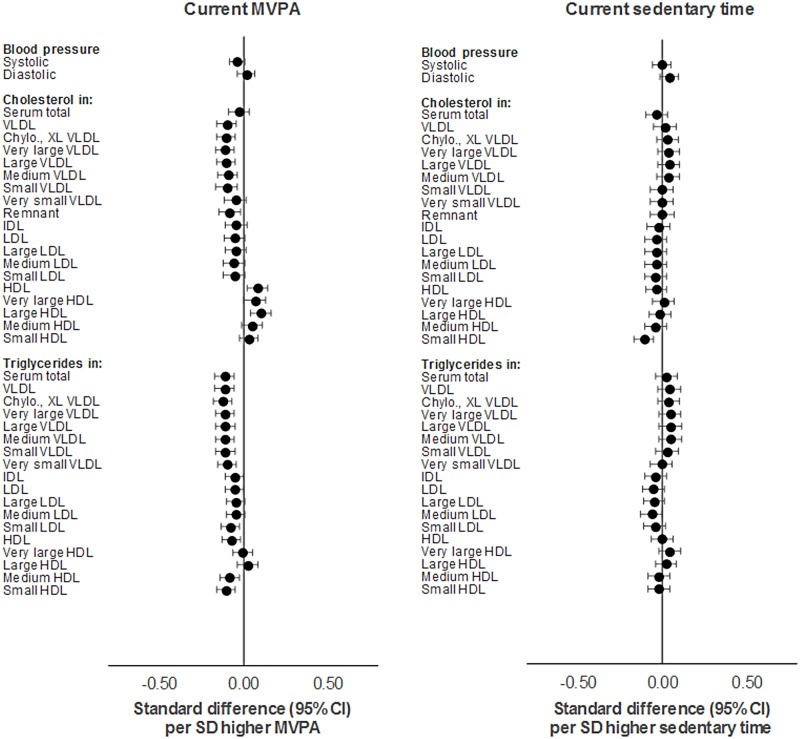
Mutually adjusted associations of current MVPA and sedentary time with blood pressure, cholesterol, and triglycerides at age 15y in ALSPAC. Metabolic traits are in standardised units. **Current MVPA/sedentary time** defined as measure at age 15y. Models are adjusted for age, sex, ethnicity, maternal education, device wear time and wear month, smoking, alcohol, alternative measure (MVPA or sedentary time), and fat mass index at age 15y. ALSPAC, Avon Longitudinal Study of Parents and Children; MVPA, moderate-to-vigorous physical activity.

**Fig 5:**
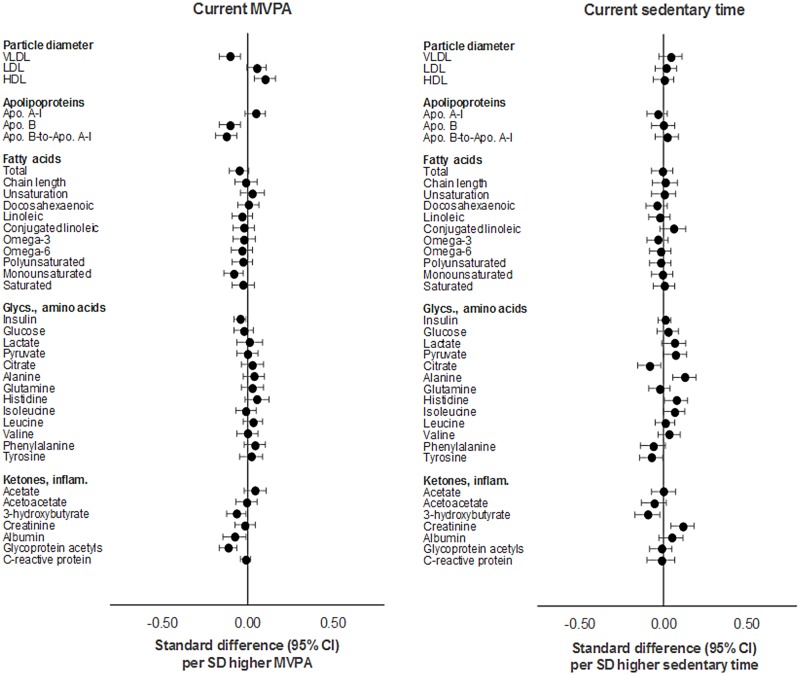
Mutually adjusted associations of current MVPA and sedentary time with lipoprotein particle diameter, apolipoproteins, fatty acids, glycolysis factors, amino acids, ketones, and inflammatory factors at age 15y in ALSPAC. Metabolic traits are in standardised units. **Current MVPA/sedentary time** defined as measure at age 15y. Models are adjusted for age, sex, ethnicity, maternal education, device wear time and wear month, smoking, alcohol, alternative measure (MVPA or sedentary time), and fat mass index at age 15y. ALSPAC, Avon Longitudinal Study of Parents and Children; MVPA, moderate-to-vigorous physical activity.

Similar association patterns and effect sizes were observed across traits with current MVPA (per SD [18 min/d] higher) as with total activity. The strongest associations were observed with cholesterol and triglycerides in VLDL and HDL particles; with diameter of VLDL and HDL particles; and with apolipoproteins and glycoprotein acetyls (S3 Table). Attenuations were minimal with adjustment for sedentary time and modest with further adjustment for FMI (e.g., from −0.15 to −0.16 to −0.11 SD units for glycoprotein acetyls). Sedentary time (per SD [66 min/d] higher) showed comparatively weaker associations with most traits; these had opposing directions of effect, particularly for cholesterol and triglycerides in VLDL and HDL particles (full results in S4 Table). Associations were positive with DBP and with VLDL particle concentrations and fractions (e.g., 5.62 × 10^−12^ mmol/l or 0.08 SD units; 95% CI = 0.02, 0.15; *P* = 0.01 for concentration of chylomicrons and XL VLDL); negative with HDL-related traits, particularly small HDL; and nearly zero with IDL- and LDL-related traits. Stronger associations were observed with amino acids (e.g., alanine at 0.01 mmol/l or 0.12 SD units; 95% CI = 0.05, 0.18; *P* = 7.32 × 10^−4^). With adjustment for MVPA, effect size attenuation was substantial across VLDL-related traits but slight to nil for DBP, small HDL traits, and alanine. These effect sizes were not much attenuated with further adjustment for FMI. These results are shown in Figs 4 and 5.

### Longer-term physical activity and sedentary time (mean across age 12y, 14y, and 15y) in relation to metabolic traits at age 15y

Based on 3 measures, total activity (per SD [132 CPM] higher) was similarly associated with most metabolic traits at age 15y versus total activity based on a single measure at age 15y (Figs 2 and 3; full results in S5 Table). Effect sizes increased for several traits, particularly SBP (from −0.05 to −0.13 SD units), HDL particle concentrations and fractions, total VLDL, LDL, and HDL cholesterol, insulin (from −0.09 to −0.14 SD units), and inflammatory glycoprotein acetyls (from −0.16 to −0.20 SD units). Attenuation of these upon adjustment for FMI based on 3 measures was more substantial versus by FMI at age 15y only in previous analyses. For example, SBP attenuated from −0.13 to −0.08 SD units and glycoprotein acetyls from −0.20 to −0.11 SD units.

**Fig 6:**
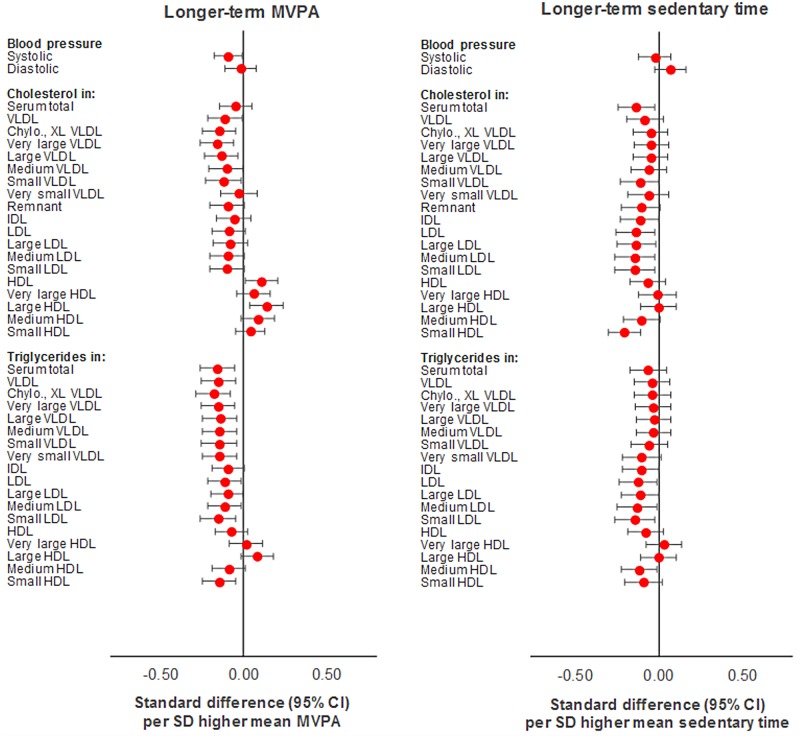
Mutually adjusted associations of longer-term MVPA and sedentary time with blood pressure, cholesterol, and triglycerides at age 15y in ALSPAC. Metabolic traits are in standardised units. **Longer-term MVPA/sedentary time** defined as mean of 3 measures (at age 12y, 14y, and 15y). Models are adjusted for age, sex, ethnicity, maternal education, device wear time and wear month, alternative measure (MVPA or sedentary time) based on mean across 3 measures, and fat mass index based on mean across 3 measures. ALSPAC, Avon Longitudinal Study of Parents and Children; MVPA, moderate-to-vigorous physical activity.

**Fig 7:**
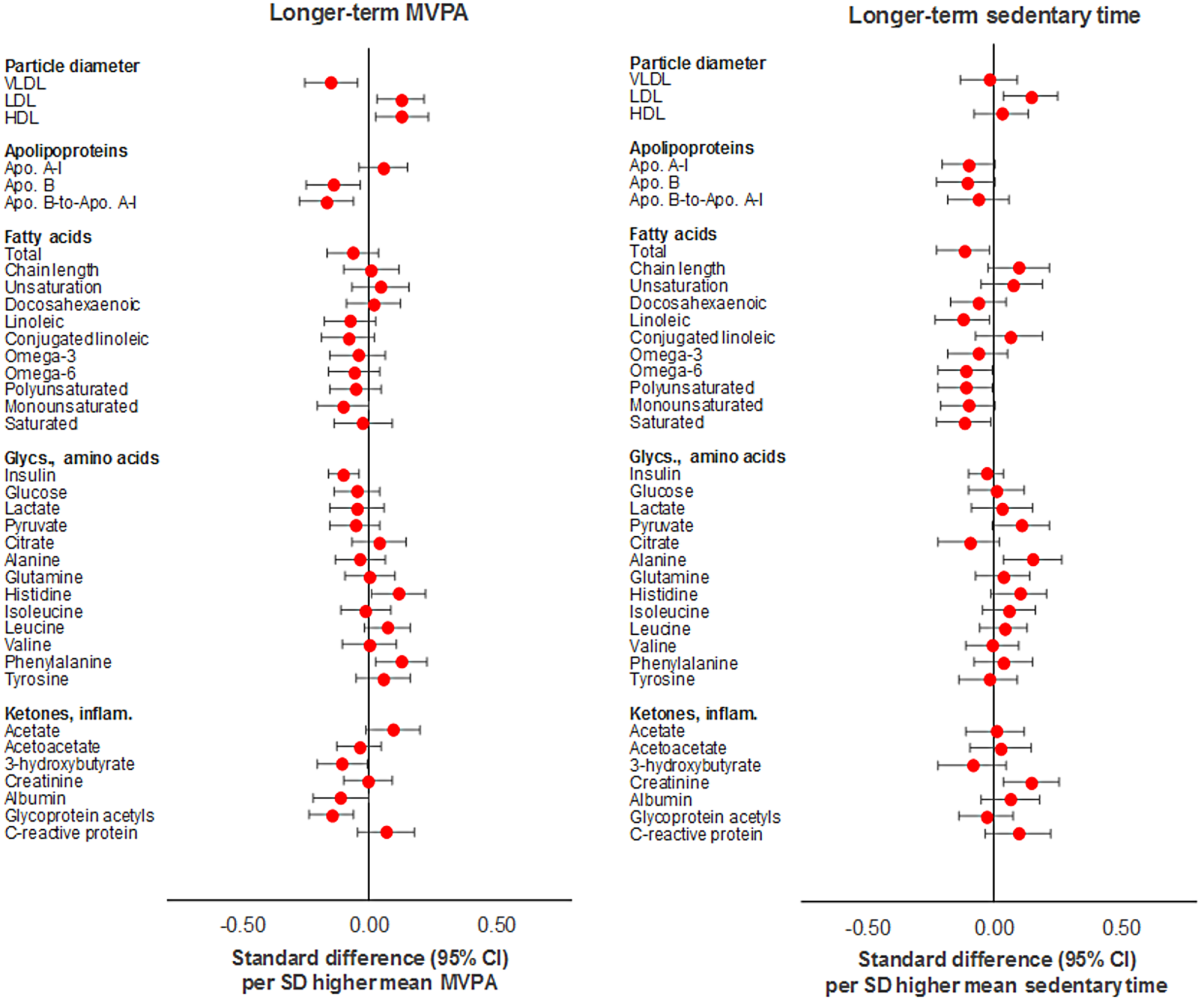
Mutually adjusted associations of longer-term MVPA and sedentary time with lipoprotein particle diameter, apolipoproteins, fatty acids, glycolysis factors, amino acids, ketones, and inflammatory factors at age 15y in ALSPAC. Metabolic traits are in standardised units. **Longer-term MVPA/sedentary time** defined as mean of 3 measures (at age 12y, 14y, and 15y). Models are adjusted for age, sex, ethnicity, maternal education, device wear time and wear month, alternative measure (MVPA or sedentary time) based on mean across 3 measures, and fat mass index based on mean across 3 measures. ALSPAC, Avon Longitudinal Study of Parents and Children; MVPA, moderate-to-vigorous physical activity.

MVPA (per SD [12 min/d] based on 3 measures) was also similarly associated with metabolic traits versus MVPA based on a single measure (S6 Table). Effect sizes increased for several traits including SBP (from −0.06 to −0.14 SD units), HDL-related traits, insulin (from −0.08 to −0.14 SD units), and glycoprotein acetyls (from −0.15 to −0.21 SD units). Attenuations were slight with adjustment for sedentary time based on 3 measures and modest with adjustment for FMI based on 3 measures (e.g. from −0.21 to −0.15 SD units for glycoprotein acetyls). Sedentary time (per SD [50 min/d] higher based on 3 measures) appeared more strongly associated with most metabolic traits versus sedentary time based on a single measure (S7 Table), with several effect sizes having increased, particularly for small HDL-related traits, pyruvate (from 0.08 to 0.15 SD units), and alanine. Attenuations were slight to nil with adjustment for MVPA and for FMI, each based on 3 measures (e.g., from −0.23 to −0.21 to −0.20 SD units for total cholesterol in small HDL). The direction of effect size appeared to flip for cholesterol and triglycerides in VLDL and LDL particles, while the direction of effect remained consistently inverse with cholesterol and triglycerides in HDL particles, particularly medium and small HDL. These are shown in Figs 6 and 7.

*P* values for an interaction term between total activity at age 15y and the mean of its two historical measures (at ages 12y and 14y) in relation to metabolic traits at age 15y ranged from *P* = 0.003 to *P* = 1.00 with a median *P* = 0.26 (S8 Table). Likewise, *P* values for an interaction term between MVPA and the mean of its two historical measures in relation to metabolic traits were high (ranging from *P* = 0.05 to *P* = 1.00; median *P* = 0.58). *P* values for an interaction term between current and historical sedentary measures in relation to metabolic traits ranged from *P* = 3.95 × 10^−4^ to *P* = 1.00 (median *P* = 0.36). The magnitude of interaction term *P* values for total activity, MVPA, and sedentary time were also inconsistent in relation to each metabolic trait and interaction term coefficients were largely in a positive direction. Together, this provided little evidence that associations of current activity with most metabolic traits differ according to previous activity.

### Change in physical activity and sedentary time (from age 12y–15y) in relation to metabolic traits at age 15y

Increased total activity from age 12y–15y (per SD unit over mean 3.7y) was weakly associated with most metabolic traits at age 15y in largely consistent directions as in previous analyses (Figs 2 and 3; full results in S9 Table). Associations were most consistent across remnant, VLDL-, and HDL-related traits (e.g., −3.51 × 10^−12^ mmol/l or −0.05 SD units; 95% CI = −0.10, 0.00; *P* = 0.05 for concentration of chylomicrons and XL VLDL particles with change in CPM). Attenuations were slight with adjustment for FMI change.

**Fig 8:**
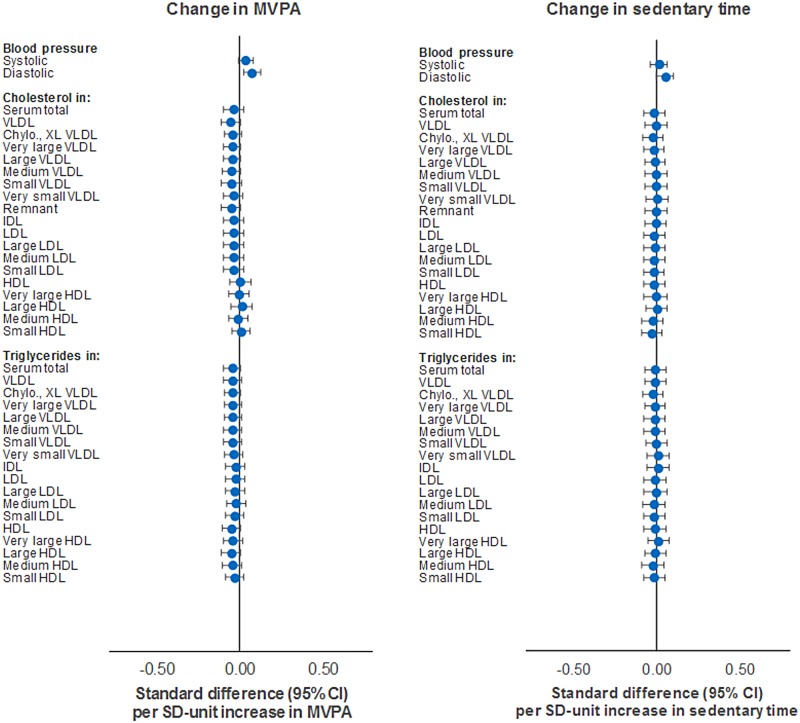
Mutually adjusted associations of change in MVPA and sedentary time with blood pressure, cholesterol, and triglycerides at age 15y in ALSPAC. Metabolic traits are in standardised units. **Change in MVPA/sedentary time** defined as difference between measures at age 12y and 15y. Models are adjusted for age, sex, ethnicity, maternal education, device wear time and wear month, alternative measure (MVPA or sedentary time), and fat mass index based on change values. ALSPAC, Avon Longitudinal Study of Parents and Children; MVPA, moderate-to-vigorous physical activity.

**Fig 9:**
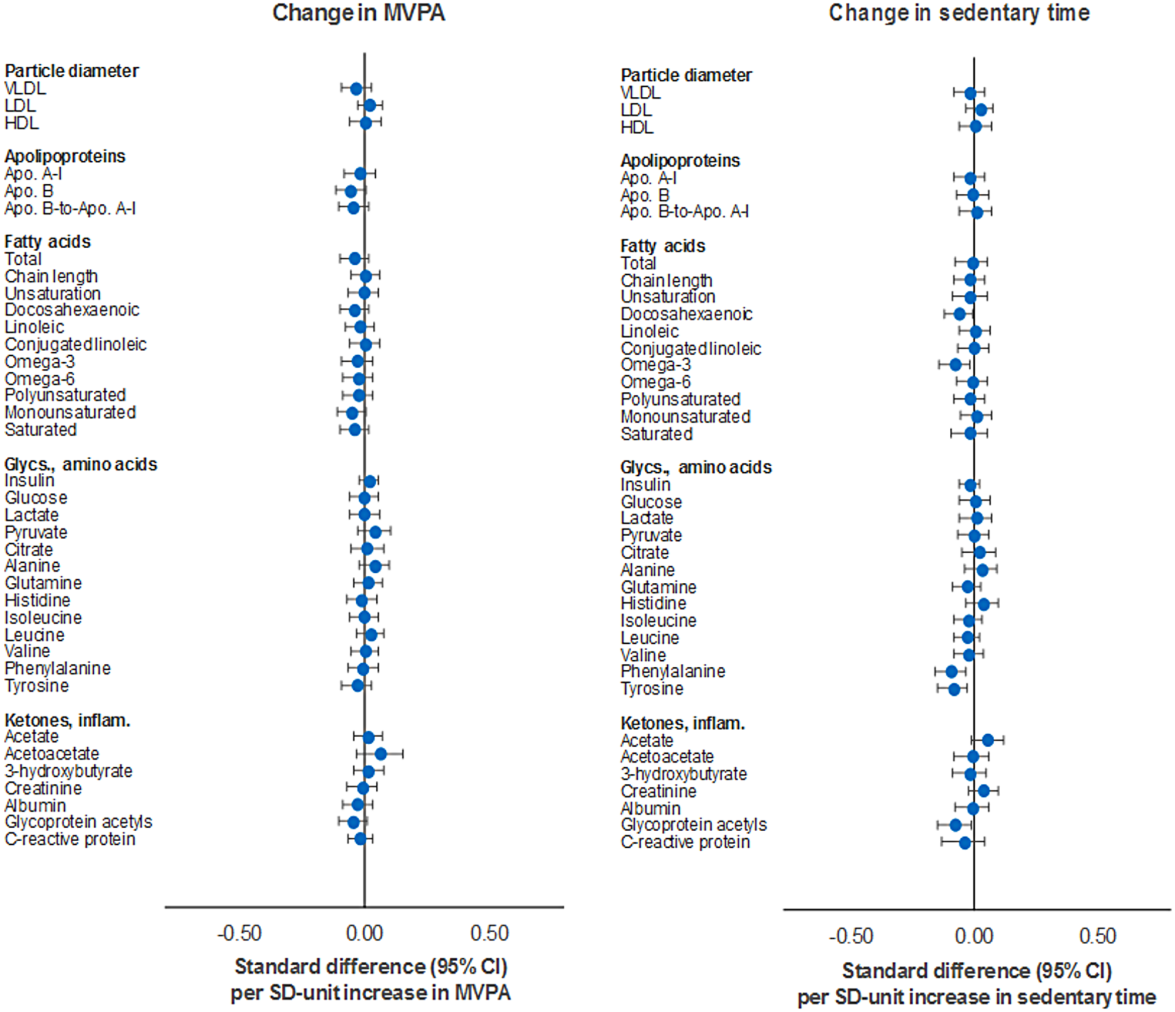
Mutually adjusted associations of change in MVPA and sedentary time with lipoprotein particle diameter, apolipoproteins, fatty acids, glycolysis factors, amino acids, ketones, and inflammatory factors at age 15y in ALSPAC. Metabolic traits are in standardised units. **Change in MVPA/sedentary time** defined as difference between measures at age 12y and 15y. Models are adjusted for age, sex, ethnicity, maternal education, device wear time and wear month, alternative measure (MVPA or sedentary time), and fat mass index based on change values. ALSPAC, Avon Longitudinal Study of Parents and Children; MVPA, moderate-to-vigorous physical activity.

Increased MVPA from age 12y–15y was weakly associated with metabolic traits at age 15y (Figs 8 and 9; S10 Table). Effect sizes were small but directionally concordant with those seen for current and longer-term MVPA measures in relation to cholesterol in VLDL and LDL particles and triglycerides across all particle types, while SBP and DBP appeared to flip their direction of effect. Cholesterol in HDL particles appeared most unassociated, in contrast to previous MVPA measures (current and longer term). Increased sedentary time was largely unassociated with cholesterol and triglycerides across all particle types, while stronger evidence of association was observed for the amino acids phenylalanine (−0.001 mmol/l or −0.09 SD units; 95% CI = −0.15, −0.03; *P* = 0.003) and tyrosine (−0.001 mmol/l or −0.06 SD units; 95% CI = −0.12, 0.00; *P* = 0.05). These were unaltered by adjustment for MVPA or FMI (full results in S11 Table).

### Supplementary results

Upon further adjustment for pre-baseline metabolic trait values measured at age 8y, attenuations of associations of current (15y) total activity, MVPA, and sedentary time with most metabolic traits at age 15y were minimal (all in S12 Table). For example, the pre-adjusted association estimate of higher total activity with lower triglycerides in VLDL particles at age 15y (−0.03 mmol/l or −0.12 SD units; 95% CI = −0.18, −0.06; *P* = 1.06 × 10^−4^) was essentially unchanged after adjusting for triglycerides in VLDL particles at age 8y (−0.11 SD units; 95% CI = −0.17, −0.04; *P* = 1.36 × 10^−3^). The same pattern was apparent for models of longer-term total activity, MVPA, and sedentary time (S13 Table) and of change in these (S14 Table).

Associations of activity and sedentary time in relation to FMI at age 15y as an outcome are shown in S15 Table. Higher current activity (at 15y) was associated with −0.39 kg/m^2^ (or −0.12 SD units of FMI; 95% CI = −0.17, −0.08; *P* = 2.96 × 10^−7^). Upon adjustment for a pre-baseline measure of FMI at age 10y, this attenuated to −0.13 kg/m^2^ (or −0.04 SD units; 95% CI = −0.07, −0.01; *P* = 0.01). A similar magnitude of association and attenuation was seen for MVPA. Associations and their attenuations were more substantial with activity measures based on 3 occasions. For example, higher longer-term total activity was associated with −0.72 kg/m^2^ (or −0.22 SD units of FMI; 95% CI = −0.28, −0.15; *P* = 3.32 × 10^−10^) at age 15y, and this attenuated to −0.16 kg/m^2^ (or −0.05 SD units; 95% CI = −0.10, −0.01; *P* = 0.03) upon adjustment for FMI at 10y. Current and longer-term sedentary time were largely unassociated with FMI at age 15y (likewise for change in activity and sedentary values).

The pattern and relative magnitude of associations of physical activity and sedentary time with metabolic traits at age 15y were similar for each above analysis based on a complete case sample of 755 participants (S2–S15 Tables).

## Discussion

This study aimed to better describe associations of physical activity and sedentary time with metabolic traits by integrating repeat accelerometry data taken several years apart with data from targeted metabolomics. At age 15y, total activity was associated with a diverse set of metabolic traits. Associations were strongest between higher-intensity activity and lower cholesterol in VLDL lipoprotein particles, higher cholesterol in HDL particles, lower triglyceride in both HDL and VLDL particles, and lower inflammatory glycoprotein acetyls, although association magnitude was generally small. Associations appeared more robust for higher MVPA than for lower sedentary time and were weak in relation to change in all activity types over several years. Activity fluctuates over time, but associations of current activity with most metabolic traits did not differ by previous activity. This suggests that the metabolic effects of physical activity, if causal, depend on most recent engagement.

In this study, higher current activity based on one accelerometry occasion and higher longer-term activity based on the mean of three separate accelerometry occasions were similarly associated with lower cholesterol in, and diameter of, VLDL particles; higher cholesterol in, and diameter of, HDL particles; lower triglycerides in VLDL and HDL particles; and with inflammatory traits, among others. Sedentary time was most associated with lower VLDL lipid fractions, lower cholesterol in small HDL particles, and several amino acids; associations were not directionally consistent with cholesterol and triglycerides across VLDL and LDL particles across sedentary time measures, however, suggesting these were less robust and more prone to confounding. Confounders could be socioeconomic given that socioeconomic advantage may lead children to accumulate a higher total sitting time from academic activities like studying yet also to better overall health [36]. This could induce associations between higher sitting time and favourable adiposity-driven lipid profiles. Differential measurement error of MVPA and sedentary time by the waist-worn device could also help explain unexpected directions of association, since light-intensity physical activities like standing could be misclassified as sedentary time [37,38]. The magnitude of associations of total activity and MVPA with metabolic traits were similar—as expected, given that CPM describes intensity—and were in agreement with previous findings based on summary metabolic traits among adolescents [5]. However, associations of change in activity over several years were weak in relation to most metabolic traits; this was the case for both MVPA and sedentary time.

To our knowledge, only one previous study used targeted NMR metabolomics to describe associations of physical activity with metabolic traits [17]. That study pooled data from three cohorts of adults each with two occasions of self-reported activity and observed similar associations of higher activity with lower cholesterol in VLDL and remnant lipoproteins, higher cholesterol in HDL, lower triglycerides across all lipoproteins, and lower inflammatory glycoproteins, among others. Associations were generally stronger and effect sizes larger for most traits (at about 0.5 SD units) compared with our study, and higher activity was more strongly associated with lower cholesterol in LDL particles, whereas these were notably weak here. Whether associations with LDL cholesterol were underestimated here due to smaller sample size or were more realistically estimated due to enhanced activity measurement with accelerometry is unclear. Weaker associations here may also reflect a general lack of metabolic disturbances and a healthier overall metabolism given the younger age of participants examined versus the previous study, in which mean age across cohorts was 31y–52y. This is also likely to be the case given lower adiposity in the present study (mean BMI of 21.4 kg/m^2^) versus the previous study (in which mean BMI was 24.6–27.1 kg/m^2^ across cohorts).

Physical activity is commonly thought to protect against cardiovascular disease [4], but direct causal evidence from randomised controlled trials and other studies designed to strengthen causal inference is sparse [39,40]. Higher cholesterol content in LDL and remnant lipoproteins and higher total triglycerides are supported as causes of CHD through Mendelian randomisation studies [41,42] and randomised controlled trials [43,44]. Lower HDL cholesterol is not supported as a cause of CHD by the same standard of evidence [41,45]. In this study, both physical activity and sedentary time were more strongly associated with the concentration, diameter, and cholesterol content of HDL particles than of LDL particles. Higher activity was, however, also associated with cholesterol and triglyceride content in VLDL particles, a known LDL precursor. Together, this suggests that any protective effects of physical activity for CHD risk via lipid pathways likely operate through triglycerides and remnant cholesterol content, not LDL cholesterol itself. Higher activity was also associated with lower glycoprotein acetyls, which are inflammatory acute-phase reactants associated with future cardiovascular events [46] and closely related to atherosclerotic inflammatory interleukins [47,48]. Associations with inflammatory markers were more reliably driven by higher MVPA than lower sedentary time duration.

Our results also suggest that associations of current activity with most metabolic traits do not differ depending upon previous levels of activity. Blood pressure appeared to be an exception, with notably larger effect sizes based on multi-occasion assessments of activity, but statistical evidence for interaction was weak. Dependency of current activity on previous activity for metabolic trait associations have not been previously examined using device-measured activity, but numerous studies conducted on college alumni using historical activity from self-reported recall found little to no additional benefit of high previous activity (e.g., having been a college athlete) above current activity levels in relation to reduced LDL cholesterol, triglycerides [49], and coronary heart disease risk [50–52]. Mechanistically, the lipolytic and anti-inflammatory effects of physical activity are initiated through skeletal muscle contraction [53], and when contraction ceases to be frequent among previously active adults, these benefits are known to deteriorate in a matter of days, even to levels experienced among sedentary adults [54]. Together, this suggests that the cardiometabolic benefits of physical activity are likely to be acute rather than cumulative in nature, with meaningful reductions in disease risk requiring sustained activity. The same may apply to more structural changes to vasculature, which appear to improve cumulatively with continued activity but deteriorate when activity ceases [55].

Associations of activity with metabolic traits showed only modest attenuation with adjustment for objective total fat mass suggesting that confounding by adiposity does not fully explain results. Attenuations were also slight upon adjustment for these same metabolic traits measured pre-baseline in a supplementary analysis at age 8y, suggesting that reverse causation is also unlikely. Adiposity was notably low among participants, however—with a mean BMI of 21.4 kg/m^2^ and only 4.2% with obesity—which would have reduced an influence of adiposity on activity levels. Results may also be susceptible to residual confounding by aspects of adiposity not captured by a total DXA measure. Effect sizes were also small before these adjustments. Meta-analyses of observational studies indicate that obesity versus normal weight more greatly amplifies risk of type 2 diabetes than does inactivity versus high activity (a 6-fold versus 1.2-fold increase) [56]. Presently, in supplementary analyses, higher current and longer-term activity were also associated with lower DXA adiposity as an outcome at age 15y. These effect sizes were of a similarly small magnitude as the most strongly associated metabolic traits, but attenuations were more substantial upon adjustment for pre-baseline adiposity at age 10y, attenuating over 4-fold in the case of longer-term total activity. This suggests that much of the association of higher activity with lower subsequent adiposity is driven by reverse causation in this data. In line with this, a recent genome-wide association study for accelerometry physical activity in the UK Biobank cohort identified a small set of common genetic variants robustly associated with total activity, and Mendelian randomisation analyses with these variants suggested a lowering effect of total activity on fat mass and blood pressure [23]. The standardised effect size was 6 times larger in the reverse direction, however—from fat mass to inactivity—suggesting that adiposity affects activity levels more than activity levels affect adiposity. Effect sizes matter a great deal for public health messaging since the existence of an association, or indeed a causal effect, does not alone describe its importance. Future work should compare magnitudes of effect size between common risk factors as the rate of discovery and the need to prioritise limited public health resources both increase.

### Strengths and limitations

Main strengths of this study include its use of three repeat accelerometry measures of physical activity and sedentary time taken several years apart, which allowed examination of longer-term activity, interaction by previous activity, and change in activity; its use of detailed metabolic trait data from targeted metabolomics; and its use of DXA measures for adiposity adjustment. Several clinical traits (SBP, DBP, insulin, and C-reactive protein) were included alongside metabolomics-derived traits, which allowed comparison of association strength between established and novel traits. The metabolic traits examined, like all biological characteristics, are subject to within-person variability over time. The expected degree of short- and long-term stability of NMR-derived traits has not been formally estimated (e.g., via intraclass correlation coefficients), but metabolic trait concentrations quantified through NMR are known to be highly consistent with those quantified through routine clinical chemistry assays and more-specialised methods including gas chromatography (correlations commonly >0.9) [57–59]. Blood was drawn while fasting at a largely consistent time of day, limiting potential for diurnal effects on traits like inflammatory markers [60]. Participants were of a young age, which reduced potential for confounding in analyses by subclinical chronic diseases and health behaviours like smoking, which were uncommon. Young participant age may, however, limit generalisability of associations to older age groups given that self-reported activity levels tend to peak in childhood, decline at adolescence, and decline further past the age of 50y [61]. Also notable is that observations spanned puberty, which is a substantial period of growth and maturity with implications for metabolism [62,63]. Specifically, a faster rate of pubertal growth as often indicated through earlier age at menarche among females and voice breaking among males is associated with numerous metabolic traits [63] and with a higher risk of developing type 2 diabetes and coronary heart disease in adulthood [64,65]. These associations may be largely confounded by pre-pubertal adiposity, however, given that higher adiposity in childhood induces earlier puberty [66] and tracks into later life stages [67,68] and that substantial overlap exists between genetic variants associated with puberty timing and adiposity [69]. Adjusting for BMI measured before puberty onset in observational studies has substantially attenuated associations of puberty timing with postpubertal adiposity and metabolic traits [70,71]. Adjustment for objectively measured adiposity over the pubertal period, as done here, likely accounts for much of the influence of pubertal growth on subsequent metabolic trait levels while avoiding the need to additionally consider this often imprecisely measured exposure. The duration of accelerometry measurement on each occasion was relatively short (3–7 d), and although repeat measures were used, the length of time between the first and last accelerometry measure was also relatively short, at about 3.7y. Longer time durations within and between activity assessments would make us better able to distinguish biological patterns from chance variation, especially in analyses of interaction and change. A simple mean of three measurement occasions was used to estimate longer-term activity and sedentary time across these time periods, which does not describe different trajectories of activity; the short time span between measures and the large scale of outcome data limits such approaches. Activity was recorded using uniaxial accelerometers, which predated more sensitive triaxial devices; future studies could expand upon metabolic trait associations using repeated measures of triaxial devices with longer durations.

### Conclusions

Our results support associations of physical activity with metabolic traits that are small in magnitude and more robust for higher MVPA than lower sedentary time. Activity was most strongly associated with cholesterol content in VLDL and HDL lipoprotein particles, with triglyceride content in all particle types, and with inflammatory glycoprotein acetyls. Associations were weaker in relation to change in activity over several years—likewise for MVPA and sedentary time. Activity fluctuates over time, but associations of current activity with most metabolic traits did not differ by previous activity. This suggests that the metabolic effects of physical activity, if causal, depend on most recent engagement.

## Supporting information

S1 Table Characteristics of included and excluded participants in ALSPAC. ALSPAC, Avon Longitudinal Study of Parents and Children.
(PDF)

S2 Table Associations of current total physical activity (CPM at age 15y) with metabolic traits at age 15y in ALSPAC. ALSPAC, Avon Longitudinal Study of Parents and Children; CPM, counts per minute.
(PDF)

S3 Table Associations of current moderate-to-vigorous physical activity (MVPA at age 15y) with metabolic traits at age 15y in ALSPAC. ALSPAC, Avon Longitudinal Study of Parents and Children; MVPA, moderate-to-vigorous physical activity.
(PDF)

S4 Table Associations of current sedentary time (SED at age 15y) with metabolic traits at age 15y in ALSPAC. ALSPAC, Avon Longitudinal Study of Parents and Children; SED, sedentary time.
(PDF)

S5 Table Associations of longer-term total physical activity (mean of CPM measures at age 12y, 14y, and 15y) with metabolic traits at age 15y in ALSPAC. ALSPAC, Avon Longitudinal Study of Parents and Children; CPM, counts per minute.
(PDF)

S6 Table Associations of longer-term moderate-to-vigorous physical activity (mean of MVPA measures at age 12y, 14y and 15y) with metabolic traits at age 15y in ALSPAC. ALSPAC, Avon Longitudinal Study of Parents and Children; MVPA, moderate-to-vigorous physical activity.
(PDF)

S7 Table Associations of longer-term sedentary time (mean of SED measures at age 12y, 14y, and 15y) with metabolic traits at age 15y in ALSPAC. ALSPAC, Avon Longitudinal Study of Parents and Children; SED, sedentary time.
(PDF)

S8 Table Interactions between current activity (measures at age 15y) with historical physical activity and sedentary time (mean of measures at age 12y and 14y) in relation to metabolic traits at age 15y in ALSPAC. ALSPAC, Avon Longitudinal Study of Parents and Children.
(PDF)

S9 Table Associations of change in total physical activity (CPM change from age 12y-15y) with metabolic traits at age 15y in ALSPAC. ALSPAC, Avon Longitudinal Study of Parents and Children; CPM, counts per minute.
(PDF)

S10 Table Associations of change in moderate-to-vigorous physical activity (MVPA change from age 12y-15y) with metabolic traits at age 15y in ALSPAC. ALSPAC, Avon Longitudinal Study of Parents and Children; MVPA, moderate-to-vigorous physical activity.
(PDF)

S11 Table Associations of change in sedentary time (SED change from age 12y-15y) with metabolic traits at age 15y in ALSPAC. ALSPAC, Avon Longitudinal Study of Parents and Children; SED, sedentary time.
(PDF)

S12 Table Associations of current physical activity and sedentary time (measures at age 15y) with metabolic traits at age 15y, with adjustment for metabolic traits at age 8y in ALSPAC. ALSPAC, Avon Longitudinal Study of Parents and Children.
(PDF)

S13 Table Associations of longer-term physical activity and sedentary time (mean of measures at age 12y, 14y, and 15y) with metabolic traits at age 15y, with adjustment for metabolic traits at age 8y in ALSPAC. ALSPAC, Avon Longitudinal Study of Parents and Children.
(PDF)

S14 Table Associations of change in physical activity and sedentary time (change in measures from age 12y–15y) with metabolic traits at age 15y, with adjustment for metabolic traits at age 8y in ALSPAC. ALSPAC, Avon Longitudinal Study of Parents and Children.
(PDF)

S15 Table Associations of physical activity and sedentary time with adiposity at age 15y in ALSPAC. ALSPAC, Avon Longitudinal Study of Parents and Children.
(PDF)

S1 Protocol Prespecified study protocol. (PDF)

S1 STROBE Checklist STROBE statement. STROBE, Strengthening the Reporting of Observational Studies in Epidemiology.
(PDF)

## Abbreviations

ALSPAC: Avon Longitudinal Study of Parents and Children
BMI: body mass index
CHD: coronary heart disease
CPM: counts per minute
CSE: Certificate of Secondary Education
DBP: diastolic blood pressure
DXA: dual-energy X-ray absorptiometry
FMI: fat mass index
HDL: high-density lipoprotein
IDL: intermediate-density lipoprotein
LDL: low-density lipoprotein
MVPA: moderate-to-vigorous physical activity
NMR: nuclear magnetic resonance
SBP: systolic blood pressure
STROBE: Strengthening the Reporting of Observational Studies in Epidemiology
VLDL: very low-density lipoprotein
XL VLDL: extremely large very low-density lipoprotein
