# Supplementary material for: Associations of device-measured physical activity across adolescence with metabolic traits: Prospective cohort study
Source: PLoS Med. 2018 Sep 11;15(9):e1002649. doi: 10.1371/journal.pmed.1002649 (PMC6133272; doi:10.1371/journal.pmed.1002649)
Supplement: S1 Table — ALSPAC, Avon Longitudinal Study of Parents and Children. (PDF) [file pmed.1002649.s001.pdf]

S1 Table Characteristics of included and excluded participants in ALSPAC

		Included		Excluded	*P-value for difference*
	**n**		**n**		
Demographics					
Age (years) at 15y clinic – mean (SD)	1826	15.4 (0.2)	3680	15.5 (0.4)	1.00E-41
Female – n (%)	1826	55.6 (1015)	13028	47.6 (6204)	2.03E-10
Non-white ethnicity – n (%)	1826	3.6 (66)	10324	5.3 (547)	0.003
Low maternal education* – n (%)	1826	50.5 (922)	10667	67.1 (7161)	<1.00E-41
Smokes weekly - n (%)	1826	4.9 (89)	3532	13.1 (467)	<1.00E-41
Consumes alcohol weekly - n (%)	1826	15.3 (280)	3524	25.0 (882)	6.66E-16
Fat mass index (kg/m^2) – mean (SD)	1794	5.6 (3.3)	3352	5.4 (3.3)	0.073
Activity trait at age 12y					
Total (counts/min) – mean (SD)	1612	586.0 (168.3)	4338	613.1 (186.6)	9.48E-08
Duration of moderate-to-vigorous (min/day) – mean (SD)	1612	21.7 (14.0)	4338	23.6 (16.0)	8.72E-06
Duration of sedentary (min/day) – mean (SD)	1612	434.5 (64.7)	4338	422.9 (69.8)	2.07E-09
Activity trait at age 14y					
Total (counts/min) – mean (SD)	1560	528.1 (177.9)	2764	550.5 (199.9)	1.46E-04
Duration of moderate-to-vigorous (min/day) – mean (SD)	1560	23.3 (16.2)	2764	24.4 (17.9)	0.041
Duration of sedentary (min/day) – mean (SD)	1560	489.4 (68.1)	2764	476.2 (76.5)	5.20E-09
Activity trait at age 15y					
Total (counts/min) – mean (SD)	1826	477.5 (164.0)	533	490.2 (222.8)	0.220
Duration of moderate-to-vigorous (min/day) – mean (SD)	1826	23.6 (17.9)	533	22.9 (21.1)	0.485
Duration of sedentary (min/day) – mean (SD)	1826	522.1 (66.0)	533	501.7 (90.6)	1.38E-06
Clinical characteristics at age 15y					
Systolic blood pressure (mmHg) – mean (SD)	1812	123.1 (10.4)	3498	122.9 (11.1)	0.507
Diastolic blood pressure (mmHg) – mean (SD)	1812	67.5 (8.5)	3498	67.4 (8.9)	0.467
Total cholesterol (mmol/l) – mean (SD)	1207	3.6 (0.6)	2159	3.5 (0.6)	0.029
LDL cholesterol (mmol/l) – mean (SD)	1207	1.1 (0.3)	2159	1.0 (0.3)	0.083
HDL cholesterol (mmol/l) – mean (SD)	1207	1.4 (0.2)	2159	1.4 (0.2)	0.002
Triglycerides (mmol/l) – mean (SD)	1207	0.9 (0.3)	2159	0.9 (0.3)	0.490
Insulin (mu/l) - median (range)	1253	8.9 (1.2 – 39.6)	2231	9.2 (0.6 - 122.0)	1.76E-08
Glucose (mmol/l) – mean (SD)	1204	4.3 (0.3)	2155	4.3 (0.4)	0.110
Glycoprotein acetyls (mmol/l) – mean (SD)	1206	1.2 (0.1)	2157	1.2 (0.1)	3.13E-04
C-reactive protein (mg/l) - median (range)	1255	0.4 (0.1 – 72.6)	2233	0.4 (0.07 - 71.34)	0.003

P-values are based on linear or logistic regression models.
*'Low maternal education' defined as highest level of education attained being Certificate of Secondary Education, vocational, or O level (not A level or degree).
