# Supplementary material for: Associations of device-measured physical activity across adolescence with metabolic traits: Prospective cohort study
Source: PLoS Med. 2018 Sep 11;15(9):e1002649. doi: 10.1371/journal.pmed.1002649 (PMC6133272; doi:10.1371/journal.pmed.1002649)
Supplement: S2 Table — ALSPAC, Avon Longitudinal Study of Parents and Children; CPM, counts per minute. (PDF) [file pmed.1002649.s002.pdf]

S2 Table Associations of current total physical activity (CPM at age 15y) with metabolic traits at age 15y in ALSPAC

	CPM at age 15y (per SD (164) higher)									
	Adj. for age, sex, ethnicity, maternal education, smoking, alcohol, wear time, wear month					*Additionally adj. for FMI at age 15y*				
**Standardised outcome at age 15y**	**N**	**Beta**	**LCL**	**UCL**	**P-value**	**N**	**Beta**	**LCL**	**UCL**	**P-value**
Systolic blood pressure (mmHg)	1812	-0.05	-0.10	0.00	0.051	1782	-0.03	-0.08	0.02	0.292
Diastolic blood pressure (mmHg)	1812	-0.02	-0.07	0.03	0.384	1782	-0.02	-0.07	0.03	0.533
Concentration of chylomicrons and extremely large VLDL particles (mol/l)	1207	-0.17	-0.24	-0.11	1.78E-07	1195	-0.13	-0.19	-0.07	1.42E-05
Total lipids in chylomicrons and extremely large VLDL (mmol/l)	1207	-0.17	-0.24	-0.11	2.56E-07	1195	-0.13	-0.19	-0.07	1.81E-05
Phospholipids in chylomicrons and extremely large VLDL (mmol/l)	1207	-0.17	-0.24	-0.11	1.75E-07	1195	-0.14	-0.20	-0.07	1.22E-05
Total cholesterol in chylomicrons and extremely large VLDL (mmol/l)	1207	-0.15	-0.22	-0.09	4.62E-06	1195	-0.11	-0.17	-0.05	2.90E-04
Cholesterol esters in chylomicrons and extremely large VLDL (mmol/l)	1207	-0.13	-0.20	-0.07	1.01E-04	1195	-0.09	-0.15	-0.03	0.005
Free cholesterol in chylomicrons and extremely large VLDL (mmol/l)	1207	-0.17	-0.24	-0.11	2.54E-07	1195	-0.13	-0.19	-0.07	1.69E-05
Triglycerides in chylomicrons and extremely large VLDL (mmol/l)	1207	-0.18	-0.24	-0.11	1.52E-07	1195	-0.14	-0.20	-0.08	1.08E-05
Concentration of very large VLDL particles (mol/l)	1207	-0.17	-0.24	-0.10	4.39E-07	1195	-0.13	-0.19	-0.07	3.07E-05
Total lipids in very large VLDL (mmol/l)	1207	-0.17	-0.23	-0.10	6.96E-07	1195	-0.13	-0.19	-0.07	4.58E-05
Phospholipids in very large VLDL (mmol/l)	1207	-0.17	-0.23	-0.10	7.14E-07	1195	-0.13	-0.19	-0.07	4.43E-05
Total cholesterol in very large VLDL (mmol/l)	1207	-0.17	-0.23	-0.10	7.89E-07	1195	-0.12	-0.18	-0.06	6.27E-05
Cholesterol esters in very large VLDL (mmol/l)	1207	-0.16	-0.23	-0.10	1.37E-06	1195	-0.12	-0.18	-0.06	1.17E-04
Free cholesterol in very large VLDL (mmol/l)	1207	-0.17	-0.23	-0.10	5.32E-07	1195	-0.13	-0.19	-0.07	3.73E-05
Triglycerides in very large VLDL (mmol/l)	1207	-0.17	-0.23	-0.10	7.51E-07	1195	-0.13	-0.19	-0.07	4.63E-05
Concentration of large VLDL particles (mol/l)	1207	-0.16	-0.23	-0.10	1.59E-06	1195	-0.12	-0.18	-0.06	1.16E-04
Total lipids in large VLDL (mmol/l)	1207	-0.16	-0.23	-0.10	1.96E-06	1195	-0.12	-0.18	-0.06	1.39E-04
Phospholipids in large VLDL (mmol/l)	1207	-0.16	-0.23	-0.09	2.10E-06	1195	-0.12	-0.18	-0.06	1.50E-04
Total cholesterol in large VLDL (mmol/l)	1207	-0.15	-0.22	-0.09	5.08E-06	1195	-0.11	-0.17	-0.05	3.72E-04
Cholesterol esters in large VLDL (mmol/l)	1207	-0.15	-0.21	-0.08	1.48E-05	1195	-0.10	-0.16	-0.04	1.08E-03
Free cholesterol in large VLDL (mmol/l)	1207	-0.16	-0.23	-0.09	2.24E-06	1195	-0.12	-0.18	-0.06	1.51E-04
Triglycerides in large VLDL (mmol/l)	1207	-0.16	-0.23	-0.10	1.45E-06	1195	-0.12	-0.18	-0.06	9.92E-05
Concentration of medium VLDL particles (mol/l)	1207	-0.17	-0.23	-0.10	1.14E-06	1195	-0.12	-0.18	-0.06	1.14E-04
Total lipids in medium VLDL (mmol/l)	1207	-0.16	-0.23	-0.10	2.01E-06	1195	-0.12	-0.18	-0.06	2.00E-04
Phospholipids in medium VLDL (mmol/l)	1207	-0.16	-0.23	-0.09	2.35E-06	1195	-0.12	-0.18	-0.06	2.09E-04
Total cholesterol in medium VLDL (mmol/l)	1207	-0.14	-0.20	-0.07	3.55E-05	1195	-0.10	-0.16	-0.03	2.48E-03
Cholesterol esters in medium VLDL (mmol/l)	1207	-0.12	-0.18	-0.05	4.60E-04	1195	-0.07	-0.14	-0.01	0.020
Free cholesterol in medium VLDL (mmol/l)	1207	-0.16	-0.22	-0.09	3.39E-06	1195	-0.12	-0.18	-0.05	2.57E-04
Triglycerides in medium VLDL (mmol/l)	1207	-0.17	-0.23	-0.10	7.79E-07	1195	-0.12	-0.19	-0.06	7.80E-05
Concentration of small VLDL particles (mol/l)	1207	-0.14	-0.21	-0.08	1.47E-05	1195	-0.10	-0.16	-0.04	1.22E-03
Total lipids in small VLDL (mmol/l)	1207	-0.14	-0.21	-0.08	2.52E-05	1195	-0.10	-0.16	-0.04	2.22E-03
Phospholipids in small VLDL (mmol/l)	1207	-0.12	-0.19	-0.06	2.45E-04	1195	-0.08	-0.14	-0.02	0.015
Total cholesterol in small VLDL (mmol/l)	1207	-0.12	-0.18	-0.05	3.69E-04	1195	-0.08	-0.14	-0.01	0.017
Cholesterol esters in small VLDL (mmol/l)	1207	-0.11	-0.17	-0.05	7.87E-04	1195	-0.07	-0.14	-0.01	0.026
Free cholesterol in small VLDL (mmol/l)	1207	-0.12	-0.18	-0.05	3.22E-04	1195	-0.08	-0.14	-0.02	0.014
Triglycerides in small VLDL (mmol/l)	1207	-0.15	-0.22	-0.08	9.19E-06	1195	-0.11	-0.17	-0.05	5.46E-04
Concentration of very small VLDL particles (mol/l)	1207	-0.05	-0.11	0.02	0.146	1195	-0.02	-0.09	0.04	0.442
Total lipids in very small VLDL (mmol/l)	1207	-0.06	-0.13	0.00	0.045	1195	-0.04	-0.10	0.02	0.223
Phospholipids in very small VLDL (mmol/l)	1207	-0.03	-0.09	0.03	0.291	1195	-0.02	-0.08	0.04	0.519
Total cholesterol in very small VLDL (mmol/l)	1207	-0.05	-0.11	0.01	0.118	1195	-0.03	-0.09	0.04	0.443
Cholesterol esters in very small VLDL (mmol/l)	1207	-0.07	-0.13	-0.01	0.033	1195	-0.04	-0.11	0.02	0.215
Free cholesterol in very small VLDL (mmol/l)	1207	0.00	-0.06	0.06	0.880	1195	0.01	-0.05	0.07	0.745
Triglycerides in very small VLDL (mmol/l)	1207	-0.10	-0.16	-0.04	1.39E-03	1195	-0.08	-0.14	-0.01	0.016
Concentration of IDL particles (mol/l)	1207	-0.02	-0.08	0.04	0.465	1195	-0.02	-0.08	0.04	0.520
Total lipids in IDL (mmol/l)	1207	-0.02	-0.08	0.04	0.471	1195	-0.02	-0.08	0.05	0.608
Phospholipids in IDL (mmol/l)	1207	-0.01	-0.07	0.05	0.713	1195	-0.01	-0.07	0.05	0.744
Total cholesterol in IDL (mmol/l)	1207	-0.03	-0.09	0.04	0.398	1195	-0.02	-0.08	0.05	0.582
Cholesterol esters in IDL (mmol/l)	1207	-0.04	-0.10	0.02	0.215	1195	-0.03	-0.09	0.04	0.422
Free cholesterol in IDL (mmol/l)	1207	0.00	-0.06	0.07	0.896	1195	0.00	-0.06	0.06	0.944
Triglycerides in IDL (mmol/l)	1207	-0.01	-0.07	0.05	0.637	1195	-0.02	-0.08	0.04	0.613
Concentration of large LDL particles (mol/l)	1207	-0.02	-0.08	0.04	0.487	1195	-0.02	-0.08	0.05	0.601
Total lipids in large LDL (mmol/l)	1207	-0.02	-0.08	0.04	0.522	1195	-0.01	-0.08	0.05	0.663
Phospholipids in large LDL (mmol/l)	1207	-0.03	-0.09	0.04	0.421	1195	-0.02	-0.08	0.05	0.624
Total cholesterol in large LDL (mmol/l)	1207	-0.02	-0.08	0.04	0.523	1195	-0.01	-0.08	0.05	0.676
Cholesterol esters in large LDL (mmol/l)	1207	-0.03	-0.09	0.03	0.400	1195	-0.02	-0.08	0.04	0.577
Free cholesterol in large LDL (mmol/l)	1207	0.00	-0.06	0.06	0.986	1195	0.00	-0.06	0.06	0.992
Triglycerides in large LDL (mmol/l)	1207	0.00	-0.06	0.06	0.940	1195	-0.01	-0.07	0.05	0.759
Concentration of medium LDL particles (mol/l)	1207	-0.04	-0.10	0.02	0.243	1195	-0.03	-0.09	0.03	0.379
Total lipids in medium LDL (mmol/l)	1207	-0.03	-0.09	0.03	0.336	1195	-0.02	-0.08	0.04	0.501
Phospholipids in medium LDL (mmol/l)	1207	-0.04	-0.10	0.02	0.175	1195	-0.03	-0.09	0.04	0.417
Total cholesterol in medium LDL (mmol/l)	1207	-0.03	-0.09	0.03	0.344	1195	-0.02	-0.08	0.04	0.500
Cholesterol esters in medium LDL (mmol/l)	1207	-0.03	-0.09	0.03	0.295	1195	-0.02	-0.09	0.04	0.442
Free cholesterol in medium LDL (mmol/l)	1207	-0.02	-0.08	0.05	0.610	1195	-0.01	-0.07	0.05	0.787
Triglycerides in medium LDL (mmol/l)	1207	0.00	-0.06	0.06	0.899	1195	0.00	-0.06	0.06	0.907
Concentration of small LDL particles (mol/l)	1207	-0.04	-0.10	0.02	0.224	1195	-0.03	-0.09	0.03	0.359
Total lipids in small LDL (mmol/l)	1207	-0.03	-0.09	0.03	0.289	1195	-0.02	-0.09	0.04	0.460
Phospholipids in small LDL (mmol/l)	1207	-0.04	-0.10	0.02	0.237	1195	-0.02	-0.08	0.04	0.461
Total cholesterol in small LDL (mmol/l)	1207	-0.03	-0.09	0.03	0.372	1195	-0.02	-0.08	0.04	0.534
Cholesterol esters in small LDL (mmol/l)	1207	-0.03	-0.09	0.03	0.331	1195	-0.02	-0.09	0.04	0.468
Free cholesterol in small LDL (mmol/l)	1207	-0.01	-0.08	0.05	0.637	1195	0.00	-0.07	0.06	0.911
Triglycerides in small LDL (mmol/l)	1207	-0.05	-0.11	0.01	0.080	1195	-0.04	-0.10	0.02	0.150
Concentration of very large HDL particles (mol/l)	1207	0.12	0.05	0.19	3.86E-04	1195	0.08	0.02	0.14	0.015
Total lipids in very large HDL (mmol/l)	1207	0.11	0.05	0.18	7.11E-04	1195	0.08	0.01	0.14	0.024
Phospholipids in very large HDL (mmol/l)	1207	0.13	0.06	0.19	1.33E-04	1195	0.09	0.02	0.15	0.008
Total cholesterol in very large HDL (mmol/l)	1207	0.10	0.03	0.16	0.004	1195	0.06	0.00	0.13	0.063
Cholesterol esters in very large HDL (mmol/l)	1207	0.09	0.02	0.16	0.007	1195	0.06	-0.01	0.12	0.089
Free cholesterol in very large HDL (mmol/l)	1207	0.11	0.05	0.18	9.50E-04	1195	0.07	0.01	0.14	0.028
Triglycerides in very large HDL (mmol/l)	1207	-0.01	-0.07	0.05	0.700	1195	-0.03	-0.09	0.03	0.385
Concentration of large HDL particles (mol/l)	1207	0.14	0.08	0.21	1.91E-05	1195	0.10	0.04	0.17	1.26E-03
Total lipids in large HDL (mmol/l)	1207	0.14	0.08	0.21	1.91E-05	1195	0.10	0.04	0.17	1.39E-03
Phospholipids in large HDL (mmol/l)	1207	0.13	0.07	0.20	5.53E-05	1195	0.10	0.03	0.16	2.49E-03
Total cholesterol in large HDL (mmol/l)	1207	0.15	0.09	0.22	7.11E-06	1195	0.11	0.05	0.17	7.99E-04
Cholesterol esters in large HDL (mmol/l)	1207	0.15	0.09	0.22	6.56E-06	1195	0.11	0.05	0.17	7.54E-04
Free cholesterol in large HDL (mmol/l)	1207	0.15	0.08	0.21	1.06E-05	1195	0.11	0.04	0.17	1.07E-03
Triglycerides in large HDL (mmol/l)	1207	0.03	-0.03	0.09	0.318	1195	0.02	-0.04	0.08	0.516
Concentration of medium HDL particles (mol/l)	1207	0.06	0.00	0.12	0.041	1195	0.07	0.01	0.13	0.029
Total lipids in medium HDL (mmol/l)	1207	0.07	0.01	0.13	0.023	1195	0.07	0.01	0.13	0.025
Phospholipids in medium HDL (mmol/l)	1207	0.07	0.01	0.13	0.015	1195	0.07	0.01	0.13	0.016
Total cholesterol in medium HDL (mmol/l)	1207	0.08	0.02	0.14	0.010	1195	0.07	0.01	0.14	0.020
Cholesterol esters in medium HDL (mmol/l)	1207	0.08	0.02	0.14	0.008	1195	0.08	0.01	0.14	0.018

S2 Table Associations of current total physical activity (CPM at age 15y) with metabolic traits at age 15y in ALSPAC

	CPM at age 15y (per SD (164) higher)									
	Adj. for age, sex, ethnicity, maternal education, smoking, alcohol, wear time, wear month					*Additionally adj. for FMI at age 15y*				
**Standardised outcome at age 15y**	**N**	**Beta**	**LCL**	**UCL**	**P-value**	**N**	**Beta**	**LCL**	**UCL**	**P-value**
Free cholesterol in medium HDL (mmol/l)	1207	0.07	0.01	0.13	0.031	1195	0.06	0.00	0.12	0.041
Triglycerides in medium HDL (mmol/l)	1207	-0.10	-0.16	-0.04	1.35E-03	1195	-0.06	-0.12	0.00	0.049
Concentration of small HDL particles (mol/l)	1207	-0.04	-0.10	0.02	0.196	1195	-0.01	-0.07	0.05	0.784
Total lipids in small HDL (mmol/l)	1207	0.01	-0.05	0.07	0.691	1195	0.03	-0.02	0.09	0.258
Phospholipids in small HDL (mmol/l)	1207	-0.05	-0.12	0.01	0.079	1195	-0.03	-0.09	0.03	0.352
Total cholesterol in small HDL (mmol/l)	1207	0.10	0.04	0.15	9.77E-04	1195	0.10	0.05	0.16	4.24E-04
Cholesterol esters in small HDL (mmol/l)	1207	0.10	0.04	0.16	4.76E-04	1195	0.11	0.05	0.16	2.22E-04
Free cholesterol in small HDL (mmol/l)	1207	0.03	-0.03	0.09	0.303	1195	0.04	-0.02	0.10	0.210
Triglycerides in small HDL (mmol/l)	1207	-0.11	-0.18	-0.05	3.20E-04	1195	-0.08	-0.14	-0.02	0.008
Phospholipids to total lipds ratio in chylomicrons and extremely large VLDL (%)	1207	-0.07	-0.13	-0.01	0.032	1195	-0.07	-0.13	0.00	0.040
Total cholesterol to total lipids ratio in chylomicrons and extremely large VLDL (%)	1207	-0.02	-0.09	0.04	0.502	1195	0.01	-0.06	0.07	0.867
Cholesterol esters to total lipids ratio in chylomicrons and extremely large VLDL (%)	1207	0.01	-0.05	0.08	0.744	1195	0.04	-0.03	0.10	0.253
Free cholesterol to total lipids ratio in chylomicrons and extremely large VLDL (%)	1207	-0.11	-0.17	-0.04	1.43E-03	1195	-0.09	-0.16	-0.02	0.010
Triglycerides to total lipids ratio in chylomicrons and extremely large VLDL (%)	1207	0.02	-0.02	0.07	0.363	1195	0.00	-0.04	0.05	0.940
Phospholipids to total lipds ratio in very large VLDL (%)	1207	-0.10	-0.17	-0.04	2.19E-03	1195	-0.08	-0.15	-0.02	0.015
Total cholesterol to total lipids ratio in very large VLDL (%)	1207	0.14	0.05	0.23	2.91E-03	1195	0.13	0.04	0.23	0.007
Cholesterol esters to total lipids ratio in very large VLDL (%)	1207	0.13	0.06	0.20	3.00E-04	1195	0.12	0.05	0.19	6.62E-04
Free cholesterol to total lipids ratio in very large VLDL (%)	1207	0.09	0.02	0.16	0.011	1195	0.08	0.01	0.15	0.023
Triglycerides to total lipids ratio in very large VLDL (%)	1207	-0.09	-0.16	-0.02	0.014	1195	-0.09	-0.16	-0.02	0.016
Phospholipids to total lipds ratio in large VLDL (%)	1207	-0.11	-0.18	-0.04	1.75E-03	1195	-0.09	-0.16	-0.02	0.009
Total cholesterol to total lipids ratio in large VLDL (%)	1207	-0.05	-0.11	0.02	0.175	1195	-0.03	-0.09	0.04	0.438
Cholesterol esters to total lipids ratio in large VLDL (%)	1207	0.07	-0.05	0.19	0.281	1195	0.08	-0.06	0.21	0.266
Free cholesterol to total lipids ratio in large VLDL (%)	1207	-0.12	-0.18	-0.05	4.32E-04	1195	-0.09	-0.16	-0.03	0.006
Triglycerides to total lipids ratio in large VLDL (%)	1207	0.08	-0.06	0.21	0.246	1195	0.09	-0.06	0.23	0.266
Phospholipids to total lipds ratio in medium VLDL (%)	1207	0.10	0.03	0.17	0.008	1195	0.06	-0.01	0.13	0.074
Total cholesterol to total lipids ratio in medium VLDL (%)	1207	0.01	-0.05	0.07	0.713	1195	0.02	-0.04	0.08	0.545
Cholesterol esters to total lipids ratio in medium VLDL (%)	1207	0.03	-0.03	0.09	0.314	1195	0.04	-0.02	0.10	0.227
Free cholesterol to total lipids ratio in medium VLDL (%)	1207	-0.06	-0.12	0.00	0.060	1195	-0.05	-0.12	0.01	0.093
Triglycerides to total lipids ratio in medium VLDL (%)	1207	-0.03	-0.09	0.03	0.325	1195	-0.03	-0.10	0.03	0.326
Phospholipids to total lipds ratio in small VLDL (%)	1207	0.17	0.10	0.23	5.24E-07	1195	0.15	0.08	0.21	6.68E-06
Total cholesterol to total lipids ratio in small VLDL (%)	1207	0.04	-0.03	0.10	0.273	1195	0.03	-0.03	0.10	0.337
Cholesterol esters to total lipids ratio in small VLDL (%)	1207	0.01	-0.05	0.08	0.678	1195	0.01	-0.05	0.08	0.704
Free cholesterol to total lipids ratio in small VLDL (%)	1207	0.16	0.09	0.22	1.16E-06	1195	0.14	0.07	0.20	1.83E-05
Triglycerides to total lipids ratio in small VLDL (%)	1207	-0.08	-0.15	-0.02	0.011	1195	-0.07	-0.14	-0.01	0.025
Phospholipids to total lipds ratio in very small VLDL (%)	1207	0.03	-0.03	0.09	0.311	1195	0.02	-0.04	0.08	0.601
Total cholesterol to total lipids ratio in very small VLDL (%)	1207	0.04	-0.02	0.10	0.234	1195	0.04	-0.02	0.10	0.204
Cholesterol esters to total lipids ratio in very small VLDL (%)	1207	-0.01	-0.07	0.05	0.660	1195	0.00	-0.06	0.06	0.894
Free cholesterol to total lipids ratio in very small VLDL (%)	1207	0.12	0.07	0.18	2.28E-05	1195	0.11	0.05	0.17	2.23E-04
Triglycerides to total lipids ratio in very small VLDL (%)	1207	-0.06	-0.13	0.00	0.039	1195	-0.06	-0.12	0.00	0.067
Phospholipids to total lipds ratio in IDL (%)	1207	0.08	0.02	0.14	0.010	1195	0.05	-0.01	0.11	0.111
Total cholesterol to total lipids ratio in IDL (%)	1207	-0.04	-0.10	0.02	0.188	1195	-0.02	-0.08	0.04	0.437
Cholesterol esters to total lipids ratio in IDL (%)	1207	-0.09	-0.15	-0.03	0.004	1195	-0.06	-0.12	0.00	0.061
Free cholesterol to total lipids ratio in IDL (%)	1207	0.11	0.05	0.17	6.29E-04	1195	0.08	0.02	0.14	0.012
Triglycerides to total lipids ratio in IDL (%)	1207	0.01	-0.05	0.07	0.694	1195	0.01	-0.06	0.07	0.839
Phospholipids to total lipds ratio in large LDL (%)	1207	0.00	-0.06	0.05	0.947	1195	0.01	-0.05	0.06	0.857
Total cholesterol to total lipids ratio in large LDL (%)	1207	-0.01	-0.07	0.04	0.626	1195	-0.01	-0.07	0.05	0.705
Cholesterol esters to total lipids ratio in large LDL (%)	1207	-0.04	-0.10	0.02	0.164	1195	-0.03	-0.09	0.03	0.302
Free cholesterol to total lipids ratio in large LDL (%)	1207	0.09	0.03	0.14	0.003	1195	0.06	0.01	0.12	0.028
Triglycerides to total lipids ratio in large LDL (%)	1207	0.03	-0.03	0.09	0.358	1195	0.01	-0.05	0.08	0.640
Phospholipids to total lipds ratio in medium LDL (%)	1207	0.00	-0.02	0.02	0.987	1195	0.00	-0.02	0.02	0.838
Total cholesterol to total lipids ratio in medium LDL (%)	1207	-0.02	-0.08	0.04	0.494	1195	-0.02	-0.08	0.04	0.517
Cholesterol esters to total lipids ratio in medium LDL (%)	1207	-0.03	-0.09	0.03	0.328	1195	-0.03	-0.09	0.04	0.402
Free cholesterol to total lipids ratio in medium LDL (%)	1207	0.01	-0.01	0.03	0.213	1195	0.01	-0.01	0.03	0.330
Triglycerides to total lipids ratio in medium LDL (%)	1207	0.05	-0.01	0.11	0.093	1195	0.03	-0.03	0.09	0.265
Phospholipids to total lipds ratio in small LDL (%)	1207	0.01	-0.02	0.05	0.545	1195	0.01	-0.03	0.05	0.578
Total cholesterol to total lipids ratio in small LDL (%)	1207	-0.01	-0.07	0.06	0.866	1195	-0.01	-0.07	0.06	0.849
Cholesterol esters to total lipids ratio in small LDL (%)	1207	-0.02	-0.08	0.04	0.529	1195	-0.02	-0.08	0.04	0.571
Free cholesterol to total lipids ratio in small LDL (%)	1207	0.02	-0.01	0.05	0.181	1195	0.02	-0.01	0.05	0.260
Triglycerides to total lipids ratio in small LDL (%)	1207	-0.03	-0.09	0.03	0.266	1195	-0.03	-0.09	0.03	0.355
Phospholipids to total lipds ratio in very large HDL (%)	1207	0.13	0.07	0.20	7.49E-05	1195	0.09	0.03	0.15	0.004
Total cholesterol to total lipids ratio in very large HDL (%)	1207	-0.11	-0.18	-0.05	3.85E-04	1195	-0.08	-0.13	-0.02	0.012
Cholesterol esters to total lipids ratio in very large HDL (%)	1207	-0.11	-0.18	-0.05	4.62E-04	1195	-0.07	-0.13	-0.01	0.015
Free cholesterol to total lipids ratio in very large HDL (%)	1207	0.03	-0.03	0.09	0.268	1195	0.02	-0.04	0.08	0.602
Triglycerides to total lipids ratio in very large HDL (%)	1207	-0.12	-0.19	-0.06	2.91E-04	1195	-0.10	-0.16	-0.03	0.003
Phospholipids to total lipds ratio in large HDL (%)	1207	-0.12	-0.18	-0.06	8.65E-05	1195	-0.08	-0.13	-0.02	0.011
Total cholesterol to total lipids ratio in large HDL (%)	1207	0.14	0.08	0.21	1.68E-05	1195	0.09	0.03	0.15	0.003
Cholesterol esters to total lipids ratio in large HDL (%)	1207	0.14	0.08	0.21	2.44E-05	1195	0.09	0.03	0.16	0.004
Free cholesterol to total lipids ratio in large HDL (%)	1207	0.11	0.05	0.17	3.00E-04	1195	0.07	0.01	0.13	0.015
Triglycerides to total lipids ratio in large HDL (%)	1207	-0.14	-0.21	-0.08	3.55E-05	1195	-0.10	-0.17	-0.04	2.29E-03
Phospholipids to total lipds ratio in medium HDL (%)	1207	0.07	0.01	0.14	0.018	1195	0.07	0.01	0.13	0.025
Total cholesterol to total lipids ratio in medium HDL (%)	1207	0.02	-0.04	0.08	0.553	1195	0.00	-0.07	0.06	0.930
Cholesterol esters to total lipids ratio in medium HDL (%)	1207	0.02	-0.04	0.08	0.465	1195	0.00	-0.06	0.06	0.966
Free cholesterol to total lipids ratio in medium HDL (%)	1207	-0.01	-0.07	0.05	0.769	1195	-0.02	-0.08	0.05	0.608
Triglycerides to total lipids ratio in medium HDL (%)	1207	-0.13	-0.20	-0.07	5.61E-05	1195	-0.09	-0.16	-0.03	0.004
Phospholipids to total lipds ratio in small HDL (%)	1207	-0.12	-0.18	-0.07	3.40E-05	1195	-0.12	-0.18	-0.06	8.29E-05
Total cholesterol to total lipids ratio in small HDL (%)	1207	0.15	0.09	0.20	9.92E-07	1195	0.13	0.08	0.19	5.65E-06
Cholesterol esters to total lipids ratio in small HDL (%)	1207	0.13	0.07	0.19	1.29E-05	1195	0.12	0.07	0.18	3.24E-05
Free cholesterol to total lipids ratio in small HDL (%)	1207	0.05	-0.01	0.12	0.093	1195	0.02	-0.04	0.08	0.539
Triglycerides to total lipids ratio in small HDL (%)	1207	-0.14	-0.20	-0.07	2.74E-05	1195	-0.11	-0.17	-0.05	5.24E-04
Mean diameter for VLDL particles (nm)	1207	-0.16	-0.23	-0.10	1.51E-06	1195	-0.12	-0.19	-0.06	1.04E-04
Mean diameter for LDL particles (nm)	1207	0.05	0.00	0.11	0.066	1195	0.03	-0.02	0.09	0.261
Mean diameter for HDL particles (nm)	1207	0.14	0.07	0.21	3.96E-05	1195	0.10	0.03	0.16	0.004
Serum total cholesterol (mmol/l)	1207	0.00	-0.06	0.06	0.930	1195	0.00	-0.06	0.06	0.932
Total cholesterol in VLDL (mmol/l)	1207	-0.13	-0.20	-0.07	5.13E-05	1195	-0.09	-0.15	-0.03	0.004
Remnant cholesterol (non-HDL, non-LDL -cholesterol) (mmol/l)	1207	-0.09	-0.16	-0.03	0.004	1195	-0.06	-0.13	0.00	0.048
Total cholesterol in LDL (mmol/l)	1207	-0.02	-0.09	0.04	0.435	1195	-0.02	-0.08	0.05	0.593
Total cholesterol in HDL (mmol/l)	1207	0.13	0.07	0.20	3.80E-05	1195	0.10	0.04	0.17	1.08E-03
Total cholesterol in HDL2 (mmol/l)	1207	0.15	0.08	0.21	1.06E-05	1195	0.11	0.05	0.18	4.81E-04
Total cholesterol in HDL3 (mmol/l)	1207	0.11	0.05	0.17	7.24E-04	1195	0.08	0.02	0.15	0.007
Esterified cholesterol (mmol/l)	1207	0.00	-0.06	0.06	0.997	1195	0.01	-0.06	0.07	0.865
Free cholesterol (mmol/l)	1207	-0.01	-0.07	0.05	0.791	1195	0.00	-0.07	0.06	0.918

S2 Table Associations of current total physical activity (CPM at age 15y) with metabolic traits at age 15y in ALSPAC

	**CPM at age 15y (per SD (164) higher)**									
	Adj. for age, sex, ethnicity, maternal education, smoking, alcohol, wear time, wear month					*Additionally adj. for FMI at age 15y*				
**Standardised outcome at age 15y**	**N**	**Beta**	**LCL**	**UCL**	**P-value**	**N**	**Beta**	**LCL**	**UCL**	**P-value**
Serum total triglycerides (mmol/l)	1207	-0.15	-0.21	-0.08	7.24E-06	1195	-0.11	-0.17	-0.05	3.58E-04
Triglycerides in VLDL (mmol/l)	1207	-0.16	-0.23	-0.10	1.32E-06	1195	-0.12	-0.18	-0.06	1.06E-04
Triglycerides in LDL (mmol/l)	1207	-0.01	-0.07	0.05	0.749	1195	-0.01	-0.07	0.05	0.647
Triglycerides in HDL (mmol/l)	1207	-0.08	-0.14	-0.02	0.007	1195	-0.06	-0.12	0.00	0.049
Diacylglycerol (mmol/l)	1166	-0.05	-0.11	0.01	0.134	1155	-0.02	-0.08	0.04	0.581
Ratio of diacylglycerol to triglycerides	1166	0.02	-0.04	0.08	0.483	1155	0.03	-0.03	0.09	0.312
Total phosphoglycerides (mmol/l)	1207	0.03	-0.02	0.09	0.262	1195	0.03	-0.03	0.09	0.363
Ratio of triglycerides to phosphoglycerides	1207	-0.14	-0.20	-0.07	9.53E-05	1195	-0.09	-0.16	-0.03	0.004
Phosphatidylcholine and other cholines (mmol/l)	1185	0.04	-0.02	0.10	0.151	1173	0.04	-0.02	0.10	0.231
Total cholines (mmol/l)	1203	0.05	-0.01	0.11	0.120	1191	0.04	-0.02	0.10	0.203
Apolipoprotein A-I (g/l)	1207	0.09	0.03	0.15	0.005	1195	0.07	0.01	0.13	0.023
Apolipoprotein B (g/l)	1207	-0.12	-0.18	-0.05	3.56E-04	1195	-0.08	-0.15	-0.02	0.008
Ratio of apolipoprotein B to apolipoprotein A-I	1207	-0.16	-0.23	-0.09	4.03E-06	1195	-0.12	-0.18	-0.05	4.08E-04
Total fatty acids (mmol/l)	1207	-0.05	-0.11	0.01	0.077	1195	-0.04	-0.09	0.02	0.241
Estimated description of fatty acid chain length, not actual carbon number	1201	-0.02	-0.08	0.05	0.606	1189	-0.01	-0.08	0.05	0.748
Estimated degree of unsaturation	1206	0.05	-0.02	0.11	0.173	1194	0.04	-0.02	0.11	0.201
22:6, docosahexaenoic acid (mmol/l)	1207	0.00	-0.06	0.06	0.981	1195	0.01	-0.05	0.07	0.790
18:2, linoleic acid (mmol/l)	1203	0.01	-0.05	0.06	0.864	1191	0.01	-0.05	0.06	0.845
Conjugated linoleic acid (mmol/l)	1207	-0.03	-0.10	0.04	0.361	1195	-0.03	-0.10	0.04	0.419
Omega-3 fatty acids (mmol/l)	1205	-0.02	-0.08	0.04	0.429	1193	-0.02	-0.08	0.04	0.591
Omega-6 fatty acids (mmol/l)	1204	-0.01	-0.07	0.05	0.779	1192	0.00	-0.06	0.06	0.927
Polyunsaturated fatty acids (mmol/l)	1202	-0.01	-0.07	0.05	0.773	1190	0.00	-0.06	0.06	0.925
Monounsaturated fatty acids; 16:1, 18:1 (mmol/l)	1204	-0.10	-0.16	-0.04	2.17E-03	1192	-0.07	-0.13	-0.01	0.028
Saturated fatty acids (mmol/l)	1202	-0.04	-0.10	0.02	0.206	1190	-0.03	-0.09	0.04	0.405
Ratio of 22:6 docosahexaenoic acid to total fatty acids (%)	1207	0.03	-0.04	0.09	0.383	1195	0.03	-0.04	0.09	0.414
Ratio of 18:2 linoleic acid to total fatty acids (%)	1203	0.09	0.02	0.15	0.009	1191	0.06	0.00	0.13	0.064
Ratio of conjugated linoleic acid to total fatty acids (%)	1207	-0.02	-0.10	0.05	0.550	1195	-0.02	-0.10	0.05	0.569
Ratio of omega-3 fatty acids to total fatty acids (%)	1205	0.01	-0.05	0.07	0.761	1193	0.00	-0.06	0.07	0.902
Ratio of omega-6 fatty acids to total fatty acids (%)	1204	0.09	0.03	0.16	0.006	1192	0.07	0.00	0.13	0.042
Ratio of polyunsaturated fatty acids to total fatty acids (%)	1202	0.09	0.02	0.15	0.008	1190	0.06	0.00	0.13	0.053
Ratio of monounsaturated fatty acids to total fatty acids (%)	1204	-0.10	-0.17	-0.03	2.98E-03	1192	-0.07	-0.13	-0.01	0.032
Ratio of saturated fatty acids to total fatty acids (%)	1202	0.02	-0.04	0.08	0.550	1190	0.01	-0.05	0.08	0.719
Insulin (mu/l)	1253	-0.09	-0.12	-0.05	9.15E-06	1241	-0.04	-0.08	-0.01	0.020
Glucose (mmol/l)	1204	-0.05	-0.11	0.00	0.070	1192	-0.04	-0.10	0.02	0.191
Lactate (mmol/l)	1205	-0.04	-0.11	0.02	0.186	1193	-0.04	-0.10	0.03	0.254
Pyruvate (mmol/l)	1203	-0.09	-0.16	-0.02	0.008	1191	-0.07	-0.13	0.00	0.057
Citrate (mmol/l)	1198	0.12	0.05	0.19	7.86E-04	1186	0.10	0.03	0.17	0.006
Alanine (mmol/l)	1207	-0.07	-0.13	0.00	0.045	1195	-0.06	-0.13	0.01	0.075
Glutamine (mmol/l)	1207	0.04	-0.02	0.10	0.216	1195	0.02	-0.04	0.08	0.484
Histidine (mmol/l)	1141	0.02	-0.05	0.08	0.589	1129	0.02	-0.05	0.09	0.547
Isoleucine (mmol/l)	1207	-0.08	-0.15	-0.02	0.013	1195	-0.05	-0.12	0.01	0.094
Leucine (mmol/l)	1207	0.01	-0.05	0.07	0.771	1195	0.03	-0.03	0.09	0.393
Valine (mmol/l)	1207	-0.05	-0.11	0.01	0.116	1195	-0.02	-0.08	0.04	0.505
Phenylalanine (mmol/l)	1206	0.05	-0.02	0.12	0.127	1194	0.08	0.01	0.15	0.031
Tyrosine (mmol/l)	1200	0.04	-0.03	0.11	0.279	1188	0.07	0.00	0.15	0.041
Acetate (mmol/l)	1206	0.06	0.00	0.12	0.046	1194	0.05	-0.01	0.11	0.100
Acetoacetate (mmol/l)	1207	0.03	-0.05	0.11	0.495	1195	0.03	-0.05	0.11	0.470
3-hydroxybutyrate (mmol/l)	1206	-0.02	-0.07	0.04	0.602	1194	-0.01	-0.07	0.05	0.650
Creatinine (mmol/l)	1206	-0.11	-0.17	-0.04	8.74E-04	1194	-0.10	-0.16	-0.04	2.04E-03
Albumin (signal area)	1207	-0.12	-0.19	-0.06	1.64E-04	1195	-0.13	-0.20	-0.06	1.09E-04
Glycoprotein acetyls, mainly a1-acid glycoprotein (mmol/l)	1206	-0.16	-0.22	-0.10	6.34E-08	1194	-0.11	-0.16	-0.06	7.88E-05
C-reactive protein (mg/l)	1255	-0.03	-0.07	0.02	0.202	1243	-0.02	-0.06	0.02	0.370

Complete case sample

	**CPM at age 15y (per SD (164) higher)**									
	Adj. for age, sex, ethnicity, maternal education, smoking, alcohol, wear time, wear month					*Additionally adj. for FMI at age 15y*				
**Standardised outcome at age 15y**	**N**	**Beta**	**LCL**	**UCL**	**P-value**	**N**	**Beta**	**LCL**	**UCL**	**P-value**
Systolic blood pressure (mmHg)	755	-0.02	-0.10	0.06	0.650	755	0.00	-0.08	0.08	0.982
Diastolic blood pressure (mmHg)	755	0.01	-0.07	0.09	0.810	755	0.01	-0.07	0.09	0.736
Concentration of chylomicrons and extremely large VLDL particles (mol/l)	755	-0.12	-0.19	-0.05	1.35E-03	755	-0.09	-0.16	-0.03	0.007
Total lipids in chylomicrons and extremely large VLDL (mmol/l)	755	-0.12	-0.19	-0.05	1.32E-03	755	-0.09	-0.16	-0.03	0.007
Phospholipids in chylomicrons and extremely large VLDL (mmol/l)	755	-0.12	-0.19	-0.05	1.06E-03	755	-0.10	-0.17	-0.03	0.005
Total cholesterol in chylomicrons and extremely large VLDL (mmol/l)	755	-0.10	-0.17	-0.03	0.007	755	-0.07	-0.14	-0.01	0.033
Cholesterol esters in chylomicrons and extremely large VLDL (mmol/l)	755	-0.08	-0.15	0.00	0.039	755	-0.05	-0.12	0.02	0.145
Free cholesterol in chylomicrons and extremely large VLDL (mmol/l)	755	-0.12	-0.19	-0.05	1.45E-03	755	-0.09	-0.16	-0.03	0.007
Triglycerides in chylomicrons and extremely large VLDL (mmol/l)	755	-0.12	-0.20	-0.05	9.52E-04	755	-0.10	-0.17	-0.03	0.005
Concentration of very large VLDL particles (mol/l)	755	-0.12	-0.19	-0.04	1.80E-03	755	-0.09	-0.16	-0.02	0.009
Total lipids in very large VLDL (mmol/l)	755	-0.11	-0.19	-0.04	2.17E-03	755	-0.09	-0.16	-0.02	0.010
Phospholipids in very large VLDL (mmol/l)	755	-0.11	-0.18	-0.04	2.26E-03	755	-0.09	-0.16	-0.02	0.010
Total cholesterol in very large VLDL (mmol/l)	755	-0.11	-0.18	-0.04	0.003	755	-0.08	-0.15	-0.02	0.017
Cholesterol esters in very large VLDL (mmol/l)	755	-0.11	-0.18	-0.03	0.005	755	-0.08	-0.15	-0.01	0.026
Free cholesterol in very large VLDL (mmol/l)	755	-0.11	-0.19	-0.04	2.42E-03	755	-0.09	-0.16	-0.02	0.012
Triglycerides in very large VLDL (mmol/l)	755	-0.11	-0.19	-0.04	1.97E-03	755	-0.09	-0.16	-0.02	0.009
Concentration of large VLDL particles (mol/l)	755	-0.11	-0.18	-0.04	0.003	755	-0.09	-0.16	-0.02	0.015
Total lipids in large VLDL (mmol/l)	755	-0.11	-0.18	-0.03	0.004	755	-0.08	-0.15	-0.01	0.018
Phospholipids in large VLDL (mmol/l)	755	-0.11	-0.18	-0.03	0.004	755	-0.08	-0.15	-0.01	0.019
Total cholesterol in large VLDL (mmol/l)	755	-0.10	-0.17	-0.02	0.010	755	-0.07	-0.14	0.00	0.042
Cholesterol esters in large VLDL (mmol/l)	755	-0.09	-0.16	-0.01	0.025	755	-0.06	-0.13	0.01	0.096
Free cholesterol in large VLDL (mmol/l)	755	-0.11	-0.18	-0.03	0.004	755	-0.08	-0.15	-0.01	0.019
Triglycerides in large VLDL (mmol/l)	755	-0.11	-0.19	-0.04	2.83E-03	755	-0.09	-0.16	-0.02	0.013
Concentration of medium VLDL particles (mol/l)	755	-0.11	-0.19	-0.03	0.004	755	-0.08	-0.16	-0.01	0.021
Total lipids in medium VLDL (mmol/l)	755	-0.11	-0.18	-0.03	0.007	755	-0.08	-0.15	-0.01	0.033
Phospholipids in medium VLDL (mmol/l)	755	-0.10	-0.18	-0.03	0.008	755	-0.08	-0.15	-0.01	0.035
Total cholesterol in medium VLDL (mmol/l)	755	-0.08	-0.16	0.00	0.047	755	-0.05	-0.13	0.02	0.162
Cholesterol esters in medium VLDL (mmol/l)	755	-0.06	-0.13	0.02	0.167	755	-0.03	-0.11	0.05	0.440
Free cholesterol in medium VLDL (mmol/l)	755	-0.10	-0.18	-0.03	0.009	755	-0.08	-0.15	0.00	0.038
Triglycerides in medium VLDL (mmol/l)	755	-0.12	-0.19	-0.04	0.003	755	-0.09	-0.16	-0.02	0.015

S2 Table Associations of current total physical activity (CPM at age 15y) with metabolic traits at age 15y in ALSPAC

	CPM at age 15y (per SD (164) higher)									
	Adj. for age, sex, ethnicity, maternal education, smoking, alcohol, wear time, wear month					*Additionally adj. for FMI at age 15y*				
**Standardised outcome at age 15y**	**N**	**Beta**	**LCL**	**UCL**	**P-value**	**N**	**Beta**	**LCL**	**UCL**	**P-value**
Concentration of small VLDL particles (mol/l)	755	-0.09	-0.17	-0.01	0.031	755	-0.06	-0.14	0.01	0.113
Total lipids in small VLDL (mmol/l)	755	-0.08	-0.16	0.00	0.044	755	-0.06	-0.13	0.02	0.159
Phospholipids in small VLDL (mmol/l)	755	-0.06	-0.14	0.02	0.127	755	-0.04	-0.11	0.04	0.364
Total cholesterol in small VLDL (mmol/l)	755	-0.07	-0.15	0.02	0.113	755	-0.04	-0.12	0.04	0.335
Cholesterol esters in small VLDL (mmol/l)	755	-0.06	-0.15	0.02	0.125	755	-0.04	-0.12	0.04	0.358
Free cholesterol in small VLDL (mmol/l)	755	-0.06	-0.14	0.02	0.125	755	-0.04	-0.11	0.04	0.340
Triglycerides in small VLDL (mmol/l)	755	-0.09	-0.17	-0.02	0.019	755	-0.07	-0.15	0.00	0.066
Concentration of very small VLDL particles (mol/l)	755	-0.01	-0.09	0.07	0.798	755	0.00	-0.07	0.08	0.936
Total lipids in very small VLDL (mmol/l)	755	-0.03	-0.11	0.04	0.398	755	-0.02	-0.10	0.06	0.677
Phospholipids in very small VLDL (mmol/l)	755	-0.01	-0.08	0.07	0.891	755	0.00	-0.07	0.08	0.945
Total cholesterol in very small VLDL (mmol/l)	755	-0.04	-0.12	0.05	0.390	755	-0.02	-0.10	0.07	0.701
Cholesterol esters in very small VLDL (mmol/l)	755	-0.05	-0.14	0.03	0.192	755	-0.03	-0.11	0.05	0.445
Free cholesterol in very small VLDL (mmol/l)	755	0.01	-0.07	0.08	0.825	755	0.02	-0.06	0.10	0.635
Triglycerides in very small VLDL (mmol/l)	755	-0.05	-0.13	0.02	0.184	755	-0.04	-0.11	0.04	0.339
Concentration of IDL particles (mol/l)	755	0.00	-0.08	0.07	0.945	755	0.00	-0.08	0.08	0.986
Total lipids in IDL (mmol/l)	755	0.00	-0.08	0.07	0.971	755	0.00	-0.07	0.08	0.938
Phospholipids in IDL (mmol/l)	755	0.01	-0.07	0.08	0.849	755	0.01	-0.07	0.09	0.829
Total cholesterol in IDL (mmol/l)	755	-0.01	-0.08	0.07	0.869	755	0.00	-0.08	0.08	0.993
Cholesterol esters in IDL (mmol/l)	755	-0.02	-0.09	0.06	0.694	755	-0.01	-0.09	0.07	0.884
Free cholesterol in IDL (mmol/l)	755	0.02	-0.06	0.09	0.697	755	0.01	-0.06	0.09	0.705
Triglycerides in IDL (mmol/l)	755	0.01	-0.06	0.08	0.793	755	0.01	-0.07	0.08	0.872
Concentration of large LDL particles (mol/l)	755	0.00	-0.07	0.08	0.927	755	0.01	-0.07	0.08	0.864
Total lipids in large LDL (mmol/l)	755	0.01	-0.07	0.08	0.895	755	0.01	-0.07	0.09	0.817
Phospholipids in large LDL (mmol/l)	755	0.00	-0.07	0.08	0.933	755	0.01	-0.07	0.09	0.812
Total cholesterol in large LDL (mmol/l)	755	0.00	-0.07	0.08	0.916	755	0.01	-0.07	0.09	0.823
Cholesterol esters in large LDL (mmol/l)	755	0.00	-0.08	0.08	0.994	755	0.01	-0.07	0.08	0.875
Free cholesterol in large LDL (mmol/l)	755	0.02	-0.06	0.09	0.690	755	0.02	-0.06	0.09	0.670
Triglycerides in large LDL (mmol/l)	755	0.02	-0.06	0.09	0.674	755	0.01	-0.07	0.08	0.835
Concentration of medium LDL particles (mol/l)	755	-0.01	-0.08	0.07	0.873	755	0.00	-0.08	0.08	0.979
Total lipids in medium LDL (mmol/l)	755	0.00	-0.08	0.07	0.966	755	0.00	-0.07	0.08	0.925
Phospholipids in medium LDL (mmol/l)	755	0.00	-0.08	0.07	0.898	755	0.00	-0.07	0.08	0.900
Total cholesterol in medium LDL (mmol/l)	755	0.00	-0.08	0.07	0.917	755	0.00	-0.08	0.08	0.969
Cholesterol esters in medium LDL (mmol/l)	755	-0.01	-0.08	0.07	0.869	755	0.00	-0.08	0.08	0.985
Free cholesterol in medium LDL (mmol/l)	755	0.01	-0.07	0.08	0.884	755	0.01	-0.07	0.09	0.783
Triglycerides in medium LDL (mmol/l)	755	0.02	-0.05	0.10	0.510	755	0.02	-0.06	0.09	0.656
Concentration of small LDL particles (mol/l)	755	-0.01	-0.09	0.07	0.810	755	0.00	-0.08	0.07	0.914
Total lipids in small LDL (mmol/l)	755	0.00	-0.08	0.07	0.904	755	0.00	-0.08	0.08	0.975
Phospholipids in small LDL (mmol/l)	755	-0.01	-0.08	0.07	0.895	755	0.00	-0.07	0.08	0.952
Total cholesterol in small LDL (mmol/l)	755	0.00	-0.08	0.07	0.937	755	0.00	-0.08	0.08	0.948
Cholesterol esters in small LDL (mmol/l)	755	0.00	-0.08	0.07	0.907	755	0.00	-0.08	0.08	0.988
Free cholesterol in small LDL (mmol/l)	755	0.00	-0.07	0.08	0.918	755	0.01	-0.07	0.09	0.772
Triglycerides in small LDL (mmol/l)	755	-0.01	-0.09	0.06	0.695	755	-0.01	-0.09	0.06	0.747
Concentration of very large HDL particles (mol/l)	755	0.10	0.01	0.18	0.027	755	0.07	-0.01	0.15	0.101
Total lipids in very large HDL (mmol/l)	755	0.09	0.00	0.18	0.039	755	0.06	-0.02	0.15	0.134
Phospholipids in very large HDL (mmol/l)	755	0.11	0.02	0.19	0.015	755	0.08	-0.01	0.16	0.066
Total cholesterol in very large HDL (mmol/l)	755	0.07	-0.01	0.16	0.102	755	0.05	-0.04	0.13	0.259
Cholesterol esters in very large HDL (mmol/l)	755	0.06	-0.02	0.15	0.145	755	0.04	-0.04	0.13	0.329
Free cholesterol in very large HDL (mmol/l)	755	0.09	0.00	0.18	0.042	755	0.06	-0.02	0.15	0.140
Triglycerides in very large HDL (mmol/l)	755	0.00	-0.08	0.07	0.908	755	-0.02	-0.09	0.06	0.657
Concentration of large HDL particles (mol/l)	755	0.12	0.03	0.20	0.007	755	0.09	0.01	0.17	0.033
Total lipids in large HDL (mmol/l)	755	0.12	0.03	0.20	0.007	755	0.09	0.01	0.17	0.032
Phospholipids in large HDL (mmol/l)	755	0.11	0.03	0.20	0.008	755	0.09	0.01	0.17	0.036
Total cholesterol in large HDL (mmol/l)	755	0.12	0.04	0.21	0.006	755	0.09	0.01	0.17	0.029
Cholesterol esters in large HDL (mmol/l)	755	0.12	0.04	0.21	0.005	755	0.09	0.01	0.17	0.028
Free cholesterol in large HDL (mmol/l)	755	0.12	0.03	0.20	0.007	755	0.09	0.01	0.17	0.034
Triglycerides in large HDL (mmol/l)	755	0.04	-0.04	0.11	0.320	755	0.03	-0.05	0.10	0.486
Concentration of medium HDL particles (mol/l)	755	0.09	0.01	0.16	0.028	755	0.08	0.00	0.16	0.040
Total lipids in medium HDL (mmol/l)	755	0.09	0.01	0.16	0.027	755	0.08	0.00	0.16	0.044
Phospholipids in medium HDL (mmol/l)	755	0.09	0.02	0.17	0.017	755	0.09	0.01	0.16	0.029
Total cholesterol in medium HDL (mmol/l)	755	0.08	0.00	0.16	0.039	755	0.07	-0.01	0.15	0.073
Cholesterol esters in medium HDL (mmol/l)	755	0.08	0.00	0.16	0.039	755	0.07	-0.01	0.15	0.073
Free cholesterol in medium HDL (mmol/l)	755	0.08	0.00	0.15	0.048	755	0.07	-0.01	0.15	0.078
Triglycerides in medium HDL (mmol/l)	755	-0.03	-0.11	0.04	0.382	755	-0.01	-0.09	0.06	0.736
Concentration of small HDL particles (mol/l)	755	0.00	-0.07	0.08	0.928	755	0.02	-0.06	0.09	0.669
Total lipids in small HDL (mmol/l)	755	0.05	-0.02	0.13	0.163	755	0.06	-0.02	0.13	0.121
Phospholipids in small HDL (mmol/l)	755	-0.02	-0.10	0.06	0.614	755	-0.01	-0.09	0.07	0.804
Total cholesterol in small HDL (mmol/l)	755	0.12	0.05	0.19	6.80E-04	755	0.12	0.05	0.19	7.89E-04
Cholesterol esters in small HDL (mmol/l)	755	0.13	0.06	0.19	3.24E-04	755	0.12	0.06	0.19	3.58E-04
Free cholesterol in small HDL (mmol/l)	755	0.05	-0.02	0.13	0.174	755	0.05	-0.03	0.13	0.208
Triglycerides in small HDL (mmol/l)	755	-0.06	-0.14	0.01	0.092	755	-0.05	-0.12	0.03	0.201
Phospholipids to total lipids ratio in chylomicrons and extremely large VLDL (%)	755	-0.07	-0.15	0.01	0.096	755	-0.06	-0.15	0.02	0.114
Total cholesterol to total lipids ratio in chylomicrons and extremely large VLDL (%)	755	0.05	-0.04	0.13	0.271	755	0.06	-0.02	0.15	0.139
Cholesterol esters to total lipids ratio in chylomicrons and extremely large VLDL (%)	755	0.08	-0.01	0.16	0.072	755	0.09	0.01	0.17	0.031
Free cholesterol to total lipids ratio in chylomicrons and extremely large VLDL (%)	755	-0.06	-0.14	0.03	0.184	755	-0.04	-0.13	0.04	0.302
Triglycerides to total lipids ratio in chylomicrons and extremely large VLDL (%)	755	-0.03	-0.09	0.03	0.347	755	-0.04	-0.10	0.02	0.185
Phospholipids to total lipds ratio in very large VLDL (%)	755	-0.04	-0.13	0.04	0.288	755	-0.03	-0.11	0.05	0.473
Total cholesterol to total lipids ratio in very large VLDL (%)	755	0.15	0.03	0.27	0.018	755	0.15	0.02	0.28	0.027
Cholesterol esters to total lipids ratio in very large VLDL (%)	755	0.12	0.03	0.21	0.008	755	0.11	0.02	0.20	0.013
Free cholesterol to total lipids ratio in very large VLDL (%)	755	0.10	0.01	0.19	0.026	755	0.09	0.01	0.18	0.036
Triglycerides to total lipids ratio in very large VLDL (%)	755	-0.11	-0.20	-0.02	0.015	755	-0.11	-0.20	-0.02	0.018
Phospholipids to total lipds ratio in large VLDL (%)	755	-0.06	-0.15	0.03	0.166	755	-0.05	-0.14	0.04	0.277
Total cholesterol to total lipids ratio in large VLDL (%)	755	0.01	-0.07	0.10	0.779	755	0.03	-0.06	0.11	0.553
Cholesterol esters to total lipids ratio in large VLDL (%)	755	0.11	-0.06	0.29	0.206	755	0.12	-0.07	0.31	0.202
Free cholesterol to total lipids ratio in large VLDL (%)	755	-0.07	-0.15	0.01	0.089	755	-0.05	-0.14	0.03	0.198
Triglycerides to total lipids ratio in large VLDL (%)	755	0.10	-0.09	0.30	0.303	755	0.11	-0.10	0.32	0.309
Phospholipids to total lipds ratio in medium VLDL (%)	755	0.08	-0.01	0.18	0.077	755	0.06	-0.03	0.15	0.175
Total cholesterol to total lipids ratio in medium VLDL (%)	755	0.05	-0.03	0.13	0.197	755	0.06	-0.02	0.14	0.167
Cholesterol esters to total lipids ratio in medium VLDL (%)	755	0.07	-0.01	0.15	0.091	755	0.07	-0.01	0.15	0.078
Free cholesterol to total lipids ratio in medium VLDL (%)	755	-0.02	-0.10	0.05	0.534	755	-0.02	-0.10	0.06	0.608
Triglycerides to total lipids ratio in medium VLDL (%)	755	-0.07	-0.14	0.01	0.103	755	-0.07	-0.14	0.01	0.108
Phospholipids to total lipds ratio in small VLDL (%)	755	0.14	0.06	0.21	8.30E-04	755	0.12	0.04	0.20	2.72E-03

S2 Table Associations of current total physical activity (CPM at age 15y) with metabolic traits at age 15y in ALSPAC

	**CPM at age 15y (per SD (164) higher)**									
	Adj. for age, sex, ethnicity, maternal education, smoking, alcohol, wear time, wear month					*Additionally adj. for FMI at age 15y*				
**Standardised outcome at age 15y**	**N**	**Beta**	**LCL**	**UCL**	**P-value**	**N**	**Beta**	**LCL**	**UCL**	**P-value**
Total cholesterol to total lipids ratio in small VLDL (%)	755	0.03	-0.05	0.10	0.527	755	0.03	-0.05	0.11	0.521
Cholesterol esters to total lipids ratio in small VLDL (%)	755	0.01	-0.07	0.08	0.899	755	0.01	-0.07	0.09	0.830
Free cholesterol to total lipids ratio in small VLDL (%)	755	0.14	0.06	0.22	6.12E-04	755	0.12	0.05	0.20	1.94E-03
Triglycerides to total lipids ratio in small VLDL (%)	755	-0.06	-0.14	0.01	0.111	755	-0.06	-0.14	0.02	0.138
Phospholipids to total lipds ratio in very small VLDL (%)	755	0.04	-0.03	0.12	0.261	755	0.03	-0.04	0.11	0.395
Total cholesterol to total lipids ratio in very small VLDL (%)	755	-0.01	-0.08	0.07	0.849	755	0.00	-0.08	0.08	0.980
Cholesterol esters to total lipids ratio in very small VLDL (%)	755	-0.05	-0.12	0.02	0.169	755	-0.04	-0.11	0.03	0.281
Free cholesterol to total lipids ratio in very small VLDL (%)	755	0.09	0.02	0.16	0.018	755	0.08	0.01	0.16	0.036
Triglycerides to total lipids ratio in very small VLDL (%)	755	-0.02	-0.10	0.05	0.564	755	-0.02	-0.10	0.05	0.576
Phospholipids to total lipds ratio in IDL (%)	755	0.06	-0.01	0.14	0.105	755	0.04	-0.03	0.12	0.265
Total cholesterol to total lipids ratio in IDL (%)	755	-0.05	-0.13	0.03	0.205	755	-0.04	-0.11	0.04	0.381
Cholesterol esters to total lipids ratio in IDL (%)	755	-0.08	-0.16	0.00	0.058	755	-0.06	-0.13	0.02	0.176
Free cholesterol to total lipids ratio in IDL (%)	755	0.06	-0.01	0.13	0.106	755	0.04	-0.03	0.11	0.236
Triglycerides to total lipids ratio in IDL (%)	755	0.03	-0.05	0.11	0.415	755	0.02	-0.06	0.10	0.567
Phospholipids to total lipds ratio in large LDL (%)	755	0.00	-0.07	0.07	0.988	755	0.00	-0.07	0.07	0.954
Total cholesterol to total lipids ratio in large LDL (%)	755	-0.02	-0.09	0.05	0.598	755	-0.01	-0.09	0.06	0.737
Cholesterol esters to total lipids ratio in large LDL (%)	755	-0.03	-0.10	0.05	0.464	755	-0.02	-0.09	0.06	0.642
Free cholesterol to total lipids ratio in large LDL (%)	755	0.03	-0.04	0.10	0.348	755	0.02	-0.05	0.09	0.551
Triglycerides to total lipids ratio in large LDL (%)	755	0.04	-0.04	0.11	0.363	755	0.02	-0.06	0.10	0.599
Phospholipids to total lipds ratio in medium LDL (%)	755	0.00	-0.03	0.03	0.908	755	0.00	-0.03	0.03	0.856
Total cholesterol to total lipids ratio in medium LDL (%)	755	-0.03	-0.11	0.05	0.425	755	-0.03	-0.11	0.05	0.495
Cholesterol esters to total lipids ratio in medium LDL (%)	755	-0.03	-0.11	0.05	0.527	755	-0.02	-0.10	0.06	0.607
Free cholesterol to total lipids ratio in medium LDL (%)	755	0.00	-0.02	0.02	0.766	755	0.00	-0.02	0.02	0.849
Triglycerides to total lipids ratio in medium LDL (%)	755	0.07	-0.01	0.14	0.095	755	0.05	-0.03	0.13	0.206
Phospholipids to total lipds ratio in small LDL (%)	755	0.01	-0.04	0.05	0.786	755	0.00	-0.04	0.05	0.839
Total cholesterol to total lipids ratio in small LDL (%)	755	-0.01	-0.09	0.07	0.761	755	-0.01	-0.09	0.07	0.822
Cholesterol esters to total lipids ratio in small LDL (%)	755	-0.01	-0.09	0.07	0.774	755	-0.01	-0.09	0.07	0.835
Free cholesterol to total lipids ratio in small LDL (%)	755	0.00	-0.04	0.04	0.826	755	0.00	-0.04	0.04	0.876
Triglycerides to total lipids ratio in small LDL (%)	755	0.01	-0.07	0.08	0.806	755	0.01	-0.07	0.08	0.843
Phospholipids to total lipds ratio in very large HDL (%)	755	0.10	0.02	0.19	0.018	755	0.08	-0.01	0.16	0.069
Total cholesterol to total lipids ratio in very large HDL (%)	755	-0.09	-0.18	-0.01	0.027	755	-0.07	-0.15	0.01	0.098
Cholesterol esters to total lipids ratio in very large HDL (%)	755	-0.09	-0.18	-0.01	0.031	755	-0.07	-0.15	0.01	0.108
Free cholesterol to total lipids ratio in very large HDL (%)	755	0.03	-0.05	0.10	0.510	755	0.01	-0.06	0.09	0.740
Triglycerides to total lipids ratio in very large HDL (%)	755	-0.08	-0.17	0.01	0.077	755	-0.06	-0.15	0.02	0.144
Phospholipids to total lipds ratio in large HDL (%)	755	-0.07	-0.15	0.01	0.087	755	-0.04	-0.12	0.04	0.302
Total cholesterol to total lipids ratio in large HDL (%)	755	0.09	0.00	0.18	0.048	755	0.06	-0.03	0.14	0.190
Cholesterol esters to total lipids ratio in large HDL (%)	755	0.09	0.00	0.18	0.047	755	0.06	-0.03	0.14	0.188
Free cholesterol to total lipids ratio in large HDL (%)	755	0.06	-0.02	0.14	0.141	755	0.04	-0.04	0.12	0.346
Triglycerides to total lipids ratio in large HDL (%)	755	-0.10	-0.19	0.00	0.039	755	-0.07	-0.15	0.02	0.128
Phospholipids to total lipds ratio in medium HDL (%)	755	0.10	0.02	0.18	0.017	755	0.09	0.01	0.17	0.029
Total cholesterol to total lipids ratio in medium HDL (%)	755	-0.04	-0.11	0.04	0.381	755	-0.04	-0.12	0.03	0.265
Cholesterol esters to total lipids ratio in medium HDL (%)	755	-0.03	-0.11	0.05	0.433	755	-0.04	-0.12	0.04	0.307
Free cholesterol to total lipids ratio in medium HDL (%)	755	-0.03	-0.11	0.05	0.513	755	-0.03	-0.11	0.05	0.479
Triglycerides to total lipids ratio in medium HDL (%)	755	-0.07	-0.16	0.01	0.099	755	-0.05	-0.13	0.04	0.278
Phospholipids to total lipds ratio in small HDL (%)	755	-0.14	-0.21	-0.07	1.09E-04	755	-0.13	-0.20	-0.06	2.12E-04
Total cholesterol to total lipids ratio in small HDL (%)	755	0.15	0.08	0.22	2.34E-05	755	0.14	0.07	0.21	6.00E-05
Cholesterol esters to total lipids ratio in small HDL (%)	755	0.14	0.07	0.21	5.49E-05	755	0.14	0.07	0.20	1.02E-04
Free cholesterol to total lipids ratio in small HDL (%)	755	0.01	-0.07	0.08	0.876	755	-0.02	-0.09	0.06	0.641
Triglycerides to total lipids ratio in small HDL (%)	755	-0.10	-0.18	-0.02	0.013	755	-0.08	-0.16	-0.01	0.034
Mean diameter for VLDL particles (nm)	755	-0.12	-0.20	-0.04	0.004	755	-0.09	-0.17	-0.02	0.017
Mean diameter for LDL particles (nm)	755	0.02	-0.05	0.09	0.579	755	0.01	-0.06	0.08	0.789
Mean diameter for HDL particles (nm)	755	0.11	0.02	0.19	0.018	755	0.08	-0.01	0.16	0.079
Serum total cholesterol (mmol/l)	755	0.02	-0.05	0.10	0.577	755	0.02	-0.05	0.10	0.542
Total cholesterol in VLDL (mmol/l)	755	-0.08	-0.16	0.00	0.048	755	-0.05	-0.13	0.02	0.181
Remnant cholesterol (non-HDL, non-LDL -cholesterol) (mmol/l)	755	-0.05	-0.13	0.03	0.195	755	-0.03	-0.11	0.05	0.428
Total cholesterol in LDL (mmol/l)	755	0.00	-0.08	0.08	0.995	755	0.01	-0.07	0.08	0.890
Total cholesterol in HDL (mmol/l)	755	0.12	0.04	0.20	0.005	755	0.10	0.02	0.18	0.019
Total cholesterol in HDL2 (mmol/l)	755	0.12	0.04	0.21	0.005	755	0.10	0.02	0.18	0.019
Total cholesterol in HDL3 (mmol/l)	755	0.11	0.03	0.19	0.008	755	0.09	0.01	0.17	0.026
Esterified cholesterol (mmol/l)	755	0.02	-0.05	0.10	0.568	755	0.02	-0.05	0.10	0.544
Free cholesterol (mmol/l)	755	0.02	-0.06	0.10	0.616	755	0.02	-0.05	0.10	0.558
Serum total triglycerides (mmol/l)	755	-0.09	-0.17	-0.02	0.013	755	-0.07	-0.15	0.00	0.045
Triglycerides in VLDL (mmol/l)	755	-0.11	-0.19	-0.03	0.004	755	-0.09	-0.16	-0.01	0.020
Triglycerides in LDL (mmol/l)	755	0.01	-0.06	0.09	0.722	755	0.01	-0.07	0.08	0.850
Triglycerides in HDL (mmol/l)	755	-0.03	-0.11	0.04	0.370	755	-0.02	-0.10	0.05	0.525
Diacylglycerol (mmol/l)	755	0.00	-0.07	0.07	0.932	755	0.01	-0.06	0.08	0.751
Ratio of diacylglycerol to triglycerides	755	0.03	-0.04	0.11	0.390	755	0.04	-0.04	0.11	0.331
Total phosphoglycerides (mmol/l)	755	0.06	-0.01	0.14	0.082	755	0.06	-0.02	0.13	0.129
Ratio of triglycerides to phosphoglycerides	755	-0.09	-0.17	-0.02	0.013	755	-0.07	-0.14	0.00	0.054
Phosphatidylcholine and other cholines (mmol/l)	755	0.07	0.00	0.14	0.043	755	0.07	-0.01	0.14	0.069
Total cholines (mmol/l)	755	0.07	0.00	0.15	0.044	755	0.07	-0.01	0.14	0.071
Apolipoprotein A-I (g/l)	755	0.09	0.01	0.17	0.025	755	0.07	0.00	0.15	0.059
Apolipoprotein B (g/l)	755	-0.07	-0.14	0.01	0.095	755	-0.05	-0.13	0.03	0.238
Ratio of apolipoprotein B to apolipoprotein A-I	755	-0.11	-0.19	-0.02	0.016	755	-0.08	-0.16	0.01	0.067
Total fatty acids (mmol/l)	755	-0.02	-0.09	0.05	0.610	755	-0.01	-0.08	0.06	0.780
Estimated description of fatty acid chain length, not actual carbon number	755	0.00	-0.07	0.08	0.908	755	0.01	-0.06	0.08	0.754
Estimated degree of unsaturation	755	0.05	-0.03	0.13	0.234	755	0.05	-0.03	0.13	0.219
22:6, docosahexaenoic acid (mmol/l)	755	0.02	-0.06	0.09	0.694	755	0.02	-0.06	0.10	0.582
18:2, linoleic acid (mmol/l)	755	0.04	-0.03	0.11	0.227	755	0.04	-0.03	0.11	0.239
Conjugated linoleic acid (mmol/l)	755	0.00	-0.07	0.07	0.999	755	0.00	-0.07	0.07	0.986
Omega-3 fatty acids (mmol/l)	755	-0.02	-0.10	0.06	0.575	755	-0.02	-0.10	0.06	0.682
Omega-6 fatty acids (mmol/l)	755	0.03	-0.04	0.10	0.390	755	0.03	-0.04	0.11	0.364
Polyunsaturated fatty acids (mmol/l)	755	0.03	-0.05	0.10	0.485	755	0.03	-0.04	0.10	0.445
Monounsaturated fatty acids; 16:1, 18:1 (mmol/l)	755	-0.05	-0.12	0.02	0.149	755	-0.04	-0.11	0.03	0.297
Saturated fatty acids (mmol/l)	755	-0.02	-0.10	0.05	0.564	755	-0.02	-0.09	0.06	0.660
Ratio of 22:6 docosahexaenoic acid to total fatty acids (%)	755	0.03	-0.06	0.11	0.539	755	0.03	-0.05	0.11	0.497
Ratio of 18:2 linoleic acid to total fatty acids (%)	755	0.10	0.02	0.18	0.018	755	0.08	0.00	0.16	0.040
Ratio of conjugated linoleic acid to total fatty acids (%)	755	0.00	-0.07	0.07	0.990	755	0.00	-0.07	0.07	0.996
Ratio of omega-3 fatty acids to total fatty acids (%)	755	-0.02	-0.11	0.06	0.567	755	-0.02	-0.11	0.06	0.573
Ratio of omega-6 fatty acids to total fatty acids (%)	755	0.09	0.01	0.17	0.028	755	0.08	0.00	0.16	0.056
Ratio of polyunsaturated fatty acids to total fatty acids (%)	755	0.08	0.00	0.16	0.043	755	0.07	-0.01	0.15	0.081

S2 Table Associations of current total physical activity (CPM at age 15y) with metabolic traits at age 15y in ALSPAC

	**CPM at age 15y (per SD (164) higher)**									
	Adj. for age, sex, ethnicity, maternal education, smoking, alcohol, wear time, wear month					*Additionally adj. for FMI at age 15y*				
**Standardised outcome at age 15y**	**N**	**Beta**	**LCL**	**UCL**	**P-value**	**N**	**Beta**	**LCL**	**UCL**	**P-value**
Ratio of monounsaturated fatty acids to total fatty acids (%)	755	-0.07	-0.15	0.02	0.116	755	-0.05	-0.13	0.03	0.231
Ratio of saturated fatty acids to total fatty acids (%)	755	-0.01	-0.09	0.07	0.780	755	-0.02	-0.10	0.06	0.647
Insulin (mu/l)	755	-0.06	-0.11	-0.01	0.016	755	-0.04	-0.08	0.01	0.122
Glucose (mmol/l)	755	-0.04	-0.11	0.03	0.272	755	-0.03	-0.10	0.04	0.335
Lactate (mmol/l)	755	-0.05	-0.14	0.03	0.212	755	-0.05	-0.14	0.03	0.201
Pyruvate (mmol/l)	755	-0.11	-0.19	-0.03	0.008	755	-0.10	-0.18	-0.02	0.016
Citrate (mmol/l)	755	0.10	0.01	0.19	0.024	755	0.09	0.00	0.17	0.049
Alanine (mmol/l)	755	-0.07	-0.15	0.01	0.096	755	-0.07	-0.15	0.01	0.107
Glutamine (mmol/l)	755	0.05	-0.03	0.12	0.207	755	0.03	-0.04	0.10	0.420
Histidine (mmol/l)	755	0.04	-0.04	0.12	0.353	755	0.04	-0.04	0.12	0.341
Isoleucine (mmol/l)	755	-0.04	-0.11	0.03	0.226	755	-0.03	-0.10	0.04	0.382
Leucine (mmol/l)	755	0.05	-0.02	0.11	0.165	755	0.05	-0.02	0.12	0.135
Valine (mmol/l)	755	0.00	-0.07	0.07	0.959	755	0.01	-0.06	0.08	0.831
Phenylalanine (mmol/l)	755	0.10	0.01	0.19	0.030	755	0.11	0.02	0.20	0.019
Tyrosine (mmol/l)	755	0.08	0.00	0.17	0.063	755	0.09	0.01	0.18	0.032
Acetate (mmol/l)	755	0.07	0.00	0.15	0.063	755	0.07	-0.01	0.14	0.089
Acetoacetate (mmol/l)	755	-0.02	-0.09	0.04	0.446	755	-0.02	-0.09	0.04	0.473
3-hydroxybutyrate (mmol/l)	755	-0.04	-0.11	0.03	0.287	755	-0.04	-0.11	0.04	0.333
Creatinine (mmol/l)	755	-0.09	-0.17	-0.01	0.025	755	-0.09	-0.17	-0.01	0.024
Albumin (signal area)	755	-0.14	-0.22	-0.07	2.41E-04	755	-0.15	-0.22	-0.07	1.69E-04
Glycoprotein acetyls, mainly a1-acid glycoprotein (mmol/l)	755	-0.13	-0.21	-0.06	3.03E-04	755	-0.10	-0.17	-0.03	0.003
C-reactive protein (mg/l)	755	-0.05	-0.11	0.01	0.100	755	-0.05	-0.11	0.02	0.161
