# Supplementary material for: Associations of device-measured physical activity across adolescence with metabolic traits: Prospective cohort study
Source: PLoS Med. 2018 Sep 11;15(9):e1002649. doi: 10.1371/journal.pmed.1002649 (PMC6133272; doi:10.1371/journal.pmed.1002649)
Supplement: S3 Table — ALSPAC, Avon Longitudinal Study of Parents and Children; MVPA, moderate-to-vigorous physical activity. (PDF) [file pmed.1002649.s003.pdf]

S3 Table Associations of current moderate-to-vigorous physical activity (MVPA at age 15y) with metabolic traits at age 15y in ALSPAC

	MVPA at age 15y (per SD (18 min/day) higher)														
	Adj. for age, sex, ethnicity, maternal education, smoking, alcohol, wear time, wear month					*Additionally adj. for SED at age 15y*					*Additionally adj. for FMI at age 15y*				
**Standardised outcome at age 15y**	**N**	**Beta**	**LCL**	**UCL**	**P-value**	**N**	**Beta**	**LCL**	**UCL**	**P-value**	**N**	**Beta**	**LCL**	**UCL**	**P-value**
Systolic blood pressure (mmHg)	1812	-0.06	-0.10	-0.01	0.017	1812	-0.06	-0.11	-0.01	0.017	1782	-0.04	-0.08	0.01	0.141
Diastolic blood pressure (mmHg)	1812	0.00	-0.04	0.05	0.860	1812	0.02	-0.04	0.07	0.559	1782	0.02	-0.03	0.07	0.484
Concentration of chylomicrons and extremely large VLDL particles (mol/l)	1207	-0.16	-0.22	-0.11	3.06E-08	1207	-0.15	-0.21	-0.10	2.51E-07	1195	-0.12	-0.18	-0.07	1.48E-05
Total lipids in chylomicrons and extremely large VLDL (mmol/l)	1207	-0.16	-0.22	-0.10	5.49E-08	1207	-0.15	-0.21	-0.09	5.03E-07	1195	-0.12	-0.17	-0.06	2.34E-05
Phospholipids in chylomicrons and extremely large VLDL (mmol/l)	1207	-0.16	-0.22	-0.11	3.70E-08	1207	-0.15	-0.21	-0.10	3.25E-07	1195	-0.12	-0.18	-0.07	1.49E-05
Total cholesterol in chylomicrons and extremely large VLDL (mmol/l)	1207	-0.15	-0.21	-0.09	7.43E-07	1207	-0.14	-0.20	-0.08	4.11E-06	1195	-0.10	-0.16	-0.05	2.07E-04
Cholesterol esters in chylomicrons and extremely large VLDL (mmol/l)	1207	-0.13	-0.19	-0.07	1.73E-05	1207	-0.12	-0.18	-0.06	6.01E-05	1195	-0.08	-0.14	-0.03	2.70E-03
Free cholesterol in chylomicrons and extremely large VLDL (mmol/l)	1207	-0.16	-0.22	-0.10	5.21E-08	1207	-0.15	-0.21	-0.09	4.30E-07	1195	-0.12	-0.17	-0.07	1.93E-05
Triglycerides in chylomicrons and extremely large VLDL (mmol/l)	1207	-0.16	-0.22	-0.11	3.62E-08	1207	-0.15	-0.21	-0.10	3.69E-07	1195	-0.12	-0.18	-0.07	1.66E-05
Concentration of very large VLDL particles (mol/l)	1207	-0.16	-0.22	-0.10	1.20E-07	1207	-0.15	-0.21	-0.09	1.42E-06	1195	-0.11	-0.17	-0.06	6.19E-05
Total lipids in very large VLDL (mmol/l)	1207	-0.16	-0.22	-0.10	1.85E-07	1207	-0.15	-0.21	-0.09	2.08E-06	1195	-0.11	-0.17	-0.06	8.50E-05
Phospholipids in very large VLDL (mmol/l)	1207	-0.16	-0.21	-0.10	1.39E-07	1207	-0.15	-0.21	-0.09	1.21E-06	1195	-0.11	-0.17	-0.06	5.30E-05
Total cholesterol in very large VLDL (mmol/l)	1207	-0.16	-0.22	-0.10	1.35E-07	1207	-0.15	-0.21	-0.09	1.11E-06	1195	-0.11	-0.17	-0.06	6.50E-05
Cholesterol esters in very large VLDL (mmol/l)	1207	-0.16	-0.21	-0.10	2.59E-07	1207	-0.15	-0.21	-0.09	2.29E-06	1195	-0.11	-0.16	-0.05	1.42E-04
Free cholesterol in very large VLDL (mmol/l)	1207	-0.16	-0.22	-0.10	8.49E-08	1207	-0.15	-0.21	-0.09	6.11E-07	1195	-0.12	-0.17	-0.06	3.23E-05
Triglycerides in very large VLDL (mmol/l)	1207	-0.16	-0.22	-0.10	2.56E-07	1207	-0.14	-0.20	-0.08	3.27E-06	1195	-0.11	-0.17	-0.05	1.16E-04
Concentration of large VLDL particles (mol/l)	1207	-0.15	-0.21	-0.09	5.09E-07	1207	-0.14	-0.20	-0.08	6.35E-06	1195	-0.11	-0.16	-0.05	2.46E-04
Total lipids in large VLDL (mmol/l)	1207	-0.15	-0.21	-0.09	5.72E-07	1207	-0.14	-0.20	-0.08	6.75E-06	1195	-0.11	-0.16	-0.05	2.65E-04
Phospholipids in large VLDL (mmol/l)	1207	-0.15	-0.21	-0.09	4.95E-07	1207	-0.14	-0.20	-0.08	5.39E-06	1195	-0.11	-0.16	-0.05	2.20E-04
Total cholesterol in large VLDL (mmol/l)	1207	-0.15	-0.21	-0.09	9.60E-07	1207	-0.14	-0.20	-0.08	8.71E-06	1195	-0.10	-0.16	-0.05	4.07E-04
Cholesterol esters in large VLDL (mmol/l)	1207	-0.14	-0.20	-0.08	2.66E-06	1207	-0.13	-0.19	-0.07	1.98E-05	1195	-0.10	-0.15	-0.04	9.90E-04
Free cholesterol in large VLDL (mmol/l)	1207	-0.15	-0.21	-0.09	4.85E-07	1207	-0.14	-0.20	-0.08	5.07E-06	1195	-0.11	-0.16	-0.05	2.06E-04
Triglycerides in large VLDL (mmol/l)	1207	-0.15	-0.22	-0.09	5.54E-07	1207	-0.14	-0.20	-0.08	7.28E-06	1195	-0.11	-0.16	-0.05	2.59E-04
Concentration of medium VLDL particles (mol/l)	1207	-0.16	-0.22	-0.10	2.71E-07	1207	-0.15	-0.21	-0.08	3.56E-06	1195	-0.11	-0.17	-0.05	1.90E-04
Total lipids in medium VLDL (mmol/l)	1207	-0.16	-0.22	-0.10	4.31E-07	1207	-0.14	-0.21	-0.08	5.06E-06	1195	-0.11	-0.16	-0.05	2.79E-04
Phospholipids in medium VLDL (mmol/l)	1207	-0.15	-0.21	-0.10	3.97E-07	1207	-0.14	-0.21	-0.08	4.30E-06	1195	-0.11	-0.17	-0.05	2.22E-04
Total cholesterol in medium VLDL (mmol/l)	1207	-0.14	-0.20	-0.08	5.23E-06	1207	-0.13	-0.19	-0.07	3.38E-05	1195	-0.09	-0.15	-0.03	1.65E-03
Cholesterol esters in medium VLDL (mmol/l)	1207	-0.12	-0.18	-0.06	8.21E-05	1207	-0.11	-0.17	-0.05	3.19E-04	1195	-0.08	-0.13	-0.02	0.011
Free cholesterol in medium VLDL (mmol/l)	1207	-0.15	-0.21	-0.09	5.97E-07	1207	-0.14	-0.20	-0.08	6.00E-06	1195	-0.11	-0.16	-0.05	2.65E-04
Triglycerides in medium VLDL (mmol/l)	1207	-0.16	-0.22	-0.10	2.69E-07	1207	-0.15	-0.21	-0.08	3.88E-06	1195	-0.11	-0.17	-0.05	1.95E-04
Concentration of small VLDL particles (mol/l)	1207	-0.15	-0.21	-0.09	6.84E-07	1207	-0.14	-0.21	-0.08	3.64E-06	1195	-0.11	-0.17	-0.05	2.41E-04
Total lipids in small VLDL (mmol/l)	1207	-0.15	-0.21	-0.09	8.36E-07	1207	-0.15	-0.21	-0.09	3.21E-06	1195	-0.11	-0.17	-0.05	2.59E-04
Phospholipids in small VLDL (mmol/l)	1207	-0.14	-0.20	-0.08	5.63E-06	1207	-0.14	-0.20	-0.08	1.30E-05	1195	-0.10	-0.16	-0.04	9.71E-04
Total cholesterol in small VLDL (mmol/l)	1207	-0.13	-0.19	-0.07	1.61E-05	1207	-0.14	-0.20	-0.07	2.63E-05	1195	-0.10	-0.16	-0.04	1.49E-03
Cholesterol esters in small VLDL (mmol/l)	1207	-0.13	-0.19	-0.07	5.05E-05	1207	-0.13	-0.19	-0.07	6.44E-05	1195	-0.10	-0.16	-0.03	2.68E-03
Free cholesterol in small VLDL (mmol/l)	1207	-0.13	-0.19	-0.07	1.07E-05	1207	-0.13	-0.19	-0.07	2.66E-05	1195	-0.10	-0.16	-0.04	1.33E-03
Triglycerides in small VLDL (mmol/l)	1207	-0.15	-0.21	-0.09	9.09E-07	1207	-0.14	-0.20	-0.08	6.69E-06	1195	-0.11	-0.17	-0.05	2.63E-04
Concentration of very small VLDL particles (mol/l)	1207	-0.07	-0.13	-0.01	0.021	1207	-0.07	-0.13	-0.01	0.017	1195	-0.06	-0.12	0.01	0.076
Total lipids in very small VLDL (mmol/l)	1207	-0.09	-0.15	-0.03	0.006	1207	-0.09	-0.15	-0.03	0.005	1195	-0.07	-0.13	0.00	0.035
Phospholipids in very small VLDL (mmol/l)	1207	-0.06	-0.12	0.00	0.061	1207	-0.06	-0.13	0.00	0.038	1195	-0.05	-0.12	0.01	0.089
Total cholesterol in very small VLDL (mmol/l)	1207	-0.07	-0.13	-0.01	0.031	1207	-0.07	-0.13	-0.01	0.031	1195	-0.05	-0.11	0.02	0.155
Cholesterol esters in very small VLDL (mmol/l)	1207	-0.09	-0.15	-0.02	0.007	1207	-0.09	-0.15	-0.02	0.007	1195	-0.06	-0.13	0.00	0.061
Free cholesterol in very small VLDL (mmol/l)	1207	-0.02	-0.08	0.04	0.442	1207	-0.02	-0.08	0.04	0.430	1195	-0.01	-0.07	0.05	0.736
Triglycerides in very small VLDL (mmol/l)	1207	-0.12	-0.17	-0.06	5.10E-05	1207	-0.12	-0.18	-0.06	6.91E-05	1195	-0.10	-0.15	-0.04	9.38E-04
Concentration of IDL particles (mol/l)	1207	-0.04	-0.10	0.02	0.152	1207	-0.05	-0.11	0.01	0.096	1195	-0.05	-0.11	0.01	0.116
Total lipids in IDL (mmol/l)	1207	-0.04	-0.10	0.02	0.152	1207	-0.05	-0.11	0.01	0.097	1195	-0.05	-0.11	0.02	0.148
Phospholipids in IDL (mmol/l)	1207	-0.03	-0.09	0.03	0.271	1207	-0.04	-0.11	0.02	0.152	1195	-0.04	-0.10	0.02	0.173
Total cholesterol in IDL (mmol/l)	1207	-0.05	-0.11	0.01	0.139	1207	-0.05	-0.12	0.01	0.101	1195	-0.04	-0.11	0.02	0.179

S3 Table Associations of current moderate-to-vigorous physical activity (MVPA at age 15y) with metabolic traits at age 15y in ALSPAC

MVPA at age 15y (per SD (18 min/day) higher)

	Adj. for age, sex, ethnicity, maternal education, smoking, alcohol, wear time, wear month					*Additionally adj. for SED at age 15y*					*Additionally adj. for FMI at age 15y*				
**Standardised outcome at age 15y**	**N**	**Beta**	**LCL**	**UCL**	**P-value**	**N**	**Beta**	**LCL**	**UCL**	**P-value**	**N**	**Beta**	**LCL**	**UCL**	**P-value**
Cholesterol esters in IDL (mmol/l)	1207	-0.06	-0.12	0.00	0.066	1207	-0.06	-0.13	0.00	0.052	1195	-0.05	-0.11	0.01	0.129
Free cholesterol in IDL (mmol/l)	1207	-0.02	-0.08	0.04	0.581	1207	-0.03	-0.09	0.03	0.393	1195	-0.03	-0.09	0.03	0.382
Triglycerides in IDL (mmol/l)	1207	-0.04	-0.09	0.02	0.183	1207	-0.05	-0.10	0.01	0.092	1195	-0.05	-0.11	0.00	0.074
Concentration of large LDL particles (mol/l)	1207	-0.05	-0.10	0.01	0.129	1207	-0.06	-0.12	0.00	0.060	1195	-0.05	-0.11	0.01	0.090
Total lipids in large LDL (mmol/l)	1207	-0.04	-0.10	0.02	0.150	1207	-0.05	-0.12	0.01	0.077	1195	-0.05	-0.11	0.01	0.120
Phospholipids in large LDL (mmol/l)	1207	-0.05	-0.11	0.01	0.101	1207	-0.06	-0.12	0.00	0.047	1195	-0.05	-0.11	0.01	0.096
Total cholesterol in large LDL (mmol/l)	1207	-0.04	-0.10	0.02	0.164	1207	-0.05	-0.11	0.01	0.091	1195	-0.05	-0.11	0.02	0.144
Cholesterol esters in large LDL (mmol/l)	1207	-0.05	-0.11	0.01	0.112	1207	-0.06	-0.12	0.00	0.062	1195	-0.05	-0.11	0.01	0.114
Free cholesterol in large LDL (mmol/l)	1207	-0.02	-0.08	0.04	0.430	1207	-0.04	-0.10	0.03	0.250	1195	-0.03	-0.10	0.03	0.279
Triglycerides in large LDL (mmol/l)	1207	-0.02	-0.08	0.03	0.387	1207	-0.04	-0.09	0.02	0.188	1195	-0.04	-0.10	0.01	0.109
Concentration of medium LDL particles (mol/l)	1207	-0.06	-0.12	0.00	0.043	1207	-0.07	-0.13	-0.01	0.017	1195	-0.06	-0.12	0.00	0.037
Total lipids in medium LDL (mmol/l)	1207	-0.05	-0.11	0.00	0.071	1207	-0.07	-0.13	-0.01	0.032	1195	-0.06	-0.12	0.00	0.065
Phospholipids in medium LDL (mmol/l)	1207	-0.07	-0.13	-0.01	0.027	1207	-0.08	-0.14	-0.02	0.012	1195	-0.06	-0.12	0.00	0.047
Total cholesterol in medium LDL (mmol/l)	1207	-0.05	-0.11	0.01	0.083	1207	-0.06	-0.12	0.00	0.042	1195	-0.06	-0.12	0.01	0.080
Cholesterol esters in medium LDL (mmol/l)	1207	-0.05	-0.11	0.00	0.068	1207	-0.07	-0.13	-0.01	0.034	1195	-0.06	-0.12	0.00	0.066
Free cholesterol in medium LDL (mmol/l)	1207	-0.04	-0.10	0.02	0.186	1207	-0.05	-0.11	0.01	0.105	1195	-0.04	-0.11	0.02	0.175
Triglycerides in medium LDL (mmol/l)	1207	-0.02	-0.08	0.03	0.450	1207	-0.04	-0.09	0.02	0.180	1195	-0.05	-0.10	0.01	0.105
Concentration of small LDL particles (mol/l)	1207	-0.06	-0.12	0.00	0.042	1207	-0.07	-0.13	-0.01	0.019	1195	-0.06	-0.12	0.00	0.040
Total lipids in small LDL (mmol/l)	1207	-0.06	-0.11	0.00	0.059	1207	-0.07	-0.13	-0.01	0.028	1195	-0.06	-0.12	0.00	0.062
Phospholipids in small LDL (mmol/l)	1207	-0.06	-0.12	0.00	0.047	1207	-0.07	-0.13	-0.01	0.026	1195	-0.06	-0.12	0.00	0.071
Total cholesterol in small LDL (mmol/l)	1207	-0.05	-0.11	0.01	0.092	1207	-0.06	-0.12	0.00	0.046	1195	-0.05	-0.12	0.01	0.087
Cholesterol esters in small LDL (mmol/l)	1207	-0.05	-0.11	0.01	0.079	1207	-0.06	-0.12	0.00	0.037	1195	-0.06	-0.12	0.00	0.069
Free cholesterol in small LDL (mmol/l)	1207	-0.04	-0.10	0.02	0.207	1207	-0.05	-0.11	0.01	0.136	1195	-0.04	-0.10	0.03	0.264
Triglycerides in small LDL (mmol/l)	1207	-0.07	-0.13	-0.02	0.008	1207	-0.08	-0.14	-0.03	2.60E-03	1195	-0.08	-0.13	-0.02	0.006
Concentration of very large HDL particles (mol/l)	1207	0.12	0.06	0.18	2.10E-04	1207	0.12	0.05	0.18	4.75E-04	1195	0.08	0.02	0.14	0.013
Total lipids in very large HDL (mmol/l)	1207	0.12	0.05	0.18	3.28E-04	1207	0.12	0.05	0.18	6.21E-04	1195	0.08	0.01	0.14	0.016
Phospholipids in very large HDL (mmol/l)	1207	0.13	0.06	0.19	7.94E-05	1207	0.12	0.06	0.19	2.28E-04	1195	0.08	0.02	0.15	0.008
Total cholesterol in very large HDL (mmol/l)	1207	0.10	0.04	0.16	1.67E-03	1207	0.10	0.04	0.17	2.20E-03	1195	0.07	0.01	0.13	0.035
Cholesterol esters in very large HDL (mmol/l)	1207	0.09	0.03	0.16	0.003	1207	0.10	0.03	0.16	0.004	1195	0.07	0.00	0.13	0.048
Free cholesterol in very large HDL (mmol/l)	1207	0.11	0.05	0.18	4.18E-04	1207	0.11	0.05	0.18	7.03E-04	1195	0.08	0.01	0.14	0.017
Triglycerides in very large HDL (mmol/l)	1207	0.00	-0.06	0.06	0.966	1207	0.01	-0.05	0.07	0.664	1195	0.00	-0.06	0.05	0.899
Concentration of large HDL particles (mol/l)	1207	0.14	0.08	0.20	1.37E-05	1207	0.13	0.07	0.20	5.90E-05	1195	0.10	0.03	0.16	2.20E-03
Total lipids in large HDL (mmol/l)	1207	0.14	0.08	0.20	1.40E-05	1207	0.13	0.07	0.20	5.67E-05	1195	0.10	0.03	0.16	2.26E-03
Phospholipids in large HDL (mmol/l)	1207	0.13	0.07	0.19	5.90E-05	1207	0.12	0.06	0.18	2.64E-04	1195	0.09	0.02	0.15	0.006
Total cholesterol in large HDL (mmol/l)	1207	0.15	0.09	0.21	3.63E-06	1207	0.14	0.08	0.21	1.41E-05	1195	0.11	0.04	0.17	8.74E-04
Cholesterol esters in large HDL (mmol/l)	1207	0.15	0.09	0.21	3.09E-06	1207	0.15	0.08	0.21	1.21E-05	1195	0.11	0.04	0.17	7.81E-04
Free cholesterol in large HDL (mmol/l)	1207	0.14	0.08	0.20	7.40E-06	1207	0.14	0.07	0.20	2.74E-05	1195	0.10	0.04	0.16	1.43E-03
Triglycerides in large HDL (mmol/l)	1207	0.03	-0.02	0.09	0.257	1207	0.04	-0.02	0.10	0.198	1195	0.03	-0.03	0.08	0.385
Concentration of medium HDL particles (mol/l)	1207	0.05	-0.01	0.10	0.097	1207	0.03	-0.02	0.09	0.267	1195	0.04	-0.02	0.09	0.220
Total lipids in medium HDL (mmol/l)	1207	0.05	0.00	0.11	0.057	1207	0.04	-0.02	0.10	0.173	1195	0.04	-0.02	0.10	0.179
Phospholipids in medium HDL (mmol/l)	1207	0.06	0.00	0.11	0.044	1207	0.04	-0.02	0.10	0.165	1195	0.04	-0.02	0.10	0.174
Total cholesterol in medium HDL (mmol/l)	1207	0.07	0.01	0.13	0.017	1207	0.06	0.00	0.12	0.051	1195	0.05	-0.01	0.12	0.081
Cholesterol esters in medium HDL (mmol/l)	1207	0.07	0.02	0.13	0.012	1207	0.06	0.00	0.12	0.036	1195	0.06	0.00	0.12	0.062
Free cholesterol in medium HDL (mmol/l)	1207	0.05	-0.01	0.11	0.093	1207	0.04	-0.02	0.10	0.223	1195	0.03	-0.03	0.09	0.255
Triglycerides in medium HDL (mmol/l)	1207	-0.11	-0.17	-0.06	8.51E-05	1207	-0.12	-0.17	-0.06	7.46E-05	1195	-0.08	-0.14	-0.03	0.004
Concentration of small HDL particles (mol/l)	1207	-0.06	-0.11	0.00	0.038	1207	-0.08	-0.13	-0.02	0.010	1195	-0.05	-0.11	0.01	0.105
Total lipids in small HDL (mmol/l)	1207	-0.02	-0.07	0.04	0.559	1207	-0.04	-0.09	0.02	0.177	1195	-0.02	-0.07	0.04	0.514
Phospholipids in small HDL (mmol/l)	1207	-0.06	-0.11	0.00	0.042	1207	-0.07	-0.13	-0.01	0.025	1195	-0.04	-0.10	0.01	0.143

S3 Table Associations of current moderate-to-vigorous physical activity (MVPA at age 15y) with metabolic traits at age 15y in ALSPAC

	**MVPA at age 15y (per SD (18 min/day) higher)**														
	Adj. for age, sex, ethnicity, maternal education, smoking, alcohol, wear time, wear month					*Additionally adj. for SED at age 15y*					*Additionally adj. for FMI at age 15y*				
**Standardised outcome at age 15y**	**N**	**Beta**	**LCL**	**UCL**	**P-value**	**N**	**Beta**	**LCL**	**UCL**	**P-value**	**N**	**Beta**	**LCL**	**UCL**	**P-value**
Total cholesterol in small HDL (mmol/l)	1207	0.05	0.00	0.11	0.051	1207	0.02	-0.03	0.08	0.399	1195	0.03	-0.02	0.08	0.287
Cholesterol esters in small HDL (mmol/l)	1207	0.06	0.00	0.11	0.044	1207	0.02	-0.03	0.08	0.386	1195	0.03	-0.03	0.08	0.290
Free cholesterol in small HDL (mmol/l)	1207	0.02	-0.03	0.08	0.440	1207	0.01	-0.05	0.07	0.718	1195	0.02	-0.04	0.08	0.556
Triglycerides in small HDL (mmol/l)	1207	-0.13	-0.18	-0.07	1.20E-05	1207	-0.13	-0.19	-0.07	1.03E-05	1195	-0.10	-0.16	-0.05	2.83E-04
Phospholipids to total lipds ratio in chylomicrons and extremely large VLDL (%)	1207	-0.07	-0.13	-0.01	0.025	1207	-0.07	-0.13	-0.01	0.023	1195	-0.07	-0.13	-0.01	0.025
Total cholesterol to total lipids ratio in chylomicrons and extremely large VLDL (%)	1207	-0.04	-0.10	0.02	0.180	1207	-0.05	-0.11	0.02	0.143	1195	-0.02	-0.08	0.04	0.472
Cholesterol esters to total lipids ratio in chylomicrons and extremely large VLDL (%)	1207	-0.01	-0.07	0.04	0.633	1207	-0.02	-0.08	0.04	0.477	1195	0.00	-0.06	0.06	0.969
Free cholesterol to total lipids ratio in chylomicrons and extremely large VLDL (%)	1207	-0.10	-0.16	-0.04	1.30E-03	1207	-0.09	-0.16	-0.03	2.45E-03	1195	-0.08	-0.14	-0.02	0.010
Triglycerides to total lipids ratio in chylomicrons and extremely large VLDL (%)	1207	0.03	-0.01	0.07	0.142	1207	0.04	-0.01	0.08	0.103	1195	0.02	-0.02	0.06	0.368
Phospholipids to total lipds ratio in very large VLDL (%)	1207	-0.11	-0.17	-0.05	5.87E-04	1207	-0.11	-0.17	-0.05	7.94E-04	1195	-0.09	-0.16	-0.03	0.004
Total cholesterol to total lipids ratio in very large VLDL (%)	1207	0.11	0.02	0.21	0.021	1207	0.10	0.01	0.19	0.038	1195	0.10	0.00	0.19	0.056
Cholesterol esters to total lipids ratio in very large VLDL (%)	1207	0.10	0.03	0.17	2.89E-03	1207	0.09	0.02	0.16	0.010	1195	0.08	0.02	0.15	0.015
Free cholesterol to total lipids ratio in very large VLDL (%)	1207	0.06	0.00	0.12	0.069	1207	0.04	-0.02	0.11	0.204	1195	0.04	-0.03	0.10	0.299
Triglycerides to total lipids ratio in very large VLDL (%)	1207	-0.06	-0.12	0.01	0.092	1207	-0.04	-0.11	0.03	0.235	1195	-0.04	-0.11	0.03	0.244
Phospholipids to total lipds ratio in large VLDL (%)	1207	-0.11	-0.17	-0.04	1.51E-03	1207	-0.10	-0.17	-0.04	0.003	1195	-0.09	-0.16	-0.02	0.010
Total cholesterol to total lipids ratio in large VLDL (%)	1207	-0.05	-0.12	0.01	0.094	1207	-0.05	-0.12	0.01	0.097	1195	-0.04	-0.10	0.03	0.241
Cholesterol esters to total lipids ratio in large VLDL (%)	1207	0.07	-0.07	0.21	0.333	1207	0.06	-0.07	0.19	0.351	1195	0.07	-0.07	0.21	0.329
Free cholesterol to total lipids ratio in large VLDL (%)	1207	-0.12	-0.18	-0.05	2.76E-04	1207	-0.11	-0.18	-0.05	8.81E-04	1195	-0.09	-0.16	-0.03	0.006
Triglycerides to total lipids ratio in large VLDL (%)	1207	0.09	-0.07	0.26	0.251	1207	0.09	-0.06	0.23	0.248	1195	0.09	-0.07	0.25	0.263
Phospholipids to total lipds ratio in medium VLDL (%)	1207	0.08	0.01	0.14	0.020	1207	0.07	0.00	0.14	0.053	1195	0.04	-0.03	0.10	0.254
Total cholesterol to total lipids ratio in medium VLDL (%)	1207	0.00	-0.06	0.05	0.882	1207	-0.01	-0.07	0.05	0.854	1195	0.00	-0.06	0.06	0.972
Cholesterol esters to total lipids ratio in medium VLDL (%)	1207	0.02	-0.04	0.07	0.614	1207	0.01	-0.05	0.07	0.660	1195	0.02	-0.04	0.08	0.516
Free cholesterol to total lipids ratio in medium VLDL (%)	1207	-0.06	-0.12	-0.01	0.033	1207	-0.06	-0.12	0.00	0.038	1195	-0.06	-0.12	0.00	0.048
Triglycerides to total lipids ratio in medium VLDL (%)	1207	-0.01	-0.07	0.05	0.659	1207	-0.01	-0.07	0.05	0.752	1195	-0.01	-0.07	0.05	0.749
Phospholipids to total lipds ratio in small VLDL (%)	1207	0.15	0.09	0.21	8.51E-07	1207	0.13	0.07	0.20	1.75E-05	1195	0.12	0.06	0.18	1.04E-04
Total cholesterol to total lipids ratio in small VLDL (%)	1207	0.02	-0.05	0.08	0.606	1207	0.01	-0.06	0.07	0.835	1195	0.01	-0.06	0.07	0.864
Cholesterol esters to total lipids ratio in small VLDL (%)	1207	0.00	-0.07	0.06	0.942	1207	-0.01	-0.08	0.05	0.738	1195	-0.01	-0.08	0.06	0.777
Free cholesterol to total lipids ratio in small VLDL (%)	1207	0.13	0.07	0.19	2.21E-05	1207	0.12	0.06	0.18	1.47E-04	1195	0.10	0.04	0.16	1.01E-03
Triglycerides to total lipids ratio in small VLDL (%)	1207	-0.06	-0.12	0.00	0.061	1207	-0.05	-0.11	0.02	0.159	1195	-0.04	-0.11	0.02	0.213
Phospholipids to total lipds ratio in very small VLDL (%)	1207	0.01	-0.05	0.07	0.763	1207	0.00	-0.06	0.06	0.986	1195	-0.01	-0.07	0.05	0.637
Total cholesterol to total lipids ratio in very small VLDL (%)	1207	0.05	-0.01	0.10	0.106	1207	0.05	-0.01	0.11	0.083	1195	0.06	0.00	0.11	0.061
Cholesterol esters to total lipids ratio in very small VLDL (%)	1207	0.00	-0.06	0.05	0.927	1207	0.00	-0.06	0.06	0.978	1195	0.01	-0.05	0.07	0.743
Free cholesterol to total lipids ratio in very small VLDL (%)	1207	0.12	0.07	0.18	3.63E-06	1207	0.13	0.08	0.19	1.37E-06	1195	0.12	0.07	0.18	1.65E-05
Triglycerides to total lipids ratio in very small VLDL (%)	1207	-0.06	-0.12	0.00	0.040	1207	-0.06	-0.12	0.00	0.054	1195	-0.05	-0.11	0.01	0.073
Phospholipids to total lipds ratio in IDL (%)	1207	0.08	0.02	0.14	0.006	1207	0.06	0.01	0.12	0.030	1195	0.04	-0.02	0.10	0.194
Total cholesterol to total lipids ratio in IDL (%)	1207	-0.04	-0.10	0.02	0.207	1207	-0.03	-0.09	0.03	0.378	1195	-0.01	-0.07	0.05	0.714
Cholesterol esters to total lipids ratio in IDL (%)	1207	-0.08	-0.14	-0.02	0.006	1207	-0.07	-0.13	-0.01	0.024	1195	-0.04	-0.10	0.02	0.170
Free cholesterol to total lipids ratio in IDL (%)	1207	0.10	0.04	0.16	1.11E-03	1207	0.10	0.03	0.16	2.56E-03	1195	0.07	0.01	0.13	0.021
Triglycerides to total lipids ratio in IDL (%)	1207	0.01	-0.05	0.07	0.777	1207	0.00	-0.06	0.06	0.927	1195	0.00	-0.07	0.06	0.884
Phospholipids to total lipds ratio in large LDL (%)	1207	0.01	-0.04	0.07	0.627	1207	0.01	-0.05	0.07	0.681	1195	0.02	-0.04	0.08	0.510
Total cholesterol to total lipids ratio in large LDL (%)	1207	-0.03	-0.09	0.03	0.393	1207	-0.02	-0.09	0.04	0.496	1195	-0.02	-0.08	0.04	0.553
Cholesterol esters to total lipids ratio in large LDL (%)	1207	-0.06	-0.12	0.00	0.062	1207	-0.05	-0.12	0.01	0.091	1195	-0.05	-0.11	0.02	0.154
Free cholesterol to total lipids ratio in large LDL (%)	1207	0.10	0.05	0.16	1.72E-04	1207	0.11	0.05	0.16	2.08E-04	1195	0.09	0.03	0.14	1.82E-03
Triglycerides to total lipids ratio in large LDL (%)	1207	0.03	-0.03	0.09	0.333	1207	0.02	-0.04	0.09	0.448	1195	0.01	-0.05	0.07	0.749
Phospholipids to total lipds ratio in medium LDL (%)	1207	0.01	-0.01	0.03	0.496	1207	0.01	-0.01	0.03	0.493	1195	0.01	-0.01	0.03	0.367
Total cholesterol to total lipids ratio in medium LDL (%)	1207	-0.04	-0.10	0.03	0.241	1207	-0.03	-0.10	0.03	0.323	1195	-0.03	-0.10	0.03	0.336
Cholesterol esters to total lipids ratio in medium LDL (%)	1207	-0.05	-0.12	0.01	0.102	1207	-0.05	-0.12	0.01	0.121	1195	-0.05	-0.11	0.02	0.146
Free cholesterol to total lipids ratio in medium LDL (%)	1207	0.02	0.00	0.03	0.037	1207	0.02	0.00	0.04	0.031	1195	0.02	0.00	0.03	0.048

S3 Table Associations of current moderate-to-vigorous physical activity (MVPA at age 15y) with metabolic traits at age 15y in ALSPAC

	MVPA at age 15y (per SD (18 min/day) higher)														
	Adj. for age, sex, ethnicity, maternal education, smoking, alcohol, wear time, wear month					*Additionally adj. for SED at age 15y*					*Additionally adj. for FMI at age 15y*				
**Standardised outcome at age 15y**	**N**	**Beta**	**LCL**	**UCL**	**P-value**	**N**	**Beta**	**LCL**	**UCL**	**P-value**	**N**	**Beta**	**LCL**	**UCL**	**P-value**
Triglycerides to total lipids ratio in medium LDL (%)	1207	0.04	-0.02	0.10	0.155	1207	0.03	-0.03	0.09	0.328	1195	0.01	-0.05	0.07	0.669
Phospholipids to total lipds ratio in small LDL (%)	1207	0.03	-0.01	0.06	0.165	1207	0.03	-0.01	0.06	0.152	1195	0.03	-0.01	0.06	0.159
Total cholesterol to total lipids ratio in small LDL (%)	1207	-0.03	-0.09	0.04	0.421	1207	-0.03	-0.09	0.04	0.439	1195	-0.03	-0.09	0.04	0.434
Cholesterol esters to total lipids ratio in small LDL (%)	1207	-0.04	-0.10	0.02	0.177	1207	-0.04	-0.11	0.02	0.171	1195	-0.04	-0.11	0.02	0.183
Free cholesterol to total lipids ratio in small LDL (%)	1207	0.03	0.00	0.06	0.036	1207	0.04	0.00	0.07	0.027	1195	0.03	0.00	0.07	0.037
Triglycerides to total lipids ratio in small LDL (%)	1207	-0.04	-0.10	0.01	0.148	1207	-0.05	-0.11	0.01	0.096	1195	-0.05	-0.10	0.01	0.112
Phospholipids to total lipds ratio in very large HDL (%)	1207	0.13	0.06	0.19	8.05E-05	1207	0.12	0.05	0.19	3.72E-04	1195	0.08	0.02	0.14	0.009
Total cholesterol to total lipids ratio in very large HDL (%)	1207	-0.11	-0.17	-0.05	4.25E-04	1207	-0.11	-0.17	-0.04	1.41E-03	1195	-0.07	-0.13	-0.01	0.024
Cholesterol esters to total lipids ratio in very large HDL (%)	1207	-0.11	-0.17	-0.05	4.37E-04	1207	-0.11	-0.17	-0.04	1.26E-03	1195	-0.07	-0.13	-0.01	0.022
Free cholesterol to total lipids ratio in very large HDL (%)	1207	0.05	-0.01	0.10	0.108	1207	0.06	0.00	0.12	0.052	1195	0.04	-0.02	0.10	0.166
Triglycerides to total lipids ratio in very large HDL (%)	1207	-0.11	-0.17	-0.06	1.23E-04	1207	-0.10	-0.16	-0.04	6.15E-04	1195	-0.08	-0.14	-0.02	0.006
Phospholipids to total lipds ratio in large HDL (%)	1207	-0.14	-0.19	-0.08	1.25E-06	1207	-0.15	-0.21	-0.09	3.86E-07	1195	-0.11	-0.16	-0.05	1.39E-04
Total cholesterol to total lipids ratio in large HDL (%)	1207	0.15	0.09	0.21	3.66E-07	1207	0.16	0.10	0.22	3.45E-07	1195	0.11	0.06	0.17	1.17E-04
Cholesterol esters to total lipids ratio in large HDL (%)	1207	0.15	0.09	0.21	5.42E-07	1207	0.16	0.10	0.22	5.85E-07	1195	0.11	0.05	0.17	1.76E-04
Free cholesterol to total lipids ratio in large HDL (%)	1207	0.11	0.06	0.17	5.61E-05	1207	0.12	0.06	0.18	3.72E-05	1195	0.09	0.03	0.14	2.25E-03
Triglycerides to total lipids ratio in large HDL (%)	1207	-0.14	-0.20	-0.08	7.78E-06	1207	-0.13	-0.19	-0.07	5.08E-05	1195	-0.09	-0.15	-0.03	2.67E-03
Phospholipids to total lipds ratio in medium HDL (%)	1207	0.05	-0.01	0.11	0.077	1207	0.03	-0.03	0.09	0.260	1195	0.03	-0.03	0.09	0.328
Total cholesterol to total lipids ratio in medium HDL (%)	1207	0.04	-0.02	0.10	0.177	1207	0.05	-0.01	0.11	0.073	1195	0.04	-0.02	0.09	0.228
Cholesterol esters to total lipids ratio in medium HDL (%)	1207	0.05	-0.01	0.10	0.112	1207	0.06	0.00	0.12	0.044	1195	0.04	-0.02	0.10	0.157
Free cholesterol to total lipids ratio in medium HDL (%)	1207	-0.02	-0.08	0.04	0.587	1207	-0.01	-0.08	0.05	0.725	1195	-0.02	-0.08	0.05	0.640
Triglycerides to total lipids ratio in medium HDL (%)	1207	-0.14	-0.20	-0.08	4.05E-06	1207	-0.14	-0.20	-0.08	7.88E-06	1195	-0.10	-0.16	-0.04	6.38E-04
Phospholipids to total lipds ratio in small HDL (%)	1207	-0.08	-0.14	-0.02	0.005	1207	-0.06	-0.11	0.00	0.058	1195	-0.05	-0.11	0.01	0.090
Total cholesterol to total lipids ratio in small HDL (%)	1207	0.11	0.05	0.16	2.16E-04	1207	0.08	0.02	0.14	0.005	1195	0.07	0.01	0.13	0.014
Cholesterol esters to total lipids ratio in small HDL (%)	1207	0.09	0.03	0.14	2.43E-03	1207	0.06	0.00	0.12	0.038	1195	0.06	0.00	0.11	0.058
Free cholesterol to total lipids ratio in small HDL (%)	1207	0.09	0.03	0.15	2.14E-03	1207	0.12	0.06	0.18	1.88E-04	1195	0.09	0.03	0.15	0.005
Triglycerides to total lipids ratio in small HDL (%)	1207	-0.13	-0.19	-0.08	4.65E-06	1207	-0.13	-0.19	-0.07	1.47E-05	1195	-0.11	-0.17	-0.05	1.91E-04
Mean diameter for VLDL particles (nm)	1207	-0.15	-0.21	-0.08	3.73E-06	1207	-0.13	-0.20	-0.07	4.43E-05	1195	-0.10	-0.16	-0.04	8.60E-04
Mean diameter for LDL particles (nm)	1207	0.07	0.02	0.12	0.012	1207	0.07	0.02	0.13	0.010	1195	0.05	0.00	0.11	0.052
Mean diameter for HDL particles (nm)	1207	0.14	0.08	0.21	1.52E-05	1207	0.14	0.08	0.21	3.19E-05	1195	0.10	0.04	0.17	1.88E-03
Serum total cholesterol (mmol/l)	1207	-0.02	-0.08	0.03	0.420	1207	-0.03	-0.09	0.03	0.277	1195	-0.03	-0.09	0.03	0.359
Total cholesterol in VLDL (mmol/l)	1207	-0.14	-0.20	-0.08	3.96E-06	1207	-0.14	-0.20	-0.08	1.55E-05	1195	-0.10	-0.16	-0.04	1.13E-03
Remnant cholesterol (non-HDL, non-LDL -cholesterol) (mmol/l)	1207	-0.11	-0.17	-0.05	4.49E-04	1207	-0.11	-0.17	-0.05	6.68E-04	1195	-0.08	-0.14	-0.02	0.010
Total cholesterol in LDL (mmol/l)	1207	-0.05	-0.11	0.01	0.120	1207	-0.06	-0.12	0.00	0.063	1195	-0.05	-0.11	0.01	0.110
Total cholesterol in HDL (mmol/l)	1207	0.12	0.06	0.18	6.82E-05	1207	0.11	0.05	0.18	3.97E-04	1195	0.09	0.02	0.15	0.006
Total cholesterol in HDL2 (mmol/l)	1207	0.13	0.07	0.20	1.80E-05	1207	0.12	0.06	0.19	1.47E-04	1195	0.09	0.03	0.15	0.003
Total cholesterol in HDL3 (mmol/l)	1207	0.10	0.04	0.16	1.25E-03	1207	0.09	0.03	0.15	0.004	1195	0.07	0.01	0.13	0.024
Esterified cholesterol (mmol/l)	1207	-0.02	-0.08	0.04	0.465	1207	-0.03	-0.09	0.03	0.297	1195	-0.03	-0.09	0.03	0.379
Free cholesterol (mmol/l)	1207	-0.03	-0.09	0.03	0.349	1207	-0.04	-0.10	0.03	0.256	1195	-0.03	-0.09	0.03	0.335
Serum total triglycerides (mmol/l)	1207	-0.15	-0.21	-0.09	6.59E-07	1207	-0.14	-0.20	-0.08	3.79E-06	1195	-0.11	-0.17	-0.05	1.32E-04
Triglycerides in VLDL (mmol/l)	1207	-0.16	-0.22	-0.10	2.72E-07	1207	-0.15	-0.21	-0.09	3.18E-06	1195	-0.11	-0.17	-0.05	1.43E-04
Triglycerides in LDL (mmol/l)	1207	-0.03	-0.09	0.02	0.244	1207	-0.05	-0.10	0.01	0.098	1195	-0.05	-0.11	0.00	0.065
Triglycerides in HDL (mmol/l)	1207	-0.09	-0.14	-0.03	1.30E-03	1207	-0.09	-0.14	-0.03	1.70E-03	1195	-0.07	-0.12	-0.02	0.011
Diacylglycerol (mmol/l)	1166	-0.05	-0.11	0.01	0.083	1166	-0.04	-0.11	0.02	0.169	1155	-0.02	-0.08	0.05	0.617
Ratio of diacylglycerol to triglycerides	1166	0.01	-0.05	0.07	0.687	1166	0.01	-0.05	0.08	0.706	1155	0.02	-0.04	0.09	0.481
Total phosphoglycerides (mmol/l)	1207	0.02	-0.04	0.07	0.520	1207	0.01	-0.05	0.06	0.788	1195	0.00	-0.06	0.06	0.986
Ratio of triglycerides to phosphoglycerides	1207	-0.13	-0.19	-0.07	1.52E-05	1207	-0.11	-0.17	-0.05	2.23E-04	1195	-0.08	-0.13	-0.02	0.006
Phosphatidylcholine and other cholines (mmol/l)	1185	0.03	-0.03	0.08	0.325	1185	0.03	-0.03	0.08	0.360	1173	0.02	-0.04	0.07	0.555
Total cholines (mmol/l)	1203	0.03	-0.03	0.09	0.293	1203	0.02	-0.04	0.08	0.442	1191	0.01	-0.05	0.07	0.672

S3 Table Associations of current moderate-to-vigorous physical activity (MVPA at age 15y) with metabolic traits at age 15y in ALSPAC

	MVPA at age 15y (per SD (18 min/day) higher)														
	Adj. for age, sex, ethnicity, maternal education, smoking, alcohol, wear time, wear month					*Additionally adj. for SED at age 15y*					*Additionally adj. for FMI at age 15y*				
**Standardised outcome at age 15y**	**N**	**Beta**	**LCL**	**UCL**	**P-value**	**N**	**Beta**	**LCL**	**UCL**	**P-value**	**N**	**Beta**	**LCL**	**UCL**	**P-value**
Apolipoprotein A-I (g/l)	1207	0.07	0.01	0.13	0.014	1207	0.06	0.00	0.12	0.039	1195	0.05	-0.01	0.11	0.125
Apolipoprotein B (g/l)	1207	-0.13	-0.19	-0.07	2.59E-05	1207	-0.13	-0.19	-0.07	4.83E-05	1195	-0.10	-0.16	-0.04	1.15E-03
Ratio of apolipoprotein B to apolipoprotein A-I	1207	-0.16	-0.23	-0.10	3.16E-07	1207	-0.16	-0.22	-0.09	1.68E-06	1195	-0.12	-0.19	-0.06	1.27E-04
Total fatty acids (mmol/l)	1207	-0.06	-0.12	0.00	0.037	1207	-0.06	-0.12	0.00	0.037	1195	-0.05	-0.11	0.01	0.108
Estimated description of fatty acid chain length, not actual carbon number	1201	-0.02	-0.08	0.05	0.590	1201	-0.01	-0.08	0.05	0.719	1189	-0.01	-0.07	0.06	0.798
Estimated degree of unsaturation	1206	0.03	-0.03	0.10	0.356	1206	0.03	-0.04	0.10	0.351	1194	0.03	-0.04	0.10	0.414
22:6, docosahexaenoic acid (mmol/l)	1207	0.01	-0.05	0.07	0.817	1207	0.00	-0.06	0.06	0.913	1195	0.00	-0.06	0.06	0.900
18:2, linoleic acid (mmol/l)	1203	-0.02	-0.08	0.03	0.425	1203	-0.03	-0.08	0.03	0.345	1191	-0.03	-0.09	0.03	0.309
Conjugated linoleic acid (mmol/l)	1207	-0.03	-0.09	0.02	0.250	1207	-0.02	-0.08	0.04	0.552	1195	-0.02	-0.08	0.04	0.531
Omega-3 fatty acids (mmol/l)	1205	-0.02	-0.08	0.05	0.619	1205	-0.02	-0.09	0.04	0.461	1193	-0.02	-0.09	0.04	0.533
Omega-6 fatty acids (mmol/l)	1204	-0.03	-0.09	0.03	0.280	1204	-0.03	-0.09	0.02	0.245	1192	-0.03	-0.09	0.03	0.288
Polyunsaturated fatty acids (mmol/l)	1202	-0.03	-0.08	0.03	0.368	1202	-0.03	-0.09	0.03	0.308	1190	-0.03	-0.09	0.03	0.357
Monounsaturated fatty acids; 16:1, 18:1 (mmol/l)	1204	-0.10	-0.16	-0.05	2.91E-04	1204	-0.11	-0.16	-0.05	3.68E-04	1192	-0.08	-0.14	-0.02	0.005
Saturated fatty acids (mmol/l)	1202	-0.04	-0.10	0.03	0.254	1202	-0.04	-0.10	0.03	0.279	1190	-0.03	-0.09	0.04	0.447
Ratio of 22:6 docosahexaenoic acid to total fatty acids (%)	1207	0.04	-0.02	0.10	0.209	1207	0.03	-0.03	0.09	0.356	1195	0.03	-0.03	0.09	0.352
Ratio of 18:2 linoleic acid to total fatty acids (%)	1203	0.06	-0.01	0.13	0.071	1203	0.06	-0.01	0.13	0.089	1191	0.03	-0.03	0.10	0.333
Ratio of conjugated linoleic acid to total fatty acids (%)	1207	-0.03	-0.09	0.03	0.342	1207	-0.01	-0.08	0.05	0.699	1195	-0.02	-0.08	0.05	0.635
Ratio of omega-3 fatty acids to total fatty acids (%)	1205	0.03	-0.03	0.09	0.388	1205	0.02	-0.04	0.09	0.536	1193	0.01	-0.05	0.08	0.712
Ratio of omega-6 fatty acids to total fatty acids (%)	1204	0.07	0.00	0.13	0.036	1204	0.07	0.00	0.13	0.049	1192	0.04	-0.02	0.11	0.185
Ratio of polyunsaturated fatty acids to total fatty acids (%)	1202	0.07	0.01	0.13	0.027	1202	0.07	0.00	0.13	0.042	1190	0.04	-0.02	0.11	0.175
Ratio of monounsaturated fatty acids to total fatty acids (%)	1204	-0.11	-0.17	-0.04	1.00E-03	1204	-0.11	-0.17	-0.04	1.29E-03	1192	-0.08	-0.14	-0.02	0.014
Ratio of saturated fatty acids to total fatty acids (%)	1202	0.05	-0.02	0.12	0.143	1202	0.05	-0.01	0.12	0.124	1190	0.05	-0.02	0.12	0.174
Insulin (mu/l)	1253	-0.08	-0.12	-0.04	1.69E-05	1253	-0.08	-0.12	-0.04	7.90E-05	1241	-0.04	-0.08	-0.01	0.022
Glucose (mmol/l)	1204	-0.04	-0.09	0.01	0.147	1204	-0.03	-0.09	0.02	0.228	1192	-0.02	-0.08	0.04	0.473
Lactate (mmol/l)	1205	-0.01	-0.08	0.06	0.757	1205	0.01	-0.07	0.08	0.887	1193	0.01	-0.06	0.09	0.739
Pyruvate (mmol/l)	1203	-0.04	-0.10	0.02	0.179	1203	-0.02	-0.09	0.04	0.472	1191	0.00	-0.06	0.06	0.958
Citrate (mmol/l)	1198	0.07	0.00	0.13	0.044	1198	0.05	-0.02	0.11	0.179	1186	0.03	-0.04	0.09	0.408
Alanine (mmol/l)	1207	0.00	-0.06	0.06	0.921	1207	0.03	-0.03	0.09	0.360	1195	0.04	-0.03	0.10	0.248
Glutamine (mmol/l)	1207	0.05	-0.01	0.11	0.131	1207	0.04	-0.02	0.10	0.219	1195	0.03	-0.04	0.09	0.389
Histidine (mmol/l)	1141	0.04	-0.03	0.10	0.261	1141	0.06	-0.01	0.13	0.096	1129	0.06	-0.01	0.12	0.116
Isoleucine (mmol/l)	1207	-0.05	-0.11	0.01	0.095	1207	-0.03	-0.09	0.03	0.272	1195	-0.01	-0.07	0.05	0.733
Leucine (mmol/l)	1207	0.02	-0.04	0.07	0.587	1207	0.02	-0.04	0.08	0.517	1195	0.03	-0.02	0.09	0.251
Valine (mmol/l)	1207	-0.03	-0.09	0.03	0.263	1207	-0.03	-0.09	0.04	0.424	1195	0.00	-0.06	0.06	0.993
Phenylalanine (mmol/l)	1206	0.04	-0.02	0.10	0.214	1206	0.02	-0.04	0.09	0.449	1194	0.04	-0.02	0.11	0.184
Tyrosine (mmol/l)	1200	0.01	-0.06	0.08	0.743	1200	-0.01	-0.08	0.06	0.833	1188	0.02	-0.05	0.09	0.563
Acetate (mmol/l)	1206	0.06	0.00	0.11	0.068	1206	0.06	-0.01	0.12	0.083	1194	0.05	-0.02	0.11	0.153
Acetoacetate (mmol/l)	1207	0.01	-0.06	0.07	0.821	1207	-0.01	-0.07	0.06	0.833	1195	0.00	-0.07	0.06	0.879
3-hydroxybutyrate (mmol/l)	1206	-0.04	-0.10	0.01	0.118	1206	-0.07	-0.13	-0.01	0.019	1194	-0.07	-0.12	-0.01	0.028
Creatinine (mmol/l)	1206	-0.05	-0.11	0.00	0.071	1206	-0.03	-0.09	0.03	0.406	1194	-0.01	-0.08	0.05	0.632
Albumin (signal area)	1207	-0.09	-0.15	-0.02	0.007	1207	-0.08	-0.14	-0.01	0.027	1195	-0.08	-0.14	-0.01	0.028
Glycoprotein acetyls, mainly a1-acid glycoprotein (mmol/l)	1206	-0.15	-0.21	-0.10	2.54E-08	1206	-0.16	-0.21	-0.10	6.04E-08	1194	-0.11	-0.16	-0.06	2.04E-05
C-reactive protein (mg/l)	1255	-0.02	-0.05	0.02	0.308	1255	-0.02	-0.05	0.01	0.199	1243	-0.01	-0.04	0.02	0.437

MVPA at age 15y (per SD (18 min/day) higher)

Complete case sample

Adj. for age, sex, ethnicity, maternal education, *Additionally adj. for SED at age 15y* *Additionally adj. for FMI at age 15y*

S3 Table Associations of current moderate-to-vigorous physical activity (MVPA at age 15y) with metabolic traits at age 15y in ALSPAC

MVPA at age 15y (per SD (18 min/day) higher)

Standardised outcome at age 15y | N | Beta | LCL | UCL | P-value | N | Beta | LCL | UCL | P-value | N | Beta | LCL | UCL | P-value
smoking, alcohol, wear time, wear month

	Adj. for age, sex, ethnicity, maternal education, smoking, alcohol, wear time, wear month					*Additionally adj. for SED at age 15y*					*Additionally adj. for FMI at age 15y*				
**Standardised outcome at age 15y**	**N**	**Beta**	**LCL**	**UCL**	**P-value**	**N**	**Beta**	**LCL**	**UCL**	**P-value**	**N**	**Beta**	**LCL**	**UCL**	**P-value**
Systolic blood pressure (mmHg)	755	0.00	-0.07	0.08	0.926	755	0.01	-0.06	0.09	0.709	755	0.02	-0.05	0.10	0.540
Diastolic blood pressure (mmHg)	755	0.03	-0.04	0.10	0.404	755	0.04	-0.04	0.12	0.306	755	0.04	-0.03	0.12	0.282
Concentration of chylomicrons and extremely large VLDL particles (mol/l)	755	-0.12	-0.19	-0.05	5.17E-04	755	-0.12	-0.19	-0.05	7.31E-04	755	-0.11	-0.17	-0.04	1.70E-03
Total lipids in chylomicrons and extremely large VLDL (mmol/l)	755	-0.12	-0.19	-0.05	5.33E-04	755	-0.12	-0.19	-0.05	8.17E-04	755	-0.11	-0.17	-0.04	1.79E-03
Phospholipids in chylomicrons and extremely large VLDL (mmol/l)	755	-0.13	-0.19	-0.06	3.80E-04	755	-0.12	-0.19	-0.05	5.28E-04	755	-0.11	-0.18	-0.04	1.18E-03
Total cholesterol in chylomicrons and extremely large VLDL (mmol/l)	755	-0.10	-0.17	-0.03	0.003	755	-0.10	-0.17	-0.03	0.005	755	-0.09	-0.15	-0.02	0.010
Cholesterol esters in chylomicrons and extremely large VLDL (mmol/l)	755	-0.08	-0.15	-0.01	0.021	755	-0.07	-0.14	-0.01	0.033	755	-0.06	-0.13	0.00	0.065
Free cholesterol in chylomicrons and extremely large VLDL (mmol/l)	755	-0.12	-0.19	-0.05	5.12E-04	755	-0.12	-0.19	-0.05	6.92E-04	755	-0.11	-0.18	-0.04	1.52E-03
Triglycerides in chylomicrons and extremely large VLDL (mmol/l)	755	-0.13	-0.20	-0.06	3.89E-04	755	-0.12	-0.19	-0.05	6.02E-04	755	-0.11	-0.18	-0.04	1.32E-03
Concentration of very large VLDL particles (mol/l)	755	-0.12	-0.19	-0.05	8.18E-04	755	-0.11	-0.18	-0.04	1.62E-03	755	-0.10	-0.17	-0.03	0.004
Total lipids in very large VLDL (mmol/l)	755	-0.12	-0.19	-0.05	9.69E-04	755	-0.11	-0.18	-0.04	1.90E-03	755	-0.10	-0.17	-0.03	0.004
Phospholipids in very large VLDL (mmol/l)	755	-0.12	-0.18	-0.05	8.64E-04	755	-0.11	-0.18	-0.04	1.42E-03	755	-0.10	-0.17	-0.03	0.003
Total cholesterol in very large VLDL (mmol/l)	755	-0.11	-0.18	-0.04	1.49E-03	755	-0.11	-0.18	-0.04	2.47E-03	755	-0.10	-0.16	-0.03	0.005
Cholesterol esters in very large VLDL (mmol/l)	755	-0.11	-0.18	-0.04	2.58E-03	755	-0.10	-0.17	-0.03	0.005	755	-0.09	-0.16	-0.02	0.010
Free cholesterol in very large VLDL (mmol/l)	755	-0.12	-0.19	-0.05	9.02E-04	755	-0.11	-0.18	-0.05	1.28E-03	755	-0.10	-0.17	-0.04	2.83E-03
Triglycerides in very large VLDL (mmol/l)	755	-0.12	-0.19	-0.05	9.38E-04	755	-0.11	-0.18	-0.04	2.01E-03	755	-0.10	-0.17	-0.03	0.004
Concentration of large VLDL particles (mol/l)	755	-0.11	-0.18	-0.04	1.49E-03	755	-0.11	-0.18	-0.04	0.003	755	-0.09	-0.16	-0.03	0.007
Total lipids in large VLDL (mmol/l)	755	-0.11	-0.18	-0.04	1.82E-03	755	-0.11	-0.18	-0.03	0.004	755	-0.09	-0.16	-0.02	0.008
Phospholipids in large VLDL (mmol/l)	755	-0.11	-0.18	-0.04	1.69E-03	755	-0.11	-0.18	-0.04	0.004	755	-0.09	-0.16	-0.03	0.008
Total cholesterol in large VLDL (mmol/l)	755	-0.10	-0.17	-0.03	0.004	755	-0.10	-0.17	-0.03	0.008	755	-0.08	-0.15	-0.02	0.017
Cholesterol esters in large VLDL (mmol/l)	755	-0.09	-0.16	-0.02	0.011	755	-0.09	-0.16	-0.01	0.020	755	-0.07	-0.14	0.00	0.040
Free cholesterol in large VLDL (mmol/l)	755	-0.11	-0.18	-0.04	1.66E-03	755	-0.11	-0.18	-0.03	0.004	755	-0.09	-0.16	-0.03	0.007
Triglycerides in large VLDL (mmol/l)	755	-0.11	-0.19	-0.04	1.42E-03	755	-0.11	-0.18	-0.04	0.003	755	-0.10	-0.16	-0.03	0.007
Concentration of medium VLDL particles (mol/l)	755	-0.11	-0.19	-0.04	1.90E-03	755	-0.11	-0.18	-0.03	0.005	755	-0.09	-0.17	-0.02	0.010
Total lipids in medium VLDL (mmol/l)	755	-0.11	-0.18	-0.04	0.003	755	-0.10	-0.18	-0.03	0.007	755	-0.09	-0.16	-0.02	0.014
Phospholipids in medium VLDL (mmol/l)	755	-0.11	-0.18	-0.04	0.003	755	-0.10	-0.18	-0.03	0.006	755	-0.09	-0.16	-0.02	0.014
Total cholesterol in medium VLDL (mmol/l)	755	-0.09	-0.16	-0.01	0.021	755	-0.08	-0.16	-0.01	0.036	755	-0.07	-0.14	0.01	0.070
Cholesterol esters in medium VLDL (mmol/l)	755	-0.06	-0.14	0.01	0.093	755	-0.06	-0.13	0.02	0.137	755	-0.04	-0.12	0.03	0.237
Free cholesterol in medium VLDL (mmol/l)	755	-0.11	-0.18	-0.04	0.003	755	-0.10	-0.18	-0.03	0.007	755	-0.09	-0.16	-0.02	0.014
Triglycerides in medium VLDL (mmol/l)	755	-0.12	-0.19	-0.05	1.46E-03	755	-0.11	-0.18	-0.04	0.004	755	-0.10	-0.17	-0.03	0.008
Concentration of small VLDL particles (mol/l)	755	-0.10	-0.18	-0.03	0.006	755	-0.10	-0.18	-0.03	0.009	755	-0.09	-0.16	-0.01	0.019
Total lipids in small VLDL (mmol/l)	755	-0.10	-0.18	-0.03	0.008	755	-0.10	-0.18	-0.02	0.011	755	-0.09	-0.16	-0.01	0.023
Phospholipids in small VLDL (mmol/l)	755	-0.09	-0.16	-0.01	0.019	755	-0.09	-0.17	-0.01	0.022	755	-0.08	-0.15	0.00	0.046
Total cholesterol in small VLDL (mmol/l)	755	-0.09	-0.16	-0.01	0.031	755	-0.09	-0.17	-0.01	0.033	755	-0.08	-0.16	0.00	0.066
Cholesterol esters in small VLDL (mmol/l)	755	-0.08	-0.16	0.00	0.044	755	-0.09	-0.17	0.00	0.042	755	-0.07	-0.15	0.01	0.081
Free cholesterol in small VLDL (mmol/l)	755	-0.09	-0.16	-0.01	0.024	755	-0.09	-0.16	-0.01	0.031	755	-0.07	-0.15	0.00	0.060
Triglycerides in small VLDL (mmol/l)	755	-0.11	-0.18	-0.04	0.004	755	-0.10	-0.18	-0.03	0.006	755	-0.09	-0.17	-0.02	0.013
Concentration of very small VLDL particles (mol/l)	755	-0.04	-0.11	0.04	0.352	755	-0.04	-0.12	0.04	0.327	755	-0.03	-0.11	0.05	0.423
Total lipids in very small VLDL (mmol/l)	755	-0.05	-0.13	0.02	0.175	755	-0.06	-0.14	0.02	0.168	755	-0.05	-0.13	0.03	0.245
Phospholipids in very small VLDL (mmol/l)	755	-0.03	-0.10	0.05	0.446	755	-0.04	-0.11	0.04	0.340	755	-0.03	-0.11	0.04	0.401
Total cholesterol in very small VLDL (mmol/l)	755	-0.04	-0.12	0.04	0.294	755	-0.04	-0.12	0.04	0.328	755	-0.03	-0.11	0.05	0.457
Cholesterol esters in very small VLDL (mmol/l)	755	-0.06	-0.14	0.02	0.142	755	-0.06	-0.14	0.02	0.156	755	-0.05	-0.13	0.03	0.247
Free cholesterol in very small VLDL (mmol/l)	755	0.00	-0.08	0.07	0.974	755	0.00	-0.07	0.08	0.928	755	0.01	-0.07	0.09	0.825
Triglycerides in very small VLDL (mmol/l)	755	-0.08	-0.15	-0.01	0.022	755	-0.09	-0.16	-0.01	0.019	755	-0.08	-0.15	-0.01	0.033
Concentration of IDL particles (mol/l)	755	-0.02	-0.10	0.05	0.526	755	-0.04	-0.11	0.04	0.364	755	-0.03	-0.11	0.04	0.383

S3 Table Associations of current moderate-to-vigorous physical activity (MVPA at age 15y) with metabolic traits at age 15y in ALSPAC

	**MVPA at age 15y (per SD (18 min/day) higher)**														
	Adj. for age, sex, ethnicity, maternal education, smoking, alcohol, wear time, wear month					*Additionally adj. for SED at age 15y*					*Additionally adj. for FMI at age 15y*				
**Standardised outcome at age 15y**	**N**	**Beta**	**LCL**	**UCL**	**P-value**	**N**	**Beta**	**LCL**	**UCL**	**P-value**	**N**	**Beta**	**LCL**	**UCL**	**P-value**
Total lipids in IDL (mmol/l)	755	-0.02	-0.10	0.05	0.581	755	-0.03	-0.11	0.05	0.432	755	-0.03	-0.11	0.05	0.473
Phospholipids in IDL (mmol/l)	755	-0.02	-0.09	0.06	0.687	755	-0.03	-0.11	0.05	0.446	755	-0.03	-0.11	0.05	0.460
Total cholesterol in IDL (mmol/l)	755	-0.02	-0.10	0.06	0.590	755	-0.03	-0.11	0.05	0.484	755	-0.02	-0.10	0.05	0.545
Cholesterol esters in IDL (mmol/l)	755	-0.03	-0.11	0.05	0.472	755	-0.03	-0.11	0.05	0.409	755	-0.03	-0.11	0.05	0.487
Free cholesterol in IDL (mmol/l)	755	0.00	-0.08	0.07	0.935	755	-0.01	-0.09	0.06	0.713	755	-0.01	-0.09	0.06	0.714
Triglycerides in IDL (mmol/l)	755	-0.03	-0.10	0.04	0.442	755	-0.04	-0.11	0.03	0.271	755	-0.04	-0.11	0.03	0.248
Concentration of large LDL particles (mol/l)	755	-0.02	-0.10	0.05	0.520	755	-0.04	-0.12	0.04	0.292	755	-0.04	-0.12	0.04	0.318
Total lipids in large LDL (mmol/l)	755	-0.02	-0.09	0.05	0.585	755	-0.04	-0.11	0.04	0.367	755	-0.03	-0.11	0.04	0.401
Phospholipids in large LDL (mmol/l)	755	-0.02	-0.10	0.05	0.539	755	-0.04	-0.11	0.04	0.327	755	-0.04	-0.11	0.04	0.373
Total cholesterol in large LDL (mmol/l)	755	-0.02	-0.09	0.06	0.618	755	-0.03	-0.11	0.04	0.409	755	-0.03	-0.11	0.05	0.451
Cholesterol esters in large LDL (mmol/l)	755	-0.02	-0.10	0.05	0.549	755	-0.04	-0.11	0.04	0.359	755	-0.03	-0.11	0.04	0.407
Free cholesterol in large LDL (mmol/l)	755	-0.01	-0.08	0.07	0.850	755	-0.02	-0.10	0.06	0.589	755	-0.02	-0.10	0.06	0.605
Triglycerides in large LDL (mmol/l)	755	-0.02	-0.09	0.05	0.556	755	-0.04	-0.11	0.03	0.287	755	-0.04	-0.11	0.03	0.235
Concentration of medium LDL particles (mol/l)	755	-0.04	-0.11	0.04	0.333	755	-0.05	-0.13	0.02	0.157	755	-0.05	-0.13	0.02	0.184
Total lipids in medium LDL (mmol/l)	755	-0.03	-0.10	0.04	0.430	755	-0.05	-0.12	0.03	0.240	755	-0.04	-0.12	0.03	0.275
Phospholipids in medium LDL (mmol/l)	755	-0.03	-0.11	0.04	0.374	755	-0.05	-0.12	0.03	0.233	755	-0.04	-0.12	0.04	0.292
Total cholesterol in medium LDL (mmol/l)	755	-0.03	-0.10	0.05	0.447	755	-0.04	-0.12	0.03	0.263	755	-0.04	-0.12	0.04	0.301
Cholesterol esters in medium LDL (mmol/l)	755	-0.03	-0.11	0.04	0.409	755	-0.05	-0.12	0.03	0.229	755	-0.04	-0.12	0.03	0.265
Free cholesterol in medium LDL (mmol/l)	755	-0.02	-0.09	0.06	0.634	755	-0.03	-0.11	0.05	0.452	755	-0.03	-0.11	0.05	0.499
Triglycerides in medium LDL (mmol/l)	755	-0.02	-0.09	0.05	0.622	755	-0.04	-0.11	0.03	0.254	755	-0.04	-0.11	0.02	0.208
Concentration of small LDL particles (mol/l)	755	-0.04	-0.11	0.03	0.300	755	-0.06	-0.13	0.02	0.150	755	-0.05	-0.13	0.02	0.175
Total lipids in small LDL (mmol/l)	755	-0.03	-0.11	0.04	0.388	755	-0.05	-0.12	0.03	0.218	755	-0.04	-0.12	0.03	0.254
Phospholipids in small LDL (mmol/l)	755	-0.03	-0.11	0.04	0.389	755	-0.04	-0.12	0.03	0.256	755	-0.04	-0.12	0.04	0.303
Total cholesterol in small LDL (mmol/l)	755	-0.03	-0.10	0.05	0.453	755	-0.04	-0.12	0.03	0.263	755	-0.04	-0.12	0.04	0.301
Cholesterol esters in small LDL (mmol/l)	755	-0.03	-0.11	0.04	0.421	755	-0.05	-0.12	0.03	0.225	755	-0.04	-0.12	0.03	0.258
Free cholesterol in small LDL (mmol/l)	755	-0.02	-0.09	0.06	0.655	755	-0.02	-0.10	0.05	0.540	755	-0.02	-0.10	0.06	0.609
Triglycerides in small LDL (mmol/l)	755	-0.05	-0.12	0.01	0.123	755	-0.07	-0.14	0.00	0.039	755	-0.07	-0.14	0.00	0.045
Concentration of very large HDL particles (mol/l)	755	0.09	0.01	0.18	0.026	755	0.09	0.00	0.18	0.042	755	0.08	-0.01	0.16	0.075
Total lipids in very large HDL (mmol/l)	755	0.09	0.01	0.17	0.035	755	0.09	0.00	0.17	0.053	755	0.07	-0.01	0.16	0.093
Phospholipids in very large HDL (mmol/l)	755	0.10	0.02	0.18	0.016	755	0.10	0.01	0.18	0.028	755	0.08	0.00	0.16	0.052
Total cholesterol in very large HDL (mmol/l)	755	0.07	-0.01	0.15	0.085	755	0.07	-0.02	0.16	0.109	755	0.06	-0.03	0.14	0.174
Cholesterol esters in very large HDL (mmol/l)	755	0.06	-0.02	0.15	0.119	755	0.06	-0.02	0.15	0.146	755	0.05	-0.03	0.14	0.223
Free cholesterol in very large HDL (mmol/l)	755	0.09	0.00	0.17	0.038	755	0.08	0.00	0.17	0.056	755	0.07	-0.01	0.15	0.097
Triglycerides in very large HDL (mmol/l)	755	0.00	-0.07	0.08	0.898	755	0.01	-0.06	0.09	0.722	755	0.01	-0.07	0.08	0.853
Concentration of large HDL particles (mol/l)	755	0.11	0.03	0.20	0.007	755	0.11	0.02	0.19	0.013	755	0.09	0.01	0.18	0.026
Total lipids in large HDL (mmol/l)	755	0.11	0.03	0.20	0.007	755	0.11	0.02	0.19	0.013	755	0.09	0.01	0.18	0.025
Phospholipids in large HDL (mmol/l)	755	0.11	0.02	0.19	0.011	755	0.10	0.01	0.18	0.023	755	0.08	0.00	0.16	0.043
Total cholesterol in large HDL (mmol/l)	755	0.12	0.04	0.20	0.005	755	0.12	0.03	0.20	0.008	755	0.10	0.02	0.18	0.015
Cholesterol esters in large HDL (mmol/l)	755	0.12	0.04	0.20	0.004	755	0.12	0.03	0.21	0.007	755	0.10	0.02	0.19	0.014
Free cholesterol in large HDL (mmol/l)	755	0.11	0.03	0.20	0.006	755	0.11	0.03	0.20	0.010	755	0.10	0.02	0.18	0.020
Triglycerides in large HDL (mmol/l)	755	0.04	-0.03	0.11	0.302	755	0.04	-0.03	0.12	0.266	755	0.04	-0.04	0.11	0.332
Concentration of medium HDL particles (mol/l)	755	0.06	-0.01	0.14	0.084	755	0.05	-0.02	0.13	0.166	755	0.05	-0.02	0.12	0.191
Total lipids in medium HDL (mmol/l)	755	0.07	-0.01	0.14	0.075	755	0.06	-0.02	0.13	0.148	755	0.05	-0.02	0.13	0.181
Phospholipids in medium HDL (mmol/l)	755	0.07	0.00	0.14	0.058	755	0.06	-0.02	0.13	0.133	755	0.05	-0.02	0.13	0.165
Total cholesterol in medium HDL (mmol/l)	755	0.07	0.00	0.15	0.064	755	0.07	-0.01	0.14	0.104	755	0.06	-0.02	0.14	0.136
Cholesterol esters in medium HDL (mmol/l)	755	0.07	0.00	0.15	0.057	755	0.07	-0.01	0.15	0.090	755	0.06	-0.02	0.14	0.120
Free cholesterol in medium HDL (mmol/l)	755	0.06	-0.02	0.13	0.123	755	0.05	-0.03	0.13	0.211	755	0.04	-0.03	0.12	0.256
Triglycerides in medium HDL (mmol/l)	755	-0.06	-0.13	0.01	0.112	755	-0.07	-0.14	0.01	0.068	755	-0.06	-0.13	0.01	0.118

S3 Table Associations of current moderate-to-vigorous physical activity (MVPA at age 15y) with metabolic traits at age 15y in ALSPAC

MVPA at age 15y (per SD (18 min/day) higher)

	Adj. for age, sex, ethnicity, maternal education, smoking, alcohol, wear time, wear month					*Additionally adj. for SED at age 15y*					*Additionally adj. for FMI at age 15y*				
**Standardised outcome at age 15y**	**N**	**Beta**	**LCL**	**UCL**	**P-value**	**N**	**Beta**	**LCL**	**UCL**	**P-value**	**N**	**Beta**	**LCL**	**UCL**	**P-value**
Concentration of small HDL particles (mol/l)	755	-0.03	-0.10	0.04	0.429	755	-0.05	-0.12	0.03	0.204	755	-0.04	-0.11	0.03	0.274
Total lipids in small HDL (mmol/l)	755	0.01	-0.06	0.08	0.725	755	-0.01	-0.08	0.06	0.783	755	-0.01	-0.08	0.06	0.852
Phospholipids in small HDL (mmol/l)	755	-0.04	-0.11	0.04	0.327	755	-0.05	-0.12	0.03	0.211	755	-0.04	-0.12	0.03	0.264
Total cholesterol in small HDL (mmol/l)	755	0.07	0.01	0.14	0.033	755	0.05	-0.02	0.12	0.168	755	0.05	-0.02	0.12	0.177
Cholesterol esters in small HDL (mmol/l)	755	0.08	0.01	0.14	0.026	755	0.05	-0.02	0.12	0.156	755	0.05	-0.02	0.12	0.162
Free cholesterol in small HDL (mmol/l)	755	0.03	-0.04	0.10	0.358	755	0.02	-0.05	0.10	0.520	755	0.02	-0.05	0.10	0.557
Triglycerides in small HDL (mmol/l)	755	-0.09	-0.16	-0.02	0.008	755	-0.10	-0.17	-0.03	0.004	755	-0.09	-0.16	-0.03	0.007
Phospholipids to total lipds ratio in chylomicrons and extremely large VLDL (%)	755	-0.08	-0.15	0.00	0.048	755	-0.08	-0.16	0.00	0.040	755	-0.08	-0.15	0.00	0.044
Total cholesterol to total lipids ratio in chylomicrons and extremely large VLDL (%)	755	0.02	-0.06	0.09	0.650	755	0.00	-0.08	0.08	0.953	755	0.01	-0.07	0.09	0.797
Cholesterol esters to total lipids ratio in chylomicrons and extremely large VLDL (%)	755	0.05	-0.03	0.12	0.235	755	0.03	-0.05	0.11	0.433	755	0.04	-0.04	0.11	0.328
Free cholesterol to total lipids ratio in chylomicrons and extremely large VLDL (%)	755	-0.07	-0.15	0.01	0.074	755	-0.08	-0.16	0.00	0.044	755	-0.07	-0.15	0.00	0.062
Triglycerides to total lipids ratio in chylomicrons and extremely large VLDL (%)	755	-0.01	-0.06	0.05	0.728	755	0.00	-0.05	0.06	0.931	755	0.00	-0.06	0.05	0.906
Phospholipids to total lipds ratio in very large VLDL (%)	755	-0.07	-0.15	0.01	0.075	755	-0.09	-0.17	-0.01	0.035	755	-0.08	-0.16	0.00	0.052
Total cholesterol to total lipids ratio in very large VLDL (%)	755	0.13	-0.01	0.27	0.068	755	0.11	-0.02	0.24	0.090	755	0.11	-0.02	0.24	0.103
Cholesterol esters to total lipids ratio in very large VLDL (%)	755	0.09	0.01	0.17	0.031	755	0.08	0.00	0.17	0.055	755	0.08	-0.01	0.17	0.066
Free cholesterol to total lipids ratio in very large VLDL (%)	755	0.06	-0.02	0.14	0.123	755	0.05	-0.04	0.13	0.286	755	0.04	-0.04	0.13	0.319
Triglycerides to total lipids ratio in very large VLDL (%)	755	-0.07	-0.15	0.02	0.115	755	-0.05	-0.13	0.04	0.278	755	-0.05	-0.13	0.04	0.291
Phospholipids to total lipds ratio in large VLDL (%)	755	-0.07	-0.16	0.01	0.101	755	-0.08	-0.17	0.01	0.084	755	-0.07	-0.16	0.02	0.112
Total cholesterol to total lipids ratio in large VLDL (%)	755	0.00	-0.08	0.08	0.981	755	-0.01	-0.10	0.07	0.779	755	-0.01	-0.09	0.08	0.903
Cholesterol esters to total lipids ratio in large VLDL (%)	755	0.13	-0.09	0.34	0.246	755	0.11	-0.08	0.30	0.259	755	0.11	-0.08	0.31	0.255
Free cholesterol to total lipids ratio in large VLDL (%)	755	-0.08	-0.16	0.00	0.044	755	-0.09	-0.17	0.00	0.042	755	-0.08	-0.16	0.00	0.064
Triglycerides to total lipids ratio in large VLDL (%)	755	0.13	-0.11	0.37	0.291	755	0.11	-0.10	0.33	0.286	755	0.12	-0.10	0.34	0.290
Phospholipids to total lipds ratio in medium VLDL (%)	755	0.07	-0.02	0.15	0.123	755	0.06	-0.03	0.15	0.190	755	0.05	-0.04	0.14	0.266
Total cholesterol to total lipids ratio in medium VLDL (%)	755	0.03	-0.04	0.11	0.409	755	0.03	-0.05	0.10	0.472	755	0.03	-0.05	0.11	0.441
Cholesterol esters to total lipids ratio in medium VLDL (%)	755	0.05	-0.03	0.12	0.198	755	0.05	-0.03	0.12	0.226	755	0.05	-0.03	0.13	0.212
Free cholesterol to total lipids ratio in medium VLDL (%)	755	-0.04	-0.12	0.03	0.291	755	-0.05	-0.12	0.03	0.226	755	-0.04	-0.12	0.03	0.251
Triglycerides to total lipids ratio in medium VLDL (%)	755	-0.04	-0.12	0.03	0.243	755	-0.04	-0.12	0.04	0.312	755	-0.04	-0.12	0.04	0.317
Phospholipids to total lipds ratio in small VLDL (%)	755	0.12	0.05	0.20	9.21E-04	755	0.12	0.04	0.20	2.09E-03	755	0.11	0.04	0.19	0.004
Total cholesterol to total lipids ratio in small VLDL (%)	755	0.02	-0.06	0.09	0.666	755	0.01	-0.07	0.09	0.763	755	0.01	-0.07	0.09	0.759
Cholesterol esters to total lipids ratio in small VLDL (%)	755	0.00	-0.08	0.08	0.994	755	-0.01	-0.08	0.07	0.880	755	0.00	-0.08	0.07	0.917
Free cholesterol to total lipids ratio in small VLDL (%)	755	0.12	0.04	0.20	2.22E-03	755	0.12	0.04	0.20	0.003	755	0.11	0.03	0.19	0.005
Triglycerides to total lipids ratio in small VLDL (%)	755	-0.05	-0.13	0.02	0.176	755	-0.05	-0.13	0.03	0.238	755	-0.05	-0.12	0.03	0.262
Phospholipids to total lipds ratio in very small VLDL (%)	755	0.01	-0.06	0.09	0.715	755	0.00	-0.08	0.08	0.998	755	-0.01	-0.08	0.07	0.881
Total cholesterol to total lipids ratio in very small VLDL (%)	755	0.03	-0.05	0.10	0.480	755	0.04	-0.03	0.11	0.299	755	0.04	-0.03	0.12	0.261
Cholesterol esters to total lipids ratio in very small VLDL (%)	755	-0.02	-0.09	0.05	0.552	755	-0.02	-0.09	0.06	0.670	755	-0.01	-0.08	0.06	0.790
Free cholesterol to total lipids ratio in very small VLDL (%)	755	0.11	0.04	0.18	2.59E-03	755	0.13	0.06	0.20	4.74E-04	755	0.13	0.05	0.20	8.81E-04
Triglycerides to total lipids ratio in very small VLDL (%)	755	-0.04	-0.11	0.03	0.273	755	-0.04	-0.12	0.03	0.224	755	-0.04	-0.12	0.03	0.230
Phospholipids to total lipds ratio in IDL (%)	755	0.06	-0.02	0.13	0.151	755	0.03	-0.05	0.11	0.471	755	0.02	-0.06	0.10	0.637
Total cholesterol to total lipids ratio in IDL (%)	755	-0.03	-0.11	0.04	0.403	755	-0.02	-0.10	0.06	0.681	755	-0.01	-0.09	0.07	0.830
Cholesterol esters to total lipids ratio in IDL (%)	755	-0.06	-0.14	0.02	0.148	755	-0.04	-0.12	0.04	0.327	755	-0.03	-0.11	0.05	0.476
Free cholesterol to total lipids ratio in IDL (%)	755	0.06	-0.01	0.13	0.111	755	0.05	-0.02	0.13	0.138	755	0.05	-0.02	0.12	0.204
Triglycerides to total lipids ratio in IDL (%)	755	0.01	-0.06	0.09	0.711	755	0.01	-0.07	0.08	0.861	755	0.00	-0.07	0.08	0.963
Phospholipids to total lipds ratio in large LDL (%)	755	0.03	-0.04	0.10	0.467	755	0.03	-0.04	0.10	0.451	755	0.03	-0.04	0.10	0.427
Total cholesterol to total lipids ratio in large LDL (%)	755	-0.03	-0.11	0.04	0.375	755	-0.03	-0.11	0.05	0.437	755	-0.03	-0.10	0.05	0.495
Cholesterol esters to total lipids ratio in large LDL (%)	755	-0.05	-0.13	0.02	0.183	755	-0.05	-0.13	0.03	0.195	755	-0.05	-0.13	0.03	0.244
Free cholesterol to total lipids ratio in large LDL (%)	755	0.07	0.00	0.13	0.043	755	0.08	0.01	0.14	0.024	755	0.07	0.00	0.14	0.037
Triglycerides to total lipids ratio in large LDL (%)	755	0.03	-0.05	0.10	0.462	755	0.02	-0.06	0.10	0.600	755	0.01	-0.06	0.09	0.742
Phospholipids to total lipds ratio in medium LDL (%)	755	0.01	-0.01	0.04	0.351	755	0.02	-0.01	0.04	0.280	755	0.02	-0.01	0.04	0.265

S3 Table Associations of current moderate-to-vigorous physical activity (MVPA at age 15y) with metabolic traits at age 15y in ALSPAC

MVPA at age 15y (per SD (18 min/day) higher)

	Adj. for age, sex, ethnicity, maternal education, smoking, alcohol, wear time, wear month					*Additionally adj. for SED at age 15y*					*Additionally adj. for FMI at age 15y*				
**Standardised outcome at age 15y**	**N**	**Beta**	**LCL**	**UCL**	**P-value**	**N**	**Beta**	**LCL**	**UCL**	**P-value**	**N**	**Beta**	**LCL**	**UCL**	**P-value**
Total cholesterol to total lipids ratio in medium LDL (%)	755	-0.05	-0.13	0.03	0.209	755	-0.05	-0.13	0.03	0.241	755	-0.05	-0.13	0.04	0.264
Cholesterol esters to total lipids ratio in medium LDL (%)	755	-0.06	-0.14	0.03	0.174	755	-0.06	-0.14	0.02	0.153	755	-0.06	-0.14	0.03	0.171
Free cholesterol to total lipids ratio in medium LDL (%)	755	0.01	-0.01	0.04	0.180	755	0.02	0.00	0.04	0.105	755	0.02	0.00	0.04	0.117
Triglycerides to total lipids ratio in medium LDL (%)	755	0.04	-0.03	0.12	0.290	755	0.02	-0.06	0.10	0.584	755	0.01	-0.06	0.09	0.732
Phospholipids to total lipds ratio in small LDL (%)	755	0.03	-0.02	0.07	0.246	755	0.03	-0.01	0.08	0.183	755	0.03	-0.02	0.08	0.195
Total cholesterol to total lipids ratio in small LDL (%)	755	-0.04	-0.11	0.04	0.374	755	-0.04	-0.12	0.04	0.361	755	-0.04	-0.11	0.04	0.382
Cholesterol esters to total lipids ratio in small LDL (%)	755	-0.04	-0.12	0.04	0.282	755	-0.05	-0.13	0.03	0.218	755	-0.05	-0.13	0.03	0.233
Free cholesterol to total lipids ratio in small LDL (%)	755	0.03	-0.02	0.07	0.217	755	0.03	-0.01	0.08	0.111	755	0.03	-0.01	0.08	0.119
Triglycerides to total lipids ratio in small LDL (%)	755	-0.02	-0.09	0.05	0.604	755	-0.04	-0.11	0.04	0.327	755	-0.04	-0.11	0.03	0.318
Phospholipids to total lipds ratio in very large HDL (%)	755	0.10	0.02	0.18	0.018	755	0.09	0.01	0.18	0.033	755	0.08	0.00	0.16	0.058
Total cholesterol to total lipids ratio in very large HDL (%)	755	-0.09	-0.17	-0.01	0.029	755	-0.09	-0.17	0.00	0.046	755	-0.07	-0.15	0.01	0.079
Cholesterol esters to total lipids ratio in very large HDL (%)	755	-0.09	-0.17	-0.01	0.032	755	-0.09	-0.17	0.00	0.048	755	-0.07	-0.15	0.01	0.082
Free cholesterol to total lipids ratio in very large HDL (%)	755	0.03	-0.04	0.10	0.455	755	0.04	-0.04	0.11	0.342	755	0.03	-0.04	0.11	0.431
Triglycerides to total lipids ratio in very large HDL (%)	755	-0.07	-0.15	0.00	0.062	755	-0.06	-0.14	0.01	0.100	755	-0.06	-0.13	0.02	0.143
Phospholipids to total lipds ratio in large HDL (%)	755	-0.09	-0.17	-0.02	0.013	755	-0.11	-0.19	-0.04	0.003	755	-0.10	-0.17	-0.03	0.008
Total cholesterol to total lipids ratio in large HDL (%)	755	0.10	0.03	0.18	0.009	755	0.12	0.04	0.20	0.004	755	0.10	0.02	0.18	0.011
Cholesterol esters to total lipids ratio in large HDL (%)	755	0.11	0.03	0.19	0.010	755	0.12	0.04	0.20	0.005	755	0.10	0.02	0.18	0.012
Free cholesterol to total lipids ratio in large HDL (%)	755	0.07	0.00	0.15	0.048	755	0.09	0.01	0.16	0.022	755	0.07	0.00	0.14	0.042
Triglycerides to total lipids ratio in large HDL (%)	755	-0.09	-0.17	-0.01	0.025	755	-0.09	-0.17	0.00	0.041	755	-0.07	-0.15	0.01	0.079
Phospholipids to total lipds ratio in medium HDL (%)	755	0.06	-0.01	0.13	0.082	755	0.04	-0.03	0.12	0.235	755	0.04	-0.03	0.11	0.281
Total cholesterol to total lipids ratio in medium HDL (%)	755	0.00	-0.07	0.07	0.992	755	0.02	-0.05	0.09	0.563	755	0.02	-0.05	0.09	0.658
Cholesterol esters to total lipids ratio in medium HDL (%)	755	0.01	-0.06	0.08	0.853	755	0.03	-0.04	0.10	0.451	755	0.02	-0.05	0.09	0.537
Free cholesterol to total lipids ratio in medium HDL (%)	755	-0.03	-0.10	0.05	0.535	755	-0.02	-0.11	0.07	0.633	755	-0.02	-0.11	0.07	0.616
Triglycerides to total lipids ratio in medium HDL (%)	755	-0.09	-0.17	-0.01	0.027	755	-0.10	-0.18	-0.02	0.019	755	-0.08	-0.16	0.00	0.038
Phospholipids to total lipds ratio in small HDL (%)	755	-0.09	-0.16	-0.02	0.008	755	-0.07	-0.14	0.00	0.043	755	-0.07	-0.14	0.00	0.052
Total cholesterol to total lipids ratio in small HDL (%)	755	0.11	0.04	0.18	1.43E-03	755	0.09	0.02	0.16	0.010	755	0.09	0.02	0.16	0.013
Cholesterol esters to total lipids ratio in small HDL (%)	755	0.10	0.03	0.17	0.005	755	0.08	0.01	0.15	0.032	755	0.07	0.00	0.14	0.037
Free cholesterol to total lipids ratio in small HDL (%)	755	0.05	-0.02	0.13	0.164	755	0.08	0.00	0.16	0.038	755	0.07	-0.01	0.15	0.074
Triglycerides to total lipids ratio in small HDL (%)	755	-0.11	-0.18	-0.04	2.46E-03	755	-0.11	-0.19	-0.04	2.51E-03	755	-0.10	-0.18	-0.03	0.005
Mean diameter for VLDL particles (nm)	755	-0.11	-0.19	-0.04	0.004	755	-0.11	-0.19	-0.03	0.007	755	-0.10	-0.17	-0.02	0.012
Mean diameter for LDL particles (nm)	755	0.05	-0.01	0.12	0.127	755	0.06	-0.01	0.13	0.074	755	0.06	-0.01	0.13	0.101
Mean diameter for HDL particles (nm)	755	0.11	0.02	0.20	0.012	755	0.11	0.02	0.20	0.015	755	0.10	0.01	0.18	0.029
Serum total cholesterol (mmol/l)	755	0.00	-0.08	0.07	0.964	755	-0.01	-0.09	0.06	0.746	755	-0.01	-0.09	0.07	0.771
Total cholesterol in VLDL (mmol/l)	755	-0.09	-0.17	-0.02	0.018	755	-0.09	-0.17	-0.01	0.027	755	-0.07	-0.15	0.00	0.057
Remnant cholesterol (non-HDL, non-LDL -cholesterol) (mmol/l)	755	-0.07	-0.14	0.01	0.092	755	-0.07	-0.15	0.01	0.096	755	-0.06	-0.14	0.02	0.156
Total cholesterol in LDL (mmol/l)	755	-0.02	-0.10	0.05	0.530	755	-0.04	-0.12	0.04	0.330	755	-0.04	-0.11	0.04	0.371
Total cholesterol in HDL (mmol/l)	755	0.11	0.03	0.19	0.009	755	0.10	0.02	0.18	0.021	755	0.09	0.01	0.17	0.036
Total cholesterol in HDL2 (mmol/l)	755	0.11	0.03	0.19	0.007	755	0.10	0.02	0.19	0.017	755	0.09	0.01	0.17	0.031
Total cholesterol in HDL3 (mmol/l)	755	0.09	0.02	0.17	0.019	755	0.09	0.01	0.17	0.035	755	0.08	0.00	0.15	0.057
Esterified cholesterol (mmol/l)	755	0.00	-0.07	0.07	0.980	755	-0.01	-0.09	0.06	0.757	755	-0.01	-0.09	0.07	0.776
Free cholesterol (mmol/l)	755	0.00	-0.08	0.07	0.930	755	-0.01	-0.09	0.06	0.731	755	-0.01	-0.09	0.07	0.768
Serum total triglycerides (mmol/l)	755	-0.11	-0.18	-0.04	2.25E-03	755	-0.11	-0.18	-0.04	0.003	755	-0.10	-0.17	-0.03	0.006
Triglycerides in VLDL (mmol/l)	755	-0.12	-0.19	-0.04	1.43E-03	755	-0.11	-0.18	-0.04	0.003	755	-0.10	-0.17	-0.03	0.007
Triglycerides in LDL (mmol/l)	755	-0.03	-0.09	0.04	0.459	755	-0.05	-0.11	0.02	0.198	755	-0.05	-0.12	0.02	0.168
Triglycerides in HDL (mmol/l)	755	-0.05	-0.12	0.02	0.129	755	-0.06	-0.12	0.01	0.099	755	-0.05	-0.12	0.02	0.132
Diacylglycerol (mmol/l)	755	-0.01	-0.08	0.06	0.762	755	0.00	-0.08	0.07	0.901	755	0.00	-0.07	0.08	0.950
Ratio of diacylglycerol to triglycerides	755	0.03	-0.05	0.11	0.437	755	0.03	-0.05	0.12	0.448	755	0.04	-0.05	0.12	0.415
Total phosphoglycerides (mmol/l)	755	0.04	-0.03	0.11	0.257	755	0.03	-0.05	0.10	0.471	755	0.02	-0.05	0.09	0.544

S3 Table Associations of current moderate-to-vigorous physical activity (MVPA at age 15y) with metabolic traits at age 15y in ALSPAC

MVPA at age 15y (per SD (18 min/day) higher)

	Adj. for age, sex, ethnicity, maternal education, smoking, alcohol, wear time, wear month					*Additionally adj. for SED at age 15y*					*Additionally adj. for FMI at age 15y*				
**Standardised outcome at age 15y**	**N**	**Beta**	**LCL**	**UCL**	**P-value**	**N**	**Beta**	**LCL**	**UCL**	**P-value**	**N**	**Beta**	**LCL**	**UCL**	**P-value**
Ratio of triglycerides to phosphoglycerides	755	-0.10	-0.16	-0.03	0.004	755	-0.08	-0.15	-0.02	0.014	755	-0.07	-0.14	-0.01	0.029
Phosphatidylcholine and other cholines (mmol/l)	755	0.05	-0.02	0.12	0.183	755	0.04	-0.03	0.11	0.285	755	0.03	-0.04	0.11	0.334
Total cholines (mmol/l)	755	0.04	-0.03	0.12	0.214	755	0.03	-0.04	0.10	0.422	755	0.03	-0.05	0.10	0.487
Apolipoprotein A-I (g/l)	755	0.07	-0.01	0.15	0.069	755	0.06	-0.02	0.14	0.123	755	0.05	-0.02	0.13	0.173
Apolipoprotein B (g/l)	755	-0.08	-0.16	-0.01	0.028	755	-0.09	-0.17	-0.01	0.027	755	-0.08	-0.16	0.00	0.050
Ratio of apolipoprotein B to apolipoprotein A-I	755	-0.12	-0.20	-0.03	0.005	755	-0.12	-0.20	-0.03	0.008	755	-0.10	-0.19	-0.02	0.017
Total fatty acids (mmol/l)	755	-0.04	-0.11	0.03	0.243	755	-0.05	-0.12	0.02	0.190	755	-0.04	-0.11	0.03	0.236
Estimated description of fatty acid chain length, not actual carbon number	755	0.01	-0.06	0.08	0.836	755	0.02	-0.05	0.09	0.598	755	0.02	-0.05	0.09	0.529
Estimated degree of unsaturation	755	0.05	-0.03	0.12	0.233	755	0.06	-0.02	0.14	0.154	755	0.06	-0.02	0.14	0.150
22:6, docosahexaenoic acid (mmol/l)	755	0.02	-0.06	0.10	0.629	755	0.00	-0.08	0.08	0.913	755	0.01	-0.07	0.09	0.850
18:2, linoleic acid (mmol/l)	755	0.00	-0.07	0.07	0.942	755	-0.01	-0.08	0.06	0.794	755	-0.01	-0.08	0.06	0.786
Conjugated linoleic acid (mmol/l)	755	-0.01	-0.08	0.05	0.671	755	-0.01	-0.08	0.06	0.736	755	-0.01	-0.08	0.06	0.727
Omega-3 fatty acids (mmol/l)	755	-0.02	-0.10	0.06	0.640	755	-0.03	-0.11	0.05	0.475	755	-0.03	-0.11	0.06	0.528
Omega-6 fatty acids (mmol/l)	755	-0.01	-0.08	0.06	0.861	755	-0.01	-0.09	0.06	0.734	755	-0.01	-0.09	0.06	0.755
Polyunsaturated fatty acids (mmol/l)	755	-0.01	-0.08	0.06	0.816	755	-0.02	-0.09	0.06	0.674	755	-0.01	-0.09	0.06	0.702
Monounsaturated fatty acids; 16:1, 18:1 (mmol/l)	755	-0.07	-0.13	0.00	0.051	755	-0.07	-0.14	0.00	0.051	755	-0.06	-0.13	0.01	0.082
Saturated fatty acids (mmol/l)	755	-0.04	-0.11	0.04	0.332	755	-0.04	-0.12	0.03	0.244	755	-0.04	-0.11	0.03	0.277
Ratio of 22:6 docosahexaenoic acid to total fatty acids (%)	755	0.04	-0.04	0.13	0.314	755	0.03	-0.06	0.12	0.503	755	0.03	-0.06	0.12	0.483
Ratio of 18:2 linoleic acid to total fatty acids (%)	755	0.06	-0.02	0.14	0.133	755	0.07	-0.02	0.15	0.118	755	0.06	-0.02	0.14	0.161
Ratio of conjugated linoleic acid to total fatty acids (%)	755	-0.01	-0.08	0.05	0.700	755	-0.01	-0.08	0.06	0.810	755	-0.01	-0.08	0.06	0.800
Ratio of omega-3 fatty acids to total fatty acids (%)	755	0.00	-0.08	0.08	0.982	755	-0.01	-0.09	0.08	0.891	755	-0.01	-0.09	0.08	0.895
Ratio of omega-6 fatty acids to total fatty acids (%)	755	0.07	-0.01	0.14	0.095	755	0.07	-0.01	0.15	0.070	755	0.07	-0.01	0.15	0.096
Ratio of polyunsaturated fatty acids to total fatty acids (%)	755	0.06	-0.01	0.14	0.102	755	0.07	-0.01	0.15	0.080	755	0.06	-0.01	0.14	0.107
Ratio of monounsaturated fatty acids to total fatty acids (%)	755	-0.06	-0.14	0.01	0.113	755	-0.06	-0.14	0.02	0.132	755	-0.05	-0.13	0.03	0.188
Ratio of saturated fatty acids to total fatty acids (%)	755	0.00	-0.07	0.08	0.909	755	-0.01	-0.09	0.08	0.900	755	-0.01	-0.09	0.07	0.829
Insulin (mu/l)	755	-0.05	-0.10	0.00	0.041	755	-0.05	-0.10	0.01	0.079	755	-0.03	-0.08	0.01	0.159
Glucose (mmol/l)	755	-0.03	-0.10	0.03	0.309	755	-0.02	-0.09	0.05	0.523	755	-0.02	-0.09	0.05	0.566
Lactate (mmol/l)	755	-0.03	-0.11	0.06	0.521	755	-0.01	-0.10	0.07	0.782	755	-0.01	-0.10	0.07	0.769
Pyruvate (mmol/l)	755	-0.05	-0.13	0.02	0.161	755	-0.03	-0.11	0.05	0.471	755	-0.02	-0.10	0.05	0.541
Citrate (mmol/l)	755	0.06	-0.03	0.14	0.208	755	0.03	-0.05	0.12	0.447	755	0.03	-0.06	0.11	0.556
Alanine (mmol/l)	755	-0.01	-0.09	0.06	0.736	755	0.03	-0.05	0.10	0.512	755	0.03	-0.05	0.10	0.500
Glutamine (mmol/l)	755	0.03	-0.04	0.10	0.398	755	0.01	-0.06	0.09	0.708	755	0.01	-0.07	0.08	0.887
Histidine (mmol/l)	755	0.06	-0.02	0.14	0.173	755	0.08	-0.01	0.16	0.068	755	0.08	-0.01	0.16	0.067
Isoleucine (mmol/l)	755	-0.01	-0.08	0.06	0.709	755	0.00	-0.07	0.08	0.919	755	0.01	-0.06	0.08	0.779
Leucine (mmol/l)	755	0.06	0.00	0.13	0.052	755	0.07	0.00	0.14	0.035	755	0.07	0.01	0.14	0.031
Valine (mmol/l)	755	0.02	-0.05	0.10	0.561	755	0.03	-0.05	0.11	0.437	755	0.04	-0.04	0.12	0.370
Phenylalanine (mmol/l)	755	0.09	0.01	0.17	0.026	755	0.08	0.00	0.16	0.052	755	0.08	0.00	0.16	0.042
Tyrosine (mmol/l)	755	0.05	-0.03	0.13	0.233	755	0.04	-0.05	0.12	0.420	755	0.04	-0.04	0.13	0.342
Acetate (mmol/l)	755	0.07	0.00	0.15	0.053	755	0.08	0.01	0.16	0.037	755	0.08	0.00	0.16	0.043
Acetoacetate (mmol/l)	755	-0.03	-0.09	0.03	0.372	755	-0.03	-0.10	0.04	0.375	755	-0.03	-0.09	0.04	0.386
3-hydroxybutyrate (mmol/l)	755	-0.06	-0.13	0.01	0.080	755	-0.08	-0.15	0.00	0.042	755	-0.08	-0.15	0.00	0.047
Creatinine (mmol/l)	755	-0.04	-0.12	0.04	0.312	755	-0.02	-0.10	0.06	0.656	755	-0.02	-0.10	0.06	0.651
Albumin (signal area)	755	-0.09	-0.16	-0.01	0.019	755	-0.07	-0.15	0.01	0.087	755	-0.07	-0.15	0.01	0.080
Glycoprotein acetyls, mainly a1-acid glycoprotein (mmol/l)	755	-0.13	-0.20	-0.06	1.61E-04	755	-0.13	-0.20	-0.06	2.39E-04	755	-0.12	-0.18	-0.05	4.15E-04
C-reactive protein (mg/l)	755	-0.02	-0.06	0.02	0.265	755	-0.01	-0.06	0.04	0.684	755	-0.01	-0.05	0.04	0.778
