# Supplementary material for: Associations of device-measured physical activity across adolescence with metabolic traits: Prospective cohort study
Source: PLoS Med. 2018 Sep 11;15(9):e1002649. doi: 10.1371/journal.pmed.1002649 (PMC6133272; doi:10.1371/journal.pmed.1002649)
Supplement: S4 Table — ALSPAC, Avon Longitudinal Study of Parents and Children; SED, sedentary time. (PDF) [file pmed.1002649.s004.pdf]

**S4 Table** Associations of current sedentary time (SED at age 15y) with metabolic traits at age 15y in ALSPAC

| | SED at age 15y (per SD (66 min/day) higher) | | | | | | | | | | | | | | |
|---|---|---|---|---|---|---|---|---|---|---|---|---|---|---|---|
| | *Adj. for age, sex, ethnicity, maternal education, smoking, alcohol, wear time, wear month* | | | | | *Additionally adj. for MVPA at age 15y* | | | | | *Additionally adj. for FMI at age 15y* | | | | |
| **Standardised outcome at age 15y** | **N** | **Beta** | **LCL** | **UCL** | **P-value** | **N** | **Beta** | **LCL** | **UCL** | **P-value** | **N** | **Beta** | **LCL** | **UCL** | **P-value** |
| Systolic blood pressure (mmHg) | 1812 | 0.01 | -0.04 | 0.06 | 0.789 | 1812 | -0.01 | -0.06 | 0.05 | 0.746 | 1782 | -0.01 | -0.06 | 0.05 | 0.821 |
| Diastolic blood pressure (mmHg) | 1812 | 0.04 | -0.01 | 0.09 | 0.108 | 1812 | 0.05 | -0.01 | 0.10 | 0.089 | 1782 | 0.04 | -0.01 | 0.09 | 0.127 |
| Concentration of chylomicrons and extremely large VLDL particles (mol/l) | 1207 | 0.08 | 0.02 | 0.15 | 0.012 | 1207 | 0.04 | -0.03 | 0.10 | 0.273 | 1195 | 0.03 | -0.03 | 0.10 | 0.292 |
| Total lipids in chylomicrons and extremely large VLDL (mmol/l) | 1207 | 0.09 | 0.02 | 0.15 | 0.011 | 1207 | 0.04 | -0.03 | 0.11 | 0.254 | 1195 | 0.04 | -0.03 | 0.10 | 0.275 |
| Phospholipids in chylomicrons and extremely large VLDL (mmol/l) | 1207 | 0.08 | 0.02 | 0.15 | 0.013 | 1207 | 0.04 | -0.03 | 0.10 | 0.280 | 1195 | 0.03 | -0.03 | 0.10 | 0.304 |
| Total cholesterol in chylomicrons and extremely large VLDL (mmol/l) | 1207 | 0.08 | 0.01 | 0.14 | 0.023 | 1207 | 0.03 | -0.03 | 0.10 | 0.322 | 1195 | 0.03 | -0.03 | 0.10 | 0.342 |
| Cholesterol esters in chylomicrons and extremely large VLDL (mmol/l) | 1207 | 0.07 | 0.00 | 0.13 | 0.049 | 1207 | 0.03 | -0.04 | 0.10 | 0.386 | 1195 | 0.03 | -0.04 | 0.09 | 0.402 |
| Free cholesterol in chylomicrons and extremely large VLDL (mmol/l) | 1207 | 0.08 | 0.02 | 0.15 | 0.014 | 1207 | 0.04 | -0.03 | 0.10 | 0.286 | 1195 | 0.03 | -0.03 | 0.10 | 0.312 |
| Triglycerides in chylomicrons and extremely large VLDL (mmol/l) | 1207 | 0.09 | 0.02 | 0.15 | 0.009 | 1207 | 0.04 | -0.03 | 0.11 | 0.239 | 1195 | 0.04 | -0.03 | 0.10 | 0.260 |
| Concentration of very large VLDL particles (mol/l) | 1207 | 0.09 | 0.03 | 0.16 | 0.007 | 1207 | 0.05 | -0.02 | 0.11 | 0.178 | 1195 | 0.04 | -0.02 | 0.11 | 0.194 |
| Total lipids in very large VLDL (mmol/l) | 1207 | 0.09 | 0.02 | 0.16 | 0.008 | 1207 | 0.04 | -0.02 | 0.11 | 0.190 | 1195 | 0.04 | -0.02 | 0.11 | 0.207 |
| Phospholipids in very large VLDL (mmol/l) | 1207 | 0.08 | 0.02 | 0.15 | 0.013 | 1207 | 0.04 | -0.03 | 0.11 | 0.256 | 1195 | 0.04 | -0.03 | 0.10 | 0.277 |
| Total cholesterol in very large VLDL (mmol/l) | 1207 | 0.08 | 0.02 | 0.15 | 0.013 | 1207 | 0.04 | -0.03 | 0.11 | 0.263 | 1195 | 0.04 | -0.03 | 0.10 | 0.277 |
| Cholesterol esters in very large VLDL (mmol/l) | 1207 | 0.09 | 0.02 | 0.15 | 0.011 | 1207 | 0.04 | -0.03 | 0.11 | 0.227 | 1195 | 0.04 | -0.03 | 0.10 | 0.236 |
| Free cholesterol in very large VLDL (mmol/l) | 1207 | 0.08 | 0.01 | 0.15 | 0.017 | 1207 | 0.03 | -0.03 | 0.10 | 0.312 | 1195 | 0.03 | -0.03 | 0.10 | 0.332 |
| Triglycerides in very large VLDL (mmol/l) | 1207 | 0.09 | 0.03 | 0.16 | 0.006 | 1207 | 0.05 | -0.02 | 0.12 | 0.160 | 1195 | 0.04 | -0.02 | 0.11 | 0.176 |
| Concentration of large VLDL particles (mol/l) | 1207 | 0.09 | 0.02 | 0.16 | 0.007 | 1207 | 0.05 | -0.02 | 0.12 | 0.169 | 1195 | 0.04 | -0.02 | 0.11 | 0.183 |
| Total lipids in large VLDL (mmol/l) | 1207 | 0.09 | 0.02 | 0.16 | 0.008 | 1207 | 0.05 | -0.02 | 0.12 | 0.179 | 1195 | 0.04 | -0.02 | 0.11 | 0.194 |
| Phospholipids in large VLDL (mmol/l) | 1207 | 0.09 | 0.02 | 0.15 | 0.011 | 1207 | 0.04 | -0.02 | 0.11 | 0.212 | 1195 | 0.04 | -0.03 | 0.11 | 0.229 |
| Total cholesterol in large VLDL (mmol/l) | 1207 | 0.08 | 0.02 | 0.15 | 0.014 | 1207 | 0.04 | -0.03 | 0.11 | 0.234 | 1195 | 0.04 | -0.03 | 0.10 | 0.248 |
| Cholesterol esters in large VLDL (mmol/l) | 1207 | 0.08 | 0.01 | 0.15 | 0.020 | 1207 | 0.04 | -0.03 | 0.11 | 0.261 | 1195 | 0.04 | -0.03 | 0.10 | 0.273 |
| Free cholesterol in large VLDL (mmol/l) | 1207 | 0.09 | 0.02 | 0.15 | 0.011 | 1207 | 0.04 | -0.03 | 0.11 | 0.215 | 1195 | 0.04 | -0.03 | 0.11 | 0.233 |
| Triglycerides in large VLDL (mmol/l) | 1207 | 0.09 | 0.03 | 0.16 | 0.006 | 1207 | 0.05 | -0.02 | 0.12 | 0.155 | 1195 | 0.05 | -0.02 | 0.11 | 0.168 |
| Concentration of medium VLDL particles (mol/l) | 1207 | 0.09 | 0.02 | 0.16 | 0.009 | 1207 | 0.05 | -0.02 | 0.12 | 0.195 | 1195 | 0.04 | -0.02 | 0.11 | 0.206 |
| Total lipids in medium VLDL (mmol/l) | 1207 | 0.09 | 0.02 | 0.16 | 0.011 | 1207 | 0.04 | -0.03 | 0.11 | 0.211 | 1195 | 0.04 | -0.03 | 0.11 | 0.221 |
| Phospholipids in medium VLDL (mmol/l) | 1207 | 0.09 | 0.02 | 0.15 | 0.014 | 1207 | 0.04 | -0.03 | 0.11 | 0.248 | 1195 | 0.04 | -0.03 | 0.11 | 0.262 |
| Total cholesterol in medium VLDL (mmol/l) | 1207 | 0.07 | 0.01 | 0.14 | 0.032 | 1207 | 0.03 | -0.04 | 0.10 | 0.330 | 1195 | 0.03 | -0.03 | 0.10 | 0.340 |
| Cholesterol esters in medium VLDL (mmol/l) | 1207 | 0.06 | -0.01 | 0.13 | 0.073 | 1207 | 0.03 | -0.04 | 0.10 | 0.433 | 1195 | 0.03 | -0.04 | 0.09 | 0.436 |
| Free cholesterol in medium VLDL (mmol/l) | 1207 | 0.08 | 0.02 | 0.15 | 0.015 | 1207 | 0.04 | -0.03 | 0.11 | 0.253 | 1195 | 0.04 | -0.03 | 0.10 | 0.270 |
| Triglycerides in medium VLDL (mmol/l) | 1207 | 0.09 | 0.03 | 0.16 | 0.006 | 1207 | 0.05 | -0.02 | 0.12 | 0.166 | 1195 | 0.05 | -0.02 | 0.11 | 0.176 |
| Concentration of small VLDL particles (mol/l) | 1207 | 0.07 | 0.00 | 0.13 | 0.052 | 1207 | 0.02 | -0.05 | 0.09 | 0.533 | 1195 | 0.02 | -0.05 | 0.09 | 0.556 |
| Total lipids in small VLDL (mmol/l) | 1207 | 0.06 | -0.01 | 0.12 | 0.092 | 1207 | 0.01 | -0.06 | 0.08 | 0.719 | 1195 | 0.01 | -0.06 | 0.08 | 0.736 |
| Phospholipids in small VLDL (mmol/l) | 1207 | 0.04 | -0.02 | 0.11 | 0.194 | 1207 | 0.00 | -0.07 | 0.07 | 0.966 | 1195 | 0.00 | -0.06 | 0.07 | 0.986 |
| Total cholesterol in small VLDL (mmol/l) | 1207 | 0.03 | -0.03 | 0.10 | 0.315 | 1207 | -0.01 | -0.08 | 0.06 | 0.844 | 1195 | -0.01 | -0.07 | 0.06 | 0.861 |
| Cholesterol esters in small VLDL (mmol/l) | 1207 | 0.03 | -0.04 | 0.09 | 0.439 | 1207 | -0.01 | -0.08 | 0.06 | 0.712 | 1195 | -0.01 | -0.08 | 0.06 | 0.741 |
| Free cholesterol in small VLDL (mmol/l) | 1207 | 0.05 | -0.02 | 0.11 | 0.175 | 1207 | 0.01 | -0.06 | 0.07 | 0.879 | 1195 | 0.00 | -0.06 | 0.07 | 0.889 |
| Triglycerides in small VLDL (mmol/l) | 1207 | 0.08 | 0.01 | 0.14 | 0.026 | 1207 | 0.03 | -0.04 | 0.10 | 0.354 | 1195 | 0.03 | -0.04 | 0.10 | 0.385 |
| Concentration of very small VLDL particles (mol/l) | 1207 | 0.00 | -0.06 | 0.07 | 0.914 | 1207 | -0.02 | -0.09 | 0.05 | 0.575 | 1195 | -0.02 | -0.08 | 0.05 | 0.611 |
| Total lipids in very small VLDL (mmol/l) | 1207 | 0.01 | -0.06 | 0.08 | 0.757 | 1207 | -0.02 | -0.09 | 0.05 | 0.622 | 1195 | -0.01 | -0.08 | 0.05 | 0.667 |
| Phospholipids in very small VLDL (mmol/l) | 1207 | -0.01 | -0.08 | 0.05 | 0.691 | 1207 | -0.03 | -0.10 | 0.03 | 0.324 | 1195 | -0.03 | -0.10 | 0.04 | 0.371 |
| Total cholesterol in very small VLDL (mmol/l) | 1207 | 0.01 | -0.05 | 0.08 | 0.710 | 1207 | -0.01 | -0.08 | 0.06 | 0.800 | 1195 | -0.01 | -0.07 | 0.06 | 0.863 |
| Cholesterol esters in very small VLDL (mmol/l) | 1207 | 0.02 | -0.05 | 0.09 | 0.618 | 1207 | -0.01 | -0.08 | 0.06 | 0.781 | 1195 | -0.01 | -0.08 | 0.06 | 0.836 |
| Free cholesterol in very small VLDL (mmol/l) | 1207 | 0.00 | -0.06 | 0.07 | 0.947 | 1207 | -0.01 | -0.07 | 0.06 | 0.875 | 1195 | 0.00 | -0.07 | 0.06 | 0.949 |
| Triglycerides in very small VLDL (mmol/l) | 1207 | 0.03 | -0.03 | 0.10 | 0.327 | 1207 | 0.00 | -0.07 | 0.06 | 0.899 | 1195 | -0.01 | -0.07 | 0.06 | 0.864 |
| Concentration of IDL particles (mol/l) | 1207 | -0.02 | -0.08 | 0.05 | 0.596 | 1207 | -0.03 | -0.10 | 0.03 | 0.322 | 1195 | -0.03 | -0.10 | 0.04 | 0.368 |
| Total lipids in IDL (mmol/l) | 1207 | -0.02 | -0.08 | 0.05 | 0.585 | 1207 | -0.03 | -0.10 | 0.03 | 0.311 | 1195 | -0.03 | -0.10 | 0.04 | 0.366 |
| Phospholipids in IDL (mmol/l) | 1207 | -0.03 | -0.09 | 0.03 | 0.354 | 1207 | -0.04 | -0.11 | 0.02 | 0.188 | 1195 | -0.04 | -0.10 | 0.03 | 0.229 |
| Total cholesterol in IDL (mmol/l) | 1207 | -0.01 | -0.07 | 0.05 | 0.745 | 1207 | -0.03 | -0.09 | 0.04 | 0.427 | 1195 | -0.02 | -0.09 | 0.04 | 0.498 |

**S4 Table** Associations of current sedentary time (SED at age 15y) with metabolic traits at age 15y in ALSPAC

| | SED at age 15y (per SD (66 min/day) higher) | | | | | | | | | | | | | | |
|---|---|---|---|---|---|---|---|---|---|---|---|---|---|---|---|
| | *Adj. for age, sex, ethnicity, maternal education, smoking, alcohol, wear time, wear month* | | | | | *Additionally adj. for MVPA at age 15y* | | | | | *Additionally adj. for FMI at age 15y* | | | | |
| **Standardised outcome at age 15y** | **N** | **Beta** | **LCL** | **UCL** | **P-value** | **N** | **Beta** | **LCL** | **UCL** | **P-value** | **N** | **Beta** | **LCL** | **UCL** | **P-value** |
| Cholesterol esters in IDL (mmol/l) | 1207 | 0.00 | -0.07 | 0.06 | 0.954 | 1207 | -0.02 | -0.09 | 0.05 | 0.534 | 1195 | -0.02 | -0.08 | 0.05 | 0.611 |
| Free cholesterol in IDL (mmol/l) | 1207 | -0.03 | -0.09 | 0.03 | 0.339 | 1207 | -0.04 | -0.10 | 0.03 | 0.238 | 1195 | -0.03 | -0.10 | 0.03 | 0.289 |
| Triglycerides in IDL (mmol/l) | 1207 | -0.03 | -0.09 | 0.04 | 0.412 | 1207 | -0.04 | -0.10 | 0.02 | 0.206 | 1195 | -0.04 | -0.10 | 0.02 | 0.207 |
| Concentration of large LDL particles (mol/l) | 1207 | -0.03 | -0.09 | 0.03 | 0.350 | 1207 | -0.05 | -0.11 | 0.02 | 0.147 | 1195 | -0.04 | -0.11 | 0.02 | 0.181 |
| Total lipids in large LDL (mmol/l) | 1207 | -0.03 | -0.09 | 0.03 | 0.384 | 1207 | -0.04 | -0.11 | 0.02 | 0.174 | 1195 | -0.04 | -0.11 | 0.02 | 0.213 |
| Phospholipids in large LDL (mmol/l) | 1207 | -0.03 | -0.09 | 0.03 | 0.373 | 1207 | -0.05 | -0.11 | 0.02 | 0.149 | 1195 | -0.04 | -0.11 | 0.02 | 0.186 |
| Total cholesterol in large LDL (mmol/l) | 1207 | -0.03 | -0.09 | 0.04 | 0.430 | 1207 | -0.04 | -0.11 | 0.02 | 0.208 | 1195 | -0.04 | -0.10 | 0.03 | 0.254 |
| Cholesterol esters in large LDL (mmol/l) | 1207 | -0.02 | -0.08 | 0.04 | 0.496 | 1207 | -0.04 | -0.10 | 0.03 | 0.229 | 1195 | -0.04 | -0.10 | 0.03 | 0.276 |
| Free cholesterol in large LDL (mmol/l) | 1207 | -0.04 | -0.10 | 0.03 | 0.267 | 1207 | -0.05 | -0.11 | 0.02 | 0.156 | 1195 | -0.04 | -0.11 | 0.02 | 0.198 |
| Triglycerides in large LDL (mmol/l) | 1207 | -0.04 | -0.10 | 0.02 | 0.224 | 1207 | -0.05 | -0.11 | 0.01 | 0.119 | 1195 | -0.05 | -0.11 | 0.01 | 0.123 |
| Concentration of medium LDL particles (mol/l) | 1207 | -0.03 | -0.09 | 0.04 | 0.411 | 1207 | -0.05 | -0.11 | 0.02 | 0.141 | 1195 | -0.05 | -0.11 | 0.02 | 0.172 |
| Total lipids in medium LDL (mmol/l) | 1207 | -0.03 | -0.09 | 0.04 | 0.418 | 1207 | -0.05 | -0.11 | 0.02 | 0.161 | 1195 | -0.04 | -0.11 | 0.02 | 0.198 |
| Phospholipids in medium LDL (mmol/l) | 1207 | -0.02 | -0.08 | 0.04 | 0.545 | 1207 | -0.04 | -0.11 | 0.02 | 0.188 | 1195 | -0.04 | -0.10 | 0.02 | 0.229 |
| Total cholesterol in medium LDL (mmol/l) | 1207 | -0.02 | -0.09 | 0.04 | 0.472 | 1207 | -0.04 | -0.11 | 0.02 | 0.198 | 1195 | -0.04 | -0.10 | 0.03 | 0.242 |
| Cholesterol esters in medium LDL (mmol/l) | 1207 | -0.02 | -0.09 | 0.04 | 0.487 | 1207 | -0.04 | -0.11 | 0.02 | 0.199 | 1195 | -0.04 | -0.10 | 0.03 | 0.241 |
| Free cholesterol in medium LDL (mmol/l) | 1207 | -0.03 | -0.09 | 0.04 | 0.425 | 1207 | -0.04 | -0.11 | 0.02 | 0.210 | 1195 | -0.04 | -0.10 | 0.03 | 0.266 |
| Triglycerides in medium LDL (mmol/l) | 1207 | -0.05 | -0.12 | 0.01 | 0.099 | 1207 | -0.06 | -0.13 | 0.00 | 0.047 | 1195 | -0.06 | -0.13 | 0.00 | 0.050 |
| Concentration of small LDL particles (mol/l) | 1207 | -0.02 | -0.08 | 0.04 | 0.511 | 1207 | -0.04 | -0.11 | 0.02 | 0.195 | 1195 | -0.04 | -0.11 | 0.03 | 0.233 |
| Total lipids in small LDL (mmol/l) | 1207 | -0.02 | -0.09 | 0.04 | 0.485 | 1207 | -0.04 | -0.11 | 0.02 | 0.192 | 1195 | -0.04 | -0.10 | 0.03 | 0.232 |
| Phospholipids in small LDL (mmol/l) | 1207 | -0.01 | -0.08 | 0.05 | 0.643 | 1207 | -0.04 | -0.10 | 0.03 | 0.276 | 1195 | -0.03 | -0.10 | 0.03 | 0.323 |
| Total cholesterol in small LDL (mmol/l) | 1207 | -0.02 | -0.09 | 0.04 | 0.451 | 1207 | -0.04 | -0.11 | 0.02 | 0.191 | 1195 | -0.04 | -0.11 | 0.03 | 0.235 |
| Cholesterol esters in small LDL (mmol/l) | 1207 | -0.03 | -0.09 | 0.04 | 0.436 | 1207 | -0.05 | -0.11 | 0.02 | 0.177 | 1195 | -0.04 | -0.11 | 0.02 | 0.215 |
| Free cholesterol in small LDL (mmol/l) | 1207 | -0.02 | -0.08 | 0.04 | 0.560 | 1207 | -0.03 | -0.10 | 0.03 | 0.315 | 1195 | -0.03 | -0.09 | 0.04 | 0.390 |
| Triglycerides in small LDL (mmol/l) | 1207 | -0.02 | -0.08 | 0.05 | 0.577 | 1207 | -0.04 | -0.11 | 0.02 | 0.181 | 1195 | -0.04 | -0.11 | 0.02 | 0.183 |
| Concentration of very large HDL particles (mol/l) | 1207 | -0.05 | -0.11 | 0.02 | 0.160 | 1207 | -0.01 | -0.08 | 0.06 | 0.771 | 1195 | -0.01 | -0.07 | 0.05 | 0.778 |
| Total lipids in very large HDL (mmol/l) | 1207 | -0.04 | -0.10 | 0.02 | 0.226 | 1207 | 0.00 | -0.07 | 0.06 | 0.892 | 1195 | 0.00 | -0.07 | 0.06 | 0.911 |
| Phospholipids in very large HDL (mmol/l) | 1207 | -0.05 | -0.12 | 0.01 | 0.100 | 1207 | -0.02 | -0.08 | 0.05 | 0.646 | 1195 | -0.01 | -0.08 | 0.05 | 0.647 |
| Total cholesterol in very large HDL (mmol/l) | 1207 | -0.03 | -0.09 | 0.04 | 0.427 | 1207 | 0.00 | -0.06 | 0.07 | 0.893 | 1195 | 0.01 | -0.06 | 0.07 | 0.862 |
| Cholesterol esters in very large HDL (mmol/l) | 1207 | -0.02 | -0.09 | 0.04 | 0.505 | 1207 | 0.01 | -0.06 | 0.08 | 0.844 | 1195 | 0.01 | -0.06 | 0.08 | 0.812 |
| Free cholesterol in very large HDL (mmol/l) | 1207 | -0.04 | -0.10 | 0.03 | 0.276 | 1207 | 0.00 | -0.07 | 0.07 | 0.972 | 1195 | 0.00 | -0.06 | 0.06 | 0.997 |
| Triglycerides in very large HDL (mmol/l) | 1207 | 0.04 | -0.02 | 0.11 | 0.210 | 1207 | 0.05 | -0.02 | 0.11 | 0.179 | 1195 | 0.04 | -0.02 | 0.11 | 0.196 |
| Concentration of large HDL particles (mol/l) | 1207 | -0.06 | -0.13 | 0.00 | 0.046 | 1207 | -0.02 | -0.09 | 0.04 | 0.471 | 1195 | -0.02 | -0.09 | 0.04 | 0.472 |
| Total lipids in large HDL (mmol/l) | 1207 | -0.06 | -0.13 | 0.00 | 0.051 | 1207 | -0.02 | -0.09 | 0.04 | 0.502 | 1195 | -0.02 | -0.08 | 0.04 | 0.507 |
| Phospholipids in large HDL (mmol/l) | 1207 | -0.07 | -0.13 | -0.01 | 0.034 | 1207 | -0.03 | -0.10 | 0.03 | 0.346 | 1195 | -0.03 | -0.09 | 0.03 | 0.349 |
| Total cholesterol in large HDL (mmol/l) | 1207 | -0.06 | -0.13 | 0.00 | 0.064 | 1207 | -0.02 | -0.08 | 0.05 | 0.621 | 1195 | -0.02 | -0.08 | 0.05 | 0.630 |
| Cholesterol esters in large HDL (mmol/l) | 1207 | -0.06 | -0.13 | 0.00 | 0.063 | 1207 | -0.02 | -0.08 | 0.05 | 0.624 | 1195 | -0.02 | -0.08 | 0.05 | 0.630 |
| Free cholesterol in large HDL (mmol/l) | 1207 | -0.06 | -0.12 | 0.00 | 0.067 | 1207 | -0.02 | -0.08 | 0.05 | 0.611 | 1195 | -0.02 | -0.08 | 0.05 | 0.628 |
| Triglycerides in large HDL (mmol/l) | 1207 | 0.01 | -0.05 | 0.07 | 0.705 | 1207 | 0.02 | -0.04 | 0.09 | 0.469 | 1195 | 0.02 | -0.04 | 0.08 | 0.512 |
| Concentration of medium HDL particles (mol/l) | 1207 | -0.07 | -0.13 | -0.01 | 0.031 | 1207 | -0.06 | -0.12 | 0.01 | 0.076 | 1195 | -0.05 | -0.12 | 0.01 | 0.089 |
| Total lipids in medium HDL (mmol/l) | 1207 | -0.07 | -0.13 | -0.01 | 0.027 | 1207 | -0.06 | -0.12 | 0.01 | 0.079 | 1195 | -0.05 | -0.12 | 0.01 | 0.093 |
| Phospholipids in medium HDL (mmol/l) | 1207 | -0.08 | -0.14 | -0.02 | 0.014 | 1207 | -0.06 | -0.13 | 0.00 | 0.046 | 1195 | -0.06 | -0.12 | 0.00 | 0.054 |
| Total cholesterol in medium HDL (mmol/l) | 1207 | -0.06 | -0.12 | 0.00 | 0.057 | 1207 | -0.04 | -0.11 | 0.02 | 0.196 | 1195 | -0.04 | -0.10 | 0.02 | 0.223 |
| Cholesterol esters in medium HDL (mmol/l) | 1207 | -0.06 | -0.12 | 0.00 | 0.060 | 1207 | -0.04 | -0.11 | 0.02 | 0.218 | 1195 | -0.04 | -0.10 | 0.03 | 0.246 |
| Free cholesterol in medium HDL (mmol/l) | 1207 | -0.06 | -0.12 | 0.00 | 0.050 | 1207 | -0.05 | -0.11 | 0.01 | 0.122 | 1195 | -0.05 | -0.11 | 0.02 | 0.145 |
| Triglycerides in medium HDL (mmol/l) | 1207 | 0.02 | -0.05 | 0.08 | 0.633 | 1207 | -0.02 | -0.09 | 0.05 | 0.549 | 1195 | -0.02 | -0.09 | 0.04 | 0.502 |
| Concentration of small HDL particles (mol/l) | 1207 | -0.05 | -0.11 | 0.02 | 0.144 | 1207 | -0.07 | -0.13 | 0.00 | 0.036 | 1195 | -0.07 | -0.13 | 0.00 | 0.035 |
| Total lipids in small HDL (mmol/l) | 1207 | -0.08 | -0.13 | -0.02 | 0.011 | 1207 | -0.09 | -0.15 | -0.03 | 0.005 | 1195 | -0.09 | -0.15 | -0.02 | 0.006 |
| Phospholipids in small HDL (mmol/l) | 1207 | -0.02 | -0.08 | 0.05 | 0.613 | 1207 | -0.04 | -0.10 | 0.03 | 0.272 | 1195 | -0.04 | -0.10 | 0.03 | 0.276 |

**S4 Table** Associations of current sedentary time (SED at age 15y) with metabolic traits at age 15y in ALSPAC

## SED at age 15y (per SD (66 min/day) higher)

| | *Adj. for age, sex, ethnicity, maternal education, smoking, alcohol, wear time, wear month* | | | | | *Additionally adj. for MVPA at age 15y* | | | | | *Additionally adj. for FMI at age 15y* | | | | |
|---|---|---|---|---|---|---|---|---|---|---|---|---|---|---|---|
| **Standardised outcome at age 15y** | **N** | **Beta** | **LCL** | **UCL** | **P-value** | **N** | **Beta** | **LCL** | **UCL** | **P-value** | **N** | **Beta** | **LCL** | **UCL** | **P-value** |
| Total cholesterol in small HDL (mmol/l) | 1207 | -0.12 | -0.18 | -0.06 | 2.63E-05 | 1207 | -0.11 | -0.17 | -0.06 | 1.33E-04 | 1195 | -0.11 | -0.17 | -0.05 | 2.43E-04 |
| Cholesterol esters in small HDL (mmol/l) | 1207 | -0.13 | -0.18 | -0.07 | 9.88E-06 | 1207 | -0.12 | -0.18 | -0.06 | 5.62E-05 | 1195 | -0.11 | -0.17 | -0.06 | 1.08E-04 |
| Free cholesterol in small HDL (mmol/l) | 1207 | -0.05 | -0.11 | 0.02 | 0.156 | 1207 | -0.04 | -0.11 | 0.02 | 0.208 | 1195 | -0.04 | -0.11 | 0.03 | 0.232 |
| Triglycerides in small HDL (mmol/l) | 1207 | 0.02 | -0.04 | 0.09 | 0.482 | 1207 | -0.02 | -0.08 | 0.05 | 0.606 | 1195 | -0.02 | -0.08 | 0.04 | 0.537 |
| Phospholipids to total lipds ratio in chylomicrons and extremely large VLDL (%) | 1207 | 0.01 | -0.05 | 0.08 | 0.697 | 1207 | -0.01 | -0.08 | 0.06 | 0.800 | 1195 | -0.01 | -0.08 | 0.06 | 0.767 |
| Total cholesterol to total lipids ratio in chylomicrons and extremely large VLDL (%) | 1207 | -0.01 | -0.07 | 0.06 | 0.867 | 1207 | -0.02 | -0.09 | 0.05 | 0.580 | 1195 | -0.02 | -0.09 | 0.05 | 0.568 |
| Cholesterol esters to total lipids ratio in chylomicrons and extremely large VLDL (%) | 1207 | -0.02 | -0.09 | 0.04 | 0.503 | 1207 | -0.03 | -0.10 | 0.04 | 0.400 | 1195 | -0.03 | -0.10 | 0.04 | 0.404 |
| Free cholesterol to total lipids ratio in chylomicrons and extremely large VLDL (%) | 1207 | 0.04 | -0.03 | 0.11 | 0.219 | 1207 | 0.01 | -0.06 | 0.09 | 0.691 | 1195 | 0.01 | -0.06 | 0.08 | 0.750 |
| Triglycerides to total lipids ratio in chylomicrons and extremely large VLDL (%) | 1207 | 0.01 | -0.04 | 0.05 | 0.824 | 1207 | 0.02 | -0.03 | 0.07 | 0.511 | 1195 | 0.02 | -0.03 | 0.07 | 0.491 |
| Phospholipids to total lipds ratio in very large VLDL (%) | 1207 | 0.03 | -0.04 | 0.09 | 0.473 | 1207 | -0.01 | -0.08 | 0.06 | 0.821 | 1195 | -0.01 | -0.08 | 0.06 | 0.776 |
| Total cholesterol to total lipids ratio in very large VLDL (%) | 1207 | -0.10 | -0.17 | -0.02 | 0.017 | 1207 | -0.07 | -0.13 | 0.00 | 0.060 | 1195 | -0.06 | -0.13 | 0.01 | 0.073 |
| Cholesterol esters to total lipids ratio in very large VLDL (%) | 1207 | -0.08 | -0.14 | -0.01 | 0.026 | 1207 | -0.05 | -0.12 | 0.02 | 0.158 | 1195 | -0.05 | -0.11 | 0.02 | 0.187 |
| Free cholesterol to total lipids ratio in very large VLDL (%) | 1207 | -0.07 | -0.14 | -0.01 | 0.027 | 1207 | -0.06 | -0.13 | 0.01 | 0.086 | 1195 | -0.06 | -0.13 | 0.01 | 0.105 |
| Triglycerides to total lipids ratio in very large VLDL (%) | 1207 | 0.07 | 0.01 | 0.14 | 0.033 | 1207 | 0.06 | -0.01 | 0.13 | 0.094 | 1195 | 0.06 | -0.01 | 0.12 | 0.112 |
| Phospholipids to total lipds ratio in large VLDL (%) | 1207 | 0.05 | -0.03 | 0.12 | 0.209 | 1207 | 0.02 | -0.06 | 0.09 | 0.691 | 1195 | 0.01 | -0.06 | 0.09 | 0.736 |
| Total cholesterol to total lipids ratio in large VLDL (%) | 1207 | 0.01 | -0.06 | 0.08 | 0.729 | 1207 | 0.00 | -0.08 | 0.07 | 0.903 | 1195 | -0.01 | -0.08 | 0.07 | 0.883 |
| Cholesterol esters to total lipids ratio in large VLDL (%) | 1207 | -0.05 | -0.15 | 0.05 | 0.292 | 1207 | -0.03 | -0.10 | 0.03 | 0.304 | 1195 | -0.03 | -0.10 | 0.03 | 0.317 |
| Free cholesterol to total lipids ratio in large VLDL (%) | 1207 | 0.05 | -0.02 | 0.12 | 0.164 | 1207 | 0.02 | -0.06 | 0.09 | 0.661 | 1195 | 0.01 | -0.06 | 0.09 | 0.730 |
| Triglycerides to total lipids ratio in large VLDL (%) | 1207 | -0.06 | -0.17 | 0.05 | 0.281 | 1207 | -0.03 | -0.10 | 0.03 | 0.323 | 1195 | -0.03 | -0.10 | 0.03 | 0.327 |
| Phospholipids to total lipds ratio in medium VLDL (%) | 1207 | -0.06 | -0.13 | 0.01 | 0.083 | 1207 | -0.04 | -0.11 | 0.03 | 0.269 | 1195 | -0.04 | -0.11 | 0.03 | 0.289 |
| Total cholesterol to total lipids ratio in medium VLDL (%) | 1207 | 0.00 | -0.07 | 0.06 | 0.930 | 1207 | 0.00 | -0.07 | 0.06 | 0.892 | 1195 | 0.00 | -0.07 | 0.07 | 0.933 |
| Cholesterol esters to total lipids ratio in medium VLDL (%) | 1207 | -0.01 | -0.08 | 0.06 | 0.769 | 1207 | -0.01 | -0.07 | 0.06 | 0.869 | 1195 | 0.00 | -0.07 | 0.07 | 0.929 |
| Free cholesterol to total lipids ratio in medium VLDL (%) | 1207 | 0.02 | -0.04 | 0.09 | 0.510 | 1207 | 0.00 | -0.06 | 0.07 | 0.946 | 1195 | 0.00 | -0.07 | 0.07 | 0.999 |
| Triglycerides to total lipids ratio in medium VLDL (%) | 1207 | 0.02 | -0.05 | 0.08 | 0.625 | 1207 | 0.01 | -0.05 | 0.08 | 0.699 | 1195 | 0.01 | -0.06 | 0.08 | 0.747 |
| Phospholipids to total lipds ratio in small VLDL (%) | 1207 | -0.09 | -0.17 | -0.02 | 0.010 | 1207 | -0.05 | -0.13 | 0.02 | 0.166 | 1195 | -0.05 | -0.12 | 0.02 | 0.189 |
| Total cholesterol to total lipids ratio in small VLDL (%) | 1207 | -0.04 | -0.10 | 0.03 | 0.233 | 1207 | -0.04 | -0.11 | 0.03 | 0.280 | 1195 | -0.03 | -0.10 | 0.04 | 0.358 |
| Cholesterol esters to total lipids ratio in small VLDL (%) | 1207 | -0.03 | -0.10 | 0.03 | 0.354 | 1207 | -0.03 | -0.10 | 0.03 | 0.324 | 1195 | -0.03 | -0.10 | 0.04 | 0.403 |
| Free cholesterol to total lipids ratio in small VLDL (%) | 1207 | -0.07 | -0.14 | -0.01 | 0.034 | 1207 | -0.04 | -0.11 | 0.03 | 0.310 | 1195 | -0.03 | -0.10 | 0.04 | 0.367 |
| Triglycerides to total lipids ratio in small VLDL (%) | 1207 | 0.07 | 0.00 | 0.13 | 0.043 | 1207 | 0.05 | -0.01 | 0.12 | 0.128 | 1195 | 0.05 | -0.02 | 0.11 | 0.179 |
| Phospholipids to total lipds ratio in very small VLDL (%) | 1207 | -0.04 | -0.10 | 0.02 | 0.214 | 1207 | -0.04 | -0.10 | 0.02 | 0.220 | 1195 | -0.03 | -0.09 | 0.03 | 0.262 |
| Total cholesterol to total lipids ratio in very small VLDL (%) | 1207 | 0.00 | -0.06 | 0.06 | 0.978 | 1207 | 0.02 | -0.05 | 0.08 | 0.608 | 1195 | 0.02 | -0.05 | 0.08 | 0.576 |
| Cholesterol esters to total lipids ratio in very small VLDL (%) | 1207 | 0.01 | -0.06 | 0.07 | 0.817 | 1207 | 0.01 | -0.06 | 0.07 | 0.828 | 1195 | 0.01 | -0.06 | 0.07 | 0.813 |
| Free cholesterol to total lipids ratio in very small VLDL (%) | 1207 | -0.01 | -0.07 | 0.05 | 0.711 | 1207 | 0.03 | -0.03 | 0.09 | 0.374 | 1195 | 0.03 | -0.03 | 0.09 | 0.332 |
| Triglycerides to total lipids ratio in very small VLDL (%) | 1207 | 0.02 | -0.04 | 0.09 | 0.422 | 1207 | 0.01 | -0.06 | 0.07 | 0.838 | 1195 | 0.00 | -0.06 | 0.07 | 0.944 |
| Phospholipids to total lipds ratio in IDL (%) | 1207 | -0.08 | -0.14 | -0.01 | 0.026 | 1207 | -0.06 | -0.13 | 0.01 | 0.109 | 1195 | -0.06 | -0.13 | 0.01 | 0.108 |
| Total cholesterol to total lipids ratio in IDL (%) | 1207 | 0.05 | -0.02 | 0.11 | 0.136 | 1207 | 0.04 | -0.03 | 0.11 | 0.236 | 1195 | 0.04 | -0.02 | 0.11 | 0.201 |
| Cholesterol esters to total lipids ratio in IDL (%) | 1207 | 0.07 | 0.00 | 0.14 | 0.042 | 1207 | 0.05 | -0.02 | 0.12 | 0.179 | 1195 | 0.05 | -0.02 | 0.12 | 0.158 |
| Free cholesterol to total lipids ratio in IDL (%) | 1207 | -0.04 | -0.10 | 0.01 | 0.133 | 1207 | -0.01 | -0.08 | 0.05 | 0.642 | 1195 | -0.01 | -0.07 | 0.05 | 0.729 |
| Triglycerides to total lipids ratio in IDL (%) | 1207 | -0.02 | -0.09 | 0.04 | 0.471 | 1207 | -0.02 | -0.09 | 0.04 | 0.505 | 1195 | -0.03 | -0.09 | 0.04 | 0.436 |
| Phospholipids to total lipds ratio in large LDL (%) | 1207 | -0.01 | -0.07 | 0.05 | 0.702 | 1207 | -0.01 | -0.07 | 0.05 | 0.809 | 1195 | -0.01 | -0.07 | 0.05 | 0.724 |
| Total cholesterol to total lipids ratio in large LDL (%) | 1207 | 0.02 | -0.03 | 0.08 | 0.431 | 1207 | 0.02 | -0.04 | 0.08 | 0.596 | 1195 | 0.02 | -0.04 | 0.08 | 0.508 |
| Cholesterol esters to total lipids ratio in large LDL (%) | 1207 | 0.03 | -0.03 | 0.09 | 0.334 | 1207 | 0.01 | -0.05 | 0.08 | 0.686 | 1195 | 0.02 | -0.05 | 0.08 | 0.607 |
| Free cholesterol to total lipids ratio in large LDL (%) | 1207 | -0.03 | -0.09 | 0.03 | 0.362 | 1207 | 0.00 | -0.06 | 0.07 | 0.873 | 1195 | 0.01 | -0.05 | 0.06 | 0.867 |
| Triglycerides to total lipids ratio in large LDL (%) | 1207 | -0.03 | -0.09 | 0.04 | 0.383 | 1207 | -0.02 | -0.09 | 0.05 | 0.536 | 1195 | -0.02 | -0.09 | 0.04 | 0.472 |
| Phospholipids to total lipds ratio in medium LDL (%) | 1207 | 0.00 | -0.02 | 0.02 | 0.898 | 1207 | 0.00 | -0.02 | 0.02 | 0.930 | 1195 | 0.00 | -0.02 | 0.02 | 0.978 |
| Total cholesterol to total lipids ratio in medium LDL (%) | 1207 | 0.03 | -0.03 | 0.09 | 0.378 | 1207 | 0.02 | -0.05 | 0.08 | 0.587 | 1195 | 0.02 | -0.04 | 0.09 | 0.508 |
| Cholesterol esters to total lipids ratio in medium LDL (%) | 1207 | 0.02 | -0.04 | 0.08 | 0.529 | 1207 | 0.00 | -0.06 | 0.07 | 0.893 | 1195 | 0.01 | -0.06 | 0.07 | 0.809 |
| Free cholesterol to total lipids ratio in medium LDL (%) | 1207 | 0.00 | -0.02 | 0.02 | 0.860 | 1207 | 0.00 | -0.01 | 0.02 | 0.635 | 1195 | 0.00 | -0.01 | 0.02 | 0.691 |

**S4 Table** Associations of current sedentary time (SED at age 15y) with metabolic traits at age 15y in ALSPAC

| | SED at age 15y (per SD (66 min/day) higher) | | | | | | | | | | | | | | |
|---|---|---|---|---|---|---|---|---|---|---|---|---|---|---|---|
| | *Adj. for age, sex, ethnicity, maternal education, smoking, alcohol, wear time, wear month* | | | | | *Additionally adj. for MVPA at age 15y* | | | | | *Additionally adj. for FMI at age 15y* | | | | |
| **Standardised outcome at age 15y** | **N** | **Beta** | **LCL** | **UCL** | **P-value** | **N** | **Beta** | **LCL** | **UCL** | **P-value** | **N** | **Beta** | **LCL** | **UCL** | **P-value** |
| Triglycerides to total lipids ratio in medium LDL (%) | 1207 | -0.06 | -0.12 | 0.01 | 0.091 | 1207 | -0.05 | -0.11 | 0.02 | 0.170 | 1195 | -0.05 | -0.11 | 0.02 | 0.153 |
| Phospholipids to total lipds ratio in small LDL (%) | 1207 | 0.00 | -0.04 | 0.03 | 0.903 | 1207 | 0.01 | -0.03 | 0.04 | 0.751 | 1195 | 0.00 | -0.03 | 0.04 | 0.851 |
| Total cholesterol to total lipids ratio in small LDL (%) | 1207 | 0.01 | -0.05 | 0.07 | 0.801 | 1207 | 0.00 | -0.06 | 0.06 | 0.997 | 1195 | 0.00 | -0.06 | 0.07 | 0.877 |
| Cholesterol esters to total lipids ratio in small LDL (%) | 1207 | 0.01 | -0.06 | 0.07 | 0.855 | 1207 | -0.01 | -0.07 | 0.06 | 0.810 | 1195 | 0.00 | -0.07 | 0.06 | 0.909 |
| Free cholesterol to total lipids ratio in small LDL (%) | 1207 | 0.00 | -0.03 | 0.03 | 0.960 | 1207 | 0.01 | -0.02 | 0.04 | 0.552 | 1195 | 0.01 | -0.02 | 0.04 | 0.592 |
| Triglycerides to total lipids ratio in small LDL (%) | 1207 | -0.01 | -0.08 | 0.05 | 0.645 | 1207 | -0.03 | -0.09 | 0.03 | 0.363 | 1195 | -0.03 | -0.10 | 0.03 | 0.316 |
| Phospholipids to total lipds ratio in very large HDL (%) | 1207 | -0.07 | -0.13 | -0.01 | 0.030 | 1207 | -0.03 | -0.10 | 0.03 | 0.330 | 1195 | -0.03 | -0.09 | 0.03 | 0.305 |
| Total cholesterol to total lipids ratio in very large HDL (%) | 1207 | 0.06 | 0.00 | 0.12 | 0.065 | 1207 | 0.02 | -0.04 | 0.09 | 0.447 | 1195 | 0.02 | -0.03 | 0.08 | 0.418 |
| Cholesterol esters to total lipids ratio in very large HDL (%) | 1207 | 0.05 | -0.01 | 0.11 | 0.091 | 1207 | 0.02 | -0.04 | 0.08 | 0.551 | 1195 | 0.02 | -0.04 | 0.08 | 0.530 |
| Free cholesterol to total lipids ratio in very large HDL (%) | 1207 | 0.03 | -0.03 | 0.09 | 0.326 | 1207 | 0.05 | -0.01 | 0.11 | 0.132 | 1195 | 0.05 | -0.01 | 0.11 | 0.119 |
| Triglycerides to total lipids ratio in very large HDL (%) | 1207 | 0.08 | 0.01 | 0.14 | 0.024 | 1207 | 0.04 | -0.02 | 0.11 | 0.192 | 1195 | 0.04 | -0.03 | 0.11 | 0.221 |
| Phospholipids to total lipds ratio in large HDL (%) | 1207 | 0.00 | -0.06 | 0.06 | 0.967 | 1207 | -0.05 | -0.11 | 0.02 | 0.147 | 1195 | -0.05 | -0.11 | 0.01 | 0.128 |
| Total cholesterol to total lipids ratio in large HDL (%) | 1207 | -0.03 | -0.10 | 0.04 | 0.380 | 1207 | 0.02 | -0.05 | 0.09 | 0.579 | 1195 | 0.02 | -0.04 | 0.08 | 0.540 |
| Cholesterol esters to total lipids ratio in large HDL (%) | 1207 | -0.03 | -0.10 | 0.03 | 0.349 | 1207 | 0.02 | -0.05 | 0.08 | 0.626 | 1195 | 0.02 | -0.05 | 0.08 | 0.602 |
| Free cholesterol to total lipids ratio in large HDL (%) | 1207 | -0.01 | -0.08 | 0.05 | 0.642 | 1207 | 0.02 | -0.04 | 0.08 | 0.490 | 1195 | 0.02 | -0.04 | 0.09 | 0.420 |
| Triglycerides to total lipids ratio in large HDL (%) | 1207 | 0.08 | 0.01 | 0.15 | 0.018 | 1207 | 0.04 | -0.03 | 0.11 | 0.217 | 1195 | 0.04 | -0.03 | 0.11 | 0.227 |
| Phospholipids to total lipds ratio in medium HDL (%) | 1207 | -0.08 | -0.14 | -0.02 | 0.015 | 1207 | -0.07 | -0.14 | 0.00 | 0.040 | 1195 | -0.07 | -0.13 | 0.00 | 0.045 |
| Total cholesterol to total lipids ratio in medium HDL (%) | 1207 | 0.04 | -0.03 | 0.10 | 0.242 | 1207 | 0.05 | -0.01 | 0.12 | 0.104 | 1195 | 0.06 | -0.01 | 0.12 | 0.103 |
| Cholesterol esters to total lipids ratio in medium HDL (%) | 1207 | 0.04 | -0.03 | 0.10 | 0.280 | 1207 | 0.05 | -0.01 | 0.12 | 0.110 | 1195 | 0.05 | -0.01 | 0.12 | 0.113 |
| Free cholesterol to total lipids ratio in medium HDL (%) | 1207 | 0.02 | -0.04 | 0.09 | 0.491 | 1207 | 0.02 | -0.05 | 0.09 | 0.591 | 1195 | 0.02 | -0.05 | 0.09 | 0.551 |
| Triglycerides to total lipids ratio in medium HDL (%) | 1207 | 0.04 | -0.02 | 0.11 | 0.194 | 1207 | 0.00 | -0.07 | 0.07 | 0.957 | 1195 | 0.00 | -0.07 | 0.07 | 0.978 |
| Phospholipids to total lipds ratio in small HDL (%) | 1207 | 0.11 | 0.05 | 0.17 | 2.35E-04 | 1207 | 0.09 | 0.03 | 0.16 | 2.42E-03 | 1195 | 0.09 | 0.03 | 0.15 | 0.003 |
| Total cholesterol to total lipids ratio in small HDL (%) | 1207 | -0.12 | -0.18 | -0.06 | 9.68E-05 | 1207 | -0.09 | -0.15 | -0.03 | 2.89E-03 | 1195 | -0.09 | -0.15 | -0.03 | 0.004 |
| Cholesterol esters to total lipids ratio in small HDL (%) | 1207 | -0.12 | -0.18 | -0.06 | 7.26E-05 | 1207 | -0.10 | -0.16 | -0.04 | 1.16E-03 | 1195 | -0.10 | -0.16 | -0.04 | 1.78E-03 |
| Free cholesterol to total lipids ratio in small HDL (%) | 1207 | 0.06 | 0.00 | 0.13 | 0.061 | 1207 | 0.10 | 0.03 | 0.17 | 0.005 | 1195 | 0.10 | 0.03 | 0.17 | 0.005 |
| Triglycerides to total lipids ratio in small HDL (%) | 1207 | 0.06 | 0.00 | 0.13 | 0.067 | 1207 | 0.02 | -0.04 | 0.09 | 0.526 | 1195 | 0.02 | -0.05 | 0.08 | 0.622 |
| Mean diameter for VLDL particles (nm) | 1207 | 0.09 | 0.02 | 0.16 | 0.010 | 1207 | 0.05 | -0.02 | 0.12 | 0.179 | 1195 | 0.04 | -0.02 | 0.11 | 0.212 |
| Mean diameter for LDL particles (nm) | 1207 | -0.01 | -0.07 | 0.06 | 0.873 | 1207 | 0.02 | -0.05 | 0.08 | 0.597 | 1195 | 0.01 | -0.05 | 0.08 | 0.661 |
| Mean diameter for HDL particles (nm) | 1207 | -0.04 | -0.10 | 0.02 | 0.201 | 1207 | 0.00 | -0.06 | 0.07 | 0.936 | 1195 | 0.00 | -0.06 | 0.07 | 0.905 |
| Serum total cholesterol (mmol/l) | 1207 | -0.03 | -0.09 | 0.03 | 0.394 | 1207 | -0.04 | -0.10 | 0.03 | 0.252 | 1195 | -0.03 | -0.10 | 0.03 | 0.301 |
| Total cholesterol in VLDL (mmol/l) | 1207 | 0.06 | -0.01 | 0.13 | 0.089 | 1207 | 0.02 | -0.05 | 0.09 | 0.633 | 1195 | 0.02 | -0.05 | 0.08 | 0.626 |
| Remnant cholesterol (non-HDL, non-LDL -cholesterol) (mmol/l) | 1207 | 0.03 | -0.04 | 0.10 | 0.371 | 1207 | 0.00 | -0.07 | 0.07 | 0.931 | 1195 | 0.00 | -0.07 | 0.07 | 0.972 |
| Total cholesterol in LDL (mmol/l) | 1207 | -0.02 | -0.09 | 0.04 | 0.445 | 1207 | -0.04 | -0.11 | 0.02 | 0.200 | 1195 | -0.04 | -0.10 | 0.03 | 0.245 |
| Total cholesterol in HDL (mmol/l) | 1207 | -0.07 | -0.14 | -0.01 | 0.021 | 1207 | -0.04 | -0.10 | 0.03 | 0.235 | 1195 | -0.04 | -0.10 | 0.03 | 0.252 |
| Total cholesterol in HDL2 (mmol/l) | 1207 | -0.08 | -0.15 | -0.02 | 0.011 | 1207 | -0.05 | -0.11 | 0.02 | 0.177 | 1195 | -0.04 | -0.11 | 0.02 | 0.188 |
| Total cholesterol in HDL3 (mmol/l) | 1207 | -0.05 | -0.11 | 0.01 | 0.083 | 1207 | -0.03 | -0.09 | 0.04 | 0.417 | 1195 | -0.02 | -0.09 | 0.04 | 0.443 |
| Esterified cholesterol (mmol/l) | 1207 | -0.03 | -0.09 | 0.03 | 0.325 | 1207 | -0.04 | -0.10 | 0.02 | 0.210 | 1195 | -0.04 | -0.10 | 0.03 | 0.255 |
| Free cholesterol (mmol/l) | 1207 | -0.02 | -0.08 | 0.05 | 0.606 | 1207 | -0.03 | -0.09 | 0.04 | 0.400 | 1195 | -0.02 | -0.09 | 0.04 | 0.456 |
| Serum total triglycerides (mmol/l) | 1207 | 0.07 | 0.00 | 0.14 | 0.037 | 1207 | 0.03 | -0.04 | 0.10 | 0.431 | 1195 | 0.02 | -0.04 | 0.09 | 0.469 |
| Triglycerides in VLDL (mmol/l) | 1207 | 0.09 | 0.02 | 0.16 | 0.010 | 1207 | 0.04 | -0.03 | 0.11 | 0.215 | 1195 | 0.04 | -0.03 | 0.11 | 0.232 |
| Triglycerides in LDL (mmol/l) | 1207 | -0.04 | -0.10 | 0.02 | 0.212 | 1207 | -0.05 | -0.12 | 0.01 | 0.094 | 1195 | -0.05 | -0.12 | 0.01 | 0.098 |
| Triglycerides in HDL (mmol/l) | 1207 | 0.03 | -0.04 | 0.09 | 0.399 | 1207 | 0.00 | -0.07 | 0.07 | 0.988 | 1195 | 0.00 | -0.07 | 0.06 | 0.944 |
| Diacylglycerol (mmol/l) | 1166 | 0.05 | -0.02 | 0.11 | 0.167 | 1166 | 0.03 | -0.04 | 0.10 | 0.352 | 1155 | 0.03 | -0.04 | 0.10 | 0.336 |
| Ratio of diacylglycerol to triglycerides | 1166 | 0.00 | -0.07 | 0.06 | 0.902 | 1166 | 0.00 | -0.07 | 0.07 | 1.000 | 1155 | 0.00 | -0.07 | 0.07 | 0.968 |
| Total phosphoglycerides (mmol/l) | 1207 | -0.04 | -0.10 | 0.02 | 0.179 | 1207 | -0.04 | -0.10 | 0.02 | 0.225 | 1195 | -0.04 | -0.10 | 0.02 | 0.213 |
| Ratio of triglycerides to phosphoglycerides | 1207 | 0.10 | 0.04 | 0.17 | 2.65E-03 | 1207 | 0.07 | 0.00 | 0.13 | 0.053 | 1195 | 0.06 | 0.00 | 0.13 | 0.057 |
| Phosphatidylcholine and other cholines (mmol/l) | 1185 | -0.01 | -0.07 | 0.05 | 0.692 | 1185 | 0.00 | -0.07 | 0.06 | 0.900 | 1173 | -0.01 | -0.07 | 0.06 | 0.837 |
| Total cholines (mmol/l) | 1203 | -0.03 | -0.09 | 0.03 | 0.262 | 1203 | -0.03 | -0.09 | 0.04 | 0.389 | 1191 | -0.03 | -0.09 | 0.03 | 0.349 |

**S4 Table** Associations of current sedentary time (SED at age 15y) with metabolic traits at age 15y in ALSPAC

## SED at age 15y (per SD (66 min/day) higher)

| | *Adj. for age, sex, ethnicity, maternal education, smoking, alcohol, wear time, wear month* | | | | | *Additionally adj. for MVPA at age 15y* | | | | | *Additionally adj. for FMI at age 15y* | | | | |
|---|---|---|---|---|---|---|---|---|---|---|---|---|---|---|---|
| **Standardised outcome at age 15y** | **N** | **Beta** | **LCL** | **UCL** | **P-value** | **N** | **Beta** | **LCL** | **UCL** | **P-value** | **N** | **Beta** | **LCL** | **UCL** | **P-value** |
| Apolipoprotein A-I (g/l) | 1207 | -0.06 | -0.12 | 0.00 | 0.059 | 1207 | -0.04 | -0.10 | 0.02 | 0.229 | 1195 | -0.04 | -0.10 | 0.03 | 0.252 |
| Apolipoprotein B (g/l) | 1207 | 0.04 | -0.03 | 0.11 | 0.259 | 1207 | 0.00 | -0.07 | 0.07 | 0.985 | 1195 | 0.00 | -0.07 | 0.07 | 0.997 |
| Ratio of apolipoprotein B to apolipoprotein A-I | 1207 | 0.07 | 0.00 | 0.14 | 0.051 | 1207 | 0.02 | -0.05 | 0.09 | 0.578 | 1195 | 0.02 | -0.05 | 0.09 | 0.573 |
| Total fatty acids (mmol/l) | 1207 | 0.01 | -0.05 | 0.07 | 0.709 | 1207 | -0.01 | -0.07 | 0.06 | 0.818 | 1195 | -0.01 | -0.07 | 0.06 | 0.818 |
| Estimated description of fatty acid chain length, not actual carbon number | 1201 | 0.02 | -0.05 | 0.09 | 0.506 | 1201 | 0.02 | -0.05 | 0.09 | 0.592 | 1189 | 0.01 | -0.06 | 0.08 | 0.776 |
| Estimated degree of unsaturation | 1206 | 0.00 | -0.07 | 0.06 | 0.919 | 1206 | 0.01 | -0.06 | 0.08 | 0.858 | 1194 | 0.00 | -0.07 | 0.07 | 0.922 |
| 22:6, docosahexaenoic acid (mmol/l) | 1207 | -0.04 | -0.10 | 0.02 | 0.221 | 1207 | -0.04 | -0.10 | 0.02 | 0.222 | 1195 | -0.04 | -0.10 | 0.02 | 0.219 |
| 18:2, linoleic acid (mmol/l) | 1203 | -0.01 | -0.07 | 0.05 | 0.702 | 1203 | -0.02 | -0.09 | 0.04 | 0.534 | 1191 | -0.02 | -0.09 | 0.04 | 0.515 |
| Conjugated linoleic acid (mmol/l) | 1207 | 0.07 | -0.01 | 0.14 | 0.070 | 1207 | 0.06 | -0.02 | 0.14 | 0.122 | 1195 | 0.06 | -0.02 | 0.14 | 0.135 |
| Omega-3 fatty acids (mmol/l) | 1205 | -0.02 | -0.09 | 0.04 | 0.432 | 1205 | -0.03 | -0.10 | 0.03 | 0.327 | 1193 | -0.03 | -0.10 | 0.03 | 0.289 |
| Omega-6 fatty acids (mmol/l) | 1204 | 0.00 | -0.07 | 0.06 | 0.878 | 1204 | -0.02 | -0.08 | 0.05 | 0.633 | 1192 | -0.02 | -0.08 | 0.05 | 0.637 |
| Polyunsaturated fatty acids (mmol/l) | 1202 | -0.01 | -0.07 | 0.05 | 0.757 | 1202 | -0.02 | -0.08 | 0.04 | 0.557 | 1190 | -0.02 | -0.08 | 0.04 | 0.553 |
| Monounsaturated fatty acids; 16:1, 18:1 (mmol/l) | 1204 | 0.03 | -0.03 | 0.09 | 0.385 | 1204 | 0.00 | -0.07 | 0.06 | 0.895 | 1192 | 0.00 | -0.07 | 0.06 | 0.881 |
| Saturated fatty acids (mmol/l) | 1202 | 0.01 | -0.05 | 0.08 | 0.650 | 1202 | 0.00 | -0.06 | 0.07 | 0.916 | 1190 | 0.00 | -0.06 | 0.07 | 0.896 |
| Ratio of 22:6 docosahexaenoic acid to total fatty acids (%) | 1207 | -0.05 | -0.11 | 0.02 | 0.151 | 1207 | -0.04 | -0.10 | 0.03 | 0.263 | 1195 | -0.04 | -0.10 | 0.03 | 0.263 |
| Ratio of 18:2 linoleic acid to total fatty acids (%) | 1203 | -0.02 | -0.09 | 0.04 | 0.492 | 1203 | -0.01 | -0.08 | 0.06 | 0.870 | 1191 | -0.01 | -0.08 | 0.06 | 0.831 |
| Ratio of conjugated linoleic acid to total fatty acids (%) | 1207 | 0.07 | -0.01 | 0.14 | 0.084 | 1207 | 0.06 | -0.02 | 0.14 | 0.130 | 1195 | 0.06 | -0.02 | 0.14 | 0.141 |
| Ratio of omega-3 fatty acids to total fatty acids (%) | 1205 | -0.03 | -0.09 | 0.03 | 0.309 | 1205 | -0.03 | -0.09 | 0.04 | 0.436 | 1193 | -0.03 | -0.10 | 0.04 | 0.382 |
| Ratio of omega-6 fatty acids to total fatty acids (%) | 1204 | -0.03 | -0.09 | 0.04 | 0.440 | 1204 | -0.01 | -0.07 | 0.06 | 0.877 | 1192 | 0.00 | -0.07 | 0.06 | 0.889 |
| Ratio of polyunsaturated fatty acids to total fatty acids (%) | 1202 | -0.03 | -0.10 | 0.03 | 0.325 | 1202 | -0.01 | -0.08 | 0.06 | 0.727 | 1190 | -0.01 | -0.08 | 0.06 | 0.720 |
| Ratio of monounsaturated fatty acids to total fatty acids (%) | 1204 | 0.03 | -0.04 | 0.09 | 0.430 | 1204 | -0.01 | -0.07 | 0.06 | 0.859 | 1192 | -0.01 | -0.07 | 0.06 | 0.823 |
| Ratio of saturated fatty acids to total fatty acids (%) | 1202 | 0.00 | -0.06 | 0.07 | 0.974 | 1202 | 0.02 | -0.05 | 0.08 | 0.605 | 1190 | 0.02 | -0.05 | 0.09 | 0.562 |
| Insulin (mu/l) | 1253 | 0.03 | -0.01 | 0.07 | 0.118 | 1253 | 0.01 | -0.03 | 0.05 | 0.750 | 1241 | 0.01 | -0.03 | 0.05 | 0.653 |
| Glucose (mmol/l) | 1204 | 0.03 | -0.03 | 0.09 | 0.307 | 1204 | 0.02 | -0.04 | 0.09 | 0.507 | 1192 | 0.03 | -0.04 | 0.09 | 0.403 |
| Lactate (mmol/l) | 1205 | 0.06 | 0.00 | 0.13 | 0.065 | 1205 | 0.06 | -0.01 | 0.13 | 0.079 | 1193 | 0.06 | -0.01 | 0.14 | 0.076 |
| Pyruvate (mmol/l) | 1203 | 0.08 | 0.01 | 0.15 | 0.022 | 1203 | 0.07 | 0.00 | 0.14 | 0.042 | 1191 | 0.07 | 0.00 | 0.14 | 0.039 |
| Citrate (mmol/l) | 1198 | -0.10 | -0.17 | -0.03 | 0.005 | 1198 | -0.09 | -0.16 | -0.02 | 0.016 | 1186 | -0.08 | -0.15 | -0.01 | 0.019 |
| Alanine (mmol/l) | 1207 | 0.12 | 0.05 | 0.18 | 7.32E-04 | 1207 | 0.13 | 0.06 | 0.20 | 4.77E-04 | 1195 | 0.13 | 0.06 | 0.20 | 4.21E-04 |
| Glutamine (mmol/l) | 1207 | -0.04 | -0.10 | 0.02 | 0.226 | 1207 | -0.03 | -0.09 | 0.04 | 0.429 | 1195 | -0.02 | -0.09 | 0.04 | 0.484 |
| Histidine (mmol/l) | 1141 | 0.06 | -0.01 | 0.13 | 0.074 | 1141 | 0.08 | 0.01 | 0.15 | 0.025 | 1129 | 0.08 | 0.01 | 0.14 | 0.034 |
| Isoleucine (mmol/l) | 1207 | 0.08 | 0.01 | 0.14 | 0.019 | 1207 | 0.07 | 0.00 | 0.13 | 0.046 | 1195 | 0.06 | 0.00 | 0.13 | 0.051 |
| Leucine (mmol/l) | 1207 | 0.01 | -0.05 | 0.06 | 0.801 | 1207 | 0.01 | -0.04 | 0.07 | 0.654 | 1195 | 0.01 | -0.05 | 0.07 | 0.672 |
| Valine (mmol/l) | 1207 | 0.04 | -0.02 | 0.10 | 0.178 | 1207 | 0.03 | -0.03 | 0.10 | 0.292 | 1195 | 0.04 | -0.03 | 0.10 | 0.277 |
| Phenylalanine (mmol/l) | 1206 | -0.07 | -0.14 | 0.01 | 0.072 | 1206 | -0.06 | -0.14 | 0.01 | 0.111 | 1194 | -0.06 | -0.14 | 0.01 | 0.105 |
| Tyrosine (mmol/l) | 1200 | -0.07 | -0.14 | 0.00 | 0.044 | 1200 | -0.07 | -0.14 | 0.00 | 0.043 | 1188 | -0.07 | -0.14 | 0.00 | 0.036 |
| Acetate (mmol/l) | 1206 | -0.02 | -0.08 | 0.05 | 0.629 | 1206 | 0.00 | -0.07 | 0.07 | 0.990 | 1194 | 0.00 | -0.07 | 0.07 | 0.961 |
| Acetoacetate (mmol/l) | 1207 | -0.05 | -0.13 | 0.02 | 0.156 | 1207 | -0.06 | -0.13 | 0.02 | 0.136 | 1195 | -0.05 | -0.13 | 0.02 | 0.149 |
| 3-hydroxybutyrate (mmol/l) | 1206 | -0.08 | -0.15 | -0.01 | 0.033 | 1206 | -0.10 | -0.17 | -0.02 | 0.010 | 1194 | -0.10 | -0.17 | -0.02 | 0.013 |
| Creatinine (mmol/l) | 1206 | 0.12 | 0.05 | 0.18 | 5.14E-04 | 1206 | 0.11 | 0.04 | 0.18 | 1.74E-03 | 1194 | 0.11 | 0.05 | 0.18 | 1.19E-03 |
| Albumin  (signal area) | 1207 | 0.07 | 0.00 | 0.14 | 0.043 | 1207 | 0.05 | -0.02 | 0.12 | 0.201 | 1195 | 0.05 | -0.02 | 0.12 | 0.186 |
| Glycoprotein acetyls, mainly a1-acid glycoprotein (mmol/l) | 1206 | 0.03 | -0.03 | 0.10 | 0.314 | 1206 | -0.01 | -0.08 | 0.06 | 0.699 | 1194 | -0.01 | -0.08 | 0.05 | 0.705 |
| C-reactive protein (mg/l) | 1255 | -0.01 | -0.09 | 0.07 | 0.873 | 1255 | -0.01 | -0.09 | 0.07 | 0.761 | 1243 | -0.01 | -0.10 | 0.07 | 0.749 |

## SED at age 15y (per SD (66 min/day) higher)

## Complete case sample

*Adj. for age, sex, ethnicity, maternal education,* *Additionally adj. for MVPA at age 15y* *Additionally adj. for FMI at age 15y*

**S4 Table** Associations of current sedentary time (SED at age 15y) with metabolic traits at age 15y in ALSPAC

## SED at age 15y (per SD (66 min/day) higher)

*Adj. for age, sex, ethnicity, maternal education, smoking, alcohol, wear time, wear month* | *Additionally adj. for MVPA at age 15y* | *Additionally adj. for FMI at age 15y*

Standardised outcome at age 15y N Beta LCL UCL P-value N Beta LCL UCL P-value N Beta LCL UCL P-value
*smoking, alcohol, wear time, wear month*

| Standardised outcome at age 15y | N | Beta | LCL | UCL | P-value | N | Beta | LCL | UCL | P-value | N | Beta | LCL | UCL | P-value |
|---|---|---|---|---|---|---|---|---|---|---|---|---|---|---|---|
| Systolic blood pressure (mmHg) | 755 | 0.04 | -0.05 | 0.12 | 0.400 | 755 | 0.04 | -0.04 | 0.12 | 0.349 | 755 | 0.03 | -0.05 | 0.11 | 0.478 |
| Diastolic blood pressure (mmHg) | 755 | 0.02 | -0.06 | 0.10 | 0.645 | 755 | 0.03 | -0.05 | 0.12 | 0.464 | 755 | 0.03 | -0.06 | 0.12 | 0.499 |
| Concentration of chylomicrons and extremely large VLDL particles (mol/l) | 755 | 0.05 | -0.04 | 0.13 | 0.271 | 755 | 0.01 | -0.08 | 0.09 | 0.853 | 755 | -0.01 | -0.09 | 0.07 | 0.866 |
| Total lipids in chylomicrons and extremely large VLDL (mmol/l) | 755 | 0.05 | -0.04 | 0.13 | 0.261 | 755 | 0.01 | -0.08 | 0.09 | 0.835 | 755 | -0.01 | -0.09 | 0.07 | 0.896 |
| Phospholipids in chylomicrons and extremely large VLDL (mmol/l) | 755 | 0.05 | -0.04 | 0.13 | 0.284 | 755 | 0.01 | -0.08 | 0.09 | 0.898 | 755 | -0.01 | -0.09 | 0.07 | 0.840 |
| Total cholesterol in chylomicrons and extremely large VLDL (mmol/l) | 755 | 0.05 | -0.04 | 0.13 | 0.291 | 755 | 0.01 | -0.07 | 0.10 | 0.766 | 755 | 0.00 | -0.08 | 0.08 | 0.963 |
| Cholesterol esters in chylomicrons and extremely large VLDL (mmol/l) | 755 | 0.04 | -0.04 | 0.13 | 0.309 | 755 | 0.02 | -0.07 | 0.11 | 0.652 | 755 | 0.00 | -0.08 | 0.09 | 0.913 |
| Free cholesterol in chylomicrons and extremely large VLDL (mmol/l) | 755 | 0.04 | -0.04 | 0.13 | 0.299 | 755 | 0.01 | -0.08 | 0.09 | 0.907 | 755 | -0.01 | -0.09 | 0.07 | 0.832 |
| Triglycerides in chylomicrons and extremely large VLDL (mmol/l) | 755 | 0.05 | -0.04 | 0.13 | 0.253 | 755 | 0.01 | -0.08 | 0.09 | 0.841 | 755 | -0.01 | -0.09 | 0.07 | 0.891 |
| Concentration of very large VLDL particles (mol/l) | 755 | 0.06 | -0.03 | 0.14 | 0.181 | 755 | 0.02 | -0.06 | 0.10 | 0.644 | 755 | 0.01 | -0.07 | 0.08 | 0.893 |
| Total lipids in very large VLDL (mmol/l) | 755 | 0.06 | -0.03 | 0.14 | 0.189 | 755 | 0.02 | -0.06 | 0.10 | 0.656 | 755 | 0.00 | -0.07 | 0.08 | 0.901 |
| Phospholipids in very large VLDL (mmol/l) | 755 | 0.05 | -0.03 | 0.13 | 0.238 | 755 | 0.01 | -0.07 | 0.10 | 0.759 | 755 | 0.00 | -0.08 | 0.08 | 0.991 |
| Total cholesterol in very large VLDL (mmol/l) | 755 | 0.05 | -0.04 | 0.13 | 0.253 | 755 | 0.01 | -0.07 | 0.10 | 0.757 | 755 | 0.00 | -0.08 | 0.08 | 0.967 |
| Cholesterol esters in very large VLDL (mmol/l) | 755 | 0.05 | -0.03 | 0.14 | 0.224 | 755 | 0.02 | -0.07 | 0.10 | 0.668 | 755 | 0.00 | -0.08 | 0.08 | 0.944 |
| Free cholesterol in very large VLDL (mmol/l) | 755 | 0.04 | -0.04 | 0.13 | 0.294 | 755 | 0.01 | -0.08 | 0.09 | 0.861 | 755 | -0.01 | -0.09 | 0.07 | 0.872 |
| Triglycerides in very large VLDL (mmol/l) | 755 | 0.06 | -0.02 | 0.14 | 0.164 | 755 | 0.02 | -0.06 | 0.11 | 0.601 | 755 | 0.01 | -0.07 | 0.09 | 0.833 |
| Concentration of large VLDL particles (mol/l) | 755 | 0.06 | -0.02 | 0.14 | 0.148 | 755 | 0.03 | -0.06 | 0.11 | 0.540 | 755 | 0.01 | -0.07 | 0.09 | 0.766 |
| Total lipids in large VLDL (mmol/l) | 755 | 0.06 | -0.02 | 0.14 | 0.162 | 755 | 0.03 | -0.06 | 0.11 | 0.561 | 755 | 0.01 | -0.07 | 0.09 | 0.790 |
| Phospholipids in large VLDL (mmol/l) | 755 | 0.06 | -0.03 | 0.14 | 0.183 | 755 | 0.02 | -0.06 | 0.11 | 0.613 | 755 | 0.01 | -0.07 | 0.09 | 0.849 |
| Total cholesterol in large VLDL (mmol/l) | 755 | 0.05 | -0.03 | 0.14 | 0.211 | 755 | 0.02 | -0.06 | 0.11 | 0.615 | 755 | 0.01 | -0.07 | 0.09 | 0.859 |
| Cholesterol esters in large VLDL (mmol/l) | 755 | 0.05 | -0.04 | 0.14 | 0.252 | 755 | 0.02 | -0.07 | 0.11 | 0.628 | 755 | 0.01 | -0.08 | 0.09 | 0.881 |
| Free cholesterol in large VLDL (mmol/l) | 755 | 0.06 | -0.03 | 0.14 | 0.180 | 755 | 0.02 | -0.06 | 0.11 | 0.606 | 755 | 0.01 | -0.07 | 0.09 | 0.840 |
| Triglycerides in large VLDL (mmol/l) | 755 | 0.06 | -0.02 | 0.15 | 0.142 | 755 | 0.03 | -0.06 | 0.11 | 0.528 | 755 | 0.01 | -0.07 | 0.09 | 0.747 |
| Concentration of medium VLDL particles (mol/l) | 755 | 0.06 | -0.02 | 0.15 | 0.155 | 755 | 0.03 | -0.06 | 0.11 | 0.550 | 755 | 0.01 | -0.07 | 0.09 | 0.788 |
| Total lipids in medium VLDL (mmol/l) | 755 | 0.06 | -0.03 | 0.14 | 0.175 | 755 | 0.03 | -0.06 | 0.11 | 0.568 | 755 | 0.01 | -0.07 | 0.09 | 0.810 |
| Phospholipids in medium VLDL (mmol/l) | 755 | 0.06 | -0.03 | 0.14 | 0.195 | 755 | 0.02 | -0.06 | 0.11 | 0.612 | 755 | 0.01 | -0.07 | 0.09 | 0.852 |
| Total cholesterol in medium VLDL (mmol/l) | 755 | 0.05 | -0.04 | 0.13 | 0.278 | 755 | 0.02 | -0.07 | 0.11 | 0.632 | 755 | 0.01 | -0.08 | 0.09 | 0.877 |
| Cholesterol esters in medium VLDL (mmol/l) | 755 | 0.04 | -0.05 | 0.13 | 0.373 | 755 | 0.02 | -0.07 | 0.11 | 0.651 | 755 | 0.01 | -0.08 | 0.09 | 0.895 |
| Free cholesterol in medium VLDL (mmol/l) | 755 | 0.05 | -0.03 | 0.14 | 0.206 | 755 | 0.02 | -0.07 | 0.11 | 0.628 | 755 | 0.01 | -0.07 | 0.09 | 0.862 |
| Triglycerides in medium VLDL (mmol/l) | 755 | 0.06 | -0.02 | 0.15 | 0.140 | 755 | 0.03 | -0.06 | 0.11 | 0.531 | 755 | 0.01 | -0.07 | 0.09 | 0.767 |
| Concentration of small VLDL particles (mol/l) | 755 | 0.04 | -0.04 | 0.13 | 0.342 | 755 | 0.01 | -0.08 | 0.09 | 0.864 | 755 | -0.01 | -0.09 | 0.08 | 0.866 |
| Total lipids in small VLDL (mmol/l) | 755 | 0.03 | -0.05 | 0.12 | 0.432 | 755 | 0.00 | -0.09 | 0.09 | 0.986 | 755 | -0.01 | -0.10 | 0.07 | 0.734 |
| Phospholipids in small VLDL (mmol/l) | 755 | 0.02 | -0.06 | 0.11 | 0.557 | 755 | 0.00 | -0.09 | 0.08 | 0.915 | 755 | -0.02 | -0.10 | 0.06 | 0.638 |
| Total cholesterol in small VLDL (mmol/l) | 755 | 0.02 | -0.07 | 0.10 | 0.673 | 755 | -0.01 | -0.10 | 0.08 | 0.813 | 755 | -0.03 | -0.11 | 0.06 | 0.553 |
| Cholesterol esters in small VLDL (mmol/l) | 755 | 0.01 | -0.07 | 0.10 | 0.784 | 755 | -0.02 | -0.10 | 0.07 | 0.723 | 755 | -0.03 | -0.12 | 0.06 | 0.478 |
| Free cholesterol in small VLDL (mmol/l) | 755 | 0.03 | -0.06 | 0.11 | 0.507 | 755 | 0.00 | -0.09 | 0.09 | 0.994 | 755 | -0.01 | -0.10 | 0.07 | 0.751 |
| Triglycerides in small VLDL (mmol/l) | 755 | 0.05 | -0.04 | 0.13 | 0.278 | 755 | 0.01 | -0.07 | 0.10 | 0.775 | 755 | 0.00 | -0.08 | 0.08 | 0.983 |
| Concentration of very small VLDL particles (mol/l) | 755 | 0.00 | -0.08 | 0.08 | 0.996 | 755 | -0.01 | -0.10 | 0.07 | 0.774 | 755 | -0.02 | -0.10 | 0.06 | 0.645 |
| Total lipids in very small VLDL (mmol/l) | 755 | 0.01 | -0.08 | 0.09 | 0.864 | 755 | -0.01 | -0.10 | 0.07 | 0.799 | 755 | -0.02 | -0.11 | 0.06 | 0.632 |
| Phospholipids in very small VLDL (mmol/l) | 755 | -0.02 | -0.10 | 0.06 | 0.626 | 755 | -0.03 | -0.12 | 0.05 | 0.446 | 755 | -0.04 | -0.12 | 0.05 | 0.386 |
| Total cholesterol in very small VLDL (mmol/l) | 755 | 0.02 | -0.06 | 0.10 | 0.649 | 755 | 0.01 | -0.08 | 0.09 | 0.891 | 755 | -0.01 | -0.09 | 0.08 | 0.905 |
| Cholesterol esters in very small VLDL (mmol/l) | 755 | 0.02 | -0.06 | 0.10 | 0.645 | 755 | 0.00 | -0.09 | 0.09 | 0.996 | 755 | -0.01 | -0.10 | 0.07 | 0.770 |
| Free cholesterol in very small VLDL (mmol/l) | 755 | 0.02 | -0.06 | 0.10 | 0.687 | 755 | 0.02 | -0.07 | 0.10 | 0.675 | 755 | 0.01 | -0.07 | 0.10 | 0.780 |
| Triglycerides in very small VLDL (mmol/l) | 755 | 0.01 | -0.07 | 0.10 | 0.746 | 755 | -0.01 | -0.10 | 0.07 | 0.737 | 755 | -0.02 | -0.11 | 0.06 | 0.592 |
| Concentration of IDL particles (mol/l) | 755 | -0.03 | -0.11 | 0.05 | 0.461 | 755 | -0.04 | -0.13 | 0.04 | 0.323 | 755 | -0.04 | -0.13 | 0.04 | 0.312 |

**S4 Table** Associations of current sedentary time (SED at age 15y) with metabolic traits at age 15y in ALSPAC

| | **SED at age 15y (per SD (66 min/day) higher)** | | | | | | | | | | | | | | |
|---|---|---|---|---|---|---|---|---|---|---|---|---|---|---|---|
| | *Adj. for age, sex, ethnicity, maternal education, smoking, alcohol, wear time, wear month* | | | | | *Additionally adj. for MVPA at age 15y* | | | | | *Additionally adj. for FMI at age 15y* | | | | |
| **Standardised outcome at age 15y** | **N** | **Beta** | **LCL** | **UCL** | **P-value** | **N** | **Beta** | **LCL** | **UCL** | **P-value** | **N** | **Beta** | **LCL** | **UCL** | **P-value** |
| Total lipids in IDL (mmol/l) | 755 | -0.03 | -0.11 | 0.05 | 0.520 | 755 | -0.04 | -0.12 | 0.05 | 0.388 | 755 | -0.04 | -0.12 | 0.04 | 0.358 |
| Phospholipids in IDL (mmol/l) | 755 | -0.04 | -0.12 | 0.04 | 0.289 | 755 | -0.05 | -0.14 | 0.03 | 0.206 | 755 | -0.05 | -0.14 | 0.03 | 0.202 |
| Total cholesterol in IDL (mmol/l) | 755 | -0.02 | -0.10 | 0.06 | 0.676 | 755 | -0.03 | -0.11 | 0.06 | 0.536 | 755 | -0.03 | -0.12 | 0.05 | 0.478 |
| Cholesterol esters in IDL (mmol/l) | 755 | -0.01 | -0.09 | 0.07 | 0.836 | 755 | -0.02 | -0.10 | 0.07 | 0.651 | 755 | -0.03 | -0.11 | 0.06 | 0.561 |
| Free cholesterol in IDL (mmol/l) | 755 | -0.04 | -0.12 | 0.04 | 0.365 | 755 | -0.04 | -0.12 | 0.04 | 0.319 | 755 | -0.04 | -0.12 | 0.04 | 0.322 |
| Triglycerides in IDL (mmol/l) | 755 | -0.03 | -0.11 | 0.05 | 0.431 | 755 | -0.04 | -0.12 | 0.04 | 0.277 | 755 | -0.04 | -0.12 | 0.04 | 0.303 |
| Concentration of large LDL particles (mol/l) | 755 | -0.05 | -0.13 | 0.03 | 0.248 | 755 | -0.06 | -0.14 | 0.02 | 0.149 | 755 | -0.06 | -0.15 | 0.02 | 0.139 |
| Total lipids in large LDL (mmol/l) | 755 | -0.04 | -0.12 | 0.04 | 0.304 | 755 | -0.05 | -0.14 | 0.03 | 0.203 | 755 | -0.06 | -0.14 | 0.03 | 0.186 |
| Phospholipids in large LDL (mmol/l) | 755 | -0.04 | -0.12 | 0.04 | 0.292 | 755 | -0.06 | -0.14 | 0.03 | 0.185 | 755 | -0.06 | -0.14 | 0.02 | 0.161 |
| Total cholesterol in large LDL (mmol/l) | 755 | -0.04 | -0.12 | 0.04 | 0.342 | 755 | -0.05 | -0.13 | 0.03 | 0.239 | 755 | -0.05 | -0.14 | 0.03 | 0.216 |
| Cholesterol esters in large LDL (mmol/l) | 755 | -0.04 | -0.12 | 0.04 | 0.367 | 755 | -0.05 | -0.13 | 0.03 | 0.247 | 755 | -0.05 | -0.14 | 0.03 | 0.218 |
| Free cholesterol in large LDL (mmol/l) | 755 | -0.04 | -0.13 | 0.04 | 0.277 | 755 | -0.05 | -0.13 | 0.03 | 0.220 | 755 | -0.05 | -0.14 | 0.03 | 0.215 |
| Triglycerides in large LDL (mmol/l) | 755 | -0.05 | -0.13 | 0.03 | 0.212 | 755 | -0.06 | -0.14 | 0.02 | 0.124 | 755 | -0.06 | -0.14 | 0.02 | 0.154 |
| Concentration of medium LDL particles (mol/l) | 755 | -0.05 | -0.13 | 0.03 | 0.239 | 755 | -0.07 | -0.15 | 0.02 | 0.116 | 755 | -0.07 | -0.15 | 0.01 | 0.102 |
| Total lipids in medium LDL (mmol/l) | 755 | -0.04 | -0.12 | 0.04 | 0.289 | 755 | -0.06 | -0.14 | 0.02 | 0.165 | 755 | -0.06 | -0.14 | 0.02 | 0.145 |
| Phospholipids in medium LDL (mmol/l) | 755 | -0.03 | -0.11 | 0.05 | 0.420 | 755 | -0.05 | -0.13 | 0.03 | 0.252 | 755 | -0.05 | -0.14 | 0.03 | 0.200 |
| Total cholesterol in medium LDL (mmol/l) | 755 | -0.04 | -0.12 | 0.04 | 0.324 | 755 | -0.06 | -0.14 | 0.03 | 0.193 | 755 | -0.06 | -0.14 | 0.03 | 0.170 |
| Cholesterol esters in medium LDL (mmol/l) | 755 | -0.04 | -0.12 | 0.04 | 0.305 | 755 | -0.06 | -0.14 | 0.03 | 0.173 | 755 | -0.06 | -0.15 | 0.02 | 0.152 |
| Free cholesterol in medium LDL (mmol/l) | 755 | -0.03 | -0.11 | 0.05 | 0.434 | 755 | -0.04 | -0.13 | 0.04 | 0.321 | 755 | -0.05 | -0.13 | 0.04 | 0.289 |
| Triglycerides in medium LDL (mmol/l) | 755 | -0.07 | -0.15 | 0.01 | 0.073 | 755 | -0.09 | -0.17 | -0.01 | 0.036 | 755 | -0.08 | -0.16 | 0.00 | 0.048 |
| Concentration of small LDL particles (mol/l) | 755 | -0.04 | -0.13 | 0.04 | 0.305 | 755 | -0.06 | -0.14 | 0.02 | 0.153 | 755 | -0.06 | -0.15 | 0.02 | 0.136 |
| Total lipids in small LDL (mmol/l) | 755 | -0.04 | -0.12 | 0.04 | 0.322 | 755 | -0.06 | -0.14 | 0.03 | 0.182 | 755 | -0.06 | -0.14 | 0.02 | 0.158 |
| Phospholipids in small LDL (mmol/l) | 755 | -0.03 | -0.11 | 0.05 | 0.474 | 755 | -0.04 | -0.13 | 0.04 | 0.299 | 755 | -0.05 | -0.13 | 0.03 | 0.255 |
| Total cholesterol in small LDL (mmol/l) | 755 | -0.04 | -0.12 | 0.04 | 0.311 | 755 | -0.06 | -0.14 | 0.03 | 0.185 | 755 | -0.06 | -0.14 | 0.02 | 0.162 |
| Cholesterol esters in small LDL (mmol/l) | 755 | -0.05 | -0.13 | 0.04 | 0.263 | 755 | -0.06 | -0.15 | 0.02 | 0.146 | 755 | -0.07 | -0.15 | 0.02 | 0.129 |
| Free cholesterol in small LDL (mmol/l) | 755 | -0.02 | -0.10 | 0.06 | 0.654 | 755 | -0.03 | -0.11 | 0.06 | 0.530 | 755 | -0.03 | -0.11 | 0.05 | 0.467 |
| Triglycerides in small LDL (mmol/l) | 755 | -0.05 | -0.13 | 0.03 | 0.267 | 755 | -0.07 | -0.15 | 0.01 | 0.095 | 755 | -0.07 | -0.15 | 0.01 | 0.089 |
| Concentration of very large HDL particles (mol/l) | 755 | -0.04 | -0.13 | 0.04 | 0.298 | 755 | -0.01 | -0.10 | 0.07 | 0.742 | 755 | 0.00 | -0.08 | 0.08 | 0.970 |
| Total lipids in very large HDL (mmol/l) | 755 | -0.04 | -0.12 | 0.04 | 0.342 | 755 | -0.01 | -0.10 | 0.07 | 0.779 | 755 | 0.00 | -0.08 | 0.09 | 0.939 |
| Phospholipids in very large HDL (mmol/l) | 755 | -0.05 | -0.13 | 0.03 | 0.222 | 755 | -0.02 | -0.10 | 0.07 | 0.659 | 755 | 0.00 | -0.08 | 0.08 | 0.960 |
| Total cholesterol in very large HDL (mmol/l) | 755 | -0.03 | -0.12 | 0.06 | 0.496 | 755 | -0.01 | -0.10 | 0.08 | 0.882 | 755 | 0.01 | -0.08 | 0.09 | 0.878 |
| Cholesterol esters in very large HDL (mmol/l) | 755 | -0.03 | -0.11 | 0.06 | 0.553 | 755 | -0.01 | -0.10 | 0.08 | 0.910 | 755 | 0.01 | -0.08 | 0.10 | 0.869 |
| Free cholesterol in very large HDL (mmol/l) | 755 | -0.04 | -0.12 | 0.05 | 0.377 | 755 | -0.01 | -0.10 | 0.08 | 0.816 | 755 | 0.01 | -0.08 | 0.09 | 0.907 |
| Triglycerides in very large HDL (mmol/l) | 755 | 0.03 | -0.06 | 0.11 | 0.515 | 755 | 0.03 | -0.05 | 0.12 | 0.465 | 755 | 0.04 | -0.05 | 0.12 | 0.362 |
| Concentration of large HDL particles (mol/l) | 755 | -0.05 | -0.13 | 0.03 | 0.224 | 755 | -0.02 | -0.10 | 0.07 | 0.720 | 755 | 0.00 | -0.08 | 0.08 | 0.974 |
| Total lipids in large HDL (mmol/l) | 755 | -0.05 | -0.13 | 0.03 | 0.231 | 755 | -0.01 | -0.10 | 0.07 | 0.737 | 755 | 0.00 | -0.08 | 0.08 | 0.948 |
| Phospholipids in large HDL (mmol/l) | 755 | -0.06 | -0.14 | 0.02 | 0.154 | 755 | -0.03 | -0.11 | 0.06 | 0.535 | 755 | -0.01 | -0.09 | 0.07 | 0.801 |
| Total cholesterol in large HDL (mmol/l) | 755 | -0.04 | -0.13 | 0.04 | 0.296 | 755 | -0.01 | -0.09 | 0.08 | 0.892 | 755 | 0.01 | -0.07 | 0.09 | 0.772 |
| Cholesterol esters in large HDL (mmol/l) | 755 | -0.04 | -0.13 | 0.04 | 0.297 | 755 | -0.01 | -0.09 | 0.08 | 0.897 | 755 | 0.01 | -0.07 | 0.09 | 0.765 |
| Free cholesterol in large HDL (mmol/l) | 755 | -0.04 | -0.13 | 0.04 | 0.299 | 755 | -0.01 | -0.09 | 0.08 | 0.875 | 755 | 0.01 | -0.07 | 0.09 | 0.794 |
| Triglycerides in large HDL (mmol/l) | 755 | 0.00 | -0.08 | 0.09 | 0.903 | 755 | 0.02 | -0.07 | 0.10 | 0.653 | 755 | 0.03 | -0.06 | 0.11 | 0.541 |
| Concentration of medium HDL particles (mol/l) | 755 | -0.06 | -0.14 | 0.02 | 0.134 | 755 | -0.04 | -0.12 | 0.04 | 0.299 | 755 | -0.04 | -0.12 | 0.04 | 0.336 |
| Total lipids in medium HDL (mmol/l) | 755 | -0.06 | -0.14 | 0.02 | 0.133 | 755 | -0.04 | -0.12 | 0.04 | 0.310 | 755 | -0.04 | -0.12 | 0.04 | 0.366 |
| Phospholipids in medium HDL (mmol/l) | 755 | -0.07 | -0.15 | 0.01 | 0.088 | 755 | -0.05 | -0.13 | 0.03 | 0.224 | 755 | -0.04 | -0.12 | 0.03 | 0.270 |
| Total cholesterol in medium HDL (mmol/l) | 755 | -0.05 | -0.12 | 0.03 | 0.263 | 755 | -0.02 | -0.11 | 0.06 | 0.568 | 755 | -0.02 | -0.10 | 0.06 | 0.678 |
| Cholesterol esters in medium HDL (mmol/l) | 755 | -0.04 | -0.12 | 0.04 | 0.286 | 755 | -0.02 | -0.10 | 0.06 | 0.616 | 755 | -0.01 | -0.10 | 0.07 | 0.732 |
| Free cholesterol in medium HDL (mmol/l) | 755 | -0.05 | -0.13 | 0.02 | 0.181 | 755 | -0.04 | -0.11 | 0.04 | 0.373 | 755 | -0.03 | -0.11 | 0.05 | 0.446 |
| Triglycerides in medium HDL (mmol/l) | 755 | -0.02 | -0.10 | 0.06 | 0.680 | 755 | -0.04 | -0.12 | 0.04 | 0.355 | 755 | -0.05 | -0.13 | 0.03 | 0.208 |

**S4 Table** Associations of current sedentary time (SED at age 15y) with metabolic traits at age 15y in ALSPAC

## SED at age 15y (per SD (66 min/day) higher)

| | *Adj. for age, sex, ethnicity, maternal education, smoking, alcohol, wear time, wear month* | | | | | *Additionally adj. for MVPA at age 15y* | | | | | *Additionally adj. for FMI at age 15y* | | | | |
|---|---|---|---|---|---|---|---|---|---|---|---|---|---|---|---|
| **Standardised outcome at age 15y** | **N** | **Beta** | **LCL** | **UCL** | **P-value** | **N** | **Beta** | **LCL** | **UCL** | **P-value** | **N** | **Beta** | **LCL** | **UCL** | **P-value** |
| Concentration of small HDL particles (mol/l) | 755 | -0.06 | -0.13 | 0.02 | 0.153 | 755 | -0.07 | -0.15 | 0.01 | 0.079 | 755 | -0.08 | -0.16 | 0.00 | 0.048 |
| Total lipids in small HDL (mmol/l) | 755 | -0.08 | -0.15 | -0.01 | 0.036 | 755 | -0.08 | -0.16 | -0.01 | 0.035 | 755 | -0.09 | -0.16 | -0.01 | 0.027 |
| Phospholipids in small HDL (mmol/l) | 755 | -0.03 | -0.11 | 0.05 | 0.462 | 755 | -0.04 | -0.13 | 0.04 | 0.279 | 755 | -0.05 | -0.13 | 0.03 | 0.216 |
| Total cholesterol in small HDL (mmol/l) | 755 | -0.11 | -0.18 | -0.04 | 2.74E-03 | 755 | -0.09 | -0.17 | -0.02 | 0.012 | 755 | -0.09 | -0.16 | -0.02 | 0.013 |
| Cholesterol esters in small HDL (mmol/l) | 755 | -0.11 | -0.18 | -0.04 | 1.32E-03 | 755 | -0.10 | -0.17 | -0.03 | 0.007 | 755 | -0.10 | -0.17 | -0.03 | 0.008 |
| Free cholesterol in small HDL (mmol/l) | 755 | -0.04 | -0.12 | 0.04 | 0.311 | 755 | -0.03 | -0.12 | 0.05 | 0.428 | 755 | -0.03 | -0.11 | 0.05 | 0.457 |
| Triglycerides in small HDL (mmol/l) | 755 | 0.00 | -0.08 | 0.08 | 0.951 | 755 | -0.04 | -0.12 | 0.05 | 0.389 | 755 | -0.05 | -0.12 | 0.03 | 0.266 |
| Phospholipids to total lipds ratio in chylomicrons and extremely large VLDL (%) | 755 | 0.01 | -0.07 | 0.10 | 0.735 | 755 | -0.01 | -0.09 | 0.07 | 0.781 | 755 | -0.01 | -0.10 | 0.07 | 0.756 |
| Total cholesterol to total lipids ratio in chylomicrons and extremely large VLDL (%) | 755 | -0.06 | -0.14 | 0.03 | 0.192 | 755 | -0.06 | -0.15 | 0.03 | 0.214 | 755 | -0.07 | -0.16 | 0.02 | 0.148 |
| Cholesterol esters to total lipids ratio in chylomicrons and extremely large VLDL (%) | 755 | -0.06 | -0.15 | 0.02 | 0.138 | 755 | -0.05 | -0.14 | 0.03 | 0.230 | 755 | -0.06 | -0.15 | 0.03 | 0.166 |
| Free cholesterol to total lipids ratio in chylomicrons and extremely large VLDL (%) | 755 | -0.02 | -0.11 | 0.07 | 0.735 | 755 | -0.04 | -0.13 | 0.05 | 0.368 | 755 | -0.05 | -0.14 | 0.04 | 0.286 |
| Triglycerides to total lipids ratio in chylomicrons and extremely large VLDL (%) | 755 | 0.04 | -0.02 | 0.11 | 0.159 | 755 | 0.05 | -0.02 | 0.11 | 0.160 | 755 | 0.05 | -0.01 | 0.12 | 0.105 |
| Phospholipids to total lipds ratio in very large VLDL (%) | 755 | -0.03 | -0.12 | 0.05 | 0.438 | 755 | -0.06 | -0.15 | 0.03 | 0.174 | 755 | -0.07 | -0.16 | 0.02 | 0.121 |
| Total cholesterol to total lipids ratio in very large VLDL (%) | 755 | -0.10 | -0.22 | 0.02 | 0.090 | 755 | -0.07 | -0.17 | 0.03 | 0.194 | 755 | -0.06 | -0.17 | 0.04 | 0.220 |
| Cholesterol esters to total lipids ratio in very large VLDL (%) | 755 | -0.06 | -0.15 | 0.03 | 0.202 | 755 | -0.03 | -0.12 | 0.06 | 0.514 | 755 | -0.03 | -0.12 | 0.06 | 0.576 |
| Free cholesterol to total lipids ratio in very large VLDL (%) | 755 | -0.08 | -0.17 | 0.01 | 0.084 | 755 | -0.06 | -0.16 | 0.03 | 0.183 | 755 | -0.06 | -0.16 | 0.03 | 0.213 |
| Triglycerides to total lipids ratio in very large VLDL (%) | 755 | 0.09 | 0.00 | 0.18 | 0.053 | 755 | 0.07 | -0.02 | 0.17 | 0.127 | 755 | 0.07 | -0.02 | 0.16 | 0.138 |
| Phospholipids to total lipds ratio in large VLDL (%) | 755 | 0.00 | -0.10 | 0.10 | 0.973 | 755 | -0.03 | -0.13 | 0.07 | 0.601 | 755 | -0.04 | -0.14 | 0.07 | 0.495 |
| Total cholesterol to total lipids ratio in large VLDL (%) | 755 | -0.04 | -0.13 | 0.05 | 0.418 | 755 | -0.04 | -0.14 | 0.05 | 0.382 | 755 | -0.05 | -0.14 | 0.04 | 0.297 |
| Cholesterol esters to total lipids ratio in large VLDL (%) | 755 | -0.10 | -0.26 | 0.06 | 0.232 | 755 | -0.06 | -0.17 | 0.05 | 0.258 | 755 | -0.07 | -0.19 | 0.05 | 0.249 |
| Free cholesterol to total lipids ratio in large VLDL (%) | 755 | 0.00 | -0.09 | 0.10 | 0.918 | 755 | -0.02 | -0.12 | 0.07 | 0.634 | 755 | -0.03 | -0.13 | 0.06 | 0.489 |
| Triglycerides to total lipids ratio in large VLDL (%) | 755 | -0.09 | -0.27 | 0.09 | 0.331 | 755 | -0.05 | -0.17 | 0.07 | 0.384 | 755 | -0.06 | -0.18 | 0.07 | 0.384 |
| Phospholipids to total lipds ratio in medium VLDL (%) | 755 | -0.05 | -0.14 | 0.05 | 0.337 | 755 | -0.03 | -0.12 | 0.07 | 0.603 | 755 | -0.01 | -0.11 | 0.08 | 0.788 |
| Total cholesterol to total lipids ratio in medium VLDL (%) | 755 | -0.02 | -0.11 | 0.06 | 0.607 | 755 | -0.01 | -0.10 | 0.07 | 0.770 | 755 | -0.02 | -0.10 | 0.07 | 0.728 |
| Cholesterol esters to total lipids ratio in medium VLDL (%) | 755 | -0.02 | -0.11 | 0.06 | 0.596 | 755 | -0.01 | -0.09 | 0.08 | 0.870 | 755 | -0.01 | -0.10 | 0.08 | 0.831 |
| Free cholesterol to total lipids ratio in medium VLDL (%) | 755 | -0.01 | -0.09 | 0.07 | 0.807 | 755 | -0.03 | -0.11 | 0.06 | 0.548 | 755 | -0.03 | -0.11 | 0.06 | 0.513 |
| Triglycerides to total lipids ratio in medium VLDL (%) | 755 | 0.03 | -0.05 | 0.11 | 0.476 | 755 | 0.02 | -0.07 | 0.10 | 0.689 | 755 | 0.02 | -0.07 | 0.10 | 0.694 |
| Phospholipids to total lipds ratio in small VLDL (%) | 755 | -0.05 | -0.14 | 0.04 | 0.248 | 755 | -0.01 | -0.10 | 0.08 | 0.783 | 755 | 0.00 | -0.09 | 0.09 | 0.945 |
| Total cholesterol to total lipids ratio in small VLDL (%) | 755 | -0.02 | -0.10 | 0.06 | 0.606 | 755 | -0.02 | -0.10 | 0.07 | 0.681 | 755 | -0.02 | -0.10 | 0.07 | 0.676 |
| Cholesterol esters to total lipids ratio in small VLDL (%) | 755 | -0.02 | -0.10 | 0.06 | 0.652 | 755 | -0.02 | -0.11 | 0.06 | 0.629 | 755 | -0.02 | -0.11 | 0.06 | 0.596 |
| Free cholesterol to total lipids ratio in small VLDL (%) | 755 | -0.03 | -0.12 | 0.06 | 0.505 | 755 | 0.01 | -0.09 | 0.10 | 0.851 | 755 | 0.02 | -0.07 | 0.11 | 0.679 |
| Triglycerides to total lipids ratio in small VLDL (%) | 755 | 0.04 | -0.05 | 0.12 | 0.387 | 755 | 0.02 | -0.06 | 0.10 | 0.630 | 755 | 0.02 | -0.07 | 0.10 | 0.673 |
| Phospholipids to total lipds ratio in very small VLDL (%) | 755 | -0.05 | -0.13 | 0.02 | 0.173 | 755 | -0.05 | -0.13 | 0.02 | 0.181 | 755 | -0.05 | -0.12 | 0.03 | 0.237 |
| Total cholesterol to total lipids ratio in very small VLDL (%) | 755 | 0.03 | -0.04 | 0.11 | 0.381 | 755 | 0.05 | -0.03 | 0.13 | 0.236 | 755 | 0.04 | -0.03 | 0.12 | 0.275 |
| Cholesterol esters to total lipids ratio in very small VLDL (%) | 755 | 0.03 | -0.05 | 0.10 | 0.492 | 755 | 0.02 | -0.06 | 0.10 | 0.581 | 755 | 0.02 | -0.06 | 0.09 | 0.701 |
| Free cholesterol to total lipids ratio in very small VLDL (%) | 755 | 0.03 | -0.05 | 0.12 | 0.442 | 755 | 0.08 | -0.01 | 0.16 | 0.084 | 755 | 0.08 | 0.00 | 0.17 | 0.061 |
| Triglycerides to total lipids ratio in very small VLDL (%) | 755 | 0.00 | -0.08 | 0.07 | 0.908 | 755 | -0.02 | -0.10 | 0.06 | 0.633 | 755 | -0.02 | -0.10 | 0.06 | 0.628 |
| Phospholipids to total lipds ratio in IDL (%) | 755 | -0.11 | -0.20 | -0.02 | 0.019 | 755 | -0.10 | -0.19 | 0.00 | 0.040 | 755 | -0.09 | -0.18 | 0.01 | 0.066 |
| Total cholesterol to total lipids ratio in IDL (%) | 755 | 0.07 | -0.02 | 0.15 | 0.126 | 755 | 0.06 | -0.03 | 0.15 | 0.172 | 755 | 0.05 | -0.04 | 0.14 | 0.243 |
| Cholesterol esters to total lipids ratio in IDL (%) | 755 | 0.08 | -0.01 | 0.16 | 0.067 | 755 | 0.07 | -0.02 | 0.15 | 0.141 | 755 | 0.05 | -0.03 | 0.14 | 0.233 |
| Free cholesterol to total lipids ratio in IDL (%) | 755 | -0.03 | -0.10 | 0.05 | 0.446 | 755 | -0.01 | -0.09 | 0.06 | 0.771 | 755 | 0.00 | -0.07 | 0.07 | 0.979 |
| Triglycerides to total lipids ratio in IDL (%) | 755 | -0.03 | -0.11 | 0.05 | 0.490 | 755 | -0.03 | -0.11 | 0.06 | 0.530 | 755 | -0.02 | -0.11 | 0.06 | 0.619 |
| Phospholipids to total lipds ratio in large LDL (%) | 755 | 0.00 | -0.08 | 0.07 | 0.913 | 755 | 0.00 | -0.07 | 0.08 | 0.895 | 755 | 0.00 | -0.07 | 0.08 | 0.930 |
| Total cholesterol to total lipids ratio in large LDL (%) | 755 | 0.02 | -0.06 | 0.10 | 0.577 | 755 | 0.01 | -0.07 | 0.09 | 0.760 | 755 | 0.01 | -0.07 | 0.09 | 0.837 |
| Cholesterol esters to total lipids ratio in large LDL (%) | 755 | 0.02 | -0.06 | 0.10 | 0.690 | 755 | 0.00 | -0.08 | 0.08 | 0.990 | 755 | -0.01 | -0.09 | 0.08 | 0.881 |
| Free cholesterol to total lipids ratio in large LDL (%) | 755 | 0.01 | -0.07 | 0.08 | 0.810 | 755 | 0.03 | -0.04 | 0.11 | 0.371 | 755 | 0.04 | -0.03 | 0.12 | 0.276 |
| Triglycerides to total lipids ratio in large LDL (%) | 755 | -0.04 | -0.12 | 0.05 | 0.418 | 755 | -0.03 | -0.12 | 0.06 | 0.524 | 755 | -0.02 | -0.11 | 0.07 | 0.660 |
| Phospholipids to total lipds ratio in medium LDL (%) | 755 | 0.00 | -0.02 | 0.03 | 0.797 | 755 | 0.01 | -0.02 | 0.04 | 0.543 | 755 | 0.01 | -0.02 | 0.04 | 0.571 |

**S4 Table** Associations of current sedentary time (SED at age 15y) with metabolic traits at age 15y in ALSPAC

| | SED at age 15y (per SD (66 min/day) higher) | | | | | | | | | | | | | | |
|---|---|---|---|---|---|---|---|---|---|---|---|---|---|---|---|
| | *Adj. for age, sex, ethnicity, maternal education, smoking, alcohol, wear time, wear month* | | | | | *Additionally adj. for MVPA at age 15y* | | | | | *Additionally adj. for FMI at age 15y* | | | | |
| **Standardised outcome at age 15y** | **N** | **Beta** | **LCL** | **UCL** | **P-value** | **N** | **Beta** | **LCL** | **UCL** | **P-value** | **N** | **Beta** | **LCL** | **UCL** | **P-value** |
| Total cholesterol to total lipids ratio in medium LDL (%) | 755 | 0.02 | -0.06 | 0.11 | 0.577 | 755 | 0.01 | -0.08 | 0.09 | 0.857 | 755 | 0.01 | -0.08 | 0.09 | 0.903 |
| Cholesterol esters to total lipids ratio in medium LDL (%) | 755 | 0.00 | -0.08 | 0.09 | 0.934 | 755 | -0.02 | -0.10 | 0.07 | 0.705 | 755 | -0.02 | -0.10 | 0.07 | 0.663 |
| Free cholesterol to total lipids ratio in medium LDL (%) | 755 | 0.01 | -0.01 | 0.03 | 0.525 | 755 | 0.01 | -0.01 | 0.04 | 0.249 | 755 | 0.01 | -0.01 | 0.04 | 0.229 |
| Triglycerides to total lipids ratio in medium LDL (%) | 755 | -0.08 | -0.16 | 0.01 | 0.068 | 755 | -0.07 | -0.16 | 0.02 | 0.106 | 755 | -0.06 | -0.15 | 0.02 | 0.156 |
| Phospholipids to total lipds ratio in small LDL (%) | 755 | 0.01 | -0.04 | 0.05 | 0.778 | 755 | 0.02 | -0.03 | 0.06 | 0.483 | 755 | 0.02 | -0.03 | 0.07 | 0.464 |
| Total cholesterol to total lipids ratio in small LDL (%) | 755 | 0.01 | -0.08 | 0.09 | 0.900 | 755 | -0.01 | -0.09 | 0.08 | 0.872 | 755 | -0.01 | -0.09 | 0.07 | 0.841 |
| Cholesterol esters to total lipids ratio in small LDL (%) | 755 | -0.01 | -0.09 | 0.07 | 0.775 | 755 | -0.03 | -0.11 | 0.06 | 0.501 | 755 | -0.03 | -0.11 | 0.05 | 0.478 |
| Free cholesterol to total lipids ratio in small LDL (%) | 755 | 0.02 | -0.02 | 0.06 | 0.347 | 755 | 0.03 | -0.01 | 0.07 | 0.151 | 755 | 0.03 | -0.01 | 0.08 | 0.144 |
| Triglycerides to total lipids ratio in small LDL (%) | 755 | -0.05 | -0.13 | 0.03 | 0.217 | 755 | -0.06 | -0.14 | 0.02 | 0.135 | 755 | -0.06 | -0.14 | 0.02 | 0.142 |
| Phospholipids to total lipds ratio in very large HDL (%) | 755 | -0.05 | -0.13 | 0.02 | 0.139 | 755 | -0.02 | -0.10 | 0.05 | 0.524 | 755 | -0.01 | -0.08 | 0.06 | 0.839 |
| Total cholesterol to total lipids ratio in very large HDL (%) | 755 | 0.05 | -0.02 | 0.12 | 0.187 | 755 | 0.02 | -0.05 | 0.09 | 0.603 | 755 | 0.00 | -0.06 | 0.07 | 0.925 |
| Cholesterol esters to total lipids ratio in very large HDL (%) | 755 | 0.04 | -0.03 | 0.11 | 0.227 | 755 | 0.02 | -0.06 | 0.09 | 0.682 | 755 | 0.00 | -0.07 | 0.07 | 0.979 |
| Free cholesterol to total lipids ratio in very large HDL (%) | 755 | 0.02 | -0.05 | 0.10 | 0.563 | 755 | 0.03 | -0.05 | 0.12 | 0.401 | 755 | 0.04 | -0.04 | 0.12 | 0.304 |
| Triglycerides to total lipids ratio in very large HDL (%) | 755 | 0.05 | -0.04 | 0.14 | 0.243 | 755 | 0.03 | -0.06 | 0.12 | 0.475 | 755 | 0.02 | -0.06 | 0.11 | 0.599 |
| Phospholipids to total lipds ratio in large HDL (%) | 755 | -0.04 | -0.12 | 0.04 | 0.346 | 755 | -0.08 | -0.16 | 0.01 | 0.069 | 755 | -0.09 | -0.17 | -0.02 | 0.016 |
| Total cholesterol to total lipids ratio in large HDL (%) | 755 | 0.01 | -0.08 | 0.09 | 0.853 | 755 | 0.05 | -0.04 | 0.13 | 0.292 | 755 | 0.06 | -0.01 | 0.14 | 0.107 |
| Cholesterol esters to total lipids ratio in large HDL (%) | 755 | 0.00 | -0.08 | 0.09 | 0.915 | 755 | 0.04 | -0.04 | 0.13 | 0.331 | 755 | 0.06 | -0.02 | 0.14 | 0.128 |
| Free cholesterol to total lipids ratio in large HDL (%) | 755 | 0.02 | -0.06 | 0.10 | 0.632 | 755 | 0.05 | -0.03 | 0.13 | 0.246 | 755 | 0.06 | -0.02 | 0.14 | 0.116 |
| Triglycerides to total lipids ratio in large HDL (%) | 755 | 0.06 | -0.03 | 0.15 | 0.220 | 755 | 0.03 | -0.06 | 0.12 | 0.541 | 755 | 0.01 | -0.07 | 0.10 | 0.795 |
| Phospholipids to total lipds ratio in medium HDL (%) | 755 | -0.09 | -0.17 | 0.00 | 0.051 | 755 | -0.07 | -0.16 | 0.02 | 0.112 | 755 | -0.07 | -0.15 | 0.02 | 0.133 |
| Total cholesterol to total lipids ratio in medium HDL (%) | 755 | 0.07 | -0.02 | 0.15 | 0.112 | 755 | 0.08 | -0.01 | 0.16 | 0.085 | 755 | 0.08 | 0.00 | 0.17 | 0.064 |
| Cholesterol esters to total lipids ratio in medium HDL (%) | 755 | 0.07 | -0.02 | 0.15 | 0.120 | 755 | 0.08 | -0.01 | 0.17 | 0.085 | 755 | 0.08 | -0.01 | 0.17 | 0.065 |
| Free cholesterol to total lipids ratio in medium HDL (%) | 755 | 0.02 | -0.07 | 0.11 | 0.674 | 755 | 0.01 | -0.09 | 0.12 | 0.803 | 755 | 0.01 | -0.09 | 0.12 | 0.778 |
| Triglycerides to total lipids ratio in medium HDL (%) | 755 | 0.00 | -0.09 | 0.09 | 0.994 | 755 | -0.03 | -0.12 | 0.06 | 0.486 | 755 | -0.05 | -0.13 | 0.04 | 0.280 |
| Phospholipids to total lipds ratio in small HDL (%) | 755 | 0.10 | 0.03 | 0.17 | 0.008 | 755 | 0.08 | 0.00 | 0.15 | 0.049 | 755 | 0.07 | 0.00 | 0.15 | 0.060 |
| Total cholesterol to total lipids ratio in small HDL (%) | 755 | -0.10 | -0.17 | -0.03 | 0.007 | 755 | -0.07 | -0.14 | 0.01 | 0.071 | 755 | -0.06 | -0.14 | 0.01 | 0.092 |
| Cholesterol esters to total lipids ratio in small HDL (%) | 755 | -0.11 | -0.18 | -0.03 | 0.004 | 755 | -0.08 | -0.15 | -0.01 | 0.032 | 755 | -0.08 | -0.15 | 0.00 | 0.039 |
| Free cholesterol to total lipids ratio in small HDL (%) | 755 | 0.09 | 0.00 | 0.17 | 0.041 | 755 | 0.12 | 0.03 | 0.21 | 0.012 | 755 | 0.13 | 0.04 | 0.22 | 0.004 |
| Triglycerides to total lipids ratio in small HDL (%) | 755 | 0.03 | -0.05 | 0.12 | 0.464 | 755 | -0.01 | -0.09 | 0.08 | 0.903 | 755 | -0.01 | -0.10 | 0.07 | 0.735 |
| Mean diameter for VLDL particles (nm) | 755 | 0.05 | -0.04 | 0.13 | 0.268 | 755 | 0.01 | -0.07 | 0.10 | 0.793 | 755 | 0.00 | -0.08 | 0.08 | 0.965 |
| Mean diameter for LDL particles (nm) | 755 | 0.02 | -0.06 | 0.10 | 0.612 | 755 | 0.04 | -0.04 | 0.12 | 0.314 | 755 | 0.05 | -0.03 | 0.12 | 0.242 |
| Mean diameter for HDL particles (nm) | 755 | -0.03 | -0.11 | 0.05 | 0.492 | 755 | 0.01 | -0.08 | 0.09 | 0.854 | 755 | 0.03 | -0.05 | 0.11 | 0.526 |
| Serum total cholesterol (mmol/l) | 755 | -0.04 | -0.11 | 0.04 | 0.361 | 755 | -0.04 | -0.12 | 0.04 | 0.325 | 755 | -0.04 | -0.12 | 0.04 | 0.311 |
| Total cholesterol in VLDL (mmol/l) | 755 | 0.04 | -0.05 | 0.13 | 0.365 | 755 | 0.01 | -0.08 | 0.10 | 0.814 | 755 | -0.01 | -0.09 | 0.08 | 0.898 |
| Remnant cholesterol (non-HDL, non-LDL -cholesterol) (mmol/l) | 755 | 0.02 | -0.07 | 0.10 | 0.718 | 755 | -0.01 | -0.09 | 0.08 | 0.880 | 755 | -0.02 | -0.10 | 0.07 | 0.677 |
| Total cholesterol in LDL (mmol/l) | 755 | -0.04 | -0.12 | 0.04 | 0.328 | 755 | -0.05 | -0.14 | 0.03 | 0.212 | 755 | -0.06 | -0.14 | 0.03 | 0.189 |
| Total cholesterol in HDL (mmol/l) | 755 | -0.06 | -0.14 | 0.02 | 0.136 | 755 | -0.03 | -0.11 | 0.05 | 0.491 | 755 | -0.02 | -0.10 | 0.07 | 0.704 |
| Total cholesterol in HDL2 (mmol/l) | 755 | -0.07 | -0.15 | 0.02 | 0.121 | 755 | -0.03 | -0.12 | 0.05 | 0.465 | 755 | -0.02 | -0.10 | 0.06 | 0.684 |
| Total cholesterol in HDL3 (mmol/l) | 755 | -0.05 | -0.13 | 0.03 | 0.197 | 755 | -0.02 | -0.11 | 0.06 | 0.569 | 755 | -0.01 | -0.09 | 0.07 | 0.760 |
| Esterified cholesterol (mmol/l) | 755 | -0.04 | -0.12 | 0.04 | 0.354 | 755 | -0.04 | -0.12 | 0.04 | 0.322 | 755 | -0.04 | -0.12 | 0.04 | 0.311 |
| Free cholesterol (mmol/l) | 755 | -0.03 | -0.11 | 0.05 | 0.403 | 755 | -0.04 | -0.12 | 0.04 | 0.360 | 755 | -0.04 | -0.12 | 0.04 | 0.336 |
| Serum total triglycerides (mmol/l) | 755 | 0.04 | -0.04 | 0.12 | 0.357 | 755 | 0.00 | -0.08 | 0.09 | 0.923 | 755 | -0.01 | -0.09 | 0.07 | 0.848 |
| Triglycerides in VLDL (mmol/l) | 755 | 0.06 | -0.03 | 0.14 | 0.180 | 755 | 0.02 | -0.06 | 0.11 | 0.625 | 755 | 0.01 | -0.07 | 0.09 | 0.866 |
| Triglycerides in LDL (mmol/l) | 755 | -0.06 | -0.14 | 0.02 | 0.158 | 755 | -0.07 | -0.15 | 0.01 | 0.078 | 755 | -0.07 | -0.15 | 0.01 | 0.095 |
| Triglycerides in HDL (mmol/l) | 755 | 0.00 | -0.08 | 0.08 | 0.994 | 755 | -0.02 | -0.10 | 0.06 | 0.657 | 755 | -0.02 | -0.11 | 0.06 | 0.559 |
| Diacylglycerol (mmol/l) | 755 | 0.03 | -0.05 | 0.10 | 0.533 | 755 | 0.02 | -0.06 | 0.11 | 0.578 | 755 | 0.02 | -0.07 | 0.10 | 0.710 |
| Ratio of diacylglycerol to triglycerides | 755 | 0.00 | -0.08 | 0.08 | 0.916 | 755 | 0.01 | -0.08 | 0.09 | 0.886 | 755 | 0.00 | -0.08 | 0.09 | 0.932 |
| Total phosphoglycerides (mmol/l) | 755 | -0.06 | -0.14 | 0.02 | 0.133 | 755 | -0.05 | -0.13 | 0.03 | 0.217 | 755 | -0.05 | -0.13 | 0.03 | 0.260 |

**S4 Table** Associations of current sedentary time (SED at age 15y) with metabolic traits at age 15y in ALSPAC

**SED at age 15y (per SD (66 min/day) higher)**

| | *Adj. for age, sex, ethnicity, maternal education, smoking, alcohol, wear time, wear month* | | | | | *Additionally adj. for MVPA at age 15y* | | | | | *Additionally adj. for FMI at age 15y* | | | | |
|---|---|---|---|---|---|---|---|---|---|---|---|---|---|---|---|
| **Standardised outcome at age 15y** | **N** | **Beta** | **LCL** | **UCL** | **P-value** | **N** | **Beta** | **LCL** | **UCL** | **P-value** | **N** | **Beta** | **LCL** | **UCL** | **P-value** |
| Ratio of triglycerides to phosphoglycerides | 755 | 0.08 | 0.00 | 0.16 | 0.057 | 755 | 0.05 | -0.03 | 0.13 | 0.231 | 755 | 0.03 | -0.04 | 0.11 | 0.373 |
| Phosphatidylcholine and other cholines (mmol/l) | 755 | -0.04 | -0.12 | 0.03 | 0.284 | 755 | -0.03 | -0.11 | 0.05 | 0.472 | 755 | -0.02 | -0.10 | 0.05 | 0.538 |
| Total cholines (mmol/l) | 755 | -0.07 | -0.14 | 0.01 | 0.097 | 755 | -0.06 | -0.14 | 0.02 | 0.174 | 755 | -0.05 | -0.13 | 0.03 | 0.210 |
| Apolipoprotein A-I (g/l) | 755 | -0.05 | -0.13 | 0.03 | 0.186 | 755 | -0.03 | -0.11 | 0.05 | 0.438 | 755 | -0.02 | -0.10 | 0.06 | 0.578 |
| Apolipoprotein B (g/l) | 755 | 0.01 | -0.07 | 0.10 | 0.739 | 755 | -0.01 | -0.10 | 0.07 | 0.747 | 755 | -0.03 | -0.11 | 0.06 | 0.557 |
| Ratio of apolipoprotein B to apolipoprotein A-I | 755 | 0.04 | -0.05 | 0.13 | 0.388 | 755 | 0.00 | -0.09 | 0.09 | 0.975 | 755 | -0.01 | -0.10 | 0.07 | 0.745 |
| Total fatty acids (mmol/l) | 755 | -0.01 | -0.09 | 0.07 | 0.834 | 755 | -0.02 | -0.10 | 0.06 | 0.561 | 755 | -0.03 | -0.11 | 0.05 | 0.482 |
| Estimated description of fatty acid chain length, not actual carbon number | 755 | 0.04 | -0.04 | 0.12 | 0.364 | 755 | 0.04 | -0.04 | 0.13 | 0.304 | 755 | 0.04 | -0.04 | 0.12 | 0.347 |
| Estimated degree of unsaturation | 755 | 0.02 | -0.06 | 0.10 | 0.590 | 755 | 0.04 | -0.04 | 0.12 | 0.338 | 755 | 0.04 | -0.04 | 0.12 | 0.346 |
| 22:6, docosahexaenoic acid (mmol/l) | 755 | -0.06 | -0.14 | 0.02 | 0.175 | 755 | -0.05 | -0.14 | 0.03 | 0.196 | 755 | -0.06 | -0.14 | 0.02 | 0.163 |
| 18:2, linoleic acid (mmol/l) | 755 | -0.02 | -0.10 | 0.05 | 0.558 | 755 | -0.03 | -0.11 | 0.06 | 0.529 | 755 | -0.03 | -0.11 | 0.06 | 0.537 |
| Conjugated linoleic acid (mmol/l) | 755 | 0.01 | -0.08 | 0.10 | 0.786 | 755 | 0.01 | -0.09 | 0.10 | 0.858 | 755 | 0.01 | -0.09 | 0.10 | 0.850 |
| Omega-3 fatty acids (mmol/l) | 755 | -0.03 | -0.11 | 0.05 | 0.431 | 755 | -0.04 | -0.12 | 0.04 | 0.312 | 755 | -0.04 | -0.12 | 0.03 | 0.270 |
| Omega-6 fatty acids (mmol/l) | 755 | -0.02 | -0.10 | 0.06 | 0.604 | 755 | -0.02 | -0.10 | 0.06 | 0.551 | 755 | -0.03 | -0.10 | 0.05 | 0.534 |
| Polyunsaturated fatty acids (mmol/l) | 755 | -0.02 | -0.10 | 0.05 | 0.559 | 755 | -0.03 | -0.11 | 0.05 | 0.492 | 755 | -0.03 | -0.11 | 0.05 | 0.470 |
| Monounsaturated fatty acids; 16:1, 18:1 (mmol/l) | 755 | 0.02 | -0.06 | 0.10 | 0.694 | 755 | -0.01 | -0.09 | 0.08 | 0.885 | 755 | -0.01 | -0.10 | 0.07 | 0.720 |
| Saturated fatty acids (mmol/l) | 755 | -0.01 | -0.09 | 0.06 | 0.711 | 755 | -0.03 | -0.11 | 0.05 | 0.472 | 755 | -0.03 | -0.11 | 0.05 | 0.427 |
| Ratio of 22:6 docosahexaenoic acid to total fatty acids (%) | 755 | -0.06 | -0.14 | 0.03 | 0.169 | 755 | -0.05 | -0.14 | 0.04 | 0.264 | 755 | -0.05 | -0.14 | 0.04 | 0.245 |
| Ratio of 18:2 linoleic acid to total fatty acids (%) | 755 | 0.00 | -0.09 | 0.08 | 0.928 | 755 | 0.02 | -0.07 | 0.10 | 0.685 | 755 | 0.03 | -0.06 | 0.11 | 0.547 |
| Ratio of conjugated linoleic acid to total fatty acids (%) | 755 | 0.02 | -0.08 | 0.12 | 0.716 | 755 | 0.02 | -0.09 | 0.12 | 0.772 | 755 | 0.02 | -0.09 | 0.12 | 0.764 |
| Ratio of omega-3 fatty acids to total fatty acids (%) | 755 | -0.02 | -0.10 | 0.06 | 0.674 | 755 | -0.02 | -0.10 | 0.07 | 0.657 | 755 | -0.02 | -0.10 | 0.07 | 0.652 |
| Ratio of omega-6 fatty acids to total fatty acids (%) | 755 | 0.01 | -0.08 | 0.09 | 0.887 | 755 | 0.03 | -0.05 | 0.11 | 0.485 | 755 | 0.04 | -0.05 | 0.12 | 0.378 |
| Ratio of polyunsaturated fatty acids to total fatty acids (%) | 755 | 0.00 | -0.08 | 0.08 | 0.967 | 755 | 0.02 | -0.06 | 0.11 | 0.567 | 755 | 0.03 | -0.05 | 0.11 | 0.455 |
| Ratio of monounsaturated fatty acids to total fatty acids (%) | 755 | 0.03 | -0.06 | 0.11 | 0.560 | 755 | 0.01 | -0.08 | 0.09 | 0.903 | 755 | 0.00 | -0.09 | 0.08 | 0.923 |
| Ratio of saturated fatty acids to total fatty acids (%) | 755 | -0.03 | -0.12 | 0.05 | 0.398 | 755 | -0.04 | -0.12 | 0.04 | 0.379 | 755 | -0.03 | -0.11 | 0.05 | 0.436 |
| Insulin (mu/l) | 755 | 0.03 | -0.01 | 0.08 | 0.175 | 755 | 0.02 | -0.03 | 0.07 | 0.478 | 755 | 0.00 | -0.04 | 0.05 | 0.862 |
| Glucose (mmol/l) | 755 | 0.05 | -0.02 | 0.13 | 0.189 | 755 | 0.04 | -0.04 | 0.12 | 0.284 | 755 | 0.04 | -0.04 | 0.12 | 0.316 |
| Lactate (mmol/l) | 755 | 0.06 | -0.02 | 0.14 | 0.164 | 755 | 0.05 | -0.03 | 0.14 | 0.220 | 755 | 0.06 | -0.03 | 0.14 | 0.211 |
| Pyruvate (mmol/l) | 755 | 0.10 | 0.02 | 0.18 | 0.013 | 755 | 0.09 | 0.01 | 0.18 | 0.030 | 755 | 0.09 | 0.00 | 0.17 | 0.039 |
| Citrate (mmol/l) | 755 | -0.09 | -0.18 | 0.00 | 0.055 | 755 | -0.08 | -0.17 | 0.01 | 0.098 | 755 | -0.07 | -0.16 | 0.02 | 0.144 |
| Alanine (mmol/l) | 755 | 0.14 | 0.05 | 0.22 | 2.19E-03 | 755 | 0.14 | 0.05 | 0.23 | 1.67E-03 | 755 | 0.14 | 0.05 | 0.23 | 1.78E-03 |
| Glutamine (mmol/l) | 755 | -0.06 | -0.14 | 0.02 | 0.120 | 755 | -0.06 | -0.14 | 0.03 | 0.174 | 755 | -0.05 | -0.13 | 0.03 | 0.254 |
| Histidine (mmol/l) | 755 | 0.06 | -0.02 | 0.13 | 0.144 | 755 | 0.08 | 0.00 | 0.16 | 0.039 | 755 | 0.08 | 0.00 | 0.16 | 0.041 |
| Isoleucine (mmol/l) | 755 | 0.06 | -0.02 | 0.14 | 0.118 | 755 | 0.06 | -0.02 | 0.14 | 0.127 | 755 | 0.06 | -0.02 | 0.13 | 0.176 |
| Leucine (mmol/l) | 755 | 0.01 | -0.06 | 0.07 | 0.863 | 755 | 0.03 | -0.04 | 0.10 | 0.406 | 755 | 0.03 | -0.04 | 0.10 | 0.443 |
| Valine (mmol/l) | 755 | 0.02 | -0.05 | 0.10 | 0.523 | 755 | 0.03 | -0.04 | 0.11 | 0.396 | 755 | 0.03 | -0.05 | 0.11 | 0.479 |
| Phenylalanine (mmol/l) | 755 | -0.07 | -0.17 | 0.03 | 0.167 | 755 | -0.04 | -0.14 | 0.06 | 0.389 | 755 | -0.05 | -0.15 | 0.05 | 0.340 |
| Tyrosine (mmol/l) | 755 | -0.07 | -0.15 | 0.02 | 0.126 | 755 | -0.06 | -0.15 | 0.03 | 0.223 | 755 | -0.06 | -0.15 | 0.03 | 0.166 |
| Acetate (mmol/l) | 755 | 0.01 | -0.07 | 0.09 | 0.796 | 755 | 0.04 | -0.05 | 0.12 | 0.382 | 755 | 0.04 | -0.04 | 0.13 | 0.338 |
| Acetoacetate (mmol/l) | 755 | 0.00 | -0.09 | 0.09 | 0.978 | 755 | -0.01 | -0.10 | 0.08 | 0.858 | 755 | -0.01 | -0.10 | 0.08 | 0.846 |
| 3-hydroxybutyrate (mmol/l) | 755 | -0.03 | -0.13 | 0.07 | 0.517 | 755 | -0.06 | -0.17 | 0.05 | 0.286 | 755 | -0.06 | -0.17 | 0.05 | 0.273 |
| Creatinine (mmol/l) | 755 | 0.09 | 0.01 | 0.17 | 0.037 | 755 | 0.08 | 0.00 | 0.16 | 0.058 | 755 | 0.08 | 0.00 | 0.16 | 0.057 |
| Albumin (signal area) | 755 | 0.10 | 0.01 | 0.18 | 0.021 | 755 | 0.08 | -0.01 | 0.16 | 0.093 | 755 | 0.08 | -0.01 | 0.17 | 0.085 |
| Glycoprotein acetyls, mainly a1-acid glycoprotein (mmol/l) | 755 | 0.03 | -0.05 | 0.12 | 0.432 | 755 | -0.01 | -0.10 | 0.08 | 0.818 | 755 | -0.03 | -0.11 | 0.05 | 0.478 |
| C-reactive protein (mg/l) | 755 | 0.05 | -0.04 | 0.15 | 0.283 | 755 | 0.05 | -0.05 | 0.16 | 0.344 | 755 | 0.05 | -0.06 | 0.15 | 0.385 |
