# Supplementary material for: Associations of device-measured physical activity across adolescence with metabolic traits: Prospective cohort study
Source: PLoS Med. 2018 Sep 11;15(9):e1002649. doi: 10.1371/journal.pmed.1002649 (PMC6133272; doi:10.1371/journal.pmed.1002649)
Supplement: S5 Table — ALSPAC, Avon Longitudinal Study of Parents and Children; CPM, counts per minute. (PDF) [file pmed.1002649.s005.pdf]

S5 Table Associations of longer-term total physical activity (mean of CPM measures at age 12y, 14y, and 15y) with metabolic traits at age 15y in ALSPAC

	**Mean of CPM at age 12y, 14y, 15y (per SD (132) higher)**									
	Adj. for age, sex, ethnicity, maternal education, smoking, alcohol, mean wear time, wear month					*Additionally adj. for mean FMI*				
**Standardised outcome at age 15y**	**N**	**Beta**	**LCL**	**UCL**	**P-value**	**N**	**Beta**	**LCL**	**UCL**	**P-value**
Systolic blood pressure (mmHg)	1298	-0.13	-0.21	-0.05	1.46E-03	1256	-0.08	-0.16	0.01	0.069
Diastolic blood pressure (mmHg)	1298	-0.11	-0.20	-0.03	0.007	1256	-0.12	-0.20	-0.03	0.009
Concentration of chylomicrons and extremely large VLDL particles (mol/l)	896	-0.19	-0.30	-0.08	5.37E-04	874	-0.14	-0.24	-0.03	0.009
Total lipids in chylomicrons and extremely large VLDL (mmol/l)	896	-0.18	-0.29	-0.08	8.35E-04	874	-0.13	-0.23	-0.03	0.011
Phospholipids in chylomicrons and extremely large VLDL (mmol/l)	896	-0.19	-0.29	-0.08	6.03E-04	874	-0.14	-0.24	-0.04	0.008
Total cholesterol in chylomicrons and extremely large VLDL (mmol/l)	896	-0.15	-0.26	-0.04	0.008	874	-0.10	-0.20	0.01	0.065
Cholesterol esters in chylomicrons and extremely large VLDL (mmol/l)	896	-0.11	-0.22	0.00	0.057	874	-0.06	-0.16	0.05	0.286
Free cholesterol in chylomicrons and extremely large VLDL (mmol/l)	896	-0.18	-0.29	-0.08	8.05E-04	874	-0.13	-0.24	-0.03	0.010
Triglycerides in chylomicrons and extremely large VLDL (mmol/l)	896	-0.19	-0.30	-0.08	5.22E-04	874	-0.14	-0.24	-0.04	0.008
Concentration of very large VLDL particles (mol/l)	896	-0.17	-0.28	-0.06	1.78E-03	874	-0.12	-0.22	-0.02	0.024
Total lipids in very large VLDL (mmol/l)	896	-0.16	-0.27	-0.06	2.71E-03	874	-0.11	-0.21	-0.01	0.031
Phospholipids in very large VLDL (mmol/l)	896	-0.17	-0.27	-0.06	2.49E-03	874	-0.12	-0.22	-0.01	0.026
Total cholesterol in very large VLDL (mmol/l)	896	-0.16	-0.27	-0.06	0.003	874	-0.11	-0.21	-0.01	0.035
Cholesterol esters in very large VLDL (mmol/l)	896	-0.16	-0.26	-0.05	0.005	874	-0.10	-0.20	0.00	0.059
Free cholesterol in very large VLDL (mmol/l)	896	-0.17	-0.28	-0.06	1.89E-03	874	-0.12	-0.22	-0.02	0.021
Triglycerides in very large VLDL (mmol/l)	896	-0.16	-0.27	-0.06	2.81E-03	874	-0.11	-0.21	-0.01	0.032
Concentration of large VLDL particles (mol/l)	896	-0.15	-0.26	-0.04	0.007	874	-0.09	-0.20	0.01	0.069
Total lipids in large VLDL (mmol/l)	896	-0.15	-0.25	-0.04	0.008	874	-0.09	-0.19	0.01	0.080
Phospholipids in large VLDL (mmol/l)	896	-0.14	-0.25	-0.04	0.008	874	-0.09	-0.19	0.01	0.081
Total cholesterol in large VLDL (mmol/l)	896	-0.13	-0.24	-0.02	0.019	874	-0.07	-0.18	0.03	0.152
Cholesterol esters in large VLDL (mmol/l)	896	-0.11	-0.22	0.00	0.042	874	-0.06	-0.16	0.05	0.281
Free cholesterol in large VLDL (mmol/l)	896	-0.14	-0.25	-0.04	0.009	874	-0.09	-0.19	0.01	0.081
Triglycerides in large VLDL (mmol/l)	896	-0.15	-0.26	-0.04	0.006	874	-0.10	-0.20	0.01	0.062
Concentration of medium VLDL particles (mol/l)	896	-0.14	-0.25	-0.04	0.009	874	-0.09	-0.19	0.02	0.099
Total lipids in medium VLDL (mmol/l)	896	-0.13	-0.24	-0.03	0.015	874	-0.08	-0.18	0.03	0.141
Phospholipids in medium VLDL (mmol/l)	896	-0.13	-0.23	-0.02	0.019	874	-0.07	-0.18	0.03	0.153
Total cholesterol in medium VLDL (mmol/l)	896	-0.09	-0.20	0.02	0.103	874	-0.04	-0.14	0.07	0.491
Cholesterol esters in medium VLDL (mmol/l)	896	-0.05	-0.16	0.05	0.318	874	0.00	-0.11	0.10	0.967
Free cholesterol in medium VLDL (mmol/l)	896	-0.12	-0.23	-0.02	0.022	874	-0.07	-0.18	0.03	0.154
Triglycerides in medium VLDL (mmol/l)	896	-0.15	-0.26	-0.05	0.005	874	-0.09	-0.20	0.01	0.067
Concentration of small VLDL particles (mol/l)	896	-0.11	-0.21	0.00	0.046	874	-0.06	-0.16	0.05	0.284
Total lipids in small VLDL (mmol/l)	896	-0.10	-0.20	0.00	0.062	874	-0.05	-0.15	0.06	0.371
Phospholipids in small VLDL (mmol/l)	896	-0.06	-0.17	0.04	0.210	874	-0.01	-0.11	0.09	0.822
Total cholesterol in small VLDL (mmol/l)	896	-0.07	-0.17	0.03	0.156	874	-0.02	-0.12	0.08	0.655
Cholesterol esters in small VLDL (mmol/l)	896	-0.07	-0.17	0.02	0.139	874	-0.03	-0.13	0.08	0.607
Free cholesterol in small VLDL (mmol/l)	896	-0.06	-0.16	0.04	0.246	874	-0.01	-0.11	0.09	0.791
Triglycerides in small VLDL (mmol/l)	896	-0.12	-0.23	-0.02	0.025	874	-0.07	-0.18	0.03	0.153
Concentration of very small VLDL particles (mol/l)	896	0.01	-0.08	0.11	0.777	874	0.03	-0.07	0.13	0.533
Total lipids in very small VLDL (mmol/l)	896	-0.02	-0.12	0.08	0.684	874	0.01	-0.10	0.11	0.915
Phospholipids in very small VLDL (mmol/l)	896	0.02	-0.08	0.11	0.748	874	0.02	-0.08	0.12	0.673
Total cholesterol in very small VLDL (mmol/l)	896	-0.02	-0.12	0.08	0.727	874	0.02	-0.09	0.12	0.779
Cholesterol esters in very small VLDL (mmol/l)	896	-0.06	-0.16	0.04	0.259	874	-0.02	-0.12	0.09	0.749
Free cholesterol in very small VLDL (mmol/l)	896	0.07	-0.03	0.17	0.169	874	0.08	-0.02	0.19	0.123
Triglycerides in very small VLDL (mmol/l)	896	-0.06	-0.16	0.03	0.198	874	-0.04	-0.14	0.06	0.402
Concentration of IDL particles (mol/l)	896	0.01	-0.09	0.10	0.841	874	0.00	-0.10	0.10	0.988
Total lipids in IDL (mmol/l)	896	0.01	-0.08	0.11	0.763	874	0.01	-0.09	0.11	0.805
Phospholipids in IDL (mmol/l)	896	0.02	-0.08	0.11	0.742	874	0.01	-0.09	0.11	0.902
Total cholesterol in IDL (mmol/l)	896	0.01	-0.08	0.11	0.800	874	0.02	-0.08	0.12	0.745
Cholesterol esters in IDL (mmol/l)	896	0.00	-0.09	0.10	0.946	874	0.01	-0.09	0.12	0.778
Free cholesterol in IDL (mmol/l)	896	0.03	-0.06	0.13	0.492	874	0.02	-0.08	0.12	0.679
Triglycerides in IDL (mmol/l)	896	0.02	-0.07	0.11	0.680	874	0.00	-0.10	0.09	0.947
Concentration of large LDL particles (mol/l)	896	0.01	-0.08	0.10	0.847	874	0.00	-0.09	0.10	0.925
Total lipids in large LDL (mmol/l)	896	0.01	-0.08	0.11	0.773	874	0.01	-0.09	0.11	0.815
Phospholipids in large LDL (mmol/l)	896	0.01	-0.08	0.10	0.824	874	0.01	-0.08	0.11	0.771
Total cholesterol in large LDL (mmol/l)	896	0.01	-0.08	0.11	0.789	874	0.01	-0.09	0.11	0.794
Cholesterol esters in large LDL (mmol/l)	896	0.01	-0.09	0.10	0.875	874	0.01	-0.09	0.11	0.836
Free cholesterol in large LDL (mmol/l)	896	0.03	-0.07	0.12	0.548	874	0.02	-0.08	0.12	0.674
Triglycerides in large LDL (mmol/l)	896	0.02	-0.07	0.11	0.622	874	-0.01	-0.10	0.09	0.852
Concentration of medium LDL particles (mol/l)	896	-0.01	-0.10	0.08	0.848	874	-0.01	-0.11	0.09	0.866
Total lipids in medium LDL (mmol/l)	896	0.00	-0.09	0.10	0.953	874	0.00	-0.09	0.10	0.932
Phospholipids in medium LDL (mmol/l)	896	0.01	-0.09	0.10	0.910	874	0.02	-0.08	0.12	0.693
Total cholesterol in medium LDL (mmol/l)	896	0.00	-0.09	0.09	0.997	874	0.00	-0.10	0.10	0.970
Cholesterol esters in medium LDL (mmol/l)	896	-0.01	-0.10	0.09	0.893	874	0.00	-0.10	0.10	0.928
Free cholesterol in medium LDL (mmol/l)	896	0.03	-0.07	0.12	0.575	874	0.03	-0.07	0.13	0.560
Triglycerides in medium LDL (mmol/l)	896	0.02	-0.07	0.11	0.683	874	-0.01	-0.11	0.09	0.833
Concentration of small LDL particles (mol/l)	896	-0.01	-0.10	0.09	0.875	874	-0.01	-0.11	0.09	0.893
Total lipids in small LDL (mmol/l)	896	0.00	-0.09	0.09	0.992	874	0.00	-0.10	0.10	0.959
Phospholipids in small LDL (mmol/l)	896	0.01	-0.08	0.11	0.790	874	0.02	-0.08	0.12	0.674
Total cholesterol in small LDL (mmol/l)	896	0.00	-0.10	0.09	0.989	874	0.00	-0.10	0.10	0.972
Cholesterol esters in small LDL (mmol/l)	896	-0.01	-0.10	0.09	0.841	874	-0.01	-0.11	0.09	0.865
Free cholesterol in small LDL (mmol/l)	896	0.04	-0.06	0.13	0.416	874	0.05	-0.05	0.15	0.349
Triglycerides in small LDL (mmol/l)	896	-0.03	-0.13	0.06	0.522	874	-0.04	-0.14	0.06	0.469
Concentration of very large HDL particles (mol/l)	896	0.14	0.04	0.24	0.006	874	0.07	-0.03	0.17	0.174
Total lipids in very large HDL (mmol/l)	896	0.13	0.03	0.23	0.009	874	0.06	-0.04	0.17	0.214
Phospholipids in very large HDL (mmol/l)	896	0.15	0.05	0.25	0.003	874	0.08	-0.02	0.18	0.128
Total cholesterol in very large HDL (mmol/l)	896	0.11	0.01	0.21	0.028	874	0.05	-0.05	0.15	0.356
Cholesterol esters in very large HDL (mmol/l)	896	0.10	0.01	0.20	0.039	874	0.04	-0.06	0.14	0.404
Free cholesterol in very large HDL (mmol/l)	896	0.13	0.03	0.22	0.014	874	0.06	-0.04	0.16	0.266
Triglycerides in very large HDL (mmol/l)	896	0.04	-0.06	0.14	0.454	874	-0.01	-0.11	0.09	0.875
Concentration of large HDL particles (mol/l)	896	0.19	0.09	0.29	1.63E-04	874	0.12	0.02	0.22	0.014
Total lipids in large HDL (mmol/l)	896	0.19	0.09	0.28	2.00E-04	874	0.12	0.02	0.22	0.017
Phospholipids in large HDL (mmol/l)	896	0.18	0.08	0.28	3.33E-04	874	0.12	0.02	0.21	0.019
Total cholesterol in large HDL (mmol/l)	896	0.19	0.09	0.29	1.55E-04	874	0.12	0.02	0.22	0.016
Cholesterol esters in large HDL (mmol/l)	896	0.19	0.09	0.29	1.46E-04	874	0.12	0.02	0.22	0.016
Free cholesterol in large HDL (mmol/l)	896	0.18	0.09	0.28	2.06E-04	874	0.12	0.02	0.22	0.018
Triglycerides in large HDL (mmol/l)	896	0.11	0.02	0.21	0.020	874	0.08	-0.02	0.18	0.106
Concentration of medium HDL particles (mol/l)	896	0.15	0.07	0.24	6.96E-04	874	0.15	0.06	0.25	9.65E-04
Total lipids in medium HDL (mmol/l)	896	0.16	0.07	0.25	6.76E-04	874	0.15	0.06	0.24	1.35E-03
Phospholipids in medium HDL (mmol/l)	896	0.17	0.08	0.26	3.06E-04	874	0.16	0.06	0.25	9.46E-04
Total cholesterol in medium HDL (mmol/l)	896	0.15	0.06	0.24	1.41E-03	874	0.14	0.05	0.24	0.003
Cholesterol esters in medium HDL (mmol/l)	896	0.15	0.06	0.24	1.77E-03	874	0.14	0.05	0.24	0.004

S5 Table Associations of longer-term total physical activity (mean of CPM measures at age 12y, 14y, and 15y) with metabolic traits at age 15y in ALSPAC

	Mean of CPM at age 12y, 14y, 15y (per SD (132) higher)									
	Adj. for age, sex, ethnicity, maternal education, smoking, alcohol, mean wear time, wear month					*Additionally adj. for mean FMI*				
**Standardised outcome at age 15y**	**N**	**Beta**	**LCL**	**UCL**	**P-value**	**N**	**Beta**	**LCL**	**UCL**	**P-value**
Free cholesterol in medium HDL (mmol/l)	896	0.15	0.06	0.23	1.16E-03	874	0.14	0.05	0.23	2.26E-03
Triglycerides in medium HDL (mmol/l)	896	-0.03	-0.13	0.06	0.484	874	0.01	-0.08	0.11	0.797
Concentration of small HDL particles (mol/l)	896	0.01	-0.08	0.11	0.755	874	0.06	-0.04	0.15	0.222
Total lipids in small HDL (mmol/l)	896	0.09	0.00	0.18	0.043	874	0.12	0.03	0.21	0.011
Phospholipids in small HDL (mmol/l)	896	-0.01	-0.11	0.08	0.760	874	0.02	-0.07	0.12	0.620
Total cholesterol in small HDL (mmol/l)	896	0.19	0.10	0.27	1.73E-05	874	0.19	0.10	0.28	1.95E-05
Cholesterol esters in small HDL (mmol/l)	896	0.19	0.10	0.27	2.10E-05	874	0.19	0.10	0.27	2.44E-05
Free cholesterol in small HDL (mmol/l)	896	0.11	0.02	0.20	0.017	874	0.11	0.02	0.21	0.016
Triglycerides in small HDL (mmol/l)	896	-0.08	-0.18	0.02	0.098	874	-0.05	-0.15	0.04	0.271
Phospholipids to total lipds ratio in chylomicrons and extremely large VLDL (%)	896	-0.07	-0.16	0.02	0.148	874	-0.06	-0.16	0.03	0.199
Total cholesterol to total lipids ratio in chylomicrons and extremely large VLDL (%)	896	0.06	-0.05	0.16	0.275	874	0.09	-0.01	0.20	0.088
Cholesterol esters to total lipids ratio in chylomicrons and extremely large VLDL (%)	896	0.10	0.00	0.20	0.053	874	0.13	0.03	0.23	0.013
Free cholesterol to total lipids ratio in chylomicrons and extremely large VLDL (%)	896	-0.09	-0.19	0.02	0.096	874	-0.06	-0.17	0.05	0.273
Triglycerides to total lipids ratio in chylomicrons and extremely large VLDL (%)	896	-0.02	-0.09	0.05	0.547	874	-0.05	-0.12	0.02	0.191
Phospholipids to total lipds ratio in very large VLDL (%)	896	-0.09	-0.19	0.02	0.098	874	-0.06	-0.16	0.05	0.306
Total cholesterol to total lipids ratio in very large VLDL (%)	896	0.12	0.02	0.23	0.021	874	0.13	0.03	0.24	0.012
Cholesterol esters to total lipids ratio in very large VLDL (%)	896	0.16	0.05	0.27	0.005	874	0.16	0.04	0.27	0.007
Free cholesterol to total lipids ratio in very large VLDL (%)	896	0.12	0.00	0.23	0.043	874	0.11	0.00	0.23	0.060
Triglycerides to total lipids ratio in very large VLDL (%)	896	-0.13	-0.24	-0.01	0.028	874	-0.13	-0.25	-0.02	0.022
Phospholipids to total lipds ratio in large VLDL (%)	896	-0.11	-0.22	0.00	0.059	874	-0.08	-0.20	0.03	0.155
Total cholesterol to total lipids ratio in large VLDL (%)	896	-0.02	-0.12	0.08	0.734	874	0.01	-0.09	0.12	0.794
Cholesterol esters to total lipids ratio in large VLDL (%)	896	0.01	-0.07	0.09	0.781	874	0.05	-0.02	0.12	0.192
Free cholesterol to total lipids ratio in large VLDL (%)	896	-0.11	-0.22	-0.01	0.032	874	-0.08	-0.18	0.03	0.162
Triglycerides to total lipids ratio in large VLDL (%)	896	-0.02	-0.10	0.06	0.615	874	0.01	-0.05	0.08	0.665
Phospholipids to total lipds ratio in medium VLDL (%)	896	0.14	0.03	0.25	0.012	874	0.09	-0.03	0.20	0.133
Total cholesterol to total lipids ratio in medium VLDL (%)	896	0.10	0.00	0.20	0.042	874	0.10	0.00	0.20	0.047
Cholesterol esters to total lipids ratio in medium VLDL (%)	896	0.12	0.02	0.21	0.016	874	0.12	0.02	0.22	0.017
Free cholesterol to total lipids ratio in medium VLDL (%)	896	-0.02	-0.11	0.08	0.759	874	-0.02	-0.12	0.08	0.689
Triglycerides to total lipids ratio in medium VLDL (%)	896	-0.12	-0.22	-0.02	0.015	874	-0.11	-0.21	-0.01	0.032
Phospholipids to total lipds ratio in small VLDL (%)	896	0.19	0.09	0.29	2.20E-04	874	0.17	0.07	0.27	1.22E-03
Total cholesterol to total lipids ratio in small VLDL (%)	896	0.05	-0.05	0.15	0.321	874	0.05	-0.06	0.15	0.369
Cholesterol esters to total lipids ratio in small VLDL (%)	896	0.01	-0.09	0.11	0.778	874	0.02	-0.09	0.12	0.742
Free cholesterol to total lipids ratio in small VLDL (%)	896	0.24	0.14	0.34	2.14E-06	874	0.20	0.10	0.31	8.09E-05
Triglycerides to total lipids ratio in small VLDL (%)	896	-0.11	-0.21	0.00	0.042	874	-0.10	-0.20	0.01	0.070
Phospholipids to total lipds ratio in very small VLDL (%)	896	0.07	-0.03	0.16	0.172	874	0.03	-0.07	0.13	0.522
Total cholesterol to total lipids ratio in very small VLDL (%)	896	0.02	-0.08	0.12	0.739	874	0.04	-0.07	0.14	0.486
Cholesterol esters to total lipids ratio in very small VLDL (%)	896	-0.07	-0.17	0.02	0.116	874	-0.04	-0.14	0.05	0.371
Free cholesterol to total lipids ratio in very small VLDL (%)	896	0.20	0.10	0.31	1.10E-04	874	0.19	0.08	0.30	8.70E-04
Triglycerides to total lipids ratio in very small VLDL (%)	896	-0.07	-0.17	0.04	0.198	874	-0.07	-0.17	0.04	0.215
Phospholipids to total lipds ratio in IDL (%)	896	0.02	-0.09	0.13	0.783	874	-0.03	-0.14	0.09	0.644
Total cholesterol to total lipids ratio in IDL (%)	896	-0.01	-0.11	0.08	0.767	874	0.02	-0.08	0.13	0.672
Cholesterol esters to total lipids ratio in IDL (%)	896	-0.05	-0.15	0.05	0.323	874	0.01	-0.10	0.11	0.915
Free cholesterol to total lipids ratio in IDL (%)	896	0.08	-0.02	0.18	0.101	874	0.04	-0.06	0.14	0.451
Triglycerides to total lipids ratio in IDL (%)	896	0.01	-0.08	0.11	0.824	874	-0.01	-0.11	0.08	0.775
Phospholipids to total lipds ratio in large LDL (%)	896	-0.02	-0.10	0.07	0.694	874	0.00	-0.09	0.09	0.956
Total cholesterol to total lipids ratio in large LDL (%)	896	0.00	-0.09	0.09	0.988	874	0.01	-0.08	0.10	0.807
Cholesterol esters to total lipids ratio in large LDL (%)	896	-0.01	-0.10	0.07	0.742	874	0.00	-0.09	0.09	0.980
Free cholesterol to total lipids ratio in large LDL (%)	896	0.05	-0.04	0.13	0.312	874	0.03	-0.06	0.11	0.574
Triglycerides to total lipids ratio in large LDL (%)	896	0.02	-0.07	0.11	0.688	874	-0.02	-0.12	0.07	0.643
Phospholipids to total lipds ratio in medium LDL (%)	896	0.00	-0.04	0.03	0.821	874	0.00	-0.03	0.04	0.857
Total cholesterol to total lipids ratio in medium LDL (%)	896	-0.01	-0.10	0.08	0.861	874	-0.01	-0.10	0.09	0.886
Cholesterol esters to total lipids ratio in medium LDL (%)	896	-0.01	-0.10	0.08	0.812	874	-0.01	-0.11	0.09	0.821
Free cholesterol to total lipids ratio in medium LDL (%)	896	0.00	-0.02	0.03	0.767	874	0.00	-0.02	0.03	0.759
Triglycerides to total lipids ratio in medium LDL (%)	896	0.04	-0.05	0.13	0.376	874	0.00	-0.10	0.10	0.959
Phospholipids to total lipds ratio in small LDL (%)	896	0.00	-0.05	0.06	0.916	874	0.01	-0.05	0.06	0.818
Total cholesterol to total lipids ratio in small LDL (%)	896	0.00	-0.09	0.10	0.928	874	0.00	-0.10	0.10	0.972
Cholesterol esters to total lipids ratio in small LDL (%)	896	-0.01	-0.10	0.08	0.866	874	-0.01	-0.11	0.09	0.825
Free cholesterol to total lipids ratio in small LDL (%)	896	0.01	-0.04	0.06	0.571	874	0.02	-0.04	0.07	0.545
Triglycerides to total lipids ratio in small LDL (%)	896	-0.03	-0.12	0.07	0.577	874	-0.04	-0.14	0.06	0.469
Phospholipids to total lipds ratio in very large HDL (%)	896	0.13	0.03	0.22	0.008	874	0.07	-0.03	0.16	0.159
Total cholesterol to total lipids ratio in very large HDL (%)	896	-0.12	-0.21	-0.03	0.012	874	-0.06	-0.15	0.03	0.202
Cholesterol esters to total lipids ratio in very large HDL (%)	896	-0.11	-0.20	-0.02	0.017	874	-0.05	-0.15	0.04	0.244
Free cholesterol to total lipids ratio in very large HDL (%)	896	0.00	-0.10	0.10	0.984	874	-0.03	-0.13	0.07	0.595
Triglycerides to total lipids ratio in very large HDL (%)	896	-0.08	-0.19	0.02	0.130	874	-0.06	-0.16	0.05	0.289
Phospholipids to total lipds ratio in large HDL (%)	896	-0.11	-0.20	-0.02	0.012	874	-0.04	-0.13	0.04	0.326
Total cholesterol to total lipids ratio in large HDL (%)	896	0.13	0.03	0.22	0.010	874	0.05	-0.04	0.15	0.266
Cholesterol esters to total lipids ratio in large HDL (%)	896	0.13	0.03	0.22	0.009	874	0.05	-0.04	0.15	0.278
Free cholesterol to total lipids ratio in large HDL (%)	896	0.09	0.00	0.19	0.052	874	0.04	-0.05	0.14	0.351
Triglycerides to total lipids ratio in large HDL (%)	896	-0.12	-0.22	-0.01	0.032	874	-0.06	-0.16	0.05	0.279
Phospholipids to total lipds ratio in medium HDL (%)	896	0.15	0.05	0.24	0.003	874	0.12	0.02	0.22	0.024
Total cholesterol to total lipids ratio in medium HDL (%)	896	-0.05	-0.15	0.05	0.310	874	-0.06	-0.16	0.04	0.254
Cholesterol esters to total lipids ratio in medium HDL (%)	896	-0.05	-0.15	0.05	0.353	874	-0.06	-0.16	0.05	0.287
Free cholesterol to total lipids ratio in medium HDL (%)	896	-0.04	-0.16	0.09	0.552	874	-0.03	-0.16	0.10	0.614
Triglycerides to total lipids ratio in medium HDL (%)	896	-0.11	-0.21	0.00	0.040	874	-0.05	-0.15	0.05	0.296
Phospholipids to total lipds ratio in small HDL (%)	896	-0.19	-0.28	-0.10	2.20E-05	874	-0.18	-0.27	-0.08	1.78E-04
Total cholesterol to total lipids ratio in small HDL (%)	896	0.21	0.12	0.30	3.71E-06	874	0.19	0.10	0.28	4.50E-05
Cholesterol esters to total lipids ratio in small HDL (%)	896	0.19	0.10	0.28	2.94E-05	874	0.18	0.09	0.27	1.39E-04
Free cholesterol to total lipids ratio in small HDL (%)	896	0.06	-0.03	0.16	0.201	874	0.01	-0.09	0.11	0.842
Triglycerides to total lipids ratio in small HDL (%)	896	-0.14	-0.24	-0.04	0.005	874	-0.12	-0.22	-0.02	0.015
Mean diameter for VLDL particles (nm)	896	-0.16	-0.26	-0.06	1.92E-03	874	-0.11	-0.21	-0.01	0.036
Mean diameter for LDL particles (nm)	896	0.03	-0.05	0.12	0.482	874	0.00	-0.08	0.09	0.933
Mean diameter for HDL particles (nm)	896	0.16	0.06	0.26	1.88E-03	874	0.08	-0.02	0.18	0.104
Serum total cholesterol (mmol/l)	896	0.05	-0.04	0.14	0.297	874	0.05	-0.05	0.14	0.356
Total cholesterol in VLDL (mmol/l)	896	-0.09	-0.19	0.01	0.084	874	-0.04	-0.14	0.07	0.488
Remnant cholesterol (non-HDL, non-LDL -cholesterol) (mmol/l)	896	-0.05	-0.15	0.05	0.344	874	-0.01	-0.12	0.09	0.800
Total cholesterol in LDL (mmol/l)	896	0.01	-0.09	0.10	0.896	874	0.01	-0.09	0.11	0.881
Total cholesterol in HDL (mmol/l)	896	0.19	0.09	0.28	9.98E-05	874	0.14	0.04	0.23	0.005
Total cholesterol in HDL2 (mmol/l)	896	0.19	0.09	0.28	1.43E-04	874	0.13	0.04	0.23	0.007
Total cholesterol in HDL3 (mmol/l)	896	0.18	0.09	0.27	1.34E-04	874	0.14	0.04	0.23	0.005
Esterified cholesterol (mmol/l)	896	0.05	-0.04	0.14	0.286	874	0.05	-0.05	0.14	0.362
Free cholesterol (mmol/l)	896	0.05	-0.05	0.14	0.345	874	0.05	-0.05	0.14	0.365

S5 Table Associations of longer-term total physical activity (mean of CPM measures at age 12y, 14y, and 15y) with metabolic traits at age 15y in ALSPAC

	Mean of CPM at age 12y, 14y, 15y (per SD (132) higher)									
	Adj. for age, sex, ethnicity, maternal education, smoking, alcohol, mean wear time, wear month					*Additionally adj. for mean FMI*				
**Standardised outcome at age 15y**	**N**	**Beta**	**LCL**	**UCL**	**P-value**	**N**	**Beta**	**LCL**	**UCL**	**P-value**
Serum total triglycerides (mmol/l)	896	-0.13	-0.23	-0.02	0.020	874	-0.08	-0.19	0.02	0.107
Triglycerides in VLDL (mmol/l)	896	-0.15	-0.25	-0.04	0.007	874	-0.09	-0.20	0.01	0.071
Triglycerides in LDL (mmol/l)	896	0.01	-0.08	0.11	0.795	874	-0.01	-0.11	0.08	0.766
Triglycerides in HDL (mmol/l)	896	-0.02	-0.11	0.08	0.746	874	0.00	-0.10	0.09	0.947
Diacylglycerol (mmol/l)	864	-0.04	-0.14	0.06	0.472	843	0.00	-0.10	0.10	0.938
Ratio of diacylglycerol to triglycerides	864	0.01	-0.09	0.11	0.830	843	0.02	-0.09	0.12	0.745
Total phosphoglycerides (mmol/l)	896	0.13	0.03	0.22	0.007	874	0.10	0.01	0.20	0.032
Ratio of triglycerides to phosphoglycerides	896	-0.16	-0.27	-0.06	2.72E-03	874	-0.11	-0.21	0.00	0.044
Phosphatidylcholine and other cholines (mmol/l)	877	0.12	0.03	0.21	0.009	855	0.10	0.00	0.19	0.045
Total cholines (mmol/l)	893	0.12	0.03	0.22	0.007	871	0.10	0.01	0.19	0.039
Apolipoprotein A-I (g/l)	896	0.15	0.06	0.25	1.13E-03	874	0.12	0.02	0.21	0.015
Apolipoprotein B (g/l)	896	-0.08	-0.18	0.02	0.126	874	-0.05	-0.15	0.06	0.384
Ratio of apolipoprotein B to apolipoprotein A-I	896	-0.15	-0.25	-0.05	0.004	874	-0.10	-0.20	0.00	0.062
Total fatty acids (mmol/l)	896	0.02	-0.08	0.11	0.743	874	0.03	-0.07	0.12	0.565
Estimated description of fatty acid chain length, not actual carbon number	892	-0.05	-0.15	0.05	0.345	870	-0.05	-0.15	0.06	0.387
Estimated degree of unsaturation	895	-0.01	-0.11	0.09	0.790	873	-0.01	-0.11	0.09	0.863
22:6, docosahexaenoic acid (mmol/l)	896	0.03	-0.08	0.13	0.606	874	0.05	-0.06	0.15	0.386
18:2, linoleic acid (mmol/l)	893	0.03	-0.05	0.12	0.444	871	0.02	-0.07	0.12	0.596
Conjugated linoleic acid (mmol/l)	896	-0.09	-0.20	0.02	0.095	874	-0.11	-0.22	-0.01	0.039
Omega-3 fatty acids (mmol/l)	894	-0.03	-0.13	0.08	0.623	872	-0.02	-0.13	0.08	0.680
Omega-6 fatty acids (mmol/l)	895	0.04	-0.05	0.13	0.422	873	0.03	-0.06	0.13	0.461
Polyunsaturated fatty acids (mmol/l)	893	0.03	-0.06	0.12	0.462	871	0.03	-0.06	0.13	0.511
Monounsaturated fatty acids; 16:1, 18:1 (mmol/l)	893	-0.04	-0.14	0.06	0.475	871	-0.01	-0.11	0.09	0.882
Saturated fatty acids (mmol/l)	892	0.05	-0.05	0.15	0.339	870	0.05	-0.04	0.15	0.277
Ratio of 22:6 docosahexaenoic acid to total fatty acids (%)	896	0.03	-0.08	0.14	0.621	874	0.04	-0.07	0.15	0.432
Ratio of 18:2 linoleic acid to total fatty acids (%)	893	0.02	-0.08	0.13	0.660	871	-0.01	-0.12	0.09	0.844
Ratio of conjugated linoleic acid to total fatty acids (%)	896	-0.10	-0.21	0.01	0.066	874	-0.13	-0.24	-0.02	0.026
Ratio of omega-3 fatty acids to total fatty acids (%)	894	-0.03	-0.14	0.07	0.516	872	-0.04	-0.15	0.06	0.437
Ratio of omega-6 fatty acids to total fatty acids (%)	895	0.03	-0.07	0.14	0.519	873	0.01	-0.10	0.11	0.912
Ratio of polyunsaturated fatty acids to total fatty acids (%)	893	0.03	-0.08	0.13	0.604	871	0.00	-0.11	0.10	0.951
Ratio of monounsaturated fatty acids to total fatty acids (%)	893	-0.07	-0.18	0.03	0.177	871	-0.04	-0.15	0.07	0.476
Ratio of saturated fatty acids to total fatty acids (%)	892	0.06	-0.04	0.16	0.253	870	0.05	-0.05	0.16	0.342
Insulin (mu/l)	927	-0.14	-0.20	-0.08	1.31E-06	904	-0.08	-0.14	-0.02	0.007
Glucose (mmol/l)	894	-0.06	-0.15	0.03	0.169	872	-0.05	-0.14	0.04	0.288
Lactate (mmol/l)	894	-0.04	-0.14	0.06	0.403	872	-0.04	-0.15	0.06	0.399
Pyruvate (mmol/l)	893	-0.14	-0.24	-0.05	2.38E-03	871	-0.11	-0.21	-0.01	0.025
Citrate (mmol/l)	891	0.16	0.06	0.27	2.47E-03	869	0.12	0.01	0.23	0.026
Alanine (mmol/l)	896	-0.13	-0.22	-0.03	0.010	874	-0.13	-0.23	-0.03	0.011
Glutamine (mmol/l)	896	0.03	-0.06	0.12	0.483	874	-0.01	-0.10	0.08	0.800
Histidine (mmol/l)	850	0.08	-0.02	0.17	0.119	828	0.05	-0.05	0.15	0.335
Isoleucine (mmol/l)	896	-0.06	-0.15	0.03	0.163	874	-0.04	-0.13	0.05	0.379
Leucine (mmol/l)	896	0.05	-0.03	0.13	0.202	874	0.05	-0.03	0.14	0.217
Valine (mmol/l)	896	-0.01	-0.09	0.07	0.842	874	0.01	-0.08	0.09	0.858
Phenylalanine (mmol/l)	895	0.09	0.00	0.19	0.061	873	0.11	0.01	0.21	0.038
Tyrosine (mmol/l)	892	0.05	-0.05	0.15	0.355	870	0.08	-0.02	0.19	0.124
Acetate (mmol/l)	895	0.13	0.03	0.22	0.007	873	0.12	0.02	0.21	0.017
Acetoacetate (mmol/l)	896	-0.03	-0.11	0.05	0.459	874	-0.03	-0.11	0.06	0.510
3-hydroxybutyrate (mmol/l)	895	-0.05	-0.14	0.04	0.239	873	-0.04	-0.14	0.05	0.386
Creatinine (mmol/l)	895	-0.10	-0.19	-0.01	0.038	873	-0.11	-0.21	-0.02	0.022
Albumin (signal area)	896	-0.18	-0.28	-0.07	7.43E-04	874	-0.17	-0.28	-0.07	1.14E-03
Glycoprotein acetyls, mainly a1-acid glycoprotein (mmol/l)	895	-0.20	-0.29	-0.11	1.66E-05	873	-0.11	-0.20	-0.02	0.016
C-reactive protein (mg/l)	929	-0.03	-0.11	0.05	0.464	906	-0.02	-0.09	0.06	0.700

Complete case sample

	Mean of CPM at age 12y, 14y, 15y (per SD (132) higher)									
	Adj. for age, sex, ethnicity, maternal education, smoking, alcohol, mean wear time, wear month					*Additionally adj. for mean FMI*				
**Standardised outcome at age 15y**	**N**	**Beta**	**LCL**	**UCL**	**P-value**	**N**	**Beta**	**LCL**	**UCL**	**P-value**
Systolic blood pressure (mmHg)	755	-0.11	-0.22	-0.01	0.031	755	-0.07	-0.18	0.04	0.193
Diastolic blood pressure (mmHg)	755	-0.04	-0.15	0.06	0.409	755	-0.03	-0.14	0.08	0.564
Concentration of chylomicrons and extremely large VLDL particles (mol/l)	755	-0.14	-0.25	-0.04	0.008	755	-0.08	-0.18	0.02	0.122
Total lipids in chylomicrons and extremely large VLDL (mmol/l)	755	-0.13	-0.24	-0.03	0.012	755	-0.07	-0.17	0.03	0.148
Phospholipids in chylomicrons and extremely large VLDL (mmol/l)	755	-0.14	-0.24	-0.03	0.011	755	-0.08	-0.18	0.02	0.123
Total cholesterol in chylomicrons and extremely large VLDL (mmol/l)	755	-0.11	-0.21	0.00	0.042	755	-0.04	-0.14	0.05	0.367
Cholesterol esters in chylomicrons and extremely large VLDL (mmol/l)	755	-0.08	-0.18	0.03	0.142	755	-0.01	-0.11	0.09	0.800
Free cholesterol in chylomicrons and extremely large VLDL (mmol/l)	755	-0.13	-0.24	-0.03	0.012	755	-0.08	-0.18	0.02	0.131
Triglycerides in chylomicrons and extremely large VLDL (mmol/l)	755	-0.14	-0.24	-0.03	0.010	755	-0.08	-0.18	0.02	0.124
Concentration of very large VLDL particles (mol/l)	755	-0.12	-0.23	-0.02	0.021	755	-0.06	-0.16	0.04	0.217
Total lipids in very large VLDL (mmol/l)	755	-0.12	-0.22	-0.01	0.029	755	-0.06	-0.16	0.04	0.257
Phospholipids in very large VLDL (mmol/l)	755	-0.12	-0.22	-0.02	0.024	755	-0.06	-0.16	0.04	0.209
Total cholesterol in very large VLDL (mmol/l)	755	-0.12	-0.22	-0.01	0.026	755	-0.05	-0.15	0.04	0.283
Cholesterol esters in very large VLDL (mmol/l)	755	-0.11	-0.22	-0.01	0.037	755	-0.04	-0.14	0.06	0.392
Free cholesterol in very large VLDL (mmol/l)	755	-0.12	-0.23	-0.02	0.019	755	-0.07	-0.17	0.03	0.194
Triglycerides in very large VLDL (mmol/l)	755	-0.11	-0.22	-0.01	0.032	755	-0.06	-0.16	0.04	0.266
Concentration of large VLDL particles (mol/l)	755	-0.10	-0.21	0.00	0.056	755	-0.04	-0.14	0.06	0.404
Total lipids in large VLDL (mmol/l)	755	-0.10	-0.21	0.01	0.066	755	-0.04	-0.14	0.06	0.446
Phospholipids in large VLDL (mmol/l)	755	-0.10	-0.20	0.01	0.066	755	-0.04	-0.14	0.06	0.438
Total cholesterol in large VLDL (mmol/l)	755	-0.09	-0.19	0.02	0.113	755	-0.02	-0.12	0.08	0.655
Cholesterol esters in large VLDL (mmol/l)	755	-0.07	-0.18	0.04	0.196	755	0.00	-0.11	0.10	0.935
Free cholesterol in large VLDL (mmol/l)	755	-0.10	-0.20	0.01	0.065	755	-0.04	-0.14	0.06	0.428
Triglycerides in large VLDL (mmol/l)	755	-0.10	-0.21	0.00	0.054	755	-0.05	-0.15	0.06	0.383
Concentration of medium VLDL particles (mol/l)	755	-0.10	-0.21	0.01	0.073	755	-0.03	-0.14	0.07	0.511
Total lipids in medium VLDL (mmol/l)	755	-0.09	-0.20	0.02	0.102	755	-0.03	-0.13	0.08	0.633
Phospholipids in medium VLDL (mmol/l)	755	-0.09	-0.19	0.02	0.124	755	-0.02	-0.13	0.08	0.660
Total cholesterol in medium VLDL (mmol/l)	755	-0.05	-0.16	0.06	0.348	755	0.01	-0.09	0.12	0.824
Cholesterol esters in medium VLDL (mmol/l)	755	-0.02	-0.13	0.09	0.670	755	0.04	-0.07	0.15	0.450
Free cholesterol in medium VLDL (mmol/l)	755	-0.08	-0.19	0.03	0.132	755	-0.02	-0.13	0.08	0.648
Triglycerides in medium VLDL (mmol/l)	755	-0.11	-0.22	0.00	0.050	755	-0.04	-0.15	0.06	0.413

S5 Table Associations of longer-term total physical activity (mean of CPM measures at age 12y, 14y, and 15y) with metabolic traits at age 15y in ALSPAC

	**Mean of CPM at age 12y, 14y, 15y (per SD (132) higher)**									
	Adj. for age, sex, ethnicity, maternal education, smoking, alcohol, mean wear time, wear month					*Additionally adj. for mean FMI*				
**Standardised outcome at age 15y**	**N**	**Beta**	**LCL**	**UCL**	**P-value**	**N**	**Beta**	**LCL**	**UCL**	**P-value**
Concentration of small VLDL particles (mol/l)	755	-0.07	-0.18	0.04	0.206	755	-0.01	-0.11	0.10	0.890
Total lipids in small VLDL (mmol/l)	755	-0.06	-0.17	0.05	0.249	755	0.00	-0.11	0.11	0.978
Phospholipids in small VLDL (mmol/l)	755	-0.03	-0.14	0.07	0.550	755	0.03	-0.07	0.14	0.545
Total cholesterol in small VLDL (mmol/l)	755	-0.05	-0.16	0.06	0.393	755	0.02	-0.09	0.13	0.734
Cholesterol esters in small VLDL (mmol/l)	755	-0.05	-0.16	0.06	0.329	755	0.01	-0.10	0.12	0.823
Free cholesterol in small VLDL (mmol/l)	755	-0.03	-0.14	0.08	0.594	755	0.03	-0.08	0.14	0.580
Triglycerides in small VLDL (mmol/l)	755	-0.08	-0.19	0.03	0.134	755	-0.03	-0.13	0.08	0.621
Concentration of very small VLDL particles (mol/l)	755	0.02	-0.08	0.12	0.702	755	0.05	-0.05	0.15	0.347
Total lipids in very small VLDL (mmol/l)	755	-0.01	-0.12	0.09	0.800	755	0.03	-0.08	0.14	0.616
Phospholipids in very small VLDL (mmol/l)	755	0.02	-0.08	0.12	0.689	755	0.04	-0.07	0.14	0.492
Total cholesterol in very small VLDL (mmol/l)	755	-0.02	-0.13	0.09	0.734	755	0.03	-0.08	0.15	0.587
Cholesterol esters in very small VLDL (mmol/l)	755	-0.06	-0.16	0.05	0.322	755	0.00	-0.11	0.12	0.944
Free cholesterol in very small VLDL (mmol/l)	755	0.06	-0.05	0.17	0.264	755	0.09	-0.03	0.20	0.129
Triglycerides in very small VLDL (mmol/l)	755	-0.04	-0.14	0.06	0.415	755	-0.01	-0.11	0.09	0.852
Concentration of IDL particles (mol/l)	755	0.02	-0.08	0.12	0.735	755	0.02	-0.09	0.12	0.774
Total lipids in IDL (mmol/l)	755	0.02	-0.08	0.13	0.652	755	0.03	-0.08	0.14	0.591
Phospholipids in IDL (mmol/l)	755	0.03	-0.08	0.13	0.622	755	0.02	-0.08	0.13	0.685
Total cholesterol in IDL (mmol/l)	755	0.02	-0.08	0.13	0.672	755	0.04	-0.07	0.14	0.526
Cholesterol esters in IDL (mmol/l)	755	0.02	-0.09	0.12	0.767	755	0.04	-0.07	0.15	0.516
Free cholesterol in IDL (mmol/l)	755	0.04	-0.07	0.14	0.477	755	0.03	-0.08	0.14	0.573
Triglycerides in IDL (mmol/l)	755	0.02	-0.08	0.11	0.748	755	0.00	-0.10	0.09	0.976
Concentration of large LDL particles (mol/l)	755	0.02	-0.08	0.13	0.631	755	0.03	-0.08	0.13	0.630
Total lipids in large LDL (mmol/l)	755	0.03	-0.07	0.13	0.603	755	0.03	-0.07	0.13	0.568
Phospholipids in large LDL (mmol/l)	755	0.03	-0.07	0.13	0.603	755	0.04	-0.07	0.14	0.496
Total cholesterol in large LDL (mmol/l)	755	0.03	-0.08	0.13	0.609	755	0.03	-0.07	0.14	0.544
Cholesterol esters in large LDL (mmol/l)	755	0.02	-0.08	0.13	0.654	755	0.03	-0.07	0.14	0.550
Free cholesterol in large LDL (mmol/l)	755	0.04	-0.07	0.14	0.483	755	0.03	-0.07	0.14	0.530
Triglycerides in large LDL (mmol/l)	755	0.02	-0.08	0.12	0.682	755	-0.01	-0.11	0.09	0.866
Concentration of medium LDL particles (mol/l)	755	0.01	-0.09	0.11	0.817	755	0.02	-0.09	0.12	0.739
Total lipids in medium LDL (mmol/l)	755	0.02	-0.08	0.12	0.697	755	0.03	-0.08	0.13	0.611
Phospholipids in medium LDL (mmol/l)	755	0.03	-0.07	0.13	0.575	755	0.05	-0.05	0.15	0.362
Total cholesterol in medium LDL (mmol/l)	755	0.02	-0.09	0.12	0.753	755	0.02	-0.08	0.13	0.651
Cholesterol esters in medium LDL (mmol/l)	755	0.01	-0.09	0.11	0.845	755	0.02	-0.09	0.13	0.735
Free cholesterol in medium LDL (mmol/l)	755	0.04	-0.06	0.14	0.419	755	0.05	-0.06	0.16	0.360
Triglycerides in medium LDL (mmol/l)	755	0.02	-0.08	0.12	0.649	755	-0.01	-0.11	0.10	0.921
Concentration of small LDL particles (mol/l)	755	0.02	-0.08	0.12	0.723	755	0.02	-0.08	0.13	0.664
Total lipids in small LDL (mmol/l)	755	0.02	-0.08	0.12	0.689	755	0.03	-0.08	0.13	0.589
Phospholipids in small LDL (mmol/l)	755	0.04	-0.06	0.14	0.434	755	0.05	-0.05	0.15	0.331
Total cholesterol in small LDL (mmol/l)	755	0.02	-0.08	0.12	0.734	755	0.03	-0.08	0.13	0.631
Cholesterol esters in small LDL (mmol/l)	755	0.01	-0.09	0.11	0.862	755	0.02	-0.09	0.12	0.769
Free cholesterol in small LDL (mmol/l)	755	0.05	-0.05	0.16	0.309	755	0.07	-0.04	0.18	0.216
Triglycerides in small LDL (mmol/l)	755	-0.01	-0.11	0.09	0.840	755	-0.01	-0.11	0.09	0.814
Concentration of very large HDL particles (mol/l)	755	0.13	0.02	0.25	0.021	755	0.05	-0.06	0.17	0.342
Total lipids in very large HDL (mmol/l)	755	0.13	0.02	0.24	0.024	755	0.05	-0.06	0.16	0.363
Phospholipids in very large HDL (mmol/l)	755	0.14	0.03	0.25	0.015	755	0.06	-0.05	0.17	0.314
Total cholesterol in very large HDL (mmol/l)	755	0.12	0.00	0.23	0.047	755	0.05	-0.07	0.16	0.425
Cholesterol esters in very large HDL (mmol/l)	755	0.11	0.00	0.22	0.060	755	0.04	-0.07	0.16	0.447
Free cholesterol in very large HDL (mmol/l)	755	0.13	0.01	0.24	0.029	755	0.05	-0.06	0.16	0.388
Triglycerides in very large HDL (mmol/l)	755	0.04	-0.07	0.14	0.488	755	0.00	-0.11	0.10	0.933
Concentration of large HDL particles (mol/l)	755	0.18	0.06	0.29	2.18E-03	755	0.09	-0.02	0.20	0.095
Total lipids in large HDL (mmol/l)	755	0.17	0.06	0.29	2.27E-03	755	0.09	-0.02	0.20	0.103
Phospholipids in large HDL (mmol/l)	755	0.17	0.06	0.28	2.67E-03	755	0.09	-0.02	0.20	0.099
Total cholesterol in large HDL (mmol/l)	755	0.18	0.06	0.29	2.24E-03	755	0.09	-0.02	0.20	0.112
Cholesterol esters in large HDL (mmol/l)	755	0.18	0.06	0.29	2.18E-03	755	0.09	-0.02	0.20	0.112
Free cholesterol in large HDL (mmol/l)	755	0.17	0.06	0.28	2.59E-03	755	0.09	-0.02	0.20	0.116
Triglycerides in large HDL (mmol/l)	755	0.11	0.01	0.22	0.035	755	0.08	-0.03	0.19	0.151
Concentration of medium HDL particles (mol/l)	755	0.18	0.07	0.28	6.97E-04	755	0.16	0.05	0.26	2.87E-03
Total lipids in medium HDL (mmol/l)	755	0.18	0.08	0.28	7.04E-04	755	0.15	0.05	0.25	0.004
Phospholipids in medium HDL (mmol/l)	755	0.18	0.08	0.28	5.43E-04	755	0.15	0.05	0.26	0.004
Total cholesterol in medium HDL (mmol/l)	755	0.17	0.07	0.28	1.28E-03	755	0.14	0.03	0.24	0.011
Cholesterol esters in medium HDL (mmol/l)	755	0.17	0.07	0.28	1.56E-03	755	0.14	0.03	0.24	0.013
Free cholesterol in medium HDL (mmol/l)	755	0.17	0.07	0.27	1.26E-03	755	0.14	0.04	0.24	0.008
Triglycerides in medium HDL (mmol/l)	755	0.00	-0.11	0.10	0.935	755	0.05	-0.05	0.16	0.343
Concentration of small HDL particles (mol/l)	755	0.05	-0.05	0.15	0.299	755	0.08	-0.02	0.18	0.107
Total lipids in small HDL (mmol/l)	755	0.12	0.02	0.21	0.019	755	0.13	0.03	0.23	0.011
Phospholipids in small HDL (mmol/l)	755	0.03	-0.07	0.13	0.559	755	0.05	-0.05	0.15	0.317
Total cholesterol in small HDL (mmol/l)	755	0.18	0.09	0.28	1.57E-04	755	0.18	0.08	0.27	2.89E-04
Cholesterol esters in small HDL (mmol/l)	755	0.17	0.08	0.27	2.98E-04	755	0.17	0.07	0.26	4.43E-04
Free cholesterol in small HDL (mmol/l)	755	0.14	0.04	0.24	0.005	755	0.13	0.03	0.23	0.015
Triglycerides in small HDL (mmol/l)	755	-0.07	-0.17	0.03	0.186	755	-0.03	-0.13	0.07	0.553
Phospholipids to total lipids ratio in chylomicrons and extremely large VLDL (%)	755	-0.04	-0.15	0.06	0.439	755	-0.05	-0.16	0.06	0.387
Total cholesterol to total lipids ratio in chylomicrons and extremely large VLDL (%)	755	0.09	-0.03	0.21	0.125	755	0.13	0.01	0.25	0.027
Cholesterol esters to total lipids ratio in chylomicrons and extremely large VLDL (%)	755	0.12	0.01	0.24	0.034	755	0.16	0.05	0.28	0.006
Free cholesterol to total lipids ratio in chylomicrons and extremely large VLDL (%)	755	-0.04	-0.16	0.08	0.500	755	-0.01	-0.14	0.11	0.830
Triglycerides to total lipids ratio in chylomicrons and extremely large VLDL (%)	755	-0.05	-0.13	0.03	0.228	755	-0.08	-0.17	0.00	0.056
Phospholipids to total lipds ratio in very large VLDL (%)	755	-0.03	-0.15	0.08	0.576	755	0.00	-0.12	0.11	0.946
Total cholesterol to total lipids ratio in very large VLDL (%)	755	0.11	0.00	0.23	0.059	755	0.12	0.00	0.24	0.043
Cholesterol esters to total lipids ratio in very large VLDL (%)	755	0.13	0.01	0.25	0.035	755	0.12	0.00	0.25	0.045
Free cholesterol to total lipids ratio in very large VLDL (%)	755	0.12	-0.01	0.25	0.071	755	0.11	-0.02	0.23	0.111
Triglycerides to total lipids ratio in very large VLDL (%)	755	-0.13	-0.25	0.00	0.049	755	-0.13	-0.26	0.00	0.048
Phospholipids to total lipds ratio in large VLDL (%)	755	-0.05	-0.18	0.07	0.397	755	-0.03	-0.16	0.10	0.668
Total cholesterol to total lipids ratio in large VLDL (%)	755	0.04	-0.08	0.15	0.532	755	0.08	-0.04	0.19	0.216
Cholesterol esters to total lipids ratio in large VLDL (%)	755	0.04	-0.05	0.13	0.418	755	0.09	-0.01	0.19	0.091
Free cholesterol to total lipids ratio in large VLDL (%)	755	-0.06	-0.18	0.05	0.281	755	-0.03	-0.14	0.09	0.670
Triglycerides to total lipids ratio in large VLDL (%)	755	-0.02	-0.10	0.07	0.661	755	0.02	-0.07	0.12	0.620
Phospholipids to total lipds ratio in medium VLDL (%)	755	0.14	0.01	0.27	0.041	755	0.07	-0.06	0.20	0.275
Total cholesterol to total lipids ratio in medium VLDL (%)	755	0.12	0.01	0.23	0.032	755	0.13	0.02	0.24	0.025
Cholesterol esters to total lipids ratio in medium VLDL (%)	755	0.13	0.02	0.24	0.018	755	0.14	0.03	0.26	0.013
Free cholesterol to total lipids ratio in medium VLDL (%)	755	0.01	-0.10	0.12	0.809	755	0.01	-0.10	0.12	0.857
Triglycerides to total lipids ratio in medium VLDL (%)	755	-0.14	-0.25	-0.03	0.016	755	-0.13	-0.25	-0.02	0.023
Phospholipids to total lipds ratio in small VLDL (%)	755	0.16	0.05	0.27	0.004	755	0.12	0.01	0.23	0.030

S5 Table Associations of longer-term total physical activity (mean of CPM measures at age 12y, 14y, and 15y) with metabolic traits at age 15y in ALSPAC

	**Mean of CPM at age 12y, 14y, 15y (per SD (132) higher)**									
	Adj. for age, sex, ethnicity, maternal education, smoking, alcohol, mean wear time, wear month					*Additionally adj. for mean FMI*				
**Standardised outcome at age 15y**	**N**	**Beta**	**LCL**	**UCL**	**P-value**	**N**	**Beta**	**LCL**	**UCL**	**P-value**
Total cholesterol to total lipids ratio in small VLDL (%)	755	0.04	-0.07	0.16	0.449	755	0.05	-0.07	0.16	0.435
Cholesterol esters to total lipids ratio in small VLDL (%)	755	0.01	-0.10	0.12	0.878	755	0.02	-0.09	0.13	0.726
Free cholesterol to total lipids ratio in small VLDL (%)	755	0.23	0.11	0.34	1.69E-04	755	0.18	0.06	0.29	0.003
Triglycerides to total lipids ratio in small VLDL (%)	755	-0.09	-0.20	0.02	0.117	755	-0.08	-0.20	0.03	0.167
Phospholipids to total lipds ratio in very small VLDL (%)	755	0.07	-0.03	0.18	0.161	755	0.04	-0.07	0.14	0.492
Total cholesterol to total lipids ratio in very small VLDL (%)	755	-0.01	-0.12	0.09	0.841	755	0.02	-0.09	0.12	0.785
Cholesterol esters to total lipids ratio in very small VLDL (%)	755	-0.09	-0.19	0.01	0.064	755	-0.05	-0.15	0.05	0.284
Free cholesterol to total lipids ratio in very small VLDL (%)	755	0.17	0.06	0.28	2.87E-03	755	0.15	0.03	0.28	0.012
Triglycerides to total lipids ratio in very small VLDL (%)	755	-0.04	-0.15	0.07	0.468	755	-0.04	-0.16	0.07	0.443
Phospholipids to total lipds ratio in IDL (%)	755	0.01	-0.11	0.14	0.836	755	-0.04	-0.17	0.09	0.535
Total cholesterol to total lipids ratio in IDL (%)	755	-0.01	-0.12	0.11	0.925	755	0.04	-0.08	0.15	0.525
Cholesterol esters to total lipids ratio in IDL (%)	755	-0.03	-0.15	0.08	0.565	755	0.03	-0.09	0.15	0.615
Free cholesterol to total lipids ratio in IDL (%)	755	0.06	-0.04	0.17	0.234	755	0.02	-0.09	0.12	0.763
Triglycerides to total lipids ratio in IDL (%)	755	0.00	-0.11	0.11	0.994	755	-0.03	-0.14	0.08	0.635
Phospholipids to total lipds ratio in large LDL (%)	755	-0.03	-0.12	0.07	0.597	755	-0.01	-0.11	0.09	0.826
Total cholesterol to total lipids ratio in large LDL (%)	755	0.02	-0.08	0.12	0.744	755	0.03	-0.07	0.14	0.552
Cholesterol esters to total lipids ratio in large LDL (%)	755	0.01	-0.09	0.11	0.828	755	0.03	-0.07	0.14	0.551
Free cholesterol to total lipids ratio in large LDL (%)	755	0.01	-0.08	0.10	0.839	755	-0.01	-0.11	0.08	0.775
Triglycerides to total lipids ratio in large LDL (%)	755	0.00	-0.10	0.11	0.980	755	-0.04	-0.15	0.07	0.449
Phospholipids to total lipds ratio in medium LDL (%)	755	-0.01	-0.04	0.03	0.720	755	0.00	-0.04	0.04	0.931
Total cholesterol to total lipids ratio in medium LDL (%)	755	0.00	-0.10	0.11	0.948	755	0.01	-0.10	0.12	0.856
Cholesterol esters to total lipids ratio in medium LDL (%)	755	0.01	-0.10	0.11	0.898	755	0.01	-0.10	0.12	0.830
Free cholesterol to total lipids ratio in medium LDL (%)	755	0.00	-0.03	0.03	0.842	755	0.00	-0.03	0.03	0.811
Triglycerides to total lipids ratio in medium LDL (%)	755	0.03	-0.07	0.14	0.538	755	-0.01	-0.12	0.09	0.808
Phospholipids to total lipds ratio in small LDL (%)	755	-0.01	-0.07	0.05	0.862	755	-0.01	-0.07	0.06	0.863
Total cholesterol to total lipids ratio in small LDL (%)	755	0.01	-0.09	0.12	0.806	755	0.02	-0.09	0.13	0.758
Cholesterol esters to total lipids ratio in small LDL (%)	755	0.01	-0.10	0.12	0.863	755	0.01	-0.10	0.12	0.842
Free cholesterol to total lipids ratio in small LDL (%)	755	0.00	-0.06	0.05	0.987	755	0.00	-0.06	0.06	0.978
Triglycerides to total lipids ratio in small LDL (%)	755	-0.01	-0.12	0.09	0.783	755	-0.02	-0.13	0.08	0.649
Phospholipids to total lipds ratio in very large HDL (%)	755	0.11	0.00	0.22	0.050	755	0.03	-0.08	0.14	0.562
Total cholesterol to total lipids ratio in very large HDL (%)	755	-0.10	-0.20	0.00	0.061	755	-0.03	-0.13	0.08	0.618
Cholesterol esters to total lipids ratio in very large HDL (%)	755	-0.10	-0.20	0.01	0.066	755	-0.02	-0.13	0.08	0.647
Free cholesterol to total lipids ratio in very large HDL (%)	755	0.03	-0.08	0.14	0.623	755	-0.01	-0.12	0.10	0.869
Triglycerides to total lipids ratio in very large HDL (%)	755	-0.07	-0.20	0.05	0.231	755	-0.04	-0.16	0.08	0.552
Phospholipids to total lipds ratio in large HDL (%)	755	-0.09	-0.20	0.01	0.083	755	-0.01	-0.11	0.09	0.806
Total cholesterol to total lipids ratio in large HDL (%)	755	0.10	-0.01	0.22	0.072	755	0.02	-0.09	0.13	0.756
Cholesterol esters to total lipids ratio in large HDL (%)	755	0.10	-0.01	0.22	0.070	755	0.02	-0.09	0.13	0.768
Free cholesterol to total lipids ratio in large HDL (%)	755	0.08	-0.03	0.19	0.164	755	0.02	-0.09	0.12	0.768
Triglycerides to total lipids ratio in large HDL (%)	755	-0.10	-0.22	0.02	0.117	755	-0.02	-0.14	0.10	0.722
Phospholipids to total lipds ratio in medium HDL (%)	755	0.13	0.02	0.24	0.020	755	0.11	0.00	0.22	0.061
Total cholesterol to total lipids ratio in medium HDL (%)	755	-0.05	-0.16	0.06	0.350	755	-0.08	-0.19	0.04	0.181
Cholesterol esters to total lipids ratio in medium HDL (%)	755	-0.05	-0.16	0.06	0.405	755	-0.07	-0.19	0.04	0.219
Free cholesterol to total lipids ratio in medium HDL (%)	755	-0.04	-0.19	0.11	0.607	755	-0.04	-0.19	0.11	0.569
Triglycerides to total lipids ratio in medium HDL (%)	755	-0.08	-0.20	0.03	0.163	755	-0.01	-0.13	0.10	0.825
Phospholipids to total lipds ratio in small HDL (%)	755	-0.16	-0.26	-0.07	9.02E-04	755	-0.15	-0.25	-0.05	0.003
Total cholesterol to total lipids ratio in small HDL (%)	755	0.18	0.09	0.28	2.30E-04	755	0.16	0.06	0.26	1.52E-03
Cholesterol esters to total lipids ratio in small HDL (%)	755	0.16	0.07	0.26	9.25E-04	755	0.15	0.05	0.25	2.83E-03
Free cholesterol to total lipids ratio in small HDL (%)	755	0.08	-0.03	0.18	0.155	755	0.01	-0.10	0.12	0.828
Triglycerides to total lipids ratio in small HDL (%)	755	-0.14	-0.24	-0.03	0.012	755	-0.10	-0.20	0.01	0.063
Mean diameter for VLDL particles (nm)	755	-0.12	-0.23	0.00	0.041	755	-0.06	-0.17	0.05	0.284
Mean diameter for LDL particles (nm)	755	-0.02	-0.11	0.07	0.714	755	-0.04	-0.13	0.06	0.443
Mean diameter for HDL particles (nm)	755	0.14	0.03	0.26	0.016	755	0.06	-0.06	0.17	0.334
Serum total cholesterol (mmol/l)	755	0.07	-0.03	0.17	0.198	755	0.06	-0.04	0.17	0.220
Total cholesterol in VLDL (mmol/l)	755	-0.06	-0.17	0.05	0.277	755	0.01	-0.10	0.12	0.858
Remnant cholesterol (non-HDL, non-LDL -cholesterol) (mmol/l)	755	-0.02	-0.13	0.08	0.649	755	0.02	-0.09	0.13	0.672
Total cholesterol in LDL (mmol/l)	755	0.02	-0.08	0.12	0.675	755	0.03	-0.08	0.14	0.591
Total cholesterol in HDL (mmol/l)	755	0.19	0.08	0.30	7.53E-04	755	0.12	0.01	0.23	0.029
Total cholesterol in HDL2 (mmol/l)	755	0.18	0.07	0.29	1.38E-03	755	0.11	0.00	0.22	0.049
Total cholesterol in HDL3 (mmol/l)	755	0.19	0.08	0.29	4.18E-04	755	0.13	0.03	0.24	0.014
Esterified cholesterol (mmol/l)	755	0.07	-0.03	0.17	0.165	755	0.07	-0.04	0.17	0.200
Free cholesterol (mmol/l)	755	0.05	-0.05	0.15	0.322	755	0.06	-0.05	0.16	0.298
Serum total triglycerides (mmol/l)	755	-0.09	-0.19	0.02	0.109	755	-0.04	-0.14	0.07	0.481
Triglycerides in VLDL (mmol/l)	755	-0.10	-0.21	0.00	0.060	755	-0.04	-0.15	0.06	0.419
Triglycerides in LDL (mmol/l)	755	0.02	-0.08	0.11	0.750	755	-0.01	-0.11	0.09	0.872
Triglycerides in HDL (mmol/l)	755	0.00	-0.10	0.10	0.985	755	0.02	-0.08	0.12	0.674
Diacylglycerol (mmol/l)	755	0.00	-0.10	0.09	0.956	755	0.03	-0.07	0.13	0.531
Ratio of diacylglycerol to triglycerides	755	0.02	-0.09	0.13	0.744	755	0.03	-0.08	0.14	0.631
Total phosphoglycerides (mmol/l)	755	0.14	0.04	0.24	0.007	755	0.11	0.01	0.21	0.036
Ratio of triglycerides to phosphoglycerides	755	-0.11	-0.22	-0.01	0.035	755	-0.05	-0.15	0.05	0.338
Phosphatidylcholine and other cholines (mmol/l)	755	0.13	0.04	0.23	0.007	755	0.11	0.01	0.21	0.032
Total cholines (mmol/l)	755	0.14	0.04	0.24	0.007	755	0.11	0.01	0.21	0.032
Apolipoprotein A-I (g/l)	755	0.16	0.06	0.27	1.99E-03	755	0.11	0.01	0.22	0.032
Apolipoprotein B (g/l)	755	-0.05	-0.15	0.06	0.375	755	0.00	-0.11	0.10	0.967
Ratio of apolipoprotein B to apolipoprotein A-I	755	-0.12	-0.24	-0.01	0.038	755	-0.05	-0.16	0.06	0.389
Total fatty acids (mmol/l)	755	0.02	-0.07	0.12	0.625	755	0.04	-0.06	0.14	0.434
Estimated description of fatty acid chain length, not actual carbon number	755	-0.03	-0.13	0.07	0.598	755	-0.01	-0.11	0.09	0.878
Estimated degree of unsaturation	755	0.00	-0.10	0.10	0.980	755	0.01	-0.10	0.11	0.923
22:6, docosahexaenoic acid (mmol/l)	755	0.01	-0.10	0.12	0.850	755	0.02	-0.09	0.13	0.706
18:2, linoleic acid (mmol/l)	755	0.07	-0.02	0.16	0.143	755	0.06	-0.04	0.16	0.227
Conjugated linoleic acid (mmol/l)	755	0.01	-0.08	0.11	0.797	755	0.01	-0.09	0.10	0.897
Omega-3 fatty acids (mmol/l)	755	-0.01	-0.12	0.09	0.781	755	-0.01	-0.12	0.10	0.847
Omega-6 fatty acids (mmol/l)	755	0.06	-0.03	0.16	0.202	755	0.06	-0.04	0.16	0.227
Polyunsaturated fatty acids (mmol/l)	755	0.06	-0.04	0.15	0.264	755	0.05	-0.05	0.15	0.287
Monounsaturated fatty acids; 16:1, 18:1 (mmol/l)	755	-0.01	-0.11	0.08	0.775	755	0.02	-0.08	0.12	0.662
Saturated fatty acids (mmol/l)	755	0.02	-0.08	0.12	0.629	755	0.03	-0.07	0.13	0.545
Ratio of 22:6 docosahexaenoic acid to total fatty acids (%)	755	0.00	-0.12	0.12	0.966	755	0.01	-0.11	0.13	0.914
Ratio of 18:2 linoleic acid to total fatty acids (%)	755	0.07	-0.03	0.17	0.179	755	0.03	-0.08	0.14	0.568
Ratio of conjugated linoleic acid to total fatty acids (%)	755	0.00	-0.10	0.10	0.934	755	-0.01	-0.11	0.09	0.831
Ratio of omega-3 fatty acids to total fatty acids (%)	755	-0.04	-0.15	0.07	0.463	755	-0.05	-0.16	0.06	0.382
Ratio of omega-6 fatty acids to total fatty acids (%)	755	0.06	-0.04	0.17	0.232	755	0.03	-0.08	0.14	0.572
Ratio of polyunsaturated fatty acids to total fatty acids (%)	755	0.05	-0.05	0.16	0.331	755	0.02	-0.09	0.13	0.738

S5 Table Associations of longer-term total physical activity (mean of CPM measures at age 12y, 14y, and 15y) with metabolic traits at age 15y in ALSPAC

	**Mean of CPM at age 12y, 14y, 15y (per SD (132) higher)**									
	Adj. for age, sex, ethnicity, maternal education, smoking, alcohol, mean wear time, wear month					*Additionally adj. for mean FMI*				
**Standardised outcome at age 15y**	**N**	**Beta**	**LCL**	**UCL**	**P-value**	**N**	**Beta**	**LCL**	**UCL**	**P-value**
Ratio of monounsaturated fatty acids to total fatty acids (%)	755	-0.05	-0.16	0.06	0.348	755	-0.01	-0.12	0.11	0.908
Ratio of saturated fatty acids to total fatty acids (%)	755	0.01	-0.10	0.11	0.914	755	-0.01	-0.12	0.09	0.801
Insulin (mu/l)	755	-0.13	-0.20	-0.06	1.16E-04	755	-0.07	-0.13	0.00	0.048
Glucose (mmol/l)	755	-0.04	-0.14	0.05	0.370	755	-0.04	-0.14	0.06	0.470
Lactate (mmol/l)	755	-0.05	-0.17	0.06	0.368	755	-0.07	-0.18	0.05	0.271
Pyruvate (mmol/l)	755	-0.14	-0.25	-0.03	0.013	755	-0.12	-0.23	-0.01	0.037
Citrate (mmol/l)	755	0.16	0.04	0.27	0.008	755	0.12	0.00	0.23	0.045
Alanine (mmol/l)	755	-0.13	-0.24	-0.03	0.015	755	-0.14	-0.25	-0.03	0.014
Glutamine (mmol/l)	755	0.04	-0.05	0.14	0.388	755	0.00	-0.10	0.09	0.938
Histidine (mmol/l)	755	0.04	-0.06	0.15	0.437	755	0.04	-0.07	0.15	0.516
Isoleucine (mmol/l)	755	-0.03	-0.13	0.07	0.552	755	0.00	-0.10	0.10	0.999
Leucine (mmol/l)	755	0.07	-0.02	0.17	0.141	755	0.08	-0.02	0.18	0.109
Valine (mmol/l)	755	0.01	-0.09	0.10	0.905	755	0.03	-0.07	0.13	0.517
Phenylalanine (mmol/l)	755	0.11	-0.01	0.22	0.068	755	0.13	0.02	0.25	0.023
Tyrosine (mmol/l)	755	0.08	-0.04	0.19	0.191	755	0.11	-0.01	0.22	0.085
Acetate (mmol/l)	755	0.14	0.04	0.25	0.008	755	0.13	0.03	0.24	0.014
Acetoacetate (mmol/l)	755	-0.06	-0.15	0.03	0.176	755	-0.05	-0.14	0.04	0.276
3-hydroxybutyrate (mmol/l)	755	-0.09	-0.19	0.01	0.083	755	-0.08	-0.19	0.02	0.134
Creatinine (mmol/l)	755	-0.09	-0.19	0.01	0.083	755	-0.09	-0.19	0.02	0.096
Albumin (signal area)	755	-0.15	-0.25	-0.04	0.005	755	-0.16	-0.27	-0.05	0.003
Glycoprotein acetyls, mainly a1-acid glycoprotein (mmol/l)	755	-0.19	-0.29	-0.09	2.78E-04	755	-0.10	-0.20	0.00	0.062
C-reactive protein (mg/l)	755	-0.08	-0.16	0.00	0.059	755	-0.06	-0.16	0.03	0.178
