# Supplementary material for: Associations of device-measured physical activity across adolescence with metabolic traits: Prospective cohort study
Source: PLoS Med. 2018 Sep 11;15(9):e1002649. doi: 10.1371/journal.pmed.1002649 (PMC6133272; doi:10.1371/journal.pmed.1002649)
Supplement: S6 Table — ALSPAC, Avon Longitudinal Study of Parents and Children; MVPA, moderate-to-vigorous physical activity. (PDF) [file pmed.1002649.s006.pdf]

S6 Table Associations of longer-term moderate-to-vigorous physical activity (mean of MVPA measures at age 12y, 14y, and 15y) with metabolic traits at age 15y in ALSPAC

Mean of MVPA at age 12y, 14y, 15y (per SD (12 min/day) higher)

	Adj. for age, sex, ethnicity, maternal education, smoking, alcohol, mean wear time, wear month					*Additionally adj. for mean SED*					*Additionally adj. for mean FMI*				
**Standardised outcome at age 15y**	**N**	**Beta**	**LCL**	**UCL**	**P-value**	**N**	**Beta**	**LCL**	**UCL**	**P-value**	**N**	**Beta**	**LCL**	**UCL**	**P-value**
Systolic blood pressure (mmHg)	1298	-0.14	-0.22	-0.06	5.28E-04	1298	-0.15	-0.23	-0.06	6.97E-04	1256	-0.09	-0.18	-0.01	0.028
Diastolic blood pressure (mmHg)	1298	-0.05	-0.13	0.03	0.255	1298	-0.02	-0.11	0.07	0.657	1256	-0.02	-0.11	0.07	0.686
Concentration of chylomicrons and extremely large VLDL particles (mol/l)	896	-0.21	-0.30	-0.11	2.79E-05	896	-0.21	-0.31	-0.11	6.24E-05	874	-0.18	-0.28	-0.08	3.18E-04
Total lipids in chylomicrons and extremely large VLDL (mmol/l)	896	-0.20	-0.30	-0.10	5.14E-05	896	-0.20	-0.30	-0.10	1.20E-04	874	-0.18	-0.28	-0.08	4.95E-04
Phospholipids in chylomicrons and extremely large VLDL (mmol/l)	896	-0.21	-0.30	-0.11	3.51E-05	896	-0.20	-0.31	-0.10	8.52E-05	874	-0.19	-0.29	-0.09	3.21E-04
Total cholesterol in chylomicrons and extremely large VLDL (mmol/l)	896	-0.17	-0.27	-0.07	6.33E-04	896	-0.17	-0.27	-0.07	7.09E-04	874	-0.15	-0.25	-0.05	0.003
Cholesterol esters in chylomicrons and extremely large VLDL (mmol/l)	896	-0.13	-0.23	-0.04	0.007	896	-0.14	-0.24	-0.04	0.006	874	-0.11	-0.21	-0.01	0.024
Free cholesterol in chylomicrons and extremely large VLDL (mmol/l)	896	-0.20	-0.30	-0.10	5.16E-05	896	-0.20	-0.30	-0.10	1.21E-04	874	-0.18	-0.28	-0.08	4.25E-04
Triglycerides in chylomicrons and extremely large VLDL (mmol/l)	896	-0.21	-0.30	-0.11	3.22E-05	896	-0.20	-0.30	-0.10	9.10E-05	874	-0.18	-0.28	-0.08	3.76E-04
Concentration of very large VLDL particles (mol/l)	896	-0.19	-0.29	-0.09	1.23E-04	896	-0.19	-0.29	-0.08	3.09E-04	874	-0.16	-0.26	-0.06	1.36E-03
Total lipids in very large VLDL (mmol/l)	896	-0.19	-0.28	-0.09	1.90E-04	896	-0.18	-0.28	-0.08	4.32E-04	874	-0.16	-0.26	-0.06	1.77E-03
Phospholipids in very large VLDL (mmol/l)	896	-0.19	-0.28	-0.09	1.67E-04	896	-0.19	-0.29	-0.08	3.18E-04	874	-0.17	-0.27	-0.07	1.15E-03
Total cholesterol in very large VLDL (mmol/l)	896	-0.19	-0.28	-0.09	1.97E-04	896	-0.18	-0.29	-0.08	3.24E-04	874	-0.16	-0.26	-0.06	1.65E-03
Cholesterol esters in very large VLDL (mmol/l)	896	-0.18	-0.28	-0.08	3.64E-04	896	-0.18	-0.28	-0.08	6.16E-04	874	-0.15	-0.24	-0.05	0.004
Free cholesterol in very large VLDL (mmol/l)	896	-0.19	-0.29	-0.09	1.16E-04	896	-0.19	-0.29	-0.09	1.86E-04	874	-0.17	-0.27	-0.07	7.45E-04
Triglycerides in very large VLDL (mmol/l)	896	-0.18	-0.28	-0.09	2.10E-04	896	-0.18	-0.28	-0.08	5.46E-04	874	-0.16	-0.26	-0.06	2.14E-03
Concentration of large VLDL particles (mol/l)	896	-0.17	-0.27	-0.08	4.85E-04	896	-0.17	-0.27	-0.07	1.07E-03	874	-0.14	-0.25	-0.04	0.005
Total lipids in large VLDL (mmol/l)	896	-0.17	-0.27	-0.07	6.31E-04	896	-0.17	-0.27	-0.07	1.27E-03	874	-0.14	-0.24	-0.04	0.006
Phospholipids in large VLDL (mmol/l)	896	-0.17	-0.27	-0.07	6.00E-04	896	-0.17	-0.27	-0.07	1.10E-03	874	-0.15	-0.25	-0.04	0.005
Total cholesterol in large VLDL (mmol/l)	896	-0.16	-0.26	-0.06	1.52E-03	896	-0.16	-0.26	-0.06	2.01E-03	874	-0.13	-0.23	-0.03	0.009
Cholesterol esters in large VLDL (mmol/l)	896	-0.14	-0.24	-0.05	0.004	896	-0.15	-0.25	-0.05	0.004	874	-0.12	-0.22	-0.02	0.022
Free cholesterol in large VLDL (mmol/l)	896	-0.17	-0.27	-0.07	6.22E-04	896	-0.17	-0.27	-0.07	1.04E-03	874	-0.15	-0.25	-0.05	0.004
Triglycerides in large VLDL (mmol/l)	896	-0.17	-0.27	-0.08	4.75E-04	896	-0.17	-0.27	-0.07	1.17E-03	874	-0.14	-0.25	-0.04	0.005
Concentration of medium VLDL particles (mol/l)	896	-0.17	-0.27	-0.07	5.54E-04	896	-0.17	-0.27	-0.07	1.08E-03	874	-0.14	-0.24	-0.04	0.006
Total lipids in medium VLDL (mmol/l)	896	-0.16	-0.26	-0.07	1.01E-03	896	-0.16	-0.27	-0.06	1.68E-03	874	-0.14	-0.24	-0.03	0.009
Phospholipids in medium VLDL (mmol/l)	896	-0.16	-0.26	-0.06	1.18E-03	896	-0.17	-0.27	-0.06	1.54E-03	874	-0.14	-0.24	-0.04	0.007
Total cholesterol in medium VLDL (mmol/l)	896	-0.12	-0.22	-0.03	0.013	896	-0.13	-0.24	-0.03	0.011	874	-0.10	-0.21	0.00	0.046
Cholesterol esters in medium VLDL (mmol/l)	896	-0.09	-0.19	0.01	0.075	896	-0.10	-0.20	0.00	0.055	874	-0.07	-0.17	0.03	0.193
Free cholesterol in medium VLDL (mmol/l)	896	-0.16	-0.26	-0.06	1.42E-03	896	-0.16	-0.27	-0.06	1.79E-03	874	-0.14	-0.24	-0.04	0.007
Triglycerides in medium VLDL (mmol/l)	896	-0.18	-0.28	-0.08	3.15E-04	896	-0.17	-0.28	-0.07	8.55E-04	874	-0.14	-0.25	-0.04	0.005
Concentration of small VLDL particles (mol/l)	896	-0.16	-0.25	-0.06	1.48E-03	896	-0.17	-0.28	-0.07	9.35E-04	874	-0.14	-0.25	-0.04	0.006
Total lipids in small VLDL (mmol/l)	896	-0.15	-0.25	-0.06	1.95E-03	896	-0.17	-0.28	-0.07	8.84E-04	874	-0.14	-0.25	-0.04	0.007
Phospholipids in small VLDL (mmol/l)	896	-0.13	-0.22	-0.03	0.008	896	-0.16	-0.26	-0.06	2.21E-03	874	-0.12	-0.23	-0.02	0.019
Total cholesterol in small VLDL (mmol/l)	896	-0.13	-0.22	-0.03	0.010	896	-0.16	-0.26	-0.06	2.53E-03	874	-0.12	-0.23	-0.02	0.022
Cholesterol esters in small VLDL (mmol/l)	896	-0.12	-0.22	-0.03	0.013	896	-0.15	-0.26	-0.05	0.003	874	-0.12	-0.22	-0.01	0.029
Free cholesterol in small VLDL (mmol/l)	896	-0.12	-0.22	-0.03	0.013	896	-0.15	-0.25	-0.05	0.004	874	-0.12	-0.23	-0.02	0.021
Triglycerides in small VLDL (mmol/l)	896	-0.16	-0.26	-0.07	9.60E-04	896	-0.17	-0.28	-0.07	1.16E-03	874	-0.15	-0.25	-0.04	0.005
Concentration of very small VLDL particles (mol/l)	896	-0.04	-0.13	0.05	0.420	896	-0.08	-0.17	0.02	0.135	874	-0.06	-0.16	0.04	0.246
Total lipids in very small VLDL (mmol/l)	896	-0.06	-0.16	0.03	0.181	896	-0.10	-0.20	0.00	0.060	874	-0.08	-0.18	0.03	0.162
Phospholipids in very small VLDL (mmol/l)	896	-0.03	-0.12	0.06	0.497	896	-0.07	-0.17	0.02	0.139	874	-0.07	-0.17	0.03	0.182
Total cholesterol in very small VLDL (mmol/l)	896	-0.04	-0.14	0.05	0.390	896	-0.06	-0.17	0.04	0.237	874	-0.03	-0.14	0.08	0.589
Cholesterol esters in very small VLDL (mmol/l)	896	-0.08	-0.18	0.02	0.114	896	-0.10	-0.20	0.01	0.074	874	-0.06	-0.17	0.05	0.295
Free cholesterol in very small VLDL (mmol/l)	896	0.04	-0.06	0.13	0.440	896	0.01	-0.09	0.12	0.824	874	0.03	-0.08	0.14	0.567
Triglycerides in very small VLDL (mmol/l)	896	-0.12	-0.22	-0.03	0.009	896	-0.15	-0.25	-0.05	2.79E-03	874	-0.14	-0.25	-0.04	0.006
Concentration of IDL particles (mol/l)	896	-0.03	-0.12	0.06	0.514	896	-0.07	-0.17	0.03	0.145	874	-0.09	-0.19	0.02	0.107
Total lipids in IDL (mmol/l)	896	-0.03	-0.12	0.07	0.587	896	-0.07	-0.17	0.03	0.171	874	-0.07	-0.17	0.03	0.174
Phospholipids in IDL (mmol/l)	896	-0.02	-0.12	0.07	0.619	896	-0.07	-0.17	0.03	0.167	874	-0.08	-0.18	0.02	0.130
Total cholesterol in IDL (mmol/l)	896	-0.02	-0.12	0.07	0.621	896	-0.06	-0.16	0.04	0.212	874	-0.06	-0.16	0.04	0.259

S6 Table Associations of longer-term moderate-to-vigorous physical activity (mean of MVPA measures at age 12y, 14y, and 15y) with metabolic traits at age 15y in ALSPAC

Mean of MVPA at age 12y, 14y, 15y (per SD (12 min/day) higher)

	Adj. for age, sex, ethnicity, maternal education, smoking, alcohol, mean wear time, wear month					*Additionally adj. for mean SED*					*Additionally adj. for mean FMI*				
**Standardised outcome at age 15y**	**N**	**Beta**	**LCL**	**UCL**	**P-value**	**N**	**Beta**	**LCL**	**UCL**	**P-value**	**N**	**Beta**	**LCL**	**UCL**	**P-value**
Cholesterol esters in IDL (mmol/l)	896	-0.03	-0.13	0.06	0.489	896	-0.07	-0.17	0.03	0.164	874	-0.06	-0.17	0.04	0.244
Free cholesterol in IDL (mmol/l)	896	0.00	-0.09	0.09	0.999	896	-0.04	-0.14	0.06	0.396	874	-0.05	-0.15	0.05	0.323
Triglycerides in IDL (mmol/l)	896	-0.03	-0.12	0.05	0.455	896	-0.07	-0.17	0.02	0.123	874	-0.09	-0.19	0.00	0.057
Concentration of large LDL particles (mol/l)	896	-0.04	-0.13	0.05	0.389	896	-0.09	-0.19	0.01	0.064	874	-0.10	-0.20	0.00	0.055
Total lipids in large LDL (mmol/l)	896	-0.03	-0.12	0.06	0.483	896	-0.08	-0.18	0.02	0.099	874	-0.09	-0.19	0.02	0.097
Phospholipids in large LDL (mmol/l)	896	-0.04	-0.13	0.05	0.405	896	-0.09	-0.19	0.01	0.069	874	-0.09	-0.19	0.01	0.083
Total cholesterol in large LDL (mmol/l)	896	-0.03	-0.12	0.06	0.509	896	-0.08	-0.18	0.02	0.115	874	-0.08	-0.18	0.02	0.123
Cholesterol esters in large LDL (mmol/l)	896	-0.04	-0.13	0.05	0.426	896	-0.09	-0.18	0.01	0.090	874	-0.09	-0.19	0.02	0.102
Free cholesterol in large LDL (mmol/l)	896	-0.01	-0.10	0.08	0.806	896	-0.06	-0.16	0.04	0.232	874	-0.07	-0.17	0.04	0.210
Triglycerides in large LDL (mmol/l)	896	-0.02	-0.11	0.06	0.591	896	-0.07	-0.16	0.03	0.168	874	-0.10	-0.19	0.00	0.050
Concentration of medium LDL particles (mol/l)	896	-0.06	-0.16	0.03	0.177	896	-0.12	-0.22	-0.02	0.020	874	-0.12	-0.23	-0.02	0.020
Total lipids in medium LDL (mmol/l)	896	-0.05	-0.14	0.04	0.298	896	-0.10	-0.20	0.00	0.044	874	-0.10	-0.21	0.00	0.048
Phospholipids in medium LDL (mmol/l)	896	-0.05	-0.14	0.04	0.262	896	-0.11	-0.20	-0.01	0.031	874	-0.10	-0.20	0.00	0.055
Total cholesterol in medium LDL (mmol/l)	896	-0.05	-0.14	0.04	0.312	896	-0.10	-0.20	0.00	0.055	874	-0.10	-0.20	0.00	0.062
Cholesterol esters in medium LDL (mmol/l)	896	-0.05	-0.14	0.04	0.265	896	-0.10	-0.20	0.00	0.047	874	-0.10	-0.21	0.00	0.052
Free cholesterol in medium LDL (mmol/l)	896	-0.02	-0.12	0.07	0.600	896	-0.08	-0.18	0.02	0.114	874	-0.08	-0.18	0.03	0.144
Triglycerides in medium LDL (mmol/l)	896	-0.03	-0.13	0.06	0.463	896	-0.08	-0.18	0.01	0.093	874	-0.11	-0.22	-0.01	0.028
Concentration of small LDL particles (mol/l)	896	-0.06	-0.16	0.03	0.175	896	-0.12	-0.22	-0.02	0.018	874	-0.12	-0.23	-0.02	0.019
Total lipids in small LDL (mmol/l)	896	-0.05	-0.14	0.04	0.256	896	-0.11	-0.20	-0.01	0.035	874	-0.11	-0.21	0.00	0.041
Phospholipids in small LDL (mmol/l)	896	-0.04	-0.13	0.05	0.335	896	-0.10	-0.19	0.00	0.046	874	-0.10	-0.20	0.00	0.060
Total cholesterol in small LDL (mmol/l)	896	-0.05	-0.14	0.04	0.300	896	-0.10	-0.20	0.00	0.050	874	-0.10	-0.20	0.00	0.059
Cholesterol esters in small LDL (mmol/l)	896	-0.06	-0.15	0.04	0.237	896	-0.11	-0.21	-0.01	0.039	874	-0.11	-0.21	0.00	0.043
Free cholesterol in small LDL (mmol/l)	896	-0.01	-0.10	0.08	0.790	896	-0.06	-0.16	0.03	0.204	874	-0.05	-0.15	0.05	0.314
Triglycerides in small LDL (mmol/l)	896	-0.09	-0.18	0.00	0.053	896	-0.14	-0.24	-0.04	0.007	874	-0.15	-0.26	-0.05	0.004
Concentration of very large HDL particles (mol/l)	896	0.15	0.06	0.25	1.64E-03	896	0.14	0.04	0.24	0.006	874	0.08	-0.02	0.19	0.103
Total lipids in very large HDL (mmol/l)	896	0.15	0.05	0.24	2.56E-03	896	0.14	0.03	0.24	0.009	874	0.08	-0.02	0.18	0.125
Phospholipids in very large HDL (mmol/l)	896	0.16	0.07	0.26	7.71E-04	896	0.15	0.05	0.25	0.003	874	0.09	-0.01	0.19	0.069
Total cholesterol in very large HDL (mmol/l)	896	0.12	0.03	0.22	0.012	896	0.11	0.01	0.21	0.033	874	0.06	-0.04	0.16	0.244
Cholesterol esters in very large HDL (mmol/l)	896	0.11	0.02	0.21	0.019	896	0.10	0.00	0.20	0.049	874	0.05	-0.05	0.16	0.293
Free cholesterol in very large HDL (mmol/l)	896	0.14	0.04	0.23	0.005	896	0.13	0.03	0.23	0.014	874	0.07	-0.03	0.18	0.161
Triglycerides in very large HDL (mmol/l)	896	0.05	-0.05	0.15	0.301	896	0.06	-0.04	0.16	0.226	874	0.01	-0.09	0.11	0.810
Concentration of large HDL particles (mol/l)	896	0.20	0.10	0.29	2.92E-05	896	0.18	0.08	0.28	3.47E-04	874	0.13	0.03	0.22	0.012
Total lipids in large HDL (mmol/l)	896	0.19	0.10	0.28	3.70E-05	896	0.18	0.08	0.28	3.76E-04	874	0.13	0.03	0.22	0.012
Phospholipids in large HDL (mmol/l)	896	0.18	0.09	0.27	1.10E-04	896	0.16	0.06	0.26	1.33E-03	874	0.11	0.01	0.21	0.029
Total cholesterol in large HDL (mmol/l)	896	0.20	0.11	0.29	1.79E-05	896	0.19	0.09	0.29	1.54E-04	874	0.14	0.04	0.24	0.006
Cholesterol esters in large HDL (mmol/l)	896	0.20	0.11	0.29	1.59E-05	896	0.19	0.09	0.29	1.34E-04	874	0.14	0.04	0.24	0.006
Free cholesterol in large HDL (mmol/l)	896	0.19	0.10	0.29	3.09E-05	896	0.18	0.09	0.28	2.73E-04	874	0.13	0.03	0.23	0.009
Triglycerides in large HDL (mmol/l)	896	0.12	0.03	0.21	0.010	896	0.12	0.02	0.22	0.014	874	0.08	-0.02	0.18	0.102
Concentration of medium HDL particles (mol/l)	896	0.12	0.03	0.21	0.006	896	0.07	-0.02	0.17	0.109	874	0.07	-0.03	0.16	0.160
Total lipids in medium HDL (mmol/l)	896	0.12	0.04	0.21	0.005	896	0.08	-0.01	0.17	0.086	874	0.07	-0.03	0.17	0.149
Phospholipids in medium HDL (mmol/l)	896	0.13	0.04	0.22	2.81E-03	896	0.08	-0.01	0.17	0.075	874	0.07	-0.02	0.16	0.145
Total cholesterol in medium HDL (mmol/l)	896	0.13	0.04	0.22	0.004	896	0.10	0.00	0.19	0.046	874	0.09	-0.01	0.18	0.090
Cholesterol esters in medium HDL (mmol/l)	896	0.13	0.04	0.22	0.004	896	0.10	0.00	0.20	0.043	874	0.09	-0.01	0.19	0.086
Free cholesterol in medium HDL (mmol/l)	896	0.12	0.03	0.21	0.006	896	0.08	-0.01	0.18	0.078	874	0.07	-0.02	0.17	0.130
Triglycerides in medium HDL (mmol/l)	896	-0.08	-0.17	0.01	0.080	896	-0.11	-0.21	-0.01	0.027	874	-0.09	-0.19	0.01	0.086
Concentration of small HDL particles (mol/l)	896	-0.04	-0.13	0.04	0.332	896	-0.09	-0.19	0.00	0.050	874	-0.07	-0.17	0.03	0.162
Total lipids in small HDL (mmol/l)	896	0.02	-0.06	0.11	0.602	896	-0.04	-0.13	0.05	0.358	874	-0.03	-0.12	0.07	0.576
Phospholipids in small HDL (mmol/l)	896	-0.05	-0.14	0.04	0.292	896	-0.08	-0.18	0.02	0.115	874	-0.06	-0.16	0.04	0.238

S6 Table Associations of longer-term moderate-to-vigorous physical activity (mean of MVPA measures at age 12y, 14y, and 15y) with metabolic traits at age 15y in ALSPAC

Mean of MVPA at age 12y, 14y, 15y (per SD (12 min/day) higher)

	Adj. for age, sex, ethnicity, maternal education, smoking, alcohol, mean wear time, wear month					*Additionally adj. for mean SED*					*Additionally adj. for mean FMI*				
**Standardised outcome at age 15y**	**N**	**Beta**	**LCL**	**UCL**	**P-value**	**N**	**Beta**	**LCL**	**UCL**	**P-value**	**N**	**Beta**	**LCL**	**UCL**	**P-value**
Total cholesterol in small HDL (mmol/l)	896	0.11	0.03	0.19	0.007	896	0.03	-0.05	0.12	0.451	874	0.04	-0.05	0.13	0.368
Cholesterol esters in small HDL (mmol/l)	896	0.11	0.03	0.19	0.009	896	0.03	-0.06	0.12	0.487	874	0.04	-0.05	0.13	0.376
Free cholesterol in small HDL (mmol/l)	896	0.07	-0.02	0.16	0.111	896	0.03	-0.07	0.12	0.551	874	0.03	-0.07	0.12	0.602
Triglycerides in small HDL (mmol/l)	896	-0.13	-0.22	-0.04	0.004	896	-0.16	-0.25	-0.06	1.39E-03	874	-0.15	-0.25	-0.05	0.004
Phospholipids to total lipids ratio in chylomicrons and extremely large VLDL (%)	896	-0.09	-0.18	0.00	0.062	896	-0.09	-0.19	0.00	0.061	874	-0.11	-0.21	-0.01	0.037
Total cholesterol to total lipids ratio in chylomicrons and extremely large VLDL (%)	896	0.00	-0.10	0.09	0.982	896	-0.04	-0.14	0.06	0.434	874	-0.02	-0.13	0.08	0.645
Cholesterol esters to total lipids ratio in chylomicrons and extremely large VLDL (%)	896	0.04	-0.06	0.13	0.446	896	-0.01	-0.11	0.09	0.831	874	0.01	-0.09	0.11	0.838
Free cholesterol to total lipids ratio in chylomicrons and extremely large VLDL (%)	896	-0.11	-0.21	-0.01	0.034	896	-0.11	-0.21	0.00	0.046	874	-0.11	-0.23	0.00	0.043
Triglycerides to total lipids ratio in chylomicrons and extremely large VLDL (%)	896	0.01	-0.05	0.08	0.665	896	0.04	-0.03	0.11	0.232	874	0.03	-0.04	0.11	0.387
Phospholipids to total lipds ratio in very large VLDL (%)	896	-0.13	-0.23	-0.03	0.012	896	-0.16	-0.26	-0.05	0.005	874	-0.16	-0.27	-0.05	0.006
Total cholesterol to total lipids ratio in very large VLDL (%)	896	0.13	0.02	0.23	0.021	896	0.10	-0.02	0.21	0.115	874	0.11	-0.01	0.24	0.080
Cholesterol esters to total lipids ratio in very large VLDL (%)	896	0.15	0.03	0.26	0.012	896	0.12	0.00	0.25	0.057	874	0.13	0.00	0.27	0.045
Free cholesterol to total lipids ratio in very large VLDL (%)	896	0.09	-0.03	0.21	0.128	896	0.06	-0.07	0.19	0.399	874	0.05	-0.08	0.19	0.427
Triglycerides to total lipids ratio in very large VLDL (%)	896	-0.09	-0.21	0.02	0.115	896	-0.05	-0.18	0.08	0.421	874	-0.06	-0.19	0.07	0.377
Phospholipids to total lipds ratio in large VLDL (%)	896	-0.13	-0.24	-0.02	0.024	896	-0.13	-0.26	-0.01	0.037	874	-0.13	-0.26	0.00	0.046
Total cholesterol to total lipids ratio in large VLDL (%)	896	-0.05	-0.15	0.05	0.362	896	-0.07	-0.18	0.04	0.216	874	-0.06	-0.17	0.06	0.342
Cholesterol esters to total lipids ratio in large VLDL (%)	896	0.04	-0.04	0.11	0.348	896	0.01	-0.06	0.09	0.720	874	0.05	-0.06	0.15	0.378
Free cholesterol to total lipids ratio in large VLDL (%)	896	-0.14	-0.24	-0.04	0.008	896	-0.15	-0.26	-0.03	0.013	874	-0.14	-0.26	-0.02	0.019
Triglycerides to total lipids ratio in large VLDL (%)	896	0.03	-0.04	0.10	0.373	896	0.02	-0.04	0.09	0.475	874	0.05	-0.05	0.15	0.300
Phospholipids to total lipds ratio in medium VLDL (%)	896	0.12	0.01	0.23	0.029	896	0.09	-0.03	0.21	0.141	874	0.05	-0.07	0.17	0.447
Total cholesterol to total lipids ratio in medium VLDL (%)	896	0.06	-0.03	0.16	0.177	896	0.03	-0.07	0.13	0.511	874	0.04	-0.07	0.14	0.481
Cholesterol esters to total lipids ratio in medium VLDL (%)	896	0.09	0.00	0.18	0.055	896	0.06	-0.04	0.17	0.218	874	0.08	-0.03	0.18	0.163
Free cholesterol to total lipids ratio in medium VLDL (%)	896	-0.06	-0.15	0.04	0.219	896	-0.08	-0.19	0.02	0.104	874	-0.11	-0.21	0.00	0.047
Triglycerides to total lipids ratio in medium VLDL (%)	896	-0.08	-0.18	0.01	0.076	896	-0.05	-0.15	0.05	0.332	874	-0.04	-0.15	0.06	0.399
Phospholipids to total lipds ratio in small VLDL (%)	896	0.18	0.09	0.28	1.76E-04	896	0.17	0.06	0.28	1.97E-03	874	0.17	0.06	0.28	2.38E-03
Total cholesterol to total lipids ratio in small VLDL (%)	896	0.05	-0.05	0.15	0.324	896	0.03	-0.08	0.13	0.647	874	0.03	-0.08	0.14	0.549
Cholesterol esters to total lipids ratio in small VLDL (%)	896	0.02	-0.08	0.12	0.714	896	0.00	-0.11	0.11	0.987	874	0.01	-0.10	0.12	0.839
Free cholesterol to total lipids ratio in small VLDL (%)	896	0.21	0.12	0.30	7.40E-06	896	0.17	0.07	0.27	7.56E-04	874	0.15	0.05	0.25	2.75E-03
Triglycerides to total lipids ratio in small VLDL (%)	896	-0.10	-0.20	0.00	0.041	896	-0.08	-0.18	0.03	0.171	874	-0.08	-0.20	0.03	0.138
Phospholipids to total lipds ratio in very small VLDL (%)	896	0.03	-0.06	0.12	0.512	896	-0.01	-0.11	0.09	0.894	874	-0.04	-0.14	0.06	0.457
Total cholesterol to total lipids ratio in very small VLDL (%)	896	0.06	-0.03	0.16	0.203	896	0.09	-0.02	0.20	0.095	874	0.12	0.01	0.23	0.032
Cholesterol esters to total lipids ratio in very small VLDL (%)	896	-0.03	-0.12	0.06	0.547	896	0.00	-0.10	0.10	0.989	874	0.03	-0.06	0.13	0.501
Free cholesterol to total lipids ratio in very small VLDL (%)	896	0.22	0.12	0.32	1.85E-05	896	0.23	0.12	0.34	8.59E-05	874	0.24	0.11	0.36	1.53E-04
Triglycerides to total lipids ratio in very small VLDL (%)	896	-0.10	-0.19	0.00	0.050	896	-0.10	-0.21	0.00	0.059	874	-0.11	-0.22	-0.01	0.040
Phospholipids to total lipds ratio in IDL (%)	896	0.03	-0.07	0.13	0.555	896	0.02	-0.09	0.13	0.678	874	-0.02	-0.13	0.10	0.783
Total cholesterol to total lipids ratio in IDL (%)	896	-0.01	-0.10	0.09	0.874	896	0.00	-0.11	0.10	0.961	874	0.03	-0.08	0.14	0.583
Cholesterol esters to total lipids ratio in IDL (%)	896	-0.05	-0.15	0.04	0.290	896	-0.04	-0.15	0.06	0.410	874	0.00	-0.11	0.11	0.994
Free cholesterol to total lipids ratio in IDL (%)	896	0.10	0.01	0.19	0.032	896	0.09	0.00	0.19	0.058	874	0.07	-0.03	0.16	0.179
Triglycerides to total lipids ratio in IDL (%)	896	-0.01	-0.09	0.08	0.896	896	-0.01	-0.11	0.09	0.870	874	-0.03	-0.13	0.07	0.568
Phospholipids to total lipds ratio in large LDL (%)	896	0.00	-0.08	0.09	0.911	896	0.02	-0.07	0.11	0.662	874	0.04	-0.06	0.13	0.434
Total cholesterol to total lipids ratio in large LDL (%)	896	-0.01	-0.10	0.07	0.743	896	-0.03	-0.13	0.07	0.569	874	-0.02	-0.12	0.08	0.696
Cholesterol esters to total lipids ratio in large LDL (%)	896	-0.04	-0.13	0.04	0.320	896	-0.06	-0.16	0.03	0.198	874	-0.06	-0.16	0.04	0.267
Free cholesterol to total lipids ratio in large LDL (%)	896	0.09	0.01	0.18	0.029	896	0.12	0.03	0.21	0.010	874	0.12	0.03	0.21	0.012
Triglycerides to total lipids ratio in large LDL (%)	896	0.02	-0.07	0.11	0.669	896	0.02	-0.08	0.12	0.631	874	-0.01	-0.11	0.09	0.855
Phospholipids to total lipds ratio in medium LDL (%)	896	0.00	-0.03	0.04	0.770	896	0.01	-0.03	0.05	0.537	874	0.02	-0.02	0.06	0.344
Total cholesterol to total lipids ratio in medium LDL (%)	896	-0.02	-0.11	0.07	0.628	896	-0.03	-0.14	0.07	0.545	874	-0.03	-0.14	0.07	0.549
Cholesterol esters to total lipids ratio in medium LDL (%)	896	-0.04	-0.13	0.06	0.423	896	-0.06	-0.16	0.05	0.299	874	-0.06	-0.17	0.05	0.268
Free cholesterol to total lipids ratio in medium LDL (%)	896	0.01	-0.01	0.04	0.258	896	0.02	-0.01	0.05	0.126	874	0.02	0.00	0.05	0.091

S6 Table Associations of longer-term moderate-to-vigorous physical activity (mean of MVPA measures at age 12y, 14y, and 15y) with metabolic traits at age 15y in ALSPAC

Mean of MVPA at age 12y, 14y, 15y (per SD (12 min/day) higher)

	Adj. for age, sex, ethnicity, maternal education, smoking, alcohol, mean wear time, wear month					*Additionally adj. for mean SED*					*Additionally adj. for mean FMI*				
**Standardised outcome at age 15y**	**N**	**Beta**	**LCL**	**UCL**	**P-value**	**N**	**Beta**	**LCL**	**UCL**	**P-value**	**N**	**Beta**	**LCL**	**UCL**	**P-value**
Triglycerides to total lipids ratio in medium LDL (%)	896	0.02	-0.07	0.11	0.608	896	0.01	-0.09	0.11	0.899	874	-0.03	-0.13	0.07	0.550
Phospholipids to total lipds ratio in small LDL (%)	896	0.02	-0.03	0.07	0.430	896	0.04	-0.02	0.09	0.239	874	0.04	-0.02	0.10	0.201
Total cholesterol to total lipids ratio in small LDL (%)	896	-0.01	-0.10	0.08	0.778	896	-0.03	-0.13	0.07	0.574	874	-0.03	-0.13	0.08	0.588
Cholesterol esters to total lipids ratio in small LDL (%)	896	-0.04	-0.13	0.06	0.459	896	-0.06	-0.16	0.05	0.278	874	-0.06	-0.17	0.04	0.242
Free cholesterol to total lipids ratio in small LDL (%)	896	0.03	-0.02	0.08	0.177	896	0.05	-0.01	0.10	0.080	874	0.06	0.00	0.11	0.046
Triglycerides to total lipids ratio in small LDL (%)	896	-0.06	-0.15	0.03	0.180	896	-0.08	-0.18	0.02	0.110	874	-0.10	-0.20	0.00	0.053
Phospholipids to total lipds ratio in very large HDL (%)	896	0.15	0.06	0.24	7.31E-04	896	0.14	0.05	0.24	2.79E-03	874	0.10	0.00	0.19	0.043
Total cholesterol to total lipids ratio in very large HDL (%)	896	-0.14	-0.23	-0.05	1.40E-03	896	-0.13	-0.23	-0.04	0.004	874	-0.09	-0.18	0.01	0.064
Cholesterol esters to total lipids ratio in very large HDL (%)	896	-0.14	-0.22	-0.05	2.06E-03	896	-0.13	-0.23	-0.04	0.006	874	-0.08	-0.18	0.01	0.076
Free cholesterol to total lipids ratio in very large HDL (%)	896	0.01	-0.08	0.10	0.825	896	0.03	-0.07	0.12	0.596	874	0.00	-0.10	0.10	0.988
Triglycerides to total lipids ratio in very large HDL (%)	896	-0.09	-0.19	0.01	0.066	896	-0.08	-0.18	0.02	0.129	874	-0.07	-0.17	0.03	0.173
Phospholipids to total lipds ratio in large HDL (%)	896	-0.16	-0.24	-0.08	7.18E-05	896	-0.20	-0.29	-0.11	8.77E-06	874	-0.16	-0.25	-0.07	7.16E-04
Total cholesterol to total lipids ratio in large HDL (%)	896	0.17	0.08	0.25	9.87E-05	896	0.19	0.10	0.28	3.75E-05	874	0.15	0.05	0.24	2.04E-03
Cholesterol esters to total lipids ratio in large HDL (%)	896	0.17	0.09	0.26	9.06E-05	896	0.20	0.10	0.29	3.34E-05	874	0.15	0.05	0.24	2.19E-03
Free cholesterol to total lipids ratio in large HDL (%)	896	0.12	0.04	0.21	0.005	896	0.14	0.05	0.23	0.003	874	0.11	0.02	0.20	0.020
Triglycerides to total lipids ratio in large HDL (%)	896	-0.13	-0.23	-0.04	0.006	896	-0.12	-0.21	-0.02	0.018	874	-0.08	-0.18	0.02	0.105
Phospholipids to total lipds ratio in medium HDL (%)	896	0.11	0.02	0.20	0.020	896	0.07	-0.03	0.17	0.183	874	0.05	-0.05	0.16	0.313
Total cholesterol to total lipids ratio in medium HDL (%)	896	0.00	-0.09	0.09	0.979	896	0.04	-0.06	0.14	0.441	874	0.03	-0.07	0.13	0.526
Cholesterol esters to total lipids ratio in medium HDL (%)	896	0.00	-0.09	0.10	0.963	896	0.04	-0.06	0.14	0.432	874	0.03	-0.07	0.13	0.530
Free cholesterol to total lipids ratio in medium HDL (%)	896	-0.02	-0.12	0.09	0.741	896	0.01	-0.09	0.11	0.824	874	0.02	-0.09	0.12	0.762
Triglycerides to total lipids ratio in medium HDL (%)	896	-0.14	-0.24	-0.05	2.95E-03	896	-0.16	-0.26	-0.05	2.65E-03	874	-0.13	-0.23	-0.02	0.016
Phospholipids to total lipds ratio in small HDL (%)	896	-0.13	-0.21	-0.05	2.59E-03	896	-0.07	-0.16	0.02	0.153	874	-0.06	-0.16	0.03	0.170
Total cholesterol to total lipids ratio in small HDL (%)	896	0.16	0.07	0.24	2.38E-04	896	0.10	0.01	0.19	0.034	874	0.10	0.00	0.19	0.042
Cholesterol esters to total lipids ratio in small HDL (%)	896	0.13	0.05	0.22	2.21E-03	896	0.07	-0.02	0.16	0.136	874	0.07	-0.02	0.17	0.122
Free cholesterol to total lipids ratio in small HDL (%)	896	0.13	0.03	0.22	0.008	896	0.17	0.07	0.28	7.38E-04	874	0.13	0.03	0.23	0.012
Triglycerides to total lipids ratio in small HDL (%)	896	-0.16	-0.26	-0.07	5.64E-04	896	-0.16	-0.26	-0.06	1.56E-03	874	-0.16	-0.26	-0.06	2.37E-03
Mean diameter for VLDL particles (nm)	896	-0.18	-0.27	-0.08	2.48E-04	896	-0.17	-0.27	-0.07	1.41E-03	874	-0.15	-0.25	-0.04	0.005
Mean diameter for LDL particles (nm)	896	0.09	0.01	0.17	0.032	896	0.13	0.04	0.22	0.003	874	0.12	0.03	0.22	0.009
Mean diameter for HDL particles (nm)	896	0.18	0.09	0.28	1.42E-04	896	0.18	0.08	0.29	4.51E-04	874	0.13	0.02	0.23	0.016
Serum total cholesterol (mmol/l)	896	0.01	-0.08	0.10	0.906	896	-0.04	-0.14	0.05	0.376	874	-0.05	-0.15	0.05	0.336
Total cholesterol in VLDL (mmol/l)	896	-0.13	-0.23	-0.04	0.007	896	-0.15	-0.25	-0.05	0.004	874	-0.11	-0.22	-0.01	0.030
Remnant cholesterol (non-HDL, non-LDL -cholesterol) (mmol/l)	896	-0.09	-0.19	0.00	0.061	896	-0.12	-0.22	-0.02	0.019	874	-0.10	-0.20	0.01	0.065
Total cholesterol in LDL (mmol/l)	896	-0.04	-0.13	0.05	0.400	896	-0.09	-0.19	0.01	0.078	874	-0.09	-0.19	0.01	0.087
Total cholesterol in HDL (mmol/l)	896	0.18	0.09	0.27	1.00E-04	896	0.15	0.05	0.24	2.64E-03	874	0.11	0.01	0.20	0.031
Total cholesterol in HDL2 (mmol/l)	896	0.18	0.09	0.27	9.35E-05	896	0.15	0.06	0.25	2.19E-03	874	0.11	0.01	0.21	0.025
Total cholesterol in HDL3 (mmol/l)	896	0.16	0.07	0.25	2.95E-04	896	0.13	0.04	0.22	0.007	874	0.09	0.00	0.18	0.059
Esterified cholesterol (mmol/l)	896	0.01	-0.08	0.10	0.854	896	-0.04	-0.13	0.06	0.421	874	-0.05	-0.15	0.05	0.354
Free cholesterol (mmol/l)	896	0.00	-0.09	0.09	0.972	896	-0.05	-0.15	0.05	0.309	874	-0.05	-0.15	0.05	0.322
Serum total triglycerides (mmol/l)	896	-0.16	-0.26	-0.07	9.02E-04	896	-0.17	-0.28	-0.07	8.64E-04	874	-0.16	-0.26	-0.06	2.67E-03
Triglycerides in VLDL (mmol/l)	896	-0.18	-0.28	-0.08	3.63E-04	896	-0.18	-0.28	-0.07	7.13E-04	874	-0.15	-0.25	-0.05	0.004
Triglycerides in LDL (mmol/l)	896	-0.04	-0.13	0.05	0.385	896	-0.09	-0.18	0.01	0.080	874	-0.11	-0.21	-0.01	0.025
Triglycerides in HDL (mmol/l)	896	-0.05	-0.14	0.04	0.273	896	-0.07	-0.17	0.03	0.156	874	-0.08	-0.17	0.02	0.129
Diacylglycerol (mmol/l)	864	-0.05	-0.14	0.05	0.321	864	-0.04	-0.14	0.06	0.470	843	-0.02	-0.12	0.08	0.740
Ratio of diacylglycerol to triglycerides	864	0.01	-0.08	0.11	0.786	864	0.03	-0.08	0.13	0.642	843	0.03	-0.08	0.14	0.582
Total phosphoglycerides (mmol/l)	896	0.08	0.00	0.17	0.059	896	0.04	-0.05	0.13	0.413	874	0.01	-0.08	0.10	0.833
Ratio of triglycerides to phosphoglycerides	896	-0.18	-0.27	-0.08	3.07E-04	896	-0.16	-0.26	-0.06	1.84E-03	874	-0.13	-0.23	-0.03	0.011
Phosphatidylcholine and other cholines (mmol/l)	877	0.10	0.01	0.18	0.029	877	0.06	-0.03	0.15	0.173	855	0.03	-0.07	0.12	0.543
Total cholines (mmol/l)	893	0.08	0.00	0.17	0.064	893	0.03	-0.06	0.12	0.479	871	0.00	-0.09	0.10	0.975

S6 Table Associations of longer-term moderate-to-vigorous physical activity (mean of MVPA measures at age 12y, 14y, and 15y) with metabolic traits at age 15y in ALSPAC

Mean of MVPA at age 12y, 14y, 15y (per SD (12 min/day) higher)

	Adj. for age, sex, ethnicity, maternal education, smoking, alcohol, mean wear time, wear month					*Additionally adj. for mean SED*					*Additionally adj. for mean FMI*				
**Standardised outcome at age 15y**	**N**	**Beta**	**LCL**	**UCL**	**P-value**	**N**	**Beta**	**LCL**	**UCL**	**P-value**	**N**	**Beta**	**LCL**	**UCL**	**P-value**
Apolipoprotein A-I (g/l)	896	0.13	0.04	0.21	0.005	896	0.09	-0.01	0.18	0.069	874	0.05	-0.04	0.15	0.269
Apolipoprotein B (g/l)	896	-0.13	-0.22	-0.03	0.009	896	-0.16	-0.26	-0.06	2.42E-03	874	-0.14	-0.25	-0.04	0.008
Ratio of apolipoprotein B to apolipoprotein A-I	896	-0.19	-0.28	-0.09	1.55E-04	896	-0.20	-0.31	-0.10	1.98E-04	874	-0.17	-0.27	-0.06	2.41E-03
Total fatty acids (mmol/l)	896	-0.03	-0.12	0.07	0.568	896	-0.06	-0.16	0.03	0.204	874	-0.07	-0.17	0.03	0.201
Estimated description of fatty acid chain length, not actual carbon number	892	-0.03	-0.13	0.07	0.591	892	0.01	-0.10	0.12	0.836	870	0.01	-0.10	0.12	0.872
Estimated degree of unsaturation	895	0.01	-0.09	0.11	0.800	895	0.04	-0.07	0.15	0.503	873	0.04	-0.07	0.16	0.447
22:6, docosahexaenoic acid (mmol/l)	896	0.03	-0.07	0.13	0.609	896	0.01	-0.09	0.11	0.852	874	0.02	-0.09	0.12	0.745
18:2, linoleic acid (mmol/l)	893	-0.02	-0.10	0.07	0.710	893	-0.06	-0.15	0.04	0.220	871	-0.08	-0.17	0.02	0.132
Conjugated linoleic acid (mmol/l)	896	-0.07	-0.18	0.03	0.154	896	-0.05	-0.16	0.06	0.408	874	-0.08	-0.19	0.02	0.114
Omega-3 fatty acids (mmol/l)	894	-0.02	-0.12	0.08	0.686	894	-0.04	-0.14	0.07	0.474	872	-0.04	-0.15	0.07	0.436
Omega-6 fatty acids (mmol/l)	895	-0.01	-0.10	0.08	0.799	895	-0.05	-0.15	0.05	0.301	873	-0.06	-0.16	0.04	0.242
Polyunsaturated fatty acids (mmol/l)	893	-0.01	-0.09	0.08	0.878	893	-0.05	-0.14	0.05	0.360	871	-0.05	-0.15	0.05	0.285
Monounsaturated fatty acids; 16:1, 18:1 (mmol/l)	893	-0.09	-0.18	0.01	0.070	893	-0.12	-0.22	-0.02	0.022	871	-0.10	-0.20	0.00	0.046
Saturated fatty acids (mmol/l)	892	0.02	-0.08	0.13	0.699	892	-0.02	-0.13	0.10	0.790	870	-0.02	-0.14	0.09	0.686
Ratio of 22:6 docosahexaenoic acid to total fatty acids (%)	896	0.05	-0.06	0.15	0.366	896	0.05	-0.06	0.17	0.359	874	0.06	-0.05	0.18	0.277
Ratio of 18:2 linoleic acid to total fatty acids (%)	893	0.02	-0.09	0.12	0.731	893	0.01	-0.10	0.13	0.805	871	-0.01	-0.12	0.11	0.903
Ratio of conjugated linoleic acid to total fatty acids (%)	896	-0.08	-0.18	0.02	0.107	896	-0.05	-0.16	0.06	0.374	874	-0.08	-0.19	0.02	0.108
Ratio of omega-3 fatty acids to total fatty acids (%)	894	0.01	-0.09	0.10	0.915	894	0.01	-0.10	0.12	0.799	872	0.01	-0.11	0.12	0.902
Ratio of omega-6 fatty acids to total fatty acids (%)	895	0.04	-0.06	0.14	0.458	895	0.05	-0.07	0.16	0.406	873	0.04	-0.08	0.15	0.535
Ratio of polyunsaturated fatty acids to total fatty acids (%)	893	0.04	-0.06	0.14	0.425	893	0.05	-0.06	0.16	0.386	871	0.04	-0.08	0.15	0.539
Ratio of monounsaturated fatty acids to total fatty acids (%)	893	-0.11	-0.21	0.00	0.045	893	-0.11	-0.22	0.01	0.064	871	-0.08	-0.20	0.03	0.159
Ratio of saturated fatty acids to total fatty acids (%)	892	0.09	-0.02	0.20	0.119	892	0.08	-0.05	0.20	0.226	870	0.06	-0.06	0.18	0.345
Insulin (mu/l)	927	-0.14	-0.20	-0.09	6.43E-07	927	-0.14	-0.20	-0.08	4.56E-06	904	-0.10	-0.16	-0.04	1.08E-03
Glucose (mmol/l)	894	-0.06	-0.14	0.02	0.125	894	-0.06	-0.15	0.03	0.200	872	-0.05	-0.14	0.04	0.289
Lactate (mmol/l)	894	-0.06	-0.15	0.04	0.221	894	-0.05	-0.15	0.05	0.356	872	-0.05	-0.16	0.06	0.350
Pyruvate (mmol/l)	893	-0.12	-0.21	-0.03	0.009	893	-0.08	-0.17	0.02	0.124	871	-0.05	-0.15	0.04	0.282
Citrate (mmol/l)	891	0.11	0.01	0.21	0.028	891	0.06	-0.04	0.17	0.212	869	0.04	-0.07	0.14	0.468
Alanine (mmol/l)	896	-0.09	-0.18	0.00	0.063	896	-0.03	-0.13	0.07	0.553	874	-0.04	-0.13	0.06	0.486
Glutamine (mmol/l)	896	0.02	-0.06	0.11	0.629	896	0.02	-0.07	0.12	0.636	874	0.00	-0.09	0.10	0.957
Histidine (mmol/l)	850	0.10	0.01	0.20	0.026	850	0.14	0.04	0.24	0.007	828	0.12	0.01	0.22	0.030
Isoleucine (mmol/l)	896	-0.05	-0.14	0.03	0.218	896	-0.03	-0.12	0.07	0.553	874	-0.01	-0.11	0.08	0.782
Leucine (mmol/l)	896	0.05	-0.03	0.13	0.198	896	0.06	-0.02	0.15	0.152	874	0.07	-0.02	0.16	0.122
Valine (mmol/l)	896	-0.01	-0.10	0.08	0.790	896	-0.01	-0.12	0.09	0.801	874	0.00	-0.10	0.11	0.990
Phenylalanine (mmol/l)	895	0.10	0.01	0.19	0.028	895	0.12	0.02	0.21	0.018	873	0.13	0.03	0.23	0.013
Tyrosine (mmol/l)	892	0.03	-0.06	0.13	0.503	892	0.03	-0.07	0.14	0.563	870	0.06	-0.05	0.16	0.312
Acetate (mmol/l)	895	0.10	0.00	0.19	0.040	895	0.09	-0.01	0.20	0.078	873	0.09	-0.01	0.20	0.086
Acetoacetate (mmol/l)	896	-0.05	-0.13	0.03	0.210	896	-0.04	-0.13	0.04	0.327	874	-0.04	-0.13	0.05	0.386
3-hydroxybutyrate (mmol/l)	895	-0.09	-0.17	0.00	0.039	895	-0.12	-0.22	-0.03	0.013	873	-0.11	-0.20	-0.01	0.032
Creatinine (mmol/l)	895	-0.05	-0.14	0.04	0.268	895	0.00	-0.09	0.10	0.952	873	0.00	-0.10	0.09	0.954
Albumin (signal area)	896	-0.13	-0.23	-0.03	0.008	896	-0.10	-0.21	0.00	0.061	874	-0.11	-0.22	0.00	0.046
Glycoprotein acetyls, mainly a1-acid glycoprotein (mmol/l)	895	-0.21	-0.30	-0.13	9.99E-07	895	-0.21	-0.30	-0.12	5.42E-06	873	-0.15	-0.24	-0.06	1.13E-03
C-reactive protein (mg/l)	929	0.02	-0.09	0.12	0.745	929	0.05	-0.07	0.17	0.421	906	0.07	-0.05	0.18	0.251

Mean of MVPA at age 12y, 14y, 15y (per SD (12 min/day) higher)

Complete case sample

Adj. for age, sex, ethnicity, maternal education,   *Additionally adj. for mean SED*   *Additionally adj. for mean FMI*

S6 Table Associations of longer-term moderate-to-vigorous physical activity (mean of MVPA measures at age 12y, 14y, and 15y) with metabolic traits at age 15y in ALSPAC

Mean of MVPA at age 12y, 14y, 15y (per SD (12 min/day) higher)

Adj. for age, sex, ethnicity, maternal education, smoking, alcohol, mean wear time, wear month | *Additionally adj. for mean SED* | *Additionally adj. for mean FMI*

Standardised outcome at age 15y	N	Beta	LCL	UCL	P-value	N	Beta	LCL	UCL	P-value	N	Beta	LCL	UCL	P-value

smoking, alcohol, mean wear time, wear month

	Adj. for age, sex, ethnicity, maternal education, smoking, alcohol, mean wear time, wear month					*Additionally adj. for mean SED*					*Additionally adj. for mean FMI*				
**Standardised outcome at age 15y**	**N**	**Beta**	**LCL**	**UCL**	**P-value**	**N**	**Beta**	**LCL**	**UCL**	**P-value**	**N**	**Beta**	**LCL**	**UCL**	**P-value**
Systolic blood pressure (mmHg)	755	-0.10	-0.20	-0.01	0.039	755	-0.10	-0.21	0.00	0.058	755	-0.07	-0.18	0.03	0.165
Diastolic blood pressure (mmHg)	755	0.01	-0.09	0.11	0.860	755	0.02	-0.09	0.13	0.675	755	0.03	-0.08	0.14	0.574
Concentration of chylomicrons and extremely large VLDL particles (mol/l)	755	-0.17	-0.26	-0.07	6.39E-04	755	-0.17	-0.27	-0.07	5.96E-04	755	-0.14	-0.23	-0.04	0.007
Total lipids in chylomicrons and extremely large VLDL (mmol/l)	755	-0.16	-0.26	-0.06	1.18E-03	755	-0.17	-0.26	-0.07	1.15E-03	755	-0.13	-0.23	-0.03	0.010
Phospholipids in chylomicrons and extremely large VLDL (mmol/l)	755	-0.16	-0.26	-0.07	9.35E-04	755	-0.17	-0.27	-0.07	8.52E-04	755	-0.14	-0.24	-0.04	0.008
Total cholesterol in chylomicrons and extremely large VLDL (mmol/l)	755	-0.13	-0.23	-0.04	0.005	755	-0.14	-0.23	-0.04	0.005	755	-0.10	-0.20	-0.01	0.037
Cholesterol esters in chylomicrons and extremely large VLDL (mmol/l)	755	-0.11	-0.20	-0.01	0.030	755	-0.11	-0.20	-0.01	0.028	755	-0.07	-0.16	0.03	0.159
Free cholesterol in chylomicrons and extremely large VLDL (mmol/l)	755	-0.16	-0.26	-0.06	1.13E-03	755	-0.17	-0.27	-0.07	1.10E-03	755	-0.13	-0.23	-0.03	0.009
Triglycerides in chylomicrons and extremely large VLDL (mmol/l)	755	-0.16	-0.26	-0.07	9.15E-04	755	-0.17	-0.27	-0.07	9.43E-04	755	-0.13	-0.23	-0.03	0.009
Concentration of very large VLDL particles (mol/l)	755	-0.15	-0.24	-0.05	2.53E-03	755	-0.15	-0.25	-0.05	0.003	755	-0.11	-0.21	-0.01	0.024
Total lipids in very large VLDL (mmol/l)	755	-0.14	-0.24	-0.05	0.004	755	-0.14	-0.24	-0.05	0.004	755	-0.11	-0.21	-0.01	0.030
Phospholipids in very large VLDL (mmol/l)	755	-0.15	-0.24	-0.05	2.65E-03	755	-0.15	-0.25	-0.05	2.74E-03	755	-0.12	-0.22	-0.02	0.020
Total cholesterol in very large VLDL (mmol/l)	755	-0.15	-0.24	-0.05	0.003	755	-0.15	-0.25	-0.05	0.003	755	-0.11	-0.21	-0.01	0.026
Cholesterol esters in very large VLDL (mmol/l)	755	-0.14	-0.24	-0.04	0.005	755	-0.14	-0.24	-0.04	0.006	755	-0.10	-0.19	0.00	0.049
Free cholesterol in very large VLDL (mmol/l)	755	-0.15	-0.25	-0.06	1.86E-03	755	-0.16	-0.26	-0.06	1.60E-03	755	-0.12	-0.22	-0.03	0.014
Triglycerides in very large VLDL (mmol/l)	755	-0.14	-0.24	-0.04	0.004	755	-0.14	-0.24	-0.04	0.006	755	-0.11	-0.21	-0.01	0.036
Concentration of large VLDL particles (mol/l)	755	-0.13	-0.23	-0.03	0.008	755	-0.13	-0.23	-0.03	0.011	755	-0.10	-0.20	0.00	0.060
Total lipids in large VLDL (mmol/l)	755	-0.13	-0.23	-0.03	0.010	755	-0.13	-0.23	-0.03	0.012	755	-0.09	-0.19	0.01	0.068
Phospholipids in large VLDL (mmol/l)	755	-0.13	-0.23	-0.03	0.009	755	-0.13	-0.23	-0.03	0.010	755	-0.10	-0.20	0.00	0.058
Total cholesterol in large VLDL (mmol/l)	755	-0.12	-0.22	-0.02	0.017	755	-0.12	-0.22	-0.02	0.017	755	-0.09	-0.18	0.01	0.094
Cholesterol esters in large VLDL (mmol/l)	755	-0.11	-0.21	-0.01	0.037	755	-0.11	-0.21	-0.01	0.034	755	-0.07	-0.17	0.03	0.169
Free cholesterol in large VLDL (mmol/l)	755	-0.13	-0.23	-0.03	0.009	755	-0.13	-0.23	-0.03	0.009	755	-0.10	-0.20	0.00	0.053
Triglycerides in large VLDL (mmol/l)	755	-0.13	-0.23	-0.03	0.008	755	-0.13	-0.23	-0.03	0.012	755	-0.09	-0.20	0.01	0.065
Concentration of medium VLDL particles (mol/l)	755	-0.13	-0.24	-0.03	0.009	755	-0.13	-0.24	-0.03	0.011	755	-0.10	-0.20	0.01	0.068
Total lipids in medium VLDL (mmol/l)	755	-0.13	-0.23	-0.03	0.015	755	-0.13	-0.23	-0.02	0.016	755	-0.09	-0.19	0.01	0.091
Phospholipids in medium VLDL (mmol/l)	755	-0.13	-0.23	-0.02	0.015	755	-0.13	-0.24	-0.03	0.014	755	-0.09	-0.20	0.01	0.074
Total cholesterol in medium VLDL (mmol/l)	755	-0.09	-0.20	0.01	0.072	755	-0.10	-0.21	0.00	0.057	755	-0.06	-0.17	0.04	0.230
Cholesterol esters in medium VLDL (mmol/l)	755	-0.06	-0.17	0.04	0.226	755	-0.07	-0.18	0.03	0.184	755	-0.03	-0.14	0.07	0.539
Free cholesterol in medium VLDL (mmol/l)	755	-0.12	-0.22	-0.02	0.016	755	-0.13	-0.24	-0.03	0.014	755	-0.10	-0.20	0.01	0.071
Triglycerides in medium VLDL (mmol/l)	755	-0.14	-0.24	-0.04	0.007	755	-0.14	-0.24	-0.03	0.011	755	-0.10	-0.20	0.01	0.066
Concentration of small VLDL particles (mol/l)	755	-0.13	-0.23	-0.03	0.013	755	-0.15	-0.25	-0.04	0.007	755	-0.11	-0.22	0.00	0.045
Total lipids in small VLDL (mmol/l)	755	-0.13	-0.23	-0.02	0.015	755	-0.15	-0.26	-0.04	0.006	755	-0.11	-0.22	0.00	0.043
Phospholipids in small VLDL (mmol/l)	755	-0.10	-0.21	0.00	0.040	755	-0.13	-0.24	-0.03	0.013	755	-0.09	-0.20	0.01	0.079
Total cholesterol in small VLDL (mmol/l)	755	-0.11	-0.21	-0.01	0.036	755	-0.14	-0.25	-0.03	0.009	755	-0.10	-0.21	0.00	0.061
Cholesterol esters in small VLDL (mmol/l)	755	-0.11	-0.21	-0.01	0.037	755	-0.14	-0.25	-0.03	0.010	755	-0.10	-0.21	0.01	0.066
Free cholesterol in small VLDL (mmol/l)	755	-0.10	-0.20	0.00	0.052	755	-0.13	-0.24	-0.02	0.016	755	-0.10	-0.20	0.01	0.077
Triglycerides in small VLDL (mmol/l)	755	-0.13	-0.24	-0.03	0.011	755	-0.14	-0.25	-0.04	0.009	755	-0.11	-0.22	0.00	0.047
Concentration of very small VLDL particles (mol/l)	755	-0.04	-0.14	0.05	0.388	755	-0.08	-0.18	0.02	0.130	755	-0.06	-0.17	0.04	0.244
Total lipids in very small VLDL (mmol/l)	755	-0.07	-0.17	0.03	0.190	755	-0.10	-0.21	0.01	0.065	755	-0.08	-0.18	0.03	0.168
Phospholipids in very small VLDL (mmol/l)	755	-0.04	-0.13	0.06	0.467	755	-0.08	-0.18	0.02	0.128	755	-0.07	-0.18	0.03	0.188
Total cholesterol in very small VLDL (mmol/l)	755	-0.05	-0.15	0.05	0.355	755	-0.07	-0.18	0.04	0.229	755	-0.04	-0.15	0.07	0.505
Cholesterol esters in very small VLDL (mmol/l)	755	-0.08	-0.19	0.02	0.121	755	-0.10	-0.21	0.01	0.076	755	-0.06	-0.18	0.05	0.253
Free cholesterol in very small VLDL (mmol/l)	755	0.03	-0.07	0.13	0.599	755	0.01	-0.10	0.12	0.896	755	0.02	-0.09	0.13	0.694
Triglycerides in very small VLDL (mmol/l)	755	-0.11	-0.21	-0.02	0.023	755	-0.15	-0.25	-0.04	0.007	755	-0.13	-0.23	-0.02	0.021
Concentration of IDL particles (mol/l)	755	-0.03	-0.13	0.06	0.488	755	-0.08	-0.19	0.02	0.113	755	-0.09	-0.19	0.02	0.116

S6 Table Associations of longer-term moderate-to-vigorous physical activity (mean of MVPA measures at age 12y, 14y, and 15y) with metabolic traits at age 15y in ALSPAC

	**Mean of MVPA at age 12y, 14y, 15y (per SD (12 min/day) higher)**														
	Adj. for age, sex, ethnicity, maternal education, smoking, alcohol, mean wear time, wear month					*Additionally adj. for mean SED*					*Additionally adj. for mean FMI*				
**Standardised outcome at age 15y**	**N**	**Beta**	**LCL**	**UCL**	**P-value**	**N**	**Beta**	**LCL**	**UCL**	**P-value**	**N**	**Beta**	**LCL**	**UCL**	**P-value**
Total lipids in IDL (mmol/l)	755	-0.03	-0.12	0.07	0.589	755	-0.07	-0.18	0.03	0.157	755	-0.07	-0.18	0.03	0.186
Phospholipids in IDL (mmol/l)	755	-0.03	-0.12	0.07	0.611	755	-0.08	-0.18	0.03	0.141	755	-0.08	-0.19	0.03	0.140
Total cholesterol in IDL (mmol/l)	755	-0.02	-0.12	0.08	0.665	755	-0.07	-0.17	0.04	0.220	755	-0.06	-0.16	0.05	0.289
Cholesterol esters in IDL (mmol/l)	755	-0.03	-0.13	0.07	0.578	755	-0.07	-0.17	0.04	0.197	755	-0.06	-0.16	0.05	0.299
Free cholesterol in IDL (mmol/l)	755	-0.01	-0.10	0.09	0.907	755	-0.05	-0.16	0.05	0.306	755	-0.06	-0.16	0.05	0.285
Triglycerides in IDL (mmol/l)	755	-0.05	-0.14	0.04	0.283	755	-0.09	-0.19	0.01	0.063	755	-0.10	-0.20	0.00	0.043
Concentration of large LDL particles (mol/l)	755	-0.04	-0.13	0.06	0.444	755	-0.10	-0.20	0.01	0.066	755	-0.10	-0.20	0.01	0.074
Total lipids in large LDL (mmol/l)	755	-0.03	-0.13	0.06	0.525	755	-0.09	-0.19	0.02	0.100	755	-0.08	-0.19	0.02	0.117
Phospholipids in large LDL (mmol/l)	755	-0.03	-0.13	0.06	0.485	755	-0.09	-0.19	0.01	0.083	755	-0.09	-0.19	0.02	0.112
Total cholesterol in large LDL (mmol/l)	755	-0.03	-0.12	0.07	0.570	755	-0.08	-0.19	0.02	0.121	755	-0.08	-0.18	0.03	0.148
Cholesterol esters in large LDL (mmol/l)	755	-0.03	-0.13	0.06	0.511	755	-0.09	-0.19	0.02	0.103	755	-0.08	-0.19	0.03	0.135
Free cholesterol in large LDL (mmol/l)	755	-0.01	-0.11	0.08	0.771	755	-0.07	-0.17	0.04	0.197	755	-0.07	-0.17	0.04	0.198
Triglycerides in large LDL (mmol/l)	755	-0.04	-0.13	0.05	0.397	755	-0.09	-0.19	0.01	0.084	755	-0.10	-0.21	0.00	0.042
Concentration of medium LDL particles (mol/l)	755	-0.06	-0.15	0.04	0.261	755	-0.12	-0.23	-0.01	0.028	755	-0.12	-0.22	-0.01	0.037
Total lipids in medium LDL (mmol/l)	755	-0.04	-0.14	0.05	0.380	755	-0.10	-0.21	0.00	0.055	755	-0.10	-0.20	0.01	0.072
Phospholipids in medium LDL (mmol/l)	755	-0.04	-0.13	0.06	0.412	755	-0.10	-0.20	0.00	0.059	755	-0.09	-0.19	0.02	0.101
Total cholesterol in medium LDL (mmol/l)	755	-0.04	-0.14	0.06	0.398	755	-0.10	-0.20	0.01	0.066	755	-0.09	-0.20	0.01	0.088
Cholesterol esters in medium LDL (mmol/l)	755	-0.05	-0.14	0.05	0.346	755	-0.10	-0.21	0.00	0.056	755	-0.10	-0.21	0.01	0.076
Free cholesterol in medium LDL (mmol/l)	755	-0.02	-0.12	0.08	0.697	755	-0.08	-0.18	0.03	0.140	755	-0.07	-0.18	0.03	0.173
Triglycerides in medium LDL (mmol/l)	755	-0.04	-0.14	0.05	0.360	755	-0.10	-0.21	0.00	0.057	755	-0.12	-0.23	-0.01	0.030
Concentration of small LDL particles (mol/l)	755	-0.05	-0.15	0.05	0.304	755	-0.11	-0.22	-0.01	0.032	755	-0.11	-0.22	0.00	0.042
Total lipids in small LDL (mmol/l)	755	-0.04	-0.14	0.05	0.375	755	-0.10	-0.21	0.00	0.052	755	-0.10	-0.20	0.01	0.071
Phospholipids in small LDL (mmol/l)	755	-0.03	-0.12	0.07	0.555	755	-0.09	-0.19	0.01	0.091	755	-0.08	-0.18	0.02	0.127
Total cholesterol in small LDL (mmol/l)	755	-0.04	-0.14	0.06	0.406	755	-0.10	-0.20	0.01	0.065	755	-0.09	-0.20	0.01	0.087
Cholesterol esters in small LDL (mmol/l)	755	-0.05	-0.15	0.05	0.330	755	-0.11	-0.21	0.00	0.050	755	-0.10	-0.21	0.01	0.066
Free cholesterol in small LDL (mmol/l)	755	0.00	-0.10	0.09	0.924	755	-0.06	-0.16	0.05	0.287	755	-0.05	-0.15	0.06	0.377
Triglycerides in small LDL (mmol/l)	755	-0.08	-0.18	0.01	0.084	755	-0.14	-0.25	-0.03	0.010	755	-0.14	-0.25	-0.03	0.011
Concentration of very large HDL particles (mol/l)	755	0.14	0.03	0.24	0.010	755	0.12	0.01	0.23	0.036	755	0.07	-0.04	0.18	0.197
Total lipids in very large HDL (mmol/l)	755	0.13	0.03	0.24	0.012	755	0.12	0.00	0.23	0.041	755	0.07	-0.04	0.18	0.211
Phospholipids in very large HDL (mmol/l)	755	0.14	0.04	0.25	0.006	755	0.13	0.02	0.24	0.023	755	0.08	-0.03	0.19	0.155
Total cholesterol in very large HDL (mmol/l)	755	0.12	0.01	0.22	0.029	755	0.10	-0.01	0.21	0.088	755	0.06	-0.05	0.17	0.315
Cholesterol esters in very large HDL (mmol/l)	755	0.11	0.01	0.22	0.040	755	0.09	-0.02	0.20	0.113	755	0.05	-0.06	0.16	0.354
Free cholesterol in very large HDL (mmol/l)	755	0.13	0.02	0.23	0.017	755	0.11	0.00	0.22	0.053	755	0.06	-0.04	0.17	0.247
Triglycerides in very large HDL (mmol/l)	755	0.05	-0.05	0.14	0.365	755	0.05	-0.05	0.15	0.351	755	0.02	-0.08	0.13	0.641
Concentration of large HDL particles (mol/l)	755	0.18	0.08	0.28	6.74E-04	755	0.16	0.05	0.27	0.004	755	0.11	0.01	0.22	0.039
Total lipids in large HDL (mmol/l)	755	0.18	0.07	0.28	7.24E-04	755	0.16	0.05	0.27	0.004	755	0.11	0.01	0.22	0.040
Phospholipids in large HDL (mmol/l)	755	0.17	0.07	0.27	1.33E-03	755	0.14	0.04	0.25	0.009	755	0.10	-0.01	0.20	0.070
Total cholesterol in large HDL (mmol/l)	755	0.18	0.08	0.29	4.99E-04	755	0.17	0.06	0.28	2.01E-03	755	0.12	0.01	0.23	0.027
Cholesterol esters in large HDL (mmol/l)	755	0.19	0.08	0.29	4.60E-04	755	0.17	0.07	0.28	1.81E-03	755	0.12	0.02	0.23	0.025
Free cholesterol in large HDL (mmol/l)	755	0.18	0.08	0.28	7.17E-04	755	0.16	0.06	0.27	0.003	755	0.11	0.01	0.22	0.036
Triglycerides in large HDL (mmol/l)	755	0.11	0.01	0.21	0.026	755	0.11	0.01	0.22	0.039	755	0.09	-0.02	0.19	0.098
Concentration of medium HDL particles (mol/l)	755	0.14	0.04	0.23	0.005	755	0.10	0.00	0.20	0.058	755	0.09	-0.02	0.19	0.100
Total lipids in medium HDL (mmol/l)	755	0.14	0.04	0.24	0.004	755	0.10	0.00	0.21	0.049	755	0.09	-0.02	0.19	0.100
Phospholipids in medium HDL (mmol/l)	755	0.14	0.05	0.24	0.004	755	0.10	0.00	0.20	0.049	755	0.08	-0.02	0.19	0.104
Total cholesterol in medium HDL (mmol/l)	755	0.15	0.05	0.25	0.003	755	0.12	0.01	0.23	0.029	755	0.10	-0.01	0.20	0.074
Cholesterol esters in medium HDL (mmol/l)	755	0.15	0.05	0.25	0.004	755	0.12	0.01	0.23	0.028	755	0.10	-0.01	0.21	0.072
Free cholesterol in medium HDL (mmol/l)	755	0.14	0.04	0.24	0.006	755	0.10	0.00	0.21	0.048	755	0.09	-0.02	0.19	0.101
Triglycerides in medium HDL (mmol/l)	755	-0.06	-0.16	0.04	0.275	755	-0.08	-0.19	0.02	0.130	755	-0.05	-0.16	0.06	0.361

S6 Table Associations of longer-term moderate-to-vigorous physical activity (mean of MVPA measures at age 12y, 14y, and 15y) with metabolic traits at age 15y in ALSPAC

Mean of MVPA at age 12y, 14y, 15y (per SD (12 min/day) higher)

	Adj. for age, sex, ethnicity, maternal education, smoking, alcohol, mean wear time, wear month					*Additionally adj. for mean SED*					*Additionally adj. for mean FMI*				
**Standardised outcome at age 15y**	**N**	**Beta**	**LCL**	**UCL**	**P-value**	**N**	**Beta**	**LCL**	**UCL**	**P-value**	**N**	**Beta**	**LCL**	**UCL**	**P-value**
Concentration of small HDL particles (mol/l)	755	-0.01	-0.11	0.09	0.846	755	-0.06	-0.16	0.05	0.294	755	-0.04	-0.14	0.07	0.493
Total lipids in small HDL (mmol/l)	755	0.04	-0.05	0.14	0.358	755	-0.01	-0.11	0.09	0.814	755	0.00	-0.11	0.10	0.931
Phospholipids in small HDL (mmol/l)	755	-0.01	-0.10	0.09	0.917	755	-0.03	-0.14	0.07	0.539	755	-0.02	-0.13	0.09	0.726
Total cholesterol in small HDL (mmol/l)	755	0.10	0.01	0.19	0.024	755	0.04	-0.06	0.13	0.433	755	0.03	-0.06	0.13	0.501
Cholesterol esters in small HDL (mmol/l)	755	0.09	0.00	0.18	0.041	755	0.03	-0.07	0.12	0.572	755	0.02	-0.07	0.12	0.627
Free cholesterol in small HDL (mmol/l)	755	0.10	0.00	0.20	0.040	755	0.06	-0.04	0.17	0.224	755	0.06	-0.05	0.16	0.302
Triglycerides in small HDL (mmol/l)	755	-0.12	-0.22	-0.03	0.012	755	-0.14	-0.25	-0.04	0.006	755	-0.12	-0.23	-0.02	0.022
Phospholipids to total lipds ratio in chylomicrons and extremely large VLDL (%)	755	-0.06	-0.17	0.04	0.220	755	-0.08	-0.19	0.03	0.164	755	-0.08	-0.19	0.03	0.151
Total cholesterol to total lipids ratio in chylomicrons and extremely large VLDL (%)	755	0.03	-0.08	0.14	0.641	755	-0.01	-0.13	0.10	0.824	755	0.01	-0.10	0.13	0.849
Cholesterol esters to total lipids ratio in chylomicrons and extremely large VLDL (%)	755	0.06	-0.05	0.16	0.285	755	0.02	-0.09	0.13	0.755	755	0.04	-0.07	0.15	0.473
Free cholesterol to total lipids ratio in chylomicrons and extremely large VLDL (%)	755	-0.08	-0.19	0.04	0.185	755	-0.10	-0.22	0.03	0.119	755	-0.08	-0.20	0.04	0.203
Triglycerides to total lipids ratio in chylomicrons and extremely large VLDL (%)	755	-0.01	-0.09	0.07	0.809	755	0.02	-0.06	0.10	0.624	755	0.00	-0.08	0.08	0.963
Phospholipids to total lipds ratio in very large VLDL (%)	755	-0.09	-0.21	0.02	0.100	755	-0.14	-0.26	-0.02	0.022	755	-0.12	-0.24	0.00	0.047
Total cholesterol to total lipids ratio in very large VLDL (%)	755	0.13	0.00	0.26	0.046	755	0.11	-0.03	0.24	0.131	755	0.11	-0.03	0.26	0.128
Cholesterol esters to total lipids ratio in very large VLDL (%)	755	0.13	0.00	0.26	0.050	755	0.12	-0.03	0.27	0.108	755	0.12	-0.03	0.26	0.121
Free cholesterol to total lipids ratio in very large VLDL (%)	755	0.10	-0.04	0.23	0.156	755	0.07	-0.08	0.21	0.370	755	0.06	-0.09	0.20	0.432
Triglycerides to total lipids ratio in very large VLDL (%)	755	-0.10	-0.23	0.04	0.153	755	-0.06	-0.21	0.09	0.417	755	-0.06	-0.21	0.09	0.409
Phospholipids to total lipds ratio in large VLDL (%)	755	-0.10	-0.23	0.03	0.130	755	-0.12	-0.26	0.02	0.084	755	-0.11	-0.25	0.03	0.135
Total cholesterol to total lipids ratio in large VLDL (%)	755	-0.01	-0.13	0.11	0.906	755	-0.05	-0.18	0.08	0.471	755	-0.02	-0.16	0.11	0.710
Cholesterol esters to total lipids ratio in large VLDL (%)	755	0.07	-0.04	0.18	0.213	755	0.04	-0.06	0.14	0.425	755	0.07	-0.06	0.21	0.292
Free cholesterol to total lipids ratio in large VLDL (%)	755	-0.11	-0.22	0.01	0.063	755	-0.13	-0.26	-0.01	0.036	755	-0.11	-0.24	0.02	0.085
Triglycerides to total lipids ratio in large VLDL (%)	755	0.05	-0.06	0.16	0.367	755	0.04	-0.05	0.14	0.393	755	0.07	-0.07	0.21	0.323
Phospholipids to total lipds ratio in medium VLDL (%)	755	0.11	-0.02	0.24	0.092	755	0.07	-0.06	0.21	0.306	755	0.03	-0.10	0.17	0.639
Total cholesterol to total lipids ratio in medium VLDL (%)	755	0.07	-0.04	0.17	0.206	755	0.03	-0.08	0.14	0.601	755	0.03	-0.08	0.14	0.548
Cholesterol esters to total lipids ratio in medium VLDL (%)	755	0.09	-0.01	0.20	0.080	755	0.06	-0.05	0.17	0.268	755	0.07	-0.04	0.18	0.229
Free cholesterol to total lipids ratio in medium VLDL (%)	755	-0.05	-0.16	0.05	0.320	755	-0.10	-0.21	0.01	0.086	755	-0.10	-0.21	0.01	0.082
Triglycerides to total lipids ratio in medium VLDL (%)	755	-0.08	-0.19	0.02	0.114	755	-0.04	-0.15	0.07	0.457	755	-0.04	-0.15	0.07	0.495
Phospholipids to total lipds ratio in small VLDL (%)	755	0.17	0.06	0.27	1.68E-03	755	0.17	0.06	0.28	0.003	755	0.15	0.03	0.26	0.011
Total cholesterol to total lipids ratio in small VLDL (%)	755	0.04	-0.07	0.15	0.503	755	0.01	-0.11	0.13	0.846	755	0.01	-0.11	0.13	0.819
Cholesterol esters to total lipids ratio in small VLDL (%)	755	0.01	-0.10	0.12	0.872	755	-0.01	-0.13	0.10	0.835	755	0.00	-0.12	0.11	0.939
Free cholesterol to total lipids ratio in small VLDL (%)	755	0.19	0.08	0.29	4.26E-04	755	0.15	0.04	0.26	0.006	755	0.12	0.01	0.23	0.027
Triglycerides to total lipids ratio in small VLDL (%)	755	-0.09	-0.19	0.02	0.124	755	-0.06	-0.18	0.06	0.303	755	-0.06	-0.18	0.06	0.350
Phospholipids to total lipds ratio in very small VLDL (%)	755	0.03	-0.07	0.13	0.555	755	-0.01	-0.12	0.09	0.788	755	-0.04	-0.14	0.07	0.503
Total cholesterol to total lipids ratio in very small VLDL (%)	755	0.05	-0.06	0.15	0.379	755	0.08	-0.03	0.20	0.159	755	0.10	-0.02	0.21	0.100
Cholesterol esters to total lipids ratio in very small VLDL (%)	755	-0.04	-0.14	0.05	0.404	755	-0.01	-0.11	0.09	0.845	755	0.01	-0.09	0.12	0.800
Free cholesterol to total lipids ratio in very small VLDL (%)	755	0.20	0.09	0.32	3.28E-04	755	0.23	0.10	0.36	3.66E-04	755	0.22	0.09	0.35	1.13E-03
Triglycerides to total lipids ratio in very small VLDL (%)	755	-0.08	-0.18	0.03	0.154	755	-0.09	-0.20	0.03	0.141	755	-0.09	-0.21	0.03	0.138
Phospholipids to total lipds ratio in IDL (%)	755	0.03	-0.09	0.14	0.639	755	0.01	-0.11	0.13	0.832	755	-0.02	-0.14	0.10	0.774
Total cholesterol to total lipids ratio in IDL (%)	755	0.00	-0.10	0.11	0.955	755	0.01	-0.10	0.12	0.869	755	0.04	-0.08	0.15	0.548
Cholesterol esters to total lipids ratio in IDL (%)	755	-0.03	-0.14	0.07	0.544	755	-0.02	-0.14	0.09	0.699	755	0.02	-0.10	0.13	0.797
Free cholesterol to total lipids ratio in IDL (%)	755	0.08	-0.02	0.18	0.109	755	0.07	-0.03	0.18	0.174	755	0.04	-0.06	0.15	0.402
Triglycerides to total lipids ratio in IDL (%)	755	-0.02	-0.12	0.08	0.736	755	-0.02	-0.13	0.09	0.741	755	-0.03	-0.14	0.07	0.529
Phospholipids to total lipds ratio in large LDL (%)	755	0.01	-0.09	0.10	0.912	755	0.03	-0.07	0.13	0.587	755	0.04	-0.06	0.14	0.474
Total cholesterol to total lipids ratio in large LDL (%)	755	-0.01	-0.10	0.09	0.902	755	-0.02	-0.13	0.08	0.649	755	-0.02	-0.12	0.09	0.772
Cholesterol esters to total lipids ratio in large LDL (%)	755	-0.03	-0.13	0.07	0.561	755	-0.06	-0.16	0.05	0.309	755	-0.04	-0.15	0.06	0.428
Free cholesterol to total lipids ratio in large LDL (%)	755	0.07	-0.02	0.16	0.129	755	0.10	0.01	0.20	0.038	755	0.09	-0.01	0.19	0.072
Triglycerides to total lipids ratio in large LDL (%)	755	0.00	-0.09	0.10	0.923	755	0.01	-0.10	0.12	0.851	755	-0.02	-0.12	0.09	0.786
Phospholipids to total lipds ratio in medium LDL (%)	755	0.01	-0.03	0.04	0.763	755	0.02	-0.02	0.06	0.457	755	0.02	-0.02	0.06	0.375

S6 Table Associations of longer-term moderate-to-vigorous physical activity (mean of MVPA measures at age 12y, 14y, and 15y) with metabolic traits at age 15y in ALSPAC

	Mean of MVPA at age 12y, 14y, 15y (per SD (12 min/day) higher)														
	Adj. for age, sex, ethnicity, maternal education, smoking, alcohol, mean wear time, wear month					*Additionally adj. for mean SED*					*Additionally adj. for mean FMI*				
**Standardised outcome at age 15y**	**N**	**Beta**	**LCL**	**UCL**	**P-value**	**N**	**Beta**	**LCL**	**UCL**	**P-value**	**N**	**Beta**	**LCL**	**UCL**	**P-value**
Total cholesterol to total lipids ratio in medium LDL (%)	755	-0.02	-0.12	0.08	0.707	755	-0.04	-0.15	0.08	0.536	755	-0.03	-0.15	0.08	0.586
Cholesterol esters to total lipids ratio in medium LDL (%)	755	-0.03	-0.14	0.07	0.556	755	-0.06	-0.17	0.06	0.331	755	-0.05	-0.17	0.06	0.361
Free cholesterol to total lipids ratio in medium LDL (%)	755	0.01	-0.02	0.04	0.423	755	0.02	-0.01	0.05	0.176	755	0.02	-0.01	0.05	0.187
Triglycerides to total lipids ratio in medium LDL (%)	755	0.01	-0.09	0.11	0.803	755	-0.01	-0.11	0.10	0.912	755	-0.03	-0.14	0.07	0.542
Phospholipids to total lipds ratio in small LDL (%)	755	0.02	-0.04	0.08	0.512	755	0.04	-0.03	0.10	0.248	755	0.04	-0.03	0.10	0.253
Total cholesterol to total lipids ratio in small LDL (%)	755	-0.01	-0.11	0.09	0.812	755	-0.03	-0.14	0.08	0.556	755	-0.03	-0.14	0.08	0.588
Cholesterol esters to total lipids ratio in small LDL (%)	755	-0.03	-0.13	0.07	0.583	755	-0.06	-0.17	0.05	0.306	755	-0.06	-0.17	0.06	0.320
Free cholesterol to total lipids ratio in small LDL (%)	755	0.03	-0.03	0.08	0.334	755	0.05	-0.01	0.11	0.119	755	0.05	-0.01	0.11	0.119
Triglycerides to total lipids ratio in small LDL (%)	755	-0.06	-0.16	0.04	0.269	755	-0.08	-0.19	0.03	0.150	755	-0.09	-0.19	0.02	0.125
Phospholipids to total lipds ratio in very large HDL (%)	755	0.13	0.03	0.23	0.009	755	0.13	0.02	0.23	0.016	755	0.08	-0.02	0.18	0.115
Total cholesterol to total lipids ratio in very large HDL (%)	755	-0.12	-0.22	-0.03	0.013	755	-0.12	-0.23	-0.02	0.020	755	-0.08	-0.18	0.02	0.125
Cholesterol esters to total lipids ratio in very large HDL (%)	755	-0.12	-0.22	-0.02	0.015	755	-0.12	-0.22	-0.02	0.022	755	-0.08	-0.18	0.02	0.137
Free cholesterol to total lipids ratio in very large HDL (%)	755	0.03	-0.07	0.13	0.582	755	0.03	-0.07	0.14	0.536	755	0.01	-0.09	0.11	0.839
Triglycerides to total lipids ratio in very large HDL (%)	755	-0.08	-0.19	0.03	0.162	755	-0.06	-0.17	0.05	0.284	755	-0.04	-0.14	0.07	0.503
Phospholipids to total lipds ratio in large HDL (%)	755	-0.15	-0.24	-0.05	2.57E-03	755	-0.19	-0.29	-0.08	4.05E-04	755	-0.14	-0.24	-0.03	0.009
Total cholesterol to total lipids ratio in large HDL (%)	755	0.15	0.05	0.25	0.003	755	0.17	0.07	0.28	1.19E-03	755	0.12	0.02	0.23	0.021
Cholesterol esters to total lipids ratio in large HDL (%)	755	0.15	0.05	0.25	0.003	755	0.18	0.07	0.28	1.02E-03	755	0.12	0.02	0.23	0.020
Free cholesterol to total lipids ratio in large HDL (%)	755	0.11	0.01	0.20	0.032	755	0.12	0.02	0.23	0.021	755	0.09	-0.02	0.19	0.103
Triglycerides to total lipids ratio in large HDL (%)	755	-0.12	-0.23	-0.01	0.038	755	-0.10	-0.21	0.01	0.071	755	-0.05	-0.16	0.05	0.316
Phospholipids to total lipds ratio in medium HDL (%)	755	0.09	-0.01	0.20	0.065	755	0.07	-0.04	0.17	0.236	755	0.05	-0.06	0.16	0.357
Total cholesterol to total lipids ratio in medium HDL (%)	755	0.00	-0.10	0.10	0.965	755	0.03	-0.08	0.14	0.613	755	0.01	-0.10	0.12	0.805
Cholesterol esters to total lipids ratio in medium HDL (%)	755	0.00	-0.10	0.11	0.993	755	0.03	-0.08	0.14	0.618	755	0.01	-0.10	0.12	0.811
Free cholesterol to total lipids ratio in medium HDL (%)	755	-0.01	-0.14	0.11	0.836	755	0.01	-0.10	0.13	0.814	755	0.01	-0.11	0.13	0.854
Triglycerides to total lipids ratio in medium HDL (%)	755	-0.12	-0.23	-0.02	0.024	755	-0.14	-0.25	-0.02	0.020	755	-0.10	-0.21	0.02	0.102
Phospholipids to total lipds ratio in small HDL (%)	755	-0.10	-0.19	-0.01	0.037	755	-0.04	-0.14	0.05	0.385	755	-0.03	-0.13	0.07	0.519
Total cholesterol to total lipids ratio in small HDL (%)	755	0.13	0.04	0.22	0.006	755	0.08	-0.02	0.18	0.121	755	0.06	-0.04	0.16	0.217
Cholesterol esters to total lipids ratio in small HDL (%)	755	0.10	0.01	0.19	0.029	755	0.05	-0.05	0.15	0.337	755	0.04	-0.06	0.14	0.440
Free cholesterol to total lipids ratio in small HDL (%)	755	0.14	0.04	0.25	0.005	755	0.19	0.08	0.30	9.23E-04	755	0.15	0.04	0.26	0.008
Triglycerides to total lipids ratio in small HDL (%)	755	-0.16	-0.26	-0.06	1.62E-03	755	-0.16	-0.27	-0.05	0.003	755	-0.14	-0.25	-0.03	0.012
Mean diameter for VLDL particles (nm)	755	-0.14	-0.24	-0.03	0.010	755	-0.14	-0.25	-0.03	0.017	755	-0.10	-0.22	0.01	0.070
Mean diameter for LDL particles (nm)	755	0.05	-0.04	0.14	0.302	755	0.09	0.00	0.19	0.058	755	0.08	-0.02	0.18	0.102
Mean diameter for HDL particles (nm)	755	0.16	0.06	0.27	2.84E-03	755	0.16	0.04	0.27	0.007	755	0.11	0.00	0.22	0.060
Serum total cholesterol (mmol/l)	755	0.01	-0.08	0.11	0.820	755	-0.04	-0.14	0.06	0.417	755	-0.04	-0.14	0.06	0.419
Total cholesterol in VLDL (mmol/l)	755	-0.11	-0.21	-0.01	0.037	755	-0.13	-0.23	-0.02	0.019	755	-0.08	-0.19	0.02	0.113
Remnant cholesterol (non-HDL, non-LDL -cholesterol) (mmol/l)	755	-0.08	-0.18	0.02	0.136	755	-0.11	-0.22	0.00	0.043	755	-0.08	-0.19	0.03	0.139
Total cholesterol in LDL (mmol/l)	755	-0.03	-0.13	0.06	0.481	755	-0.09	-0.19	0.01	0.089	755	-0.09	-0.19	0.02	0.114
Total cholesterol in HDL (mmol/l)	755	0.17	0.07	0.27	8.66E-04	755	0.14	0.03	0.25	0.010	755	0.10	-0.01	0.20	0.064
Total cholesterol in HDL2 (mmol/l)	755	0.17	0.07	0.27	1.08E-03	755	0.14	0.03	0.25	0.010	755	0.10	-0.01	0.20	0.067
Total cholesterol in HDL3 (mmol/l)	755	0.16	0.06	0.26	1.07E-03	755	0.13	0.03	0.23	0.014	755	0.09	-0.01	0.19	0.073
Esterified cholesterol (mmol/l)	755	0.02	-0.08	0.11	0.728	755	-0.04	-0.14	0.06	0.478	755	-0.04	-0.14	0.06	0.463
Free cholesterol (mmol/l)	755	0.00	-0.10	0.09	0.957	755	-0.05	-0.16	0.05	0.322	755	-0.05	-0.15	0.06	0.355
Serum total triglycerides (mmol/l)	755	-0.13	-0.23	-0.03	0.009	755	-0.15	-0.25	-0.04	0.006	755	-0.12	-0.22	-0.01	0.028
Triglycerides in VLDL (mmol/l)	755	-0.14	-0.24	-0.04	0.006	755	-0.14	-0.25	-0.04	0.007	755	-0.11	-0.21	0.00	0.045
Triglycerides in LDL (mmol/l)	755	-0.05	-0.14	0.04	0.295	755	-0.10	-0.20	0.00	0.049	755	-0.12	-0.22	-0.01	0.028
Triglycerides in HDL (mmol/l)	755	-0.04	-0.14	0.06	0.422	755	-0.06	-0.16	0.04	0.257	755	-0.05	-0.15	0.06	0.374
Diacylglycerol (mmol/l)	755	-0.02	-0.12	0.07	0.644	755	-0.02	-0.12	0.08	0.755	755	0.00	-0.10	0.11	0.944
Ratio of diacylglycerol to triglycerides	755	0.01	-0.09	0.12	0.816	755	0.01	-0.10	0.13	0.804	755	0.02	-0.09	0.13	0.734
Total phosphoglycerides (mmol/l)	755	0.08	-0.01	0.17	0.078	755	0.03	-0.07	0.13	0.516	755	0.01	-0.08	0.11	0.782

S6 Table Associations of longer-term moderate-to-vigorous physical activity (mean of MVPA measures at age 12y, 14y, and 15y) with metabolic traits at age 15y in ALSPAC

Mean of MVPA at age 12y, 14y, 15y (per SD (12 min/day) higher)

	Adj. for age, sex, ethnicity, maternal education, smoking, alcohol, mean wear time, wear month					*Additionally adj. for mean SED*					*Additionally adj. for mean FMI*				
**Standardised outcome at age 15y**	**N**	**Beta**	**LCL**	**UCL**	**P-value**	**N**	**Beta**	**LCL**	**UCL**	**P-value**	**N**	**Beta**	**LCL**	**UCL**	**P-value**
Ratio of triglycerides to phosphoglycerides	755	-0.14	-0.23	-0.04	0.005	755	-0.12	-0.22	-0.02	0.016	755	-0.08	-0.18	0.01	0.094
Phosphatidylcholine and other cholines (mmol/l)	755	0.09	0.00	0.18	0.054	755	0.05	-0.05	0.14	0.331	755	0.03	-0.07	0.13	0.522
Total cholines (mmol/l)	755	0.08	-0.01	0.17	0.093	755	0.03	-0.07	0.12	0.590	755	0.01	-0.09	0.11	0.840
Apolipoprotein A-I (g/l)	755	0.13	0.03	0.22	0.010	755	0.09	-0.02	0.19	0.097	755	0.06	-0.05	0.16	0.279
Apolipoprotein B (g/l)	755	-0.11	-0.21	-0.01	0.035	755	-0.14	-0.25	-0.04	0.008	755	-0.12	-0.23	-0.01	0.033
Ratio of apolipoprotein B to apolipoprotein A-I	755	-0.17	-0.27	-0.06	2.53E-03	755	-0.18	-0.30	-0.07	1.76E-03	755	-0.14	-0.25	-0.03	0.016
Total fatty acids (mmol/l)	755	-0.03	-0.12	0.06	0.509	755	-0.07	-0.17	0.03	0.152	755	-0.06	-0.16	0.04	0.213
Estimated description of fatty acid chain length, not actual carbon number	755	-0.02	-0.12	0.08	0.692	755	0.01	-0.10	0.11	0.880	755	0.02	-0.08	0.12	0.717
Estimated degree of unsaturation	755	0.01	-0.09	0.11	0.851	755	0.03	-0.08	0.13	0.639	755	0.03	-0.08	0.14	0.599
22:6, docosahexaenoic acid (mmol/l)	755	0.00	-0.11	0.11	0.953	755	-0.02	-0.13	0.09	0.746	755	-0.01	-0.13	0.10	0.841
18:2, linoleic acid (mmol/l)	755	0.00	-0.09	0.09	0.927	755	-0.06	-0.16	0.04	0.243	755	-0.07	-0.17	0.03	0.195
Conjugated linoleic acid (mmol/l)	755	-0.02	-0.11	0.07	0.691	755	-0.01	-0.10	0.08	0.791	755	-0.02	-0.11	0.08	0.714
Omega-3 fatty acids (mmol/l)	755	-0.02	-0.13	0.08	0.654	755	-0.05	-0.17	0.06	0.375	755	-0.05	-0.17	0.07	0.416
Omega-6 fatty acids (mmol/l)	755	0.00	-0.10	0.09	0.925	755	-0.05	-0.15	0.05	0.299	755	-0.05	-0.16	0.05	0.289
Polyunsaturated fatty acids (mmol/l)	755	-0.01	-0.10	0.09	0.877	755	-0.06	-0.16	0.05	0.280	755	-0.06	-0.16	0.05	0.277
Monounsaturated fatty acids; 16:1, 18:1 (mmol/l)	755	-0.07	-0.16	0.03	0.152	755	-0.10	-0.20	0.00	0.054	755	-0.08	-0.18	0.02	0.137
Saturated fatty acids (mmol/l)	755	-0.01	-0.11	0.09	0.812	755	-0.05	-0.16	0.06	0.395	755	-0.04	-0.15	0.07	0.445
Ratio of 22:6 docosahexaenoic acid to total fatty acids (%)	755	0.02	-0.09	0.14	0.689	755	0.02	-0.10	0.15	0.708	755	0.03	-0.10	0.15	0.676
Ratio of 18:2 linoleic acid to total fatty acids (%)	755	0.05	-0.06	0.15	0.395	755	0.03	-0.09	0.15	0.612	755	0.01	-0.11	0.13	0.913
Ratio of conjugated linoleic acid to total fatty acids (%)	755	-0.03	-0.12	0.06	0.523	755	-0.02	-0.11	0.08	0.720	755	-0.02	-0.11	0.07	0.644
Ratio of omega-3 fatty acids to total fatty acids (%)	755	-0.01	-0.12	0.10	0.844	755	-0.01	-0.14	0.12	0.866	755	-0.02	-0.14	0.11	0.813
Ratio of omega-6 fatty acids to total fatty acids (%)	755	0.06	-0.05	0.16	0.300	755	0.05	-0.06	0.17	0.354	755	0.03	-0.08	0.15	0.563
Ratio of polyunsaturated fatty acids to total fatty acids (%)	755	0.05	-0.05	0.16	0.343	755	0.05	-0.07	0.17	0.396	755	0.03	-0.09	0.15	0.619
Ratio of monounsaturated fatty acids to total fatty acids (%)	755	-0.08	-0.19	0.03	0.163	755	-0.08	-0.20	0.04	0.198	755	-0.05	-0.17	0.07	0.405
Ratio of saturated fatty acids to total fatty acids (%)	755	0.04	-0.07	0.15	0.474	755	0.04	-0.08	0.16	0.507	755	0.03	-0.09	0.15	0.632
Insulin (mu/l)	755	-0.14	-0.20	-0.07	3.33E-05	755	-0.13	-0.20	-0.06	1.59E-04	755	-0.10	-0.16	-0.03	0.005
Glucose (mmol/l)	755	-0.07	-0.16	0.03	0.158	755	-0.07	-0.16	0.03	0.194	755	-0.06	-0.16	0.04	0.221
Lactate (mmol/l)	755	-0.05	-0.16	0.05	0.325	755	-0.04	-0.15	0.08	0.546	755	-0.04	-0.16	0.07	0.460
Pyruvate (mmol/l)	755	-0.10	-0.20	0.01	0.064	755	-0.05	-0.16	0.06	0.381	755	-0.04	-0.15	0.07	0.505
Citrate (mmol/l)	755	0.11	0.00	0.22	0.051	755	0.05	-0.06	0.17	0.360	755	0.03	-0.08	0.15	0.597
Alanine (mmol/l)	755	-0.08	-0.18	0.02	0.121	755	-0.02	-0.13	0.08	0.666	755	-0.03	-0.13	0.08	0.624
Glutamine (mmol/l)	755	0.03	-0.07	0.12	0.591	755	0.03	-0.08	0.13	0.638	755	0.00	-0.11	0.10	0.952
Histidine (mmol/l)	755	0.08	-0.02	0.18	0.120	755	0.11	0.00	0.22	0.044	755	0.11	0.00	0.22	0.057
Isoleucine (mmol/l)	755	-0.02	-0.12	0.08	0.720	755	0.00	-0.10	0.11	0.927	755	0.02	-0.08	0.13	0.667
Leucine (mmol/l)	755	0.08	-0.01	0.17	0.090	755	0.09	-0.01	0.19	0.071	755	0.10	0.00	0.19	0.060
Valine (mmol/l)	755	0.01	-0.09	0.11	0.889	755	0.01	-0.11	0.12	0.897	755	0.02	-0.09	0.14	0.690
Phenylalanine (mmol/l)	755	0.11	0.01	0.21	0.037	755	0.13	0.02	0.23	0.022	755	0.14	0.03	0.25	0.011
Tyrosine (mmol/l)	755	0.05	-0.06	0.17	0.349	755	0.05	-0.07	0.17	0.424	755	0.07	-0.06	0.19	0.297
Acetate (mmol/l)	755	0.12	0.01	0.22	0.029	755	0.11	0.00	0.23	0.053	755	0.10	-0.01	0.22	0.073
Acetoacetate (mmol/l)	755	-0.06	-0.14	0.03	0.176	755	-0.05	-0.15	0.04	0.250	755	-0.05	-0.14	0.04	0.310
3-hydroxybutyrate (mmol/l)	755	-0.12	-0.21	-0.02	0.017	755	-0.13	-0.24	-0.03	0.013	755	-0.13	-0.23	-0.02	0.017
Creatinine (mmol/l)	755	-0.03	-0.12	0.07	0.602	755	0.02	-0.08	0.12	0.686	755	0.02	-0.08	0.12	0.654
Albumin (signal area)	755	-0.09	-0.19	0.01	0.092	755	-0.05	-0.17	0.06	0.335	755	-0.06	-0.17	0.05	0.285
Glycoprotein acetyls, mainly a1-acid glycoprotein (mmol/l)	755	-0.20	-0.30	-0.11	1.11E-05	755	-0.19	-0.29	-0.10	7.04E-05	755	-0.14	-0.23	-0.05	0.003
C-reactive protein (mg/l)	755	-0.04	-0.10	0.02	0.148	755	0.00	-0.07	0.07	0.993	755	0.01	-0.06	0.07	0.791
