# Supplementary material for: Associations of device-measured physical activity across adolescence with metabolic traits: Prospective cohort study
Source: PLoS Med. 2018 Sep 11;15(9):e1002649. doi: 10.1371/journal.pmed.1002649 (PMC6133272; doi:10.1371/journal.pmed.1002649)
Supplement: S7 Table — ALSPAC, Avon Longitudinal Study of Parents and Children; SED, sedentary time. (PDF) [file pmed.1002649.s007.pdf]

S7 Table Associations of longer-term sedentary time (mean of SED measures at age 12y, 14y, and 15y) with metabolic traits at age 15y in ALSPAC

Mean of SED at age 12y, 14y, 15y (per SD (50 min/day) higher)

	Adj. for age, sex, ethnicity, maternal education, smoking, alcohol, mean wear time, wear month					*Additionally adj. for mean MVPA*					*Additionally adj. for mean FMI*				
**Standardised outcome at age 15y**	**N**	**Beta**	**LCL**	**UCL**	**P-value**	**N**	**Beta**	**LCL**	**UCL**	**P-value**	**N**	**Beta**	**LCL**	**UCL**	**P-value**
Systolic blood pressure (mmHg)	1298	0.04	-0.05	0.13	0.375	1298	-0.02	-0.11	0.07	0.697	1256	-0.02	-0.12	0.07	0.639
Diastolic blood pressure (mmHg)	1298	0.08	0.00	0.16	0.053	1298	0.07	-0.02	0.16	0.113	1256	0.07	-0.02	0.17	0.136
Concentration of chylomicrons and extremely large VLDL particles (mol/l)	896	0.09	-0.02	0.20	0.091	896	0.00	-0.11	0.12	0.956	874	-0.04	-0.15	0.07	0.441
Total lipids in chylomicrons and extremely large VLDL (mmol/l)	896	0.09	-0.02	0.20	0.092	896	0.01	-0.11	0.12	0.924	874	-0.04	-0.15	0.07	0.464
Phospholipids in chylomicrons and extremely large VLDL (mmol/l)	896	0.09	-0.02	0.20	0.097	896	0.00	-0.11	0.12	0.964	874	-0.04	-0.15	0.07	0.438
Total cholesterol in chylomicrons and extremely large VLDL (mmol/l)	896	0.07	-0.04	0.18	0.198	896	-0.01	-0.11	0.10	0.908	874	-0.05	-0.15	0.06	0.366
Cholesterol esters in chylomicrons and extremely large VLDL (mmol/l)	896	0.05	-0.06	0.15	0.402	896	-0.02	-0.12	0.09	0.761	874	-0.05	-0.15	0.05	0.320
Free cholesterol in chylomicrons and extremely large VLDL (mmol/l)	896	0.09	-0.02	0.20	0.095	896	0.00	-0.11	0.12	0.933	874	-0.04	-0.15	0.07	0.460
Triglycerides in chylomicrons and extremely large VLDL (mmol/l)	896	0.10	-0.01	0.21	0.077	896	0.01	-0.10	0.12	0.881	874	-0.04	-0.15	0.07	0.496
Concentration of very large VLDL particles (mol/l)	896	0.10	-0.01	0.20	0.079	896	0.01	-0.10	0.12	0.806	874	-0.03	-0.14	0.07	0.535
Total lipids in very large VLDL (mmol/l)	896	0.09	-0.02	0.20	0.095	896	0.01	-0.10	0.12	0.847	874	-0.04	-0.14	0.07	0.509
Phospholipids in very large VLDL (mmol/l)	896	0.09	-0.02	0.19	0.119	896	0.00	-0.11	0.11	0.943	874	-0.04	-0.15	0.07	0.450
Total cholesterol in very large VLDL (mmol/l)	896	0.08	-0.02	0.19	0.128	896	0.00	-0.11	0.11	0.976	874	-0.04	-0.15	0.06	0.428
Cholesterol esters in very large VLDL (mmol/l)	896	0.08	-0.02	0.19	0.125	896	0.01	-0.10	0.11	0.917	874	-0.04	-0.14	0.07	0.473
Free cholesterol in very large VLDL (mmol/l)	896	0.08	-0.03	0.19	0.138	896	0.00	-0.11	0.11	0.960	874	-0.05	-0.15	0.06	0.391
Triglycerides in very large VLDL (mmol/l)	896	0.09	-0.01	0.20	0.083	896	0.02	-0.09	0.12	0.783	874	-0.03	-0.14	0.07	0.557
Concentration of large VLDL particles (mol/l)	896	0.09	-0.02	0.19	0.106	896	0.01	-0.10	0.12	0.823	874	-0.03	-0.14	0.07	0.531
Total lipids in large VLDL (mmol/l)	896	0.08	-0.02	0.19	0.121	896	0.01	-0.10	0.12	0.856	874	-0.04	-0.14	0.07	0.508
Phospholipids in large VLDL (mmol/l)	896	0.08	-0.03	0.19	0.140	896	0.01	-0.10	0.11	0.925	874	-0.04	-0.14	0.06	0.453
Total cholesterol in large VLDL (mmol/l)	896	0.07	-0.04	0.17	0.215	896	0.00	-0.11	0.11	0.949	874	-0.05	-0.15	0.06	0.374
Cholesterol esters in large VLDL (mmol/l)	896	0.06	-0.05	0.16	0.309	896	-0.01	-0.12	0.10	0.855	874	-0.05	-0.16	0.05	0.338
Free cholesterol in large VLDL (mmol/l)	896	0.08	-0.03	0.18	0.153	896	0.00	-0.11	0.11	0.961	874	-0.04	-0.15	0.06	0.420
Triglycerides in large VLDL (mmol/l)	896	0.09	-0.01	0.20	0.092	896	0.02	-0.09	0.13	0.764	874	-0.03	-0.13	0.08	0.586
Concentration of medium VLDL particles (mol/l)	896	0.08	-0.03	0.19	0.142	896	0.00	-0.11	0.12	0.934	874	-0.04	-0.15	0.07	0.467
Total lipids in medium VLDL (mmol/l)	896	0.07	-0.03	0.18	0.179	896	0.00	-0.11	0.11	0.992	874	-0.04	-0.15	0.06	0.431
Phospholipids in medium VLDL (mmol/l)	896	0.06	-0.04	0.17	0.250	896	-0.01	-0.12	0.10	0.853	874	-0.05	-0.16	0.05	0.336
Total cholesterol in medium VLDL (mmol/l)	896	0.04	-0.07	0.14	0.507	896	-0.02	-0.13	0.09	0.687	874	-0.06	-0.17	0.05	0.273
Cholesterol esters in medium VLDL (mmol/l)	896	0.01	-0.09	0.12	0.808	896	-0.03	-0.14	0.08	0.585	874	-0.06	-0.17	0.05	0.261
Free cholesterol in medium VLDL (mmol/l)	896	0.06	-0.05	0.17	0.264	896	-0.01	-0.12	0.10	0.842	874	-0.05	-0.16	0.05	0.332
Triglycerides in medium VLDL (mmol/l)	896	0.09	-0.01	0.20	0.090	896	0.02	-0.09	0.13	0.782	874	-0.03	-0.13	0.08	0.584
Concentration of small VLDL particles (mol/l)	896	0.03	-0.08	0.14	0.577	896	-0.05	-0.16	0.07	0.426	874	-0.08	-0.19	0.03	0.153
Total lipids in small VLDL (mmol/l)	896	0.02	-0.09	0.12	0.776	896	-0.06	-0.18	0.05	0.291	874	-0.09	-0.20	0.02	0.103
Phospholipids in small VLDL (mmol/l)	896	-0.01	-0.12	0.09	0.826	896	-0.08	-0.19	0.03	0.152	874	-0.11	-0.22	0.00	0.046
Total cholesterol in small VLDL (mmol/l)	896	-0.02	-0.13	0.09	0.721	896	-0.09	-0.21	0.03	0.130	874	-0.11	-0.23	0.00	0.056
Cholesterol esters in small VLDL (mmol/l)	896	-0.02	-0.13	0.09	0.715	896	-0.09	-0.21	0.03	0.141	874	-0.11	-0.23	0.01	0.072
Free cholesterol in small VLDL (mmol/l)	896	-0.02	-0.12	0.09	0.752	896	-0.08	-0.20	0.03	0.143	874	-0.11	-0.22	0.00	0.050
Triglycerides in small VLDL (mmol/l)	896	0.06	-0.05	0.16	0.313	896	-0.02	-0.13	0.09	0.718	874	-0.06	-0.17	0.05	0.316
Concentration of very small VLDL particles (mol/l)	896	-0.07	-0.17	0.03	0.184	896	-0.10	-0.22	0.01	0.069	874	-0.11	-0.22	0.01	0.063
Total lipids in very small VLDL (mmol/l)	896	-0.05	-0.16	0.06	0.370	896	-0.09	-0.21	0.02	0.118	874	-0.10	-0.22	0.02	0.101
Phospholipids in very small VLDL (mmol/l)	896	-0.08	-0.19	0.02	0.109	896	-0.12	-0.23	-0.01	0.039	874	-0.12	-0.23	0.00	0.043
Total cholesterol in very small VLDL (mmol/l)	896	-0.03	-0.14	0.08	0.594	896	-0.06	-0.18	0.06	0.339	874	-0.06	-0.18	0.06	0.322
Cholesterol esters in very small VLDL (mmol/l)	896	-0.01	-0.12	0.10	0.905	896	-0.05	-0.17	0.07	0.418	874	-0.06	-0.18	0.06	0.359
Free cholesterol in very small VLDL (mmol/l)	896	-0.08	-0.18	0.03	0.169	896	-0.07	-0.19	0.05	0.241	874	-0.06	-0.18	0.06	0.295
Triglycerides in very small VLDL (mmol/l)	896	-0.01	-0.12	0.09	0.789	896	-0.08	-0.20	0.03	0.152	874	-0.10	-0.21	0.01	0.084
Concentration of IDL particles (mol/l)	896	-0.09	-0.19	0.02	0.111	896	-0.12	-0.23	0.00	0.041	874	-0.12	-0.23	0.00	0.050
Total lipids in IDL (mmol/l)	896	-0.09	-0.19	0.02	0.096	896	-0.12	-0.23	-0.01	0.038	874	-0.12	-0.23	0.00	0.045
Phospholipids in IDL (mmol/l)	896	-0.10	-0.20	0.01	0.075	896	-0.13	-0.24	-0.01	0.029	874	-0.12	-0.24	-0.01	0.037
Total cholesterol in IDL (mmol/l)	896	-0.08	-0.19	0.02	0.127	896	-0.11	-0.22	0.00	0.057	874	-0.11	-0.23	0.01	0.063

S7 Table Associations of longer-term sedentary time (mean of SED measures at age 12y, 14y, and 15y) with metabolic traits at age 15y in ALSPAC

Mean of SED at age 12y, 14y, 15y (per SD (50 min/day) higher)

	Adj. for age, sex, ethnicity, maternal education, smoking, alcohol, mean wear time, wear month					*Additionally adj. for mean MVPA*					*Additionally adj. for mean FMI*				
**Standardised outcome at age 15y**	**N**	**Beta**	**LCL**	**UCL**	**P-value**	**N**	**Beta**	**LCL**	**UCL**	**P-value**	**N**	**Beta**	**LCL**	**UCL**	**P-value**
Cholesterol esters in IDL (mmol/l)	896	-0.07	-0.18	0.03	0.174	896	-0.11	-0.22	0.01	0.072	874	-0.11	-0.23	0.01	0.069
Free cholesterol in IDL (mmol/l)	896	-0.10	-0.20	0.01	0.064	896	-0.12	-0.23	-0.01	0.041	874	-0.11	-0.23	0.00	0.060
Triglycerides in IDL (mmol/l)	896	-0.08	-0.18	0.02	0.116	896	-0.11	-0.22	0.00	0.040	874	-0.11	-0.21	0.00	0.058
Concentration of large LDL particles (mol/l)	896	-0.10	-0.21	0.00	0.054	896	-0.15	-0.26	-0.03	0.013	874	-0.15	-0.26	-0.03	0.014
Total lipids in large LDL (mmol/l)	896	-0.10	-0.21	0.00	0.061	896	-0.14	-0.25	-0.02	0.018	874	-0.14	-0.25	-0.02	0.020
Phospholipids in large LDL (mmol/l)	896	-0.10	-0.21	0.00	0.055	896	-0.14	-0.26	-0.03	0.013	874	-0.15	-0.26	-0.03	0.014
Total cholesterol in large LDL (mmol/l)	896	-0.10	-0.20	0.01	0.070	896	-0.13	-0.25	-0.02	0.022	874	-0.13	-0.25	-0.02	0.025
Cholesterol esters in large LDL (mmol/l)	896	-0.09	-0.20	0.01	0.080	896	-0.13	-0.25	-0.02	0.023	874	-0.13	-0.25	-0.02	0.023
Free cholesterol in large LDL (mmol/l)	896	-0.11	-0.21	0.00	0.047	896	-0.13	-0.25	-0.02	0.021	874	-0.13	-0.24	-0.01	0.031
Triglycerides in large LDL (mmol/l)	896	-0.09	-0.19	0.02	0.095	896	-0.12	-0.23	-0.01	0.037	874	-0.11	-0.22	0.00	0.055
Concentration of medium LDL particles (mol/l)	896	-0.10	-0.21	0.01	0.072	896	-0.15	-0.27	-0.03	0.011	874	-0.16	-0.28	-0.04	0.010
Total lipids in medium LDL (mmol/l)	896	-0.10	-0.21	0.01	0.067	896	-0.14	-0.26	-0.03	0.014	874	-0.15	-0.26	-0.03	0.014
Phospholipids in medium LDL (mmol/l)	896	-0.10	-0.21	0.00	0.052	896	-0.15	-0.26	-0.04	0.008	874	-0.16	-0.27	-0.04	0.006
Total cholesterol in medium LDL (mmol/l)	896	-0.09	-0.20	0.01	0.084	896	-0.14	-0.25	-0.02	0.020	874	-0.14	-0.26	-0.02	0.021
Cholesterol esters in medium LDL (mmol/l)	896	-0.09	-0.20	0.02	0.107	896	-0.13	-0.25	-0.02	0.025	874	-0.14	-0.26	-0.02	0.025
Free cholesterol in medium LDL (mmol/l)	896	-0.11	-0.22	-0.01	0.033	896	-0.15	-0.26	-0.04	0.010	874	-0.15	-0.26	-0.03	0.013
Triglycerides in medium LDL (mmol/l)	896	-0.10	-0.21	0.01	0.071	896	-0.14	-0.25	-0.02	0.021	874	-0.13	-0.25	-0.01	0.030
Concentration of small LDL particles (mol/l)	896	-0.10	-0.21	0.01	0.071	896	-0.15	-0.27	-0.04	0.011	874	-0.16	-0.28	-0.04	0.009
Total lipids in small LDL (mmol/l)	896	-0.10	-0.21	0.01	0.073	896	-0.14	-0.26	-0.03	0.014	874	-0.15	-0.27	-0.03	0.014
Phospholipids in small LDL (mmol/l)	896	-0.10	-0.21	0.00	0.050	896	-0.15	-0.26	-0.04	0.009	874	-0.16	-0.27	-0.04	0.007
Total cholesterol in small LDL (mmol/l)	896	-0.10	-0.20	0.01	0.082	896	-0.14	-0.26	-0.02	0.019	874	-0.14	-0.26	-0.02	0.020
Cholesterol esters in small LDL (mmol/l)	896	-0.09	-0.20	0.02	0.107	896	-0.14	-0.26	-0.02	0.024	874	-0.14	-0.26	-0.02	0.024
Free cholesterol in small LDL (mmol/l)	896	-0.11	-0.22	-0.01	0.036	896	-0.14	-0.25	-0.03	0.015	874	-0.14	-0.25	-0.02	0.019
Triglycerides in small LDL (mmol/l)	896	-0.07	-0.18	0.04	0.220	896	-0.13	-0.25	-0.01	0.031	874	-0.14	-0.26	-0.02	0.022
Concentration of very large HDL particles (mol/l)	896	-0.09	-0.19	0.01	0.073	896	-0.03	-0.14	0.08	0.575	874	-0.01	-0.11	0.10	0.922
Total lipids in very large HDL (mmol/l)	896	-0.09	-0.19	0.01	0.092	896	-0.03	-0.14	0.08	0.610	874	0.00	-0.11	0.11	0.952
Phospholipids in very large HDL (mmol/l)	896	-0.10	-0.20	0.01	0.063	896	-0.03	-0.14	0.08	0.593	874	0.00	-0.11	0.10	0.954
Total cholesterol in very large HDL (mmol/l)	896	-0.08	-0.18	0.03	0.143	896	-0.03	-0.14	0.08	0.612	874	-0.01	-0.12	0.11	0.922
Cholesterol esters in very large HDL (mmol/l)	896	-0.08	-0.18	0.03	0.158	896	-0.03	-0.14	0.08	0.597	874	-0.01	-0.12	0.11	0.896
Free cholesterol in very large HDL (mmol/l)	896	-0.08	-0.18	0.02	0.126	896	-0.02	-0.13	0.09	0.666	874	0.00	-0.11	0.11	0.992
Triglycerides in very large HDL (mmol/l)	896	0.00	-0.10	0.11	0.957	896	0.03	-0.08	0.14	0.578	874	0.03	-0.08	0.14	0.573
Concentration of large HDL particles (mol/l)	896	-0.12	-0.23	-0.02	0.016	896	-0.04	-0.15	0.06	0.415	874	-0.02	-0.13	0.08	0.684
Total lipids in large HDL (mmol/l)	896	-0.12	-0.22	-0.02	0.023	896	-0.04	-0.15	0.07	0.482	874	-0.02	-0.12	0.09	0.774
Phospholipids in large HDL (mmol/l)	896	-0.13	-0.23	-0.03	0.013	896	-0.06	-0.16	0.05	0.300	874	-0.04	-0.14	0.07	0.495
Total cholesterol in large HDL (mmol/l)	896	-0.11	-0.21	-0.01	0.035	896	-0.03	-0.13	0.08	0.652	874	0.00	-0.11	0.11	0.981
Cholesterol esters in large HDL (mmol/l)	896	-0.11	-0.21	-0.01	0.036	896	-0.02	-0.13	0.09	0.668	874	0.00	-0.10	0.11	0.964
Free cholesterol in large HDL (mmol/l)	896	-0.11	-0.21	-0.01	0.033	896	-0.03	-0.14	0.08	0.596	874	0.00	-0.11	0.10	0.955
Triglycerides in large HDL (mmol/l)	896	-0.05	-0.15	0.05	0.341	896	0.00	-0.10	0.11	0.928	874	0.00	-0.11	0.10	0.970
Concentration of medium HDL particles (mol/l)	896	-0.16	-0.25	-0.06	1.06E-03	896	-0.12	-0.22	-0.02	0.016	874	-0.14	-0.24	-0.03	0.010
Total lipids in medium HDL (mmol/l)	896	-0.16	-0.25	-0.06	1.29E-03	896	-0.12	-0.22	-0.02	0.021	874	-0.13	-0.23	-0.03	0.014
Phospholipids in medium HDL (mmol/l)	896	-0.17	-0.26	-0.07	5.35E-04	896	-0.13	-0.23	-0.03	0.011	874	-0.13	-0.24	-0.03	0.010
Total cholesterol in medium HDL (mmol/l)	896	-0.14	-0.24	-0.04	0.008	896	-0.09	-0.20	0.02	0.093	874	-0.10	-0.21	0.01	0.069
Cholesterol esters in medium HDL (mmol/l)	896	-0.13	-0.24	-0.03	0.010	896	-0.09	-0.20	0.02	0.110	874	-0.10	-0.21	0.01	0.083
Free cholesterol in medium HDL (mmol/l)	896	-0.14	-0.23	-0.04	0.004	896	-0.10	-0.20	0.00	0.051	874	-0.11	-0.21	-0.01	0.037
Triglycerides in medium HDL (mmol/l)	896	-0.03	-0.13	0.07	0.557	896	-0.08	-0.19	0.03	0.155	874	-0.12	-0.22	-0.01	0.033
Concentration of small HDL particles (mol/l)	896	-0.10	-0.19	0.00	0.040	896	-0.14	-0.24	-0.04	0.007	874	-0.17	-0.27	-0.07	1.08E-03
Total lipids in small HDL (mmol/l)	896	-0.16	-0.25	-0.07	6.26E-04	896	-0.18	-0.28	-0.08	4.11E-04	874	-0.19	-0.29	-0.09	1.36E-04
Phospholipids in small HDL (mmol/l)	896	-0.05	-0.15	0.05	0.317	896	-0.08	-0.19	0.02	0.121	874	-0.12	-0.22	-0.01	0.030

S7 Table Associations of longer-term sedentary time (mean of SED measures at age 12y, 14y, and 15y) with metabolic traits at age 15y in ALSPAC

Mean of SED at age 12y, 14y, 15y (per SD (50 min/day) higher)

	Adj. for age, sex, ethnicity, maternal education, smoking, alcohol, mean wear time, wear month					*Additionally adj. for mean MVPA*					*Additionally adj. for mean FMI*				
**Standardised outcome at age 15y**	**N**	**Beta**	**LCL**	**UCL**	**P-value**	**N**	**Beta**	**LCL**	**UCL**	**P-value**	**N**	**Beta**	**LCL**	**UCL**	**P-value**
Total cholesterol in small HDL (mmol/l)	896	-0.23	-0.32	-0.14	7.82E-07	896	-0.21	-0.31	-0.12	1.56E-05	874	-0.20	-0.30	-0.11	4.69E-05
Cholesterol esters in small HDL (mmol/l)	896	-0.23	-0.32	-0.14	9.44E-07	896	-0.21	-0.31	-0.12	1.75E-05	874	-0.20	-0.30	-0.10	6.67E-05
Free cholesterol in small HDL (mmol/l)	896	-0.13	-0.22	-0.03	0.010	896	-0.11	-0.22	-0.01	0.033	874	-0.13	-0.23	-0.02	0.019
Triglycerides in small HDL (mmol/l)	896	0.00	-0.10	0.11	0.959	896	-0.07	-0.18	0.05	0.244	874	-0.09	-0.20	0.02	0.105
Phospholipids to total lipids ratio in chylomicrons and extremely large VLDL (%)	896	0.02	-0.08	0.12	0.684	896	-0.02	-0.13	0.08	0.681	874	-0.04	-0.15	0.06	0.424
Total cholesterol to total lipids ratio in chylomicrons and extremely large VLDL (%)	896	-0.09	-0.19	0.01	0.087	896	-0.11	-0.22	0.00	0.052	874	-0.13	-0.24	-0.03	0.015
Cholesterol esters to total lipids ratio in chylomicrons and extremely large VLDL (%)	896	-0.12	-0.23	-0.02	0.017	896	-0.13	-0.24	-0.02	0.020	874	-0.15	-0.25	-0.04	0.009
Free cholesterol to total lipids ratio in chylomicrons and extremely large VLDL (%)	896	0.05	-0.06	0.15	0.392	896	0.00	-0.11	0.11	0.986	874	-0.04	-0.16	0.07	0.454
Triglycerides to total lipids ratio in chylomicrons and extremely large VLDL (%)	896	0.06	-0.01	0.13	0.110	896	0.08	0.00	0.15	0.042	874	0.10	0.03	0.18	0.008
Phospholipids to total lipds ratio in very large VLDL (%)	896	-0.01	-0.11	0.10	0.904	896	-0.08	-0.19	0.04	0.196	874	-0.12	-0.23	0.00	0.047
Total cholesterol to total lipids ratio in very large VLDL (%)	896	-0.13	-0.23	-0.03	0.014	896	-0.08	-0.20	0.03	0.142	874	-0.08	-0.20	0.04	0.193
Cholesterol esters to total lipids ratio in very large VLDL (%)	896	-0.12	-0.22	-0.01	0.034	896	-0.06	-0.18	0.06	0.310	874	-0.05	-0.17	0.08	0.459
Free cholesterol to total lipids ratio in very large VLDL (%)	896	-0.12	-0.23	-0.01	0.030	896	-0.10	-0.22	0.02	0.116	874	-0.09	-0.22	0.03	0.154
Triglycerides to total lipids ratio in very large VLDL (%)	896	0.14	0.03	0.25	0.015	896	0.11	-0.01	0.23	0.071	874	0.11	-0.01	0.24	0.076
Phospholipids to total lipds ratio in large VLDL (%)	896	0.05	-0.06	0.16	0.369	896	-0.01	-0.13	0.11	0.899	874	-0.03	-0.16	0.09	0.594
Total cholesterol to total lipids ratio in large VLDL (%)	896	-0.03	-0.14	0.07	0.517	896	-0.07	-0.18	0.05	0.257	874	-0.09	-0.21	0.03	0.130
Cholesterol esters to total lipids ratio in large VLDL (%)	896	-0.07	-0.15	0.02	0.117	896	-0.06	-0.14	0.02	0.154	874	-0.08	-0.18	0.03	0.157
Free cholesterol to total lipids ratio in large VLDL (%)	896	0.05	-0.06	0.16	0.400	896	-0.02	-0.14	0.11	0.792	874	-0.06	-0.18	0.07	0.367
Triglycerides to total lipids ratio in large VLDL (%)	896	-0.03	-0.11	0.05	0.444	896	-0.02	-0.10	0.06	0.595	874	-0.04	-0.14	0.07	0.504
Phospholipids to total lipds ratio in medium VLDL (%)	896	-0.13	-0.24	-0.02	0.026	896	-0.09	-0.21	0.03	0.151	874	-0.07	-0.19	0.06	0.294
Total cholesterol to total lipids ratio in medium VLDL (%)	896	-0.10	-0.20	0.00	0.061	896	-0.08	-0.19	0.03	0.141	874	-0.08	-0.19	0.03	0.155
Cholesterol esters to total lipids ratio in medium VLDL (%)	896	-0.10	-0.21	0.00	0.057	896	-0.07	-0.19	0.04	0.209	874	-0.07	-0.18	0.05	0.264
Free cholesterol to total lipids ratio in medium VLDL (%)	896	-0.03	-0.13	0.07	0.559	896	-0.07	-0.18	0.04	0.221	874	-0.09	-0.20	0.02	0.125
Triglycerides to total lipids ratio in medium VLDL (%)	896	0.12	0.01	0.22	0.026	896	0.09	-0.02	0.20	0.093	874	0.09	-0.02	0.20	0.122
Phospholipids to total lipds ratio in small VLDL (%)	896	-0.12	-0.23	-0.01	0.040	896	-0.04	-0.16	0.08	0.512	874	-0.01	-0.14	0.11	0.843
Total cholesterol to total lipids ratio in small VLDL (%)	896	-0.08	-0.19	0.03	0.160	896	-0.07	-0.18	0.05	0.271	874	-0.05	-0.17	0.07	0.422
Cholesterol esters to total lipids ratio in small VLDL (%)	896	-0.05	-0.16	0.06	0.341	896	-0.05	-0.17	0.07	0.383	874	-0.04	-0.16	0.08	0.518
Free cholesterol to total lipids ratio in small VLDL (%)	896	-0.19	-0.31	-0.07	1.65E-03	896	-0.11	-0.24	0.01	0.075	874	-0.08	-0.21	0.04	0.199
Triglycerides to total lipids ratio in small VLDL (%)	896	0.11	0.00	0.22	0.046	896	0.08	-0.04	0.19	0.205	874	0.05	-0.07	0.17	0.401
Phospholipids to total lipds ratio in very small VLDL (%)	896	-0.10	-0.20	0.00	0.046	896	-0.10	-0.21	0.00	0.055	874	-0.09	-0.20	0.01	0.085
Total cholesterol to total lipids ratio in very small VLDL (%)	896	0.03	-0.07	0.14	0.533	896	0.07	-0.04	0.19	0.207	874	0.08	-0.04	0.20	0.175
Cholesterol esters to total lipids ratio in very small VLDL (%)	896	0.08	-0.02	0.17	0.114	896	0.08	-0.03	0.18	0.139	874	0.07	-0.03	0.18	0.166
Free cholesterol to total lipids ratio in very small VLDL (%)	896	-0.08	-0.20	0.04	0.206	896	0.02	-0.11	0.16	0.729	874	0.05	-0.09	0.18	0.473
Triglycerides to total lipids ratio in very small VLDL (%)	896	0.03	-0.07	0.14	0.561	896	-0.01	-0.13	0.10	0.812	874	-0.03	-0.15	0.09	0.629
Phospholipids to total lipds ratio in IDL (%)	896	-0.03	-0.16	0.10	0.627	896	-0.02	-0.16	0.12	0.761	874	-0.01	-0.15	0.13	0.896
Total cholesterol to total lipids ratio in IDL (%)	896	0.01	-0.09	0.12	0.794	896	0.01	-0.11	0.13	0.828	874	0.00	-0.12	0.12	0.991
Cholesterol esters to total lipids ratio in IDL (%)	896	0.04	-0.07	0.15	0.484	896	0.02	-0.10	0.15	0.741	874	0.00	-0.13	0.12	0.944
Free cholesterol to total lipids ratio in IDL (%)	896	-0.06	-0.16	0.04	0.232	896	-0.02	-0.12	0.09	0.740	874	0.01	-0.09	0.11	0.819
Triglycerides to total lipids ratio in IDL (%)	896	0.00	-0.10	0.10	0.961	896	-0.01	-0.12	0.11	0.914	874	0.00	-0.11	0.12	0.952
Phospholipids to total lipds ratio in large LDL (%)	896	0.03	-0.06	0.12	0.464	896	0.04	-0.06	0.14	0.392	874	0.03	-0.07	0.14	0.503
Total cholesterol to total lipids ratio in large LDL (%)	896	-0.03	-0.12	0.07	0.608	896	-0.04	-0.15	0.07	0.495	874	-0.04	-0.15	0.07	0.473
Cholesterol esters to total lipids ratio in large LDL (%)	896	-0.03	-0.12	0.07	0.592	896	-0.06	-0.16	0.05	0.319	874	-0.07	-0.18	0.05	0.243
Free cholesterol to total lipids ratio in large LDL (%)	896	0.02	-0.07	0.10	0.741	896	0.07	-0.03	0.16	0.159	874	0.09	0.00	0.19	0.054
Triglycerides to total lipids ratio in large LDL (%)	896	0.00	-0.10	0.11	0.953	896	0.01	-0.10	0.13	0.814	874	0.03	-0.09	0.15	0.630
Phospholipids to total lipds ratio in medium LDL (%)	896	0.01	-0.02	0.05	0.490	896	0.02	-0.02	0.06	0.380	874	0.02	-0.03	0.06	0.470
Total cholesterol to total lipids ratio in medium LDL (%)	896	-0.01	-0.11	0.09	0.820	896	-0.03	-0.14	0.09	0.657	874	-0.03	-0.15	0.09	0.665
Cholesterol esters to total lipids ratio in medium LDL (%)	896	-0.02	-0.13	0.08	0.665	896	-0.05	-0.16	0.07	0.422	874	-0.05	-0.17	0.07	0.401
Free cholesterol to total lipids ratio in medium LDL (%)	896	0.01	-0.02	0.04	0.508	896	0.02	-0.01	0.05	0.224	874	0.02	-0.01	0.05	0.183

S7 Table Associations of longer-term sedentary time (mean of SED measures at age 12y, 14y, and 15y) with metabolic traits at age 15y in ALSPAC

	**Mean of SED at age 12y, 14y, 15y (per SD (50 min/day) higher)**														
	Adj. for age, sex, ethnicity, maternal education, smoking, alcohol, mean wear time, wear month					*Additionally adj. for mean MVPA*					*Additionally adj. for mean FMI*				
**Standardised outcome at age 15y**	**N**	**Beta**	**LCL**	**UCL**	**P-value**	**N**	**Beta**	**LCL**	**UCL**	**P-value**	**N**	**Beta**	**LCL**	**UCL**	**P-value**
Triglycerides to total lipids ratio in medium LDL (%)	896	-0.05	-0.15	0.05	0.352	896	-0.05	-0.16	0.07	0.429	874	-0.03	-0.15	0.09	0.620
Phospholipids to total lipds ratio in small LDL (%)	896	0.02	-0.04	0.08	0.455	896	0.04	-0.03	0.10	0.254	874	0.04	-0.03	0.10	0.298
Total cholesterol to total lipids ratio in small LDL (%)	896	-0.03	-0.13	0.07	0.546	896	-0.04	-0.16	0.07	0.446	874	-0.04	-0.16	0.08	0.533
Cholesterol esters to total lipids ratio in small LDL (%)	896	-0.04	-0.14	0.07	0.503	896	-0.06	-0.18	0.06	0.303	874	-0.06	-0.18	0.06	0.317
Free cholesterol to total lipids ratio in small LDL (%)	896	0.02	-0.04	0.08	0.513	896	0.04	-0.02	0.10	0.199	874	0.05	-0.02	0.11	0.146
Triglycerides to total lipids ratio in small LDL (%)	896	-0.01	-0.12	0.09	0.773	896	-0.05	-0.16	0.06	0.368	874	-0.06	-0.17	0.05	0.294
Phospholipids to total lipds ratio in very large HDL (%)	896	-0.09	-0.18	0.01	0.091	896	-0.02	-0.13	0.08	0.686	874	0.01	-0.09	0.10	0.913
Total cholesterol to total lipids ratio in very large HDL (%)	896	0.07	-0.02	0.17	0.127	896	0.01	-0.09	0.12	0.779	874	-0.01	-0.10	0.09	0.866
Cholesterol esters to total lipids ratio in very large HDL (%)	896	0.07	-0.03	0.16	0.160	896	0.01	-0.09	0.11	0.847	874	-0.01	-0.11	0.08	0.799
Free cholesterol to total lipids ratio in very large HDL (%)	896	0.03	-0.08	0.14	0.577	896	0.04	-0.07	0.16	0.474	874	0.05	-0.07	0.17	0.430
Triglycerides to total lipids ratio in very large HDL (%)	896	0.07	-0.04	0.19	0.197	896	0.04	-0.08	0.16	0.488	874	0.01	-0.10	0.13	0.813
Phospholipids to total lipds ratio in large HDL (%)	896	-0.02	-0.13	0.09	0.718	896	-0.11	-0.23	0.01	0.067	874	-0.15	-0.26	-0.03	0.012
Total cholesterol to total lipids ratio in large HDL (%)	896	-0.02	-0.13	0.09	0.746	896	0.07	-0.05	0.19	0.275	874	0.11	-0.01	0.22	0.070
Cholesterol esters to total lipids ratio in large HDL (%)	896	-0.02	-0.13	0.10	0.752	896	0.07	-0.05	0.19	0.267	874	0.11	-0.01	0.22	0.070
Free cholesterol to total lipids ratio in large HDL (%)	896	-0.01	-0.12	0.09	0.786	896	0.05	-0.07	0.16	0.418	874	0.08	-0.03	0.19	0.160
Triglycerides to total lipids ratio in large HDL (%)	896	0.09	-0.02	0.21	0.114	896	0.04	-0.08	0.16	0.490	874	0.01	-0.11	0.12	0.925
Phospholipids to total lipds ratio in medium HDL (%)	896	-0.14	-0.25	-0.03	0.010	896	-0.11	-0.23	0.00	0.060	874	-0.09	-0.21	0.03	0.141
Total cholesterol to total lipids ratio in medium HDL (%)	896	0.09	-0.02	0.20	0.095	896	0.11	-0.01	0.23	0.063	874	0.11	0.00	0.23	0.058
Cholesterol esters to total lipids ratio in medium HDL (%)	896	0.08	-0.03	0.20	0.153	896	0.10	-0.02	0.22	0.100	874	0.10	-0.02	0.22	0.093
Free cholesterol to total lipids ratio in medium HDL (%)	896	0.08	-0.08	0.23	0.345	896	0.08	-0.08	0.24	0.324	874	0.08	-0.08	0.24	0.336
Triglycerides to total lipids ratio in medium HDL (%)	896	0.04	-0.08	0.15	0.533	896	-0.03	-0.15	0.08	0.572	874	-0.07	-0.19	0.05	0.240
Phospholipids to total lipds ratio in small HDL (%)	896	0.20	0.11	0.30	3.74E-05	896	0.17	0.07	0.28	1.09E-03	874	0.15	0.04	0.25	0.006
Total cholesterol to total lipids ratio in small HDL (%)	896	-0.21	-0.30	-0.11	3.19E-05	896	-0.16	-0.27	-0.06	2.44E-03	874	-0.13	-0.24	-0.03	0.014
Cholesterol esters to total lipids ratio in small HDL (%)	896	-0.20	-0.30	-0.11	4.31E-05	896	-0.17	-0.28	-0.07	1.41E-03	874	-0.14	-0.25	-0.04	0.007
Free cholesterol to total lipids ratio in small HDL (%)	896	0.06	-0.05	0.17	0.317	896	0.13	0.01	0.26	0.031	874	0.14	0.02	0.26	0.019
Triglycerides to total lipids ratio in small HDL (%)	896	0.08	-0.03	0.19	0.139	896	0.01	-0.10	0.13	0.851	874	-0.01	-0.13	0.10	0.859
Mean diameter for VLDL particles (nm)	896	0.10	-0.01	0.21	0.063	896	0.03	-0.09	0.14	0.653	874	-0.02	-0.13	0.09	0.699
Mean diameter for LDL particles (nm)	896	0.06	-0.04	0.16	0.225	896	0.12	0.01	0.23	0.027	874	0.14	0.04	0.25	0.008
Mean diameter for HDL particles (nm)	896	-0.08	-0.18	0.02	0.112	896	0.00	-0.11	0.11	0.995	874	0.03	-0.08	0.14	0.581
Serum total cholesterol (mmol/l)	896	-0.11	-0.21	-0.01	0.028	896	-0.13	-0.24	-0.02	0.016	874	-0.13	-0.24	-0.02	0.018
Total cholesterol in VLDL (mmol/l)	896	0.02	-0.09	0.12	0.772	896	-0.05	-0.16	0.06	0.388	874	-0.08	-0.19	0.03	0.155
Remnant cholesterol (non-HDL, non-LDL -cholesterol) (mmol/l)	896	-0.03	-0.14	0.08	0.562	896	-0.09	-0.20	0.03	0.144	874	-0.10	-0.22	0.01	0.078
Total cholesterol in LDL (mmol/l)	896	-0.10	-0.20	0.01	0.075	896	-0.14	-0.25	-0.02	0.020	874	-0.14	-0.25	-0.02	0.022
Total cholesterol in HDL (mmol/l)	896	-0.15	-0.25	-0.05	0.003	896	-0.08	-0.19	0.02	0.119	874	-0.07	-0.17	0.04	0.230
Total cholesterol in HDL2 (mmol/l)	896	-0.15	-0.25	-0.05	0.005	896	-0.08	-0.19	0.03	0.152	874	-0.06	-0.16	0.05	0.308
Total cholesterol in HDL3 (mmol/l)	896	-0.15	-0.24	-0.05	2.95E-03	896	-0.09	-0.19	0.01	0.086	874	-0.08	-0.18	0.03	0.140
Esterified cholesterol (mmol/l)	896	-0.11	-0.21	-0.01	0.029	896	-0.13	-0.24	-0.02	0.019	874	-0.13	-0.24	-0.02	0.020
Free cholesterol (mmol/l)	896	-0.11	-0.21	-0.01	0.031	896	-0.13	-0.24	-0.03	0.015	874	-0.13	-0.25	-0.02	0.018
Serum total triglycerides (mmol/l)	896	0.05	-0.06	0.16	0.351	896	-0.03	-0.14	0.09	0.650	874	-0.06	-0.17	0.05	0.254
Triglycerides in VLDL (mmol/l)	896	0.08	-0.03	0.19	0.138	896	0.00	-0.11	0.11	0.963	874	-0.04	-0.15	0.07	0.458
Triglycerides in LDL (mmol/l)	896	-0.09	-0.19	0.02	0.099	896	-0.13	-0.24	-0.01	0.028	874	-0.12	-0.24	-0.01	0.036
Triglycerides in HDL (mmol/l)	896	-0.02	-0.12	0.08	0.708	896	-0.05	-0.16	0.06	0.361	874	-0.08	-0.19	0.03	0.164
Diacylglycerol (mmol/l)	864	0.05	-0.06	0.15	0.384	864	0.03	-0.08	0.14	0.589	843	0.01	-0.10	0.12	0.896
Ratio of diacylglycerol to triglycerides	864	0.02	-0.09	0.13	0.710	864	0.03	-0.09	0.15	0.602	843	0.03	-0.10	0.15	0.643
Total phosphoglycerides (mmol/l)	896	-0.14	-0.24	-0.04	0.005	896	-0.12	-0.23	-0.02	0.020	874	-0.13	-0.24	-0.02	0.017
Ratio of triglycerides to phosphoglycerides	896	0.12	0.02	0.22	0.023	896	0.05	-0.06	0.16	0.370	874	0.01	-0.10	0.11	0.903
Phosphatidylcholine and other cholines (mmol/l)	877	-0.12	-0.21	-0.02	0.018	877	-0.09	-0.19	0.01	0.087	855	-0.10	-0.20	0.00	0.062
Total cholines (mmol/l)	893	-0.15	-0.24	-0.05	2.63E-03	893	-0.13	-0.23	-0.03	0.011	871	-0.14	-0.24	-0.03	0.009

S7 Table Associations of longer-term sedentary time (mean of SED measures at age 12y, 14y, and 15y) with metabolic traits at age 15y in ALSPAC

Mean of SED at age 12y, 14y, 15y (per SD (50 min/day) higher)

	Adj. for age, sex, ethnicity, maternal education, smoking, alcohol, mean wear time, wear month					*Additionally adj. for mean MVPA*					*Additionally adj. for mean FMI*				
**Standardised outcome at age 15y**	**N**	**Beta**	**LCL**	**UCL**	**P-value**	**N**	**Beta**	**LCL**	**UCL**	**P-value**	**N**	**Beta**	**LCL**	**UCL**	**P-value**
Apolipoprotein A-I (g/l)	896	-0.15	-0.24	-0.05	2.60E-03	896	-0.11	-0.21	-0.01	0.036	874	-0.10	-0.20	0.00	0.054
Apolipoprotein B (g/l)	896	-0.02	-0.13	0.09	0.756	896	-0.09	-0.20	0.03	0.139	874	-0.11	-0.23	0.01	0.063
Ratio of apolipoprotein B to apolipoprotein A-I	896	0.05	-0.06	0.16	0.356	896	-0.04	-0.16	0.08	0.555	874	-0.06	-0.18	0.06	0.295
Total fatty acids (mmol/l)	896	-0.07	-0.17	0.02	0.139	896	-0.10	-0.20	0.00	0.054	874	-0.12	-0.23	-0.02	0.022
Estimated description of fatty acid chain length, not actual carbon number	892	0.10	0.00	0.21	0.060	892	0.11	-0.01	0.22	0.067	870	0.10	-0.02	0.21	0.113
Estimated degree of unsaturation	895	0.05	-0.05	0.16	0.342	895	0.07	-0.05	0.18	0.252	873	0.07	-0.05	0.19	0.250
22:6, docosahexaenoic acid (mmol/l)	896	-0.05	-0.16	0.06	0.377	896	-0.04	-0.16	0.07	0.438	874	-0.06	-0.17	0.05	0.275
18:2, linoleic acid (mmol/l)	893	-0.09	-0.19	0.00	0.057	893	-0.12	-0.22	-0.01	0.028	871	-0.13	-0.23	-0.02	0.023
Conjugated linoleic acid (mmol/l)	896	0.10	-0.02	0.21	0.112	896	0.08	-0.05	0.20	0.250	874	0.06	-0.07	0.19	0.372
Omega-3 fatty acids (mmol/l)	894	-0.03	-0.14	0.08	0.566	894	-0.05	-0.16	0.07	0.410	872	-0.06	-0.18	0.05	0.294
Omega-6 fatty acids (mmol/l)	895	-0.08	-0.18	0.01	0.082	895	-0.11	-0.21	0.00	0.045	873	-0.12	-0.22	-0.01	0.036
Polyunsaturated fatty acids (mmol/l)	893	-0.08	-0.18	0.01	0.084	893	-0.10	-0.21	0.00	0.051	871	-0.11	-0.22	-0.01	0.039
Monounsaturated fatty acids; 16:1, 18:1 (mmol/l)	893	-0.03	-0.13	0.07	0.602	893	-0.08	-0.18	0.03	0.145	871	-0.10	-0.21	0.00	0.055
Saturated fatty acids (mmol/l)	892	-0.09	-0.19	0.00	0.058	892	-0.10	-0.20	0.01	0.063	870	-0.12	-0.23	-0.01	0.027
Ratio of 22:6 docosahexaenoic acid to total fatty acids (%)	896	-0.01	-0.12	0.10	0.833	896	0.01	-0.10	0.13	0.842	874	0.00	-0.12	0.12	0.979
Ratio of 18:2 linoleic acid to total fatty acids (%)	893	-0.02	-0.12	0.09	0.759	893	-0.01	-0.13	0.11	0.866	871	0.01	-0.11	0.13	0.859
Ratio of conjugated linoleic acid to total fatty acids (%)	896	0.11	-0.01	0.24	0.073	896	0.09	-0.04	0.22	0.180	874	0.08	-0.06	0.22	0.257
Ratio of omega-3 fatty acids to total fatty acids (%)	894	0.02	-0.09	0.13	0.746	894	0.02	-0.10	0.15	0.695	872	0.02	-0.10	0.15	0.701
Ratio of omega-6 fatty acids to total fatty acids (%)	895	0.00	-0.10	0.11	0.951	895	0.02	-0.09	0.14	0.676	873	0.05	-0.06	0.17	0.387
Ratio of polyunsaturated fatty acids to total fatty acids (%)	893	0.00	-0.10	0.10	0.991	893	0.02	-0.09	0.14	0.703	871	0.05	-0.07	0.16	0.412
Ratio of monounsaturated fatty acids to total fatty acids (%)	893	0.04	-0.06	0.15	0.400	893	0.00	-0.12	0.11	0.961	871	-0.02	-0.13	0.09	0.732
Ratio of saturated fatty acids to total fatty acids (%)	892	-0.07	-0.17	0.04	0.203	892	-0.03	-0.14	0.08	0.587	870	-0.04	-0.16	0.08	0.497
Insulin (mu/l)	927	0.06	-0.01	0.12	0.075	927	-0.01	-0.07	0.06	0.882	904	-0.03	-0.10	0.04	0.350
Glucose (mmol/l)	894	0.04	-0.06	0.14	0.435	894	0.01	-0.10	0.12	0.813	872	0.01	-0.10	0.12	0.890
Lactate (mmol/l)	894	0.05	-0.06	0.16	0.360	894	0.03	-0.09	0.15	0.627	872	0.03	-0.09	0.15	0.618
Pyruvate (mmol/l)	893	0.15	0.05	0.25	2.81E-03	893	0.12	0.01	0.23	0.031	871	0.10	-0.01	0.21	0.064
Citrate (mmol/l)	891	-0.15	-0.27	-0.04	0.009	891	-0.12	-0.24	0.00	0.044	869	-0.10	-0.22	0.02	0.107
Alanine (mmol/l)	896	0.17	0.06	0.27	1.83E-03	896	0.15	0.04	0.27	0.008	874	0.15	0.03	0.27	0.011
Glutamine (mmol/l)	896	0.00	-0.10	0.09	0.920	896	0.01	-0.10	0.11	0.923	874	0.03	-0.07	0.14	0.535
Histidine (mmol/l)	850	0.03	-0.07	0.13	0.528	850	0.09	-0.02	0.20	0.099	828	0.10	-0.01	0.21	0.080
Isoleucine (mmol/l)	896	0.08	-0.01	0.18	0.081	896	0.07	-0.03	0.17	0.176	874	0.06	-0.05	0.16	0.274
Leucine (mmol/l)	896	0.00	-0.08	0.09	0.953	896	0.03	-0.06	0.12	0.511	874	0.04	-0.06	0.13	0.428
Valine (mmol/l)	896	0.00	-0.09	0.09	0.956	896	0.00	-0.11	0.10	0.950	874	-0.01	-0.11	0.10	0.881
Phenylalanine (mmol/l)	895	-0.01	-0.12	0.10	0.844	895	0.04	-0.07	0.15	0.480	873	0.04	-0.08	0.15	0.537
Tyrosine (mmol/l)	892	-0.02	-0.13	0.09	0.736	892	0.00	-0.12	0.11	0.937	870	-0.02	-0.14	0.09	0.692
Acetate (mmol/l)	895	-0.05	-0.16	0.05	0.299	895	-0.01	-0.13	0.10	0.830	873	0.01	-0.11	0.12	0.919
Acetoacetate (mmol/l)	896	0.04	-0.07	0.14	0.481	896	0.02	-0.10	0.13	0.756	874	0.02	-0.10	0.14	0.693
3-hydroxybutyrate (mmol/l)	895	-0.04	-0.15	0.08	0.504	895	-0.09	-0.22	0.04	0.156	873	-0.09	-0.22	0.05	0.203
Creatinine (mmol/l)	895	0.14	0.04	0.24	0.005	895	0.15	0.04	0.25	0.008	873	0.15	0.04	0.26	0.009
Albumin (signal area)	896	0.13	0.02	0.24	0.016	896	0.08	-0.03	0.20	0.149	874	0.06	-0.05	0.18	0.282
Glycoprotein acetyls, mainly a1-acid glycoprotein (mmol/l)	895	0.10	-0.01	0.20	0.065	895	0.00	-0.10	0.11	0.956	873	-0.03	-0.14	0.07	0.546
C-reactive protein (mg/l)	929	0.07	-0.04	0.17	0.224	929	0.09	-0.04	0.21	0.165	906	0.09	-0.04	0.22	0.159

Mean of SED at age 12y, 14y, 15y (per SD (50 min/day) higher)

Complete case sample

Adj. for age, sex, ethnicity, maternal education, *Additionally adj. for mean MVPA* *Additionally adj. for mean FMI*

S7 Table Associations of longer-term sedentary time (mean of SED measures at age 12y, 14y, and 15y) with metabolic traits at age 15y in ALSPAC

Mean of SED at age 12y, 14y, 15y (per SD (50 min/day) higher)

	Adj. for age, sex, ethnicity, maternal education, smoking, alcohol, mean wear time, wear month					*Additionally adj. for mean MVPA*					*Additionally adj. for mean FMI*				
**Standardised outcome at age 15y**	**N**	**Beta**	**LCL**	**UCL**	**P-value**	**N**	**Beta**	**LCL**	**UCL**	**P-value**	**N**	**Beta**	**LCL**	**UCL**	**P-value**
Systolic blood pressure (mmHg)	755	0.05	-0.07	0.17	0.411	755	0.01	-0.12	0.13	0.911	755	-0.02	-0.14	0.11	0.777
Diastolic blood pressure (mmHg)	755	0.03	-0.08	0.14	0.580	755	0.04	-0.08	0.17	0.511	755	0.03	-0.09	0.16	0.597
Concentration of chylomicrons and extremely large VLDL particles (mol/l)	755	0.05	-0.06	0.17	0.368	755	-0.02	-0.14	0.10	0.769	755	-0.05	-0.17	0.06	0.370
Total lipids in chylomicrons and extremely large VLDL (mmol/l)	755	0.05	-0.06	0.17	0.380	755	-0.02	-0.14	0.10	0.786	755	-0.05	-0.17	0.07	0.394
Phospholipids in chylomicrons and extremely large VLDL (mmol/l)	755	0.05	-0.07	0.17	0.429	755	-0.02	-0.15	0.10	0.700	755	-0.06	-0.17	0.06	0.352
Total cholesterol in chylomicrons and extremely large VLDL (mmol/l)	755	0.05	-0.07	0.16	0.409	755	-0.01	-0.13	0.11	0.877	755	-0.04	-0.16	0.07	0.433
Cholesterol esters in chylomicrons and extremely large VLDL (mmol/l)	755	0.04	-0.07	0.16	0.454	755	0.00	-0.12	0.12	0.998	755	-0.04	-0.15	0.07	0.513
Free cholesterol in chylomicrons and extremely large VLDL (mmol/l)	755	0.05	-0.07	0.17	0.396	755	-0.02	-0.14	0.10	0.763	755	-0.05	-0.17	0.07	0.396
Triglycerides in chylomicrons and extremely large VLDL (mmol/l)	755	0.05	-0.06	0.17	0.369	755	-0.02	-0.14	0.10	0.783	755	-0.05	-0.17	0.07	0.397
Concentration of very large VLDL particles (mol/l)	755	0.06	-0.05	0.17	0.305	755	0.00	-0.12	0.12	0.972	755	-0.04	-0.15	0.08	0.533
Total lipids in very large VLDL (mmol/l)	755	0.06	-0.06	0.17	0.333	755	0.00	-0.12	0.11	0.952	755	-0.04	-0.15	0.08	0.523
Phospholipids in very large VLDL (mmol/l)	755	0.05	-0.06	0.17	0.382	755	-0.01	-0.13	0.11	0.855	755	-0.04	-0.16	0.07	0.464
Total cholesterol in very large VLDL (mmol/l)	755	0.05	-0.06	0.17	0.364	755	-0.01	-0.13	0.11	0.890	755	-0.04	-0.16	0.07	0.439
Cholesterol esters in very large VLDL (mmol/l)	755	0.06	-0.06	0.17	0.317	755	0.00	-0.12	0.12	0.983	755	-0.04	-0.15	0.07	0.512
Free cholesterol in very large VLDL (mmol/l)	755	0.05	-0.07	0.16	0.426	755	-0.02	-0.14	0.10	0.760	755	-0.05	-0.17	0.06	0.377
Triglycerides in very large VLDL (mmol/l)	755	0.06	-0.06	0.17	0.314	755	0.00	-0.12	0.12	0.998	755	-0.03	-0.14	0.08	0.571
Concentration of large VLDL particles (mol/l)	755	0.06	-0.06	0.17	0.315	755	0.00	-0.11	0.12	0.957	755	-0.03	-0.14	0.08	0.593
Total lipids in large VLDL (mmol/l)	755	0.06	-0.06	0.17	0.339	755	0.00	-0.11	0.12	0.979	755	-0.03	-0.14	0.08	0.573
Phospholipids in large VLDL (mmol/l)	755	0.05	-0.06	0.17	0.379	755	0.00	-0.12	0.11	0.945	755	-0.04	-0.15	0.07	0.515
Total cholesterol in large VLDL (mmol/l)	755	0.04	-0.07	0.16	0.463	755	-0.01	-0.12	0.11	0.888	755	-0.04	-0.15	0.07	0.445
Cholesterol esters in large VLDL (mmol/l)	755	0.04	-0.08	0.15	0.539	755	-0.01	-0.13	0.11	0.867	755	-0.05	-0.16	0.06	0.410
Free cholesterol in large VLDL (mmol/l)	755	0.05	-0.07	0.16	0.402	755	-0.01	-0.12	0.11	0.910	755	-0.04	-0.15	0.07	0.490
Triglycerides in large VLDL (mmol/l)	755	0.06	-0.05	0.18	0.290	755	0.01	-0.11	0.12	0.905	755	-0.03	-0.14	0.09	0.648
Concentration of medium VLDL particles (mol/l)	755	0.05	-0.06	0.17	0.354	755	0.00	-0.12	0.12	0.975	755	-0.04	-0.15	0.08	0.511
Total lipids in medium VLDL (mmol/l)	755	0.05	-0.07	0.16	0.403	755	0.00	-0.12	0.11	0.942	755	-0.04	-0.15	0.07	0.478
Phospholipids in medium VLDL (mmol/l)	755	0.04	-0.08	0.15	0.529	755	-0.02	-0.14	0.10	0.766	755	-0.05	-0.17	0.06	0.367
Total cholesterol in medium VLDL (mmol/l)	755	0.02	-0.10	0.14	0.737	755	-0.02	-0.14	0.10	0.706	755	-0.06	-0.17	0.06	0.316
Cholesterol esters in medium VLDL (mmol/l)	755	0.01	-0.11	0.12	0.912	755	-0.02	-0.14	0.10	0.700	755	-0.06	-0.18	0.06	0.313
Free cholesterol in medium VLDL (mmol/l)	755	0.03	-0.08	0.15	0.559	755	-0.02	-0.14	0.10	0.736	755	-0.05	-0.17	0.06	0.361
Triglycerides in medium VLDL (mmol/l)	755	0.07	-0.05	0.18	0.260	755	0.01	-0.11	0.13	0.878	755	-0.03	-0.14	0.09	0.636
Concentration of small VLDL particles (mol/l)	755	0.01	-0.11	0.13	0.875	755	-0.05	-0.17	0.07	0.399	755	-0.09	-0.20	0.03	0.145
Total lipids in small VLDL (mmol/l)	755	0.00	-0.12	0.11	0.947	755	-0.07	-0.19	0.05	0.281	755	-0.10	-0.22	0.01	0.086
Phospholipids in small VLDL (mmol/l)	755	-0.02	-0.14	0.09	0.669	755	-0.08	-0.20	0.04	0.183	755	-0.12	-0.23	0.00	0.047
Total cholesterol in small VLDL (mmol/l)	755	-0.04	-0.15	0.08	0.556	755	-0.09	-0.22	0.03	0.131	755	-0.13	-0.25	-0.01	0.034
Cholesterol esters in small VLDL (mmol/l)	755	-0.03	-0.15	0.08	0.566	755	-0.09	-0.22	0.03	0.140	755	-0.13	-0.26	-0.01	0.039
Free cholesterol in small VLDL (mmol/l)	755	-0.03	-0.15	0.08	0.565	755	-0.09	-0.21	0.03	0.148	755	-0.12	-0.24	0.00	0.044
Triglycerides in small VLDL (mmol/l)	755	0.03	-0.08	0.15	0.592	755	-0.03	-0.15	0.09	0.649	755	-0.06	-0.18	0.06	0.317
Concentration of very small VLDL particles (mol/l)	755	-0.07	-0.18	0.04	0.190	755	-0.11	-0.23	0.01	0.077	755	-0.12	-0.24	0.00	0.043
Total lipids in very small VLDL (mmol/l)	755	-0.06	-0.17	0.06	0.346	755	-0.10	-0.22	0.03	0.120	755	-0.12	-0.24	0.00	0.058
Phospholipids in very small VLDL (mmol/l)	755	-0.09	-0.21	0.02	0.100	755	-0.13	-0.25	-0.01	0.038	755	-0.14	-0.26	-0.01	0.028
Total cholesterol in very small VLDL (mmol/l)	755	-0.03	-0.14	0.09	0.646	755	-0.06	-0.18	0.07	0.382	755	-0.08	-0.21	0.04	0.196
Cholesterol esters in very small VLDL (mmol/l)	755	-0.01	-0.13	0.11	0.855	755	-0.05	-0.18	0.07	0.409	755	-0.09	-0.21	0.04	0.185
Free cholesterol in very small VLDL (mmol/l)	755	-0.06	-0.18	0.06	0.317	755	-0.06	-0.18	0.07	0.383	755	-0.07	-0.20	0.06	0.279
Triglycerides in very small VLDL (mmol/l)	755	-0.03	-0.14	0.08	0.576	755	-0.09	-0.21	0.03	0.134	755	-0.11	-0.23	0.01	0.070
Concentration of IDL particles (mol/l)	755	-0.11	-0.22	0.01	0.065	755	-0.14	-0.27	-0.02	0.023	755	-0.14	-0.27	-0.02	0.025

smoking, alcohol, mean wear time, wear month
Standardised outcome at age 15y N Beta LCL UCL P-value N Beta LCL UCL P-value N Beta LCL UCL P-value

S7 Table Associations of longer-term sedentary time (mean of SED measures at age 12y, 14y, and 15y) with metabolic traits at age 15y in ALSPAC

	**Mean of SED at age 12y, 14y, 15y (per SD (50 min/day) higher)**														
	Adj. for age, sex, ethnicity, maternal education, smoking, alcohol, mean wear time, wear month					*Additionally adj. for mean MVPA*					*Additionally adj. for mean FMI*				
**Standardised outcome at age 15y**	**N**	**Beta**	**LCL**	**UCL**	**P-value**	**N**	**Beta**	**LCL**	**UCL**	**P-value**	**N**	**Beta**	**LCL**	**UCL**	**P-value**
Total lipids in IDL (mmol/l)	755	-0.11	-0.22	0.01	0.068	755	-0.14	-0.26	-0.02	0.028	755	-0.14	-0.27	-0.02	0.026
Phospholipids in IDL (mmol/l)	755	-0.12	-0.24	0.00	0.044	755	-0.15	-0.28	-0.03	0.017	755	-0.15	-0.28	-0.02	0.019
Total cholesterol in IDL (mmol/l)	755	-0.10	-0.21	0.02	0.098	755	-0.13	-0.25	0.00	0.048	755	-0.13	-0.26	-0.01	0.039
Cholesterol esters in IDL (mmol/l)	755	-0.09	-0.21	0.03	0.136	755	-0.12	-0.24	0.01	0.064	755	-0.13	-0.26	0.00	0.045
Free cholesterol in IDL (mmol/l)	755	-0.12	-0.23	0.00	0.048	755	-0.14	-0.26	-0.02	0.028	755	-0.14	-0.26	-0.01	0.033
Triglycerides in IDL (mmol/l)	755	-0.09	-0.19	0.02	0.102	755	-0.13	-0.24	-0.01	0.032	755	-0.12	-0.23	0.00	0.045
Concentration of large LDL particles (mol/l)	755	-0.13	-0.25	-0.02	0.026	755	-0.17	-0.30	-0.05	0.007	755	-0.17	-0.30	-0.05	0.007
Total lipids in large LDL (mmol/l)	755	-0.12	-0.24	-0.01	0.037	755	-0.16	-0.28	-0.04	0.012	755	-0.16	-0.28	-0.04	0.011
Phospholipids in large LDL (mmol/l)	755	-0.12	-0.24	-0.01	0.034	755	-0.16	-0.29	-0.04	0.010	755	-0.17	-0.29	-0.04	0.008
Total cholesterol in large LDL (mmol/l)	755	-0.12	-0.23	0.00	0.043	755	-0.15	-0.28	-0.03	0.015	755	-0.16	-0.28	-0.03	0.014
Cholesterol esters in large LDL (mmol/l)	755	-0.12	-0.23	0.00	0.047	755	-0.15	-0.28	-0.03	0.015	755	-0.16	-0.28	-0.03	0.013
Free cholesterol in large LDL (mmol/l)	755	-0.12	-0.24	-0.01	0.034	755	-0.15	-0.28	-0.03	0.015	755	-0.15	-0.28	-0.03	0.017
Triglycerides in large LDL (mmol/l)	755	-0.10	-0.21	0.01	0.068	755	-0.14	-0.25	-0.02	0.022	755	-0.12	-0.24	-0.01	0.041
Concentration of medium LDL particles (mol/l)	755	-0.13	-0.25	-0.01	0.029	755	-0.18	-0.31	-0.05	0.005	755	-0.18	-0.31	-0.06	0.005
Total lipids in medium LDL (mmol/l)	755	-0.12	-0.24	-0.01	0.035	755	-0.17	-0.29	-0.04	0.008	755	-0.17	-0.30	-0.05	0.007
Phospholipids in medium LDL (mmol/l)	755	-0.12	-0.23	-0.01	0.031	755	-0.16	-0.28	-0.04	0.007	755	-0.17	-0.29	-0.05	0.004
Total cholesterol in medium LDL (mmol/l)	755	-0.12	-0.24	0.00	0.045	755	-0.16	-0.29	-0.04	0.012	755	-0.17	-0.29	-0.04	0.010
Cholesterol esters in medium LDL (mmol/l)	755	-0.12	-0.23	0.00	0.054	755	-0.16	-0.29	-0.03	0.014	755	-0.16	-0.29	-0.04	0.012
Free cholesterol in medium LDL (mmol/l)	755	-0.13	-0.25	-0.02	0.024	755	-0.17	-0.29	-0.04	0.008	755	-0.17	-0.29	-0.05	0.007
Triglycerides in medium LDL (mmol/l)	755	-0.12	-0.23	-0.01	0.038	755	-0.16	-0.29	-0.04	0.010	755	-0.15	-0.27	-0.02	0.019
Concentration of small LDL particles (mol/l)	755	-0.13	-0.25	-0.02	0.025	755	-0.18	-0.31	-0.06	0.005	755	-0.18	-0.31	-0.06	0.004
Total lipids in small LDL (mmol/l)	755	-0.13	-0.24	-0.01	0.034	755	-0.17	-0.29	-0.04	0.008	755	-0.17	-0.30	-0.05	0.007
Phospholipids in small LDL (mmol/l)	755	-0.13	-0.24	-0.02	0.024	755	-0.17	-0.28	-0.05	0.006	755	-0.17	-0.29	-0.05	0.005
Total cholesterol in small LDL (mmol/l)	755	-0.12	-0.24	0.00	0.042	755	-0.16	-0.29	-0.04	0.011	755	-0.17	-0.30	-0.04	0.010
Cholesterol esters in small LDL (mmol/l)	755	-0.12	-0.24	0.00	0.049	755	-0.16	-0.29	-0.04	0.012	755	-0.17	-0.30	-0.04	0.011
Free cholesterol in small LDL (mmol/l)	755	-0.12	-0.24	-0.01	0.037	755	-0.15	-0.27	-0.02	0.018	755	-0.15	-0.28	-0.03	0.014
Triglycerides in small LDL (mmol/l)	755	-0.10	-0.21	0.02	0.092	755	-0.16	-0.28	-0.03	0.015	755	-0.16	-0.28	-0.03	0.014
Concentration of very large HDL particles (mol/l)	755	-0.10	-0.22	0.01	0.075	755	-0.05	-0.17	0.07	0.379	755	-0.01	-0.12	0.11	0.876
Total lipids in very large HDL (mmol/l)	755	-0.10	-0.22	0.01	0.080	755	-0.05	-0.17	0.07	0.381	755	-0.01	-0.13	0.11	0.866
Phospholipids in very large HDL (mmol/l)	755	-0.10	-0.22	0.01	0.073	755	-0.05	-0.17	0.07	0.410	755	0.00	-0.12	0.11	0.951
Total cholesterol in very large HDL (mmol/l)	755	-0.10	-0.21	0.02	0.095	755	-0.06	-0.18	0.06	0.351	755	-0.02	-0.14	0.10	0.748
Cholesterol esters in very large HDL (mmol/l)	755	-0.10	-0.21	0.02	0.099	755	-0.06	-0.18	0.06	0.338	755	-0.02	-0.14	0.10	0.702
Free cholesterol in very large HDL (mmol/l)	755	-0.10	-0.21	0.02	0.095	755	-0.05	-0.17	0.07	0.401	755	-0.01	-0.13	0.11	0.877
Triglycerides in very large HDL (mmol/l)	755	-0.01	-0.12	0.10	0.860	755	0.01	-0.11	0.13	0.859	755	0.03	-0.08	0.15	0.565
Concentration of large HDL particles (mol/l)	755	-0.12	-0.23	-0.01	0.041	755	-0.05	-0.17	0.07	0.393	755	-0.01	-0.12	0.11	0.927
Total lipids in large HDL (mmol/l)	755	-0.11	-0.23	0.00	0.049	755	-0.05	-0.17	0.07	0.441	755	0.00	-0.12	0.12	0.999
Phospholipids in large HDL (mmol/l)	755	-0.13	-0.24	-0.01	0.027	755	-0.07	-0.18	0.05	0.270	755	-0.02	-0.13	0.09	0.710
Total cholesterol in large HDL (mmol/l)	755	-0.10	-0.22	0.01	0.078	755	-0.03	-0.15	0.09	0.608	755	0.02	-0.10	0.13	0.775
Cholesterol esters in large HDL (mmol/l)	755	-0.10	-0.22	0.01	0.080	755	-0.03	-0.15	0.09	0.625	755	0.02	-0.10	0.14	0.753
Free cholesterol in large HDL (mmol/l)	755	-0.11	-0.22	0.01	0.070	755	-0.04	-0.16	0.08	0.550	755	0.01	-0.11	0.13	0.857
Triglycerides in large HDL (mmol/l)	755	-0.05	-0.16	0.06	0.335	755	-0.01	-0.12	0.11	0.901	755	0.01	-0.10	0.13	0.821
Concentration of medium HDL particles (mol/l)	755	-0.15	-0.26	-0.05	0.005	755	-0.11	-0.22	0.00	0.052	755	-0.10	-0.21	0.01	0.080
Total lipids in medium HDL (mmol/l)	755	-0.15	-0.26	-0.05	0.005	755	-0.11	-0.22	0.00	0.053	755	-0.10	-0.21	0.02	0.095
Phospholipids in medium HDL (mmol/l)	755	-0.16	-0.26	-0.05	0.004	755	-0.12	-0.23	0.00	0.043	755	-0.10	-0.21	0.01	0.080
Total cholesterol in medium HDL (mmol/l)	755	-0.14	-0.25	-0.03	0.013	755	-0.09	-0.21	0.03	0.132	755	-0.07	-0.19	0.05	0.240
Cholesterol esters in medium HDL (mmol/l)	755	-0.14	-0.25	-0.03	0.016	755	-0.09	-0.21	0.03	0.147	755	-0.07	-0.19	0.05	0.264
Free cholesterol in medium HDL (mmol/l)	755	-0.14	-0.24	-0.03	0.010	755	-0.09	-0.20	0.02	0.097	755	-0.08	-0.19	0.03	0.168
Triglycerides in medium HDL (mmol/l)	755	-0.04	-0.15	0.07	0.460	755	-0.08	-0.19	0.04	0.204	755	-0.11	-0.22	0.01	0.067

S7 Table Associations of longer-term sedentary time (mean of SED measures at age 12y, 14y, and 15y) with metabolic traits at age 15y in ALSPAC

Mean of SED at age 12y, 14y, 15y (per SD (50 min/day) higher)

	Adj. for age, sex, ethnicity, maternal education, smoking, alcohol, mean wear time, wear month					*Additionally adj. for mean MVPA*					*Additionally adj. for mean FMI*				
**Standardised outcome at age 15y**	**N**	**Beta**	**LCL**	**UCL**	**P-value**	**N**	**Beta**	**LCL**	**UCL**	**P-value**	**N**	**Beta**	**LCL**	**UCL**	**P-value**
Concentration of small HDL particles (mol/l)	755	-0.11	-0.21	-0.01	0.038	755	-0.13	-0.24	-0.02	0.021	755	-0.15	-0.26	-0.04	0.008
Total lipids in small HDL (mmol/l)	755	-0.15	-0.25	-0.05	2.44E-03	755	-0.16	-0.26	-0.05	0.004	755	-0.17	-0.27	-0.06	2.25E-03
Phospholipids in small HDL (mmol/l)	755	-0.07	-0.17	0.04	0.209	755	-0.08	-0.19	0.03	0.168	755	-0.09	-0.21	0.02	0.106
Total cholesterol in small HDL (mmol/l)	755	-0.20	-0.30	-0.10	8.52E-05	755	-0.18	-0.29	-0.08	6.29E-04	755	-0.18	-0.28	-0.07	8.29E-04
Cholesterol esters in small HDL (mmol/l)	755	-0.20	-0.30	-0.10	1.16E-04	755	-0.18	-0.29	-0.08	6.65E-04	755	-0.18	-0.29	-0.08	8.05E-04
Free cholesterol in small HDL (mmol/l)	755	-0.13	-0.24	-0.02	0.017	755	-0.10	-0.22	0.01	0.077	755	-0.09	-0.21	0.02	0.104
Triglycerides in small HDL (mmol/l)	755	0.00	-0.12	0.11	0.955	755	-0.06	-0.19	0.06	0.309	755	-0.08	-0.20	0.03	0.163
Phospholipids to total lipds ratio in chylomicrons and extremely large VLDL (%)	755	-0.01	-0.11	0.09	0.896	755	-0.04	-0.15	0.07	0.472	755	-0.04	-0.14	0.07	0.519
Total cholesterol to total lipids ratio in chylomicrons and extremely large VLDL (%)	755	-0.11	-0.22	0.01	0.071	755	-0.11	-0.23	0.01	0.068	755	-0.13	-0.25	-0.01	0.028
Cholesterol esters to total lipids ratio in chylomicrons and extremely large VLDL (%)	755	-0.12	-0.23	-0.01	0.037	755	-0.11	-0.23	0.01	0.064	755	-0.14	-0.26	-0.02	0.027
Free cholesterol to total lipids ratio in chylomicrons and extremely large VLDL (%)	755	-0.02	-0.13	0.10	0.791	755	-0.06	-0.18	0.07	0.367	755	-0.07	-0.19	0.05	0.252
Triglycerides to total lipids ratio in chylomicrons and extremely large VLDL (%)	755	0.08	0.00	0.16	0.061	755	0.09	0.00	0.17	0.043	755	0.10	0.02	0.18	0.015
Phospholipids to total lipds ratio in very large VLDL (%)	755	-0.07	-0.19	0.04	0.228	755	-0.13	-0.25	-0.01	0.037	755	-0.15	-0.27	-0.02	0.019
Total cholesterol to total lipids ratio in very large VLDL (%)	755	-0.11	-0.23	0.01	0.069	755	-0.07	-0.19	0.06	0.311	755	-0.07	-0.21	0.06	0.295
Cholesterol esters to total lipids ratio in very large VLDL (%)	755	-0.08	-0.19	0.04	0.178	755	-0.03	-0.16	0.10	0.671	755	-0.03	-0.16	0.10	0.699
Free cholesterol to total lipids ratio in very large VLDL (%)	755	-0.11	-0.24	0.01	0.079	755	-0.09	-0.22	0.05	0.222	755	-0.08	-0.22	0.06	0.262
Triglycerides to total lipids ratio in very large VLDL (%)	755	0.13	0.01	0.25	0.039	755	0.10	-0.03	0.24	0.135	755	0.10	-0.03	0.24	0.130
Phospholipids to total lipds ratio in large VLDL (%)	755	-0.02	-0.14	0.11	0.787	755	-0.07	-0.20	0.06	0.305	755	-0.08	-0.21	0.05	0.214
Total cholesterol to total lipids ratio in large VLDL (%)	755	-0.10	-0.21	0.02	0.103	755	-0.11	-0.24	0.01	0.067	755	-0.14	-0.26	-0.01	0.031
Cholesterol esters to total lipids ratio in large VLDL (%)	755	-0.10	-0.22	0.02	0.097	755	-0.08	-0.19	0.02	0.130	755	-0.11	-0.26	0.03	0.125
Free cholesterol to total lipids ratio in large VLDL (%)	755	-0.02	-0.14	0.10	0.768	755	-0.07	-0.21	0.06	0.268	755	-0.10	-0.23	0.04	0.155
Triglycerides to total lipids ratio in large VLDL (%)	755	-0.04	-0.17	0.08	0.478	755	-0.03	-0.14	0.08	0.613	755	-0.05	-0.21	0.10	0.486
Phospholipids to total lipds ratio in medium VLDL (%)	755	-0.14	-0.27	-0.01	0.035	755	-0.11	-0.25	0.03	0.118	755	-0.07	-0.21	0.06	0.284
Total cholesterol to total lipids ratio in medium VLDL (%)	755	-0.12	-0.23	-0.01	0.037	755	-0.11	-0.22	0.01	0.076	755	-0.11	-0.23	0.01	0.068
Cholesterol esters to total lipids ratio in medium VLDL (%)	755	-0.11	-0.23	0.00	0.059	755	-0.08	-0.21	0.04	0.181	755	-0.09	-0.21	0.03	0.155
Free cholesterol to total lipids ratio in medium VLDL (%)	755	-0.08	-0.19	0.02	0.126	755	-0.13	-0.24	-0.01	0.032	755	-0.12	-0.24	-0.01	0.036
Triglycerides to total lipids ratio in medium VLDL (%)	755	0.14	0.02	0.25	0.018	755	0.12	0.00	0.24	0.050	755	0.12	0.00	0.24	0.057
Phospholipids to total lipds ratio in small VLDL (%)	755	-0.06	-0.18	0.06	0.334	755	0.01	-0.12	0.14	0.859	755	0.03	-0.09	0.16	0.611
Total cholesterol to total lipids ratio in small VLDL (%)	755	-0.08	-0.20	0.04	0.212	755	-0.07	-0.20	0.06	0.281	755	-0.07	-0.21	0.06	0.270
Cholesterol esters to total lipids ratio in small VLDL (%)	755	-0.05	-0.17	0.06	0.365	755	-0.06	-0.19	0.07	0.362	755	-0.07	-0.20	0.06	0.310
Free cholesterol to total lipids ratio in small VLDL (%)	755	-0.17	-0.30	-0.03	0.015	755	-0.10	-0.24	0.04	0.152	755	-0.07	-0.21	0.06	0.299
Triglycerides to total lipids ratio in small VLDL (%)	755	0.09	-0.03	0.21	0.133	755	0.07	-0.06	0.20	0.324	755	0.06	-0.07	0.19	0.358
Phospholipids to total lipds ratio in very small VLDL (%)	755	-0.12	-0.23	-0.01	0.027	755	-0.13	-0.24	-0.01	0.031	755	-0.11	-0.22	0.01	0.069
Total cholesterol to total lipids ratio in very small VLDL (%)	755	0.07	-0.04	0.18	0.225	755	0.10	-0.02	0.22	0.099	755	0.09	-0.03	0.21	0.153
Cholesterol esters to total lipids ratio in very small VLDL (%)	755	0.09	-0.01	0.19	0.079	755	0.09	-0.02	0.20	0.122	755	0.06	-0.04	0.17	0.245
Free cholesterol to total lipids ratio in very small VLDL (%)	755	-0.02	-0.15	0.11	0.762	755	0.08	-0.07	0.22	0.301	755	0.09	-0.06	0.23	0.234
Triglycerides to total lipids ratio in very small VLDL (%)	755	0.01	-0.11	0.12	0.916	755	-0.03	-0.15	0.10	0.641	755	-0.03	-0.15	0.10	0.667
Phospholipids to total lipds ratio in IDL (%)	755	-0.05	-0.18	0.09	0.506	755	-0.04	-0.19	0.10	0.579	755	-0.01	-0.16	0.13	0.870
Total cholesterol to total lipids ratio in IDL (%)	755	0.01	-0.11	0.13	0.812	755	0.02	-0.11	0.15	0.782	755	-0.01	-0.14	0.13	0.936
Cholesterol esters to total lipids ratio in IDL (%)	755	0.04	-0.08	0.16	0.531	755	0.03	-0.10	0.16	0.661	755	-0.01	-0.14	0.13	0.927
Free cholesterol to total lipids ratio in IDL (%)	755	-0.05	-0.16	0.05	0.319	755	-0.02	-0.14	0.09	0.674	755	0.00	-0.11	0.11	0.970
Triglycerides to total lipids ratio in IDL (%)	755	0.00	-0.11	0.12	0.940	755	0.00	-0.13	0.12	0.961	755	0.01	-0.11	0.14	0.847
Phospholipids to total lipds ratio in large LDL (%)	755	0.05	-0.05	0.15	0.312	755	0.06	-0.04	0.17	0.247	755	0.05	-0.05	0.16	0.322
Total cholesterol to total lipids ratio in large LDL (%)	755	-0.04	-0.15	0.07	0.450	755	-0.05	-0.18	0.07	0.393	755	-0.06	-0.19	0.06	0.331
Cholesterol esters to total lipids ratio in large LDL (%)	755	-0.05	-0.16	0.06	0.353	755	-0.08	-0.20	0.04	0.217	755	-0.09	-0.21	0.04	0.163
Free cholesterol to total lipids ratio in large LDL (%)	755	0.05	-0.05	0.14	0.336	755	0.09	-0.01	0.19	0.081	755	0.10	0.00	0.20	0.045
Triglycerides to total lipids ratio in large LDL (%)	755	0.01	-0.10	0.13	0.846	755	0.02	-0.11	0.14	0.809	755	0.04	-0.09	0.17	0.547
Phospholipids to total lipds ratio in medium LDL (%)	755	0.02	-0.02	0.06	0.320	755	0.03	-0.02	0.07	0.230	755	0.02	-0.02	0.07	0.292

S7 Table Associations of longer-term sedentary time (mean of SED measures at age 12y, 14y, and 15y) with metabolic traits at age 15y in ALSPAC

	**Mean of SED at age 12y, 14y, 15y (per SD (50 min/day) higher)**														
	Adj. for age, sex, ethnicity, maternal education, smoking, alcohol, mean wear time, wear month					*Additionally adj. for mean MVPA*					*Additionally adj. for mean FMI*				
**Standardised outcome at age 15y**	**N**	**Beta**	**LCL**	**UCL**	**P-value**	**N**	**Beta**	**LCL**	**UCL**	**P-value**	**N**	**Beta**	**LCL**	**UCL**	**P-value**
Total cholesterol to total lipids ratio in medium LDL (%)	755	-0.03	-0.15	0.09	0.600	755	-0.05	-0.17	0.08	0.482	755	-0.05	-0.18	0.08	0.456
Cholesterol esters to total lipids ratio in medium LDL (%)	755	-0.05	-0.17	0.07	0.416	755	-0.07	-0.20	0.06	0.268	755	-0.08	-0.21	0.06	0.260
Free cholesterol to total lipids ratio in medium LDL (%)	755	0.02	-0.01	0.05	0.257	755	0.03	-0.01	0.06	0.116	755	0.03	-0.01	0.06	0.117
Triglycerides to total lipids ratio in medium LDL (%)	755	-0.05	-0.16	0.06	0.379	755	-0.05	-0.18	0.07	0.400	755	-0.03	-0.15	0.10	0.666
Phospholipids to total lipds ratio in small LDL (%)	755	0.04	-0.03	0.10	0.291	755	0.05	-0.02	0.12	0.162	755	0.05	-0.02	0.13	0.170
Total cholesterol to total lipids ratio in small LDL (%)	755	-0.05	-0.16	0.07	0.435	755	-0.06	-0.19	0.07	0.354	755	-0.06	-0.19	0.07	0.345
Cholesterol esters to total lipids ratio in small LDL (%)	755	-0.06	-0.18	0.06	0.305	755	-0.09	-0.22	0.04	0.188	755	-0.09	-0.22	0.04	0.191
Free cholesterol to total lipids ratio in small LDL (%)	755	0.04	-0.02	0.10	0.207	755	0.06	-0.01	0.13	0.081	755	0.06	-0.01	0.13	0.087
Triglycerides to total lipids ratio in small LDL (%)	755	-0.03	-0.14	0.08	0.556	755	-0.07	-0.19	0.06	0.283	755	-0.06	-0.18	0.06	0.327
Phospholipids to total lipds ratio in very large HDL (%)	755	-0.07	-0.18	0.04	0.200	755	-0.02	-0.13	0.09	0.769	755	0.03	-0.08	0.13	0.623
Total cholesterol to total lipids ratio in very large HDL (%)	755	0.06	-0.04	0.16	0.268	755	0.01	-0.10	0.11	0.899	755	-0.03	-0.14	0.07	0.524
Cholesterol esters to total lipids ratio in very large HDL (%)	755	0.06	-0.05	0.16	0.289	755	0.01	-0.10	0.11	0.921	755	-0.04	-0.14	0.07	0.502
Free cholesterol to total lipids ratio in very large HDL (%)	755	0.00	-0.12	0.12	0.996	755	0.01	-0.11	0.14	0.833	755	0.03	-0.09	0.16	0.590
Triglycerides to total lipids ratio in very large HDL (%)	755	0.08	-0.05	0.20	0.223	755	0.05	-0.07	0.18	0.407	755	0.03	-0.09	0.16	0.614
Phospholipids to total lipds ratio in large HDL (%)	755	-0.04	-0.16	0.08	0.531	755	-0.12	-0.24	0.01	0.077	755	-0.16	-0.28	-0.04	0.010
Total cholesterol to total lipids ratio in large HDL (%)	755	0.00	-0.13	0.12	0.967	755	0.07	-0.06	0.20	0.293	755	0.12	-0.01	0.24	0.063
Cholesterol esters to total lipids ratio in large HDL (%)	755	0.00	-0.13	0.12	0.986	755	0.07	-0.06	0.20	0.278	755	0.12	0.00	0.25	0.058
Free cholesterol to total lipids ratio in large HDL (%)	755	-0.01	-0.12	0.11	0.920	755	0.05	-0.08	0.17	0.472	755	0.08	-0.04	0.20	0.191
Triglycerides to total lipids ratio in large HDL (%)	755	0.09	-0.04	0.21	0.187	755	0.04	-0.08	0.17	0.501	755	0.00	-0.12	0.13	0.989
Phospholipids to total lipds ratio in medium HDL (%)	755	-0.11	-0.23	0.01	0.070	755	-0.08	-0.21	0.05	0.207	755	-0.07	-0.20	0.06	0.283
Total cholesterol to total lipids ratio in medium HDL (%)	755	0.07	-0.04	0.19	0.217	755	0.09	-0.04	0.21	0.180	755	0.10	-0.03	0.23	0.119
Cholesterol esters to total lipids ratio in medium HDL (%)	755	0.07	-0.06	0.19	0.310	755	0.08	-0.05	0.21	0.253	755	0.09	-0.04	0.22	0.177
Free cholesterol to total lipids ratio in medium HDL (%)	755	0.07	-0.11	0.25	0.438	755	0.08	-0.10	0.26	0.402	755	0.08	-0.10	0.26	0.387
Triglycerides to total lipids ratio in medium HDL (%)	755	0.02	-0.10	0.14	0.725	755	-0.04	-0.16	0.09	0.592	755	-0.07	-0.20	0.05	0.247
Phospholipids to total lipds ratio in small HDL (%)	755	0.17	0.06	0.27	1.69E-03	755	0.15	0.04	0.26	0.009	755	0.14	0.03	0.25	0.015
Total cholesterol to total lipids ratio in small HDL (%)	755	-0.17	-0.28	-0.07	1.50E-03	755	-0.14	-0.25	-0.02	0.017	755	-0.13	-0.24	-0.01	0.031
Cholesterol esters to total lipids ratio in small HDL (%)	755	-0.17	-0.27	-0.06	1.68E-03	755	-0.15	-0.26	-0.03	0.011	755	-0.14	-0.25	-0.03	0.016
Free cholesterol to total lipids ratio in small HDL (%)	755	0.04	-0.08	0.16	0.489	755	0.12	-0.01	0.25	0.070	755	0.16	0.03	0.29	0.018
Triglycerides to total lipids ratio in small HDL (%)	755	0.07	-0.05	0.19	0.226	755	0.01	-0.12	0.13	0.923	755	-0.01	-0.14	0.11	0.816
Mean diameter for VLDL particles (nm)	755	0.06	-0.06	0.18	0.308	755	0.00	-0.12	0.13	0.963	755	-0.03	-0.15	0.09	0.645
Mean diameter for LDL particles (nm)	755	0.09	-0.01	0.20	0.077	755	0.13	0.02	0.24	0.020	755	0.14	0.03	0.25	0.011
Mean diameter for HDL particles (nm)	755	-0.08	-0.19	0.03	0.163	755	-0.01	-0.13	0.11	0.816	755	0.03	-0.08	0.15	0.568
Serum total cholesterol (mmol/l)	755	-0.13	-0.24	-0.02	0.019	755	-0.15	-0.27	-0.03	0.012	755	-0.15	-0.27	-0.03	0.013
Total cholesterol in VLDL (mmol/l)	755	0.00	-0.12	0.12	0.995	755	-0.05	-0.17	0.07	0.391	755	-0.09	-0.21	0.03	0.127
Remnant cholesterol (non-HDL, non-LDL -cholesterol) (mmol/l)	755	-0.05	-0.17	0.07	0.408	755	-0.09	-0.22	0.03	0.129	755	-0.12	-0.25	0.00	0.053
Total cholesterol in LDL (mmol/l)	755	-0.12	-0.24	0.00	0.043	755	-0.16	-0.28	-0.03	0.013	755	-0.16	-0.29	-0.04	0.011
Total cholesterol in HDL (mmol/l)	755	-0.15	-0.26	-0.04	0.010	755	-0.09	-0.21	0.03	0.135	755	-0.05	-0.17	0.06	0.385
Total cholesterol in HDL2 (mmol/l)	755	-0.14	-0.25	-0.03	0.015	755	-0.08	-0.20	0.04	0.180	755	-0.04	-0.16	0.08	0.491
Total cholesterol in HDL3 (mmol/l)	755	-0.15	-0.26	-0.04	0.006	755	-0.10	-0.21	0.01	0.087	755	-0.07	-0.18	0.05	0.246
Esterified cholesterol (mmol/l)	755	-0.14	-0.25	-0.03	0.016	755	-0.15	-0.27	-0.03	0.012	755	-0.15	-0.27	-0.03	0.014
Free cholesterol (mmol/l)	755	-0.12	-0.23	-0.01	0.034	755	-0.14	-0.26	-0.02	0.019	755	-0.14	-0.26	-0.03	0.018
Serum total triglycerides (mmol/l)	755	0.02	-0.09	0.14	0.702	755	-0.04	-0.16	0.08	0.526	755	-0.07	-0.18	0.05	0.262
Triglycerides in VLDL (mmol/l)	755	0.05	-0.06	0.17	0.372	755	-0.01	-0.13	0.11	0.908	755	-0.04	-0.15	0.07	0.483
Triglycerides in LDL (mmol/l)	755	-0.11	-0.22	0.00	0.056	755	-0.15	-0.27	-0.03	0.015	755	-0.14	-0.26	-0.02	0.025
Triglycerides in HDL (mmol/l)	755	-0.03	-0.14	0.08	0.589	755	-0.05	-0.17	0.06	0.357	755	-0.07	-0.18	0.05	0.258
Diacylglycerol (mmol/l)	755	0.02	-0.08	0.13	0.659	755	0.02	-0.10	0.13	0.763	755	0.00	-0.12	0.12	0.993
Ratio of diacylglycerol to triglycerides	755	0.00	-0.12	0.12	0.992	755	0.01	-0.12	0.13	0.935	755	0.00	-0.13	0.13	0.995
Total phosphoglycerides (mmol/l)	755	-0.16	-0.27	-0.05	0.004	755	-0.14	-0.26	-0.03	0.014	755	-0.13	-0.24	-0.01	0.030

S7 Table Associations of longer-term sedentary time (mean of SED measures at age 12y, 14y, and 15y) with metabolic traits at age 15y in ALSPAC

Mean of SED at age 12y, 14y, 15y (per SD (50 min/day) higher)

	Adj. for age, sex, ethnicity, maternal education, smoking, alcohol, mean wear time, wear month					*Additionally adj. for mean MVPA*					*Additionally adj. for mean FMI*				
**Standardised outcome at age 15y**	**N**	**Beta**	**LCL**	**UCL**	**P-value**	**N**	**Beta**	**LCL**	**UCL**	**P-value**	**N**	**Beta**	**LCL**	**UCL**	**P-value**
Ratio of triglycerides to phosphoglycerides	755	0.10	-0.01	0.21	0.082	755	0.05	-0.07	0.16	0.422	755	0.01	-0.10	0.12	0.852
Phosphatidylcholine and other cholines (mmol/l)	755	-0.14	-0.24	-0.03	0.009	755	-0.12	-0.23	-0.01	0.036	755	-0.10	-0.21	0.01	0.062
Total cholines (mmol/l)	755	-0.16	-0.26	-0.05	2.96E-03	755	-0.15	-0.26	-0.04	0.009	755	-0.13	-0.24	-0.02	0.019
Apolipoprotein A-I (g/l)	755	-0.15	-0.26	-0.05	0.005	755	-0.12	-0.23	-0.01	0.039	755	-0.09	-0.20	0.02	0.113
Apolipoprotein B (g/l)	755	-0.05	-0.16	0.07	0.448	755	-0.11	-0.23	0.02	0.095	755	-0.13	-0.26	-0.01	0.038
Ratio of apolipoprotein B to apolipoprotein A-I	755	0.03	-0.10	0.15	0.661	755	-0.05	-0.18	0.08	0.461	755	-0.09	-0.22	0.04	0.176
Total fatty acids (mmol/l)	755	-0.09	-0.19	0.02	0.104	755	-0.12	-0.23	-0.01	0.037	755	-0.12	-0.23	-0.02	0.025
Estimated description of fatty acid chain length, not actual carbon number	755	0.08	-0.03	0.18	0.156	755	0.08	-0.03	0.19	0.155	755	0.07	-0.04	0.18	0.217
Estimated degree of unsaturation	755	0.04	-0.07	0.14	0.519	755	0.05	-0.07	0.16	0.440	755	0.04	-0.07	0.16	0.472
22:6, docosahexaenoic acid (mmol/l)	755	-0.05	-0.17	0.06	0.355	755	-0.06	-0.18	0.06	0.310	755	-0.07	-0.19	0.05	0.257
18:2, linoleic acid (mmol/l)	755	-0.13	-0.23	-0.03	0.010	755	-0.16	-0.27	-0.05	0.005	755	-0.15	-0.26	-0.04	0.008
Conjugated linoleic acid (mmol/l)	755	0.02	-0.10	0.14	0.743	755	0.02	-0.11	0.15	0.817	755	0.02	-0.11	0.15	0.765
Omega-3 fatty acids (mmol/l)	755	-0.06	-0.17	0.05	0.309	755	-0.08	-0.20	0.04	0.195	755	-0.08	-0.20	0.04	0.176
Omega-6 fatty acids (mmol/l)	755	-0.12	-0.22	-0.01	0.025	755	-0.14	-0.25	-0.03	0.014	755	-0.14	-0.25	-0.03	0.015
Polyunsaturated fatty acids (mmol/l)	755	-0.11	-0.22	-0.01	0.030	755	-0.14	-0.25	-0.03	0.015	755	-0.14	-0.25	-0.03	0.016
Monounsaturated fatty acids; 16:1, 18:1 (mmol/l)	755	-0.04	-0.15	0.07	0.468	755	-0.08	-0.19	0.03	0.161	755	-0.10	-0.21	0.01	0.076
Saturated fatty acids (mmol/l)	755	-0.08	-0.18	0.02	0.128	755	-0.10	-0.21	0.01	0.079	755	-0.10	-0.21	0.01	0.066
Ratio of 22:6 docosahexaenoic acid to total fatty acids (%)	755	-0.01	-0.13	0.11	0.870	755	0.00	-0.13	0.13	0.999	755	0.00	-0.13	0.12	0.967
Ratio of 18:2 linoleic acid to total fatty acids (%)	755	-0.06	-0.16	0.05	0.287	755	-0.05	-0.16	0.07	0.456	755	-0.02	-0.14	0.10	0.708
Ratio of conjugated linoleic acid to total fatty acids (%)	755	0.04	-0.09	0.17	0.538	755	0.03	-0.10	0.17	0.631	755	0.04	-0.10	0.18	0.584
Ratio of omega-3 fatty acids to total fatty acids (%)	755	0.01	-0.11	0.12	0.924	755	0.00	-0.13	0.13	0.987	755	0.01	-0.13	0.14	0.936
Ratio of omega-6 fatty acids to total fatty acids (%)	755	-0.03	-0.13	0.08	0.636	755	0.00	-0.12	0.12	0.957	755	0.02	-0.10	0.13	0.796
Ratio of polyunsaturated fatty acids to total fatty acids (%)	755	-0.02	-0.13	0.09	0.670	755	0.00	-0.12	0.12	0.964	755	0.02	-0.10	0.14	0.790
Ratio of monounsaturated fatty acids to total fatty acids (%)	755	0.03	-0.08	0.14	0.559	755	0.00	-0.12	0.12	0.996	755	-0.03	-0.14	0.09	0.675
Ratio of saturated fatty acids to total fatty acids (%)	755	-0.01	-0.12	0.09	0.788	755	0.00	-0.11	0.12	0.958	755	0.01	-0.10	0.13	0.818
Insulin (mu/l)	755	0.06	-0.01	0.14	0.082	755	0.01	-0.07	0.08	0.848	755	-0.03	-0.10	0.05	0.456
Glucose (mmol/l)	755	0.03	-0.08	0.13	0.624	755	0.00	-0.12	0.11	0.988	755	0.00	-0.12	0.11	0.941
Lactate (mmol/l)	755	0.07	-0.05	0.19	0.274	755	0.05	-0.08	0.18	0.424	755	0.06	-0.07	0.19	0.361
Pyruvate (mmol/l)	755	0.16	0.05	0.27	0.006	755	0.14	0.02	0.26	0.023	755	0.13	0.01	0.25	0.038
Citrate (mmol/l)	755	-0.18	-0.31	-0.06	0.004	755	-0.16	-0.29	-0.03	0.018	755	-0.14	-0.27	-0.01	0.040
Alanine (mmol/l)	755	0.17	0.06	0.29	2.72E-03	755	0.16	0.04	0.28	0.008	755	0.17	0.05	0.29	0.007
Glutamine (mmol/l)	755	-0.01	-0.12	0.09	0.811	755	0.00	-0.12	0.11	0.973	755	0.02	-0.09	0.14	0.671
Histidine (mmol/l)	755	0.05	-0.06	0.15	0.410	755	0.09	-0.02	0.21	0.119	755	0.10	-0.02	0.21	0.104
Isoleucine (mmol/l)	755	0.06	-0.04	0.17	0.235	755	0.07	-0.05	0.18	0.251	755	0.05	-0.06	0.16	0.397
Leucine (mmol/l)	755	-0.01	-0.10	0.09	0.883	755	0.03	-0.07	0.13	0.543	755	0.03	-0.07	0.13	0.606
Valine (mmol/l)	755	0.00	-0.10	0.10	0.967	755	0.00	-0.11	0.11	0.985	755	-0.01	-0.13	0.10	0.809
Phenylalanine (mmol/l)	755	-0.01	-0.13	0.12	0.929	755	0.05	-0.08	0.17	0.472	755	0.03	-0.09	0.16	0.605
Tyrosine (mmol/l)	755	-0.03	-0.15	0.09	0.591	755	-0.01	-0.14	0.12	0.855	755	-0.03	-0.16	0.10	0.684
Acetate (mmol/l)	755	-0.06	-0.17	0.06	0.329	755	-0.01	-0.13	0.11	0.891	755	0.00	-0.12	0.12	0.992
Acetoacetate (mmol/l)	755	0.03	-0.07	0.14	0.564	755	0.01	-0.11	0.13	0.886	755	0.00	-0.12	0.12	0.967
3-hydroxybutyrate (mmol/l)	755	0.00	-0.13	0.13	0.955	755	-0.05	-0.20	0.09	0.475	755	-0.06	-0.21	0.09	0.433
Creatinine (mmol/l)	755	0.12	0.02	0.23	0.022	755	0.13	0.02	0.24	0.020	755	0.13	0.02	0.24	0.022
Albumin (signal area)	755	0.12	0.01	0.23	0.031	755	0.10	-0.02	0.21	0.109	755	0.10	-0.01	0.22	0.087
Glycoprotein acetyls, mainly a1-acid glycoprotein (mmol/l)	755	0.11	0.00	0.22	0.043	755	0.03	-0.08	0.14	0.580	755	-0.02	-0.13	0.09	0.727
C-reactive protein (mg/l)	755	0.12	-0.01	0.25	0.064	755	0.12	-0.02	0.27	0.094	755	0.12	-0.04	0.27	0.138
