# Supplementary material for: Associations of device-measured physical activity across adolescence with metabolic traits: Prospective cohort study
Source: PLoS Med. 2018 Sep 11;15(9):e1002649. doi: 10.1371/journal.pmed.1002649 (PMC6133272; doi:10.1371/journal.pmed.1002649)
Supplement: S8 Table — ALSPAC, Avon Longitudinal Study of Parents and Children. (PDF) [file pmed.1002649.s008.pdf]

S8 Table Interactions between current physical activity (measures at age 15y) with historical physical activity (mean of measures at age 12y and 14y) in relation to metabolic traits at age 15y in ALSPAC

Models adjusted for historical measure, age, age*historical measure, sex, sex*historical measure, ethnicity, ethnicity*historical measure, education, education*historical measure, smoking, smoking*historical measure, alcohol, alcohol*historical measure, wear time, wear time*historical measure, wear month, wear month*historical measure

	Interaction of CPM at age 15y with historical CPM (mean of CPM at 12y, 14y)					Interaction of MVPA at age 15y with historical MVPA (mean of MVPA at 12y, 14y)					Interaction of SED at age 15y with historical SED (mean of SED at 12y, 14y)				
**Standardised outcome at age 15y**	**N**	**Beta**	**LCI**	**UCI**	**P-value**	**N**	**Beta**	**LCI**	**UCI**	**P-value**	**N**	**Beta**	**LCI**	**UCI**	**P-value**
Systolic blood pressure (mmHg)	1298	-0.02	-0.09	0.05	0.566	1298	-0.02	-0.08	0.04	0.586	1298	0.00	-0.07	0.08	0.950
Diastolic blood pressure (mmHg)	1298	0.02	-0.05	0.09	0.622	1298	0.05	-0.02	0.12	0.135	1298	0.02	-0.05	0.08	0.596
Concentration of chylomicrons and extremely large VLDL particles (mol/l)	896	0.07	-0.02	0.15	0.128	896	0.04	-0.03	0.11	0.254	896	0.04	-0.06	0.15	0.413
Total lipids in chylomicrons and extremely large VLDL (mmol/l)	896	0.06	-0.03	0.14	0.168	896	0.04	-0.04	0.11	0.328	896	0.03	-0.07	0.14	0.517
Phospholipids in chylomicrons and extremely large VLDL (mmol/l)	896	0.06	-0.02	0.15	0.141	896	0.04	-0.03	0.11	0.276	896	0.04	-0.06	0.14	0.450
Total cholesterol in chylomicrons and extremely large VLDL (mmol/l)	896	0.06	-0.03	0.14	0.213	896	0.03	-0.04	0.10	0.443	896	0.02	-0.08	0.13	0.647
Cholesterol esters in chylomicrons and extremely large VLDL (mmol/l)	896	0.04	-0.04	0.13	0.324	896	0.02	-0.06	0.09	0.658	896	0.01	-0.09	0.11	0.835
Free cholesterol in chylomicrons and extremely large VLDL (mmol/l)	896	0.06	-0.02	0.15	0.137	896	0.04	-0.03	0.11	0.281	896	0.04	-0.07	0.14	0.485
Triglycerides in chylomicrons and extremely large VLDL (mmol/l)	896	0.06	-0.02	0.14	0.165	896	0.04	-0.04	0.11	0.316	896	0.04	-0.07	0.14	0.504
Concentration of very large VLDL particles (mol/l)	896	0.06	-0.03	0.15	0.168	896	0.04	-0.03	0.11	0.311	896	0.02	-0.08	0.13	0.635
Total lipids in very large VLDL (mmol/l)	896	0.06	-0.03	0.14	0.182	896	0.04	-0.04	0.11	0.335	896	0.02	-0.08	0.12	0.682
Phospholipids in very large VLDL (mmol/l)	896	0.07	-0.02	0.15	0.129	896	0.04	-0.03	0.11	0.246	896	0.03	-0.07	0.13	0.574
Total cholesterol in very large VLDL (mmol/l)	896	0.06	-0.03	0.15	0.174	896	0.03	-0.04	0.10	0.376	896	0.03	-0.07	0.13	0.577
Cholesterol esters in very large VLDL (mmol/l)	896	0.05	-0.03	0.14	0.224	896	0.03	-0.05	0.10	0.480	896	0.02	-0.08	0.13	0.672
Free cholesterol in very large VLDL (mmol/l)	896	0.07	-0.02	0.15	0.132	896	0.04	-0.03	0.11	0.284	896	0.04	-0.07	0.14	0.487
Triglycerides in very large VLDL (mmol/l)	896	0.06	-0.03	0.14	0.202	896	0.03	-0.04	0.11	0.350	896	0.02	-0.08	0.12	0.748
Concentration of large VLDL particles (mol/l)	896	0.05	-0.04	0.13	0.277	896	0.02	-0.05	0.10	0.522	896	0.00	-0.10	0.11	0.922
Total lipids in large VLDL (mmol/l)	896	0.05	-0.04	0.13	0.271	896	0.02	-0.05	0.10	0.531	896	0.01	-0.09	0.11	0.912
Phospholipids in large VLDL (mmol/l)	896	0.05	-0.03	0.14	0.219	896	0.03	-0.05	0.10	0.463	896	0.01	-0.09	0.11	0.833
Total cholesterol in large VLDL (mmol/l)	896	0.05	-0.03	0.14	0.222	896	0.02	-0.05	0.10	0.526	896	0.02	-0.09	0.12	0.762
Cholesterol esters in large VLDL (mmol/l)	896	0.05	-0.04	0.13	0.284	896	0.01	-0.06	0.09	0.723	896	0.01	-0.09	0.12	0.781
Free cholesterol in large VLDL (mmol/l)	896	0.06	-0.03	0.14	0.176	896	0.03	-0.04	0.11	0.371	896	0.02	-0.08	0.12	0.747
Triglycerides in large VLDL (mmol/l)	896	0.04	-0.04	0.13	0.312	896	0.02	-0.05	0.09	0.556	896	0.00	-0.10	0.10	0.995
Concentration of medium VLDL particles (mol/l)	896	0.04	-0.04	0.13	0.313	896	0.01	-0.06	0.09	0.694	896	0.01	-0.09	0.11	0.898
Total lipids in medium VLDL (mmol/l)	896	0.04	-0.04	0.13	0.323	896	0.01	-0.06	0.08	0.759	896	0.01	-0.09	0.11	0.869
Phospholipids in medium VLDL (mmol/l)	896	0.05	-0.03	0.13	0.247	896	0.01	-0.06	0.09	0.689	896	0.02	-0.08	0.12	0.739
Total cholesterol in medium VLDL (mmol/l)	896	0.05	-0.04	0.13	0.268	896	0.00	-0.07	0.07	0.939	896	0.03	-0.07	0.13	0.611
Cholesterol esters in medium VLDL (mmol/l)	896	0.04	-0.04	0.13	0.337	896	-0.01	-0.08	0.06	0.804	896	0.03	-0.07	0.13	0.531
Free cholesterol in medium VLDL (mmol/l)	896	0.05	-0.03	0.14	0.221	896	0.02	-0.06	0.09	0.654	896	0.02	-0.08	0.12	0.733
Triglycerides in medium VLDL (mmol/l)	896	0.04	-0.05	0.12	0.397	896	0.01	-0.06	0.09	0.707	896	0.00	-0.10	0.10	0.953
Concentration of small VLDL particles (mol/l)	896	0.06	-0.02	0.14	0.152	896	0.01	-0.06	0.08	0.718	896	0.05	-0.05	0.15	0.342
Total lipids in small VLDL (mmol/l)	896	0.06	-0.02	0.14	0.141	896	0.01	-0.06	0.08	0.779	896	0.06	-0.04	0.16	0.232
Phospholipids in small VLDL (mmol/l)	896	0.06	-0.02	0.14	0.153	896	0.01	-0.07	0.08	0.885	896	0.06	-0.03	0.16	0.189
Total cholesterol in small VLDL (mmol/l)	896	0.06	-0.02	0.14	0.120	896	0.01	-0.06	0.08	0.783	896	0.09	0.00	0.19	0.063
Cholesterol esters in small VLDL (mmol/l)	896	0.06	-0.02	0.13	0.158	896	0.01	-0.06	0.08	0.797	896	0.10	0.00	0.19	0.049
Free cholesterol in small VLDL (mmol/l)	896	0.07	-0.01	0.15	0.099	896	0.01	-0.06	0.08	0.789	896	0.07	-0.02	0.17	0.132
Triglycerides in small VLDL (mmol/l)	896	0.05	-0.03	0.14	0.213	896	0.01	-0.06	0.08	0.762	896	0.02	-0.07	0.12	0.631
Concentration of very small VLDL particles (mol/l)	896	0.07	0.00	0.15	0.048	896	0.00	-0.06	0.07	0.935	896	0.09	0.01	0.17	0.033
Total lipids in very small VLDL (mmol/l)	896	0.06	-0.01	0.14	0.095	896	0.00	-0.07	0.07	0.955	896	0.09	0.01	0.18	0.036
Phospholipids in very small VLDL (mmol/l)	896	0.08	0.00	0.15	0.057	896	0.01	-0.05	0.08	0.682	896	0.10	0.02	0.18	0.018
Total cholesterol in very small VLDL (mmol/l)	896	0.03	-0.05	0.11	0.440	896	-0.01	-0.08	0.06	0.697	896	0.08	-0.02	0.17	0.103
Cholesterol esters in very small VLDL (mmol/l)	896	0.03	-0.04	0.11	0.416	896	-0.01	-0.08	0.07	0.885	896	0.08	-0.01	0.17	0.086
Free cholesterol in very small VLDL (mmol/l)	896	0.02	-0.05	0.10	0.535	896	-0.03	-0.10	0.04	0.384	896	0.05	-0.03	0.14	0.223
Triglycerides in very small VLDL (mmol/l)	896	0.09	0.01	0.17	0.023	896	0.02	-0.05	0.09	0.583	896	0.08	-0.01	0.17	0.098
Concentration of IDL particles (mol/l)	896	0.08	0.00	0.17	0.043	896	0.03	-0.04	0.10	0.394	896	0.11	0.03	0.19	0.009
Total lipids in IDL (mmol/l)	896	0.08	-0.01	0.16	0.067	896	0.03	-0.05	0.10	0.491	896	0.11	0.03	0.19	0.010
Phospholipids in IDL (mmol/l)	896	0.08	0.00	0.16	0.061	896	0.03	-0.04	0.10	0.378	896	0.11	0.03	0.20	0.009

S8 Table Interactions between current physical activity (measures at age 15y) with historical physical activity (mean of measures at age 12y and 14y) in relation to metabolic traits at age 15y in ALSPAC

Models adjusted for historical measure, age, age*historical measure, sex, sex*historical measure, ethnicity, ethnicity*historical measure, education, education*historical measure, smoking, smoking*historical measure, alcohol, alcohol*historical measure, wear time, wear time*historical measure, wear month, wear month*historical measure

	Interaction of CPM at age 15y with historical CPM (mean of CPM at 12y, 14y)					Interaction of MVPA at age 15y with historical MVPA (mean of MVPA at 12y, 14y)					Interaction of SED at age 15y with historical SED (mean of SED at 12y, 14y)				
**Standardised outcome at age 15y**	**N**	**Beta**	**LCI**	**UCI**	**P-value**	**N**	**Beta**	**LCI**	**UCI**	**P-value**	**N**	**Beta**	**LCI**	**UCI**	**P-value**
Total cholesterol in IDL (mmol/l)	896	0.07	-0.02	0.15	0.118	896	0.02	-0.05	0.09	0.590	896	0.10	0.02	0.18	0.015
Cholesterol esters in IDL (mmol/l)	896	0.06	-0.02	0.15	0.130	896	0.02	-0.05	0.09	0.640	896	0.10	0.02	0.18	0.020
Free cholesterol in IDL (mmol/l)	896	0.07	-0.01	0.15	0.104	896	0.03	-0.05	0.10	0.494	896	0.11	0.02	0.19	0.010
Triglycerides in IDL (mmol/l)	896	0.10	0.03	0.17	0.005	896	0.03	-0.04	0.10	0.399	896	0.09	0.00	0.17	0.041
Concentration of large LDL particles (mol/l)	896	0.08	0.00	0.16	0.047	896	0.04	-0.03	0.11	0.315	896	0.12	0.03	0.20	0.009
Total lipids in large LDL (mmol/l)	896	0.08	0.00	0.16	0.054	896	0.03	-0.04	0.10	0.383	896	0.11	0.03	0.19	0.010
Phospholipids in large LDL (mmol/l)	896	0.08	0.00	0.16	0.059	896	0.03	-0.04	0.10	0.381	896	0.11	0.03	0.19	0.010
Total cholesterol in large LDL (mmol/l)	896	0.07	-0.01	0.16	0.072	896	0.03	-0.04	0.10	0.415	896	0.11	0.03	0.19	0.010
Cholesterol esters in large LDL (mmol/l)	896	0.08	-0.01	0.16	0.069	896	0.03	-0.04	0.10	0.410	896	0.11	0.02	0.19	0.011
Free cholesterol in large LDL (mmol/l)	896	0.07	-0.01	0.15	0.087	896	0.03	-0.04	0.10	0.438	896	0.11	0.03	0.19	0.009
Triglycerides in large LDL (mmol/l)	896	0.10	0.02	0.17	0.010	896	0.04	-0.03	0.11	0.301	896	0.08	-0.01	0.17	0.071
Concentration of medium LDL particles (mol/l)	896	0.08	0.00	0.16	0.038	896	0.04	-0.03	0.11	0.246	896	0.12	0.03	0.21	0.009
Total lipids in medium LDL (mmol/l)	896	0.08	0.00	0.16	0.049	896	0.03	-0.04	0.10	0.342	896	0.11	0.03	0.20	0.010
Phospholipids in medium LDL (mmol/l)	896	0.08	0.00	0.16	0.052	896	0.03	-0.04	0.10	0.426	896	0.10	0.02	0.19	0.014
Total cholesterol in medium LDL (mmol/l)	896	0.07	-0.01	0.15	0.066	896	0.03	-0.04	0.10	0.369	896	0.11	0.03	0.20	0.010
Cholesterol esters in medium LDL (mmol/l)	896	0.08	0.00	0.15	0.063	896	0.03	-0.04	0.10	0.351	896	0.11	0.03	0.20	0.011
Free cholesterol in medium LDL (mmol/l)	896	0.07	-0.01	0.15	0.089	896	0.03	-0.04	0.10	0.465	896	0.11	0.03	0.20	0.008
Triglycerides in medium LDL (mmol/l)	896	0.09	0.02	0.17	0.013	896	0.05	-0.02	0.12	0.188	896	0.09	-0.01	0.18	0.071
Concentration of small LDL particles (mol/l)	896	0.08	0.00	0.16	0.047	896	0.04	-0.03	0.11	0.257	896	0.12	0.03	0.21	0.012
Total lipids in small LDL (mmol/l)	896	0.08	0.00	0.16	0.056	896	0.03	-0.04	0.10	0.336	896	0.11	0.02	0.20	0.012
Phospholipids in small LDL (mmol/l)	896	0.08	0.00	0.15	0.063	896	0.03	-0.04	0.10	0.377	896	0.10	0.01	0.18	0.024
Total cholesterol in small LDL (mmol/l)	896	0.07	-0.01	0.15	0.078	896	0.03	-0.04	0.10	0.378	896	0.11	0.03	0.20	0.011
Cholesterol esters in small LDL (mmol/l)	896	0.07	0.00	0.15	0.066	896	0.04	-0.04	0.11	0.324	896	0.12	0.03	0.21	0.012
Free cholesterol in small LDL (mmol/l)	896	0.05	-0.03	0.13	0.194	896	0.01	-0.06	0.09	0.733	896	0.10	0.01	0.18	0.021
Triglycerides in small LDL (mmol/l)	896	0.10	0.02	0.17	0.010	896	0.05	-0.02	0.12	0.165	896	0.09	0.00	0.18	0.063
Concentration of very large HDL particles (mol/l)	896	0.03	-0.06	0.12	0.537	896	0.02	-0.06	0.10	0.682	896	-0.01	-0.10	0.08	0.881
Total lipids in very large HDL (mmol/l)	896	0.03	-0.06	0.12	0.551	896	0.02	-0.06	0.10	0.632	896	-0.01	-0.10	0.08	0.904
Phospholipids in very large HDL (mmol/l)	896	0.03	-0.06	0.12	0.509	896	0.02	-0.06	0.10	0.669	896	-0.01	-0.10	0.08	0.870
Total cholesterol in very large HDL (mmol/l)	896	0.02	-0.07	0.11	0.675	896	0.02	-0.06	0.10	0.605	896	0.00	-0.10	0.09	0.941
Cholesterol esters in very large HDL (mmol/l)	896	0.01	-0.07	0.10	0.749	896	0.02	-0.06	0.10	0.624	896	0.00	-0.09	0.09	0.963
Free cholesterol in very large HDL (mmol/l)	896	0.03	-0.06	0.12	0.522	896	0.02	-0.06	0.10	0.579	896	-0.01	-0.10	0.08	0.891
Triglycerides in very large HDL (mmol/l)	896	0.07	-0.03	0.16	0.154	896	0.01	-0.07	0.09	0.791	896	0.00	-0.09	0.09	0.956
Concentration of large HDL particles (mol/l)	896	0.01	-0.07	0.10	0.754	896	0.00	-0.07	0.08	0.905	896	-0.02	-0.11	0.07	0.680
Total lipids in large HDL (mmol/l)	896	0.02	-0.07	0.11	0.702	896	0.01	-0.07	0.08	0.856	896	-0.02	-0.11	0.07	0.649
Phospholipids in large HDL (mmol/l)	896	0.02	-0.07	0.11	0.632	896	0.01	-0.07	0.09	0.790	896	-0.01	-0.09	0.08	0.851
Total cholesterol in large HDL (mmol/l)	896	0.01	-0.08	0.10	0.783	896	0.00	-0.07	0.08	0.901	896	-0.03	-0.12	0.06	0.510
Cholesterol esters in large HDL (mmol/l)	896	0.01	-0.08	0.10	0.798	896	0.00	-0.07	0.08	0.913	896	-0.03	-0.12	0.06	0.490
Free cholesterol in large HDL (mmol/l)	896	0.02	-0.07	0.11	0.725	896	0.01	-0.07	0.08	0.858	896	-0.02	-0.11	0.06	0.593
Triglycerides in large HDL (mmol/l)	896	0.04	-0.05	0.12	0.372	896	-0.01	-0.09	0.06	0.762	896	-0.01	-0.09	0.07	0.802
Concentration of medium HDL particles (mol/l)	896	0.00	-0.07	0.08	0.918	896	-0.01	-0.08	0.06	0.784	896	0.00	-0.08	0.08	0.973
Total lipids in medium HDL (mmol/l)	896	0.01	-0.07	0.08	0.884	896	0.00	-0.07	0.06	0.891	896	0.00	-0.08	0.08	0.967
Phospholipids in medium HDL (mmol/l)	896	0.01	-0.07	0.08	0.854	896	-0.01	-0.08	0.06	0.793	896	0.00	-0.08	0.08	0.972
Total cholesterol in medium HDL (mmol/l)	896	0.00	-0.08	0.07	0.940	896	0.00	-0.07	0.07	0.978	896	-0.01	-0.09	0.07	0.883
Cholesterol esters in medium HDL (mmol/l)	896	-0.01	-0.08	0.07	0.838	896	0.00	-0.07	0.07	0.977	896	-0.01	-0.09	0.07	0.837
Free cholesterol in medium HDL (mmol/l)	896	0.02	-0.06	0.09	0.629	896	0.00	-0.07	0.07	0.998	896	0.01	-0.07	0.08	0.893
Triglycerides in medium HDL (mmol/l)	896	0.04	-0.04	0.12	0.347	896	0.00	-0.07	0.07	0.942	896	0.05	-0.04	0.13	0.323
Concentration of small HDL particles (mol/l)	896	0.01	-0.07	0.08	0.890	896	0.01	-0.07	0.08	0.874	896	0.03	-0.04	0.11	0.394

S8 Table Interactions between current physical activity (measures at age 15y) with historical physical activity (mean of measures at age 12y and 14y) in relation to metabolic traits at age 15y in ALSPAC

Models adjusted for historical measure, age, age*historical measure, sex, sex*historical measure, ethnicity, ethnicity*historical measure, education, education*historical measure, smoking, smoking*historical measure, alcohol, alcohol*historical measure, wear time, wear time*historical measure, wear month, wear month*historical measure

	Interaction of CPM at age 15y with historical CPM (mean of CPM at 12y, 14y)					Interaction of MVPA at age 15y with historical MVPA (mean of MVPA at 12y, 14y)					Interaction of SED at age 15y with historical SED (mean of SED at 12y, 14y)				
**Standardised outcome at age 15y**	**N**	**Beta**	**LCI**	**UCI**	**P-value**	**N**	**Beta**	**LCI**	**UCI**	**P-value**	**N**	**Beta**	**LCI**	**UCI**	**P-value**
Total lipids in small HDL (mmol/l)	896	0.01	-0.06	0.08	0.765	896	0.00	-0.07	0.08	0.940	896	0.03	-0.04	0.11	0.358
Phospholipids in small HDL (mmol/l)	896	-0.01	-0.09	0.07	0.803	896	0.00	-0.08	0.08	0.963	896	0.02	-0.06	0.09	0.695
Total cholesterol in small HDL (mmol/l)	896	0.02	-0.05	0.09	0.570	896	0.00	-0.06	0.06	0.977	896	0.04	-0.04	0.11	0.313
Cholesterol esters in small HDL (mmol/l)	896	0.03	-0.04	0.10	0.445	896	0.00	-0.06	0.07	0.910	896	0.04	-0.03	0.12	0.241
Free cholesterol in small HDL (mmol/l)	896	-0.02	-0.09	0.06	0.668	896	-0.01	-0.09	0.07	0.802	896	0.00	-0.08	0.07	0.910
Triglycerides in small HDL (mmol/l)	896	0.06	-0.02	0.14	0.153	896	0.01	-0.06	0.08	0.733	896	0.03	-0.06	0.13	0.452
Phospholipids to total lipds ratio in chylomicrons and extremely large VLDL (%)	896	0.05	-0.03	0.13	0.217	896	0.03	-0.05	0.11	0.429	896	0.06	-0.02	0.14	0.161
Total cholesterol to total lipids ratio in chylomicrons and extremely large VLDL (%)	896	0.02	-0.07	0.12	0.611	896	0.00	-0.08	0.08	0.944	896	0.02	-0.07	0.11	0.680
Cholesterol esters to total lipids ratio in chylomicrons and extremely large VLDL (%)	896	0.01	-0.08	0.10	0.801	896	0.00	-0.08	0.07	0.905	896	0.00	-0.09	0.09	0.949
Free cholesterol to total lipids ratio in chylomicrons and extremely large VLDL (%)	896	0.05	-0.04	0.13	0.282	896	0.00	-0.08	0.08	0.920	896	0.06	-0.03	0.15	0.215
Triglycerides to total lipids ratio in chylomicrons and extremely large VLDL (%)	896	-0.02	-0.08	0.04	0.553	896	0.00	-0.05	0.06	0.977	896	-0.02	-0.09	0.04	0.466
Phospholipids to total lipds ratio in very large VLDL (%)	896	0.07	-0.01	0.15	0.095	896	0.02	-0.06	0.10	0.594	896	0.08	-0.01	0.17	0.077
Total cholesterol to total lipids ratio in very large VLDL (%)	896	0.01	-0.10	0.12	0.864	896	0.04	-0.07	0.14	0.474	896	0.05	-0.04	0.14	0.272
Cholesterol esters to total lipids ratio in very large VLDL (%)	896	0.01	-0.09	0.11	0.806	896	0.05	-0.06	0.15	0.381	896	0.04	-0.05	0.14	0.368
Free cholesterol to total lipids ratio in very large VLDL (%)	896	0.07	-0.03	0.17	0.146	896	0.08	-0.01	0.18	0.089	896	0.06	-0.03	0.16	0.204
Triglycerides to total lipids ratio in very large VLDL (%)	896	-0.06	-0.17	0.04	0.232	896	-0.07	-0.18	0.03	0.155	896	-0.09	-0.18	0.01	0.079
Phospholipids to total lipds ratio in large VLDL (%)	896	0.00	-0.09	0.10	0.921	896	-0.05	-0.14	0.04	0.240	896	0.02	-0.08	0.12	0.724
Total cholesterol to total lipids ratio in large VLDL (%)	896	0.00	-0.08	0.08	0.941	896	-0.06	-0.14	0.02	0.123	896	0.08	-0.01	0.16	0.077
Cholesterol esters to total lipids ratio in large VLDL (%)	896	-0.06	-0.17	0.05	0.259	896	-0.08	-0.17	0.01	0.101	896	0.04	-0.01	0.09	0.145
Free cholesterol to total lipids ratio in large VLDL (%)	896	0.04	-0.04	0.12	0.316	896	-0.01	-0.09	0.07	0.833	896	0.04	-0.05	0.14	0.384
Triglycerides to total lipids ratio in large VLDL (%)	896	-0.06	-0.18	0.06	0.340	896	-0.04	-0.14	0.06	0.437	896	0.00	-0.05	0.04	0.853
Phospholipids to total lipds ratio in medium VLDL (%)	896	0.09	0.00	0.17	0.055	896	0.06	-0.03	0.15	0.211	896	0.09	0.00	0.18	0.057
Total cholesterol to total lipids ratio in medium VLDL (%)	896	0.03	-0.06	0.12	0.479	896	-0.02	-0.10	0.05	0.520	896	0.08	0.00	0.16	0.040
Cholesterol esters to total lipids ratio in medium VLDL (%)	896	0.01	-0.07	0.10	0.742	896	-0.03	-0.10	0.04	0.441	896	0.08	-0.01	0.16	0.082
Free cholesterol to total lipids ratio in medium VLDL (%)	896	0.07	-0.01	0.15	0.082	896	0.00	-0.07	0.08	0.905	896	0.06	-0.03	0.14	0.170
Triglycerides to total lipids ratio in medium VLDL (%)	896	-0.05	-0.13	0.04	0.316	896	0.01	-0.06	0.08	0.784	896	-0.09	-0.17	-0.02	0.015
Phospholipids to total lipds ratio in small VLDL (%)	896	-0.03	-0.11	0.05	0.429	896	-0.01	-0.08	0.06	0.814	896	-0.01	-0.11	0.08	0.812
Total cholesterol to total lipids ratio in small VLDL (%)	896	0.01	-0.07	0.10	0.743	896	0.02	-0.06	0.10	0.650	896	0.08	0.00	0.17	0.059
Cholesterol esters to total lipids ratio in small VLDL (%)	896	0.01	-0.07	0.09	0.858	896	0.02	-0.06	0.10	0.669	896	0.08	-0.01	0.16	0.092
Free cholesterol to total lipids ratio in small VLDL (%)	896	0.04	-0.04	0.12	0.316	896	0.01	-0.06	0.08	0.756	896	0.08	-0.01	0.17	0.071
Triglycerides to total lipids ratio in small VLDL (%)	896	0.00	-0.09	0.08	0.923	896	-0.02	-0.10	0.06	0.701	896	-0.08	-0.17	0.01	0.091
Phospholipids to total lipds ratio in very small VLDL (%)	896	0.08	-0.01	0.17	0.098	896	0.04	-0.04	0.12	0.298	896	0.08	0.01	0.16	0.033
Total cholesterol to total lipids ratio in very small VLDL (%)	896	-0.09	-0.17	-0.01	0.034	896	-0.04	-0.12	0.03	0.268	896	-0.05	-0.14	0.04	0.268
Cholesterol esters to total lipids ratio in very small VLDL (%)	896	-0.07	-0.15	0.01	0.084	896	-0.02	-0.09	0.06	0.653	896	-0.03	-0.12	0.05	0.412
Free cholesterol to total lipids ratio in very small VLDL (%)	896	-0.07	-0.15	0.00	0.059	896	-0.07	-0.15	0.00	0.053	896	-0.05	-0.16	0.05	0.311
Triglycerides to total lipids ratio in very small VLDL (%)	896	0.05	-0.04	0.13	0.261	896	0.02	-0.06	0.10	0.594	896	0.00	-0.09	0.09	0.989
Phospholipids to total lipds ratio in IDL (%)	896	0.00	-0.08	0.08	1.000	896	0.03	-0.04	0.10	0.343	896	0.00	-0.10	0.11	0.985
Total cholesterol to total lipids ratio in IDL (%)	896	-0.04	-0.12	0.04	0.332	896	-0.02	-0.09	0.06	0.696	896	0.01	-0.09	0.10	0.895
Cholesterol esters to total lipids ratio in IDL (%)	896	-0.04	-0.12	0.04	0.292	896	-0.02	-0.10	0.05	0.542	896	-0.01	-0.12	0.09	0.778
Free cholesterol to total lipids ratio in IDL (%)	896	0.01	-0.08	0.09	0.903	896	0.02	-0.05	0.09	0.615	896	0.05	-0.03	0.13	0.250
Triglycerides to total lipids ratio in IDL (%)	896	0.05	-0.03	0.13	0.235	896	0.00	-0.08	0.08	0.945	896	-0.01	-0.10	0.08	0.854
Phospholipids to total lipds ratio in large LDL (%)	896	-0.07	-0.15	0.01	0.094	896	-0.03	-0.10	0.04	0.393	896	-0.09	-0.16	-0.03	0.007
Total cholesterol to total lipids ratio in large LDL (%)	896	0.02	-0.06	0.10	0.591	896	0.02	-0.05	0.10	0.566	896	0.07	0.00	0.15	0.059
Cholesterol esters to total lipids ratio in large LDL (%)	896	0.04	-0.04	0.13	0.298	896	0.03	-0.05	0.10	0.453	896	0.08	0.01	0.16	0.035
Free cholesterol to total lipids ratio in large LDL (%)	896	-0.07	-0.15	0.00	0.054	896	-0.03	-0.10	0.04	0.389	896	-0.06	-0.14	0.02	0.140
Triglycerides to total lipids ratio in large LDL (%)	896	0.04	-0.03	0.12	0.284	896	0.00	-0.08	0.07	0.982	896	-0.01	-0.11	0.08	0.758
Phospholipids to total lipds ratio in medium LDL (%)	896	-0.02	-0.05	0.01	0.137	896	-0.01	-0.04	0.01	0.325	896	-0.04	-0.07	-0.01	0.009

S8 Table Interactions between current physical activity (measures at age 15y) with historical physical activity (mean of measures at age 12y and 14y) in relation to metabolic traits at age 15y in ALSPAC

Models adjusted for historical measure, age, age*historical measure, sex, sex*historical measure, ethnicity, ethnicity*historical measure, education, education*historical measure, smoking, smoking*historical measure, alcohol, alcohol*historical measure, wear time, wear time*historical measure, wear month, wear month*historical measure

	Interaction of CPM at age 15y with historical CPM (mean of CPM at 12y, 14y)					Interaction of MVPA at age 15y with historical MVPA (mean of MVPA at 12y, 14y)					Interaction of SED at age 15y with historical SED (mean of SED at 12y, 14y)				
**Standardised outcome at age 15y**	**N**	**Beta**	**LCI**	**UCI**	**P-value**	**N**	**Beta**	**LCI**	**UCI**	**P-value**	**N**	**Beta**	**LCI**	**UCI**	**P-value**
Total cholesterol to total lipids ratio in medium LDL (%)	896	0.03	-0.06	0.11	0.497	896	0.03	-0.05	0.10	0.531	896	0.08	0.01	0.16	0.035
Cholesterol esters to total lipids ratio in medium LDL (%)	896	0.05	-0.03	0.14	0.215	896	0.03	-0.04	0.11	0.389	896	0.10	0.02	0.18	0.018
Free cholesterol to total lipids ratio in medium LDL (%)	896	-0.02	-0.04	0.00	0.060	896	-0.01	-0.03	0.01	0.278	896	-0.03	-0.05	0.00	0.019
Triglycerides to total lipids ratio in medium LDL (%)	896	0.07	-0.01	0.14	0.073	896	0.02	-0.05	0.10	0.516	896	0.02	-0.07	0.12	0.648
Phospholipids to total lipds ratio in small LDL (%)	896	-0.04	-0.08	0.01	0.129	896	-0.02	-0.07	0.02	0.327	896	-0.06	-0.11	-0.02	0.007
Total cholesterol to total lipids ratio in small LDL (%)	896	0.03	-0.05	0.12	0.435	896	0.03	-0.05	0.10	0.511	896	0.09	0.01	0.17	0.023
Cholesterol esters to total lipids ratio in small LDL (%)	896	0.06	-0.03	0.14	0.181	896	0.04	-0.04	0.12	0.328	896	0.10	0.02	0.18	0.014
Free cholesterol to total lipids ratio in small LDL (%)	896	-0.05	-0.09	0.00	0.039	896	-0.03	-0.07	0.01	0.174	896	-0.05	-0.10	-0.01	0.025
Triglycerides to total lipids ratio in small LDL (%)	896	0.08	0.00	0.15	0.059	896	0.03	-0.05	0.10	0.488	896	0.03	-0.06	0.11	0.563
Phospholipids to total lipds ratio in very large HDL (%)	896	0.02	-0.07	0.10	0.728	896	-0.01	-0.08	0.07	0.886	896	-0.01	-0.10	0.08	0.855
Total cholesterol to total lipids ratio in very large HDL (%)	896	-0.03	-0.11	0.06	0.534	896	0.01	-0.07	0.08	0.847	896	0.00	-0.08	0.09	0.953
Cholesterol esters to total lipids ratio in very large HDL (%)	896	-0.03	-0.12	0.06	0.516	896	0.00	-0.07	0.08	0.926	896	0.00	-0.08	0.09	0.935
Free cholesterol to total lipids ratio in very large HDL (%)	896	0.03	-0.06	0.11	0.537	896	0.04	-0.03	0.11	0.301	896	-0.01	-0.10	0.08	0.787
Triglycerides to total lipids ratio in very large HDL (%)	896	0.06	-0.04	0.15	0.224	896	-0.01	-0.08	0.06	0.827	896	0.03	-0.07	0.14	0.540
Phospholipids to total lipds ratio in large HDL (%)	896	0.04	-0.04	0.12	0.344	896	0.02	-0.04	0.08	0.454	896	0.11	0.02	0.21	0.019
Total cholesterol to total lipids ratio in large HDL (%)	896	-0.04	-0.13	0.04	0.337	896	-0.02	-0.08	0.05	0.613	896	-0.10	-0.20	0.00	0.057
Cholesterol esters to total lipids ratio in large HDL (%)	896	-0.05	-0.13	0.04	0.259	896	-0.02	-0.08	0.04	0.497	896	-0.11	-0.22	0.00	0.041
Free cholesterol to total lipids ratio in large HDL (%)	896	-0.01	-0.09	0.08	0.843	896	0.01	-0.06	0.08	0.883	896	-0.05	-0.13	0.04	0.320
Triglycerides to total lipids ratio in large HDL (%)	896	0.04	-0.06	0.13	0.441	896	0.00	-0.07	0.06	0.894	896	0.04	-0.07	0.15	0.448
Phospholipids to total lipds ratio in medium HDL (%)	896	0.01	-0.07	0.08	0.880	896	-0.03	-0.10	0.04	0.390	896	0.00	-0.10	0.10	0.986
Total cholesterol to total lipids ratio in medium HDL (%)	896	-0.03	-0.11	0.05	0.452	896	0.02	-0.05	0.10	0.506	896	-0.03	-0.13	0.06	0.488
Cholesterol esters to total lipids ratio in medium HDL (%)	896	-0.04	-0.12	0.04	0.279	896	0.02	-0.05	0.09	0.561	896	-0.04	-0.13	0.06	0.466
Free cholesterol to total lipids ratio in medium HDL (%)	896	0.06	-0.02	0.14	0.162	896	0.03	-0.05	0.10	0.506	896	0.00	-0.11	0.11	0.977
Triglycerides to total lipids ratio in medium HDL (%)	896	0.05	-0.04	0.13	0.299	896	0.00	-0.07	0.08	0.970	896	0.06	-0.04	0.16	0.239
Phospholipids to total lipds ratio in small HDL (%)	896	-0.04	-0.12	0.04	0.319	896	0.00	-0.07	0.06	0.925	896	-0.04	-0.12	0.04	0.367
Total cholesterol to total lipids ratio in small HDL (%)	896	0.02	-0.06	0.10	0.583	896	0.00	-0.07	0.07	0.997	896	0.03	-0.05	0.11	0.503
Cholesterol esters to total lipids ratio in small HDL (%)	896	0.03	-0.05	0.11	0.442	896	0.00	-0.06	0.07	0.892	896	0.04	-0.04	0.12	0.342
Free cholesterol to total lipids ratio in small HDL (%)	896	-0.07	-0.15	0.00	0.061	896	-0.03	-0.10	0.04	0.336	896	-0.09	-0.19	0.00	0.055
Triglycerides to total lipids ratio in small HDL (%)	896	0.06	-0.02	0.14	0.132	896	0.01	-0.06	0.09	0.732	896	0.03	-0.07	0.12	0.583
Mean diameter for VLDL particles (nm)	896	0.01	-0.07	0.09	0.744	896	0.00	-0.07	0.08	0.979	896	-0.02	-0.12	0.07	0.619
Mean diameter for LDL particles (nm)	896	-0.02	-0.09	0.05	0.542	896	-0.04	-0.11	0.03	0.235	896	-0.05	-0.13	0.03	0.256
Mean diameter for HDL particles (nm)	896	0.01	-0.08	0.11	0.767	896	0.00	-0.08	0.09	0.925	896	-0.02	-0.11	0.06	0.606
Serum total cholesterol (mmol/l)	896	0.07	-0.01	0.15	0.096	896	0.02	-0.05	0.10	0.495	896	0.09	0.01	0.17	0.025
Total cholesterol in VLDL (mmol/l)	896	0.06	-0.02	0.14	0.171	896	0.01	-0.06	0.08	0.851	896	0.06	-0.04	0.16	0.228
Remnant cholesterol (non-HDL, non-LDL -cholesterol) (mmol/l)	896	0.07	-0.01	0.15	0.095	896	0.01	-0.05	0.08	0.682	896	0.09	0.00	0.18	0.058
Total cholesterol in LDL (mmol/l)	896	0.07	-0.01	0.15	0.071	896	0.03	-0.04	0.10	0.393	896	0.11	0.03	0.20	0.010
Total cholesterol in HDL (mmol/l)	896	0.01	-0.07	0.10	0.731	896	0.01	-0.06	0.08	0.814	896	-0.01	-0.09	0.08	0.858
Total cholesterol in HDL2 (mmol/l)	896	0.01	-0.08	0.09	0.868	896	0.01	-0.06	0.08	0.791	896	-0.01	-0.10	0.07	0.792
Total cholesterol in HDL3 (mmol/l)	896	0.03	-0.06	0.11	0.522	896	0.01	-0.07	0.08	0.864	896	0.00	-0.08	0.08	0.999
Esterified cholesterol (mmol/l)	896	0.07	-0.01	0.15	0.099	896	0.03	-0.04	0.10	0.381	896	0.08	0.00	0.16	0.039
Free cholesterol (mmol/l)	896	0.07	-0.01	0.15	0.102	896	0.01	-0.06	0.08	0.830	896	0.10	0.02	0.18	0.012
Serum total triglycerides (mmol/l)	896	0.07	-0.02	0.15	0.115	896	0.02	-0.05	0.10	0.498	896	0.03	-0.07	0.13	0.556
Triglycerides in VLDL (mmol/l)	896	0.05	-0.04	0.13	0.255	896	0.02	-0.05	0.09	0.600	896	0.01	-0.09	0.11	0.818
Triglycerides in LDL (mmol/l)	896	0.10	0.02	0.17	0.009	896	0.04	-0.03	0.11	0.229	896	0.08	-0.01	0.17	0.065
Triglycerides in HDL (mmol/l)	896	0.06	-0.02	0.14	0.133	896	0.00	-0.07	0.07	0.904	896	0.03	-0.06	0.12	0.504
Diacylglycerol (mmol/l)	864	0.03	-0.05	0.10	0.530	864	-0.02	-0.10	0.06	0.582	864	0.00	-0.10	0.10	0.950
Ratio of diacylglycerol to triglycerides	864	0.00	-0.07	0.07	0.981	864	-0.03	-0.12	0.05	0.461	864	-0.01	-0.10	0.09	0.852

S8 Table Interactions between current physical activity (measures at age 15y) with historical physical activity (mean of measures at age 12y and 14y) in relation to metabolic traits at age 15y in ALSPAC

Models adjusted for historical measure, age, age*historical measure, sex, sex*historical measure, ethnicity, ethnicity*historical measure, education, education*historical measure, smoking, smoking*historical measure, alcohol, alcohol*historical measure, wear time, wear time*historical measure, wear month, wear month*historical measure

	Interaction of CPM at age 15y with historical CPM (mean of CPM at 12y, 14y)					Interaction of MVPA at age 15y with historical MVPA (mean of MVPA at 12y, 14y)					Interaction of SED at age 15y with historical SED (mean of SED at 12y, 14y)				
**Standardised outcome at age 15y**	**N**	**Beta**	**LCI**	**UCI**	**P-value**	**N**	**Beta**	**LCI**	**UCI**	**P-value**	**N**	**Beta**	**LCI**	**UCI**	**P-value**
Total phosphoglycerides (mmol/l)	896	0.03	-0.05	0.11	0.401	896	0.01	-0.06	0.08	0.704	896	0.05	-0.03	0.13	0.241
Ratio of triglycerides to phosphoglycerides	896	0.05	-0.04	0.13	0.306	896	0.00	-0.07	0.07	0.989	896	-0.02	-0.11	0.08	0.739
Phosphatidylcholine and other cholines (mmol/l)	877	0.04	-0.03	0.12	0.288	877	-0.03	-0.10	0.04	0.396	877	0.07	-0.01	0.15	0.074
Total cholines (mmol/l)	893	0.05	-0.03	0.13	0.217	893	0.00	-0.06	0.07	0.907	893	0.07	-0.01	0.14	0.099
Apolipoprotein A-I (g/l)	896	0.04	-0.04	0.12	0.338	896	0.02	-0.05	0.09	0.611	896	0.02	-0.06	0.09	0.673
Apolipoprotein B (g/l)	896	0.08	0.00	0.16	0.042	896	0.03	-0.04	0.10	0.444	896	0.09	0.00	0.18	0.058
Ratio of apolipoprotein B to apolipoprotein A-I	896	0.07	-0.02	0.15	0.113	896	0.02	-0.05	0.09	0.582	896	0.09	-0.01	0.19	0.091
Total fatty acids (mmol/l)	896	0.06	-0.01	0.14	0.111	896	0.03	-0.04	0.10	0.455	896	0.03	-0.06	0.11	0.525
Estimated description of fatty acid chain length, not actual carbon number	892	0.05	-0.04	0.13	0.307	892	-0.03	-0.12	0.07	0.586	892	0.12	0.03	0.21	0.010
Estimated degree of unsaturation	895	0.00	-0.08	0.08	0.983	895	-0.04	-0.12	0.04	0.298	895	0.11	0.02	0.21	0.020
22:6, docosahexaenoic acid (mmol/l)	896	0.10	0.02	0.18	0.017	896	0.06	-0.02	0.14	0.131	896	0.04	-0.05	0.14	0.399
18:2, linoleic acid (mmol/l)	893	0.06	-0.01	0.13	0.074	893	0.03	-0.04	0.09	0.420	893	0.06	-0.02	0.14	0.128
Conjugated linoleic acid (mmol/l)	896	0.06	-0.02	0.14	0.157	896	0.01	-0.07	0.09	0.841	896	0.05	-0.05	0.15	0.325
Omega-3 fatty acids (mmol/l)	894	0.09	0.01	0.17	0.027	894	0.07	-0.01	0.15	0.078	894	0.04	-0.05	0.13	0.413
Omega-6 fatty acids (mmol/l)	895	0.05	-0.02	0.12	0.154	895	0.02	-0.05	0.08	0.638	895	0.06	-0.02	0.13	0.135
Polyunsaturated fatty acids (mmol/l)	893	0.06	-0.01	0.13	0.112	893	0.02	-0.04	0.09	0.495	893	0.05	-0.02	0.13	0.180
Monounsaturated fatty acids; 16:1, 18:1 (mmol/l)	893	0.09	0.01	0.17	0.036	893	0.03	-0.05	0.10	0.497	893	0.06	-0.03	0.16	0.168
Saturated fatty acids (mmol/l)	892	0.02	-0.06	0.10	0.598	892	0.02	-0.06	0.09	0.636	892	-0.03	-0.12	0.06	0.469
Ratio of 22:6 docosahexaenoic acid to total fatty acids (%)	896	0.09	-0.01	0.18	0.064	896	0.07	-0.02	0.16	0.135	896	0.02	-0.07	0.12	0.600
Ratio of 18:2 linoleic acid to total fatty acids (%)	893	0.02	-0.06	0.10	0.619	893	0.03	-0.06	0.11	0.518	893	0.06	-0.04	0.15	0.238
Ratio of conjugated linoleic acid to total fatty acids (%)	896	0.05	-0.03	0.13	0.220	896	0.00	-0.07	0.08	0.949	896	0.05	-0.05	0.15	0.335
Ratio of omega-3 fatty acids to total fatty acids (%)	894	0.07	-0.01	0.15	0.103	894	0.08	0.00	0.17	0.062	894	0.01	-0.07	0.10	0.741
Ratio of omega-6 fatty acids to total fatty acids (%)	895	-0.02	-0.10	0.06	0.587	895	-0.01	-0.09	0.08	0.884	895	0.06	-0.03	0.15	0.224
Ratio of polyunsaturated fatty acids to total fatty acids (%)	893	0.00	-0.09	0.08	0.910	893	0.02	-0.06	0.10	0.680	893	0.05	-0.04	0.14	0.251
Ratio of monounsaturated fatty acids to total fatty acids (%)	893	0.08	-0.01	0.17	0.099	893	0.00	-0.08	0.09	0.956	893	0.08	-0.01	0.18	0.070
Ratio of saturated fatty acids to total fatty acids (%)	892	-0.09	-0.18	-0.01	0.032	892	-0.02	-0.10	0.06	0.592	892	-0.18	-0.27	-0.08	3.95E-04
Insulin (mu/l)	927	-0.02	-0.07	0.03	0.398	927	0.00	-0.05	0.06	0.912	927	-0.04	-0.10	0.01	0.124
Glucose (mmol/l)	894	0.00	-0.06	0.07	0.976	894	0.01	-0.05	0.08	0.720	894	-0.01	-0.10	0.08	0.828
Lactate (mmol/l)	894	0.02	-0.06	0.10	0.589	894	0.02	-0.06	0.10	0.558	894	-0.03	-0.12	0.07	0.588
Pyruvate (mmol/l)	893	0.00	-0.08	0.08	0.991	893	0.00	-0.08	0.08	0.919	893	-0.02	-0.11	0.07	0.645
Citrate (mmol/l)	891	0.08	0.00	0.17	0.047	891	0.04	-0.04	0.11	0.330	891	0.10	-0.01	0.20	0.063
Alanine (mmol/l)	896	-0.01	-0.09	0.07	0.808	896	0.00	-0.08	0.08	0.939	896	-0.03	-0.11	0.06	0.580
Glutamine (mmol/l)	896	0.04	-0.03	0.12	0.267	896	0.05	-0.01	0.11	0.129	896	0.01	-0.08	0.09	0.878
Histidine (mmol/l)	850	0.00	-0.09	0.09	0.959	850	0.00	-0.08	0.09	0.934	850	-0.02	-0.11	0.06	0.586
Isoleucine (mmol/l)	896	-0.11	-0.18	-0.04	0.003	896	-0.06	-0.12	0.01	0.106	896	-0.06	-0.15	0.02	0.154
Leucine (mmol/l)	896	-0.09	-0.16	-0.03	0.006	896	-0.02	-0.09	0.04	0.461	896	-0.06	-0.13	0.02	0.137
Valine (mmol/l)	896	-0.11	-0.18	-0.04	2.70E-03	896	-0.03	-0.09	0.04	0.386	896	-0.03	-0.11	0.05	0.413
Phenylalanine (mmol/l)	895	-0.04	-0.11	0.04	0.328	895	0.01	-0.07	0.09	0.817	895	-0.04	-0.14	0.06	0.459
Tyrosine (mmol/l)	892	0.01	-0.07	0.09	0.830	892	0.03	-0.06	0.11	0.538	892	-0.03	-0.14	0.07	0.547
Acetate (mmol/l)	895	-0.02	-0.08	0.05	0.656	895	0.03	-0.04	0.11	0.333	895	0.00	-0.09	0.09	0.992
Acetoacetate (mmol/l)	896	-0.05	-0.14	0.05	0.323	896	-0.03	-0.10	0.05	0.489	896	-0.04	-0.15	0.07	0.450
3-hydroxybutyrate (mmol/l)	895	0.04	-0.03	0.11	0.213	895	0.04	-0.03	0.11	0.304	895	0.06	-0.04	0.16	0.213
Creatinine (mmol/l)	895	-0.02	-0.10	0.05	0.538	895	0.02	-0.05	0.09	0.549	895	-0.11	-0.20	-0.03	0.009
Albumin (signal area)	896	0.04	-0.04	0.12	0.345	896	0.05	-0.03	0.13	0.220	896	-0.01	-0.11	0.09	0.887
Glycoprotein acetyls, mainly a1-acid glycoprotein (mmol/l)	895	0.01	-0.06	0.09	0.756	895	0.00	-0.08	0.07	0.908	895	-0.02	-0.11	0.07	0.713
C-reactive protein (mg/l)	929	0.01	-0.03	0.05	0.563	929	0.00	-0.05	0.04	0.935	929	0.02	-0.09	0.14	0.692

S8 Table Interactions between current physical activity (measures at age 15y) with historical physical activity (mean of measures at age 12y and 14y) in relation to metabolic traits at age 15y in ALSPAC

Models adjusted for historical measure, age, age*historical measure, sex, sex*historical measure, ethnicity, ethnicity*historical measure, education, education*historical measure, smoking, smoking*historical measure, alcohol, alcohol*historical measure, wear time, wear time*historical measure, wear month, wear month*historical measure

	Interaction of CPM at age 15y with historical CPM (mean of CPM at 12y, 14y)					Interaction of MVPA at age 15y with historical MVPA (mean of MVPA at 12y, 14y)					Interaction of SED at age 15y with historical SED (mean of SED at 12y, 14y)				
**Standardised outcome at age 15y**	**N**	**Beta**	**LCI**	**UCI**	**P-value**	**N**	**Beta**	**LCI**	**UCI**	**P-value**	**N**	**Beta**	**LCI**	**UCI**	**P-value**

Complete case sample

	Interaction of CPM at age 15y with historical CPM (mean of CPM at 12y, 14y)					Interaction of MVPA at age 15y with historical MVPA (mean of MVPA at 12y, 14y)					Interaction of SED at age 15y with historical SED (mean of SED at 12y, 14y)				
**Standardised outcome at age 15y**	**N**	**Beta**	**LCI**	**UCI**	**P-value**	**N**	**Beta**	**LCI**	**UCI**	**P-value**	**N**	**Beta**	**LCI**	**UCI**	**P-value**
Systolic blood pressure (mmHg)	755	-0.07	-0.15	0.01	0.082	755	-0.07	-0.14	0.00	0.055	755	0.03	-0.08	0.14	0.622
Diastolic blood pressure (mmHg)	755	-0.02	-0.10	0.06	0.706	755	0.03	-0.06	0.11	0.540	755	0.04	-0.06	0.14	0.424
Concentration of chylomicrons and extremely large VLDL particles (mol/l)	755	0.06	-0.02	0.14	0.125	755	0.04	-0.03	0.11	0.238	755	0.07	-0.04	0.19	0.203
Total lipids in chylomicrons and extremely large VLDL (mmol/l)	755	0.05	-0.03	0.13	0.207	755	0.03	-0.04	0.10	0.366	755	0.06	-0.05	0.18	0.269
Phospholipids in chylomicrons and extremely large VLDL (mmol/l)	755	0.05	-0.03	0.13	0.185	755	0.04	-0.03	0.10	0.316	755	0.07	-0.04	0.18	0.240
Total cholesterol in chylomicrons and extremely large VLDL (mmol/l)	755	0.05	-0.03	0.13	0.228	755	0.02	-0.04	0.09	0.493	755	0.06	-0.05	0.17	0.309
Cholesterol esters in chylomicrons and extremely large VLDL (mmol/l)	755	0.04	-0.04	0.12	0.320	755	0.01	-0.05	0.07	0.748	755	0.05	-0.06	0.16	0.391
Free cholesterol in chylomicrons and extremely large VLDL (mmol/l)	755	0.06	-0.02	0.14	0.168	755	0.04	-0.03	0.10	0.313	755	0.06	-0.05	0.18	0.255
Triglycerides in chylomicrons and extremely large VLDL (mmol/l)	755	0.05	-0.03	0.13	0.208	755	0.03	-0.04	0.10	0.353	755	0.06	-0.05	0.18	0.269
Concentration of very large VLDL particles (mol/l)	755	0.06	-0.02	0.14	0.164	755	0.04	-0.03	0.11	0.270	755	0.06	-0.05	0.17	0.309
Total lipids in very large VLDL (mmol/l)	755	0.05	-0.03	0.13	0.195	755	0.03	-0.03	0.10	0.321	755	0.05	-0.06	0.16	0.337
Phospholipids in very large VLDL (mmol/l)	755	0.06	-0.02	0.14	0.135	755	0.04	-0.03	0.11	0.234	755	0.06	-0.05	0.17	0.283
Total cholesterol in very large VLDL (mmol/l)	755	0.06	-0.02	0.14	0.172	755	0.03	-0.04	0.10	0.381	755	0.06	-0.05	0.18	0.268
Cholesterol esters in very large VLDL (mmol/l)	755	0.05	-0.03	0.14	0.207	755	0.03	-0.04	0.09	0.469	755	0.06	-0.05	0.17	0.304
Free cholesterol in very large VLDL (mmol/l)	755	0.06	-0.02	0.14	0.143	755	0.04	-0.03	0.10	0.302	755	0.07	-0.04	0.18	0.237
Triglycerides in very large VLDL (mmol/l)	755	0.05	-0.03	0.13	0.224	755	0.03	-0.03	0.10	0.331	755	0.05	-0.06	0.16	0.379
Concentration of large VLDL particles (mol/l)	755	0.04	-0.04	0.13	0.304	755	0.03	-0.05	0.10	0.488	755	0.04	-0.07	0.15	0.457
Total lipids in large VLDL (mmol/l)	755	0.04	-0.04	0.13	0.306	755	0.02	-0.05	0.09	0.520	755	0.04	-0.07	0.15	0.451
Phospholipids in large VLDL (mmol/l)	755	0.05	-0.03	0.13	0.258	755	0.03	-0.04	0.10	0.474	755	0.05	-0.06	0.15	0.402
Total cholesterol in large VLDL (mmol/l)	755	0.05	-0.04	0.13	0.259	755	0.02	-0.05	0.09	0.574	755	0.05	-0.06	0.16	0.342
Cholesterol esters in large VLDL (mmol/l)	755	0.04	-0.04	0.13	0.332	755	0.01	-0.06	0.08	0.811	755	0.05	-0.06	0.16	0.336
Free cholesterol in large VLDL (mmol/l)	755	0.05	-0.03	0.13	0.204	755	0.03	-0.04	0.10	0.384	755	0.05	-0.06	0.16	0.352
Triglycerides in large VLDL (mmol/l)	755	0.04	-0.04	0.12	0.344	755	0.02	-0.05	0.10	0.517	755	0.04	-0.07	0.14	0.516
Concentration of medium VLDL particles (mol/l)	755	0.04	-0.04	0.13	0.318	755	0.02	-0.06	0.09	0.632	755	0.04	-0.07	0.15	0.439
Total lipids in medium VLDL (mmol/l)	755	0.04	-0.04	0.13	0.339	755	0.01	-0.06	0.09	0.729	755	0.05	-0.06	0.15	0.416
Phospholipids in medium VLDL (mmol/l)	755	0.05	-0.04	0.13	0.286	755	0.01	-0.06	0.09	0.725	755	0.05	-0.06	0.16	0.336
Total cholesterol in medium VLDL (mmol/l)	755	0.04	-0.04	0.13	0.338	755	-0.01	-0.08	0.07	0.876	755	0.06	-0.05	0.17	0.253
Cholesterol esters in medium VLDL (mmol/l)	755	0.04	-0.05	0.12	0.426	755	-0.02	-0.09	0.05	0.560	755	0.07	-0.04	0.18	0.211
Free cholesterol in medium VLDL (mmol/l)	755	0.05	-0.04	0.13	0.270	755	0.01	-0.06	0.09	0.716	755	0.05	-0.06	0.16	0.338
Triglycerides in medium VLDL (mmol/l)	755	0.04	-0.05	0.12	0.375	755	0.02	-0.05	0.10	0.572	755	0.03	-0.08	0.14	0.554
Concentration of small VLDL particles (mol/l)	755	0.07	-0.02	0.15	0.130	755	0.02	-0.06	0.09	0.611	755	0.08	-0.02	0.19	0.118
Total lipids in small VLDL (mmol/l)	755	0.07	-0.02	0.16	0.118	755	0.02	-0.06	0.09	0.677	755	0.10	-0.01	0.20	0.077
Phospholipids in small VLDL (mmol/l)	755	0.07	-0.02	0.16	0.109	755	0.01	-0.06	0.09	0.707	755	0.11	0.00	0.21	0.043
Total cholesterol in small VLDL (mmol/l)	755	0.07	-0.01	0.16	0.103	755	0.01	-0.06	0.08	0.780	755	0.12	0.02	0.22	0.024
Cholesterol esters in small VLDL (mmol/l)	755	0.07	-0.02	0.16	0.123	755	0.01	-0.06	0.08	0.797	755	0.12	0.01	0.22	0.026
Free cholesterol in small VLDL (mmol/l)	755	0.07	-0.01	0.16	0.093	755	0.01	-0.06	0.08	0.771	755	0.11	0.01	0.21	0.032
Triglycerides in small VLDL (mmol/l)	755	0.06	-0.03	0.14	0.193	755	0.02	-0.06	0.10	0.627	755	0.06	-0.05	0.16	0.272
Concentration of very small VLDL particles (mol/l)	755	0.07	-0.01	0.15	0.079	755	-0.01	-0.08	0.06	0.723	755	0.12	0.03	0.20	0.011
Total lipids in very small VLDL (mmol/l)	755	0.07	-0.01	0.15	0.101	755	-0.01	-0.08	0.07	0.843	755	0.11	0.02	0.21	0.017
Phospholipids in very small VLDL (mmol/l)	755	0.06	-0.02	0.15	0.126	755	-0.01	-0.09	0.06	0.759	755	0.11	0.03	0.20	0.009
Total cholesterol in very small VLDL (mmol/l)	755	0.05	-0.04	0.13	0.274	755	-0.01	-0.09	0.06	0.723	755	0.09	-0.01	0.18	0.067
Cholesterol esters in very small VLDL (mmol/l)	755	0.05	-0.03	0.14	0.225	755	0.00	-0.07	0.08	0.994	755	0.09	0.00	0.19	0.060

S8 Table Interactions between current physical activity (measures at age 15y) with historical physical activity (mean of measures at age 12y and 14y) in relation to metabolic traits at age 15y in ALSPAC

Models adjusted for historical measure, age, age*historical measure, sex, sex*historical measure, ethnicity, ethnicity*historical measure, education, education*historical measure, smoking, smoking*historical measure, alcohol, alcohol*historical measure, wear time, wear time*historical measure, wear month, wear month*historical measure

	Interaction of CPM at age 15y with historical CPM (mean of CPM at 12y, 14y)					Interaction of MVPA at age 15y with historical MVPA (mean of MVPA at 12y, 14y)					Interaction of SED at age 15y with historical SED (mean of SED at 12y, 14y)				
**Standardised outcome at age 15y**	**N**	**Beta**	**LCI**	**UCI**	**P-value**	**N**	**Beta**	**LCI**	**UCI**	**P-value**	**N**	**Beta**	**LCI**	**UCI**	**P-value**
Free cholesterol in very small VLDL (mmol/l)	755	0.03	-0.05	0.11	0.465	755	-0.04	-0.12	0.04	0.293	755	0.07	-0.02	0.16	0.149
Triglycerides in very small VLDL (mmol/l)	755	0.09	0.01	0.17	0.026	755	0.02	-0.05	0.09	0.627	755	0.11	0.01	0.20	0.032
Concentration of IDL particles (mol/l)	755	0.06	-0.02	0.15	0.144	755	0.00	-0.08	0.07	0.923	755	0.12	0.03	0.21	0.007
Total lipids in IDL (mmol/l)	755	0.06	-0.03	0.14	0.192	755	-0.01	-0.09	0.07	0.816	755	0.12	0.03	0.20	0.007
Phospholipids in IDL (mmol/l)	755	0.06	-0.03	0.14	0.207	755	0.00	-0.08	0.07	0.915	755	0.12	0.03	0.21	0.008
Total cholesterol in IDL (mmol/l)	755	0.05	-0.04	0.14	0.268	755	-0.01	-0.09	0.07	0.740	755	0.11	0.03	0.20	0.010
Cholesterol esters in IDL (mmol/l)	755	0.05	-0.04	0.14	0.270	755	-0.01	-0.09	0.06	0.722	755	0.11	0.03	0.20	0.011
Free cholesterol in IDL (mmol/l)	755	0.05	-0.04	0.13	0.284	755	-0.01	-0.09	0.07	0.793	755	0.11	0.02	0.19	0.012
Triglycerides in IDL (mmol/l)	755	0.09	0.01	0.16	0.020	755	0.01	-0.06	0.08	0.834	755	0.10	0.01	0.18	0.029
Concentration of large LDL particles (mol/l)	755	0.06	-0.03	0.14	0.176	755	0.00	-0.08	0.08	0.965	755	0.13	0.04	0.22	0.005
Total lipids in large LDL (mmol/l)	755	0.06	-0.03	0.14	0.188	755	0.00	-0.08	0.07	0.927	755	0.12	0.04	0.21	0.006
Phospholipids in large LDL (mmol/l)	755	0.06	-0.03	0.14	0.198	755	0.00	-0.08	0.07	0.935	755	0.13	0.04	0.22	0.005
Total cholesterol in large LDL (mmol/l)	755	0.05	-0.03	0.14	0.223	755	-0.01	-0.08	0.07	0.900	755	0.12	0.03	0.21	0.007
Cholesterol esters in large LDL (mmol/l)	755	0.05	-0.03	0.14	0.217	755	0.00	-0.08	0.07	0.909	755	0.12	0.03	0.21	0.007
Free cholesterol in large LDL (mmol/l)	755	0.05	-0.04	0.14	0.249	755	-0.01	-0.09	0.07	0.875	755	0.12	0.03	0.21	0.007
Triglycerides in large LDL (mmol/l)	755	0.07	0.00	0.15	0.063	755	0.01	-0.07	0.08	0.858	755	0.09	0.00	0.17	0.056
Concentration of medium LDL particles (mol/l)	755	0.06	-0.02	0.15	0.148	755	0.01	-0.07	0.09	0.800	755	0.13	0.04	0.23	0.005
Total lipids in medium LDL (mmol/l)	755	0.06	-0.03	0.15	0.166	755	0.00	-0.08	0.08	0.961	755	0.13	0.04	0.22	0.005
Phospholipids in medium LDL (mmol/l)	755	0.06	-0.03	0.15	0.181	755	0.00	-0.08	0.07	0.921	755	0.13	0.04	0.22	0.004
Total cholesterol in medium LDL (mmol/l)	755	0.06	-0.03	0.14	0.193	755	0.00	-0.08	0.08	0.973	755	0.13	0.04	0.22	0.006
Cholesterol esters in medium LDL (mmol/l)	755	0.06	-0.03	0.14	0.186	755	0.00	-0.08	0.08	0.941	755	0.12	0.03	0.22	0.008
Free cholesterol in medium LDL (mmol/l)	755	0.05	-0.04	0.14	0.236	755	-0.01	-0.09	0.08	0.897	755	0.13	0.04	0.22	0.003
Triglycerides in medium LDL (mmol/l)	755	0.07	-0.01	0.15	0.087	755	0.02	-0.06	0.09	0.639	755	0.09	0.00	0.18	0.060
Concentration of small LDL particles (mol/l)	755	0.06	-0.03	0.14	0.189	755	0.01	-0.07	0.09	0.807	755	0.13	0.04	0.23	0.006
Total lipids in small LDL (mmol/l)	755	0.06	-0.03	0.14	0.191	755	0.00	-0.07	0.08	0.930	755	0.13	0.04	0.22	0.007
Phospholipids in small LDL (mmol/l)	755	0.05	-0.04	0.14	0.254	755	0.00	-0.08	0.08	0.936	755	0.12	0.03	0.21	0.008
Total cholesterol in small LDL (mmol/l)	755	0.05	-0.03	0.14	0.216	755	0.00	-0.08	0.08	0.950	755	0.13	0.03	0.22	0.008
Cholesterol esters in small LDL (mmol/l)	755	0.06	-0.03	0.14	0.198	755	0.01	-0.07	0.08	0.879	755	0.13	0.03	0.22	0.009
Free cholesterol in small LDL (mmol/l)	755	0.04	-0.05	0.14	0.346	755	-0.01	-0.10	0.07	0.756	755	0.12	0.03	0.20	0.008
Triglycerides in small LDL (mmol/l)	755	0.07	0.00	0.15	0.054	755	0.03	-0.05	0.10	0.487	755	0.10	0.01	0.20	0.033
Concentration of very large HDL particles (mol/l)	755	-0.03	-0.13	0.07	0.512	755	-0.04	-0.14	0.05	0.357	755	-0.03	-0.12	0.06	0.509
Total lipids in very large HDL (mmol/l)	755	-0.03	-0.13	0.07	0.533	755	-0.04	-0.13	0.06	0.424	755	-0.03	-0.12	0.06	0.514
Phospholipids in very large HDL (mmol/l)	755	-0.03	-0.13	0.07	0.528	755	-0.04	-0.14	0.05	0.348	755	-0.03	-0.12	0.06	0.501
Total cholesterol in very large HDL (mmol/l)	755	-0.03	-0.13	0.07	0.521	755	-0.03	-0.12	0.07	0.570	755	-0.03	-0.12	0.07	0.549
Cholesterol esters in very large HDL (mmol/l)	755	-0.03	-0.13	0.07	0.519	755	-0.02	-0.12	0.07	0.623	755	-0.03	-0.12	0.07	0.540
Free cholesterol in very large HDL (mmol/l)	755	-0.03	-0.13	0.07	0.540	755	-0.04	-0.13	0.06	0.458	755	-0.03	-0.12	0.07	0.592
Triglycerides in very large HDL (mmol/l)	755	0.02	-0.07	0.11	0.648	755	-0.05	-0.13	0.03	0.228	755	-0.01	-0.10	0.08	0.832
Concentration of large HDL particles (mol/l)	755	-0.05	-0.15	0.05	0.336	755	-0.06	-0.15	0.03	0.212	755	-0.04	-0.13	0.05	0.417
Total lipids in large HDL (mmol/l)	755	-0.04	-0.14	0.05	0.366	755	-0.05	-0.14	0.04	0.234	755	-0.04	-0.13	0.05	0.412
Phospholipids in large HDL (mmol/l)	755	-0.04	-0.14	0.06	0.398	755	-0.05	-0.14	0.04	0.247	755	-0.03	-0.12	0.06	0.569
Total cholesterol in large HDL (mmol/l)	755	-0.05	-0.15	0.05	0.343	755	-0.05	-0.14	0.04	0.241	755	-0.05	-0.14	0.05	0.316
Cholesterol esters in large HDL (mmol/l)	755	-0.05	-0.15	0.05	0.333	755	-0.05	-0.14	0.04	0.236	755	-0.05	-0.15	0.05	0.303
Free cholesterol in large HDL (mmol/l)	755	-0.04	-0.14	0.05	0.387	755	-0.05	-0.14	0.04	0.261	755	-0.04	-0.13	0.05	0.374
Triglycerides in large HDL (mmol/l)	755	-0.02	-0.11	0.06	0.631	755	-0.08	-0.15	0.00	0.058	755	-0.02	-0.11	0.06	0.577
Concentration of medium HDL particles (mol/l)	755	-0.05	-0.14	0.04	0.283	755	-0.06	-0.14	0.03	0.192	755	0.02	-0.07	0.10	0.675
Total lipids in medium HDL (mmol/l)	755	-0.05	-0.14	0.04	0.277	755	-0.05	-0.14	0.03	0.222	755	0.01	-0.07	0.10	0.729
Phospholipids in medium HDL (mmol/l)	755	-0.04	-0.13	0.05	0.364	755	-0.05	-0.14	0.03	0.214	755	0.02	-0.07	0.10	0.728

S8 Table Interactions between current physical activity (measures at age 15y) with historical physical activity (mean of measures at age 12y and 14y) in relation to metabolic traits at age 15y in ALSPAC

Models adjusted for historical measure, age, age*historical measure, sex, sex*historical measure, ethnicity, ethnicity*historical measure, education, education*historical measure, smoking, smoking*historical measure, alcohol, alcohol*historical measure, wear time, wear time*historical measure, wear month, wear month*historical measure

	Interaction of CPM at age 15y with historical CPM (mean of CPM at 12y, 14y)					Interaction of MVPA at age 15y with historical MVPA (mean of MVPA at 12y, 14y)					Interaction of SED at age 15y with historical SED (mean of SED at 12y, 14y)				
**Standardised outcome at age 15y**	**N**	**Beta**	**LCI**	**UCI**	**P-value**	**N**	**Beta**	**LCI**	**UCI**	**P-value**	**N**	**Beta**	**LCI**	**UCI**	**P-value**
Total cholesterol in medium HDL (mmol/l)	755	-0.06	-0.15	0.03	0.172	755	-0.05	-0.13	0.04	0.264	755	0.00	-0.09	0.09	0.980
Cholesterol esters in medium HDL (mmol/l)	755	-0.07	-0.16	0.02	0.141	755	-0.05	-0.13	0.04	0.275	755	0.00	-0.09	0.09	0.958
Free cholesterol in medium HDL (mmol/l)	755	-0.04	-0.12	0.05	0.415	755	-0.05	-0.14	0.03	0.232	755	0.02	-0.07	0.10	0.708
Triglycerides in medium HDL (mmol/l)	755	0.03	-0.06	0.12	0.528	755	-0.01	-0.09	0.07	0.777	755	0.07	-0.02	0.17	0.129
Concentration of small HDL particles (mol/l)	755	-0.01	-0.09	0.08	0.887	755	0.01	-0.08	0.10	0.892	755	0.07	-0.01	0.14	0.100
Total lipids in small HDL (mmol/l)	755	-0.01	-0.09	0.08	0.875	755	-0.01	-0.10	0.08	0.826	755	0.06	-0.01	0.14	0.101
Phospholipids in small HDL (mmol/l)	755	-0.02	-0.10	0.06	0.628	755	0.01	-0.09	0.10	0.909	755	0.04	-0.03	0.12	0.271
Total cholesterol in small HDL (mmol/l)	755	0.00	-0.08	0.08	0.980	755	-0.03	-0.10	0.05	0.520	755	0.06	-0.02	0.13	0.156
Cholesterol esters in small HDL (mmol/l)	755	0.01	-0.07	0.09	0.825	755	-0.02	-0.10	0.05	0.546	755	0.06	-0.02	0.14	0.137
Free cholesterol in small HDL (mmol/l)	755	-0.04	-0.12	0.04	0.342	755	-0.02	-0.11	0.06	0.590	755	0.02	-0.06	0.10	0.612
Triglycerides in small HDL (mmol/l)	755	0.06	-0.02	0.14	0.141	755	0.02	-0.06	0.09	0.649	755	0.06	-0.04	0.16	0.216
Phospholipids to total lipids ratio in chylomicrons and extremely large VLDL (%)	755	0.03	-0.06	0.12	0.495	755	0.00	-0.09	0.09	0.959	755	0.06	-0.02	0.15	0.130
Total cholesterol to total lipids ratio in chylomicrons and extremely large VLDL (%)	755	0.01	-0.10	0.11	0.902	755	-0.03	-0.12	0.06	0.460	755	0.05	-0.05	0.15	0.354
Cholesterol esters to total lipids ratio in chylomicrons and extremely large VLDL (%)	755	0.00	-0.10	0.11	0.931	755	-0.03	-0.11	0.06	0.569	755	0.03	-0.07	0.13	0.506
Free cholesterol to total lipids ratio in chylomicrons and extremely large VLDL (%)	755	0.01	-0.08	0.10	0.840	755	-0.04	-0.13	0.05	0.350	755	0.07	-0.03	0.16	0.157
Triglycerides to total lipids ratio in chylomicrons and extremely large VLDL (%)	755	0.00	-0.08	0.07	0.897	755	0.02	-0.04	0.09	0.440	755	-0.04	-0.11	0.03	0.231
Phospholipids to total lipds ratio in very large VLDL (%)	755	0.04	-0.05	0.13	0.382	755	-0.02	-0.10	0.07	0.731	755	0.10	0.00	0.19	0.049
Total cholesterol to total lipids ratio in very large VLDL (%)	755	0.03	-0.08	0.14	0.598	755	0.05	-0.07	0.17	0.385	755	0.05	-0.04	0.14	0.300
Cholesterol esters to total lipids ratio in very large VLDL (%)	755	0.04	-0.06	0.14	0.451	755	0.07	-0.06	0.19	0.298	755	0.04	-0.05	0.14	0.406
Free cholesterol to total lipids ratio in very large VLDL (%)	755	0.08	-0.04	0.19	0.183	755	0.07	-0.05	0.19	0.251	755	0.08	-0.03	0.18	0.145
Triglycerides to total lipids ratio in very large VLDL (%)	755	-0.07	-0.19	0.04	0.210	755	-0.07	-0.19	0.06	0.279	755	-0.09	-0.19	0.01	0.065
Phospholipids to total lipds ratio in large VLDL (%)	755	-0.03	-0.12	0.07	0.597	755	-0.08	-0.19	0.02	0.120	755	0.02	-0.08	0.13	0.643
Total cholesterol to total lipids ratio in large VLDL (%)	755	-0.03	-0.13	0.06	0.487	755	-0.10	-0.19	-0.01	0.025	755	0.10	0.01	0.19	0.038
Cholesterol esters to total lipids ratio in large VLDL (%)	755	-0.07	-0.17	0.04	0.200	755	-0.08	-0.15	-0.01	0.033	755	0.05	-0.02	0.11	0.186
Free cholesterol to total lipids ratio in large VLDL (%)	755	0.01	-0.07	0.10	0.772	755	-0.04	-0.12	0.05	0.435	755	0.05	-0.05	0.16	0.332
Triglycerides to total lipids ratio in large VLDL (%)	755	-0.05	-0.16	0.06	0.406	755	-0.01	-0.09	0.06	0.717	755	-0.02	-0.08	0.05	0.615
Phospholipids to total lipds ratio in medium VLDL (%)	755	0.07	-0.04	0.18	0.197	755	0.02	-0.09	0.14	0.681	755	0.09	-0.01	0.18	0.081
Total cholesterol to total lipids ratio in medium VLDL (%)	755	0.00	-0.09	0.10	0.938	755	-0.07	-0.15	0.02	0.109	755	0.11	0.02	0.19	0.011
Cholesterol esters to total lipids ratio in medium VLDL (%)	755	-0.01	-0.11	0.09	0.864	755	-0.07	-0.15	0.02	0.115	755	0.10	0.01	0.19	0.022
Free cholesterol to total lipids ratio in medium VLDL (%)	755	0.04	-0.04	0.12	0.330	755	-0.03	-0.11	0.05	0.474	755	0.07	-0.01	0.15	0.101
Triglycerides to total lipids ratio in medium VLDL (%)	755	-0.02	-0.12	0.08	0.735	755	0.06	-0.03	0.14	0.192	755	-0.11	-0.19	-0.03	0.005
Phospholipids to total lipds ratio in small VLDL (%)	755	-0.02	-0.11	0.06	0.628	755	0.00	-0.07	0.08	0.900	755	0.00	-0.10	0.10	0.982
Total cholesterol to total lipids ratio in small VLDL (%)	755	0.02	-0.07	0.11	0.725	755	0.01	-0.09	0.10	0.913	755	0.07	-0.02	0.16	0.127
Cholesterol esters to total lipids ratio in small VLDL (%)	755	0.01	-0.08	0.10	0.783	755	0.01	-0.08	0.09	0.870	755	0.06	-0.03	0.15	0.217
Free cholesterol to total lipids ratio in small VLDL (%)	755	0.03	-0.07	0.12	0.599	755	-0.01	-0.10	0.08	0.766	755	0.10	0.01	0.20	0.036
Triglycerides to total lipids ratio in small VLDL (%)	755	-0.01	-0.10	0.08	0.835	755	-0.01	-0.10	0.08	0.886	755	-0.07	-0.16	0.02	0.152
Phospholipids to total lipds ratio in very small VLDL (%)	755	0.04	-0.05	0.13	0.384	755	-0.01	-0.09	0.08	0.880	755	0.09	0.01	0.16	0.025
Total cholesterol to total lipids ratio in very small VLDL (%)	755	-0.06	-0.14	0.02	0.149	755	-0.02	-0.10	0.06	0.680	755	-0.07	-0.15	0.02	0.120
Cholesterol esters to total lipids ratio in very small VLDL (%)	755	-0.04	-0.12	0.04	0.354	755	0.02	-0.06	0.09	0.657	755	-0.05	-0.13	0.03	0.201
Free cholesterol to total lipids ratio in very small VLDL (%)	755	-0.07	-0.15	0.02	0.109	755	-0.08	-0.17	0.01	0.076	755	-0.06	-0.17	0.05	0.289
Triglycerides to total lipids ratio in very small VLDL (%)	755	0.04	-0.04	0.12	0.356	755	0.02	-0.06	0.11	0.572	755	0.02	-0.07	0.10	0.692
Phospholipids to total lipds ratio in IDL (%)	755	-0.02	-0.10	0.07	0.667	755	0.02	-0.05	0.10	0.541	755	-0.03	-0.13	0.08	0.637
Total cholesterol to total lipids ratio in IDL (%)	755	-0.03	-0.12	0.05	0.456	755	-0.02	-0.10	0.07	0.712	755	0.01	-0.08	0.11	0.787
Cholesterol esters to total lipids ratio in IDL (%)	755	-0.03	-0.11	0.06	0.533	755	-0.01	-0.10	0.07	0.740	755	0.01	-0.10	0.11	0.913
Free cholesterol to total lipids ratio in IDL (%)	755	-0.01	-0.10	0.07	0.778	755	0.00	-0.09	0.08	0.925	755	0.02	-0.07	0.10	0.698
Triglycerides to total lipids ratio in IDL (%)	755	0.05	-0.03	0.14	0.245	755	0.01	-0.08	0.09	0.851	755	0.00	-0.10	0.09	0.938
Phospholipids to total lipds ratio in large LDL (%)	755	-0.05	-0.14	0.04	0.248	755	0.00	-0.08	0.08	0.921	755	-0.09	-0.16	-0.02	0.009

S8 Table Interactions between current physical activity (measures at age 15y) with historical physical activity (mean of measures at age 12y and 14y) in relation to metabolic traits at age 15y in ALSPAC

Models adjusted for historical measure, age, age*historical measure, sex, sex*historical measure, ethnicity, ethnicity*historical measure, education, education*historical measure, smoking, smoking*historical measure, alcohol, alcohol*historical measure, wear time, wear time*historical measure, wear month, wear month*historical measure

	Interaction of CPM at age 15y with historical CPM (mean of CPM at 12y, 14y)					Interaction of MVPA at age 15y with historical MVPA (mean of MVPA at 12y, 14y)					Interaction of SED at age 15y with historical SED (mean of SED at 12y, 14y)				
**Standardised outcome at age 15y**	**N**	**Beta**	**LCI**	**UCI**	**P-value**	**N**	**Beta**	**LCI**	**UCI**	**P-value**	**N**	**Beta**	**LCI**	**UCI**	**P-value**
Total cholesterol to total lipids ratio in large LDL (%)	755	0.02	-0.07	0.11	0.738	755	0.01	-0.08	0.09	0.885	755	0.07	-0.01	0.16	0.078
Cholesterol esters to total lipids ratio in large LDL (%)	755	0.03	-0.06	0.13	0.480	755	0.01	-0.08	0.09	0.838	755	0.09	0.01	0.18	0.030
Free cholesterol to total lipids ratio in large LDL (%)	755	-0.06	-0.14	0.02	0.134	755	-0.01	-0.08	0.06	0.768	755	-0.09	-0.17	0.00	0.039
Triglycerides to total lipids ratio in large LDL (%)	755	0.03	-0.05	0.12	0.409	755	-0.01	-0.09	0.08	0.881	755	-0.02	-0.12	0.08	0.727
Phospholipids to total lipds ratio in medium LDL (%)	755	-0.02	-0.05	0.01	0.255	755	-0.01	-0.04	0.02	0.667	755	-0.03	-0.06	-0.01	0.017
Total cholesterol to total lipids ratio in medium LDL (%)	755	0.03	-0.07	0.12	0.576	755	0.01	-0.07	0.10	0.754	755	0.08	0.00	0.17	0.063
Cholesterol esters to total lipids ratio in medium LDL (%)	755	0.05	-0.05	0.14	0.343	755	0.02	-0.07	0.11	0.701	755	0.10	0.01	0.18	0.028
Free cholesterol to total lipids ratio in medium LDL (%)	755	-0.02	-0.04	0.01	0.164	755	-0.01	-0.03	0.02	0.658	755	-0.03	-0.05	0.00	0.017
Triglycerides to total lipids ratio in medium LDL (%)	755	0.05	-0.03	0.13	0.199	755	0.01	-0.07	0.09	0.854	755	0.02	-0.08	0.11	0.709
Phospholipids to total lipds ratio in small LDL (%)	755	-0.03	-0.09	0.02	0.215	755	-0.01	-0.06	0.04	0.629	755	-0.06	-0.11	-0.01	0.011
Total cholesterol to total lipids ratio in small LDL (%)	755	0.03	-0.06	0.12	0.511	755	0.01	-0.07	0.10	0.772	755	0.09	0.00	0.17	0.044
Cholesterol esters to total lipids ratio in small LDL (%)	755	0.05	-0.05	0.14	0.317	755	0.02	-0.07	0.11	0.666	755	0.10	0.01	0.19	0.022
Free cholesterol to total lipids ratio in small LDL (%)	755	-0.04	-0.08	0.01	0.146	755	-0.01	-0.06	0.03	0.540	755	-0.05	-0.10	-0.01	0.023
Triglycerides to total lipids ratio in small LDL (%)	755	0.07	-0.01	0.14	0.096	755	0.02	-0.06	0.10	0.634	755	0.04	-0.05	0.13	0.409
Phospholipids to total lipds ratio in very large HDL (%)	755	-0.04	-0.13	0.05	0.396	755	-0.06	-0.14	0.02	0.149	755	-0.03	-0.13	0.06	0.488
Total cholesterol to total lipids ratio in very large HDL (%)	755	0.03	-0.06	0.12	0.565	755	0.06	-0.02	0.14	0.124	755	0.02	-0.06	0.11	0.579
Cholesterol esters to total lipids ratio in very large HDL (%)	755	0.03	-0.06	0.12	0.544	755	0.06	-0.02	0.14	0.138	755	0.02	-0.06	0.11	0.610
Free cholesterol to total lipids ratio in very large HDL (%)	755	-0.03	-0.11	0.05	0.500	755	-0.01	-0.09	0.07	0.827	755	0.01	-0.08	0.10	0.777
Triglycerides to total lipids ratio in very large HDL (%)	755	0.08	-0.02	0.19	0.114	755	0.00	-0.08	0.07	0.912	755	0.05	-0.06	0.17	0.367
Phospholipids to total lipds ratio in large HDL (%)	755	0.06	-0.03	0.16	0.160	755	0.05	-0.03	0.12	0.202	755	0.13	0.03	0.24	0.011
Total cholesterol to total lipids ratio in large HDL (%)	755	-0.08	-0.18	0.02	0.137	755	-0.04	-0.12	0.03	0.258	755	-0.12	-0.23	-0.01	0.032
Cholesterol esters to total lipids ratio in large HDL (%)	755	-0.08	-0.18	0.02	0.103	755	-0.05	-0.12	0.03	0.222	755	-0.13	-0.25	-0.02	0.023
Free cholesterol to total lipids ratio in large HDL (%)	755	-0.04	-0.13	0.06	0.466	755	-0.03	-0.11	0.06	0.522	755	-0.06	-0.15	0.04	0.217
Triglycerides to total lipids ratio in large HDL (%)	755	0.08	-0.04	0.19	0.181	755	0.02	-0.06	0.10	0.591	755	0.06	-0.05	0.18	0.286
Phospholipids to total lipds ratio in medium HDL (%)	755	0.00	-0.09	0.10	0.974	755	-0.05	-0.13	0.04	0.316	755	0.01	-0.10	0.12	0.909
Total cholesterol to total lipids ratio in medium HDL (%)	755	-0.04	-0.13	0.05	0.420	755	0.03	-0.06	0.11	0.522	755	-0.05	-0.16	0.05	0.295
Cholesterol esters to total lipids ratio in medium HDL (%)	755	-0.05	-0.14	0.04	0.283	755	0.03	-0.05	0.11	0.497	755	-0.06	-0.16	0.05	0.278
Free cholesterol to total lipids ratio in medium HDL (%)	755	0.05	-0.04	0.14	0.274	755	0.00	-0.08	0.09	0.935	755	0.00	-0.11	0.11	0.977
Triglycerides to total lipids ratio in medium HDL (%)	755	0.06	-0.03	0.16	0.208	755	0.01	-0.07	0.10	0.743	755	0.09	-0.02	0.19	0.113
Phospholipids to total lipds ratio in small HDL (%)	755	-0.02	-0.10	0.06	0.606	755	0.03	-0.05	0.10	0.454	755	-0.04	-0.12	0.04	0.338
Total cholesterol to total lipids ratio in small HDL (%)	755	0.00	-0.08	0.08	0.966	755	-0.03	-0.11	0.04	0.410	755	0.03	-0.05	0.11	0.517
Cholesterol esters to total lipids ratio in small HDL (%)	755	0.01	-0.06	0.09	0.731	755	-0.03	-0.10	0.05	0.504	755	0.04	-0.04	0.12	0.340
Free cholesterol to total lipids ratio in small HDL (%)	755	-0.09	-0.17	-0.01	0.031	755	-0.04	-0.11	0.04	0.328	755	-0.10	-0.21	0.00	0.053
Triglycerides to total lipids ratio in small HDL (%)	755	0.08	-0.01	0.16	0.083	755	0.02	-0.05	0.10	0.535	755	0.04	-0.06	0.14	0.418
Mean diameter for VLDL particles (nm)	755	0.01	-0.08	0.10	0.777	755	0.01	-0.08	0.10	0.836	755	0.00	-0.10	0.10	0.977
Mean diameter for LDL particles (nm)	755	-0.01	-0.09	0.07	0.768	755	-0.03	-0.11	0.04	0.394	755	-0.07	-0.16	0.01	0.087
Mean diameter for HDL particles (nm)	755	-0.05	-0.15	0.05	0.349	755	-0.06	-0.15	0.04	0.238	755	-0.05	-0.15	0.04	0.250
Serum total cholesterol (mmol/l)	755	0.04	-0.05	0.13	0.403	755	-0.02	-0.10	0.06	0.643	755	0.10	0.02	0.18	0.015
Total cholesterol in VLDL (mmol/l)	755	0.06	-0.03	0.15	0.171	755	0.00	-0.07	0.07	0.927	755	0.09	-0.01	0.20	0.085
Remnant cholesterol (non-HDL, non-LDL -cholesterol) (mmol/l)	755	0.06	-0.02	0.15	0.160	755	0.00	-0.08	0.07	0.902	755	0.11	0.02	0.21	0.022
Total cholesterol in LDL (mmol/l)	755	0.05	-0.03	0.14	0.212	755	0.00	-0.08	0.08	0.968	755	0.12	0.03	0.21	0.007
Total cholesterol in HDL (mmol/l)	755	-0.04	-0.14	0.05	0.375	755	-0.05	-0.14	0.04	0.291	755	-0.02	-0.11	0.07	0.684
Total cholesterol in HDL2 (mmol/l)	755	-0.05	-0.14	0.05	0.345	755	-0.04	-0.13	0.05	0.363	755	-0.03	-0.12	0.07	0.571
Total cholesterol in HDL3 (mmol/l)	755	-0.04	-0.13	0.06	0.455	755	-0.06	-0.14	0.03	0.201	755	0.00	-0.09	0.08	0.950
Esterified cholesterol (mmol/l)	755	0.03	-0.05	0.12	0.437	755	-0.01	-0.09	0.07	0.767	755	0.09	0.01	0.18	0.029
Free cholesterol (mmol/l)	755	0.04	-0.05	0.13	0.349	755	-0.03	-0.11	0.05	0.411	755	0.12	0.04	0.20	0.004
Serum total triglycerides (mmol/l)	755	0.06	-0.02	0.14	0.147	755	0.02	-0.05	0.09	0.551	755	0.06	-0.04	0.17	0.244

S8 Table Interactions between current physical activity (measures at age 15y) with historical physical activity (mean of measures at age 12y and 14y) in relation to metabolic traits at age 15y in ALSPAC

Models adjusted for historical measure, age, age*historical measure, sex, sex*historical measure, ethnicity, ethnicity*historical measure, education, education*historical measure, smoking, smoking*historical measure, alcohol, alcohol*historical measure, wear time, wear time*historical measure, wear month, wear month*historical measure

	Interaction of CPM at age 15y with historical CPM (mean of CPM at 12y, 14y)					Interaction of MVPA at age 15y with historical MVPA (mean of MVPA at 12y, 14y)					Interaction of SED at age 15y with historical SED (mean of SED at 12y, 14y)				
**Standardised outcome at age 15y**	**N**	**Beta**	**LCI**	**UCI**	**P-value**	**N**	**Beta**	**LCI**	**UCI**	**P-value**	**N**	**Beta**	**LCI**	**UCI**	**P-value**
Triglycerides in VLDL (mmol/l)	755	0.05	-0.04	0.13	0.257	755	0.02	-0.05	0.10	0.524	755	0.05	-0.06	0.15	0.391
Triglycerides in LDL (mmol/l)	755	0.07	0.00	0.15	0.064	755	0.01	-0.06	0.09	0.713	755	0.09	0.00	0.18	0.048
Triglycerides in HDL (mmol/l)	755	0.04	-0.05	0.12	0.384	755	-0.02	-0.09	0.05	0.512	755	0.05	-0.05	0.14	0.337
Diacylglycerol (mmol/l)	755	0.00	-0.08	0.08	0.975	755	-0.04	-0.12	0.04	0.326	755	0.02	-0.08	0.12	0.712
Ratio of diacylglycerol to triglycerides	755	-0.02	-0.10	0.06	0.655	755	-0.05	-0.14	0.05	0.342	755	0.00	-0.10	0.10	0.969
Total phosphoglycerides (mmol/l)	755	-0.03	-0.11	0.06	0.535	755	-0.04	-0.12	0.03	0.261	755	0.05	-0.04	0.13	0.270
Ratio of triglycerides to phosphoglycerides	755	0.05	-0.03	0.14	0.222	755	0.01	-0.07	0.08	0.843	755	0.03	-0.07	0.13	0.609
Phosphatidylcholine and other cholines (mmol/l)	755	0.01	-0.07	0.08	0.891	755	-0.06	-0.13	0.01	0.100	755	0.07	-0.01	0.15	0.108
Total cholines (mmol/l)	755	0.00	-0.08	0.08	0.994	755	-0.04	-0.12	0.03	0.278	755	0.07	-0.01	0.15	0.107
Apolipoprotein A-I (g/l)	755	-0.02	-0.11	0.07	0.688	755	-0.04	-0.13	0.05	0.357	755	0.02	-0.07	0.10	0.708
Apolipoprotein B (g/l)	755	0.07	-0.01	0.16	0.086	755	0.01	-0.06	0.08	0.797	755	0.12	0.02	0.22	0.021
Ratio of apolipoprotein B to apolipoprotein A-I	755	0.09	0.00	0.18	0.056	755	0.03	-0.04	0.11	0.415	755	0.12	0.01	0.22	0.034
Total fatty acids (mmol/l)	755	0.02	-0.05	0.10	0.554	755	-0.02	-0.09	0.05	0.568	755	0.05	-0.03	0.14	0.226
Estimated description of fatty acid chain length, not actual carbon number	755	0.07	-0.02	0.15	0.121	755	0.03	-0.06	0.12	0.571	755	0.07	-0.01	0.16	0.075
Estimated degree of unsaturation	755	0.01	-0.07	0.10	0.773	755	-0.01	-0.09	0.07	0.755	755	0.05	-0.04	0.14	0.243
22:6, docosahexaenoic acid (mmol/l)	755	0.09	0.00	0.18	0.049	755	0.05	-0.04	0.14	0.308	755	0.04	-0.06	0.14	0.384
18:2, linoleic acid (mmol/l)	755	0.03	-0.05	0.10	0.525	755	-0.01	-0.08	0.06	0.739	755	0.04	-0.04	0.12	0.290
Conjugated linoleic acid (mmol/l)	755	0.03	-0.05	0.11	0.477	755	0.01	-0.07	0.08	0.872	755	0.07	-0.03	0.16	0.150
Omega-3 fatty acids (mmol/l)	755	0.07	-0.02	0.15	0.143	755	0.05	-0.04	0.14	0.284	755	0.06	-0.04	0.15	0.239
Omega-6 fatty acids (mmol/l)	755	0.01	-0.07	0.09	0.789	755	-0.03	-0.10	0.04	0.443	755	0.05	-0.03	0.13	0.201
Polyunsaturated fatty acids (mmol/l)	755	0.02	-0.06	0.10	0.635	755	-0.02	-0.09	0.05	0.609	755	0.05	-0.03	0.14	0.179
Monounsaturated fatty acids; 16:1, 18:1 (mmol/l)	755	0.06	-0.02	0.14	0.134	755	0.00	-0.07	0.07	0.997	755	0.10	0.00	0.19	0.052
Saturated fatty acids (mmol/l)	755	-0.01	-0.09	0.07	0.795	755	-0.03	-0.11	0.04	0.365	755	0.00	-0.09	0.09	0.926
Ratio of 22:6 docosahexaenoic acid to total fatty acids (%)	755	0.10	0.00	0.20	0.047	755	0.08	-0.02	0.18	0.118	755	0.01	-0.08	0.11	0.774
Ratio of 18:2 linoleic acid to total fatty acids (%)	755	0.01	-0.07	0.10	0.750	755	0.03	-0.06	0.12	0.520	755	-0.02	-0.11	0.07	0.727
Ratio of conjugated linoleic acid to total fatty acids (%)	755	0.03	-0.05	0.11	0.480	755	0.01	-0.06	0.08	0.823	755	0.07	-0.03	0.17	0.158
Ratio of omega-3 fatty acids to total fatty acids (%)	755	0.07	-0.02	0.16	0.152	755	0.09	-0.01	0.19	0.086	755	0.02	-0.07	0.12	0.641
Ratio of omega-6 fatty acids to total fatty acids (%)	755	-0.02	-0.11	0.06	0.609	755	0.00	-0.09	0.09	0.967	755	-0.01	-0.10	0.08	0.798
Ratio of polyunsaturated fatty acids to total fatty acids (%)	755	-0.01	-0.09	0.08	0.886	755	0.02	-0.07	0.11	0.626	755	-0.01	-0.10	0.08	0.891
Ratio of monounsaturated fatty acids to total fatty acids (%)	755	0.08	-0.01	0.18	0.093	755	0.02	-0.08	0.11	0.709	755	0.11	0.02	0.21	0.023
Ratio of saturated fatty acids to total fatty acids (%)	755	-0.10	-0.20	0.00	0.041	755	-0.05	-0.13	0.03	0.244	755	-0.14	-0.23	-0.04	0.005
Insulin (mu/l)	755	-0.01	-0.07	0.05	0.680	755	0.02	-0.05	0.08	0.576	755	-0.04	-0.10	0.02	0.183
Glucose (mmol/l)	755	-0.02	-0.09	0.06	0.691	755	-0.02	-0.09	0.06	0.677	755	0.01	-0.09	0.11	0.907
Lactate (mmol/l)	755	0.04	-0.06	0.13	0.464	755	0.03	-0.06	0.13	0.472	755	-0.02	-0.12	0.08	0.713
Pyruvate (mmol/l)	755	0.00	-0.09	0.10	0.959	755	-0.01	-0.10	0.09	0.887	755	0.02	-0.07	0.11	0.683
Citrate (mmol/l)	755	0.11	0.02	0.20	0.013	755	0.06	-0.02	0.15	0.155	755	0.10	-0.01	0.21	0.074
Alanine (mmol/l)	755	-0.02	-0.11	0.06	0.586	755	-0.03	-0.12	0.06	0.546	755	0.03	-0.07	0.12	0.551
Glutamine (mmol/l)	755	0.05	-0.03	0.13	0.206	755	0.06	-0.01	0.13	0.095	755	0.01	-0.07	0.10	0.751
Histidine (mmol/l)	755	-0.01	-0.11	0.08	0.762	755	-0.02	-0.12	0.07	0.618	755	-0.02	-0.10	0.06	0.641
Isoleucine (mmol/l)	755	-0.11	-0.19	-0.03	0.008	755	-0.06	-0.13	0.02	0.148	755	-0.03	-0.12	0.06	0.532
Leucine (mmol/l)	755	-0.09	-0.16	-0.01	0.026	755	-0.03	-0.11	0.04	0.381	755	-0.01	-0.09	0.07	0.745
Valine (mmol/l)	755	-0.10	-0.19	-0.02	0.012	755	-0.02	-0.10	0.05	0.514	755	0.00	-0.08	0.09	0.928
Phenylalanine (mmol/l)	755	-0.07	-0.15	0.02	0.144	755	-0.02	-0.11	0.06	0.584	755	0.01	-0.11	0.12	0.920
Tyrosine (mmol/l)	755	0.00	-0.10	0.10	0.989	755	0.01	-0.10	0.11	0.894	755	0.00	-0.11	0.11	0.979
Acetate (mmol/l)	755	-0.01	-0.08	0.06	0.797	755	0.05	-0.03	0.12	0.245	755	-0.04	-0.13	0.06	0.476
Acetoacetate (mmol/l)	755	0.00	-0.08	0.08	0.945	755	0.03	-0.05	0.10	0.486	755	-0.02	-0.12	0.07	0.646
3-hydroxybutyrate (mmol/l)	755	0.07	-0.01	0.16	0.071	755	0.07	-0.01	0.16	0.086	755	0.06	-0.05	0.17	0.257

S8 Table Interactions between current physical activity (measures at age 15y) with historical physical activity (mean of measures at age 12y and 14y) in relation to metabolic traits at age 15y in ALSPAC

Models adjusted for historical measure, age, age*historical measure, sex, sex*historical measure, ethnicity, ethnicity*historical measure, education, education*historical measure, smoking, smoking*historical measure, alcohol, alcohol*historical measure, wear time, wear time*historical measure, wear month, wear month*historical measure

	Interaction of CPM at age 15y with historical CPM (mean of CPM at 12y, 14y)					Interaction of MVPA at age 15y with historical MVPA (mean of MVPA at 12y, 14y)					Interaction of SED at age 15y with historical SED (mean of SED at 12y, 14y)				
**Standardised outcome at age 15y**	**N**	**Beta**	**LCI**	**UCI**	**P-value**	**N**	**Beta**	**LCI**	**UCI**	**P-value**	**N**	**Beta**	**LCI**	**UCI**	**P-value**
Creatinine (mmol/l)	755	-0.03	-0.11	0.05	0.462	755	0.02	-0.06	0.10	0.647	755	-0.09	-0.18	0.00	0.046
Albumin (signal area)	755	0.04	-0.05	0.12	0.429	755	0.04	-0.04	0.13	0.308	755	0.02	-0.09	0.12	0.758
Glycoprotein acetyls, mainly a1-acid glycoprotein (mmol/l)	755	0.01	-0.07	0.09	0.842	755	-0.01	-0.09	0.07	0.873	755	0.00	-0.10	0.09	0.994
C-reactive protein (mg/l)	755	0.02	-0.03	0.07	0.371	755	-0.01	-0.05	0.03	0.603	755	0.06	-0.08	0.19	0.404
