# Supplementary material for: Associations of device-measured physical activity across adolescence with metabolic traits: Prospective cohort study
Source: PLoS Med. 2018 Sep 11;15(9):e1002649. doi: 10.1371/journal.pmed.1002649 (PMC6133272; doi:10.1371/journal.pmed.1002649)
Supplement: S9 Table — ALSPAC, Avon Longitudinal Study of Parents and Children; CPM, counts per minute. (PDF) [file pmed.1002649.s009.pdf]

S9 Table Associations of change in total physical activity (CPM change from age 12y-15y) with metabolic traits at age 15y in ALSPAC

	**Change in CPM from age 12y-15y (per SD-unit increase)**									
	Adj. for age, sex, ethnicity, maternal education change in wear time, wear month					*Additionally adj. for change in FMI*				
**Standardised outcome at age 15y**	**N**	**Beta**	**LCL**	**UCL**	**P-value**	**N**	**Beta**	**LCL**	**UCL**	**P-value**
Systolic blood pressure (mmHg)	1599	0.02	-0.03	0.06	0.492	1553	0.01	-0.04	0.06	0.622
Diastolic blood pressure (mmHg)	1599	0.04	-0.01	0.09	0.101	1553	0.03	-0.02	0.08	0.198
Concentration of chylomicrons and extremely large VLDL particles (mol/l)	1076	-0.05	-0.10	0.00	0.054	1054	-0.03	-0.08	0.02	0.233
Total lipids in chylomicrons and extremely large VLDL (mmol/l)	1076	-0.05	-0.10	0.00	0.056	1054	-0.03	-0.08	0.02	0.229
Phospholipids in chylomicrons and extremely large VLDL (mmol/l)	1076	-0.05	-0.10	0.00	0.047	1054	-0.03	-0.08	0.02	0.198
Total cholesterol in chylomicrons and extremely large VLDL (mmol/l)	1076	-0.04	-0.10	0.01	0.089	1054	-0.03	-0.08	0.03	0.339
Cholesterol esters in chylomicrons and extremely large VLDL (mmol/l)	1076	-0.04	-0.09	0.01	0.157	1054	-0.02	-0.07	0.04	0.515
Free cholesterol in chylomicrons and extremely large VLDL (mmol/l)	1076	-0.05	-0.10	0.00	0.052	1054	-0.03	-0.08	0.02	0.208
Triglycerides in chylomicrons and extremely large VLDL (mmol/l)	1076	-0.05	-0.10	0.00	0.052	1054	-0.03	-0.08	0.02	0.214
Concentration of very large VLDL particles (mol/l)	1076	-0.05	-0.10	0.00	0.043	1054	-0.03	-0.08	0.02	0.191
Total lipids in very large VLDL (mmol/l)	1076	-0.05	-0.10	0.00	0.046	1054	-0.03	-0.08	0.02	0.198
Phospholipids in very large VLDL (mmol/l)	1076	-0.05	-0.10	0.00	0.043	1054	-0.03	-0.08	0.02	0.188
Total cholesterol in very large VLDL (mmol/l)	1076	-0.05	-0.10	0.00	0.055	1054	-0.03	-0.08	0.02	0.234
Cholesterol esters in very large VLDL (mmol/l)	1076	-0.05	-0.10	0.00	0.061	1054	-0.03	-0.08	0.02	0.255
Free cholesterol in very large VLDL (mmol/l)	1076	-0.05	-0.10	0.00	0.050	1054	-0.03	-0.08	0.02	0.216
Triglycerides in very large VLDL (mmol/l)	1076	-0.05	-0.10	0.00	0.046	1054	-0.03	-0.08	0.02	0.193
Concentration of large VLDL particles (mol/l)	1076	-0.05	-0.10	0.00	0.057	1054	-0.03	-0.08	0.02	0.234
Total lipids in large VLDL (mmol/l)	1076	-0.05	-0.10	0.00	0.056	1054	-0.03	-0.09	0.02	0.228
Phospholipids in large VLDL (mmol/l)	1076	-0.05	-0.10	0.00	0.053	1054	-0.03	-0.09	0.02	0.221
Total cholesterol in large VLDL (mmol/l)	1076	-0.05	-0.11	0.00	0.051	1054	-0.03	-0.09	0.02	0.218
Cholesterol esters in large VLDL (mmol/l)	1076	-0.05	-0.11	0.00	0.062	1054	-0.03	-0.09	0.02	0.247
Free cholesterol in large VLDL (mmol/l)	1076	-0.05	-0.11	0.00	0.044	1054	-0.03	-0.09	0.02	0.196
Triglycerides in large VLDL (mmol/l)	1076	-0.05	-0.10	0.00	0.060	1054	-0.03	-0.08	0.02	0.236
Concentration of medium VLDL particles (mol/l)	1076	-0.06	-0.11	0.00	0.042	1054	-0.04	-0.09	0.02	0.185
Total lipids in medium VLDL (mmol/l)	1076	-0.06	-0.11	0.00	0.044	1054	-0.04	-0.09	0.02	0.190
Phospholipids in medium VLDL (mmol/l)	1076	-0.06	-0.11	0.00	0.035	1054	-0.04	-0.09	0.02	0.164
Total cholesterol in medium VLDL (mmol/l)	1076	-0.06	-0.11	0.00	0.045	1054	-0.04	-0.09	0.02	0.194
Cholesterol esters in medium VLDL (mmol/l)	1076	-0.05	-0.11	0.00	0.065	1054	-0.03	-0.09	0.02	0.239
Free cholesterol in medium VLDL (mmol/l)	1076	-0.06	-0.11	0.00	0.036	1054	-0.04	-0.09	0.02	0.168
Triglycerides in medium VLDL (mmol/l)	1076	-0.05	-0.11	0.00	0.053	1054	-0.03	-0.09	0.02	0.211
Concentration of small VLDL particles (mol/l)	1076	-0.06	-0.11	0.00	0.042	1054	-0.04	-0.10	0.02	0.192
Total lipids in small VLDL (mmol/l)	1076	-0.06	-0.12	0.00	0.044	1054	-0.04	-0.09	0.02	0.205
Phospholipids in small VLDL (mmol/l)	1076	-0.05	-0.11	0.00	0.065	1054	-0.03	-0.09	0.03	0.268
Total cholesterol in small VLDL (mmol/l)	1076	-0.06	-0.11	0.00	0.054	1054	-0.04	-0.09	0.02	0.236
Cholesterol esters in small VLDL (mmol/l)	1076	-0.05	-0.11	0.01	0.076	1054	-0.03	-0.09	0.03	0.277
Free cholesterol in small VLDL (mmol/l)	1076	-0.06	-0.12	0.00	0.045	1054	-0.04	-0.10	0.02	0.217
Triglycerides in small VLDL (mmol/l)	1076	-0.05	-0.11	0.00	0.057	1054	-0.04	-0.09	0.02	0.217
Concentration of very small VLDL particles (mol/l)	1076	-0.04	-0.10	0.01	0.134	1054	-0.03	-0.09	0.03	0.389
Total lipids in very small VLDL (mmol/l)	1076	-0.05	-0.10	0.01	0.107	1054	-0.03	-0.09	0.03	0.343
Phospholipids in very small VLDL (mmol/l)	1076	-0.04	-0.09	0.02	0.207	1054	-0.02	-0.08	0.04	0.478
Total cholesterol in very small VLDL (mmol/l)	1076	-0.04	-0.10	0.02	0.156	1054	-0.02	-0.08	0.03	0.413
Cholesterol esters in very small VLDL (mmol/l)	1076	-0.04	-0.10	0.02	0.183	1054	-0.02	-0.08	0.04	0.449
Free cholesterol in very small VLDL (mmol/l)	1076	-0.04	-0.09	0.01	0.136	1054	-0.03	-0.08	0.03	0.370
Triglycerides in very small VLDL (mmol/l)	1076	-0.05	-0.11	0.01	0.111	1054	-0.03	-0.09	0.03	0.312
Concentration of IDL particles (mol/l)	1076	-0.03	-0.09	0.02	0.240	1054	-0.02	-0.08	0.04	0.488
Total lipids in IDL (mmol/l)	1076	-0.03	-0.09	0.02	0.267	1054	-0.02	-0.08	0.04	0.531
Phospholipids in IDL (mmol/l)	1076	-0.03	-0.08	0.03	0.375	1054	-0.01	-0.07	0.04	0.636
Total cholesterol in IDL (mmol/l)	1076	-0.03	-0.09	0.02	0.244	1054	-0.02	-0.08	0.04	0.509
Cholesterol esters in IDL (mmol/l)	1076	-0.04	-0.09	0.02	0.194	1054	-0.02	-0.08	0.04	0.453
Free cholesterol in IDL (mmol/l)	1076	-0.02	-0.08	0.03	0.419	1054	-0.01	-0.07	0.05	0.669
Triglycerides in IDL (mmol/l)	1076	-0.03	-0.09	0.04	0.415	1054	-0.02	-0.08	0.05	0.626
Concentration of large LDL particles (mol/l)	1076	-0.03	-0.08	0.03	0.345	1054	-0.01	-0.07	0.04	0.647
Total lipids in large LDL (mmol/l)	1076	-0.03	-0.08	0.03	0.363	1054	-0.01	-0.07	0.05	0.669
Phospholipids in large LDL (mmol/l)	1076	-0.03	-0.08	0.03	0.376	1054	-0.01	-0.07	0.05	0.697
Total cholesterol in large LDL (mmol/l)	1076	-0.03	-0.08	0.03	0.363	1054	-0.01	-0.07	0.05	0.672
Cholesterol esters in large LDL (mmol/l)	1076	-0.03	-0.08	0.03	0.334	1054	-0.01	-0.07	0.04	0.649
Free cholesterol in large LDL (mmol/l)	1076	-0.02	-0.08	0.04	0.466	1054	-0.01	-0.07	0.05	0.746
Triglycerides in large LDL (mmol/l)	1076	-0.02	-0.08	0.04	0.513	1054	-0.01	-0.08	0.05	0.707
Concentration of medium LDL particles (mol/l)	1076	-0.03	-0.08	0.03	0.314	1054	-0.01	-0.07	0.04	0.627
Total lipids in medium LDL (mmol/l)	1076	-0.03	-0.09	0.03	0.325	1054	-0.01	-0.07	0.04	0.643
Phospholipids in medium LDL (mmol/l)	1076	-0.03	-0.09	0.03	0.288	1054	-0.01	-0.07	0.04	0.638
Total cholesterol in medium LDL (mmol/l)	1076	-0.03	-0.09	0.03	0.313	1054	-0.01	-0.07	0.04	0.620
Cholesterol esters in medium LDL (mmol/l)	1076	-0.03	-0.09	0.03	0.313	1054	-0.01	-0.07	0.04	0.616
Free cholesterol in medium LDL (mmol/l)	1076	-0.03	-0.09	0.03	0.316	1054	-0.01	-0.07	0.04	0.630
Triglycerides in medium LDL (mmol/l)	1076	-0.01	-0.07	0.05	0.785	1054	0.00	-0.06	0.06	0.975
Concentration of small LDL particles (mol/l)	1076	-0.03	-0.09	0.02	0.255	1054	-0.02	-0.07	0.04	0.569
Total lipids in small LDL (mmol/l)	1076	-0.03	-0.09	0.03	0.301	1054	-0.01	-0.07	0.04	0.629
Phospholipids in small LDL (mmol/l)	1076	-0.03	-0.09	0.02	0.237	1054	-0.02	-0.07	0.04	0.577
Total cholesterol in small LDL (mmol/l)	1076	-0.03	-0.08	0.03	0.336	1054	-0.01	-0.07	0.04	0.657
Cholesterol esters in small LDL (mmol/l)	1076	-0.03	-0.08	0.03	0.364	1054	-0.01	-0.07	0.05	0.678
Free cholesterol in small LDL (mmol/l)	1076	-0.03	-0.09	0.02	0.260	1054	-0.02	-0.07	0.04	0.591
Triglycerides in small LDL (mmol/l)	1076	-0.03	-0.08	0.03	0.389	1054	-0.01	-0.07	0.05	0.679
Concentration of very large HDL particles (mol/l)	1076	0.02	-0.04	0.08	0.581	1054	0.01	-0.05	0.07	0.688
Total lipids in very large HDL (mmol/l)	1076	0.01	-0.05	0.07	0.664	1054	0.01	-0.05	0.07	0.745
Phospholipids in very large HDL (mmol/l)	1076	0.02	-0.04	0.08	0.457	1054	0.02	-0.04	0.08	0.611
Total cholesterol in very large HDL (mmol/l)	1076	0.01	-0.06	0.07	0.863	1054	0.01	-0.06	0.07	0.847
Cholesterol esters in very large HDL (mmol/l)	1076	0.00	-0.06	0.06	0.953	1054	0.00	-0.06	0.07	0.893
Free cholesterol in very large HDL (mmol/l)	1076	0.01	-0.05	0.07	0.651	1054	0.01	-0.05	0.07	0.738
Triglycerides in very large HDL (mmol/l)	1076	-0.05	-0.10	0.01	0.112	1054	-0.04	-0.10	0.02	0.192
Concentration of large HDL particles (mol/l)	1076	0.02	-0.04	0.08	0.461	1054	0.01	-0.05	0.07	0.632
Total lipids in large HDL (mmol/l)	1076	0.02	-0.04	0.08	0.430	1054	0.02	-0.04	0.08	0.617
Phospholipids in large HDL (mmol/l)	1076	0.02	-0.04	0.08	0.473	1054	0.01	-0.05	0.07	0.637
Total cholesterol in large HDL (mmol/l)	1076	0.03	-0.03	0.09	0.363	1054	0.02	-0.04	0.08	0.563
Cholesterol esters in large HDL (mmol/l)	1076	0.03	-0.03	0.09	0.355	1054	0.02	-0.04	0.08	0.557
Free cholesterol in large HDL (mmol/l)	1076	0.03	-0.03	0.08	0.399	1054	0.02	-0.04	0.08	0.593
Triglycerides in large HDL (mmol/l)	1076	-0.04	-0.10	0.02	0.183	1054	-0.04	-0.09	0.02	0.234
Concentration of medium HDL particles (mol/l)	1076	0.01	-0.05	0.07	0.764	1054	0.01	-0.05	0.07	0.707
Total lipids in medium HDL (mmol/l)	1076	0.01	-0.05	0.07	0.705	1054	0.01	-0.05	0.07	0.697
Phospholipids in medium HDL (mmol/l)	1076	0.01	-0.05	0.07	0.666	1054	0.01	-0.04	0.07	0.622
Total cholesterol in medium HDL (mmol/l)	1076	0.02	-0.04	0.07	0.597	1054	0.01	-0.05	0.07	0.690
Cholesterol esters in medium HDL (mmol/l)	1076	0.02	-0.04	0.08	0.553	1054	0.01	-0.05	0.07	0.665

S9 Table Associations of change in total physical activity (CPM change from age 12y-15y) with metabolic traits at age 15y in ALSPAC

	Change in CPM from age 12y-15y (per SD-unit increase)									
	Adj. for age, sex, ethnicity, maternal education change in wear time, wear month					*Additionally adj. for change in FMI*				
**Standardised outcome at age 15y**	**N**	**Beta**	**LCL**	**UCL**	**P-value**	**N**	**Beta**	**LCL**	**UCL**	**P-value**
Free cholesterol in medium HDL (mmol/l)	1076	0.01	-0.05	0.06	0.795	1054	0.01	-0.05	0.07	0.788
Triglycerides in medium HDL (mmol/l)	1076	-0.04	-0.10	0.02	0.208	1054	-0.03	-0.09	0.04	0.416
Concentration of small HDL particles (mol/l)	1076	0.00	-0.06	0.06	0.948	1054	0.01	-0.05	0.07	0.847
Total lipids in small HDL (mmol/l)	1076	0.01	-0.05	0.07	0.741	1054	0.02	-0.04	0.08	0.570
Phospholipids in small HDL (mmol/l)	1076	-0.01	-0.07	0.05	0.673	1054	-0.01	-0.07	0.05	0.776
Total cholesterol in small HDL (mmol/l)	1076	0.04	-0.02	0.09	0.206	1054	0.04	-0.01	0.10	0.144
Cholesterol esters in small HDL (mmol/l)	1076	0.04	-0.01	0.10	0.135	1054	0.05	-0.01	0.11	0.090
Free cholesterol in small HDL (mmol/l)	1076	0.00	-0.06	0.06	0.926	1054	0.00	-0.06	0.06	0.995
Triglycerides in small HDL (mmol/l)	1076	-0.03	-0.10	0.03	0.263	1054	-0.02	-0.08	0.04	0.555
Phospholipids to total lipds ratio in chylomicrons and extremely large VLDL (%)	1076	-0.04	-0.09	0.02	0.201	1054	-0.03	-0.09	0.03	0.287
Total cholesterol to total lipids ratio in chylomicrons and extremely large VLDL (%)	1076	0.00	-0.06	0.06	0.976	1054	0.01	-0.05	0.07	0.672
Cholesterol esters to total lipids ratio in chylomicrons and extremely large VLDL (%)	1076	0.01	-0.05	0.07	0.706	1054	0.02	-0.04	0.08	0.442
Free cholesterol to total lipids ratio in chylomicrons and extremely large VLDL (%)	1076	-0.03	-0.09	0.03	0.332	1054	-0.02	-0.08	0.04	0.442
Triglycerides to total lipids ratio in chylomicrons and extremely large VLDL (%)	1076	0.00	-0.05	0.04	0.844	1054	-0.01	-0.06	0.03	0.565
Phospholipids to total lipds ratio in very large VLDL (%)	1076	-0.02	-0.08	0.03	0.425	1054	-0.01	-0.07	0.05	0.774
Total cholesterol to total lipids ratio in very large VLDL (%)	1076	0.05	-0.07	0.16	0.396	1054	0.04	-0.07	0.15	0.439
Cholesterol esters to total lipids ratio in very large VLDL (%)	1076	0.01	-0.06	0.07	0.826	1054	0.00	-0.07	0.06	0.980
Free cholesterol to total lipids ratio in very large VLDL (%)	1076	0.00	-0.06	0.06	0.965	1054	0.00	-0.06	0.06	0.898
Triglycerides to total lipids ratio in very large VLDL (%)	1076	0.00	-0.06	0.06	0.992	1054	0.00	-0.06	0.06	0.984
Phospholipids to total lipds ratio in large VLDL (%)	1076	-0.01	-0.07	0.05	0.789	1054	0.00	-0.06	0.07	0.892
Total cholesterol to total lipids ratio in large VLDL (%)	1076	-0.01	-0.07	0.05	0.749	1054	0.00	-0.07	0.06	0.969
Cholesterol esters to total lipids ratio in large VLDL (%)	1076	0.08	-0.10	0.27	0.380	1054	0.08	-0.09	0.25	0.375
Free cholesterol to total lipids ratio in large VLDL (%)	1076	-0.02	-0.08	0.04	0.514	1054	-0.01	-0.07	0.05	0.841
Triglycerides to total lipids ratio in large VLDL (%)	1076	0.11	-0.10	0.32	0.299	1054	0.10	-0.09	0.29	0.306
Phospholipids to total lipds ratio in medium VLDL (%)	1076	0.00	-0.06	0.06	0.933	1054	0.00	-0.06	0.06	0.892
Total cholesterol to total lipids ratio in medium VLDL (%)	1076	-0.02	-0.08	0.03	0.385	1054	-0.01	-0.07	0.04	0.610
Cholesterol esters to total lipids ratio in medium VLDL (%)	1076	-0.02	-0.07	0.04	0.505	1054	-0.01	-0.07	0.04	0.672
Free cholesterol to total lipids ratio in medium VLDL (%)	1076	-0.03	-0.09	0.03	0.346	1054	-0.01	-0.07	0.05	0.678
Triglycerides to total lipids ratio in medium VLDL (%)	1076	0.02	-0.03	0.08	0.469	1054	0.01	-0.04	0.07	0.699
Phospholipids to total lipds ratio in small VLDL (%)	1076	0.06	0.00	0.11	0.050	1054	0.04	-0.01	0.10	0.122
Total cholesterol to total lipids ratio in small VLDL (%)	1076	-0.01	-0.07	0.05	0.673	1054	-0.01	-0.07	0.05	0.698
Cholesterol esters to total lipids ratio in small VLDL (%)	1076	-0.01	-0.07	0.05	0.641	1054	-0.01	-0.08	0.05	0.650
Free cholesterol to total lipids ratio in small VLDL (%)	1076	0.01	-0.05	0.07	0.688	1054	0.02	-0.04	0.07	0.586
Triglycerides to total lipids ratio in small VLDL (%)	1076	0.00	-0.06	0.05	0.894	1054	0.00	-0.06	0.06	0.966
Phospholipids to total lipds ratio in very small VLDL (%)	1076	0.00	-0.06	0.05	0.888	1054	0.00	-0.06	0.06	0.966
Total cholesterol to total lipids ratio in very small VLDL (%)	1076	0.00	-0.06	0.06	0.954	1054	0.00	-0.06	0.05	0.889
Cholesterol esters to total lipids ratio in very small VLDL (%)	1076	0.00	-0.06	0.06	0.949	1054	0.00	-0.06	0.06	0.899
Free cholesterol to total lipids ratio in very small VLDL (%)	1076	0.00	-0.05	0.04	0.925	1054	0.00	-0.05	0.04	0.850
Triglycerides to total lipids ratio in very small VLDL (%)	1076	0.00	-0.05	0.06	0.956	1054	0.00	-0.05	0.06	0.871
Phospholipids to total lipds ratio in IDL (%)	1076	0.05	0.00	0.10	0.049	1054	0.04	-0.01	0.09	0.156
Total cholesterol to total lipids ratio in IDL (%)	1076	-0.04	-0.09	0.02	0.199	1054	-0.03	-0.09	0.03	0.305
Cholesterol esters to total lipids ratio in IDL (%)	1076	-0.05	-0.10	0.01	0.115	1054	-0.04	-0.09	0.02	0.226
Free cholesterol to total lipids ratio in IDL (%)	1076	0.02	-0.03	0.07	0.480	1054	0.01	-0.04	0.06	0.684
Triglycerides to total lipids ratio in IDL (%)	1076	0.02	-0.04	0.08	0.483	1054	0.02	-0.04	0.08	0.520
Phospholipids to total lipds ratio in large LDL (%)	1076	0.02	-0.03	0.07	0.359	1054	0.01	-0.04	0.07	0.587
Total cholesterol to total lipids ratio in large LDL (%)	1076	-0.03	-0.08	0.02	0.299	1054	-0.02	-0.07	0.03	0.474
Cholesterol esters to total lipids ratio in large LDL (%)	1076	-0.03	-0.09	0.02	0.225	1054	-0.02	-0.07	0.03	0.458
Free cholesterol to total lipids ratio in large LDL (%)	1076	0.03	-0.02	0.08	0.306	1054	0.01	-0.04	0.06	0.684
Triglycerides to total lipids ratio in large LDL (%)	1076	0.02	-0.04	0.08	0.487	1054	0.02	-0.04	0.08	0.563
Phospholipids to total lipds ratio in medium LDL (%)	1076	0.01	-0.01	0.03	0.412	1054	0.01	-0.01	0.03	0.607
Total cholesterol to total lipids ratio in medium LDL (%)	1076	-0.04	-0.09	0.02	0.203	1054	-0.03	-0.08	0.03	0.338
Cholesterol esters to total lipids ratio in medium LDL (%)	1076	-0.03	-0.09	0.02	0.264	1054	-0.02	-0.08	0.04	0.457
Free cholesterol to total lipids ratio in medium LDL (%)	1076	0.01	-0.01	0.02	0.474	1054	0.00	-0.01	0.02	0.754
Triglycerides to total lipids ratio in medium LDL (%)	1076	0.04	-0.02	0.09	0.236	1054	0.03	-0.03	0.09	0.270
Phospholipids to total lipds ratio in small LDL (%)	1076	0.01	-0.02	0.05	0.400	1054	0.01	-0.02	0.04	0.635
Total cholesterol to total lipids ratio in small LDL (%)	1076	-0.02	-0.08	0.03	0.387	1054	-0.02	-0.07	0.04	0.553
Cholesterol esters to total lipids ratio in small LDL (%)	1076	-0.02	-0.08	0.03	0.439	1054	-0.01	-0.07	0.04	0.656
Free cholesterol to total lipids ratio in small LDL (%)	1076	0.01	-0.02	0.04	0.622	1054	0.00	-0.03	0.03	0.901
Triglycerides to total lipids ratio in small LDL (%)	1076	0.01	-0.05	0.07	0.736	1054	0.02	-0.04	0.07	0.594
Phospholipids to total lipds ratio in very large HDL (%)	1076	0.03	-0.02	0.09	0.221	1054	0.02	-0.04	0.07	0.596
Total cholesterol to total lipids ratio in very large HDL (%)	1076	-0.02	-0.08	0.03	0.395	1054	-0.01	-0.06	0.05	0.829
Cholesterol esters to total lipids ratio in very large HDL (%)	1076	-0.03	-0.08	0.03	0.383	1054	-0.01	-0.06	0.05	0.806
Free cholesterol to total lipids ratio in very large HDL (%)	1076	0.02	-0.04	0.07	0.548	1054	0.01	-0.04	0.07	0.655
Triglycerides to total lipids ratio in very large HDL (%)	1076	-0.06	-0.12	-0.01	0.027	1054	-0.05	-0.11	0.01	0.079
Phospholipids to total lipds ratio in large HDL (%)	1076	-0.03	-0.08	0.03	0.357	1054	-0.01	-0.06	0.05	0.768
Total cholesterol to total lipids ratio in large HDL (%)	1076	0.04	-0.02	0.09	0.171	1054	0.02	-0.04	0.08	0.485
Cholesterol esters to total lipids ratio in large HDL (%)	1076	0.04	-0.02	0.09	0.167	1054	0.02	-0.04	0.08	0.493
Free cholesterol to total lipids ratio in large HDL (%)	1076	0.03	-0.03	0.09	0.323	1054	0.02	-0.04	0.07	0.589
Triglycerides to total lipids ratio in large HDL (%)	1076	-0.05	-0.11	0.00	0.061	1054	-0.04	-0.09	0.02	0.206
Phospholipids to total lipds ratio in medium HDL (%)	1076	0.02	-0.04	0.08	0.551	1054	0.03	-0.04	0.09	0.400
Total cholesterol to total lipids ratio in medium HDL (%)	1076	0.01	-0.05	0.07	0.793	1054	-0.01	-0.07	0.06	0.845
Cholesterol esters to total lipids ratio in medium HDL (%)	1076	0.01	-0.05	0.07	0.745	1054	0.00	-0.07	0.06	0.882
Free cholesterol to total lipids ratio in medium HDL (%)	1076	0.00	-0.07	0.07	0.975	1054	0.00	-0.07	0.06	0.895
Triglycerides to total lipids ratio in medium HDL (%)	1076	-0.04	-0.10	0.02	0.178	1054	-0.03	-0.09	0.04	0.394
Phospholipids to total lipds ratio in small HDL (%)	1076	-0.05	-0.10	0.01	0.102	1054	-0.05	-0.11	0.00	0.066
Total cholesterol to total lipids ratio in small HDL (%)	1076	0.05	0.00	0.11	0.058	1054	0.05	0.00	0.11	0.049
Cholesterol esters to total lipids ratio in small HDL (%)	1076	0.05	0.00	0.11	0.051	1054	0.06	0.00	0.11	0.039
Free cholesterol to total lipids ratio in small HDL (%)	1076	-0.03	-0.09	0.02	0.255	1054	-0.04	-0.10	0.02	0.144
Triglycerides to total lipids ratio in small HDL (%)	1076	-0.04	-0.10	0.02	0.184	1054	-0.02	-0.08	0.04	0.433
Mean diameter for VLDL particles (nm)	1076	-0.04	-0.10	0.02	0.162	1054	-0.03	-0.08	0.03	0.352
Mean diameter for LDL particles (nm)	1076	0.01	-0.04	0.06	0.685	1054	0.00	-0.06	0.05	0.878
Mean diameter for HDL particles (nm)	1076	0.02	-0.04	0.08	0.506	1054	0.01	-0.05	0.07	0.722
Serum total cholesterol (mmol/l)	1076	-0.03	-0.08	0.03	0.360	1054	-0.01	-0.07	0.05	0.676
Total cholesterol in VLDL (mmol/l)	1076	-0.06	-0.12	0.00	0.037	1054	-0.04	-0.09	0.02	0.197
Remnant cholesterol (non-HDL, non-LDL -cholesterol) (mmol/l)	1076	-0.05	-0.11	0.00	0.065	1054	-0.03	-0.09	0.02	0.264
Total cholesterol in LDL (mmol/l)	1076	-0.03	-0.08	0.03	0.342	1054	-0.01	-0.07	0.04	0.653
Total cholesterol in HDL (mmol/l)	1076	0.03	-0.03	0.08	0.400	1054	0.02	-0.04	0.08	0.478
Total cholesterol in HDL2 (mmol/l)	1076	0.03	-0.02	0.09	0.256	1054	0.03	-0.03	0.09	0.361
Total cholesterol in HDL3 (mmol/l)	1076	0.01	-0.05	0.07	0.783	1054	0.01	-0.05	0.07	0.756
Esterified cholesterol (mmol/l)	1076	-0.02	-0.08	0.03	0.407	1054	-0.01	-0.07	0.05	0.750
Free cholesterol (mmol/l)	1076	-0.03	-0.09	0.03	0.294	1054	-0.02	-0.08	0.04	0.544

S9 Table Associations of change in total physical activity (CPM change from age 12y-15y) with metabolic traits at age 15y in ALSPAC

Standardised outcome at age 15y	Change in CPM from age 12y-15y (per SD-unit increase): *Adj. for age, sex, ethnicity, maternal education change in wear time, wear month* N	Beta	LCL	UCL	P-value	*Additionally adj. for change in FMI* N	Beta	LCL	UCL	P-value
Serum total triglycerides (mmol/l)	1076	-0.05	-0.11	0.00	0.058	1054	-0.03	-0.09	0.02	0.231
Triglycerides in VLDL (mmol/l)	1076	-0.05	-0.11	0.00	0.051	1054	-0.03	-0.09	0.02	0.208
Triglycerides in LDL (mmol/l)	1076	-0.02	-0.08	0.04	0.562	1054	-0.01	-0.07	0.05	0.780
Triglycerides in HDL (mmol/l)	1076	-0.05	-0.11	0.01	0.118	1054	-0.03	-0.10	0.03	0.282
Diacylglycerol (mmol/l)	1044	0.00	-0.05	0.06	0.931	1023	0.02	-0.04	0.08	0.550
Ratio of diacylglycerol to triglycerides	1044	0.05	-0.01	0.10	0.129	1023	0.05	-0.01	0.11	0.091
Total phosphoglycerides (mmol/l)	1076	-0.02	-0.08	0.04	0.454	1054	-0.01	-0.07	0.05	0.696
Ratio of triglycerides to phosphoglycerides	1076	-0.03	-0.08	0.03	0.331	1054	-0.01	-0.07	0.04	0.625
Phosphatidylcholine and other cholines (mmol/l)	1056	-0.04	-0.10	0.03	0.264	1034	-0.03	-0.09	0.03	0.367
Total cholines (mmol/l)	1073	-0.02	-0.08	0.04	0.577	1051	-0.01	-0.07	0.05	0.778
Apolipoprotein A-I (g/l)	1076	0.01	-0.05	0.06	0.860	1054	0.01	-0.05	0.07	0.783
Apolipoprotein B (g/l)	1076	-0.06	-0.11	0.00	0.052	1054	-0.04	-0.09	0.02	0.229
Ratio of apolipoprotein B to apolipoprotein A-I	1076	-0.06	-0.12	0.00	0.053	1054	-0.04	-0.10	0.02	0.222
Total fatty acids (mmol/l)	1076	-0.04	-0.09	0.02	0.213	1054	-0.02	-0.08	0.04	0.518
Estimated description of fatty acid chain length, not actual carbon number	1072	0.01	-0.05	0.08	0.646	1050	0.01	-0.05	0.08	0.675
Estimated degree of unsaturation	1075	0.03	-0.03	0.10	0.336	1053	0.02	-0.04	0.08	0.543
22:6, docosahexaenoic acid (mmol/l)	1076	-0.01	-0.07	0.04	0.624	1054	0.00	-0.06	0.05	0.904
18:2, linoleic acid (mmol/l)	1073	-0.01	-0.06	0.05	0.818	1051	0.00	-0.05	0.06	0.875
Conjugated linoleic acid (mmol/l)	1076	-0.01	-0.07	0.06	0.824	1054	0.00	-0.07	0.07	0.962
Omega-3 fatty acids (mmol/l)	1074	0.00	-0.06	0.06	0.929	1052	0.01	-0.04	0.07	0.637
Omega-6 fatty acids (mmol/l)	1075	-0.01	-0.07	0.05	0.707	1053	0.00	-0.06	0.06	0.963
Polyunsaturated fatty acids (mmol/l)	1073	-0.01	-0.07	0.05	0.762	1051	0.00	-0.05	0.06	0.877
Monounsaturated fatty acids; 16:1, 18:1 (mmol/l)	1073	-0.06	-0.12	0.00	0.054	1051	-0.04	-0.11	0.02	0.156
Saturated fatty acids (mmol/l)	1072	-0.03	-0.09	0.03	0.307	1050	-0.01	-0.07	0.05	0.701
Ratio of 22:6 docosahexaenoic acid to total fatty acids (%)	1076	0.00	-0.06	0.05	0.947	1054	0.00	-0.05	0.06	0.948
Ratio of 18:2 linoleic acid to total fatty acids (%)	1073	0.04	-0.02	0.11	0.180	1051	0.03	-0.03	0.10	0.304
Ratio of conjugated linoleic acid to total fatty acids (%)	1076	0.00	-0.07	0.07	0.948	1054	0.01	-0.06	0.08	0.874
Ratio of omega-3 fatty acids to total fatty acids (%)	1074	0.01	-0.05	0.08	0.677	1052	0.02	-0.04	0.09	0.473
Ratio of omega-6 fatty acids to total fatty acids (%)	1075	0.05	-0.01	0.11	0.116	1053	0.04	-0.03	0.10	0.237
Ratio of polyunsaturated fatty acids to total fatty acids (%)	1073	0.05	-0.02	0.11	0.146	1051	0.04	-0.03	0.10	0.242
Ratio of monounsaturated fatty acids to total fatty acids (%)	1073	-0.05	-0.11	0.01	0.123	1051	-0.04	-0.11	0.02	0.173
Ratio of saturated fatty acids to total fatty acids (%)	1072	0.01	-0.06	0.07	0.844	1050	0.01	-0.05	0.08	0.735
Insulin (mu/l)	1118	0.01	-0.02	0.05	0.426	1095	0.03	-0.01	0.06	0.125
Glucose (mmol/l)	1074	-0.02	-0.07	0.03	0.456	1052	-0.01	-0.06	0.04	0.723
Lactate (mmol/l)	1074	-0.03	-0.09	0.03	0.390	1052	-0.02	-0.09	0.04	0.486
Pyruvate (mmol/l)	1073	0.00	-0.06	0.06	0.959	1051	0.01	-0.06	0.07	0.824
Citrate (mmol/l)	1070	0.03	-0.04	0.09	0.390	1048	0.03	-0.04	0.09	0.433
Alanine (mmol/l)	1076	-0.01	-0.07	0.06	0.846	1054	0.00	-0.07	0.06	0.930
Glutamine (mmol/l)	1076	0.03	-0.02	0.09	0.268	1054	0.03	-0.03	0.08	0.361
Histidine (mmol/l)	1015	-0.02	-0.08	0.04	0.504	994	-0.01	-0.07	0.05	0.756
Isoleucine (mmol/l)	1076	0.01	-0.06	0.07	0.869	1054	0.02	-0.05	0.08	0.626
Leucine (mmol/l)	1076	0.04	-0.01	0.10	0.140	1054	0.05	-0.01	0.11	0.089
Valine (mmol/l)	1076	0.01	-0.05	0.07	0.779	1054	0.02	-0.04	0.08	0.524
Phenylalanine (mmol/l)	1075	0.06	0.00	0.11	0.058	1053	0.06	0.00	0.12	0.039
Tyrosine (mmol/l)	1070	0.02	-0.05	0.08	0.623	1048	0.03	-0.03	0.10	0.331
Acetate (mmol/l)	1075	-0.02	-0.08	0.04	0.559	1053	-0.02	-0.08	0.04	0.504
Acetoacetate (mmol/l)	1076	0.08	-0.03	0.18	0.156	1054	0.07	-0.04	0.18	0.206
3-hydroxybutyrate (mmol/l)	1075	0.03	-0.03	0.09	0.373	1053	0.02	-0.04	0.09	0.453
Creatinine (mmol/l)	1075	-0.03	-0.09	0.03	0.336	1053	-0.03	-0.09	0.02	0.254
Albumin (signal area)	1076	-0.04	-0.09	0.02	0.215	1054	-0.04	-0.10	0.02	0.170
Glycoprotein acetyls, mainly a1-acid glycoprotein (mmol/l)	1075	-0.02	-0.08	0.03	0.399	1053	-0.01	-0.07	0.04	0.676
C-reactive protein (mg/l)	1120	0.00	-0.03	0.03	0.964	1097	0.00	-0.03	0.03	0.765

Complete case sample

Standardised outcome at age 15y	Change in CPM from age 12y-15y (per SD-unit increase): *Adj. for age, sex, ethnicity, maternal education change in wear time, wear month* N	Beta	LCL	UCL	P-value	*Additionally adj. for change in FMI* N	Beta	LCL	UCL	P-value
Systolic blood pressure (mmHg)	755	0.03	-0.04	0.11	0.346	755	0.04	-0.03	0.11	0.314
Diastolic blood pressure (mmHg)	755	0.02	-0.05	0.09	0.639	755	0.02	-0.05	0.09	0.644
Concentration of chylomicrons and extremely large VLDL particles (mol/l)	755	-0.03	-0.09	0.03	0.338	755	-0.01	-0.07	0.05	0.729
Total lipids in chylomicrons and extremely large VLDL (mmol/l)	755	-0.03	-0.09	0.03	0.307	755	-0.01	-0.07	0.05	0.670
Phospholipids in chylomicrons and extremely large VLDL (mmol/l)	755	-0.03	-0.09	0.03	0.282	755	-0.01	-0.07	0.04	0.619
Total cholesterol in chylomicrons and extremely large VLDL (mmol/l)	755	-0.02	-0.08	0.04	0.453	755	0.00	-0.06	0.06	0.907
Cholesterol esters in chylomicrons and extremely large VLDL (mmol/l)	755	-0.02	-0.08	0.05	0.601	755	0.00	-0.06	0.07	0.882
Free cholesterol in chylomicrons and extremely large VLDL (mmol/l)	755	-0.03	-0.09	0.03	0.331	755	-0.01	-0.07	0.05	0.683
Triglycerides in chylomicrons and extremely large VLDL (mmol/l)	755	-0.03	-0.09	0.03	0.284	755	-0.01	-0.07	0.04	0.627
Concentration of very large VLDL particles (mol/l)	755	-0.03	-0.09	0.03	0.292	755	-0.01	-0.07	0.04	0.624
Total lipids in very large VLDL (mmol/l)	755	-0.03	-0.09	0.03	0.286	755	-0.02	-0.08	0.04	0.613
Phospholipids in very large VLDL (mmol/l)	755	-0.03	-0.09	0.03	0.312	755	-0.01	-0.07	0.05	0.654
Total cholesterol in very large VLDL (mmol/l)	755	-0.03	-0.09	0.03	0.350	755	-0.01	-0.07	0.05	0.748
Cholesterol esters in very large VLDL (mmol/l)	755	-0.03	-0.09	0.03	0.362	755	-0.01	-0.07	0.05	0.772
Free cholesterol in very large VLDL (mmol/l)	755	-0.03	-0.09	0.03	0.341	755	-0.01	-0.07	0.05	0.723
Triglycerides in very large VLDL (mmol/l)	755	-0.03	-0.10	0.03	0.264	755	-0.02	-0.08	0.04	0.567
Concentration of large VLDL particles (mol/l)	755	-0.03	-0.10	0.03	0.280	755	-0.02	-0.08	0.05	0.596
Total lipids in large VLDL (mmol/l)	755	-0.03	-0.10	0.03	0.282	755	-0.02	-0.08	0.05	0.603
Phospholipids in large VLDL (mmol/l)	755	-0.03	-0.10	0.03	0.296	755	-0.02	-0.08	0.05	0.625
Total cholesterol in large VLDL (mmol/l)	755	-0.03	-0.10	0.03	0.314	755	-0.01	-0.08	0.05	0.676
Cholesterol esters in large VLDL (mmol/l)	755	-0.03	-0.09	0.03	0.355	755	-0.01	-0.07	0.05	0.745
Free cholesterol in large VLDL (mmol/l)	755	-0.03	-0.10	0.03	0.283	755	-0.02	-0.08	0.05	0.614
Triglycerides in large VLDL (mmol/l)	755	-0.04	-0.10	0.03	0.269	755	-0.02	-0.08	0.04	0.571
Concentration of medium VLDL particles (mol/l)	755	-0.04	-0.10	0.03	0.250	755	-0.02	-0.08	0.04	0.556
Total lipids in medium VLDL (mmol/l)	755	-0.04	-0.10	0.03	0.265	755	-0.02	-0.08	0.05	0.590
Phospholipids in medium VLDL (mmol/l)	755	-0.04	-0.10	0.03	0.244	755	-0.02	-0.08	0.05	0.559
Total cholesterol in medium VLDL (mmol/l)	755	-0.03	-0.10	0.03	0.331	755	-0.01	-0.08	0.05	0.729
Cholesterol esters in medium VLDL (mmol/l)	755	-0.03	-0.09	0.04	0.431	755	0.00	-0.07	0.06	0.884
Free cholesterol in medium VLDL (mmol/l)	755	-0.04	-0.10	0.03	0.254	755	-0.02	-0.08	0.05	0.571
Triglycerides in medium VLDL (mmol/l)	755	-0.04	-0.10	0.03	0.256	755	-0.02	-0.08	0.04	0.545

S9 Table Associations of change in total physical activity (CPM change from age 12y-15y) with metabolic traits at age 15y in ALSPAC

	Change in CPM from age 12y-15y (per SD-unit increase)									
	Adj. for age, sex, ethnicity, maternal education change in wear time, wear month					*Additionally adj. for change in FMI*				
**Standardised outcome at age 15y**	**N**	**Beta**	**LCL**	**UCL**	**P-value**	**N**	**Beta**	**LCL**	**UCL**	**P-value**
Concentration of small VLDL particles (mol/l)	755	-0.04	-0.10	0.03	0.300	755	-0.02	-0.08	0.05	0.652
Total lipids in small VLDL (mmol/l)	755	-0.03	-0.10	0.03	0.313	755	-0.01	-0.08	0.05	0.700
Phospholipids in small VLDL (mmol/l)	755	-0.03	-0.10	0.04	0.363	755	-0.01	-0.08	0.06	0.755
Total cholesterol in small VLDL (mmol/l)	755	-0.03	-0.10	0.03	0.339	755	-0.01	-0.08	0.06	0.785
Cholesterol esters in small VLDL (mmol/l)	755	-0.03	-0.09	0.04	0.387	755	-0.01	-0.07	0.06	0.849
Free cholesterol in small VLDL (mmol/l)	755	-0.03	-0.10	0.03	0.315	755	-0.01	-0.08	0.05	0.706
Triglycerides in small VLDL (mmol/l)	755	-0.03	-0.10	0.03	0.335	755	-0.02	-0.08	0.05	0.645
Concentration of very small VLDL particles (mol/l)	755	-0.02	-0.08	0.05	0.614	755	0.00	-0.07	0.07	0.970
Total lipids in very small VLDL (mmol/l)	755	-0.02	-0.09	0.04	0.461	755	0.00	-0.07	0.06	0.888
Phospholipids in very small VLDL (mmol/l)	755	-0.01	-0.08	0.05	0.702	755	0.00	-0.06	0.07	0.920
Total cholesterol in very small VLDL (mmol/l)	755	-0.03	-0.09	0.04	0.381	755	-0.01	-0.08	0.06	0.774
Cholesterol esters in very small VLDL (mmol/l)	755	-0.03	-0.09	0.04	0.385	755	-0.01	-0.08	0.06	0.771
Free cholesterol in very small VLDL (mmol/l)	755	-0.03	-0.09	0.04	0.417	755	-0.01	-0.07	0.05	0.789
Triglycerides in very small VLDL (mmol/l)	755	-0.02	-0.09	0.05	0.635	755	0.00	-0.07	0.07	0.951
Concentration of IDL particles (mol/l)	755	-0.01	-0.08	0.05	0.674	755	0.00	-0.07	0.07	0.989
Total lipids in IDL (mmol/l)	755	-0.01	-0.08	0.05	0.699	755	0.00	-0.07	0.07	0.954
Phospholipids in IDL (mmol/l)	755	-0.01	-0.07	0.06	0.822	755	0.00	-0.06	0.07	0.886
Total cholesterol in IDL (mmol/l)	755	-0.02	-0.08	0.05	0.606	755	0.00	-0.07	0.07	0.981
Cholesterol esters in IDL (mmol/l)	755	-0.02	-0.09	0.04	0.523	755	0.00	-0.07	0.06	0.924
Free cholesterol in IDL (mmol/l)	755	-0.01	-0.07	0.06	0.830	755	0.00	-0.06	0.07	0.893
Triglycerides in IDL (mmol/l)	755	0.00	-0.07	0.08	0.904	755	0.01	-0.06	0.09	0.765
Concentration of large LDL particles (mol/l)	755	-0.01	-0.08	0.06	0.789	755	0.01	-0.06	0.07	0.884
Total lipids in large LDL (mmol/l)	755	-0.01	-0.07	0.06	0.832	755	0.01	-0.06	0.08	0.831
Phospholipids in large LDL (mmol/l)	755	-0.01	-0.07	0.06	0.833	755	0.01	-0.06	0.08	0.804
Total cholesterol in large LDL (mmol/l)	755	-0.01	-0.07	0.06	0.804	755	0.01	-0.06	0.07	0.845
Cholesterol esters in large LDL (mmol/l)	755	-0.01	-0.08	0.06	0.784	755	0.01	-0.06	0.07	0.850
Free cholesterol in large LDL (mmol/l)	755	-0.01	-0.07	0.06	0.871	755	0.01	-0.06	0.07	0.833
Triglycerides in large LDL (mmol/l)	755	0.00	-0.07	0.08	0.918	755	0.01	-0.07	0.08	0.822
Concentration of medium LDL particles (mol/l)	755	-0.01	-0.08	0.06	0.765	755	0.00	-0.06	0.07	0.893
Total lipids in medium LDL (mmol/l)	755	-0.01	-0.08	0.06	0.782	755	0.01	-0.06	0.07	0.866
Phospholipids in medium LDL (mmol/l)	755	-0.01	-0.08	0.05	0.730	755	0.01	-0.06	0.07	0.848
Total cholesterol in medium LDL (mmol/l)	755	-0.01	-0.08	0.05	0.737	755	0.00	-0.06	0.07	0.908
Cholesterol esters in medium LDL (mmol/l)	755	-0.01	-0.08	0.06	0.766	755	0.00	-0.06	0.07	0.886
Free cholesterol in medium LDL (mmol/l)	755	-0.02	-0.08	0.05	0.611	755	0.00	-0.07	0.07	0.981
Triglycerides in medium LDL (mmol/l)	755	0.01	-0.06	0.09	0.708	755	0.02	-0.06	0.09	0.642
Concentration of small LDL particles (mol/l)	755	-0.02	-0.08	0.05	0.595	755	0.00	-0.07	0.06	0.941
Total lipids in small LDL (mmol/l)	755	-0.01	-0.08	0.05	0.703	755	0.00	-0.06	0.07	0.932
Phospholipids in small LDL (mmol/l)	755	-0.02	-0.09	0.05	0.560	755	0.00	-0.07	0.07	0.960
Total cholesterol in small LDL (mmol/l)	755	-0.01	-0.08	0.05	0.729	755	0.00	-0.06	0.07	0.918
Cholesterol esters in small LDL (mmol/l)	755	-0.01	-0.07	0.06	0.807	755	0.01	-0.06	0.07	0.860
Free cholesterol in small LDL (mmol/l)	755	-0.03	-0.09	0.04	0.445	755	-0.01	-0.07	0.06	0.826
Triglycerides in small LDL (mmol/l)	755	0.00	-0.07	0.07	0.995	755	0.01	-0.06	0.08	0.791
Concentration of very large HDL particles (mol/l)	755	0.02	-0.06	0.09	0.668	755	0.01	-0.06	0.09	0.784
Total lipids in very large HDL (mmol/l)	755	0.01	-0.06	0.08	0.794	755	0.00	-0.07	0.08	0.898
Phospholipids in very large HDL (mmol/l)	755	0.02	-0.05	0.10	0.512	755	0.02	-0.06	0.09	0.657
Total cholesterol in very large HDL (mmol/l)	755	-0.01	-0.08	0.07	0.866	755	-0.01	-0.08	0.07	0.828
Cholesterol esters in very large HDL (mmol/l)	755	-0.01	-0.09	0.06	0.742	755	-0.01	-0.09	0.06	0.733
Free cholesterol in very large HDL (mmol/l)	755	0.01	-0.07	0.08	0.812	755	0.00	-0.07	0.08	0.919
Triglycerides in very large HDL (mmol/l)	755	-0.03	-0.10	0.05	0.476	755	-0.02	-0.09	0.05	0.599
Concentration of large HDL particles (mol/l)	755	0.02	-0.05	0.10	0.546	755	0.01	-0.06	0.09	0.702
Total lipids in large HDL (mmol/l)	755	0.02	-0.05	0.10	0.517	755	0.02	-0.06	0.09	0.684
Phospholipids in large HDL (mmol/l)	755	0.02	-0.05	0.10	0.516	755	0.02	-0.06	0.09	0.658
Total cholesterol in large HDL (mmol/l)	755	0.03	-0.05	0.10	0.497	755	0.02	-0.06	0.09	0.688
Cholesterol esters in large HDL (mmol/l)	755	0.03	-0.05	0.10	0.491	755	0.02	-0.06	0.09	0.685
Free cholesterol in large HDL (mmol/l)	755	0.02	-0.05	0.10	0.530	755	0.01	-0.06	0.09	0.708
Triglycerides in large HDL (mmol/l)	755	-0.01	-0.09	0.06	0.704	755	-0.01	-0.08	0.06	0.787
Concentration of medium HDL particles (mol/l)	755	0.02	-0.05	0.09	0.629	755	0.02	-0.05	0.09	0.585
Total lipids in medium HDL (mmol/l)	755	0.02	-0.05	0.09	0.616	755	0.02	-0.05	0.09	0.606
Phospholipids in medium HDL (mmol/l)	755	0.02	-0.05	0.09	0.576	755	0.02	-0.05	0.09	0.553
Total cholesterol in medium HDL (mmol/l)	755	0.02	-0.05	0.09	0.643	755	0.01	-0.06	0.09	0.697
Cholesterol esters in medium HDL (mmol/l)	755	0.02	-0.05	0.09	0.639	755	0.01	-0.06	0.09	0.710
Free cholesterol in medium HDL (mmol/l)	755	0.02	-0.05	0.08	0.639	755	0.02	-0.05	0.09	0.620
Triglycerides in medium HDL (mmol/l)	755	-0.01	-0.08	0.06	0.790	755	0.00	-0.07	0.08	0.907
Concentration of small HDL particles (mol/l)	755	0.00	-0.07	0.07	0.917	755	0.01	-0.06	0.08	0.771
Total lipids in small HDL (mmol/l)	755	0.02	-0.05	0.09	0.585	755	0.03	-0.04	0.10	0.473
Phospholipids in small HDL (mmol/l)	755	-0.01	-0.08	0.06	0.733	755	-0.01	-0.08	0.06	0.789
Total cholesterol in small HDL (mmol/l)	755	0.05	-0.02	0.11	0.163	755	0.05	-0.01	0.12	0.118
Cholesterol esters in small HDL (mmol/l)	755	0.06	-0.01	0.12	0.095	755	0.06	0.00	0.13	0.063
Free cholesterol in small HDL (mmol/l)	755	0.00	-0.07	0.06	0.888	755	0.00	-0.07	0.07	0.901
Triglycerides in small HDL (mmol/l)	755	-0.01	-0.08	0.06	0.778	755	0.00	-0.07	0.07	0.960
Phospholipids to total lipds ratio in chylomicrons and extremely large VLDL (%)	755	-0.04	-0.11	0.03	0.254	755	-0.03	-0.10	0.03	0.335
Total cholesterol to total lipids ratio in chylomicrons and extremely large VLDL (%)	755	0.02	-0.05	0.09	0.541	755	0.04	-0.03	0.11	0.298
Cholesterol esters to total lipids ratio in chylomicrons and extremely large VLDL (%)	755	0.03	-0.04	0.10	0.397	755	0.05	-0.03	0.12	0.209
Free cholesterol to total lipids ratio in chylomicrons and extremely large VLDL (%)	755	-0.01	-0.08	0.06	0.758	755	0.00	-0.07	0.07	0.989
Triglycerides to total lipids ratio in chylomicrons and extremely large VLDL (%)	755	-0.02	-0.07	0.03	0.428	755	-0.03	-0.08	0.02	0.237
Phospholipids to total lipds ratio in very large VLDL (%)	755	0.00	-0.07	0.07	0.990	755	0.02	-0.05	0.09	0.631
Total cholesterol to total lipids ratio in very large VLDL (%)	755	0.08	-0.07	0.23	0.277	755	0.07	-0.07	0.21	0.303
Cholesterol esters to total lipids ratio in very large VLDL (%)	755	0.02	-0.06	0.10	0.553	755	0.02	-0.06	0.10	0.676
Free cholesterol to total lipids ratio in very large VLDL (%)	755	0.03	-0.05	0.10	0.492	755	0.02	-0.05	0.10	0.519
Triglycerides to total lipids ratio in very large VLDL (%)	755	-0.03	-0.11	0.05	0.441	755	-0.03	-0.11	0.05	0.437
Phospholipids to total lipds ratio in large VLDL (%)	755	-0.01	-0.08	0.07	0.890	755	0.01	-0.07	0.08	0.810
Total cholesterol to total lipids ratio in large VLDL (%)	755	0.00	-0.08	0.08	0.946	755	0.02	-0.06	0.09	0.701
Cholesterol esters to total lipids ratio in large VLDL (%)	755	0.11	-0.13	0.36	0.356	755	0.11	-0.11	0.32	0.346
Free cholesterol to total lipids ratio in large VLDL (%)	755	-0.01	-0.08	0.06	0.783	755	0.01	-0.06	0.08	0.835
Triglycerides to total lipids ratio in large VLDL (%)	755	0.14	-0.13	0.42	0.304	755	0.13	-0.12	0.37	0.312
Phospholipids to total lipds ratio in medium VLDL (%)	755	0.01	-0.07	0.08	0.849	755	0.01	-0.07	0.08	0.882
Total cholesterol to total lipids ratio in medium VLDL (%)	755	-0.01	-0.07	0.06	0.817	755	0.01	-0.06	0.07	0.851
Cholesterol esters to total lipids ratio in medium VLDL (%)	755	0.00	-0.07	0.06	0.936	755	0.01	-0.06	0.07	0.790
Free cholesterol to total lipids ratio in medium VLDL (%)	755	-0.02	-0.09	0.05	0.647	755	0.00	-0.07	0.07	0.959
Triglycerides to total lipids ratio in medium VLDL (%)	755	0.00	-0.06	0.07	0.916	755	-0.01	-0.07	0.06	0.794
Phospholipids to total lipds ratio in small VLDL (%)	755	0.04	-0.03	0.10	0.246	755	0.02	-0.04	0.09	0.482

S9 Table Associations of change in total physical activity (CPM change from age 12y-15y) with metabolic traits at age 15y in ALSPAC

	**Change in CPM from age 12y-15y (per SD-unit increase)**									
	Adj. for age, sex, ethnicity, maternal education change in wear time, wear month					*Additionally adj. for change in FMI*				
**Standardised outcome at age 15y**	**N**	**Beta**	**LCL**	**UCL**	**P-value**	**N**	**Beta**	**LCL**	**UCL**	**P-value**
Total cholesterol to total lipids ratio in small VLDL (%)	755	-0.01	-0.08	0.06	0.745	755	-0.01	-0.08	0.06	0.867
Cholesterol esters to total lipids ratio in small VLDL (%)	755	-0.01	-0.08	0.06	0.738	755	-0.01	-0.08	0.06	0.858
Free cholesterol to total lipids ratio in small VLDL (%)	755	0.01	-0.06	0.08	0.838	755	0.01	-0.06	0.08	0.799
Triglycerides to total lipids ratio in small VLDL (%)	755	0.00	-0.07	0.07	0.985	755	0.00	-0.07	0.07	0.992
Phospholipids to total lipds ratio in very small VLDL (%)	755	0.01	-0.05	0.08	0.705	755	0.02	-0.05	0.09	0.595
Total cholesterol to total lipids ratio in very small VLDL (%)	755	-0.02	-0.09	0.04	0.479	755	-0.03	-0.10	0.04	0.442
Cholesterol esters to total lipids ratio in very small VLDL (%)	755	-0.02	-0.10	0.05	0.507	755	-0.03	-0.10	0.05	0.477
Free cholesterol to total lipids ratio in very small VLDL (%)	755	-0.01	-0.07	0.04	0.599	755	-0.02	-0.07	0.04	0.570
Triglycerides to total lipids ratio in very small VLDL (%)	755	0.02	-0.04	0.09	0.527	755	0.02	-0.05	0.09	0.552
Phospholipids to total lipds ratio in IDL (%)	755	0.04	-0.02	0.11	0.167	755	0.03	-0.03	0.09	0.386
Total cholesterol to total lipids ratio in IDL (%)	755	-0.05	-0.12	0.02	0.133	755	-0.04	-0.11	0.03	0.262
Cholesterol esters to total lipids ratio in IDL (%)	755	-0.06	-0.13	0.01	0.096	755	-0.04	-0.11	0.03	0.228
Free cholesterol to total lipids ratio in IDL (%)	755	0.01	-0.05	0.07	0.681	755	0.01	-0.06	0.07	0.853
Triglycerides to total lipids ratio in IDL (%)	755	0.04	-0.03	0.11	0.224	755	0.04	-0.03	0.11	0.322
Phospholipids to total lipds ratio in large LDL (%)	755	0.01	-0.05	0.07	0.803	755	0.00	-0.06	0.06	0.960
Total cholesterol to total lipids ratio in large LDL (%)	755	-0.03	-0.09	0.04	0.414	755	-0.01	-0.08	0.05	0.667
Cholesterol esters to total lipids ratio in large LDL (%)	755	-0.02	-0.09	0.04	0.486	755	-0.01	-0.07	0.06	0.839
Free cholesterol to total lipids ratio in large LDL (%)	755	0.00	-0.06	0.06	0.992	755	-0.02	-0.08	0.05	0.621
Triglycerides to total lipids ratio in large LDL (%)	755	0.04	-0.03	0.11	0.291	755	0.03	-0.04	0.10	0.446
Phospholipids to total lipds ratio in medium LDL (%)	755	0.00	-0.02	0.03	0.801	755	0.00	-0.02	0.02	0.999
Total cholesterol to total lipids ratio in medium LDL (%)	755	-0.03	-0.10	0.03	0.336	755	-0.02	-0.09	0.04	0.526
Cholesterol esters to total lipids ratio in medium LDL (%)	755	-0.02	-0.09	0.05	0.596	755	-0.01	-0.07	0.06	0.862
Free cholesterol to total lipids ratio in medium LDL (%)	755	0.00	-0.02	0.02	0.882	755	0.00	-0.02	0.01	0.621
Triglycerides to total lipids ratio in medium LDL (%)	755	0.06	-0.01	0.13	0.105	755	0.05	-0.02	0.12	0.162
Phospholipids to total lipds ratio in small LDL (%)	755	0.00	-0.03	0.04	0.848	755	0.00	-0.04	0.04	0.901
Total cholesterol to total lipids ratio in small LDL (%)	755	-0.02	-0.08	0.05	0.592	755	-0.01	-0.07	0.06	0.797
Cholesterol esters to total lipids ratio in small LDL (%)	755	-0.01	-0.07	0.06	0.855	755	0.00	-0.06	0.07	0.900
Free cholesterol to total lipids ratio in small LDL (%)	755	-0.01	-0.04	0.03	0.687	755	-0.01	-0.05	0.02	0.483
Triglycerides to total lipids ratio in small LDL (%)	755	0.04	-0.03	0.11	0.252	755	0.04	-0.03	0.11	0.241
Phospholipids to total lipds ratio in very large HDL (%)	755	0.05	-0.02	0.12	0.169	755	0.03	-0.04	0.11	0.336
Total cholesterol to total lipids ratio in very large HDL (%)	755	-0.05	-0.12	0.03	0.210	755	-0.03	-0.10	0.04	0.373
Cholesterol esters to total lipids ratio in very large HDL (%)	755	-0.04	-0.12	0.03	0.233	755	-0.03	-0.10	0.04	0.403
Free cholesterol to total lipids ratio in very large HDL (%)	755	0.00	-0.07	0.06	0.910	755	-0.01	-0.07	0.06	0.841
Triglycerides to total lipids ratio in very large HDL (%)	755	-0.03	-0.10	0.03	0.299	755	-0.02	-0.09	0.04	0.515
Phospholipids to total lipds ratio in large HDL (%)	755	0.00	-0.07	0.06	0.889	755	0.01	-0.05	0.07	0.720
Total cholesterol to total lipids ratio in large HDL (%)	755	0.02	-0.05	0.08	0.614	755	0.00	-0.07	0.06	0.978
Cholesterol esters to total lipids ratio in large HDL (%)	755	0.02	-0.05	0.08	0.572	755	0.00	-0.06	0.06	0.999
Free cholesterol to total lipids ratio in large HDL (%)	755	0.01	-0.06	0.07	0.873	755	0.00	-0.07	0.06	0.883
Triglycerides to total lipids ratio in large HDL (%)	755	-0.03	-0.10	0.03	0.322	755	-0.02	-0.08	0.05	0.597
Phospholipids to total lipds ratio in medium HDL (%)	755	0.03	-0.04	0.10	0.403	755	0.03	-0.04	0.11	0.346
Total cholesterol to total lipids ratio in medium HDL (%)	755	-0.02	-0.09	0.05	0.612	755	-0.03	-0.10	0.04	0.415
Cholesterol esters to total lipids ratio in medium HDL (%)	755	-0.02	-0.09	0.05	0.610	755	-0.03	-0.10	0.04	0.399
Free cholesterol to total lipids ratio in medium HDL (%)	755	0.00	-0.08	0.09	0.930	755	0.00	-0.08	0.08	0.947
Triglycerides to total lipids ratio in medium HDL (%)	755	-0.01	-0.08	0.06	0.732	755	0.00	-0.07	0.07	0.946
Phospholipids to total lipds ratio in small HDL (%)	755	-0.06	-0.13	0.00	0.062	755	-0.07	-0.14	0.00	0.041
Total cholesterol to total lipids ratio in small HDL (%)	755	0.06	0.00	0.13	0.058	755	0.07	0.00	0.13	0.045
Cholesterol esters to total lipids ratio in small HDL (%)	755	0.07	0.00	0.13	0.039	755	0.07	0.01	0.14	0.027
Free cholesterol to total lipids ratio in small HDL (%)	755	-0.06	-0.12	0.00	0.059	755	-0.07	-0.14	-0.01	0.023
Triglycerides to total lipids ratio in small HDL (%)	755	-0.02	-0.08	0.05	0.650	755	0.00	-0.07	0.06	0.899
Mean diameter for VLDL particles (nm)	755	-0.03	-0.10	0.04	0.360	755	-0.02	-0.09	0.05	0.593
Mean diameter for LDL particles (nm)	755	0.01	-0.05	0.07	0.785	755	0.00	-0.06	0.06	0.929
Mean diameter for HDL particles (nm)	755	0.02	-0.06	0.09	0.614	755	0.01	-0.06	0.09	0.783
Serum total cholesterol (mmol/l)	755	-0.01	-0.08	0.06	0.790	755	0.01	-0.06	0.07	0.863
Total cholesterol in VLDL (mmol/l)	755	-0.04	-0.10	0.03	0.278	755	-0.01	-0.08	0.05	0.712
Remnant cholesterol (non-HDL, non-LDL -cholesterol) (mmol/l)	755	-0.03	-0.10	0.03	0.348	755	-0.01	-0.07	0.06	0.797
Total cholesterol in LDL (mmol/l)	755	-0.01	-0.08	0.06	0.770	755	0.01	-0.06	0.07	0.877
Total cholesterol in HDL (mmol/l)	755	0.02	-0.05	0.10	0.525	755	0.02	-0.05	0.09	0.609
Total cholesterol in HDL2 (mmol/l)	755	0.03	-0.04	0.10	0.430	755	0.02	-0.05	0.09	0.551
Total cholesterol in HDL3 (mmol/l)	755	0.01	-0.06	0.09	0.736	755	0.01	-0.06	0.09	0.732
Esterified cholesterol (mmol/l)	755	-0.01	-0.07	0.06	0.817	755	0.01	-0.06	0.07	0.828
Free cholesterol (mmol/l)	755	-0.01	-0.08	0.06	0.746	755	0.00	-0.07	0.07	0.945
Serum total triglycerides (mmol/l)	755	-0.03	-0.10	0.04	0.369	755	-0.01	-0.08	0.05	0.703
Triglycerides in VLDL (mmol/l)	755	-0.04	-0.10	0.03	0.280	755	-0.02	-0.08	0.05	0.588
Triglycerides in LDL (mmol/l)	755	0.01	-0.07	0.08	0.866	755	0.01	-0.06	0.09	0.758
Triglycerides in HDL (mmol/l)	755	-0.02	-0.09	0.06	0.648	755	0.00	-0.08	0.07	0.912
Diacylglycerol (mmol/l)	755	0.03	-0.04	0.10	0.343	755	0.05	-0.02	0.12	0.187
Ratio of diacylglycerol to triglycerides	755	0.06	-0.01	0.13	0.102	755	0.07	-0.01	0.14	0.079
Total phosphoglycerides (mmol/l)	755	0.00	-0.07	0.08	0.913	755	0.01	-0.06	0.09	0.730
Ratio of triglycerides to phosphoglycerides	755	-0.02	-0.08	0.04	0.455	755	-0.01	-0.07	0.05	0.803
Phosphatidylcholine and other cholines (mmol/l)	755	-0.01	-0.09	0.06	0.748	755	0.00	-0.08	0.07	0.898
Total cholines (mmol/l)	755	0.01	-0.06	0.09	0.736	755	0.02	-0.05	0.09	0.597
Apolipoprotein A-I (g/l)	755	0.01	-0.06	0.09	0.729	755	0.02	-0.06	0.09	0.679
Apolipoprotein B (g/l)	755	-0.03	-0.10	0.03	0.353	755	-0.01	-0.08	0.06	0.779
Ratio of apolipoprotein B to apolipoprotein A-I	755	-0.04	-0.10	0.03	0.304	755	-0.01	-0.08	0.05	0.678
Total fatty acids (mmol/l)	755	-0.01	-0.08	0.06	0.809	755	0.01	-0.06	0.08	0.832
Estimated description of fatty acid chain length, not actual carbon number	755	0.01	-0.05	0.07	0.808	755	0.01	-0.05	0.07	0.783
Estimated degree of unsaturation	755	0.02	-0.05	0.09	0.637	755	0.01	-0.06	0.08	0.755
22:6, docosahexaenoic acid (mmol/l)	755	0.02	-0.04	0.08	0.549	755	0.03	-0.03	0.09	0.356
18:2, linoleic acid (mmol/l)	755	0.02	-0.05	0.09	0.597	755	0.03	-0.04	0.10	0.353
Conjugated linoleic acid (mmol/l)	755	-0.01	-0.08	0.05	0.701	755	-0.01	-0.07	0.06	0.855
Omega-3 fatty acids (mmol/l)	755	-0.01	-0.07	0.06	0.863	755	0.01	-0.06	0.07	0.811
Omega-6 fatty acids (mmol/l)	755	0.01	-0.06	0.08	0.705	755	0.03	-0.04	0.10	0.445
Polyunsaturated fatty acids (mmol/l)	755	0.01	-0.06	0.08	0.745	755	0.03	-0.04	0.09	0.462
Monounsaturated fatty acids; 16:1, 18:1 (mmol/l)	755	-0.03	-0.10	0.04	0.386	755	-0.02	-0.09	0.05	0.644
Saturated fatty acids (mmol/l)	755	0.00	-0.08	0.07	0.910	755	0.01	-0.06	0.08	0.774
Ratio of 22:6 docosahexaenoic acid to total fatty acids (%)	755	0.02	-0.05	0.08	0.631	755	0.02	-0.05	0.09	0.547
Ratio of 18:2 linoleic acid to total fatty acids (%)	755	0.04	-0.03	0.11	0.240	755	0.04	-0.03	0.11	0.266
Ratio of conjugated linoleic acid to total fatty acids (%)	755	0.00	-0.07	0.06	0.964	755	0.00	-0.06	0.07	0.914
Ratio of omega-3 fatty acids to total fatty acids (%)	755	-0.02	-0.09	0.05	0.549	755	-0.02	-0.09	0.05	0.650
Ratio of omega-6 fatty acids to total fatty acids (%)	755	0.04	-0.04	0.11	0.315	755	0.03	-0.04	0.11	0.392
Ratio of polyunsaturated fatty acids to total fatty acids (%)	755	0.03	-0.04	0.11	0.399	755	0.03	-0.05	0.10	0.465

S9 Table Associations of change in total physical activity (CPM change from age 12y-15y) with metabolic traits at age 15y in ALSPAC

	Change in CPM from age 12y-15y (per SD-unit increase)									
	Adj. for age, sex, ethnicity, maternal education change in wear time, wear month					*Additionally adj. for change in FMI*				
**Standardised outcome at age 15y**	**N**	**Beta**	**LCL**	**UCL**	**P-value**	**N**	**Beta**	**LCL**	**UCL**	**P-value**
Ratio of monounsaturated fatty acids to total fatty acids (%)	755	-0.04	-0.11	0.04	0.331	755	-0.03	-0.10	0.04	0.402
Ratio of saturated fatty acids to total fatty acids (%)	755	0.01	-0.06	0.08	0.802	755	0.01	-0.06	0.08	0.831
Insulin (mu/l)	755	0.02	-0.03	0.06	0.470	755	0.03	-0.01	0.07	0.181
Glucose (mmol/l)	755	-0.02	-0.08	0.05	0.639	755	-0.01	-0.07	0.05	0.776
Lactate (mmol/l)	755	-0.03	-0.09	0.04	0.440	755	-0.02	-0.09	0.05	0.507
Pyruvate (mmol/l)	755	-0.03	-0.10	0.04	0.389	755	-0.03	-0.10	0.04	0.450
Citrate (mmol/l)	755	0.02	-0.06	0.10	0.600	755	0.02	-0.07	0.10	0.704
Alanine (mmol/l)	755	0.00	-0.07	0.07	0.997	755	0.00	-0.07	0.08	0.898
Glutamine (mmol/l)	755	0.03	-0.03	0.09	0.315	755	0.03	-0.04	0.09	0.412
Histidine (mmol/l)	755	0.02	-0.05	0.09	0.575	755	0.03	-0.04	0.10	0.455
Isoleucine (mmol/l)	755	-0.02	-0.08	0.04	0.481	755	-0.01	-0.07	0.05	0.703
Leucine (mmol/l)	755	0.02	-0.03	0.08	0.402	755	0.03	-0.03	0.08	0.313
Valine (mmol/l)	755	-0.01	-0.08	0.06	0.700	755	-0.01	-0.08	0.06	0.838
Phenylalanine (mmol/l)	755	0.04	-0.03	0.11	0.240	755	0.04	-0.03	0.11	0.221
Tyrosine (mmol/l)	755	0.05	-0.03	0.12	0.221	755	0.06	-0.02	0.13	0.126
Acetate (mmol/l)	755	0.01	-0.07	0.08	0.824	755	0.01	-0.07	0.08	0.848
Acetoacetate (mmol/l)	755	0.03	-0.03	0.10	0.310	755	0.02	-0.04	0.09	0.481
3-hydroxybutyrate (mmol/l)	755	0.03	-0.04	0.11	0.355	755	0.03	-0.04	0.10	0.425
Creatinine (mmol/l)	755	-0.02	-0.09	0.05	0.557	755	-0.03	-0.10	0.04	0.382
Albumin (signal area)	755	-0.02	-0.09	0.04	0.464	755	-0.03	-0.09	0.03	0.342
Glycoprotein acetyls, mainly a1-acid glycoprotein (mmol/l)	755	0.00	-0.07	0.07	0.999	755	0.01	-0.06	0.08	0.721
C-reactive protein (mg/l)	755	0.00	-0.04	0.03	0.851	755	0.00	-0.04	0.04	0.943
