# Supplementary material for: Associations of device-measured physical activity across adolescence with metabolic traits: Prospective cohort study
Source: PLoS Med. 2018 Sep 11;15(9):e1002649. doi: 10.1371/journal.pmed.1002649 (PMC6133272; doi:10.1371/journal.pmed.1002649)
Supplement: S10 Table — ALSPAC, Avon Longitudinal Study of Parents and Children; MVPA, moderate-to-vigorous physical activity. (PDF) [file pmed.1002649.s010.pdf]

S10 Table Associations of change in moderate-to-vigorous physical activity (MVPA change from age 12y-15y) with metabolic traits at age 15y in ALSPAC

Change in MVPA from age 12y-15y (per SD-unit increase)

	Adj. for age, sex, ethnicity, maternal education change in wear time, wear month					*Additionally adj. for change in SED*					*Additionally adj. for change in FMI*				
**Standardised outcome at age 15y**	**N**	**Beta**	**LCL**	**UCL**	**P-value**	**N**	**Beta**	**LCL**	**UCL**	**P-value**	**N**	**Beta**	**LCL**	**UCL**	**P-value**
Systolic blood pressure (mmHg)	1599	0.03	-0.01	0.08	0.133	1599	0.04	-0.01	0.08	0.124	1553	0.04	-0.01	0.08	0.121
Diastolic blood pressure (mmHg)	1599	0.07	0.02	0.11	0.004	1599	0.08	0.03	0.13	1.16E-03	1553	0.07	0.02	0.12	0.004
Concentration of chylomicrons and extremely large VLDL particles (mol/l)	1076	-0.05	-0.10	-0.01	0.030	1076	-0.05	-0.10	0.00	0.040	1054	-0.04	-0.09	0.01	0.084
Total lipids in chylomicrons and extremely large VLDL (mmol/l)	1076	-0.05	-0.10	-0.01	0.028	1076	-0.05	-0.10	0.00	0.036	1054	-0.05	-0.10	0.00	0.074
Phospholipids in chylomicrons and extremely large VLDL (mmol/l)	1076	-0.05	-0.10	-0.01	0.024	1076	-0.05	-0.10	-0.01	0.030	1054	-0.05	-0.10	0.00	0.061
Total cholesterol in chylomicrons and extremely large VLDL (mmol/l)	1076	-0.05	-0.10	0.00	0.033	1076	-0.05	-0.10	0.00	0.039	1054	-0.04	-0.09	0.01	0.094
Cholesterol esters in chylomicrons and extremely large VLDL (mmol/l)	1076	-0.05	-0.10	0.00	0.048	1076	-0.05	-0.10	0.00	0.055	1054	-0.04	-0.09	0.01	0.146
Free cholesterol in chylomicrons and extremely large VLDL (mmol/l)	1076	-0.05	-0.10	-0.01	0.026	1076	-0.05	-0.10	0.00	0.032	1054	-0.05	-0.10	0.00	0.064
Triglycerides in chylomicrons and extremely large VLDL (mmol/l)	1076	-0.05	-0.10	-0.01	0.029	1076	-0.05	-0.10	0.00	0.036	1054	-0.05	-0.09	0.00	0.074
Concentration of very large VLDL particles (mol/l)	1076	-0.05	-0.10	-0.01	0.028	1076	-0.05	-0.10	0.00	0.046	1054	-0.04	-0.09	0.01	0.091
Total lipids in very large VLDL (mmol/l)	1076	-0.05	-0.10	-0.01	0.027	1076	-0.05	-0.10	0.00	0.043	1054	-0.04	-0.09	0.01	0.085
Phospholipids in very large VLDL (mmol/l)	1076	-0.06	-0.10	-0.01	0.019	1076	-0.06	-0.10	-0.01	0.028	1054	-0.05	-0.10	0.00	0.058
Total cholesterol in very large VLDL (mmol/l)	1076	-0.05	-0.10	-0.01	0.026	1076	-0.05	-0.11	0.00	0.036	1054	-0.05	-0.10	0.01	0.083
Cholesterol esters in very large VLDL (mmol/l)	1076	-0.05	-0.10	0.00	0.032	1076	-0.05	-0.10	0.00	0.046	1054	-0.04	-0.09	0.01	0.107
Free cholesterol in very large VLDL (mmol/l)	1076	-0.05	-0.10	-0.01	0.022	1076	-0.06	-0.11	-0.01	0.028	1054	-0.05	-0.10	0.00	0.063
Triglycerides in very large VLDL (mmol/l)	1076	-0.05	-0.10	0.00	0.030	1076	-0.05	-0.10	0.00	0.051	1054	-0.04	-0.09	0.01	0.096
Concentration of large VLDL particles (mol/l)	1076	-0.05	-0.10	0.00	0.033	1076	-0.05	-0.10	0.00	0.054	1054	-0.04	-0.09	0.01	0.110
Total lipids in large VLDL (mmol/l)	1076	-0.05	-0.10	-0.01	0.030	1076	-0.05	-0.10	0.00	0.049	1054	-0.04	-0.10	0.01	0.102
Phospholipids in large VLDL (mmol/l)	1076	-0.05	-0.10	-0.01	0.026	1076	-0.05	-0.11	0.00	0.041	1054	-0.05	-0.10	0.01	0.088
Total cholesterol in large VLDL (mmol/l)	1076	-0.06	-0.11	-0.01	0.023	1076	-0.06	-0.11	0.00	0.039	1054	-0.05	-0.10	0.01	0.091
Cholesterol esters in large VLDL (mmol/l)	1076	-0.06	-0.11	-0.01	0.027	1076	-0.05	-0.11	0.00	0.045	1054	-0.04	-0.10	0.01	0.114
Free cholesterol in large VLDL (mmol/l)	1076	-0.06	-0.10	-0.01	0.020	1076	-0.05	-0.11	0.00	0.035	1054	-0.05	-0.10	0.00	0.075
Triglycerides in large VLDL (mmol/l)	1076	-0.05	-0.10	0.00	0.036	1076	-0.05	-0.10	0.00	0.058	1054	-0.04	-0.09	0.01	0.113
Concentration of medium VLDL particles (mol/l)	1076	-0.06	-0.11	-0.01	0.025	1076	-0.05	-0.11	0.00	0.044	1054	-0.05	-0.10	0.01	0.098
Total lipids in medium VLDL (mmol/l)	1076	-0.06	-0.11	-0.01	0.023	1076	-0.06	-0.11	0.00	0.041	1054	-0.05	-0.10	0.01	0.096
Phospholipids in medium VLDL (mmol/l)	1076	-0.06	-0.11	-0.01	0.018	1076	-0.06	-0.11	0.00	0.034	1054	-0.05	-0.10	0.01	0.081
Total cholesterol in medium VLDL (mmol/l)	1076	-0.06	-0.11	-0.01	0.016	1076	-0.06	-0.12	-0.01	0.030	1054	-0.05	-0.10	0.01	0.087
Cholesterol esters in medium VLDL (mmol/l)	1076	-0.06	-0.11	-0.01	0.020	1076	-0.06	-0.12	0.00	0.035	1054	-0.05	-0.10	0.01	0.109
Free cholesterol in medium VLDL (mmol/l)	1076	-0.06	-0.11	-0.01	0.017	1076	-0.06	-0.11	0.00	0.033	1054	-0.05	-0.10	0.01	0.079
Triglycerides in medium VLDL (mmol/l)	1076	-0.05	-0.10	0.00	0.036	1076	-0.05	-0.10	0.00	0.059	1054	-0.04	-0.10	0.01	0.117
Concentration of small VLDL particles (mol/l)	1076	-0.06	-0.12	-0.01	0.016	1076	-0.06	-0.11	0.00	0.033	1054	-0.05	-0.10	0.01	0.090
Total lipids in small VLDL (mmol/l)	1076	-0.07	-0.12	-0.01	0.014	1076	-0.06	-0.12	-0.01	0.028	1054	-0.05	-0.11	0.01	0.085
Phospholipids in small VLDL (mmol/l)	1076	-0.06	-0.12	-0.01	0.017	1076	-0.06	-0.12	-0.01	0.032	1054	-0.05	-0.10	0.01	0.099
Total cholesterol in small VLDL (mmol/l)	1076	-0.07	-0.13	-0.01	0.015	1076	-0.07	-0.13	-0.01	0.025	1054	-0.05	-0.11	0.01	0.087
Cholesterol esters in small VLDL (mmol/l)	1076	-0.07	-0.12	-0.01	0.022	1076	-0.07	-0.13	-0.01	0.031	1054	-0.05	-0.11	0.01	0.103
Free cholesterol in small VLDL (mmol/l)	1076	-0.07	-0.12	-0.01	0.012	1076	-0.06	-0.12	-0.01	0.028	1054	-0.05	-0.11	0.01	0.090
Triglycerides in small VLDL (mmol/l)	1076	-0.06	-0.11	0.00	0.033	1076	-0.05	-0.11	0.00	0.061	1054	-0.04	-0.10	0.01	0.129
Concentration of very small VLDL particles (mol/l)	1076	-0.06	-0.11	0.00	0.034	1076	-0.06	-0.11	0.00	0.055	1054	-0.04	-0.10	0.02	0.160
Total lipids in very small VLDL (mmol/l)	1076	-0.06	-0.12	-0.01	0.029	1076	-0.06	-0.12	0.00	0.042	1054	-0.05	-0.11	0.01	0.130
Phospholipids in very small VLDL (mmol/l)	1076	-0.05	-0.11	0.00	0.056	1076	-0.05	-0.11	0.00	0.066	1054	-0.04	-0.10	0.02	0.164
Total cholesterol in very small VLDL (mmol/l)	1076	-0.06	-0.11	0.00	0.052	1076	-0.06	-0.12	0.00	0.070	1054	-0.04	-0.10	0.02	0.190
Cholesterol esters in very small VLDL (mmol/l)	1076	-0.05	-0.11	0.00	0.068	1076	-0.05	-0.12	0.01	0.080	1054	-0.04	-0.10	0.02	0.205
Free cholesterol in very small VLDL (mmol/l)	1076	-0.06	-0.11	0.00	0.038	1076	-0.05	-0.11	0.00	0.066	1054	-0.04	-0.09	0.02	0.184
Triglycerides in very small VLDL (mmol/l)	1076	-0.05	-0.11	0.00	0.042	1076	-0.05	-0.10	0.01	0.075	1054	-0.04	-0.09	0.02	0.164
Concentration of IDL particles (mol/l)	1076	-0.05	-0.11	0.01	0.080	1076	-0.05	-0.11	0.01	0.092	1054	-0.04	-0.10	0.02	0.187
Total lipids in IDL (mmol/l)	1076	-0.05	-0.10	0.01	0.087	1076	-0.05	-0.11	0.01	0.096	1054	-0.04	-0.10	0.02	0.204
Phospholipids in IDL (mmol/l)	1076	-0.04	-0.10	0.01	0.126	1076	-0.05	-0.10	0.01	0.120	1054	-0.04	-0.10	0.02	0.224
Total cholesterol in IDL (mmol/l)	1076	-0.05	-0.11	0.01	0.083	1076	-0.05	-0.11	0.01	0.094	1054	-0.04	-0.10	0.02	0.207
Cholesterol esters in IDL (mmol/l)	1076	-0.05	-0.11	0.00	0.064	1076	-0.05	-0.11	0.01	0.078	1054	-0.04	-0.10	0.02	0.186

S10 Table Associations of change in moderate-to-vigorous physical activity (MVPA change from age 12y-15y) with metabolic traits at age 15y in ALSPAC

	Change in MVPA from age 12y-15y (per SD-unit increase)														
	Adj. for age, sex, ethnicity, maternal education change in wear time, wear month					*Additionally adj. for change in SED*					*Additionally adj. for change in FMI*				
**Standardised outcome at age 15y**	**N**	**Beta**	**LCL**	**UCL**	**P-value**	**N**	**Beta**	**LCL**	**UCL**	**P-value**	**N**	**Beta**	**LCL**	**UCL**	**P-value**
Free cholesterol in IDL (mmol/l)	1076	-0.04	-0.10	0.02	0.158	1076	-0.04	-0.10	0.02	0.161	1054	-0.03	-0.09	0.03	0.281
Triglycerides in IDL (mmol/l)	1076	-0.04	-0.09	0.02	0.186	1076	-0.03	-0.09	0.02	0.228	1054	-0.03	-0.08	0.03	0.340
Concentration of large LDL particles (mol/l)	1076	-0.04	-0.10	0.01	0.108	1076	-0.05	-0.11	0.01	0.105	1054	-0.04	-0.10	0.02	0.207
Total lipids in large LDL (mmol/l)	1076	-0.04	-0.10	0.01	0.116	1076	-0.05	-0.11	0.01	0.110	1054	-0.04	-0.10	0.02	0.221
Phospholipids in large LDL (mmol/l)	1076	-0.05	-0.10	0.01	0.112	1076	-0.05	-0.11	0.01	0.101	1054	-0.04	-0.10	0.02	0.212
Total cholesterol in large LDL (mmol/l)	1076	-0.04	-0.10	0.01	0.120	1076	-0.05	-0.11	0.01	0.116	1054	-0.04	-0.10	0.02	0.232
Cholesterol esters in large LDL (mmol/l)	1076	-0.05	-0.10	0.01	0.107	1076	-0.05	-0.11	0.01	0.103	1054	-0.04	-0.10	0.02	0.215
Free cholesterol in large LDL (mmol/l)	1076	-0.04	-0.09	0.02	0.173	1076	-0.04	-0.10	0.02	0.165	1054	-0.03	-0.09	0.03	0.296
Triglycerides in large LDL (mmol/l)	1076	-0.03	-0.09	0.02	0.228	1076	-0.03	-0.09	0.02	0.226	1054	-0.03	-0.08	0.03	0.307
Concentration of medium LDL particles (mol/l)	1076	-0.05	-0.10	0.01	0.097	1076	-0.05	-0.11	0.01	0.092	1054	-0.04	-0.10	0.02	0.186
Total lipids in medium LDL (mmol/l)	1076	-0.05	-0.10	0.01	0.100	1076	-0.05	-0.11	0.01	0.095	1054	-0.04	-0.10	0.02	0.196
Phospholipids in medium LDL (mmol/l)	1076	-0.05	-0.11	0.01	0.079	1076	-0.05	-0.11	0.01	0.077	1054	-0.04	-0.10	0.02	0.185
Total cholesterol in medium LDL (mmol/l)	1076	-0.05	-0.10	0.01	0.100	1076	-0.05	-0.11	0.01	0.096	1054	-0.04	-0.10	0.02	0.197
Cholesterol esters in medium LDL (mmol/l)	1076	-0.05	-0.10	0.01	0.097	1076	-0.05	-0.11	0.01	0.090	1054	-0.04	-0.10	0.02	0.183
Free cholesterol in medium LDL (mmol/l)	1076	-0.05	-0.10	0.01	0.114	1076	-0.05	-0.11	0.01	0.131	1054	-0.03	-0.09	0.03	0.268
Triglycerides in medium LDL (mmol/l)	1076	-0.02	-0.07	0.03	0.408	1076	-0.03	-0.08	0.03	0.356	1054	-0.02	-0.08	0.03	0.436
Concentration of small LDL particles (mol/l)	1076	-0.05	-0.10	0.01	0.076	1076	-0.05	-0.11	0.01	0.078	1054	-0.04	-0.10	0.02	0.159
Total lipids in small LDL (mmol/l)	1076	-0.05	-0.10	0.01	0.089	1076	-0.05	-0.11	0.01	0.086	1054	-0.04	-0.10	0.02	0.180
Phospholipids in small LDL (mmol/l)	1076	-0.05	-0.11	0.00	0.062	1076	-0.06	-0.11	0.00	0.063	1054	-0.04	-0.10	0.02	0.144
Total cholesterol in small LDL (mmol/l)	1076	-0.05	-0.10	0.01	0.108	1076	-0.05	-0.11	0.01	0.102	1054	-0.04	-0.10	0.02	0.205
Cholesterol esters in small LDL (mmol/l)	1076	-0.04	-0.10	0.01	0.119	1076	-0.05	-0.11	0.01	0.106	1054	-0.04	-0.10	0.02	0.205
Free cholesterol in small LDL (mmol/l)	1076	-0.05	-0.11	0.01	0.085	1076	-0.05	-0.11	0.01	0.102	1054	-0.04	-0.10	0.02	0.230
Triglycerides in small LDL (mmol/l)	1076	-0.04	-0.09	0.01	0.149	1076	-0.04	-0.09	0.01	0.145	1054	-0.03	-0.09	0.02	0.229
Concentration of very large HDL particles (mol/l)	1076	0.01	-0.05	0.07	0.749	1076	0.01	-0.05	0.07	0.817	1054	0.00	-0.06	0.07	0.922
Total lipids in very large HDL (mmol/l)	1076	0.01	-0.05	0.07	0.823	1076	0.00	-0.06	0.07	0.878	1054	0.00	-0.06	0.06	0.983
Phospholipids in very large HDL (mmol/l)	1076	0.02	-0.04	0.07	0.584	1076	0.01	-0.05	0.07	0.663	1054	0.01	-0.05	0.07	0.791
Total cholesterol in very large HDL (mmol/l)	1076	0.00	-0.06	0.06	0.963	1076	0.00	-0.06	0.06	0.934	1054	-0.01	-0.07	0.06	0.858
Cholesterol esters in very large HDL (mmol/l)	1076	0.00	-0.06	0.05	0.873	1076	-0.01	-0.07	0.06	0.858	1054	-0.01	-0.07	0.05	0.799
Free cholesterol in very large HDL (mmol/l)	1076	0.01	-0.05	0.07	0.809	1076	0.01	-0.06	0.07	0.873	1054	0.00	-0.06	0.06	0.985
Triglycerides in very large HDL (mmol/l)	1076	-0.05	-0.10	0.00	0.071	1076	-0.04	-0.10	0.01	0.110	1054	-0.04	-0.10	0.01	0.147
Concentration of large HDL particles (mol/l)	1076	0.01	-0.04	0.07	0.654	1076	0.01	-0.05	0.07	0.760	1054	0.00	-0.06	0.07	0.906
Total lipids in large HDL (mmol/l)	1076	0.02	-0.04	0.07	0.600	1076	0.01	-0.05	0.07	0.707	1054	0.01	-0.06	0.07	0.865
Phospholipids in large HDL (mmol/l)	1076	0.01	-0.05	0.07	0.695	1076	0.01	-0.05	0.07	0.829	1054	0.00	-0.06	0.06	0.971
Total cholesterol in large HDL (mmol/l)	1076	0.02	-0.04	0.08	0.477	1076	0.02	-0.04	0.08	0.562	1054	0.01	-0.05	0.07	0.728
Cholesterol esters in large HDL (mmol/l)	1076	0.02	-0.04	0.08	0.468	1076	0.02	-0.04	0.08	0.553	1054	0.01	-0.05	0.07	0.720
Free cholesterol in large HDL (mmol/l)	1076	0.02	-0.04	0.08	0.525	1076	0.02	-0.04	0.08	0.607	1054	0.01	-0.05	0.07	0.767
Triglycerides in large HDL (mmol/l)	1076	-0.05	-0.10	0.00	0.058	1076	-0.05	-0.11	0.00	0.061	1054	-0.05	-0.11	0.00	0.070
Concentration of medium HDL particles (mol/l)	1076	-0.01	-0.06	0.04	0.688	1076	-0.02	-0.08	0.04	0.498	1054	-0.02	-0.07	0.04	0.552
Total lipids in medium HDL (mmol/l)	1076	-0.01	-0.06	0.05	0.779	1076	-0.02	-0.07	0.04	0.569	1054	-0.02	-0.07	0.04	0.588
Phospholipids in medium HDL (mmol/l)	1076	-0.01	-0.06	0.05	0.778	1076	-0.02	-0.07	0.04	0.563	1054	-0.01	-0.07	0.04	0.621
Total cholesterol in medium HDL (mmol/l)	1076	0.00	-0.05	0.06	0.969	1076	-0.01	-0.06	0.05	0.819	1054	-0.01	-0.07	0.05	0.743
Cholesterol esters in medium HDL (mmol/l)	1076	0.00	-0.05	0.06	0.869	1076	0.00	-0.06	0.06	0.932	1054	-0.01	-0.07	0.05	0.834
Free cholesterol in medium HDL (mmol/l)	1076	-0.01	-0.07	0.04	0.641	1076	-0.02	-0.08	0.03	0.427	1054	-0.02	-0.08	0.03	0.432
Triglycerides in medium HDL (mmol/l)	1076	-0.05	-0.11	0.00	0.060	1076	-0.06	-0.11	0.00	0.054	1054	-0.05	-0.11	0.01	0.114
Concentration of small HDL particles (mol/l)	1076	-0.02	-0.07	0.04	0.555	1076	-0.03	-0.08	0.03	0.383	1054	-0.02	-0.08	0.04	0.467
Total lipids in small HDL (mmol/l)	1076	-0.01	-0.06	0.04	0.735	1076	-0.02	-0.07	0.04	0.517	1054	-0.01	-0.07	0.04	0.642
Phospholipids in small HDL (mmol/l)	1076	-0.02	-0.07	0.04	0.491	1076	-0.03	-0.08	0.03	0.368	1054	-0.03	-0.08	0.03	0.380
Total cholesterol in small HDL (mmol/l)	1076	0.01	-0.04	0.06	0.699	1076	0.00	-0.05	0.06	0.967	1054	0.01	-0.05	0.06	0.767
Cholesterol esters in small HDL (mmol/l)	1076	0.02	-0.04	0.07	0.564	1076	0.01	-0.05	0.06	0.830	1054	0.01	-0.04	0.07	0.613

S10 Table Associations of change in moderate-to-vigorous physical activity (MVPA change from age 12y-15y) with metabolic traits at age 15y in ALSPAC

Change in MVPA from age 12y-15y (per SD-unit increase)

	Adj. for age, sex, ethnicity, maternal education change in wear time, wear month					*Additionally adj. for change in SED*					*Additionally adj. for change in FMI*				
**Standardised outcome at age 15y**	**N**	**Beta**	**LCL**	**UCL**	**P-value**	**N**	**Beta**	**LCL**	**UCL**	**P-value**	**N**	**Beta**	**LCL**	**UCL**	**P-value**
Free cholesterol in small HDL (mmol/l)	1076	-0.01	-0.07	0.04	0.636	1076	-0.02	-0.07	0.04	0.542	1054	-0.02	-0.08	0.04	0.550
Triglycerides in small HDL (mmol/l)	1076	-0.04	-0.09	0.01	0.124	1076	-0.04	-0.10	0.01	0.133	1054	-0.03	-0.09	0.02	0.240
Phospholipids to total lipds ratio in chylomicrons and extremely large VLDL (%)	1076	-0.04	-0.10	0.01	0.150	1076	-0.04	-0.10	0.01	0.124	1054	-0.05	-0.10	0.01	0.116
Total cholesterol to total lipids ratio in chylomicrons and extremely large VLDL (%)	1076	-0.02	-0.07	0.04	0.583	1076	-0.02	-0.08	0.04	0.556	1054	-0.01	-0.07	0.05	0.786
Cholesterol esters to total lipids ratio in chylomicrons and extremely large VLDL (%)	1076	-0.01	-0.06	0.05	0.765	1076	-0.01	-0.07	0.05	0.738	1054	0.00	-0.06	0.06	0.999
Free cholesterol to total lipids ratio in chylomicrons and extremely large VLDL (%)	1076	-0.03	-0.09	0.03	0.303	1076	-0.03	-0.09	0.03	0.276	1054	-0.03	-0.09	0.03	0.343
Triglycerides to total lipids ratio in chylomicrons and extremely large VLDL (%)	1076	0.01	-0.04	0.05	0.812	1076	0.01	-0.04	0.05	0.750	1054	0.00	-0.04	0.05	0.941
Phospholipids to total lipds ratio in very large VLDL (%)	1076	-0.03	-0.08	0.03	0.358	1076	-0.03	-0.09	0.03	0.370	1054	-0.02	-0.08	0.04	0.508
Total cholesterol to total lipids ratio in very large VLDL (%)	1076	0.05	-0.08	0.18	0.480	1076	0.05	-0.08	0.18	0.442	1054	0.05	-0.08	0.17	0.463
Cholesterol esters to total lipids ratio in very large VLDL (%)	1076	-0.01	-0.07	0.06	0.850	1076	0.00	-0.06	0.06	0.959	1054	0.00	-0.07	0.06	0.883
Free cholesterol to total lipids ratio in very large VLDL (%)	1076	-0.01	-0.07	0.05	0.793	1076	0.00	-0.06	0.06	0.924	1054	0.00	-0.07	0.06	0.926
Triglycerides to total lipids ratio in very large VLDL (%)	1076	0.01	-0.05	0.08	0.669	1076	0.01	-0.06	0.07	0.790	1054	0.01	-0.06	0.07	0.797
Phospholipids to total lipds ratio in large VLDL (%)	1076	-0.01	-0.07	0.06	0.838	1076	-0.01	-0.07	0.05	0.793	1054	0.00	-0.06	0.06	0.988
Total cholesterol to total lipids ratio in large VLDL (%)	1076	-0.01	-0.07	0.06	0.841	1076	0.00	-0.06	0.07	0.978	1054	0.01	-0.06	0.08	0.787
Cholesterol esters to total lipids ratio in large VLDL (%)	1076	0.10	-0.12	0.32	0.359	1076	0.11	-0.11	0.32	0.332	1054	0.11	-0.10	0.31	0.317
Free cholesterol to total lipids ratio in large VLDL (%)	1076	-0.02	-0.08	0.04	0.452	1076	-0.02	-0.08	0.04	0.451	1054	-0.02	-0.08	0.04	0.606
Triglycerides to total lipids ratio in large VLDL (%)	1076	0.13	-0.12	0.38	0.299	1076	0.13	-0.12	0.37	0.302	1054	0.12	-0.11	0.36	0.306
Phospholipids to total lipds ratio in medium VLDL (%)	1076	0.01	-0.05	0.07	0.852	1076	0.02	-0.05	0.08	0.633	1054	0.02	-0.05	0.08	0.658
Total cholesterol to total lipids ratio in medium VLDL (%)	1076	-0.03	-0.09	0.02	0.215	1076	-0.03	-0.08	0.03	0.344	1054	-0.02	-0.07	0.04	0.578
Cholesterol esters to total lipids ratio in medium VLDL (%)	1076	-0.03	-0.08	0.02	0.269	1076	-0.02	-0.08	0.03	0.403	1054	-0.01	-0.07	0.04	0.653
Free cholesterol to total lipids ratio in medium VLDL (%)	1076	-0.03	-0.09	0.03	0.353	1076	-0.02	-0.08	0.04	0.482	1054	-0.02	-0.08	0.05	0.621
Triglycerides to total lipids ratio in medium VLDL (%)	1076	0.03	-0.03	0.08	0.303	1076	0.02	-0.04	0.08	0.495	1054	0.01	-0.05	0.07	0.738
Phospholipids to total lipds ratio in small VLDL (%)	1076	0.05	0.00	0.11	0.055	1076	0.05	-0.01	0.11	0.080	1054	0.04	-0.01	0.10	0.125
Total cholesterol to total lipids ratio in small VLDL (%)	1076	-0.03	-0.09	0.03	0.335	1076	-0.03	-0.09	0.03	0.357	1054	-0.02	-0.08	0.04	0.481
Cholesterol esters to total lipids ratio in small VLDL (%)	1076	-0.03	-0.09	0.03	0.330	1076	-0.03	-0.09	0.03	0.320	1054	-0.02	-0.09	0.04	0.430
Free cholesterol to total lipids ratio in small VLDL (%)	1076	0.00	-0.05	0.05	0.963	1076	0.01	-0.04	0.07	0.653	1054	0.02	-0.04	0.07	0.556
Triglycerides to total lipids ratio in small VLDL (%)	1076	0.01	-0.04	0.07	0.669	1076	0.01	-0.05	0.07	0.671	1054	0.01	-0.05	0.07	0.782
Phospholipids to total lipds ratio in very small VLDL (%)	1076	-0.03	-0.08	0.03	0.359	1076	-0.03	-0.09	0.03	0.310	1054	-0.02	-0.08	0.03	0.401
Total cholesterol to total lipids ratio in very small VLDL (%)	1076	0.01	-0.04	0.06	0.778	1076	0.01	-0.05	0.06	0.811	1054	0.01	-0.05	0.06	0.841
Cholesterol esters to total lipids ratio in very small VLDL (%)	1076	0.01	-0.05	0.06	0.759	1076	0.00	-0.05	0.06	0.881	1054	0.00	-0.05	0.06	0.935
Free cholesterol to total lipids ratio in very small VLDL (%)	1076	0.00	-0.04	0.04	0.924	1076	0.00	-0.04	0.05	0.821	1054	0.01	-0.04	0.05	0.764
Triglycerides to total lipids ratio in very small VLDL (%)	1076	0.01	-0.04	0.06	0.689	1076	0.01	-0.04	0.07	0.600	1054	0.01	-0.04	0.06	0.664
Phospholipids to total lipds ratio in IDL (%)	1076	0.05	0.00	0.10	0.033	1076	0.04	-0.01	0.09	0.155	1054	0.02	-0.03	0.08	0.344
Total cholesterol to total lipids ratio in IDL (%)	1076	-0.04	-0.09	0.01	0.129	1076	-0.04	-0.09	0.02	0.191	1054	-0.03	-0.09	0.03	0.288
Cholesterol esters to total lipids ratio in IDL (%)	1076	-0.04	-0.10	0.01	0.107	1076	-0.04	-0.09	0.02	0.184	1054	-0.03	-0.09	0.03	0.302
Free cholesterol to total lipids ratio in IDL (%)	1076	0.00	-0.04	0.05	0.863	1076	0.00	-0.05	0.05	0.995	1054	0.00	-0.05	0.05	0.910
Triglycerides to total lipids ratio in IDL (%)	1076	0.03	-0.03	0.08	0.335	1076	0.03	-0.03	0.08	0.313	1054	0.03	-0.03	0.08	0.366
Phospholipids to total lipds ratio in large LDL (%)	1076	0.05	0.00	0.10	0.073	1076	0.05	-0.01	0.10	0.095	1054	0.04	-0.02	0.09	0.168
Total cholesterol to total lipids ratio in large LDL (%)	1076	-0.05	-0.10	0.01	0.078	1076	-0.05	-0.10	0.01	0.099	1054	-0.04	-0.09	0.02	0.168
Cholesterol esters to total lipids ratio in large LDL (%)	1076	-0.06	-0.11	0.00	0.041	1076	-0.06	-0.11	0.00	0.050	1054	-0.05	-0.10	0.01	0.104
Free cholesterol to total lipids ratio in large LDL (%)	1076	0.05	0.00	0.10	0.060	1076	0.05	0.00	0.10	0.057	1054	0.04	-0.01	0.09	0.125
Triglycerides to total lipids ratio in large LDL (%)	1076	0.03	-0.03	0.08	0.328	1076	0.03	-0.03	0.08	0.345	1054	0.02	-0.03	0.08	0.421
Phospholipids to total lipds ratio in medium LDL (%)	1076	0.02	0.00	0.04	0.080	1076	0.02	0.00	0.04	0.085	1054	0.02	-0.01	0.04	0.137
Total cholesterol to total lipids ratio in medium LDL (%)	1076	-0.06	-0.11	0.00	0.043	1076	-0.06	-0.11	0.00	0.054	1054	-0.05	-0.11	0.01	0.090
Cholesterol esters to total lipids ratio in medium LDL (%)	1076	-0.06	-0.12	0.00	0.043	1076	-0.06	-0.12	0.00	0.044	1054	-0.05	-0.11	0.01	0.080
Free cholesterol to total lipids ratio in medium LDL (%)	1076	0.01	0.00	0.03	0.077	1076	0.02	0.00	0.03	0.060	1054	0.01	0.00	0.03	0.111
Triglycerides to total lipids ratio in medium LDL (%)	1076	0.03	-0.02	0.08	0.272	1076	0.03	-0.03	0.08	0.357	1054	0.02	-0.03	0.08	0.390
Phospholipids to total lipds ratio in small LDL (%)	1076	0.03	0.00	0.06	0.076	1076	0.03	0.00	0.06	0.080	1054	0.03	-0.01	0.06	0.146
Total cholesterol to total lipids ratio in small LDL (%)	1076	-0.05	-0.10	0.01	0.088	1076	-0.05	-0.11	0.01	0.097	1054	-0.04	-0.10	0.02	0.161

S10 Table Associations of change in moderate-to-vigorous physical activity (MVPA change from age 12y-15y) with metabolic traits at age 15y in ALSPAC

Change in MVPA from age 12y-15y (per SD-unit increase)

	Adj. for age, sex, ethnicity, maternal education change in wear time, wear month					*Additionally adj. for change in SED*					*Additionally adj. for change in FMI*				
**Standardised outcome at age 15y**	**N**	**Beta**	**LCL**	**UCL**	**P-value**	**N**	**Beta**	**LCL**	**UCL**	**P-value**	**N**	**Beta**	**LCL**	**UCL**	**P-value**
Cholesterol esters to total lipids ratio in small LDL (%)	1076	-0.05	-0.11	0.01	0.085	1076	-0.05	-0.11	0.01	0.080	1054	-0.04	-0.10	0.01	0.138
Free cholesterol to total lipids ratio in small LDL (%)	1076	0.02	-0.01	0.05	0.136	1076	0.03	0.00	0.06	0.101	1054	0.02	-0.01	0.05	0.161
Triglycerides to total lipids ratio in small LDL (%)	1076	0.01	-0.04	0.06	0.776	1076	0.01	-0.05	0.06	0.814	1054	0.01	-0.05	0.06	0.773
Phospholipids to total lipds ratio in very large HDL (%)	1076	0.03	-0.03	0.08	0.348	1076	0.02	-0.04	0.07	0.508	1054	0.01	-0.04	0.07	0.681
Total cholesterol to total lipids ratio in very large HDL (%)	1076	-0.02	-0.07	0.04	0.569	1076	-0.01	-0.07	0.05	0.729	1054	0.00	-0.06	0.05	0.890
Cholesterol esters to total lipids ratio in very large HDL (%)	1076	-0.02	-0.07	0.04	0.564	1076	-0.01	-0.07	0.05	0.724	1054	0.00	-0.06	0.05	0.887
Free cholesterol to total lipids ratio in very large HDL (%)	1076	0.01	-0.04	0.06	0.695	1076	0.01	-0.05	0.06	0.786	1054	0.00	-0.05	0.06	0.902
Triglycerides to total lipids ratio in very large HDL (%)	1076	-0.06	-0.11	-0.01	0.024	1076	-0.05	-0.11	0.00	0.054	1054	-0.05	-0.10	0.01	0.104
Phospholipids to total lipds ratio in large HDL (%)	1076	-0.03	-0.08	0.02	0.208	1076	-0.04	-0.09	0.02	0.172	1054	-0.03	-0.08	0.03	0.325
Total cholesterol to total lipids ratio in large HDL (%)	1076	0.04	-0.01	0.09	0.104	1076	0.04	-0.01	0.10	0.109	1054	0.03	-0.02	0.09	0.228
Cholesterol esters to total lipids ratio in large HDL (%)	1076	0.04	-0.01	0.10	0.103	1076	0.04	-0.01	0.10	0.110	1054	0.03	-0.02	0.09	0.234
Free cholesterol to total lipids ratio in large HDL (%)	1076	0.03	-0.02	0.08	0.250	1076	0.03	-0.02	0.09	0.244	1054	0.03	-0.03	0.08	0.373
Triglycerides to total lipids ratio in large HDL (%)	1076	-0.05	-0.10	0.00	0.062	1076	-0.05	-0.10	0.01	0.111	1054	-0.04	-0.09	0.02	0.213
Phospholipids to total lipds ratio in medium HDL (%)	1076	0.00	-0.06	0.05	0.905	1076	-0.01	-0.07	0.05	0.715	1054	0.00	-0.06	0.05	0.958
Total cholesterol to total lipids ratio in medium HDL (%)	1076	0.03	-0.03	0.08	0.316	1076	0.03	-0.02	0.09	0.241	1054	0.02	-0.04	0.08	0.473
Cholesterol esters to total lipids ratio in medium HDL (%)	1076	0.03	-0.02	0.09	0.224	1076	0.04	-0.01	0.10	0.140	1054	0.03	-0.03	0.09	0.317
Free cholesterol to total lipids ratio in medium HDL (%)	1076	-0.01	-0.07	0.05	0.691	1076	-0.02	-0.09	0.04	0.461	1054	-0.03	-0.09	0.04	0.409
Triglycerides to total lipids ratio in medium HDL (%)	1076	-0.05	-0.10	0.01	0.104	1076	-0.05	-0.11	0.01	0.116	1054	-0.04	-0.10	0.02	0.224
Phospholipids to total lipds ratio in small HDL (%)	1076	-0.02	-0.07	0.03	0.425	1076	-0.02	-0.07	0.04	0.556	1054	-0.03	-0.08	0.03	0.372
Total cholesterol to total lipids ratio in small HDL (%)	1076	0.03	-0.02	0.08	0.276	1076	0.02	-0.03	0.08	0.401	1054	0.03	-0.03	0.08	0.289
Cholesterol esters to total lipids ratio in small HDL (%)	1076	0.03	-0.02	0.08	0.276	1076	0.02	-0.03	0.08	0.424	1054	0.03	-0.02	0.08	0.287
Free cholesterol to total lipids ratio in small HDL (%)	1076	-0.01	-0.07	0.04	0.675	1076	0.00	-0.06	0.06	0.990	1054	-0.01	-0.07	0.05	0.673
Triglycerides to total lipids ratio in small HDL (%)	1076	-0.04	-0.09	0.01	0.158	1076	-0.03	-0.09	0.02	0.215	1054	-0.03	-0.08	0.03	0.340
Mean diameter for VLDL particles (nm)	1076	-0.03	-0.09	0.02	0.213	1076	-0.03	-0.09	0.02	0.243	1054	-0.03	-0.09	0.03	0.304
Mean diameter for LDL particles (nm)	1076	0.03	-0.02	0.07	0.287	1076	0.03	-0.02	0.08	0.267	1054	0.02	-0.03	0.07	0.355
Mean diameter for HDL particles (nm)	1076	0.01	-0.05	0.07	0.677	1076	0.01	-0.05	0.07	0.723	1054	0.01	-0.06	0.07	0.872
Serum total cholesterol (mmol/l)	1076	-0.05	-0.10	0.01	0.104	1076	-0.05	-0.11	0.01	0.100	1054	-0.04	-0.10	0.02	0.200
Total cholesterol in VLDL (mmol/l)	1076	-0.07	-0.12	-0.02	0.010	1076	-0.07	-0.13	-0.01	0.018	1054	-0.05	-0.11	0.00	0.068
Remnant cholesterol (non-HDL, non-LDL -cholesterol) (mmol/l)	1076	-0.07	-0.12	-0.01	0.017	1076	-0.07	-0.13	-0.01	0.025	1054	-0.05	-0.11	0.01	0.084
Total cholesterol in LDL (mmol/l)	1076	-0.05	-0.10	0.01	0.111	1076	-0.05	-0.11	0.01	0.107	1054	-0.04	-0.10	0.02	0.216
Total cholesterol in HDL (mmol/l)	1076	0.01	-0.05	0.07	0.686	1076	0.01	-0.05	0.07	0.832	1054	0.00	-0.06	0.06	0.916
Total cholesterol in HDL2 (mmol/l)	1076	0.02	-0.04	0.08	0.469	1076	0.01	-0.05	0.08	0.628	1054	0.01	-0.05	0.07	0.745
Total cholesterol in HDL3 (mmol/l)	1076	-0.01	-0.06	0.05	0.849	1076	-0.01	-0.07	0.05	0.774	1054	-0.01	-0.07	0.05	0.768
Esterified cholesterol (mmol/l)	1076	-0.04	-0.10	0.01	0.108	1076	-0.05	-0.11	0.01	0.091	1054	-0.04	-0.10	0.02	0.188
Free cholesterol (mmol/l)	1076	-0.05	-0.10	0.01	0.113	1076	-0.05	-0.11	0.02	0.143	1054	-0.04	-0.10	0.03	0.255
Serum total triglycerides (mmol/l)	1076	-0.06	-0.11	-0.01	0.024	1076	-0.06	-0.11	0.00	0.038	1054	-0.05	-0.10	0.01	0.085
Triglycerides in VLDL (mmol/l)	1076	-0.05	-0.10	-0.01	0.030	1076	-0.05	-0.10	0.00	0.051	1054	-0.04	-0.10	0.01	0.106
Triglycerides in LDL (mmol/l)	1076	-0.03	-0.08	0.02	0.253	1076	-0.03	-0.09	0.02	0.238	1054	-0.03	-0.08	0.03	0.322
Triglycerides in HDL (mmol/l)	1076	-0.06	-0.11	-0.01	0.028	1076	-0.06	-0.11	-0.01	0.030	1054	-0.05	-0.11	0.00	0.066
Diacylglycerol (mmol/l)	1044	-0.02	-0.07	0.04	0.573	1044	-0.02	-0.08	0.04	0.499	1023	-0.01	-0.07	0.05	0.690
Ratio of diacylglycerol to triglycerides	1044	0.02	-0.04	0.08	0.434	1044	0.02	-0.05	0.08	0.622	1023	0.02	-0.05	0.08	0.591
Total phosphoglycerides (mmol/l)	1076	-0.03	-0.09	0.02	0.272	1076	-0.03	-0.09	0.03	0.309	1054	-0.02	-0.08	0.04	0.437
Ratio of triglycerides to phosphoglycerides	1076	-0.03	-0.08	0.02	0.269	1076	-0.03	-0.08	0.02	0.295	1054	-0.02	-0.08	0.03	0.387
Phosphatidylcholine and other cholines (mmol/l)	1056	-0.05	-0.11	0.00	0.070	1056	-0.05	-0.11	0.01	0.108	1034	-0.05	-0.11	0.02	0.142
Total cholines (mmol/l)	1073	-0.03	-0.08	0.03	0.315	1073	-0.03	-0.09	0.03	0.367	1051	-0.02	-0.08	0.04	0.466
Apolipoprotein A-I (g/l)	1076	-0.01	-0.07	0.04	0.677	1076	-0.02	-0.08	0.04	0.572	1054	-0.02	-0.08	0.04	0.593
Apolipoprotein B (g/l)	1076	-0.07	-0.12	-0.01	0.015	1076	-0.07	-0.12	-0.01	0.024	1054	-0.05	-0.11	0.01	0.076
Ratio of apolipoprotein B to apolipoprotein A-I	1076	-0.06	-0.12	0.00	0.037	1076	-0.06	-0.12	0.00	0.069	1054	-0.04	-0.10	0.02	0.176
Total fatty acids (mmol/l)	1076	-0.05	-0.11	0.00	0.065	1076	-0.05	-0.11	0.01	0.075	1054	-0.04	-0.10	0.02	0.167

S10 Table Associations of change in moderate-to-vigorous physical activity (MVPA change from age 12y-15y) with metabolic traits at age 15y in ALSPAC

Change in MVPA from age 12y-15y (per SD-unit increase)

	Adj. for age, sex, ethnicity, maternal education change in wear time, wear month					*Additionally adj. for change in SED*					*Additionally adj. for change in FMI*				
**Standardised outcome at age 15y**	**N**	**Beta**	**LCL**	**UCL**	**P-value**	**N**	**Beta**	**LCL**	**UCL**	**P-value**	**N**	**Beta**	**LCL**	**UCL**	**P-value**
Estimated description of fatty acid chain length, not actual carbon number	1072	0.01	-0.05	0.06	0.737	1072	0.01	-0.05	0.06	0.811	1050	0.00	-0.05	0.06	0.881
Estimated degree of unsaturation	1075	0.01	-0.04	0.07	0.680	1075	0.01	-0.05	0.06	0.846	1053	0.00	-0.06	0.06	0.934
22:6, docosahexaenoic acid (mmol/l)	1076	-0.03	-0.08	0.03	0.318	1076	-0.04	-0.10	0.01	0.131	1054	-0.04	-0.09	0.02	0.215
18:2, linoleic acid (mmol/l)	1073	-0.03	-0.08	0.03	0.336	1073	-0.02	-0.08	0.03	0.412	1051	-0.02	-0.08	0.04	0.545
Conjugated linoleic acid (mmol/l)	1076	0.00	-0.06	0.06	0.939	1076	0.00	-0.07	0.06	0.900	1054	0.00	-0.06	0.06	0.912
Omega-3 fatty acids (mmol/l)	1074	-0.02	-0.08	0.04	0.571	1074	-0.04	-0.10	0.03	0.269	1052	-0.03	-0.09	0.03	0.383
Omega-6 fatty acids (mmol/l)	1075	-0.03	-0.09	0.02	0.269	1075	-0.03	-0.09	0.03	0.294	1053	-0.02	-0.08	0.03	0.423
Polyunsaturated fatty acids (mmol/l)	1073	-0.03	-0.08	0.03	0.312	1073	-0.03	-0.09	0.03	0.286	1051	-0.02	-0.08	0.03	0.418
Monounsaturated fatty acids; 16:1, 18:1 (mmol/l)	1073	-0.06	-0.12	-0.01	0.021	1073	-0.06	-0.11	0.00	0.039	1051	-0.05	-0.10	0.01	0.096
Saturated fatty acids (mmol/l)	1072	-0.04	-0.10	0.01	0.103	1072	-0.05	-0.10	0.01	0.100	1050	-0.04	-0.09	0.02	0.219
Ratio of 22:6 docosahexaenoic acid to total fatty acids (%)	1076	-0.01	-0.06	0.04	0.719	1076	-0.03	-0.08	0.03	0.311	1054	-0.02	-0.08	0.03	0.380
Ratio of 18:2 linoleic acid to total fatty acids (%)	1073	0.03	-0.03	0.09	0.322	1073	0.04	-0.03	0.10	0.253	1051	0.03	-0.03	0.09	0.366
Ratio of conjugated linoleic acid to total fatty acids (%)	1076	0.01	-0.05	0.07	0.805	1076	0.01	-0.06	0.07	0.853	1054	0.01	-0.05	0.07	0.718
Ratio of omega-3 fatty acids to total fatty acids (%)	1074	0.01	-0.05	0.07	0.796	1074	-0.01	-0.07	0.05	0.681	1052	-0.01	-0.07	0.05	0.733
Ratio of omega-6 fatty acids to total fatty acids (%)	1075	0.04	-0.02	0.10	0.209	1075	0.04	-0.02	0.10	0.210	1053	0.03	-0.03	0.09	0.333
Ratio of polyunsaturated fatty acids to total fatty acids (%)	1073	0.03	-0.02	0.09	0.259	1073	0.03	-0.03	0.09	0.319	1051	0.02	-0.04	0.08	0.456
Ratio of monounsaturated fatty acids to total fatty acids (%)	1073	-0.04	-0.10	0.02	0.147	1073	-0.04	-0.10	0.02	0.239	1051	-0.03	-0.09	0.03	0.322
Ratio of saturated fatty acids to total fatty acids (%)	1072	0.01	-0.04	0.07	0.618	1072	0.01	-0.05	0.07	0.746	1050	0.01	-0.05	0.07	0.676
Insulin (mu/l)	1118	0.02	-0.02	0.05	0.365	1118	0.01	-0.02	0.05	0.484	1095	0.02	-0.02	0.06	0.290
Glucose (mmol/l)	1074	-0.01	-0.06	0.04	0.691	1074	-0.01	-0.07	0.05	0.722	1052	0.00	-0.05	0.05	0.997
Lactate (mmol/l)	1074	0.00	-0.06	0.05	0.896	1074	0.00	-0.06	0.06	0.979	1052	0.00	-0.06	0.06	0.945
Pyruvate (mmol/l)	1073	0.04	-0.02	0.10	0.218	1073	0.04	-0.02	0.10	0.226	1051	0.04	-0.02	0.10	0.201
Citrate (mmol/l)	1070	0.01	-0.06	0.07	0.850	1070	0.01	-0.06	0.07	0.793	1048	0.01	-0.05	0.08	0.714
Alanine (mmol/l)	1076	0.03	-0.02	0.09	0.255	1076	0.04	-0.02	0.10	0.167	1054	0.04	-0.02	0.10	0.181
Glutamine (mmol/l)	1076	0.03	-0.02	0.08	0.278	1076	0.02	-0.03	0.07	0.474	1054	0.02	-0.04	0.07	0.568
Histidine (mmol/l)	1015	-0.02	-0.08	0.04	0.461	1015	-0.01	-0.07	0.05	0.748	994	-0.01	-0.07	0.05	0.778
Isoleucine (mmol/l)	1076	0.00	-0.06	0.05	0.955	1076	-0.01	-0.06	0.05	0.803	1054	0.00	-0.06	0.06	0.989
Leucine (mmol/l)	1076	0.03	-0.03	0.08	0.325	1076	0.02	-0.03	0.07	0.480	1054	0.02	-0.03	0.08	0.372
Valine (mmol/l)	1076	0.00	-0.05	0.06	0.977	1076	0.00	-0.06	0.05	0.881	1054	0.00	-0.05	0.06	0.904
Phenylalanine (mmol/l)	1075	0.02	-0.04	0.08	0.514	1075	-0.01	-0.07	0.06	0.863	1053	0.00	-0.07	0.06	0.917
Tyrosine (mmol/l)	1070	-0.02	-0.07	0.04	0.614	1070	-0.03	-0.10	0.03	0.263	1048	-0.03	-0.09	0.03	0.344
Acetate (mmol/l)	1075	0.00	-0.05	0.06	0.930	1075	0.01	-0.04	0.07	0.615	1053	0.02	-0.04	0.07	0.549
Acetoacetate (mmol/l)	1076	0.07	-0.02	0.16	0.149	1076	0.06	-0.03	0.15	0.171	1054	0.06	-0.03	0.16	0.185
3-hydroxybutyrate (mmol/l)	1075	0.02	-0.03	0.08	0.418	1075	0.02	-0.04	0.07	0.590	1053	0.02	-0.04	0.08	0.541
Creatinine (mmol/l)	1075	-0.02	-0.07	0.04	0.583	1075	-0.01	-0.07	0.05	0.808	1053	-0.01	-0.07	0.05	0.805
Albumin (signal area)	1076	-0.02	-0.07	0.04	0.541	1076	-0.02	-0.08	0.04	0.539	1054	-0.02	-0.09	0.04	0.427
Glycoprotein acetyls, mainly a1-acid glycoprotein (mmol/l)	1075	-0.03	-0.08	0.02	0.222	1075	-0.05	-0.11	0.01	0.076	1053	-0.05	-0.10	0.01	0.117
C-reactive protein (mg/l)	1120	0.00	-0.03	0.02	0.829	1120	-0.01	-0.06	0.03	0.518	1097	-0.02	-0.06	0.03	0.526

Change in MVPA from age 12y-15y (per SD-unit increase)

Complete case sample

	Adj. for age, sex, ethnicity, maternal education change in wear time, wear month					*Additionally adj. for change in SED*					*Additionally adj. for change in FMI*				
**Standardised outcome at age 15y**	**N**	**Beta**	**LCL**	**UCL**	**P-value**	**N**	**Beta**	**LCL**	**UCL**	**P-value**	**N**	**Beta**	**LCL**	**UCL**	**P-value**
Systolic blood pressure (mmHg)	755	0.06	0.00	0.13	0.056	755	0.07	0.01	0.14	0.023	755	0.08	0.01	0.14	0.021
Diastolic blood pressure (mmHg)	755	0.02	-0.05	0.08	0.587	755	0.02	-0.04	0.09	0.482	755	0.02	-0.04	0.09	0.487

S10 Table Associations of change in moderate-to-vigorous physical activity (MVPA change from age 12y-15y) with metabolic traits at age 15y in ALSPAC

Change in MVPA from age 12y-15y (per SD-unit increase)

	Adj. for age, sex, ethnicity, maternal education change in wear time, wear month					*Additionally adj. for change in SED*					*Additionally adj. for change in FMI*				
**Standardised outcome at age 15y**	**N**	**Beta**	**LCL**	**UCL**	**P-value**	**N**	**Beta**	**LCL**	**UCL**	**P-value**	**N**	**Beta**	**LCL**	**UCL**	**P-value**
Concentration of chylomicrons and extremely large VLDL particles (mol/l)	755	-0.03	-0.09	0.03	0.289	755	-0.03	-0.09	0.03	0.358	755	-0.02	-0.08	0.04	0.619
Total lipids in chylomicrons and extremely large VLDL (mmol/l)	755	-0.03	-0.09	0.02	0.245	755	-0.03	-0.09	0.03	0.293	755	-0.02	-0.08	0.04	0.524
Phospholipids in chylomicrons and extremely large VLDL (mmol/l)	755	-0.04	-0.09	0.02	0.218	755	-0.03	-0.09	0.03	0.263	755	-0.02	-0.08	0.04	0.476
Total cholesterol in chylomicrons and extremely large VLDL (mmol/l)	755	-0.03	-0.09	0.03	0.367	755	-0.02	-0.09	0.04	0.439	755	-0.01	-0.07	0.05	0.734
Cholesterol esters in chylomicrons and extremely large VLDL (mmol/l)	755	-0.02	-0.08	0.04	0.511	755	-0.02	-0.08	0.05	0.615	755	0.00	-0.07	0.06	0.962
Free cholesterol in chylomicrons and extremely large VLDL (mmol/l)	755	-0.03	-0.09	0.02	0.251	755	-0.03	-0.09	0.03	0.291	755	-0.02	-0.08	0.04	0.509
Triglycerides in chylomicrons and extremely large VLDL (mmol/l)	755	-0.03	-0.09	0.02	0.226	755	-0.03	-0.09	0.03	0.270	755	-0.02	-0.08	0.04	0.488
Concentration of very large VLDL particles (mol/l)	755	-0.03	-0.09	0.02	0.247	755	-0.03	-0.09	0.03	0.331	755	-0.02	-0.08	0.04	0.564
Total lipids in very large VLDL (mmol/l)	755	-0.03	-0.09	0.02	0.236	755	-0.03	-0.09	0.03	0.312	755	-0.02	-0.08	0.04	0.538
Phospholipids in very large VLDL (mmol/l)	755	-0.03	-0.09	0.02	0.247	755	-0.03	-0.09	0.03	0.315	755	-0.02	-0.08	0.04	0.542
Total cholesterol in very large VLDL (mmol/l)	755	-0.03	-0.09	0.03	0.300	755	-0.03	-0.09	0.03	0.375	755	-0.01	-0.08	0.05	0.642
Cholesterol esters in very large VLDL (mmol/l)	755	-0.03	-0.09	0.03	0.323	755	-0.03	-0.09	0.04	0.410	755	-0.01	-0.08	0.05	0.690
Free cholesterol in very large VLDL (mmol/l)	755	-0.03	-0.09	0.03	0.279	755	-0.03	-0.09	0.03	0.341	755	-0.02	-0.08	0.04	0.590
Triglycerides in very large VLDL (mmol/l)	755	-0.04	-0.09	0.02	0.218	755	-0.03	-0.09	0.03	0.296	755	-0.02	-0.08	0.04	0.509
Concentration of large VLDL particles (mol/l)	755	-0.04	-0.10	0.02	0.215	755	-0.03	-0.10	0.03	0.295	755	-0.02	-0.08	0.04	0.511
Total lipids in large VLDL (mmol/l)	755	-0.04	-0.10	0.02	0.219	755	-0.03	-0.10	0.03	0.299	755	-0.02	-0.08	0.04	0.518
Phospholipids in large VLDL (mmol/l)	755	-0.04	-0.10	0.02	0.223	755	-0.03	-0.10	0.03	0.305	755	-0.02	-0.08	0.04	0.528
Total cholesterol in large VLDL (mmol/l)	755	-0.03	-0.09	0.03	0.262	755	-0.03	-0.09	0.03	0.375	755	-0.02	-0.08	0.05	0.634
Cholesterol esters in large VLDL (mmol/l)	755	-0.03	-0.09	0.03	0.311	755	-0.03	-0.09	0.04	0.445	755	-0.01	-0.08	0.05	0.728
Free cholesterol in large VLDL (mmol/l)	755	-0.04	-0.09	0.02	0.223	755	-0.03	-0.09	0.03	0.317	755	-0.02	-0.08	0.04	0.550
Triglycerides in large VLDL (mmol/l)	755	-0.04	-0.10	0.02	0.204	755	-0.04	-0.10	0.03	0.273	755	-0.02	-0.09	0.04	0.475
Concentration of medium VLDL particles (mol/l)	755	-0.04	-0.10	0.02	0.206	755	-0.03	-0.10	0.03	0.304	755	-0.02	-0.09	0.04	0.527
Total lipids in medium VLDL (mmol/l)	755	-0.04	-0.10	0.02	0.222	755	-0.03	-0.10	0.03	0.327	755	-0.02	-0.08	0.05	0.563
Phospholipids in medium VLDL (mmol/l)	755	-0.04	-0.10	0.02	0.215	755	-0.03	-0.10	0.03	0.338	755	-0.02	-0.08	0.05	0.583
Total cholesterol in medium VLDL (mmol/l)	755	-0.03	-0.09	0.03	0.311	755	-0.02	-0.09	0.04	0.484	755	-0.01	-0.08	0.06	0.788
Cholesterol esters in medium VLDL (mmol/l)	755	-0.02	-0.09	0.04	0.432	755	-0.02	-0.08	0.05	0.648	755	0.00	-0.07	0.07	0.981
Free cholesterol in medium VLDL (mmol/l)	755	-0.04	-0.10	0.02	0.215	755	-0.03	-0.10	0.03	0.336	755	-0.02	-0.08	0.05	0.579
Triglycerides in medium VLDL (mmol/l)	755	-0.04	-0.10	0.02	0.199	755	-0.04	-0.10	0.03	0.270	755	-0.02	-0.09	0.04	0.467
Concentration of small VLDL particles (mol/l)	755	-0.04	-0.10	0.02	0.240	755	-0.03	-0.09	0.04	0.411	755	-0.01	-0.08	0.05	0.684
Total lipids in small VLDL (mmol/l)	755	-0.04	-0.10	0.03	0.252	755	-0.03	-0.09	0.04	0.434	755	-0.01	-0.08	0.05	0.728
Phospholipids in small VLDL (mmol/l)	755	-0.04	-0.10	0.03	0.262	755	-0.02	-0.09	0.04	0.467	755	-0.01	-0.08	0.06	0.765
Total cholesterol in small VLDL (mmol/l)	755	-0.03	-0.10	0.03	0.309	755	-0.02	-0.09	0.05	0.543	755	-0.01	-0.07	0.06	0.882
Cholesterol esters in small VLDL (mmol/l)	755	-0.03	-0.09	0.03	0.366	755	-0.02	-0.09	0.05	0.582	755	0.00	-0.07	0.06	0.919
Free cholesterol in small VLDL (mmol/l)	755	-0.04	-0.10	0.03	0.263	755	-0.02	-0.09	0.04	0.519	755	-0.01	-0.07	0.06	0.842
Triglycerides in small VLDL (mmol/l)	755	-0.03	-0.10	0.03	0.262	755	-0.03	-0.09	0.04	0.390	755	-0.02	-0.08	0.05	0.619
Concentration of very small VLDL particles (mol/l)	755	-0.02	-0.08	0.05	0.590	755	0.00	-0.07	0.06	0.934	755	0.01	-0.06	0.08	0.770
Total lipids in very small VLDL (mmol/l)	755	-0.02	-0.09	0.04	0.439	755	-0.01	-0.08	0.05	0.703	755	0.00	-0.07	0.07	0.984
Phospholipids in very small VLDL (mmol/l)	755	-0.01	-0.08	0.05	0.706	755	0.00	-0.07	0.07	0.959	755	0.01	-0.06	0.08	0.782
Total cholesterol in very small VLDL (mmol/l)	755	-0.03	-0.09	0.03	0.370	755	-0.02	-0.08	0.05	0.585	755	-0.01	-0.07	0.06	0.878
Cholesterol esters in very small VLDL (mmol/l)	755	-0.03	-0.09	0.03	0.359	755	-0.02	-0.09	0.05	0.524	755	-0.01	-0.08	0.06	0.805
Free cholesterol in very small VLDL (mmol/l)	755	-0.02	-0.08	0.03	0.424	755	-0.01	-0.07	0.05	0.748	755	0.00	-0.06	0.06	0.966
Triglycerides in very small VLDL (mmol/l)	755	-0.02	-0.08	0.04	0.525	755	-0.01	-0.07	0.05	0.790	755	0.00	-0.06	0.06	0.957
Concentration of IDL particles (mol/l)	755	-0.01	-0.07	0.05	0.737	755	0.00	-0.07	0.07	0.968	755	0.01	-0.06	0.08	0.812
Total lipids in IDL (mmol/l)	755	-0.01	-0.07	0.05	0.771	755	0.00	-0.07	0.07	0.984	755	0.01	-0.06	0.08	0.749
Phospholipids in IDL (mmol/l)	755	-0.01	-0.07	0.06	0.874	755	0.00	-0.06	0.07	0.950	755	0.01	-0.06	0.08	0.752
Total cholesterol in IDL (mmol/l)	755	-0.01	-0.08	0.05	0.711	755	0.00	-0.07	0.07	0.971	755	0.01	-0.06	0.08	0.772
Cholesterol esters in IDL (mmol/l)	755	-0.02	-0.08	0.05	0.634	755	0.00	-0.07	0.06	0.911	755	0.01	-0.06	0.08	0.805
Free cholesterol in IDL (mmol/l)	755	0.00	-0.07	0.06	0.908	755	0.00	-0.06	0.07	0.892	755	0.01	-0.05	0.08	0.710
Triglycerides in IDL (mmol/l)	755	0.00	-0.06	0.06	0.991	755	0.01	-0.05	0.07	0.781	755	0.01	-0.05	0.08	0.672

S10 Table Associations of change in moderate-to-vigorous physical activity (MVPA change from age 12y-15y) with metabolic traits at age 15y in ALSPAC

Change in MVPA from age 12y-15y (per SD-unit increase)

	Adj. for age, sex, ethnicity, maternal education change in wear time, wear month					*Additionally adj. for change in SED*					*Additionally adj. for change in FMI*				
**Standardised outcome at age 15y**	**N**	**Beta**	**LCL**	**UCL**	**P-value**	**N**	**Beta**	**LCL**	**UCL**	**P-value**	**N**	**Beta**	**LCL**	**UCL**	**P-value**
Concentration of large LDL particles (mol/l)	755	-0.01	-0.07	0.06	0.813	755	0.00	-0.07	0.07	0.991	755	0.01	-0.06	0.08	0.768
Total lipids in large LDL (mmol/l)	755	-0.01	-0.07	0.06	0.851	755	0.00	-0.07	0.07	0.958	755	0.01	-0.06	0.08	0.732
Phospholipids in large LDL (mmol/l)	755	-0.01	-0.07	0.06	0.856	755	0.00	-0.07	0.07	0.951	755	0.01	-0.05	0.08	0.709
Total cholesterol in large LDL (mmol/l)	755	-0.01	-0.07	0.06	0.846	755	0.00	-0.07	0.07	0.957	755	0.01	-0.06	0.08	0.725
Cholesterol esters in large LDL (mmol/l)	755	-0.01	-0.07	0.06	0.819	755	0.00	-0.07	0.07	0.983	755	0.01	-0.06	0.08	0.739
Free cholesterol in large LDL (mmol/l)	755	0.00	-0.07	0.06	0.927	755	0.01	-0.06	0.07	0.882	755	0.01	-0.05	0.08	0.689
Triglycerides in large LDL (mmol/l)	755	0.00	-0.06	0.06	0.947	755	0.00	-0.06	0.06	0.962	755	0.00	-0.06	0.07	0.885
Concentration of medium LDL particles (mol/l)	755	-0.01	-0.07	0.05	0.774	755	0.00	-0.07	0.06	0.958	755	0.01	-0.06	0.07	0.804
Total lipids in medium LDL (mmol/l)	755	-0.01	-0.07	0.05	0.786	755	0.00	-0.07	0.07	0.971	755	0.01	-0.06	0.08	0.788
Phospholipids in medium LDL (mmol/l)	755	-0.01	-0.08	0.05	0.717	755	0.00	-0.07	0.07	0.959	755	0.01	-0.06	0.08	0.758
Total cholesterol in medium LDL (mmol/l)	755	-0.01	-0.07	0.05	0.770	755	0.00	-0.07	0.07	0.953	755	0.01	-0.06	0.08	0.805
Cholesterol esters in medium LDL (mmol/l)	755	-0.01	-0.07	0.05	0.783	755	0.00	-0.07	0.06	0.936	755	0.01	-0.06	0.07	0.825
Free cholesterol in medium LDL (mmol/l)	755	-0.01	-0.08	0.05	0.705	755	0.00	-0.07	0.07	0.994	755	0.01	-0.06	0.08	0.743
Triglycerides in medium LDL (mmol/l)	755	0.01	-0.05	0.07	0.842	755	0.01	-0.06	0.07	0.834	755	0.01	-0.05	0.07	0.777
Concentration of small LDL particles (mol/l)	755	-0.02	-0.08	0.05	0.582	755	-0.01	-0.08	0.06	0.776	755	0.00	-0.07	0.07	0.978
Total lipids in small LDL (mmol/l)	755	-0.01	-0.08	0.05	0.690	755	-0.01	-0.07	0.06	0.871	755	0.01	-0.06	0.07	0.877
Phospholipids in small LDL (mmol/l)	755	-0.02	-0.08	0.04	0.536	755	-0.01	-0.08	0.06	0.756	755	0.00	-0.07	0.07	0.965
Total cholesterol in small LDL (mmol/l)	755	-0.01	-0.07	0.05	0.738	755	0.00	-0.07	0.06	0.909	755	0.01	-0.06	0.07	0.849
Cholesterol esters in small LDL (mmol/l)	755	-0.01	-0.07	0.06	0.807	755	0.00	-0.07	0.06	0.944	755	0.01	-0.06	0.07	0.828
Free cholesterol in small LDL (mmol/l)	755	-0.02	-0.09	0.04	0.491	755	-0.01	-0.08	0.06	0.779	755	0.00	-0.07	0.07	0.939
Triglycerides in small LDL (mmol/l)	755	-0.01	-0.07	0.05	0.837	755	0.00	-0.06	0.06	0.929	755	0.00	-0.06	0.06	0.899
Concentration of very large HDL particles (mol/l)	755	0.02	-0.06	0.09	0.661	755	0.02	-0.06	0.09	0.685	755	0.01	-0.07	0.09	0.766
Total lipids in very large HDL (mmol/l)	755	0.01	-0.06	0.08	0.792	755	0.01	-0.07	0.09	0.815	755	0.01	-0.07	0.08	0.886
Phospholipids in very large HDL (mmol/l)	755	0.03	-0.05	0.10	0.482	755	0.02	-0.05	0.10	0.524	755	0.02	-0.06	0.09	0.624
Total cholesterol in very large HDL (mmol/l)	755	-0.01	-0.08	0.06	0.825	755	-0.01	-0.08	0.07	0.829	755	-0.01	-0.09	0.07	0.804
Cholesterol esters in very large HDL (mmol/l)	755	-0.01	-0.09	0.06	0.685	755	-0.01	-0.09	0.06	0.697	755	-0.02	-0.09	0.06	0.691
Free cholesterol in very large HDL (mmol/l)	755	0.01	-0.06	0.08	0.804	755	0.01	-0.07	0.08	0.823	755	0.01	-0.07	0.08	0.896
Triglycerides in very large HDL (mmol/l)	755	-0.02	-0.09	0.04	0.541	755	-0.02	-0.08	0.05	0.653	755	-0.01	-0.08	0.06	0.753
Concentration of large HDL particles (mol/l)	755	0.02	-0.05	0.09	0.543	755	0.02	-0.05	0.10	0.573	755	0.02	-0.06	0.09	0.682
Total lipids in large HDL (mmol/l)	755	0.02	-0.05	0.09	0.506	755	0.02	-0.05	0.10	0.542	755	0.02	-0.06	0.09	0.658
Phospholipids in large HDL (mmol/l)	755	0.02	-0.05	0.09	0.515	755	0.02	-0.05	0.10	0.560	755	0.02	-0.06	0.09	0.660
Total cholesterol in large HDL (mmol/l)	755	0.03	-0.04	0.10	0.478	755	0.03	-0.05	0.10	0.509	755	0.02	-0.06	0.09	0.639
Cholesterol esters in large HDL (mmol/l)	755	0.03	-0.04	0.10	0.478	755	0.02	-0.05	0.10	0.512	755	0.02	-0.06	0.09	0.645
Free cholesterol in large HDL (mmol/l)	755	0.02	-0.05	0.09	0.489	755	0.03	-0.05	0.10	0.509	755	0.02	-0.06	0.09	0.627
Triglycerides in large HDL (mmol/l)	755	-0.01	-0.08	0.05	0.692	755	-0.01	-0.08	0.06	0.740	755	-0.01	-0.08	0.06	0.800
Concentration of medium HDL particles (mol/l)	755	0.01	-0.06	0.07	0.830	755	0.01	-0.06	0.08	0.826	755	0.01	-0.06	0.08	0.791
Total lipids in medium HDL (mmol/l)	755	0.01	-0.06	0.07	0.802	755	0.01	-0.06	0.08	0.808	755	0.01	-0.06	0.08	0.801
Phospholipids in medium HDL (mmol/l)	755	0.01	-0.06	0.07	0.803	755	0.01	-0.06	0.08	0.828	755	0.01	-0.06	0.08	0.809
Total cholesterol in medium HDL (mmol/l)	755	0.01	-0.06	0.08	0.745	755	0.01	-0.06	0.08	0.737	755	0.01	-0.06	0.08	0.778
Cholesterol esters in medium HDL (mmol/l)	755	0.01	-0.05	0.08	0.720	755	0.01	-0.06	0.09	0.702	755	0.01	-0.06	0.08	0.755
Free cholesterol in medium HDL (mmol/l)	755	0.01	-0.06	0.07	0.817	755	0.01	-0.06	0.08	0.866	755	0.01	-0.06	0.08	0.851
Triglycerides in medium HDL (mmol/l)	755	-0.02	-0.08	0.05	0.583	755	-0.02	-0.08	0.05	0.616	755	-0.01	-0.07	0.06	0.826
Concentration of small HDL particles (mol/l)	755	-0.01	-0.07	0.05	0.775	755	-0.01	-0.08	0.05	0.712	755	-0.01	-0.07	0.06	0.816
Total lipids in small HDL (mmol/l)	755	0.01	-0.06	0.07	0.859	755	0.00	-0.06	0.07	0.895	755	0.01	-0.06	0.07	0.797
Phospholipids in small HDL (mmol/l)	755	-0.02	-0.09	0.04	0.519	755	-0.03	-0.09	0.04	0.449	755	-0.02	-0.09	0.04	0.483
Total cholesterol in small HDL (mmol/l)	755	0.03	-0.03	0.10	0.273	755	0.04	-0.03	0.10	0.276	755	0.04	-0.03	0.11	0.225
Cholesterol esters in small HDL (mmol/l)	755	0.04	-0.02	0.11	0.165	755	0.05	-0.02	0.11	0.174	755	0.05	-0.02	0.12	0.134
Free cholesterol in small HDL (mmol/l)	755	-0.01	-0.08	0.05	0.676	755	-0.01	-0.08	0.05	0.718	755	-0.01	-0.08	0.06	0.726
Triglycerides in small HDL (mmol/l)	755	-0.02	-0.08	0.04	0.512	755	-0.02	-0.08	0.04	0.522	755	-0.01	-0.07	0.05	0.711

S10 Table Associations of change in moderate-to-vigorous physical activity (MVPA change from age 12y-15y) with metabolic traits at age 15y in ALSPAC

Change in MVPA from age 12y-15y (per SD-unit increase)

	Adj. for age, sex, ethnicity, maternal education change in wear time, wear month					*Additionally adj. for change in SED*					*Additionally adj. for change in FMI*				
**Standardised outcome at age 15y**	**N**	**Beta**	**LCL**	**UCL**	**P-value**	**N**	**Beta**	**LCL**	**UCL**	**P-value**	**N**	**Beta**	**LCL**	**UCL**	**P-value**
Phospholipids to total lipds ratio in chylomicrons and extremely large VLDL (%)	755	-0.05	-0.11	0.02	0.169	755	-0.05	-0.11	0.02	0.168	755	-0.04	-0.11	0.02	0.210
Total cholesterol to total lipids ratio in chylomicrons and extremely large VLDL (%)	755	0.02	-0.05	0.08	0.613	755	0.02	-0.05	0.09	0.576	755	0.03	-0.04	0.10	0.389
Cholesterol esters to total lipids ratio in chylomicrons and extremely large VLDL (%)	755	0.03	-0.04	0.09	0.433	755	0.03	-0.04	0.10	0.378	755	0.04	-0.03	0.11	0.239
Free cholesterol to total lipids ratio in chylomicrons and extremely large VLDL (%)	755	-0.02	-0.08	0.05	0.663	755	-0.02	-0.09	0.05	0.593	755	-0.01	-0.08	0.06	0.747
Triglycerides to total lipids ratio in chylomicrons and extremely large VLDL (%)	755	-0.02	-0.07	0.03	0.466	755	-0.02	-0.07	0.03	0.453	755	-0.03	-0.08	0.03	0.310
Phospholipids to total lipds ratio in very large VLDL (%)	755	0.00	-0.07	0.07	0.991	755	0.00	-0.07	0.07	0.977	755	0.01	-0.06	0.09	0.719
Total cholesterol to total lipids ratio in very large VLDL (%)	755	0.08	-0.09	0.25	0.343	755	0.09	-0.08	0.25	0.309	755	0.08	-0.08	0.24	0.329
Cholesterol esters to total lipids ratio in very large VLDL (%)	755	0.01	-0.07	0.09	0.795	755	0.02	-0.06	0.10	0.682	755	0.01	-0.07	0.09	0.779
Free cholesterol to total lipids ratio in very large VLDL (%)	755	0.02	-0.06	0.09	0.665	755	0.02	-0.06	0.10	0.566	755	0.02	-0.06	0.10	0.588
Triglycerides to total lipids ratio in very large VLDL (%)	755	-0.02	-0.10	0.06	0.652	755	-0.03	-0.11	0.06	0.534	755	-0.03	-0.11	0.06	0.536
Phospholipids to total lipds ratio in large VLDL (%)	755	-0.01	-0.08	0.07	0.897	755	-0.01	-0.09	0.07	0.848	755	0.00	-0.08	0.08	0.952
Total cholesterol to total lipids ratio in large VLDL (%)	755	0.02	-0.06	0.10	0.702	755	0.03	-0.05	0.11	0.502	755	0.04	-0.04	0.12	0.377
Cholesterol esters to total lipids ratio in large VLDL (%)	755	0.14	-0.14	0.42	0.321	755	0.15	-0.13	0.42	0.292	755	0.14	-0.12	0.40	0.283
Free cholesterol to total lipids ratio in large VLDL (%)	755	-0.01	-0.08	0.06	0.760	755	-0.01	-0.08	0.06	0.776	755	0.00	-0.07	0.08	0.966
Triglycerides to total lipids ratio in large VLDL (%)	755	0.17	-0.15	0.48	0.305	755	0.16	-0.15	0.47	0.308	755	0.15	-0.14	0.44	0.313
Phospholipids to total lipds ratio in medium VLDL (%)	755	0.02	-0.05	0.09	0.583	755	0.04	-0.05	0.12	0.379	755	0.04	-0.05	0.12	0.401
Total cholesterol to total lipids ratio in medium VLDL (%)	755	0.00	-0.07	0.06	0.882	755	0.01	-0.05	0.08	0.734	755	0.02	-0.05	0.09	0.535
Cholesterol esters to total lipids ratio in medium VLDL (%)	755	0.00	-0.06	0.06	0.978	755	0.01	-0.05	0.08	0.651	755	0.02	-0.04	0.09	0.494
Free cholesterol to total lipids ratio in medium VLDL (%)	755	-0.01	-0.08	0.06	0.762	755	0.00	-0.07	0.07	0.976	755	0.01	-0.06	0.08	0.808
Triglycerides to total lipids ratio in medium VLDL (%)	755	0.00	-0.07	0.06	0.943	755	-0.02	-0.09	0.05	0.546	755	-0.03	-0.10	0.04	0.400
Phospholipids to total lipds ratio in small VLDL (%)	755	0.03	-0.03	0.09	0.277	755	0.03	-0.03	0.10	0.329	755	0.02	-0.04	0.09	0.505
Total cholesterol to total lipids ratio in small VLDL (%)	755	-0.01	-0.08	0.05	0.655	755	-0.01	-0.07	0.06	0.852	755	0.00	-0.07	0.07	0.937
Cholesterol esters to total lipids ratio in small VLDL (%)	755	-0.02	-0.08	0.05	0.637	755	-0.01	-0.08	0.06	0.756	755	-0.01	-0.07	0.06	0.839
Free cholesterol to total lipids ratio in small VLDL (%)	755	0.01	-0.05	0.07	0.731	755	0.03	-0.03	0.10	0.328	755	0.04	-0.04	0.11	0.325
Triglycerides to total lipids ratio in small VLDL (%)	755	0.01	-0.06	0.07	0.877	755	0.00	-0.07	0.06	0.937	755	0.00	-0.07	0.06	0.925
Phospholipids to total lipds ratio in very small VLDL (%)	755	0.01	-0.06	0.07	0.878	755	0.01	-0.06	0.08	0.807	755	0.01	-0.06	0.08	0.729
Total cholesterol to total lipids ratio in very small VLDL (%)	755	-0.02	-0.08	0.04	0.600	755	-0.02	-0.08	0.04	0.575	755	-0.02	-0.08	0.04	0.543
Cholesterol esters to total lipids ratio in very small VLDL (%)	755	-0.02	-0.08	0.05	0.575	755	-0.02	-0.09	0.04	0.459	755	-0.03	-0.09	0.04	0.440
Free cholesterol to total lipids ratio in very small VLDL (%)	755	-0.01	-0.05	0.04	0.808	755	0.00	-0.04	0.05	0.856	755	0.00	-0.05	0.05	0.898
Triglycerides to total lipids ratio in very small VLDL (%)	755	0.02	-0.04	0.07	0.572	755	0.02	-0.04	0.07	0.595	755	0.01	-0.04	0.07	0.614
Phospholipids to total lipds ratio in IDL (%)	755	0.04	-0.01	0.09	0.142	755	0.02	-0.04	0.08	0.567	755	0.01	-0.06	0.07	0.851
Total cholesterol to total lipids ratio in IDL (%)	755	-0.04	-0.10	0.02	0.177	755	-0.03	-0.09	0.03	0.349	755	-0.02	-0.08	0.04	0.513
Cholesterol esters to total lipids ratio in IDL (%)	755	-0.05	-0.11	0.02	0.147	755	-0.03	-0.10	0.03	0.320	755	-0.02	-0.09	0.04	0.513
Free cholesterol to total lipids ratio in IDL (%)	755	0.01	-0.05	0.07	0.797	755	0.01	-0.06	0.07	0.870	755	0.00	-0.06	0.06	0.998
Triglycerides to total lipids ratio in IDL (%)	755	0.03	-0.03	0.09	0.294	755	0.03	-0.03	0.09	0.355	755	0.02	-0.04	0.09	0.453
Phospholipids to total lipds ratio in large LDL (%)	755	0.03	-0.04	0.09	0.399	755	0.02	-0.04	0.09	0.525	755	0.01	-0.05	0.08	0.655
Total cholesterol to total lipids ratio in large LDL (%)	755	-0.03	-0.10	0.03	0.266	755	-0.03	-0.09	0.04	0.410	755	-0.02	-0.08	0.05	0.570
Cholesterol esters to total lipids ratio in large LDL (%)	755	-0.04	-0.10	0.03	0.257	755	-0.03	-0.10	0.04	0.385	755	-0.02	-0.09	0.05	0.581
Free cholesterol to total lipids ratio in large LDL (%)	755	0.02	-0.04	0.08	0.492	755	0.02	-0.04	0.08	0.543	755	0.01	-0.05	0.07	0.793
Triglycerides to total lipids ratio in large LDL (%)	755	0.03	-0.03	0.09	0.337	755	0.02	-0.04	0.09	0.473	755	0.02	-0.05	0.08	0.619
Phospholipids to total lipds ratio in medium LDL (%)	755	0.01	-0.01	0.03	0.389	755	0.01	-0.01	0.04	0.428	755	0.01	-0.02	0.03	0.526
Total cholesterol to total lipids ratio in medium LDL (%)	755	-0.05	-0.11	0.02	0.177	755	-0.04	-0.11	0.03	0.247	755	-0.03	-0.10	0.04	0.343
Cholesterol esters to total lipids ratio in medium LDL (%)	755	-0.04	-0.11	0.03	0.266	755	-0.04	-0.11	0.03	0.311	755	-0.03	-0.10	0.04	0.431
Free cholesterol to total lipids ratio in medium LDL (%)	755	0.01	-0.01	0.03	0.522	755	0.01	-0.01	0.03	0.497	755	0.00	-0.02	0.02	0.647
Triglycerides to total lipids ratio in medium LDL (%)	755	0.04	-0.02	0.11	0.172	755	0.03	-0.03	0.10	0.293	755	0.03	-0.04	0.09	0.377
Phospholipids to total lipds ratio in small LDL (%)	755	0.02	-0.02	0.06	0.415	755	0.01	-0.03	0.05	0.479	755	0.01	-0.03	0.05	0.614
Total cholesterol to total lipids ratio in small LDL (%)	755	-0.03	-0.10	0.03	0.318	755	-0.03	-0.10	0.04	0.401	755	-0.02	-0.09	0.04	0.510
Cholesterol esters to total lipids ratio in small LDL (%)	755	-0.03	-0.09	0.04	0.441	755	-0.03	-0.10	0.05	0.484	755	-0.02	-0.09	0.05	0.617
Free cholesterol to total lipids ratio in small LDL (%)	755	0.01	-0.03	0.04	0.764	755	0.01	-0.03	0.04	0.701	755	0.00	-0.03	0.04	0.852

S10 Table Associations of change in moderate-to-vigorous physical activity (MVPA change from age 12y-15y) with metabolic traits at age 15y in ALSPAC

Change in MVPA from age 12y-15y (per SD-unit increase)

	Adj. for age, sex, ethnicity, maternal education change in wear time, wear month					*Additionally adj. for change in SED*					*Additionally adj. for change in FMI*				
**Standardised outcome at age 15y**	**N**	**Beta**	**LCL**	**UCL**	**P-value**	**N**	**Beta**	**LCL**	**UCL**	**P-value**	**N**	**Beta**	**LCL**	**UCL**	**P-value**
Triglycerides to total lipids ratio in small LDL (%)	755	0.03	-0.03	0.09	0.367	755	0.02	-0.04	0.08	0.478	755	0.02	-0.04	0.08	0.466
Phospholipids to total lipds ratio in very large HDL (%)	755	0.04	-0.02	0.11	0.197	755	0.04	-0.03	0.11	0.272	755	0.03	-0.04	0.10	0.421
Total cholesterol to total lipids ratio in very large HDL (%)	755	-0.04	-0.11	0.03	0.227	755	-0.04	-0.11	0.03	0.294	755	-0.03	-0.10	0.04	0.429
Cholesterol esters to total lipids ratio in very large HDL (%)	755	-0.04	-0.11	0.03	0.256	755	-0.04	-0.11	0.04	0.323	755	-0.03	-0.10	0.04	0.463
Free cholesterol to total lipids ratio in very large HDL (%)	755	-0.01	-0.06	0.05	0.858	755	0.00	-0.07	0.06	0.885	755	-0.01	-0.07	0.06	0.838
Triglycerides to total lipids ratio in very large HDL (%)	755	-0.03	-0.08	0.03	0.380	755	-0.02	-0.08	0.04	0.517	755	-0.01	-0.08	0.05	0.710
Phospholipids to total lipds ratio in large HDL (%)	755	0.00	-0.06	0.05	0.891	755	-0.01	-0.06	0.05	0.857	755	0.01	-0.05	0.06	0.842
Total cholesterol to total lipids ratio in large HDL (%)	755	0.01	-0.04	0.07	0.628	755	0.01	-0.05	0.08	0.647	755	0.00	-0.06	0.06	0.947
Cholesterol esters to total lipids ratio in large HDL (%)	755	0.01	-0.05	0.07	0.662	755	0.01	-0.05	0.07	0.710	755	0.00	-0.06	0.06	0.969
Free cholesterol to total lipids ratio in large HDL (%)	755	0.01	-0.05	0.08	0.632	755	0.02	-0.04	0.08	0.549	755	0.01	-0.05	0.07	0.713
Triglycerides to total lipids ratio in large HDL (%)	755	-0.03	-0.09	0.03	0.375	755	-0.02	-0.09	0.04	0.466	755	-0.01	-0.08	0.05	0.688
Phospholipids to total lipds ratio in medium HDL (%)	755	0.01	-0.05	0.07	0.815	755	0.00	-0.06	0.06	0.997	755	0.00	-0.06	0.07	0.931
Total cholesterol to total lipids ratio in medium HDL (%)	755	0.00	-0.06	0.06	0.983	755	0.01	-0.06	0.07	0.833	755	0.00	-0.06	0.06	0.969
Cholesterol esters to total lipids ratio in medium HDL (%)	755	0.00	-0.06	0.06	0.936	755	0.01	-0.05	0.08	0.721	755	0.00	-0.06	0.07	0.927
Free cholesterol to total lipids ratio in medium HDL (%)	755	0.00	-0.07	0.07	0.985	755	-0.01	-0.09	0.06	0.734	755	-0.01	-0.09	0.07	0.739
Triglycerides to total lipids ratio in medium HDL (%)	755	-0.02	-0.08	0.05	0.648	755	-0.02	-0.09	0.05	0.649	755	-0.01	-0.08	0.06	0.869
Phospholipids to total lipds ratio in small HDL (%)	755	-0.05	-0.12	0.01	0.101	755	-0.06	-0.12	0.01	0.093	755	-0.06	-0.13	0.01	0.071
Total cholesterol to total lipids ratio in small HDL (%)	755	0.05	-0.01	0.12	0.090	755	0.06	-0.01	0.12	0.086	755	0.06	-0.01	0.13	0.074
Cholesterol esters to total lipids ratio in small HDL (%)	755	0.06	0.00	0.12	0.069	755	0.06	-0.01	0.13	0.071	755	0.06	0.00	0.13	0.057
Free cholesterol to total lipids ratio in small HDL (%)	755	-0.05	-0.11	0.01	0.124	755	-0.04	-0.11	0.02	0.218	755	-0.05	-0.12	0.02	0.132
Triglycerides to total lipids ratio in small HDL (%)	755	-0.02	-0.08	0.04	0.492	755	-0.02	-0.09	0.04	0.494	755	-0.01	-0.08	0.05	0.658
Mean diameter for VLDL particles (nm)	755	-0.03	-0.10	0.04	0.381	755	-0.03	-0.10	0.04	0.390	755	-0.02	-0.09	0.05	0.554
Mean diameter for LDL particles (nm)	755	0.02	-0.03	0.08	0.425	755	0.02	-0.04	0.08	0.479	755	0.01	-0.05	0.07	0.652
Mean diameter for HDL particles (nm)	755	0.02	-0.05	0.09	0.633	755	0.02	-0.06	0.09	0.637	755	0.01	-0.06	0.09	0.755
Serum total cholesterol (mmol/l)	755	-0.01	-0.07	0.06	0.789	755	0.00	-0.07	0.07	0.996	755	0.01	-0.06	0.08	0.774
Total cholesterol in VLDL (mmol/l)	755	-0.04	-0.10	0.03	0.253	755	-0.03	-0.09	0.04	0.441	755	-0.01	-0.08	0.06	0.774
Remnant cholesterol (non-HDL, non-LDL -cholesterol) (mmol/l)	755	-0.03	-0.09	0.03	0.372	755	-0.02	-0.09	0.05	0.614	755	0.00	-0.07	0.07	0.958
Total cholesterol in LDL (mmol/l)	755	-0.01	-0.07	0.06	0.802	755	0.00	-0.07	0.07	0.990	755	0.01	-0.06	0.08	0.772
Total cholesterol in HDL (mmol/l)	755	0.02	-0.05	0.09	0.591	755	0.02	-0.06	0.09	0.610	755	0.02	-0.06	0.09	0.671
Total cholesterol in HDL2 (mmol/l)	755	0.02	-0.05	0.09	0.506	755	0.02	-0.05	0.10	0.560	755	0.02	-0.06	0.09	0.652
Total cholesterol in HDL3 (mmol/l)	755	0.01	-0.06	0.08	0.773	755	0.01	-0.06	0.09	0.717	755	0.01	-0.06	0.09	0.717
Esterified cholesterol (mmol/l)	755	-0.01	-0.07	0.06	0.793	755	0.00	-0.07	0.07	0.984	755	0.01	-0.06	0.08	0.781
Free cholesterol (mmol/l)	755	-0.01	-0.08	0.06	0.791	755	0.00	-0.07	0.07	0.979	755	0.01	-0.06	0.08	0.770
Serum total triglycerides (mmol/l)	755	-0.03	-0.09	0.03	0.267	755	-0.03	-0.09	0.03	0.366	755	-0.02	-0.08	0.05	0.600
Triglycerides in VLDL (mmol/l)	755	-0.04	-0.10	0.02	0.215	755	-0.03	-0.10	0.03	0.303	755	-0.02	-0.08	0.04	0.518
Triglycerides in LDL (mmol/l)	755	0.00	-0.06	0.06	0.992	755	0.00	-0.06	0.07	0.941	755	0.01	-0.06	0.07	0.852
Triglycerides in HDL (mmol/l)	755	-0.02	-0.08	0.04	0.476	755	-0.02	-0.08	0.04	0.526	755	-0.01	-0.08	0.05	0.717
Diacylglycerol (mmol/l)	755	0.02	-0.05	0.08	0.647	755	0.01	-0.06	0.08	0.749	755	0.02	-0.05	0.09	0.565
Ratio of diacylglycerol to triglycerides	755	0.04	-0.03	0.12	0.278	755	0.03	-0.05	0.11	0.416	755	0.04	-0.04	0.12	0.367
Total phosphoglycerides (mmol/l)	755	0.01	-0.06	0.07	0.873	755	0.01	-0.06	0.08	0.780	755	0.02	-0.06	0.09	0.653
Ratio of triglycerides to phosphoglycerides	755	-0.02	-0.08	0.03	0.399	755	-0.02	-0.08	0.04	0.584	755	-0.01	-0.07	0.05	0.839
Phosphatidylcholine and other cholines (mmol/l)	755	-0.02	-0.09	0.05	0.569	755	-0.01	-0.08	0.06	0.737	755	-0.01	-0.08	0.06	0.844
Total cholines (mmol/l)	755	0.01	-0.06	0.08	0.773	755	0.01	-0.06	0.09	0.712	755	0.02	-0.05	0.09	0.615
Apolipoprotein A-I (g/l)	755	0.01	-0.06	0.08	0.814	755	0.01	-0.06	0.09	0.760	755	0.01	-0.06	0.09	0.728
Apolipoprotein B (g/l)	755	-0.03	-0.09	0.03	0.371	755	-0.02	-0.08	0.05	0.604	755	0.00	-0.07	0.06	0.935
Ratio of apolipoprotein B to apolipoprotein A-I	755	-0.03	-0.10	0.04	0.369	755	-0.02	-0.09	0.05	0.573	755	-0.01	-0.08	0.06	0.878
Total fatty acids (mmol/l)	755	-0.01	-0.07	0.05	0.742	755	0.00	-0.07	0.07	0.947	755	0.01	-0.06	0.08	0.797
Estimated description of fatty acid chain length, not actual carbon number	755	0.00	-0.06	0.05	0.882	755	-0.01	-0.07	0.05	0.723	755	-0.01	-0.07	0.05	0.742
Estimated degree of unsaturation	755	0.01	-0.06	0.07	0.856	755	0.01	-0.06	0.07	0.794	755	0.00	-0.06	0.07	0.892

S10 Table Associations of change in moderate-to-vigorous physical activity (MVPA change from age 12y-15y) with metabolic traits at age 15y in ALSPAC

Change in MVPA from age 12y-15y (per SD-unit increase)

	Adj. for age, sex, ethnicity, maternal education change in wear time, wear month					*Additionally adj. for change in SED*					*Additionally adj. for change in FMI*				
**Standardised outcome at age 15y**	**N**	**Beta**	**LCL**	**UCL**	**P-value**	**N**	**Beta**	**LCL**	**UCL**	**P-value**	**N**	**Beta**	**LCL**	**UCL**	**P-value**
22:6, docosahexaenoic acid (mmol/l)	755	0.02	-0.05	0.08	0.562	755	0.01	-0.06	0.07	0.871	755	0.01	-0.05	0.08	0.700
18:2, linoleic acid (mmol/l)	755	0.01	-0.06	0.07	0.862	755	0.02	-0.05	0.09	0.565	755	0.03	-0.04	0.10	0.397
Conjugated linoleic acid (mmol/l)	755	-0.01	-0.07	0.05	0.707	755	-0.01	-0.07	0.05	0.664	755	-0.01	-0.07	0.05	0.769
Omega-3 fatty acids (mmol/l)	755	0.00	-0.07	0.07	0.970	755	-0.01	-0.08	0.06	0.816	755	0.00	-0.07	0.07	0.972
Omega-6 fatty acids (mmol/l)	755	0.00	-0.06	0.07	0.902	755	0.02	-0.05	0.09	0.663	755	0.03	-0.05	0.10	0.486
Polyunsaturated fatty acids (mmol/l)	755	0.00	-0.06	0.07	0.916	755	0.01	-0.06	0.08	0.715	755	0.02	-0.05	0.09	0.520
Monounsaturated fatty acids; 16:1, 18:1 (mmol/l)	755	-0.03	-0.09	0.03	0.373	755	-0.02	-0.08	0.05	0.602	755	-0.01	-0.07	0.06	0.835
Saturated fatty acids (mmol/l)	755	-0.01	-0.07	0.06	0.864	755	0.00	-0.07	0.07	0.941	755	0.01	-0.06	0.08	0.826
Ratio of 22:6 docosahexaenoic acid to total fatty acids (%)	755	0.02	-0.05	0.09	0.560	755	0.00	-0.07	0.07	0.963	755	0.00	-0.07	0.07	0.960
Ratio of 18:2 linoleic acid to total fatty acids (%)	755	0.02	-0.05	0.09	0.505	755	0.03	-0.04	0.11	0.341	755	0.03	-0.04	0.10	0.370
Ratio of conjugated linoleic acid to total fatty acids (%)	755	0.00	-0.06	0.06	0.982	755	0.00	-0.07	0.06	0.925	755	0.00	-0.06	0.06	0.986
Ratio of omega-3 fatty acids to total fatty acids (%)	755	-0.01	-0.08	0.06	0.855	755	-0.02	-0.09	0.06	0.625	755	-0.01	-0.09	0.06	0.701
Ratio of omega-6 fatty acids to total fatty acids (%)	755	0.02	-0.05	0.09	0.524	755	0.03	-0.04	0.10	0.410	755	0.03	-0.05	0.10	0.482
Ratio of polyunsaturated fatty acids to total fatty acids (%)	755	0.02	-0.05	0.09	0.558	755	0.02	-0.04	0.09	0.482	755	0.02	-0.05	0.09	0.544
Ratio of monounsaturated fatty acids to total fatty acids (%)	755	-0.03	-0.10	0.03	0.347	755	-0.03	-0.10	0.04	0.442	755	-0.02	-0.09	0.05	0.508
Ratio of saturated fatty acids to total fatty acids (%)	755	0.02	-0.05	0.08	0.591	755	0.01	-0.06	0.07	0.870	755	0.00	-0.06	0.07	0.885
Insulin (mu/l)	755	0.02	-0.02	0.07	0.301	755	0.02	-0.02	0.07	0.285	755	0.03	-0.01	0.08	0.139
Glucose (mmol/l)	755	-0.01	-0.07	0.06	0.815	755	0.00	-0.07	0.07	0.905	755	0.01	-0.06	0.08	0.810
Lactate (mmol/l)	755	0.00	-0.07	0.07	0.963	755	0.01	-0.06	0.08	0.804	755	0.01	-0.06	0.08	0.755
Pyruvate (mmol/l)	755	0.02	-0.05	0.09	0.614	755	0.03	-0.04	0.11	0.371	755	0.04	-0.04	0.11	0.342
Citrate (mmol/l)	755	0.00	-0.08	0.08	0.950	755	0.00	-0.08	0.09	0.929	755	0.00	-0.08	0.08	0.987
Alanine (mmol/l)	755	0.05	-0.02	0.12	0.127	755	0.08	0.00	0.15	0.040	755	0.08	0.01	0.15	0.032
Glutamine (mmol/l)	755	0.01	-0.04	0.07	0.639	755	0.00	-0.06	0.06	0.988	755	0.00	-0.06	0.06	0.912
Histidine (mmol/l)	755	0.01	-0.06	0.08	0.792	755	0.01	-0.06	0.08	0.720	755	0.02	-0.05	0.09	0.624
Isoleucine (mmol/l)	755	-0.01	-0.06	0.05	0.857	755	0.00	-0.06	0.06	0.959	755	0.01	-0.05	0.07	0.852
Leucine (mmol/l)	755	0.04	-0.02	0.09	0.197	755	0.04	-0.02	0.09	0.201	755	0.04	-0.02	0.09	0.160
Valine (mmol/l)	755	0.02	-0.05	0.08	0.569	755	0.02	-0.04	0.09	0.466	755	0.03	-0.04	0.09	0.385
Phenylalanine (mmol/l)	755	0.02	-0.04	0.09	0.485	755	0.01	-0.07	0.08	0.869	755	0.01	-0.07	0.08	0.835
Tyrosine (mmol/l)	755	0.00	-0.07	0.07	0.982	755	-0.02	-0.09	0.05	0.529	755	-0.01	-0.09	0.06	0.691
Acetate (mmol/l)	755	0.02	-0.04	0.09	0.505	755	0.03	-0.04	0.10	0.358	755	0.03	-0.04	0.10	0.373
Acetoacetate (mmol/l)	755	0.03	-0.03	0.09	0.316	755	0.03	-0.03	0.10	0.309	755	0.03	-0.04	0.09	0.428
3-hydroxybutyrate (mmol/l)	755	0.03	-0.04	0.09	0.402	755	0.02	-0.04	0.09	0.473	755	0.02	-0.05	0.09	0.542
Creatinine (mmol/l)	755	0.01	-0.06	0.07	0.845	755	0.02	-0.05	0.09	0.610	755	0.01	-0.06	0.08	0.755
Albumin (signal area)	755	-0.01	-0.06	0.05	0.852	755	0.00	-0.06	0.06	0.941	755	-0.01	-0.07	0.06	0.821
Glycoprotein acetyls, mainly a1-acid glycoprotein (mmol/l)	755	0.00	-0.07	0.06	0.919	755	-0.02	-0.08	0.05	0.632	755	-0.01	-0.07	0.06	0.830
C-reactive protein (mg/l)	755	0.01	-0.03	0.04	0.701	755	0.00	-0.03	0.04	0.819	755	0.01	-0.03	0.04	0.753
