# Supplementary material for: Associations of device-measured physical activity across adolescence with metabolic traits: Prospective cohort study
Source: PLoS Med. 2018 Sep 11;15(9):e1002649. doi: 10.1371/journal.pmed.1002649 (PMC6133272; doi:10.1371/journal.pmed.1002649)
Supplement: S11 Table — ALSPAC, Avon Longitudinal Study of Parents and Children; SED, sedentary time. (PDF) [file pmed.1002649.s011.pdf]

S11 Table Associations of change in sedentary time (SED change from age 12y-15y) with metabolic traits at age 15y in ALSPAC

Change in SED from age 12y-15y (per SD-unit increase)

	Adj. for age, sex, ethnicity, maternal education change in wear time, wear month					*Additionally adj. for change in MVPA*					*Additionally adj. for change in FMI*				
**Standardised outcome at age 15y**	**N**	**Beta**	**LCL**	**UCL**	**P-value**	**N**	**Beta**	**LCL**	**UCL**	**P-value**	**N**	**Beta**	**LCL**	**UCL**	**P-value**
Systolic blood pressure (mmHg)	1599	0.00	-0.05	0.04	0.885	1599	0.01	-0.04	0.06	0.790	1553	0.01	-0.04	0.06	0.611
Diastolic blood pressure (mmHg)	1599	0.03	-0.02	0.07	0.219	1599	0.05	0.00	0.10	0.036	1553	0.05	0.00	0.10	0.046
Concentration of chylomicrons and extremely large VLDL particles (mol/l)	1076	0.01	-0.04	0.07	0.632	1076	0.00	-0.06	0.06	0.954	1054	-0.02	-0.08	0.04	0.510
Total lipids in chylomicrons and extremely large VLDL (mmol/l)	1076	0.01	-0.05	0.07	0.682	1076	0.00	-0.06	0.06	0.894	1054	-0.02	-0.08	0.04	0.465
Phospholipids in chylomicrons and extremely large VLDL (mmol/l)	1076	0.01	-0.05	0.07	0.677	1076	0.00	-0.06	0.06	0.889	1054	-0.02	-0.08	0.04	0.448
Total cholesterol in chylomicrons and extremely large VLDL (mmol/l)	1076	0.01	-0.05	0.07	0.733	1076	-0.01	-0.07	0.06	0.847	1054	-0.02	-0.08	0.04	0.476
Cholesterol esters in chylomicrons and extremely large VLDL (mmol/l)	1076	0.01	-0.05	0.07	0.781	1076	-0.01	-0.07	0.06	0.832	1054	-0.02	-0.08	0.04	0.525
Free cholesterol in chylomicrons and extremely large VLDL (mmol/l)	1076	0.01	-0.05	0.07	0.698	1076	0.00	-0.07	0.06	0.873	1054	-0.02	-0.08	0.04	0.452
Triglycerides in chylomicrons and extremely large VLDL (mmol/l)	1076	0.01	-0.04	0.07	0.672	1076	0.00	-0.06	0.06	0.907	1054	-0.02	-0.08	0.04	0.468
Concentration of very large VLDL particles (mol/l)	1076	0.02	-0.04	0.08	0.480	1076	0.01	-0.06	0.07	0.861	1054	-0.01	-0.07	0.05	0.669
Total lipids in very large VLDL (mmol/l)	1076	0.02	-0.04	0.08	0.515	1076	0.00	-0.06	0.07	0.908	1054	-0.02	-0.08	0.05	0.627
Phospholipids in very large VLDL (mmol/l)	1076	0.02	-0.04	0.07	0.597	1076	0.00	-0.06	0.06	0.972	1054	-0.02	-0.08	0.04	0.524
Total cholesterol in very large VLDL (mmol/l)	1076	0.01	-0.04	0.07	0.620	1076	0.00	-0.06	0.06	0.957	1054	-0.02	-0.08	0.04	0.545
Cholesterol esters in very large VLDL (mmol/l)	1076	0.02	-0.04	0.08	0.577	1076	0.00	-0.06	0.06	0.978	1054	-0.02	-0.08	0.05	0.624
Free cholesterol in very large VLDL (mmol/l)	1076	0.01	-0.05	0.07	0.673	1076	0.00	-0.07	0.06	0.885	1054	-0.02	-0.08	0.04	0.468
Triglycerides in very large VLDL (mmol/l)	1076	0.02	-0.04	0.08	0.467	1076	0.01	-0.06	0.07	0.837	1054	-0.01	-0.07	0.05	0.685
Concentration of large VLDL particles (mol/l)	1076	0.02	-0.04	0.08	0.481	1076	0.01	-0.06	0.07	0.850	1054	-0.01	-0.07	0.05	0.698
Total lipids in large VLDL (mmol/l)	1076	0.02	-0.04	0.08	0.495	1076	0.01	-0.06	0.07	0.875	1054	-0.01	-0.08	0.05	0.685
Phospholipids in large VLDL (mmol/l)	1076	0.02	-0.04	0.08	0.506	1076	0.00	-0.06	0.07	0.901	1054	-0.01	-0.08	0.05	0.658
Total cholesterol in large VLDL (mmol/l)	1076	0.02	-0.04	0.08	0.464	1076	0.01	-0.06	0.07	0.859	1054	-0.01	-0.07	0.05	0.742
Cholesterol esters in large VLDL (mmol/l)	1076	0.02	-0.04	0.08	0.464	1076	0.01	-0.06	0.07	0.848	1054	-0.01	-0.07	0.06	0.814
Free cholesterol in large VLDL (mmol/l)	1076	0.02	-0.04	0.08	0.472	1076	0.01	-0.06	0.07	0.873	1054	-0.01	-0.08	0.05	0.676
Triglycerides in large VLDL (mmol/l)	1076	0.02	-0.04	0.08	0.506	1076	0.01	-0.06	0.07	0.873	1054	-0.01	-0.08	0.05	0.673
Concentration of medium VLDL particles (mol/l)	1076	0.03	-0.04	0.09	0.419	1076	0.01	-0.06	0.07	0.794	1054	-0.01	-0.07	0.06	0.791
Total lipids in medium VLDL (mmol/l)	1076	0.02	-0.04	0.09	0.429	1076	0.01	-0.06	0.07	0.812	1054	-0.01	-0.07	0.06	0.796
Phospholipids in medium VLDL (mmol/l)	1076	0.03	-0.03	0.09	0.381	1076	0.01	-0.06	0.07	0.767	1054	-0.01	-0.07	0.06	0.839
Total cholesterol in medium VLDL (mmol/l)	1076	0.03	-0.04	0.09	0.398	1076	0.01	-0.06	0.07	0.798	1054	0.00	-0.07	0.06	0.920
Cholesterol esters in medium VLDL (mmol/l)	1076	0.03	-0.04	0.09	0.437	1076	0.01	-0.06	0.07	0.835	1054	0.00	-0.07	0.07	0.992
Free cholesterol in medium VLDL (mmol/l)	1076	0.03	-0.03	0.09	0.379	1076	0.01	-0.05	0.07	0.765	1054	-0.01	-0.07	0.06	0.835
Triglycerides in medium VLDL (mmol/l)	1076	0.02	-0.04	0.08	0.477	1076	0.01	-0.06	0.07	0.840	1054	-0.01	-0.07	0.05	0.727
Concentration of small VLDL particles (mol/l)	1076	0.03	-0.03	0.09	0.295	1076	0.01	-0.05	0.08	0.655	1054	0.00	-0.07	0.06	0.991
Total lipids in small VLDL (mmol/l)	1076	0.03	-0.03	0.09	0.320	1076	0.01	-0.05	0.08	0.710	1054	0.00	-0.07	0.06	0.964
Phospholipids in small VLDL (mmol/l)	1076	0.03	-0.03	0.09	0.313	1076	0.01	-0.05	0.08	0.688	1054	0.00	-0.06	0.07	0.993
Total cholesterol in small VLDL (mmol/l)	1076	0.03	-0.03	0.09	0.379	1076	0.01	-0.06	0.07	0.816	1054	0.00	-0.07	0.06	0.955
Cholesterol esters in small VLDL (mmol/l)	1076	0.02	-0.04	0.08	0.495	1076	0.00	-0.06	0.07	0.955	1054	-0.01	-0.07	0.06	0.863
Free cholesterol in small VLDL (mmol/l)	1076	0.04	-0.02	0.10	0.238	1076	0.02	-0.05	0.08	0.578	1054	0.01	-0.06	0.07	0.858
Triglycerides in small VLDL (mmol/l)	1076	0.03	-0.03	0.09	0.338	1076	0.01	-0.05	0.08	0.666	1054	0.00	-0.07	0.06	0.958
Concentration of very small VLDL particles (mol/l)	1076	0.03	-0.03	0.09	0.365	1076	0.01	-0.05	0.08	0.720	1054	0.01	-0.06	0.08	0.802
Total lipids in very small VLDL (mmol/l)	1076	0.02	-0.04	0.09	0.443	1076	0.01	-0.06	0.07	0.866	1054	0.00	-0.07	0.07	0.987
Phospholipids in very small VLDL (mmol/l)	1076	0.01	-0.05	0.08	0.630	1076	0.00	-0.06	0.06	0.955	1054	0.00	-0.07	0.06	0.887
Total cholesterol in very small VLDL (mmol/l)	1076	0.02	-0.04	0.08	0.538	1076	0.00	-0.06	0.07	0.937	1054	0.00	-0.07	0.07	0.997
Cholesterol esters in very small VLDL (mmol/l)	1076	0.01	-0.05	0.08	0.651	1076	0.00	-0.07	0.06	0.948	1054	-0.01	-0.07	0.06	0.860
Free cholesterol in very small VLDL (mmol/l)	1076	0.03	-0.03	0.09	0.358	1076	0.01	-0.05	0.08	0.698	1054	0.01	-0.05	0.08	0.711
Triglycerides in very small VLDL (mmol/l)	1076	0.03	-0.03	0.10	0.279	1076	0.02	-0.05	0.08	0.553	1054	0.01	-0.06	0.08	0.785
Concentration of IDL particles (mol/l)	1076	0.01	-0.05	0.07	0.657	1076	0.00	-0.06	0.06	0.961	1054	-0.01	-0.07	0.06	0.873
Total lipids in IDL (mmol/l)	1076	0.01	-0.05	0.07	0.712	1076	0.00	-0.07	0.06	0.902	1054	-0.01	-0.07	0.06	0.846
Phospholipids in IDL (mmol/l)	1076	0.00	-0.06	0.06	0.918	1076	-0.01	-0.07	0.05	0.732	1054	-0.01	-0.08	0.05	0.689
Total cholesterol in IDL (mmol/l)	1076	0.01	-0.05	0.07	0.690	1076	0.00	-0.07	0.06	0.918	1054	-0.01	-0.07	0.06	0.866

S11 Table Associations of change in sedentary time (SED change from age 12y-15y) with metabolic traits at age 15y in ALSPAC

Change in SED from age 12y-15y (per SD-unit increase)

	Adj. for age, sex, ethnicity, maternal education change in wear time, wear month					*Additionally adj. for change in MVPA*					*Additionally adj. for change in FMI*				
**Standardised outcome at age 15y**	**N**	**Beta**	**LCL**	**UCL**	**P-value**	**N**	**Beta**	**LCL**	**UCL**	**P-value**	**N**	**Beta**	**LCL**	**UCL**	**P-value**
Cholesterol esters in IDL (mmol/l)	1076	0.01	-0.05	0.07	0.632	1076	0.00	-0.06	0.06	0.960	1054	0.00	-0.07	0.06	0.893
Free cholesterol in IDL (mmol/l)	1076	0.01	-0.05	0.06	0.853	1076	-0.01	-0.07	0.05	0.820	1054	-0.01	-0.07	0.06	0.808
Triglycerides in IDL (mmol/l)	1076	0.02	-0.04	0.08	0.546	1076	0.01	-0.06	0.08	0.776	1054	0.01	-0.06	0.07	0.855
Concentration of large LDL particles (mol/l)	1076	0.00	-0.05	0.06	0.875	1076	-0.01	-0.07	0.05	0.755	1054	-0.01	-0.08	0.05	0.659
Total lipids in large LDL (mmol/l)	1076	0.00	-0.06	0.06	0.905	1076	-0.01	-0.07	0.05	0.729	1054	-0.01	-0.08	0.05	0.659
Phospholipids in large LDL (mmol/l)	1076	0.00	-0.06	0.06	0.980	1076	-0.01	-0.08	0.05	0.651	1054	-0.02	-0.08	0.05	0.588
Total cholesterol in large LDL (mmol/l)	1076	0.00	-0.05	0.06	0.899	1076	-0.01	-0.07	0.05	0.737	1054	-0.01	-0.08	0.05	0.670
Cholesterol esters in large LDL (mmol/l)	1076	0.00	-0.05	0.06	0.887	1076	-0.01	-0.07	0.05	0.735	1054	-0.01	-0.08	0.05	0.653
Free cholesterol in large LDL (mmol/l)	1076	0.00	-0.06	0.06	0.937	1076	-0.01	-0.07	0.05	0.743	1054	-0.01	-0.07	0.05	0.723
Triglycerides in large LDL (mmol/l)	1076	0.01	-0.06	0.07	0.835	1076	0.00	-0.07	0.06	0.921	1054	-0.01	-0.08	0.06	0.833
Concentration of medium LDL particles (mol/l)	1076	0.00	-0.05	0.06	0.878	1076	-0.01	-0.07	0.05	0.743	1054	-0.02	-0.08	0.05	0.606
Total lipids in medium LDL (mmol/l)	1076	0.00	-0.05	0.06	0.893	1076	-0.01	-0.07	0.05	0.726	1054	-0.02	-0.08	0.05	0.614
Phospholipids in medium LDL (mmol/l)	1076	0.01	-0.05	0.06	0.842	1076	-0.01	-0.07	0.05	0.744	1054	-0.02	-0.08	0.05	0.633
Total cholesterol in medium LDL (mmol/l)	1076	0.00	-0.05	0.06	0.882	1076	-0.01	-0.07	0.05	0.736	1054	-0.02	-0.08	0.05	0.624
Cholesterol esters in medium LDL (mmol/l)	1076	0.00	-0.06	0.06	0.935	1076	-0.01	-0.07	0.05	0.687	1054	-0.02	-0.08	0.05	0.571
Free cholesterol in medium LDL (mmol/l)	1076	0.01	-0.05	0.07	0.668	1076	0.00	-0.06	0.06	0.972	1054	0.00	-0.07	0.06	0.895
Triglycerides in medium LDL (mmol/l)	1076	0.00	-0.07	0.06	0.898	1076	-0.01	-0.08	0.06	0.728	1054	-0.02	-0.08	0.05	0.640
Concentration of small LDL particles (mol/l)	1076	0.01	-0.05	0.07	0.795	1076	-0.01	-0.07	0.05	0.797	1054	-0.02	-0.08	0.05	0.602
Total lipids in small LDL (mmol/l)	1076	0.00	-0.05	0.06	0.884	1076	-0.01	-0.07	0.05	0.722	1054	-0.02	-0.08	0.04	0.577
Phospholipids in small LDL (mmol/l)	1076	0.01	-0.05	0.06	0.804	1076	-0.01	-0.07	0.05	0.752	1054	-0.02	-0.08	0.04	0.562
Total cholesterol in small LDL (mmol/l)	1076	0.00	-0.06	0.06	0.921	1076	-0.01	-0.07	0.05	0.706	1054	-0.02	-0.08	0.05	0.588
Cholesterol esters in small LDL (mmol/l)	1076	0.00	-0.06	0.06	0.995	1076	-0.01	-0.08	0.05	0.654	1054	-0.02	-0.08	0.04	0.540
Free cholesterol in small LDL (mmol/l)	1076	0.01	-0.04	0.07	0.614	1076	0.00	-0.06	0.06	0.990	1054	0.00	-0.07	0.06	0.875
Triglycerides in small LDL (mmol/l)	1076	0.01	-0.06	0.07	0.835	1076	-0.01	-0.07	0.06	0.874	1054	-0.02	-0.08	0.05	0.651
Concentration of very large HDL particles (mol/l)	1076	-0.01	-0.07	0.05	0.742	1076	-0.01	-0.07	0.06	0.806	1054	0.00	-0.07	0.06	0.927
Total lipids in very large HDL (mmol/l)	1076	-0.01	-0.07	0.05	0.795	1076	-0.01	-0.07	0.06	0.840	1054	0.00	-0.07	0.06	0.903
Phospholipids in very large HDL (mmol/l)	1076	-0.01	-0.07	0.05	0.664	1076	-0.01	-0.07	0.06	0.775	1054	0.00	-0.07	0.06	0.949
Total cholesterol in very large HDL (mmol/l)	1076	0.00	-0.07	0.06	0.907	1076	0.00	-0.07	0.06	0.893	1054	-0.01	-0.07	0.06	0.835
Cholesterol esters in very large HDL (mmol/l)	1076	0.00	-0.06	0.06	0.965	1076	0.00	-0.07	0.06	0.927	1054	-0.01	-0.08	0.06	0.818
Free cholesterol in very large HDL (mmol/l)	1076	-0.01	-0.07	0.05	0.763	1076	-0.01	-0.07	0.06	0.811	1054	-0.01	-0.07	0.06	0.881
Triglycerides in very large HDL (mmol/l)	1076	0.03	-0.03	0.09	0.354	1076	0.01	-0.05	0.08	0.641	1054	0.01	-0.05	0.07	0.743
Concentration of large HDL particles (mol/l)	1076	-0.02	-0.08	0.04	0.603	1076	-0.01	-0.08	0.05	0.688	1054	-0.01	-0.07	0.06	0.860
Total lipids in large HDL (mmol/l)	1076	-0.02	-0.08	0.04	0.575	1076	-0.01	-0.08	0.05	0.674	1054	-0.01	-0.07	0.06	0.861
Phospholipids in large HDL (mmol/l)	1076	-0.02	-0.08	0.04	0.522	1076	-0.02	-0.08	0.05	0.589	1054	-0.01	-0.07	0.05	0.741
Total cholesterol in large HDL (mmol/l)	1076	-0.02	-0.08	0.04	0.606	1076	-0.01	-0.07	0.05	0.748	1054	0.00	-0.07	0.06	0.967
Cholesterol esters in large HDL (mmol/l)	1076	-0.02	-0.08	0.04	0.600	1076	-0.01	-0.07	0.05	0.745	1054	0.00	-0.07	0.06	0.966
Free cholesterol in large HDL (mmol/l)	1076	-0.01	-0.07	0.05	0.632	1076	-0.01	-0.07	0.05	0.762	1054	0.00	-0.07	0.06	0.969
Triglycerides in large HDL (mmol/l)	1076	0.01	-0.05	0.07	0.754	1076	-0.01	-0.07	0.05	0.830	1054	-0.01	-0.07	0.05	0.790
Concentration of medium HDL particles (mol/l)	1076	-0.03	-0.08	0.03	0.400	1076	-0.03	-0.09	0.03	0.323	1054	-0.03	-0.09	0.03	0.352
Total lipids in medium HDL (mmol/l)	1076	-0.03	-0.09	0.03	0.365	1076	-0.03	-0.09	0.03	0.308	1054	-0.03	-0.09	0.03	0.343
Phospholipids in medium HDL (mmol/l)	1076	-0.03	-0.09	0.03	0.355	1076	-0.03	-0.09	0.03	0.299	1054	-0.03	-0.09	0.03	0.340
Total cholesterol in medium HDL (mmol/l)	1076	-0.03	-0.09	0.03	0.381	1076	-0.03	-0.09	0.03	0.370	1054	-0.03	-0.09	0.04	0.422
Cholesterol esters in medium HDL (mmol/l)	1076	-0.03	-0.09	0.04	0.417	1076	-0.03	-0.09	0.04	0.426	1054	-0.02	-0.09	0.04	0.488
Free cholesterol in medium HDL (mmol/l)	1076	-0.03	-0.09	0.02	0.275	1076	-0.04	-0.10	0.02	0.206	1054	-0.04	-0.10	0.02	0.220
Triglycerides in medium HDL (mmol/l)	1076	0.00	-0.06	0.07	0.910	1076	-0.01	-0.08	0.05	0.684	1054	-0.02	-0.09	0.05	0.525
Concentration of small HDL particles (mol/l)	1076	-0.02	-0.08	0.03	0.403	1076	-0.03	-0.09	0.03	0.292	1054	-0.04	-0.10	0.02	0.194
Total lipids in small HDL (mmol/l)	1076	-0.03	-0.09	0.03	0.332	1076	-0.03	-0.09	0.03	0.265	1054	-0.04	-0.10	0.02	0.224
Phospholipids in small HDL (mmol/l)	1076	-0.02	-0.08	0.04	0.566	1076	-0.03	-0.09	0.04	0.421	1054	-0.03	-0.10	0.03	0.282

S11 Table Associations of change in sedentary time (SED change from age 12y-15y) with metabolic traits at age 15y in ALSPAC

Change in SED from age 12y-15y (per SD-unit increase)

	Adj. for age, sex, ethnicity, maternal education change in wear time, wear month					*Additionally adj. for change in MVPA*					*Additionally adj. for change in FMI*				
**Standardised outcome at age 15y**	**N**	**Beta**	**LCL**	**UCL**	**P-value**	**N**	**Beta**	**LCL**	**UCL**	**P-value**	**N**	**Beta**	**LCL**	**UCL**	**P-value**
Total cholesterol in small HDL (mmol/l)	1076	-0.03	-0.09	0.02	0.239	1076	-0.03	-0.09	0.03	0.267	1054	-0.03	-0.09	0.03	0.339
Cholesterol esters in small HDL (mmol/l)	1076	-0.04	-0.09	0.02	0.208	1076	-0.03	-0.09	0.02	0.253	1054	-0.03	-0.09	0.03	0.348
Free cholesterol in small HDL (mmol/l)	1076	-0.01	-0.07	0.05	0.717	1076	-0.02	-0.08	0.05	0.608	1054	-0.02	-0.09	0.04	0.526
Triglycerides in small HDL (mmol/l)	1076	0.01	-0.05	0.07	0.715	1076	0.00	-0.07	0.06	0.976	1054	-0.01	-0.08	0.05	0.669
Phospholipids to total lipds ratio in chylomicrons and extremely large VLDL (%)	1076	0.00	-0.06	0.06	0.978	1076	-0.01	-0.07	0.05	0.675	1054	-0.02	-0.08	0.04	0.439
Total cholesterol to total lipids ratio in chylomicrons and extremely large VLDL (%)	1076	0.00	-0.06	0.06	0.971	1076	-0.01	-0.07	0.06	0.845	1054	-0.01	-0.08	0.05	0.751
Cholesterol esters to total lipids ratio in chylomicrons and extremely large VLDL (%)	1076	0.00	-0.06	0.06	0.946	1076	-0.01	-0.07	0.06	0.877	1054	-0.01	-0.07	0.06	0.798
Free cholesterol to total lipids ratio in chylomicrons and extremely large VLDL (%)	1076	0.00	-0.06	0.07	0.945	1076	-0.01	-0.07	0.06	0.825	1054	-0.01	-0.08	0.05	0.725
Triglycerides to total lipids ratio in chylomicrons and extremely large VLDL (%)	1076	0.00	-0.04	0.05	0.824	1076	0.01	-0.04	0.05	0.756	1054	0.01	-0.03	0.06	0.617
Phospholipids to total lipds ratio in very large VLDL (%)	1076	0.01	-0.06	0.07	0.811	1076	0.00	-0.07	0.06	0.985	1054	-0.01	-0.08	0.05	0.691
Total cholesterol to total lipids ratio in very large VLDL (%)	1076	0.00	-0.07	0.07	0.969	1076	0.01	-0.04	0.07	0.613	1054	0.02	-0.03	0.08	0.462
Cholesterol esters to total lipids ratio in very large VLDL (%)	1076	0.02	-0.04	0.08	0.578	1076	0.02	-0.04	0.08	0.583	1054	0.03	-0.03	0.09	0.407
Free cholesterol to total lipids ratio in very large VLDL (%)	1076	0.02	-0.04	0.08	0.566	1076	0.02	-0.05	0.08	0.604	1054	0.02	-0.05	0.09	0.605
Triglycerides to total lipids ratio in very large VLDL (%)	1076	-0.02	-0.08	0.04	0.501	1076	-0.02	-0.08	0.04	0.560	1054	-0.02	-0.08	0.04	0.532
Phospholipids to total lipds ratio in large VLDL (%)	1076	0.00	-0.07	0.06	0.880	1076	-0.01	-0.07	0.06	0.824	1054	-0.02	-0.08	0.05	0.627
Total cholesterol to total lipids ratio in large VLDL (%)	1076	0.03	-0.03	0.09	0.388	1076	0.03	-0.04	0.09	0.387	1054	0.03	-0.04	0.09	0.434
Cholesterol esters to total lipids ratio in large VLDL (%)	1076	-0.01	-0.09	0.07	0.717	1076	0.02	-0.02	0.05	0.326	1054	0.02	-0.01	0.06	0.199
Free cholesterol to total lipids ratio in large VLDL (%)	1076	0.00	-0.06	0.07	0.914	1076	0.00	-0.07	0.06	0.915	1054	-0.01	-0.08	0.05	0.657
Triglycerides to total lipids ratio in large VLDL (%)	1076	-0.05	-0.13	0.04	0.296	1076	-0.01	-0.03	0.02	0.603	1054	0.00	-0.03	0.02	0.862
Phospholipids to total lipds ratio in medium VLDL (%)	1076	0.03	-0.04	0.11	0.382	1076	0.04	-0.04	0.12	0.358	1054	0.04	-0.05	0.12	0.367
Total cholesterol to total lipids ratio in medium VLDL (%)	1076	0.04	-0.03	0.10	0.268	1076	0.03	-0.04	0.09	0.410	1054	0.03	-0.03	0.10	0.346
Cholesterol esters to total lipids ratio in medium VLDL (%)	1076	0.03	-0.03	0.09	0.328	1076	0.02	-0.04	0.09	0.464	1054	0.03	-0.03	0.10	0.321
Free cholesterol to total lipids ratio in medium VLDL (%)	1076	0.03	-0.03	0.09	0.361	1076	0.02	-0.04	0.08	0.497	1054	0.01	-0.05	0.07	0.790
Triglycerides to total lipids ratio in medium VLDL (%)	1076	-0.04	-0.10	0.02	0.234	1076	-0.03	-0.10	0.03	0.333	1054	-0.04	-0.10	0.03	0.285
Phospholipids to total lipds ratio in small VLDL (%)	1076	-0.02	-0.08	0.04	0.491	1076	-0.01	-0.07	0.06	0.838	1054	0.00	-0.06	0.07	0.892
Total cholesterol to total lipids ratio in small VLDL (%)	1076	0.01	-0.05	0.07	0.782	1076	0.00	-0.06	0.07	0.994	1054	0.01	-0.06	0.08	0.772
Cholesterol esters to total lipids ratio in small VLDL (%)	1076	0.00	-0.06	0.06	0.933	1076	-0.01	-0.07	0.06	0.837	1054	0.00	-0.06	0.07	0.947
Free cholesterol to total lipids ratio in small VLDL (%)	1076	0.04	-0.04	0.11	0.328	1076	0.04	-0.04	0.12	0.308	1054	0.05	-0.03	0.13	0.229
Triglycerides to total lipids ratio in small VLDL (%)	1076	0.00	-0.06	0.06	0.949	1076	0.00	-0.06	0.07	0.955	1054	-0.01	-0.08	0.05	0.745
Phospholipids to total lipds ratio in very small VLDL (%)	1076	-0.01	-0.06	0.05	0.860	1076	-0.01	-0.07	0.04	0.640	1054	-0.01	-0.07	0.05	0.713
Total cholesterol to total lipids ratio in very small VLDL (%)	1076	-0.01	-0.07	0.06	0.865	1076	0.00	-0.07	0.06	0.918	1054	0.00	-0.06	0.07	0.935
Cholesterol esters to total lipids ratio in very small VLDL (%)	1076	-0.02	-0.08	0.04	0.590	1076	-0.02	-0.08	0.05	0.631	1054	-0.01	-0.08	0.05	0.698
Free cholesterol to total lipids ratio in very small VLDL (%)	1076	0.02	-0.04	0.08	0.451	1076	0.02	-0.04	0.09	0.440	1054	0.03	-0.03	0.10	0.287
Triglycerides to total lipids ratio in very small VLDL (%)	1076	0.01	-0.05	0.07	0.746	1076	0.01	-0.05	0.07	0.654	1054	0.00	-0.06	0.07	0.882
Phospholipids to total lipds ratio in IDL (%)	1076	-0.06	-0.14	0.02	0.119	1076	-0.05	-0.14	0.03	0.220	1054	-0.05	-0.13	0.03	0.249
Total cholesterol to total lipids ratio in IDL (%)	1076	0.03	-0.04	0.09	0.441	1076	0.01	-0.05	0.08	0.681	1054	0.01	-0.05	0.08	0.689
Cholesterol esters to total lipids ratio in IDL (%)	1076	0.03	-0.04	0.10	0.366	1076	0.02	-0.05	0.09	0.581	1054	0.02	-0.06	0.09	0.665
Free cholesterol to total lipids ratio in IDL (%)	1076	-0.01	-0.07	0.04	0.608	1076	-0.01	-0.07	0.05	0.631	1054	0.00	-0.06	0.05	0.872
Triglycerides to total lipids ratio in IDL (%)	1076	0.00	-0.06	0.06	0.997	1076	0.01	-0.05	0.07	0.782	1054	0.01	-0.06	0.07	0.819
Phospholipids to total lipds ratio in large LDL (%)	1076	-0.02	-0.07	0.03	0.467	1076	-0.01	-0.06	0.05	0.848	1054	-0.01	-0.06	0.05	0.826
Total cholesterol to total lipids ratio in large LDL (%)	1076	0.02	-0.03	0.07	0.513	1076	0.00	-0.05	0.06	0.907	1054	0.00	-0.05	0.06	0.881
Cholesterol esters to total lipids ratio in large LDL (%)	1076	0.02	-0.04	0.07	0.511	1076	0.00	-0.05	0.06	0.985	1054	0.00	-0.06	0.05	0.948
Free cholesterol to total lipids ratio in large LDL (%)	1076	-0.01	-0.06	0.05	0.769	1076	0.01	-0.05	0.06	0.808	1054	0.02	-0.04	0.07	0.567
Triglycerides to total lipids ratio in large LDL (%)	1076	-0.01	-0.07	0.05	0.807	1076	0.00	-0.06	0.06	0.985	1054	0.00	-0.06	0.06	0.998
Phospholipids to total lipds ratio in medium LDL (%)	1076	0.00	-0.02	0.02	0.721	1076	0.00	-0.02	0.02	0.868	1054	0.00	-0.02	0.02	0.873
Total cholesterol to total lipids ratio in medium LDL (%)	1076	0.02	-0.04	0.07	0.502	1076	0.00	-0.05	0.06	0.965	1054	0.00	-0.06	0.06	0.985
Cholesterol esters to total lipids ratio in medium LDL (%)	1076	0.01	-0.05	0.07	0.696	1076	-0.01	-0.07	0.05	0.806	1054	-0.01	-0.07	0.05	0.766
Free cholesterol to total lipids ratio in medium LDL (%)	1076	0.00	-0.02	0.02	0.972	1076	0.00	-0.01	0.02	0.563	1054	0.01	-0.01	0.02	0.510

S11 Table Associations of change in sedentary time (SED change from age 12y-15y) with metabolic traits at age 15y in ALSPAC

Change in SED from age 12y-15y (per SD-unit increase)

	Adj. for age, sex, ethnicity, maternal education change in wear time, wear month					*Additionally adj. for change in MVPA*					*Additionally adj. for change in FMI*				
**Standardised outcome at age 15y**	**N**	**Beta**	**LCL**	**UCL**	**P-value**	**N**	**Beta**	**LCL**	**UCL**	**P-value**	**N**	**Beta**	**LCL**	**UCL**	**P-value**
Triglycerides to total lipids ratio in medium LDL (%)	1076	-0.02	-0.09	0.04	0.506	1076	-0.01	-0.08	0.05	0.683	1054	-0.01	-0.08	0.05	0.727
Phospholipids to total lipds ratio in small LDL (%)	1076	-0.01	-0.04	0.03	0.709	1076	0.00	-0.03	0.04	0.871	1054	0.00	-0.03	0.04	0.882
Total cholesterol to total lipids ratio in small LDL (%)	1076	0.01	-0.04	0.07	0.682	1076	0.00	-0.06	0.05	0.910	1054	0.00	-0.06	0.06	0.981
Cholesterol esters to total lipids ratio in small LDL (%)	1076	0.01	-0.05	0.06	0.850	1076	-0.01	-0.07	0.05	0.732	1054	-0.01	-0.07	0.05	0.742
Free cholesterol to total lipids ratio in small LDL (%)	1076	0.00	-0.03	0.03	0.853	1076	0.01	-0.02	0.04	0.524	1054	0.01	-0.02	0.05	0.452
Triglycerides to total lipids ratio in small LDL (%)	1076	-0.01	-0.07	0.05	0.854	1076	0.00	-0.07	0.06	0.906	1054	-0.01	-0.07	0.05	0.744
Phospholipids to total lipds ratio in very large HDL (%)	1076	-0.03	-0.09	0.03	0.316	1076	-0.02	-0.08	0.04	0.445	1054	0.00	-0.06	0.06	0.981
Total cholesterol to total lipids ratio in very large HDL (%)	1076	0.02	-0.03	0.08	0.415	1076	0.02	-0.04	0.08	0.498	1054	0.00	-0.06	0.05	0.913
Cholesterol esters to total lipids ratio in very large HDL (%)	1076	0.02	-0.03	0.08	0.408	1076	0.02	-0.04	0.08	0.494	1054	0.00	-0.06	0.05	0.929
Free cholesterol to total lipids ratio in very large HDL (%)	1076	-0.01	-0.08	0.05	0.697	1076	-0.01	-0.08	0.06	0.762	1054	-0.01	-0.07	0.06	0.878
Triglycerides to total lipids ratio in very large HDL (%)	1076	0.04	-0.03	0.10	0.245	1076	0.02	-0.04	0.09	0.535	1054	0.01	-0.05	0.08	0.711
Phospholipids to total lipds ratio in large HDL (%)	1076	0.00	-0.06	0.05	0.912	1076	-0.01	-0.07	0.04	0.634	1054	-0.03	-0.09	0.03	0.291
Total cholesterol to total lipids ratio in large HDL (%)	1076	-0.01	-0.07	0.05	0.764	1076	0.00	-0.06	0.07	0.891	1054	0.02	-0.04	0.08	0.484
Cholesterol esters to total lipids ratio in large HDL (%)	1076	-0.01	-0.07	0.05	0.743	1076	0.00	-0.06	0.07	0.913	1054	0.02	-0.04	0.08	0.500
Free cholesterol to total lipids ratio in large HDL (%)	1076	0.00	-0.06	0.05	0.903	1076	0.01	-0.05	0.07	0.837	1054	0.02	-0.04	0.08	0.530
Triglycerides to total lipids ratio in large HDL (%)	1076	0.03	-0.03	0.10	0.344	1076	0.02	-0.05	0.08	0.622	1054	0.00	-0.07	0.07	0.941
Phospholipids to total lipds ratio in medium HDL (%)	1076	-0.02	-0.09	0.04	0.475	1076	-0.03	-0.09	0.04	0.439	1054	-0.02	-0.09	0.04	0.507
Total cholesterol to total lipids ratio in medium HDL (%)	1076	0.01	-0.05	0.08	0.740	1076	0.02	-0.05	0.09	0.530	1054	0.02	-0.04	0.09	0.479
Cholesterol esters to total lipids ratio in medium HDL (%)	1076	0.02	-0.05	0.09	0.628	1076	0.03	-0.04	0.11	0.421	1054	0.03	-0.04	0.11	0.373
Free cholesterol to total lipids ratio in medium HDL (%)	1076	-0.04	-0.15	0.08	0.554	1076	-0.04	-0.17	0.08	0.500	1054	-0.05	-0.17	0.08	0.472
Triglycerides to total lipids ratio in medium HDL (%)	1076	0.01	-0.05	0.08	0.693	1076	0.00	-0.07	0.07	0.966	1054	-0.01	-0.08	0.06	0.743
Phospholipids to total lipds ratio in small HDL (%)	1076	0.02	-0.04	0.08	0.434	1076	0.02	-0.04	0.08	0.554	1054	0.01	-0.05	0.07	0.757
Total cholesterol to total lipids ratio in small HDL (%)	1076	-0.03	-0.08	0.03	0.356	1076	-0.02	-0.08	0.04	0.514	1054	-0.01	-0.07	0.05	0.776
Cholesterol esters to total lipids ratio in small HDL (%)	1076	-0.03	-0.09	0.03	0.281	1076	-0.02	-0.08	0.03	0.417	1054	-0.01	-0.07	0.04	0.647
Free cholesterol to total lipids ratio in small HDL (%)	1076	0.04	-0.02	0.11	0.204	1076	0.04	-0.02	0.11	0.219	1054	0.04	-0.03	0.11	0.243
Triglycerides to total lipids ratio in small HDL (%)	1076	0.02	-0.04	0.08	0.456	1076	0.01	-0.05	0.08	0.697	1054	0.00	-0.07	0.06	0.973
Mean diameter for VLDL particles (nm)	1076	0.01	-0.05	0.07	0.707	1076	0.00	-0.06	0.07	0.969	1054	-0.02	-0.08	0.05	0.601
Mean diameter for LDL particles (nm)	1076	0.00	-0.06	0.05	0.977	1076	0.01	-0.05	0.06	0.788	1054	0.02	-0.03	0.08	0.422
Mean diameter for HDL particles (nm)	1076	-0.01	-0.07	0.05	0.793	1076	0.00	-0.07	0.06	0.885	1054	0.01	-0.06	0.07	0.869
Serum total cholesterol (mmol/l)	1076	0.00	-0.05	0.06	0.902	1076	-0.01	-0.07	0.05	0.713	1054	-0.01	-0.08	0.05	0.631
Total cholesterol in VLDL (mmol/l)	1076	0.03	-0.04	0.09	0.392	1076	0.01	-0.06	0.07	0.844	1054	-0.01	-0.07	0.06	0.879
Remnant cholesterol (non-HDL, non-LDL -cholesterol) (mmol/l)	1076	0.02	-0.04	0.08	0.468	1076	0.00	-0.06	0.07	0.943	1054	-0.01	-0.07	0.06	0.862
Total cholesterol in LDL (mmol/l)	1076	0.00	-0.05	0.06	0.898	1076	-0.01	-0.07	0.05	0.730	1054	-0.02	-0.08	0.05	0.639
Total cholesterol in HDL (mmol/l)	1076	-0.02	-0.08	0.04	0.489	1076	-0.02	-0.08	0.04	0.555	1054	-0.01	-0.08	0.05	0.663
Total cholesterol in HDL2 (mmol/l)	1076	-0.03	-0.09	0.03	0.384	1076	-0.02	-0.09	0.04	0.496	1054	-0.02	-0.08	0.05	0.628
Total cholesterol in HDL3 (mmol/l)	1076	-0.01	-0.07	0.05	0.754	1076	-0.01	-0.07	0.05	0.704	1054	-0.01	-0.07	0.05	0.753
Esterified cholesterol (mmol/l)	1076	0.00	-0.06	0.05	0.912	1076	-0.02	-0.08	0.04	0.548	1054	-0.02	-0.08	0.04	0.474
Free cholesterol (mmol/l)	1076	0.02	-0.04	0.08	0.525	1076	0.01	-0.06	0.07	0.864	1054	0.00	-0.06	0.06	0.942
Serum total triglycerides (mmol/l)	1076	0.02	-0.04	0.08	0.449	1076	0.01	-0.06	0.07	0.839	1054	-0.01	-0.07	0.05	0.749
Triglycerides in VLDL (mmol/l)	1076	0.02	-0.04	0.08	0.434	1076	0.01	-0.06	0.07	0.800	1054	-0.01	-0.07	0.05	0.765
Triglycerides in LDL (mmol/l)	1076	0.00	-0.06	0.07	0.917	1076	-0.01	-0.07	0.06	0.849	1054	-0.01	-0.08	0.06	0.736
Triglycerides in HDL (mmol/l)	1076	0.01	-0.05	0.08	0.649	1076	0.00	-0.07	0.06	0.914	1054	-0.01	-0.08	0.05	0.691
Diacylglycerol (mmol/l)	1044	-0.01	-0.07	0.05	0.753	1044	-0.02	-0.08	0.05	0.618	1023	-0.02	-0.09	0.04	0.427
Ratio of diacylglycerol to triglycerides	1044	-0.03	-0.09	0.02	0.226	1044	-0.03	-0.09	0.03	0.328	1023	-0.03	-0.09	0.02	0.251
Total phosphoglycerides (mmol/l)	1076	0.01	-0.05	0.07	0.710	1076	0.00	-0.06	0.07	0.943	1054	0.00	-0.07	0.06	0.942
Ratio of triglycerides to phosphoglycerides	1076	0.01	-0.05	0.07	0.772	1076	0.00	-0.06	0.06	0.992	1054	-0.01	-0.07	0.05	0.654
Phosphatidylcholine and other cholines (mmol/l)	1056	0.03	-0.03	0.09	0.335	1056	0.01	-0.05	0.08	0.642	1034	0.01	-0.05	0.08	0.722
Total cholines (mmol/l)	1073	0.01	-0.05	0.07	0.660	1073	0.01	-0.06	0.07	0.872	1051	0.00	-0.06	0.07	0.967

S11 Table Associations of change in sedentary time (SED change from age 12y-15y) with metabolic traits at age 15y in ALSPAC

Change in SED from age 12y-15y (per SD-unit increase)

	Adj. for age, sex, ethnicity, maternal education change in wear time, wear month					*Additionally adj. for change in MVPA*					*Additionally adj. for change in FMI*				
**Standardised outcome at age 15y**	**N**	**Beta**	**LCL**	**UCL**	**P-value**	**N**	**Beta**	**LCL**	**UCL**	**P-value**	**N**	**Beta**	**LCL**	**UCL**	**P-value**
Apolipoprotein A-I (g/l)	1076	-0.01	-0.07	0.04	0.635	1076	-0.02	-0.08	0.04	0.537	1054	-0.02	-0.08	0.04	0.539
Apolipoprotein B (g/l)	1076	0.03	-0.04	0.09	0.414	1076	0.01	-0.06	0.07	0.865	1054	-0.01	-0.07	0.06	0.867
Ratio of apolipoprotein B to apolipoprotein A-I	1076	0.03	-0.03	0.10	0.287	1076	0.02	-0.05	0.08	0.602	1054	0.01	-0.06	0.07	0.863
Total fatty acids (mmol/l)	1076	0.01	-0.05	0.07	0.672	1076	0.00	-0.07	0.06	0.935	1054	-0.01	-0.07	0.05	0.763
Estimated description of fatty acid chain length, not actual carbon number	1072	-0.01	-0.07	0.05	0.717	1072	-0.01	-0.07	0.05	0.780	1050	-0.02	-0.08	0.04	0.521
Estimated degree of unsaturation	1075	-0.02	-0.09	0.04	0.486	1075	-0.02	-0.09	0.05	0.536	1053	-0.02	-0.09	0.05	0.617
22:6, docosahexaenoic acid (mmol/l)	1076	-0.05	-0.10	0.01	0.114	1076	-0.06	-0.12	0.00	0.048	1054	-0.06	-0.12	0.00	0.034
18:2, linoleic acid (mmol/l)	1073	0.02	-0.04	0.07	0.589	1073	0.01	-0.05	0.07	0.777	1051	0.00	-0.06	0.07	0.883
Conjugated linoleic acid (mmol/l)	1076	0.00	-0.06	0.05	0.876	1076	-0.01	-0.06	0.05	0.846	1054	0.00	-0.06	0.06	0.928
Omega-3 fatty acids (mmol/l)	1074	-0.05	-0.11	0.01	0.075	1074	-0.07	-0.13	0.00	0.038	1052	-0.08	-0.14	-0.01	0.016
Omega-6 fatty acids (mmol/l)	1075	0.01	-0.05	0.07	0.773	1075	0.00	-0.06	0.06	0.977	1053	-0.01	-0.07	0.06	0.867
Polyunsaturated fatty acids (mmol/l)	1073	0.00	-0.06	0.05	0.922	1073	-0.01	-0.07	0.05	0.692	1051	-0.02	-0.08	0.04	0.574
Monounsaturated fatty acids; 16:1, 18:1 (mmol/l)	1073	0.04	-0.03	0.10	0.254	1073	0.02	-0.04	0.08	0.583	1051	0.01	-0.05	0.07	0.746
Saturated fatty acids (mmol/l)	1072	0.00	-0.06	0.07	0.930	1072	-0.01	-0.08	0.06	0.749	1050	-0.02	-0.09	0.05	0.611
Ratio of 22:6 docosahexaenoic acid to total fatty acids (%)	1076	-0.06	-0.12	0.00	0.046	1076	-0.07	-0.13	-0.01	0.027	1054	-0.07	-0.13	-0.01	0.024
Ratio of 18:2 linoleic acid to total fatty acids (%)	1073	0.01	-0.05	0.08	0.697	1073	0.02	-0.04	0.09	0.486	1051	0.03	-0.04	0.10	0.394
Ratio of conjugated linoleic acid to total fatty acids (%)	1076	-0.01	-0.07	0.05	0.770	1076	-0.01	-0.06	0.05	0.819	1054	0.00	-0.06	0.06	0.921
Ratio of omega-3 fatty acids to total fatty acids (%)	1074	-0.07	-0.13	-0.01	0.018	1074	-0.08	-0.14	-0.02	0.014	1052	-0.09	-0.15	-0.02	0.007
Ratio of omega-6 fatty acids to total fatty acids (%)	1075	-0.01	-0.07	0.06	0.844	1075	0.01	-0.06	0.07	0.867	1053	0.01	-0.05	0.08	0.696
Ratio of polyunsaturated fatty acids to total fatty acids (%)	1073	-0.02	-0.08	0.04	0.531	1073	-0.01	-0.08	0.05	0.737	1051	-0.01	-0.07	0.06	0.849
Ratio of monounsaturated fatty acids to total fatty acids (%)	1073	0.04	-0.03	0.10	0.273	1073	0.02	-0.04	0.09	0.463	1051	0.02	-0.05	0.09	0.558
Ratio of saturated fatty acids to total fatty acids (%)	1072	-0.02	-0.09	0.05	0.581	1072	-0.02	-0.09	0.06	0.657	1050	-0.02	-0.09	0.06	0.672
Insulin (mu/l)	1118	-0.01	-0.05	0.02	0.447	1118	-0.01	-0.05	0.03	0.599	1095	-0.02	-0.06	0.02	0.361
Glucose (mmol/l)	1074	0.00	-0.06	0.06	0.879	1074	0.00	-0.06	0.07	0.960	1052	0.00	-0.06	0.07	0.913
Lactate (mmol/l)	1074	0.01	-0.05	0.07	0.703	1074	0.01	-0.05	0.07	0.720	1052	0.01	-0.06	0.07	0.840
Pyruvate (mmol/l)	1073	-0.01	-0.07	0.05	0.778	1073	0.00	-0.06	0.06	0.923	1051	0.00	-0.06	0.06	0.982
Citrate (mmol/l)	1070	0.01	-0.06	0.07	0.820	1070	0.01	-0.06	0.08	0.769	1048	0.02	-0.05	0.09	0.571
Alanine (mmol/l)	1076	0.02	-0.04	0.08	0.512	1076	0.03	-0.03	0.10	0.310	1054	0.03	-0.04	0.09	0.392
Glutamine (mmol/l)	1076	-0.04	-0.09	0.01	0.154	1076	-0.03	-0.09	0.02	0.246	1054	-0.03	-0.09	0.03	0.282
Histidine (mmol/l)	1015	0.05	-0.02	0.11	0.150	1015	0.04	-0.02	0.11	0.192	994	0.03	-0.03	0.10	0.321
Isoleucine (mmol/l)	1076	-0.02	-0.08	0.04	0.530	1076	-0.02	-0.08	0.04	0.488	1054	-0.02	-0.08	0.03	0.423
Leucine (mmol/l)	1076	-0.03	-0.08	0.02	0.207	1076	-0.03	-0.08	0.02	0.301	1054	-0.03	-0.08	0.02	0.281
Valine (mmol/l)	1076	-0.02	-0.07	0.04	0.553	1076	-0.02	-0.08	0.04	0.530	1054	-0.02	-0.08	0.04	0.464
Phenylalanine (mmol/l)	1075	-0.09	-0.15	-0.03	0.003	1075	-0.09	-0.15	-0.03	0.005	1053	-0.10	-0.16	-0.03	0.003
Tyrosine (mmol/l)	1070	-0.06	-0.12	0.00	0.050	1070	-0.07	-0.13	-0.01	0.026	1048	-0.09	-0.15	-0.02	0.006
Acetate (mmol/l)	1075	0.04	-0.02	0.10	0.188	1075	0.04	-0.02	0.11	0.167	1053	0.06	-0.01	0.12	0.087
Acetoacetate (mmol/l)	1076	-0.04	-0.11	0.04	0.316	1076	-0.02	-0.09	0.05	0.581	1054	-0.01	-0.08	0.06	0.783
3-hydroxybutyrate (mmol/l)	1075	-0.03	-0.10	0.03	0.328	1075	-0.03	-0.09	0.04	0.414	1053	-0.02	-0.09	0.05	0.557
Creatinine (mmol/l)	1075	0.03	-0.03	0.09	0.270	1075	0.03	-0.03	0.09	0.328	1053	0.04	-0.02	0.10	0.223
Albumin (signal area)	1076	0.00	-0.06	0.06	0.983	1076	-0.01	-0.07	0.06	0.884	1054	-0.01	-0.08	0.06	0.772
Glycoprotein acetyls, mainly a1-acid glycoprotein (mmol/l)	1075	-0.05	-0.11	0.01	0.100	1075	-0.07	-0.13	0.00	0.047	1053	-0.08	-0.15	-0.01	0.019
C-reactive protein (mg/l)	1120	-0.04	-0.11	0.04	0.341	1120	-0.04	-0.13	0.05	0.349	1097	-0.04	-0.13	0.05	0.349

Change in SED from age 12y-15y (per SD-unit increase)

Complete case sample

Adj. for age, sex, ethnicity, maternal education *Additionally adj. for change in MVPA* *Additionally adj. for change in FMI*

S11 Table Associations of change in sedentary time (SED change from age 12y-15y) with metabolic traits at age 15y in ALSPAC

Change in SED from age 12y-15y (per SD-unit increase)

Standardised outcome at age 15y | N | Beta | LCL | UCL | P-value | N | Beta | LCL | UCL | P-value | N | Beta | LCL | UCL | P-value
change in wear time, wear month

	Adj. for age, sex, ethnicity, maternal education change in wear time, wear month					*Additionally adj. for change in MVPA*					*Additionally adj. for change in FMI*				
**Standardised outcome at age 15y**	**N**	**Beta**	**LCL**	**UCL**	**P-value**	**N**	**Beta**	**LCL**	**UCL**	**P-value**	**N**	**Beta**	**LCL**	**UCL**	**P-value**
Systolic blood pressure (mmHg)	755	0.02	-0.05	0.09	0.536	755	0.04	-0.02	0.11	0.209	755	0.04	-0.03	0.11	0.220
Diastolic blood pressure (mmHg)	755	0.02	-0.05	0.08	0.614	755	0.02	-0.05	0.10	0.492	755	0.02	-0.05	0.10	0.491
Concentration of chylomicrons and extremely large VLDL particles (mol/l)	755	0.02	-0.05	0.09	0.627	755	0.01	-0.06	0.08	0.818	755	0.00	-0.07	0.07	0.970
Total lipids in chylomicrons and extremely large VLDL (mmol/l)	755	0.01	-0.06	0.08	0.682	755	0.00	-0.07	0.08	0.900	755	0.00	-0.08	0.07	0.950
Phospholipids in chylomicrons and extremely large VLDL (mmol/l)	755	0.01	-0.05	0.08	0.676	755	0.00	-0.07	0.08	0.905	755	0.00	-0.07	0.07	0.948
Total cholesterol in chylomicrons and extremely large VLDL (mmol/l)	755	0.02	-0.05	0.09	0.659	755	0.01	-0.07	0.08	0.825	755	0.00	-0.07	0.07	0.983
Cholesterol esters in chylomicrons and extremely large VLDL (mmol/l)	755	0.02	-0.05	0.09	0.614	755	0.01	-0.06	0.09	0.728	755	0.01	-0.07	0.08	0.889
Free cholesterol in chylomicrons and extremely large VLDL (mmol/l)	755	0.01	-0.06	0.08	0.730	755	0.00	-0.07	0.08	0.945	755	0.00	-0.08	0.07	0.913
Triglycerides in chylomicrons and extremely large VLDL (mmol/l)	755	0.01	-0.06	0.08	0.690	755	0.00	-0.07	0.08	0.917	755	0.00	-0.08	0.07	0.936
Concentration of very large VLDL particles (mol/l)	755	0.02	-0.05	0.09	0.540	755	0.01	-0.06	0.09	0.735	755	0.01	-0.07	0.08	0.871
Total lipids in very large VLDL (mmol/l)	755	0.02	-0.05	0.09	0.561	755	0.01	-0.06	0.09	0.765	755	0.00	-0.07	0.08	0.902
Phospholipids in very large VLDL (mmol/l)	755	0.02	-0.05	0.09	0.614	755	0.01	-0.07	0.08	0.817	755	0.00	-0.07	0.08	0.955
Total cholesterol in very large VLDL (mmol/l)	755	0.02	-0.05	0.09	0.615	755	0.01	-0.07	0.08	0.800	755	0.00	-0.07	0.08	0.951
Cholesterol esters in very large VLDL (mmol/l)	755	0.02	-0.05	0.09	0.585	755	0.01	-0.06	0.09	0.758	755	0.00	-0.07	0.08	0.909
Free cholesterol in very large VLDL (mmol/l)	755	0.02	-0.05	0.09	0.652	755	0.01	-0.07	0.08	0.849	755	0.00	-0.07	0.07	0.998
Triglycerides in very large VLDL (mmol/l)	755	0.02	-0.05	0.09	0.534	755	0.01	-0.06	0.09	0.742	755	0.01	-0.07	0.08	0.874
Concentration of large VLDL particles (mol/l)	755	0.02	-0.05	0.10	0.518	755	0.01	-0.06	0.09	0.725	755	0.01	-0.07	0.08	0.858
Total lipids in large VLDL (mmol/l)	755	0.02	-0.05	0.10	0.524	755	0.01	-0.06	0.09	0.731	755	0.01	-0.07	0.08	0.865
Phospholipids in large VLDL (mmol/l)	755	0.02	-0.05	0.10	0.525	755	0.01	-0.06	0.09	0.729	755	0.01	-0.07	0.08	0.864
Total cholesterol in large VLDL (mmol/l)	755	0.03	-0.04	0.10	0.447	755	0.02	-0.06	0.10	0.619	755	0.01	-0.06	0.09	0.753
Cholesterol esters in large VLDL (mmol/l)	755	0.03	-0.04	0.10	0.421	755	0.02	-0.06	0.10	0.571	755	0.02	-0.06	0.09	0.702
Free cholesterol in large VLDL (mmol/l)	755	0.03	-0.05	0.10	0.480	755	0.02	-0.06	0.09	0.674	755	0.01	-0.07	0.08	0.808
Triglycerides in large VLDL (mmol/l)	755	0.02	-0.05	0.09	0.557	755	0.01	-0.07	0.09	0.777	755	0.00	-0.07	0.08	0.910
Concentration of medium VLDL particles (mol/l)	755	0.03	-0.05	0.10	0.445	755	0.02	-0.06	0.10	0.642	755	0.01	-0.07	0.09	0.771
Total lipids in medium VLDL (mmol/l)	755	0.03	-0.05	0.10	0.437	755	0.02	-0.06	0.10	0.625	755	0.01	-0.07	0.09	0.755
Phospholipids in medium VLDL (mmol/l)	755	0.03	-0.04	0.11	0.361	755	0.03	-0.05	0.10	0.533	755	0.02	-0.06	0.10	0.656
Total cholesterol in medium VLDL (mmol/l)	755	0.04	-0.04	0.11	0.320	755	0.03	-0.05	0.11	0.445	755	0.02	-0.06	0.10	0.564
Cholesterol esters in medium VLDL (mmol/l)	755	0.04	-0.04	0.12	0.305	755	0.04	-0.05	0.12	0.395	755	0.03	-0.05	0.11	0.507
Free cholesterol in medium VLDL (mmol/l)	755	0.03	-0.04	0.11	0.364	755	0.02	-0.05	0.10	0.537	755	0.02	-0.06	0.09	0.660
Triglycerides in medium VLDL (mmol/l)	755	0.02	-0.05	0.10	0.547	755	0.01	-0.07	0.09	0.768	755	0.01	-0.07	0.08	0.899
Concentration of small VLDL particles (mol/l)	755	0.04	-0.03	0.12	0.253	755	0.04	-0.04	0.11	0.380	755	0.03	-0.05	0.11	0.484
Total lipids in small VLDL (mmol/l)	755	0.05	-0.03	0.12	0.235	755	0.04	-0.04	0.12	0.354	755	0.03	-0.05	0.11	0.460
Phospholipids in small VLDL (mmol/l)	755	0.05	-0.03	0.12	0.196	755	0.04	-0.04	0.12	0.294	755	0.03	-0.04	0.11	0.386
Total cholesterol in small VLDL (mmol/l)	755	0.05	-0.02	0.13	0.180	755	0.04	-0.03	0.12	0.267	755	0.04	-0.04	0.11	0.363
Cholesterol esters in small VLDL (mmol/l)	755	0.04	-0.03	0.12	0.236	755	0.04	-0.04	0.12	0.329	755	0.03	-0.05	0.11	0.439
Free cholesterol in small VLDL (mmol/l)	755	0.06	-0.02	0.13	0.131	755	0.05	-0.03	0.13	0.203	755	0.04	-0.03	0.12	0.275
Triglycerides in small VLDL (mmol/l)	755	0.03	-0.04	0.11	0.387	755	0.02	-0.05	0.10	0.544	755	0.02	-0.06	0.10	0.654
Concentration of very small VLDL particles (mol/l)	755	0.05	-0.02	0.13	0.152	755	0.05	-0.02	0.13	0.179	755	0.05	-0.03	0.12	0.237
Total lipids in very small VLDL (mmol/l)	755	0.05	-0.02	0.12	0.186	755	0.04	-0.03	0.12	0.248	755	0.04	-0.04	0.11	0.330
Phospholipids in very small VLDL (mmol/l)	755	0.04	-0.03	0.11	0.276	755	0.04	-0.04	0.11	0.307	755	0.03	-0.04	0.11	0.389
Total cholesterol in very small VLDL (mmol/l)	755	0.04	-0.03	0.12	0.225	755	0.04	-0.04	0.11	0.312	755	0.03	-0.04	0.10	0.405
Cholesterol esters in very small VLDL (mmol/l)	755	0.04	-0.03	0.11	0.305	755	0.03	-0.04	0.10	0.418	755	0.02	-0.05	0.10	0.533
Free cholesterol in very small VLDL (mmol/l)	755	0.05	-0.02	0.13	0.136	755	0.05	-0.02	0.13	0.183	755	0.04	-0.03	0.12	0.238
Triglycerides in very small VLDL (mmol/l)	755	0.04	-0.03	0.12	0.255	755	0.04	-0.04	0.12	0.299	755	0.04	-0.04	0.11	0.368
Concentration of IDL particles (mol/l)	755	0.04	-0.03	0.11	0.318	755	0.04	-0.04	0.11	0.346	755	0.03	-0.04	0.10	0.424

S11 Table Associations of change in sedentary time (SED change from age 12y-15y) with metabolic traits at age 15y in ALSPAC

Change in SED from age 12y-15y (per SD-unit increase)

	Adj. for age, sex, ethnicity, maternal education change in wear time, wear month					*Additionally adj. for change in MVPA*					*Additionally adj. for change in FMI*				
**Standardised outcome at age 15y**	**N**	**Beta**	**LCL**	**UCL**	**P-value**	**N**	**Beta**	**LCL**	**UCL**	**P-value**	**N**	**Beta**	**LCL**	**UCL**	**P-value**
Total lipids in IDL (mmol/l)	755	0.04	-0.03	0.11	0.292	755	0.04	-0.04	0.11	0.313	755	0.03	-0.04	0.11	0.392
Phospholipids in IDL (mmol/l)	755	0.03	-0.04	0.10	0.460	755	0.03	-0.05	0.10	0.471	755	0.02	-0.05	0.10	0.555
Total cholesterol in IDL (mmol/l)	755	0.04	-0.03	0.11	0.249	755	0.04	-0.03	0.11	0.278	755	0.03	-0.04	0.11	0.357
Cholesterol esters in IDL (mmol/l)	755	0.04	-0.03	0.11	0.210	755	0.04	-0.03	0.12	0.247	755	0.04	-0.04	0.11	0.326
Free cholesterol in IDL (mmol/l)	755	0.03	-0.04	0.10	0.398	755	0.03	-0.04	0.10	0.399	755	0.03	-0.05	0.10	0.472
Triglycerides in IDL (mmol/l)	755	0.03	-0.05	0.11	0.463	755	0.03	-0.05	0.11	0.432	755	0.03	-0.05	0.11	0.473
Concentration of large LDL particles (mol/l)	755	0.03	-0.04	0.10	0.400	755	0.03	-0.04	0.10	0.422	755	0.02	-0.05	0.10	0.512
Total lipids in large LDL (mmol/l)	755	0.03	-0.04	0.10	0.416	755	0.03	-0.04	0.10	0.431	755	0.02	-0.05	0.10	0.524
Phospholipids in large LDL (mmol/l)	755	0.03	-0.04	0.10	0.408	755	0.03	-0.04	0.10	0.422	755	0.02	-0.05	0.10	0.522
Total cholesterol in large LDL (mmol/l)	755	0.03	-0.04	0.10	0.395	755	0.03	-0.04	0.10	0.411	755	0.02	-0.05	0.10	0.505
Cholesterol esters in large LDL (mmol/l)	755	0.03	-0.04	0.10	0.390	755	0.03	-0.04	0.10	0.411	755	0.02	-0.05	0.10	0.510
Free cholesterol in large LDL (mmol/l)	755	0.03	-0.04	0.10	0.419	755	0.03	-0.04	0.10	0.418	755	0.03	-0.05	0.10	0.498
Triglycerides in large LDL (mmol/l)	755	0.01	-0.07	0.09	0.747	755	0.01	-0.07	0.09	0.745	755	0.01	-0.07	0.09	0.779
Concentration of medium LDL particles (mol/l)	755	0.03	-0.04	0.10	0.427	755	0.03	-0.05	0.10	0.459	755	0.02	-0.05	0.10	0.558
Total lipids in medium LDL (mmol/l)	755	0.03	-0.04	0.10	0.424	755	0.03	-0.05	0.10	0.454	755	0.02	-0.05	0.10	0.554
Phospholipids in medium LDL (mmol/l)	755	0.04	-0.03	0.11	0.281	755	0.04	-0.04	0.11	0.315	755	0.03	-0.04	0.10	0.411
Total cholesterol in medium LDL (mmol/l)	755	0.03	-0.04	0.10	0.424	755	0.03	-0.05	0.10	0.458	755	0.02	-0.05	0.10	0.559
Cholesterol esters in medium LDL (mmol/l)	755	0.02	-0.05	0.09	0.509	755	0.02	-0.05	0.10	0.544	755	0.02	-0.06	0.09	0.650
Free cholesterol in medium LDL (mmol/l)	755	0.05	-0.02	0.12	0.182	755	0.05	-0.03	0.12	0.206	755	0.04	-0.03	0.11	0.270
Triglycerides in medium LDL (mmol/l)	755	0.00	-0.08	0.08	0.999	755	0.00	-0.08	0.08	0.962	755	0.00	-0.08	0.08	0.988
Concentration of small LDL particles (mol/l)	755	0.03	-0.04	0.10	0.353	755	0.03	-0.04	0.10	0.424	755	0.02	-0.05	0.10	0.524
Total lipids in small LDL (mmol/l)	755	0.03	-0.04	0.10	0.412	755	0.03	-0.05	0.10	0.463	755	0.02	-0.05	0.10	0.568
Phospholipids in small LDL (mmol/l)	755	0.04	-0.03	0.11	0.266	755	0.04	-0.04	0.11	0.338	755	0.03	-0.04	0.10	0.439
Total cholesterol in small LDL (mmol/l)	755	0.03	-0.04	0.10	0.450	755	0.03	-0.05	0.10	0.493	755	0.02	-0.05	0.09	0.596
Cholesterol esters in small LDL (mmol/l)	755	0.02	-0.05	0.09	0.561	755	0.02	-0.05	0.10	0.594	755	0.01	-0.06	0.09	0.698
Free cholesterol in small LDL (mmol/l)	755	0.05	-0.02	0.12	0.145	755	0.05	-0.02	0.12	0.195	755	0.04	-0.03	0.11	0.262
Triglycerides in small LDL (mmol/l)	755	0.01	-0.06	0.09	0.720	755	0.01	-0.06	0.09	0.745	755	0.01	-0.07	0.09	0.819
Concentration of very large HDL particles (mol/l)	755	-0.01	-0.08	0.07	0.880	755	0.00	-0.08	0.08	0.979	755	0.00	-0.08	0.08	0.976
Total lipids in very large HDL (mmol/l)	755	0.00	-0.08	0.07	0.902	755	0.00	-0.08	0.08	0.962	755	0.00	-0.08	0.08	1.000
Phospholipids in very large HDL (mmol/l)	755	-0.01	-0.09	0.06	0.774	755	0.00	-0.08	0.08	0.930	755	0.00	-0.08	0.08	0.990
Total cholesterol in very large HDL (mmol/l)	755	0.00	-0.07	0.08	0.974	755	0.00	-0.08	0.08	0.975	755	0.00	-0.08	0.08	0.989
Cholesterol esters in very large HDL (mmol/l)	755	0.00	-0.07	0.08	0.932	755	0.00	-0.08	0.08	0.975	755	0.00	-0.08	0.08	0.980
Free cholesterol in very large HDL (mmol/l)	755	0.00	-0.08	0.07	0.918	755	0.00	-0.08	0.08	0.974	755	0.00	-0.08	0.08	0.988
Triglycerides in very large HDL (mmol/l)	755	0.02	-0.05	0.09	0.540	755	0.02	-0.06	0.09	0.642	755	0.02	-0.06	0.09	0.690
Concentration of large HDL particles (mol/l)	755	-0.01	-0.08	0.07	0.842	755	0.00	-0.08	0.08	0.980	755	0.00	-0.08	0.08	0.956
Total lipids in large HDL (mmol/l)	755	-0.01	-0.08	0.06	0.807	755	0.00	-0.08	0.08	0.958	755	0.00	-0.08	0.08	0.973
Phospholipids in large HDL (mmol/l)	755	-0.01	-0.08	0.06	0.773	755	0.00	-0.08	0.07	0.920	755	0.00	-0.08	0.08	0.980
Total cholesterol in large HDL (mmol/l)	755	-0.01	-0.08	0.07	0.822	755	0.00	-0.08	0.08	0.982	755	0.00	-0.08	0.08	0.938
Cholesterol esters in large HDL (mmol/l)	755	-0.01	-0.08	0.07	0.811	755	0.00	-0.08	0.08	0.970	755	0.00	-0.08	0.08	0.949
Free cholesterol in large HDL (mmol/l)	755	-0.01	-0.08	0.07	0.870	755	0.00	-0.08	0.08	0.970	755	0.01	-0.07	0.08	0.897
Triglycerides in large HDL (mmol/l)	755	0.01	-0.06	0.08	0.794	755	0.01	-0.07	0.08	0.876	755	0.00	-0.07	0.08	0.908
Concentration of medium HDL particles (mol/l)	755	0.00	-0.07	0.07	0.993	755	0.00	-0.07	0.08	0.957	755	0.00	-0.07	0.08	0.976
Total lipids in medium HDL (mmol/l)	755	0.00	-0.07	0.07	0.956	755	0.00	-0.07	0.08	0.986	755	0.00	-0.07	0.07	0.991
Phospholipids in medium HDL (mmol/l)	755	0.00	-0.07	0.07	0.889	755	0.00	-0.08	0.07	0.944	755	0.00	-0.08	0.07	0.933
Total cholesterol in medium HDL (mmol/l)	755	0.00	-0.07	0.07	0.993	755	0.00	-0.07	0.08	0.919	755	0.01	-0.07	0.08	0.899
Cholesterol esters in medium HDL (mmol/l)	755	0.00	-0.07	0.08	0.958	755	0.01	-0.07	0.09	0.879	755	0.01	-0.07	0.09	0.853
Free cholesterol in medium HDL (mmol/l)	755	-0.01	-0.07	0.06	0.808	755	-0.01	-0.08	0.06	0.860	755	-0.01	-0.08	0.06	0.851
Triglycerides in medium HDL (mmol/l)	755	0.01	-0.07	0.08	0.827	755	0.00	-0.08	0.08	0.934	755	0.00	-0.08	0.08	0.960

S11 Table Associations of change in sedentary time (SED change from age 12y-15y) with metabolic traits at age 15y in ALSPAC

Change in SED from age 12y-15y (per SD-unit increase)

	Adj. for age, sex, ethnicity, maternal education change in wear time, wear month					*Additionally adj. for change in MVPA*					*Additionally adj. for change in FMI*				
**Standardised outcome at age 15y**	**N**	**Beta**	**LCL**	**UCL**	**P-value**	**N**	**Beta**	**LCL**	**UCL**	**P-value**	**N**	**Beta**	**LCL**	**UCL**	**P-value**
Concentration of small HDL particles (mol/l)	755	-0.01	-0.08	0.06	0.835	755	-0.01	-0.08	0.06	0.757	755	-0.01	-0.08	0.06	0.703
Total lipids in small HDL (mmol/l)	755	-0.01	-0.07	0.06	0.853	755	-0.01	-0.07	0.06	0.887	755	-0.01	-0.08	0.06	0.835
Phospholipids in small HDL (mmol/l)	755	-0.01	-0.08	0.06	0.826	755	-0.02	-0.09	0.06	0.668	755	-0.02	-0.09	0.05	0.648
Total cholesterol in small HDL (mmol/l)	755	0.00	-0.07	0.06	0.898	755	0.01	-0.06	0.08	0.850	755	0.00	-0.07	0.07	0.902
Cholesterol esters in small HDL (mmol/l)	755	-0.01	-0.07	0.06	0.827	755	0.01	-0.06	0.08	0.857	755	0.00	-0.07	0.07	0.915
Free cholesterol in small HDL (mmol/l)	755	0.01	-0.06	0.08	0.801	755	0.01	-0.07	0.08	0.886	755	0.01	-0.07	0.08	0.890
Triglycerides in small HDL (mmol/l)	755	0.01	-0.07	0.08	0.858	755	0.00	-0.08	0.08	0.984	755	0.00	-0.08	0.07	0.922
Phospholipids to total lipds ratio in chylomicrons and extremely large VLDL (%)	755	0.01	-0.06	0.08	0.728	755	0.00	-0.07	0.07	0.954	755	0.00	-0.07	0.06	0.895
Total cholesterol to total lipids ratio in chylomicrons and extremely large VLDL (%)	755	0.00	-0.07	0.08	0.915	755	0.01	-0.07	0.09	0.801	755	0.00	-0.07	0.08	0.921
Cholesterol esters to total lipids ratio in chylomicrons and extremely large VLDL (%)	755	0.01	-0.07	0.08	0.835	755	0.02	-0.06	0.09	0.668	755	0.01	-0.07	0.09	0.777
Free cholesterol to total lipids ratio in chylomicrons and extremely large VLDL (%)	755	-0.01	-0.09	0.07	0.830	755	-0.01	-0.09	0.07	0.726	755	-0.02	-0.10	0.06	0.651
Triglycerides to total lipids ratio in chylomicrons and extremely large VLDL (%)	755	0.00	-0.05	0.05	0.953	755	0.00	-0.06	0.05	0.868	755	0.00	-0.05	0.05	0.982
Phospholipids to total lipds ratio in very large VLDL (%)	755	0.00	-0.07	0.08	0.899	755	0.01	-0.08	0.09	0.897	755	0.00	-0.08	0.08	0.971
Total cholesterol to total lipids ratio in very large VLDL (%)	755	-0.01	-0.10	0.08	0.836	755	0.02	-0.06	0.09	0.657	755	0.02	-0.05	0.09	0.570
Cholesterol esters to total lipids ratio in very large VLDL (%)	755	0.02	-0.06	0.09	0.628	755	0.02	-0.05	0.10	0.528	755	0.03	-0.05	0.10	0.472
Free cholesterol to total lipids ratio in very large VLDL (%)	755	0.02	-0.06	0.10	0.649	755	0.03	-0.06	0.11	0.560	755	0.03	-0.06	0.12	0.551
Triglycerides to total lipids ratio in very large VLDL (%)	755	-0.02	-0.10	0.06	0.603	755	-0.03	-0.11	0.05	0.482	755	-0.03	-0.11	0.05	0.480
Phospholipids to total lipds ratio in large VLDL (%)	755	-0.01	-0.09	0.07	0.851	755	-0.01	-0.09	0.07	0.813	755	-0.02	-0.10	0.07	0.710
Total cholesterol to total lipids ratio in large VLDL (%)	755	0.04	-0.04	0.11	0.356	755	0.05	-0.03	0.12	0.260	755	0.04	-0.04	0.12	0.313
Cholesterol esters to total lipids ratio in large VLDL (%)	755	-0.03	-0.15	0.09	0.675	755	0.02	-0.04	0.07	0.506	755	0.02	-0.03	0.07	0.404
Free cholesterol to total lipids ratio in large VLDL (%)	755	0.00	-0.07	0.08	0.913	755	0.00	-0.08	0.08	0.979	755	-0.01	-0.09	0.08	0.890
Triglycerides to total lipids ratio in large VLDL (%)	755	-0.07	-0.20	0.06	0.298	755	-0.02	-0.07	0.03	0.418	755	-0.01	-0.06	0.03	0.518
Phospholipids to total lipds ratio in medium VLDL (%)	755	0.05	-0.05	0.15	0.289	755	0.07	-0.04	0.17	0.244	755	0.07	-0.04	0.17	0.237
Total cholesterol to total lipids ratio in medium VLDL (%)	755	0.06	-0.02	0.13	0.132	755	0.06	-0.02	0.14	0.127	755	0.06	-0.02	0.13	0.162
Cholesterol esters to total lipids ratio in medium VLDL (%)	755	0.05	-0.02	0.13	0.153	755	0.06	-0.02	0.13	0.139	755	0.05	-0.02	0.13	0.170
Free cholesterol to total lipids ratio in medium VLDL (%)	755	0.04	-0.04	0.11	0.326	755	0.04	-0.04	0.11	0.352	755	0.03	-0.05	0.11	0.428
Triglycerides to total lipids ratio in medium VLDL (%)	755	-0.06	-0.14	0.02	0.116	755	-0.07	-0.15	0.01	0.102	755	-0.06	-0.14	0.02	0.124
Phospholipids to total lipds ratio in small VLDL (%)	755	-0.01	-0.09	0.06	0.681	755	-0.01	-0.08	0.07	0.894	755	0.00	-0.07	0.08	0.990
Total cholesterol to total lipids ratio in small VLDL (%)	755	0.03	-0.04	0.11	0.377	755	0.03	-0.05	0.11	0.420	755	0.03	-0.05	0.11	0.449
Cholesterol esters to total lipids ratio in small VLDL (%)	755	0.02	-0.05	0.09	0.540	755	0.02	-0.05	0.09	0.608	755	0.02	-0.06	0.09	0.646
Free cholesterol to total lipids ratio in small VLDL (%)	755	0.08	-0.02	0.18	0.137	755	0.09	-0.02	0.19	0.113	755	0.09	-0.02	0.19	0.110
Triglycerides to total lipids ratio in small VLDL (%)	755	-0.03	-0.10	0.05	0.456	755	-0.03	-0.11	0.05	0.460	755	-0.03	-0.11	0.05	0.464
Phospholipids to total lipds ratio in very small VLDL (%)	755	0.01	-0.06	0.08	0.768	755	0.01	-0.06	0.08	0.722	755	0.01	-0.06	0.08	0.766
Total cholesterol to total lipids ratio in very small VLDL (%)	755	0.00	-0.07	0.07	0.999	755	-0.01	-0.08	0.07	0.888	755	0.00	-0.08	0.07	0.908
Cholesterol esters to total lipids ratio in very small VLDL (%)	755	-0.02	-0.09	0.05	0.646	755	-0.02	-0.10	0.05	0.515	755	-0.02	-0.10	0.05	0.529
Free cholesterol to total lipids ratio in very small VLDL (%)	755	0.04	-0.04	0.11	0.335	755	0.04	-0.04	0.12	0.337	755	0.04	-0.04	0.12	0.334
Triglycerides to total lipids ratio in very small VLDL (%)	755	-0.01	-0.08	0.06	0.829	755	0.00	-0.07	0.07	0.938	755	0.00	-0.07	0.07	0.947
Phospholipids to total lipds ratio in IDL (%)	755	-0.09	-0.19	0.01	0.090	755	-0.08	-0.20	0.03	0.135	755	-0.08	-0.19	0.03	0.156
Total cholesterol to total lipids ratio in IDL (%)	755	0.05	-0.02	0.13	0.175	755	0.04	-0.04	0.12	0.277	755	0.04	-0.04	0.12	0.329
Cholesterol esters to total lipids ratio in IDL (%)	755	0.06	-0.03	0.14	0.178	755	0.05	-0.04	0.14	0.288	755	0.04	-0.05	0.13	0.346
Free cholesterol to total lipids ratio in IDL (%)	755	-0.01	-0.08	0.06	0.762	755	-0.01	-0.08	0.06	0.806	755	-0.01	-0.08	0.07	0.862
Triglycerides to total lipids ratio in IDL (%)	755	-0.02	-0.09	0.05	0.540	755	-0.01	-0.08	0.06	0.719	755	-0.01	-0.08	0.06	0.781
Phospholipids to total lipds ratio in large LDL (%)	755	-0.03	-0.09	0.03	0.360	755	-0.02	-0.08	0.04	0.495	755	-0.02	-0.08	0.04	0.568
Total cholesterol to total lipids ratio in large LDL (%)	755	0.04	-0.02	0.10	0.213	755	0.03	-0.03	0.09	0.341	755	0.03	-0.04	0.09	0.419
Cholesterol esters to total lipids ratio in large LDL (%)	755	0.04	-0.02	0.10	0.237	755	0.03	-0.04	0.09	0.385	755	0.02	-0.04	0.09	0.492
Free cholesterol to total lipids ratio in large LDL (%)	755	-0.01	-0.08	0.05	0.705	755	-0.01	-0.07	0.06	0.846	755	0.00	-0.07	0.07	0.988
Triglycerides to total lipids ratio in large LDL (%)	755	-0.03	-0.10	0.04	0.350	755	-0.03	-0.10	0.05	0.469	755	-0.02	-0.09	0.05	0.535
Phospholipids to total lipds ratio in medium LDL (%)	755	-0.01	-0.03	0.02	0.686	755	0.00	-0.03	0.02	0.880	755	0.00	-0.03	0.02	0.950

S11 Table Associations of change in sedentary time (SED change from age 12y-15y) with metabolic traits at age 15y in ALSPAC

Change in SED from age 12y-15y (per SD-unit increase)

	Adj. for age, sex, ethnicity, maternal education change in wear time, wear month					*Additionally adj. for change in MVPA*					*Additionally adj. for change in FMI*				
**Standardised outcome at age 15y**	**N**	**Beta**	**LCL**	**UCL**	**P-value**	**N**	**Beta**	**LCL**	**UCL**	**P-value**	**N**	**Beta**	**LCL**	**UCL**	**P-value**
Total cholesterol to total lipids ratio in medium LDL (%)	755	0.03	-0.03	0.10	0.330	755	0.02	-0.05	0.09	0.558	755	0.02	-0.05	0.08	0.643
Cholesterol esters to total lipids ratio in medium LDL (%)	755	0.02	-0.05	0.09	0.567	755	0.01	-0.06	0.08	0.809	755	0.00	-0.07	0.07	0.912
Free cholesterol to total lipids ratio in medium LDL (%)	755	0.00	-0.02	0.02	0.984	755	0.00	-0.02	0.02	0.829	755	0.00	-0.02	0.02	0.738
Triglycerides to total lipids ratio in medium LDL (%)	755	-0.05	-0.12	0.03	0.242	755	-0.03	-0.11	0.04	0.382	755	-0.03	-0.11	0.05	0.422
Phospholipids to total lipds ratio in small LDL (%)	755	-0.01	-0.05	0.03	0.599	755	-0.01	-0.05	0.03	0.765	755	0.00	-0.04	0.04	0.852
Total cholesterol to total lipids ratio in small LDL (%)	755	0.03	-0.04	0.09	0.453	755	0.02	-0.05	0.08	0.634	755	0.01	-0.05	0.08	0.708
Cholesterol esters to total lipids ratio in small LDL (%)	755	0.01	-0.05	0.08	0.693	755	0.01	-0.06	0.08	0.863	755	0.00	-0.07	0.07	0.950
Free cholesterol to total lipids ratio in small LDL (%)	755	0.00	-0.03	0.04	0.824	755	0.01	-0.03	0.05	0.745	755	0.01	-0.03	0.05	0.669
Triglycerides to total lipids ratio in small LDL (%)	755	-0.03	-0.10	0.04	0.450	755	-0.02	-0.09	0.05	0.578	755	-0.02	-0.09	0.05	0.570
Phospholipids to total lipds ratio in very large HDL (%)	755	-0.03	-0.10	0.04	0.404	755	-0.02	-0.09	0.05	0.638	755	-0.01	-0.08	0.06	0.751
Total cholesterol to total lipids ratio in very large HDL (%)	755	0.03	-0.04	0.09	0.458	755	0.01	-0.06	0.09	0.694	755	0.01	-0.06	0.08	0.799
Cholesterol esters to total lipids ratio in very large HDL (%)	755	0.02	-0.04	0.09	0.481	755	0.01	-0.06	0.08	0.710	755	0.01	-0.06	0.08	0.814
Free cholesterol to total lipids ratio in very large HDL (%)	755	0.00	-0.08	0.08	0.923	755	0.00	-0.08	0.09	0.953	755	0.00	-0.08	0.09	0.931
Triglycerides to total lipids ratio in very large HDL (%)	755	0.03	-0.05	0.10	0.498	755	0.02	-0.06	0.10	0.628	755	0.02	-0.07	0.10	0.713
Phospholipids to total lipds ratio in large HDL (%)	755	0.00	-0.07	0.06	0.911	755	-0.01	-0.07	0.06	0.876	755	-0.01	-0.08	0.05	0.733
Total cholesterol to total lipids ratio in large HDL (%)	755	-0.01	-0.08	0.06	0.882	755	0.00	-0.07	0.07	0.980	755	0.01	-0.07	0.08	0.872
Cholesterol esters to total lipids ratio in large HDL (%)	755	-0.01	-0.08	0.06	0.797	755	-0.01	-0.08	0.07	0.880	755	0.00	-0.07	0.07	0.966
Free cholesterol to total lipids ratio in large HDL (%)	755	0.01	-0.06	0.08	0.770	755	0.02	-0.05	0.09	0.657	755	0.02	-0.05	0.09	0.574
Triglycerides to total lipids ratio in large HDL (%)	755	0.02	-0.06	0.10	0.603	755	0.01	-0.07	0.10	0.756	755	0.01	-0.07	0.09	0.868
Phospholipids to total lipds ratio in medium HDL (%)	755	-0.03	-0.10	0.05	0.493	755	-0.03	-0.11	0.05	0.511	755	-0.03	-0.11	0.05	0.489
Total cholesterol to total lipids ratio in medium HDL (%)	755	0.02	-0.06	0.10	0.608	755	0.02	-0.06	0.10	0.584	755	0.03	-0.05	0.11	0.512
Cholesterol esters to total lipids ratio in medium HDL (%)	755	0.03	-0.06	0.12	0.528	755	0.03	-0.06	0.13	0.500	755	0.04	-0.06	0.14	0.439
Free cholesterol to total lipids ratio in medium HDL (%)	755	-0.05	-0.21	0.12	0.581	755	-0.05	-0.23	0.13	0.573	755	-0.05	-0.22	0.12	0.567
Triglycerides to total lipids ratio in medium HDL (%)	755	0.00	-0.08	0.08	0.961	755	0.00	-0.09	0.08	0.945	755	-0.01	-0.09	0.07	0.839
Phospholipids to total lipds ratio in small HDL (%)	755	0.00	-0.07	0.07	0.957	755	-0.02	-0.09	0.06	0.681	755	-0.01	-0.09	0.06	0.732
Total cholesterol to total lipids ratio in small HDL (%)	755	0.00	-0.07	0.07	0.945	755	0.02	-0.06	0.09	0.685	755	0.01	-0.06	0.09	0.712
Cholesterol esters to total lipids ratio in small HDL (%)	755	-0.01	-0.07	0.06	0.816	755	0.01	-0.06	0.08	0.774	755	0.01	-0.06	0.08	0.817
Free cholesterol to total lipids ratio in small HDL (%)	755	0.04	-0.04	0.12	0.297	755	0.03	-0.05	0.11	0.488	755	0.03	-0.05	0.11	0.413
Triglycerides to total lipids ratio in small HDL (%)	755	0.00	-0.07	0.08	0.926	755	0.00	-0.08	0.07	0.933	755	-0.01	-0.09	0.07	0.846
Mean diameter for VLDL particles (nm)	755	0.01	-0.07	0.08	0.884	755	0.00	-0.08	0.07	0.926	755	-0.01	-0.09	0.07	0.821
Mean diameter for LDL particles (nm)	755	-0.01	-0.08	0.05	0.713	755	-0.01	-0.07	0.06	0.868	755	0.00	-0.07	0.07	0.966
Mean diameter for HDL particles (nm)	755	0.00	-0.08	0.07	0.943	755	0.00	-0.08	0.08	0.944	755	0.01	-0.07	0.08	0.876
Serum total cholesterol (mmol/l)	755	0.03	-0.04	0.10	0.350	755	0.03	-0.04	0.10	0.379	755	0.03	-0.05	0.10	0.470
Total cholesterol in VLDL (mmol/l)	755	0.05	-0.03	0.12	0.228	755	0.04	-0.04	0.12	0.347	755	0.03	-0.05	0.11	0.465
Remnant cholesterol (non-HDL, non-LDL -cholesterol) (mmol/l)	755	0.05	-0.02	0.12	0.187	755	0.04	-0.03	0.12	0.266	755	0.03	-0.04	0.11	0.366
Total cholesterol in LDL (mmol/l)	755	0.03	-0.04	0.10	0.413	755	0.03	-0.04	0.10	0.439	755	0.02	-0.05	0.10	0.538
Total cholesterol in HDL (mmol/l)	755	0.00	-0.08	0.07	0.903	755	0.00	-0.08	0.08	0.972	755	0.00	-0.07	0.08	0.937
Total cholesterol in HDL2 (mmol/l)	755	-0.01	-0.09	0.06	0.752	755	-0.01	-0.08	0.07	0.899	755	0.00	-0.08	0.08	0.952
Total cholesterol in HDL3 (mmol/l)	755	0.01	-0.06	0.08	0.803	755	0.01	-0.06	0.09	0.733	755	0.01	-0.06	0.09	0.735
Esterified cholesterol (mmol/l)	755	0.03	-0.04	0.10	0.386	755	0.03	-0.04	0.10	0.418	755	0.02	-0.05	0.09	0.515
Free cholesterol (mmol/l)	755	0.04	-0.03	0.11	0.302	755	0.04	-0.04	0.11	0.325	755	0.03	-0.04	0.11	0.400
Serum total triglycerides (mmol/l)	755	0.03	-0.05	0.10	0.480	755	0.02	-0.06	0.09	0.652	755	0.01	-0.07	0.09	0.776
Triglycerides in VLDL (mmol/l)	755	0.03	-0.05	0.10	0.485	755	0.02	-0.06	0.09	0.686	755	0.01	-0.07	0.09	0.814
Triglycerides in LDL (mmol/l)	755	0.01	-0.07	0.09	0.818	755	0.01	-0.07	0.09	0.809	755	0.01	-0.07	0.09	0.849
Triglycerides in HDL (mmol/l)	755	0.01	-0.06	0.09	0.732	755	0.01	-0.07	0.09	0.861	755	0.00	-0.08	0.08	0.958
Diacylglycerol (mmol/l)	755	-0.02	-0.09	0.05	0.576	755	-0.02	-0.09	0.06	0.663	755	-0.02	-0.09	0.05	0.557
Ratio of diacylglycerol to triglycerides	755	-0.04	-0.11	0.02	0.212	755	-0.03	-0.10	0.04	0.364	755	-0.03	-0.11	0.04	0.331
Total phosphoglycerides (mmol/l)	755	0.01	-0.06	0.09	0.708	755	0.02	-0.06	0.10	0.669	755	0.01	-0.07	0.09	0.733

S11 Table Associations of change in sedentary time (SED change from age 12y-15y) with metabolic traits at age 15y in ALSPAC

Change in SED from age 12y-15y (per SD-unit increase)

	Adj. for age, sex, ethnicity, maternal education change in wear time, wear month					*Additionally adj. for change in MVPA*					*Additionally adj. for change in FMI*				
**Standardised outcome at age 15y**	**N**	**Beta**	**LCL**	**UCL**	**P-value**	**N**	**Beta**	**LCL**	**UCL**	**P-value**	**N**	**Beta**	**LCL**	**UCL**	**P-value**
Ratio of triglycerides to phosphoglycerides	755	0.03	-0.04	0.10	0.353	755	0.03	-0.05	0.10	0.463	755	0.02	-0.05	0.09	0.558
Phosphatidylcholine and other cholines (mmol/l)	755	0.03	-0.04	0.10	0.398	755	0.03	-0.05	0.10	0.478	755	0.02	-0.05	0.10	0.525
Total cholines (mmol/l)	755	0.01	-0.06	0.08	0.800	755	0.01	-0.06	0.09	0.732	755	0.01	-0.07	0.09	0.786
Apolipoprotein A-I (g/l)	755	0.01	-0.06	0.08	0.808	755	0.01	-0.06	0.09	0.749	755	0.01	-0.06	0.09	0.769
Apolipoprotein B (g/l)	755	0.05	-0.03	0.12	0.208	755	0.04	-0.04	0.12	0.292	755	0.03	-0.04	0.11	0.394
Ratio of apolipoprotein B to apolipoprotein A-I	755	0.04	-0.03	0.12	0.258	755	0.04	-0.04	0.12	0.359	755	0.03	-0.05	0.11	0.466
Total fatty acids (mmol/l)	755	0.03	-0.04	0.11	0.395	755	0.03	-0.05	0.11	0.430	755	0.03	-0.05	0.10	0.525
Estimated description of fatty acid chain length, not actual carbon number	755	-0.02	-0.08	0.04	0.515	755	-0.02	-0.09	0.04	0.468	755	-0.02	-0.09	0.04	0.460
Estimated degree of unsaturation	755	0.01	-0.07	0.09	0.837	755	0.01	-0.07	0.09	0.795	755	0.01	-0.07	0.09	0.752
22:6, docosahexaenoic acid (mmol/l)	755	-0.05	-0.12	0.01	0.118	755	-0.05	-0.12	0.02	0.146	755	-0.05	-0.12	0.01	0.114
18:2, linoleic acid (mmol/l)	755	0.05	-0.02	0.12	0.180	755	0.05	-0.02	0.13	0.158	755	0.05	-0.03	0.12	0.207
Conjugated linoleic acid (mmol/l)	755	-0.01	-0.06	0.05	0.861	755	-0.01	-0.07	0.05	0.768	755	-0.01	-0.07	0.05	0.712
Omega-3 fatty acids (mmol/l)	755	-0.02	-0.09	0.04	0.481	755	-0.03	-0.10	0.04	0.456	755	-0.03	-0.10	0.04	0.371
Omega-6 fatty acids (mmol/l)	755	0.04	-0.03	0.11	0.275	755	0.04	-0.03	0.12	0.251	755	0.04	-0.04	0.11	0.315
Polyunsaturated fatty acids (mmol/l)	755	0.03	-0.04	0.10	0.365	755	0.04	-0.04	0.11	0.339	755	0.03	-0.04	0.10	0.421
Monounsaturated fatty acids; 16:1, 18:1 (mmol/l)	755	0.05	-0.03	0.12	0.224	755	0.04	-0.04	0.12	0.296	755	0.04	-0.04	0.11	0.362
Saturated fatty acids (mmol/l)	755	0.01	-0.07	0.10	0.776	755	0.01	-0.08	0.10	0.801	755	0.01	-0.08	0.09	0.899
Ratio of 22:6 docosahexaenoic acid to total fatty acids (%)	755	-0.08	-0.15	-0.01	0.029	755	-0.08	-0.15	-0.01	0.033	755	-0.08	-0.15	-0.01	0.029
Ratio of 18:2 linoleic acid to total fatty acids (%)	755	0.03	-0.04	0.11	0.396	755	0.04	-0.03	0.12	0.278	755	0.04	-0.03	0.12	0.265
Ratio of conjugated linoleic acid to total fatty acids (%)	755	-0.01	-0.07	0.05	0.791	755	-0.01	-0.07	0.05	0.784	755	-0.01	-0.07	0.05	0.741
Ratio of omega-3 fatty acids to total fatty acids (%)	755	-0.04	-0.11	0.03	0.290	755	-0.04	-0.12	0.03	0.243	755	-0.05	-0.12	0.03	0.221
Ratio of omega-6 fatty acids to total fatty acids (%)	755	0.02	-0.06	0.09	0.620	755	0.03	-0.05	0.11	0.476	755	0.03	-0.05	0.11	0.435
Ratio of polyunsaturated fatty acids to total fatty acids (%)	755	0.01	-0.07	0.09	0.807	755	0.02	-0.06	0.10	0.668	755	0.02	-0.06	0.10	0.632
Ratio of monounsaturated fatty acids to total fatty acids (%)	755	0.03	-0.05	0.10	0.510	755	0.02	-0.06	0.10	0.666	755	0.02	-0.06	0.10	0.701
Ratio of saturated fatty acids to total fatty acids (%)	755	-0.04	-0.13	0.04	0.297	755	-0.04	-0.13	0.05	0.341	755	-0.04	-0.13	0.05	0.347
Insulin (mu/l)	755	0.00	-0.05	0.04	0.927	755	0.01	-0.04	0.05	0.819	755	0.00	-0.04	0.04	0.998
Glucose (mmol/l)	755	0.04	-0.03	0.11	0.237	755	0.04	-0.03	0.12	0.260	755	0.04	-0.03	0.12	0.284
Lactate (mmol/l)	755	0.04	-0.03	0.11	0.305	755	0.04	-0.04	0.11	0.298	755	0.04	-0.04	0.11	0.314
Pyruvate (mmol/l)	755	0.05	-0.02	0.12	0.161	755	0.06	-0.01	0.13	0.108	755	0.06	-0.01	0.13	0.115
Citrate (mmol/l)	755	0.02	-0.06	0.10	0.576	755	0.02	-0.06	0.11	0.571	755	0.03	-0.06	0.11	0.532
Alanine (mmol/l)	755	0.06	-0.01	0.13	0.102	755	0.08	0.01	0.16	0.034	755	0.08	0.00	0.16	0.038
Glutamine (mmol/l)	755	-0.05	-0.11	0.01	0.129	755	-0.05	-0.12	0.02	0.144	755	-0.05	-0.11	0.02	0.160
Histidine (mmol/l)	755	0.01	-0.06	0.08	0.794	755	0.01	-0.06	0.09	0.718	755	0.01	-0.06	0.08	0.770
Isoleucine (mmol/l)	755	0.01	-0.05	0.08	0.670	755	0.01	-0.05	0.08	0.692	755	0.01	-0.06	0.08	0.778
Leucine (mmol/l)	755	-0.01	-0.06	0.05	0.792	755	0.00	-0.05	0.06	0.909	755	0.00	-0.06	0.06	0.962
Valine (mmol/l)	755	0.01	-0.05	0.08	0.705	755	0.02	-0.05	0.09	0.556	755	0.02	-0.05	0.08	0.607
Phenylalanine (mmol/l)	755	-0.07	-0.14	0.00	0.048	755	-0.07	-0.14	0.00	0.066	755	-0.07	-0.14	0.00	0.064
Tyrosine (mmol/l)	755	-0.08	-0.15	-0.01	0.029	755	-0.09	-0.16	-0.01	0.021	755	-0.09	-0.17	-0.02	0.014
Acetate (mmol/l)	755	0.03	-0.04	0.10	0.439	755	0.04	-0.04	0.11	0.320	755	0.04	-0.04	0.11	0.315
Acetoacetate (mmol/l)	755	0.00	-0.07	0.07	0.988	755	0.01	-0.07	0.09	0.787	755	0.01	-0.06	0.09	0.710
3-hydroxybutyrate (mmol/l)	755	-0.02	-0.10	0.06	0.575	755	-0.02	-0.10	0.06	0.704	755	-0.01	-0.09	0.07	0.735
Creatinine (mmol/l)	755	0.04	-0.03	0.11	0.280	755	0.04	-0.03	0.12	0.240	755	0.05	-0.02	0.12	0.193
Albumin (signal area)	755	0.01	-0.06	0.09	0.743	755	0.01	-0.07	0.09	0.771	755	0.01	-0.06	0.09	0.720
Glycoprotein acetyls, mainly a1-acid glycoprotein (mmol/l)	755	-0.04	-0.11	0.03	0.235	755	-0.05	-0.12	0.03	0.215	755	-0.05	-0.13	0.02	0.166
C-reactive protein (mg/l)	755	-0.01	-0.05	0.03	0.639	755	-0.01	-0.05	0.04	0.692	755	-0.01	-0.06	0.03	0.659
