# Supplementary material for: Associations of device-measured physical activity across adolescence with metabolic traits: Prospective cohort study
Source: PLoS Med. 2018 Sep 11;15(9):e1002649. doi: 10.1371/journal.pmed.1002649 (PMC6133272; doi:10.1371/journal.pmed.1002649)
Supplement: S12 Table — ALSPAC, Avon Longitudinal Study of Parents and Children. (PDF) [file pmed.1002649.s012.pdf]

S12 Table Associations of current physical activity (measures at age 15y) with metabolic traits at age 15y, with adjustment for metabolic traits at age 8y in ALSPAC

	**CPM at age 15y (per SD higher)** *Adj. for age, sex, ethnicity, maternal education, wear time, wear month, FMI, metabolic trait at 8y*					**MVPA at age 15y (per SD higher)** *Adj. for age, sex, ethnicity, maternal education, wear time, wear month, SED, FMI, metabolic trait at 8y*					**SED at age 15y (per SD higher)** *Adj. for age, sex, ethnicity, maternal education, wear time, wear month, MVPA, FMI, metabolic trait at 8y*				
**Standardised outcome at age 15y**	**N**	**Beta**	**LCL**	**UCL**	**P-value**	**N**	**Beta**	**LCL**	**UCL**	**P-value**	**N**	**Beta**	**LCL**	**UCL**	**P-value**
Systolic blood pressure (mmHg)	1603	-0.03	-0.08	0.02	0.227	1603	-0.04	-0.09	0.01	0.122	1603	0.01	-0.05	0.06	0.857
Diastolic blood pressure (mmHg)	1602	-0.02	-0.07	0.04	0.541	1602	0.02	-0.03	0.08	0.460	1602	0.05	-0.01	0.10	0.114
Concentration of chylomicrons and extremely large VLDL particles (mol/l)	839	-0.12	-0.19	-0.06	8.79E-05	839	-0.12	-0.18	-0.06	2.02E-04	839	0.04	-0.04	0.11	0.331
Total lipids in chylomicrons and extremely large VLDL (mmol/l)	839	-0.12	-0.19	-0.06	1.16E-04	839	-0.11	-0.18	-0.05	3.52E-04	839	0.04	-0.03	0.12	0.279
Phospholipids in chylomicrons and extremely large VLDL (mmol/l)	839	-0.13	-0.19	-0.06	9.41E-05	839	-0.12	-0.18	-0.06	2.43E-04	839	0.04	-0.04	0.11	0.338
Total cholesterol in chylomicrons and extremely large VLDL (mmol/l)	839	-0.10	-0.16	-0.04	6.38E-04	839	-0.10	-0.16	-0.04	1.21E-03	839	0.04	-0.03	0.11	0.271
Cholesterol esters in chylomicrons and extremely large VLDL (mmol/l)	839	-0.08	-0.14	-0.02	0.006	839	-0.08	-0.13	-0.02	0.007	839	0.04	-0.03	0.11	0.279
Free cholesterol in chylomicrons and extremely large VLDL (mmol/l)	839	-0.12	-0.19	-0.06	1.24E-04	839	-0.12	-0.18	-0.05	3.26E-04	839	0.04	-0.04	0.11	0.330
Triglycerides in chylomicrons and extremely large VLDL (mmol/l)	839	-0.13	-0.19	-0.06	8.98E-05	839	-0.12	-0.18	-0.05	3.05E-04	839	0.04	-0.03	0.12	0.278
Concentration of very large VLDL particles (mol/l)	839	-0.12	-0.18	-0.05	2.63E-04	839	-0.10	-0.17	-0.04	1.49E-03	839	0.05	-0.02	0.13	0.175
Total lipids in very large VLDL (mmol/l)	839	-0.12	-0.18	-0.05	3.41E-04	839	-0.10	-0.16	-0.04	1.83E-03	839	0.05	-0.02	0.13	0.176
Phospholipids in very large VLDL (mmol/l)	839	-0.12	-0.18	-0.05	2.53E-04	839	-0.11	-0.17	-0.04	8.29E-04	839	0.04	-0.03	0.12	0.263
Total cholesterol in very large VLDL (mmol/l)	839	-0.11	-0.18	-0.05	2.41E-04	839	-0.11	-0.17	-0.05	6.35E-04	839	0.04	-0.03	0.12	0.249
Cholesterol esters in very large VLDL (mmol/l)	839	-0.11	-0.17	-0.05	4.39E-04	839	-0.10	-0.16	-0.04	1.32E-03	839	0.05	-0.02	0.12	0.196
Free cholesterol in very large VLDL (mmol/l)	839	-0.12	-0.18	-0.06	1.58E-04	839	-0.11	-0.17	-0.05	3.24E-04	839	0.04	-0.04	0.11	0.333
Triglycerides in very large VLDL (mmol/l)	839	-0.12	-0.18	-0.05	4.43E-04	839	-0.10	-0.16	-0.03	0.003	839	0.06	-0.02	0.13	0.143
Concentration of large VLDL particles (mol/l)	839	-0.11	-0.17	-0.04	1.32E-03	839	-0.09	-0.15	-0.02	0.009	839	0.06	-0.02	0.14	0.121
Total lipids in large VLDL (mmol/l)	839	-0.11	-0.17	-0.04	1.34E-03	839	-0.09	-0.15	-0.02	0.008	839	0.06	-0.02	0.14	0.131
Phospholipids in large VLDL (mmol/l)	839	-0.11	-0.17	-0.04	1.29E-03	839	-0.09	-0.16	-0.03	0.006	839	0.05	-0.02	0.13	0.160
Total cholesterol in large VLDL (mmol/l)	839	-0.10	-0.16	-0.04	1.99E-03	839	-0.09	-0.15	-0.03	0.006	839	0.05	-0.02	0.13	0.175
Cholesterol esters in large VLDL (mmol/l)	839	-0.09	-0.15	-0.03	0.005	839	-0.08	-0.14	-0.02	0.009	839	0.05	-0.03	0.12	0.203
Free cholesterol in large VLDL (mmol/l)	839	-0.11	-0.17	-0.04	1.06E-03	839	-0.09	-0.16	-0.03	0.005	839	0.05	-0.02	0.13	0.167
Triglycerides in large VLDL (mmol/l)	839	-0.11	-0.17	-0.04	1.24E-03	839	-0.09	-0.15	-0.02	0.009	839	0.06	-0.01	0.14	0.111
Concentration of medium VLDL particles (mol/l)	839	-0.10	-0.17	-0.04	1.48E-03	839	-0.09	-0.15	-0.03	0.006	839	0.05	-0.02	0.13	0.159
Total lipids in medium VLDL (mmol/l)	839	-0.10	-0.16	-0.04	2.02E-03	839	-0.09	-0.15	-0.02	0.007	839	0.05	-0.02	0.13	0.178
Phospholipids in medium VLDL (mmol/l)	839	-0.10	-0.16	-0.04	1.88E-03	839	-0.09	-0.15	-0.03	0.005	839	0.05	-0.03	0.12	0.213
Total cholesterol in medium VLDL (mmol/l)	839	-0.08	-0.14	-0.02	0.011	839	-0.08	-0.14	-0.02	0.009	839	0.03	-0.04	0.11	0.337
Cholesterol esters in medium VLDL (mmol/l)	839	-0.06	-0.11	0.00	0.060	839	-0.07	-0.12	-0.01	0.020	839	0.02	-0.05	0.09	0.562
Free cholesterol in medium VLDL (mmol/l)	839	-0.10	-0.16	-0.04	2.21E-03	839	-0.09	-0.15	-0.03	0.006	839	0.05	-0.03	0.12	0.210
Triglycerides in medium VLDL (mmol/l)	839	-0.11	-0.17	-0.04	1.25E-03	839	-0.09	-0.15	-0.02	0.008	839	0.06	-0.02	0.14	0.134
Concentration of small VLDL particles (mol/l)	839	-0.08	-0.14	-0.02	0.008	839	-0.09	-0.15	-0.03	0.004	839	0.02	-0.05	0.09	0.578
Total lipids in small VLDL (mmol/l)	839	-0.08	-0.13	-0.02	0.011	839	-0.09	-0.15	-0.03	2.15E-03	839	0.00	-0.06	0.07	0.921
Phospholipids in small VLDL (mmol/l)	839	-0.05	-0.11	0.00	0.068	839	-0.08	-0.14	-0.02	0.009	839	-0.01	-0.07	0.06	0.797
Total cholesterol in small VLDL (mmol/l)	839	-0.05	-0.10	0.00	0.061	839	-0.09	-0.14	-0.04	7.17E-04	839	-0.04	-0.10	0.02	0.202
Cholesterol esters in small VLDL (mmol/l)	839	-0.04	-0.09	0.01	0.088	839	-0.09	-0.14	-0.04	3.22E-04	839	-0.05	-0.11	0.01	0.073
Free cholesterol in small VLDL (mmol/l)	839	-0.06	-0.11	0.00	0.056	839	-0.08	-0.14	-0.02	0.007	839	-0.01	-0.07	0.06	0.853
Triglycerides in small VLDL (mmol/l)	839	-0.09	-0.16	-0.03	0.006	839	-0.09	-0.15	-0.02	0.009	839	0.04	-0.04	0.11	0.327
Concentration of very small VLDL particles (mol/l)	839	0.00	-0.05	0.05	0.887	839	-0.05	-0.10	0.00	0.042	839	-0.05	-0.11	0.01	0.084
Total lipids in very small VLDL (mmol/l)	839	-0.01	-0.06	0.04	0.608	839	-0.06	-0.11	-0.02	0.007	839	-0.06	-0.12	0.00	0.042
Phospholipids in very small VLDL (mmol/l)	839	-0.01	-0.05	0.04	0.843	839	-0.06	-0.11	-0.01	0.025	839	-0.06	-0.12	-0.01	0.026
Total cholesterol in very small VLDL (mmol/l)	839	0.00	-0.05	0.05	0.981	839	-0.05	-0.11	0.00	0.041	839	-0.06	-0.12	0.01	0.084
Cholesterol esters in very small VLDL (mmol/l)	839	-0.01	-0.07	0.04	0.641	839	-0.06	-0.12	-0.01	0.012	839	-0.06	-0.12	0.00	0.069
Free cholesterol in very small VLDL (mmol/l)	839	0.02	-0.03	0.08	0.445	839	-0.03	-0.08	0.03	0.393	839	-0.04	-0.11	0.03	0.290
Triglycerides in very small VLDL (mmol/l)	839	-0.06	-0.12	0.00	0.058	839	-0.08	-0.14	-0.02	0.012	839	0.00	-0.07	0.06	0.890
Concentration of IDL particles (mol/l)	839	-0.02	-0.07	0.04	0.547	839	-0.05	-0.11	0.00	0.053	839	-0.05	-0.11	0.01	0.124
Total lipids in IDL (mmol/l)	839	0.00	-0.06	0.05	0.860	839	-0.05	-0.10	0.00	0.063	839	-0.06	-0.12	0.00	0.044
Phospholipids in IDL (mmol/l)	839	-0.01	-0.07	0.05	0.778	839	-0.05	-0.11	0.01	0.086	839	-0.06	-0.12	0.00	0.065

Total cholesterol in IDL (mmol/l)	839	0.00	-0.05	0.05	0.992	839	-0.05	-0.10	0.00	0.063	839	-0.06	-0.12	0.00	0.034
Cholesterol esters in IDL (mmol/l)	839	0.00	-0.05	0.05	0.905	839	-0.05	-0.10	0.00	0.036	839	-0.06	-0.12	0.00	0.034
Free cholesterol in IDL (mmol/l)	839	0.01	-0.05	0.06	0.818	839	-0.04	-0.09	0.02	0.186	839	-0.06	-0.12	0.00	0.050
Triglycerides in IDL (mmol/l)	839	-0.01	-0.07	0.04	0.639	839	-0.04	-0.10	0.01	0.141	839	-0.04	-0.10	0.03	0.256
Concentration of large LDL particles (mol/l)	839	-0.01	-0.07	0.04	0.623	839	-0.06	-0.12	0.00	0.053	839	-0.06	-0.12	0.00	0.067
Total lipids in large LDL (mmol/l)	839	-0.01	-0.06	0.05	0.802	839	-0.05	-0.11	0.00	0.066	839	-0.06	-0.12	0.00	0.041
Phospholipids in large LDL (mmol/l)	839	-0.01	-0.06	0.05	0.828	839	-0.06	-0.11	0.00	0.052	839	-0.07	-0.13	-0.01	0.026
Total cholesterol in large LDL (mmol/l)	839	0.00	-0.06	0.05	0.888	839	-0.05	-0.11	0.00	0.074	839	-0.06	-0.12	0.00	0.037
Cholesterol esters in large LDL (mmol/l)	839	-0.01	-0.06	0.05	0.793	839	-0.05	-0.11	0.00	0.059	839	-0.06	-0.12	0.00	0.041
Free cholesterol in large LDL (mmol/l)	839	0.01	-0.05	0.06	0.834	839	-0.04	-0.10	0.01	0.145	839	-0.07	-0.13	-0.01	0.028
Triglycerides in large LDL (mmol/l)	839	-0.01	-0.08	0.05	0.643	839	-0.04	-0.10	0.02	0.158	839	-0.04	-0.11	0.02	0.206
Concentration of medium LDL particles (mol/l)	839	-0.03	-0.09	0.03	0.391	839	-0.07	-0.13	-0.01	0.027	839	-0.06	-0.12	0.01	0.092
Total lipids in medium LDL (mmol/l)	839	-0.02	-0.07	0.04	0.606	839	-0.06	-0.12	0.00	0.040	839	-0.06	-0.12	0.00	0.051
Phospholipids in medium LDL (mmol/l)	839	-0.01	-0.06	0.04	0.729	839	-0.06	-0.11	-0.01	0.027	839	-0.07	-0.12	-0.01	0.018
Total cholesterol in medium LDL (mmol/l)	839	-0.01	-0.07	0.04	0.639	839	-0.06	-0.12	0.00	0.045	839	-0.06	-0.12	0.00	0.057
Cholesterol esters in medium LDL (mmol/l)	839	-0.02	-0.08	0.04	0.531	839	-0.06	-0.12	0.00	0.038	839	-0.06	-0.12	0.01	0.074
Free cholesterol in medium LDL (mmol/l)	839	0.01	-0.05	0.06	0.791	839	-0.04	-0.10	0.01	0.107	839	-0.07	-0.13	-0.01	0.016
Triglycerides in medium LDL (mmol/l)	839	-0.01	-0.07	0.06	0.791	839	-0.05	-0.11	0.02	0.138	839	-0.06	-0.14	0.01	0.078
Concentration of small LDL particles (mol/l)	839	-0.03	-0.09	0.03	0.365	839	-0.07	-0.13	-0.01	0.028	839	-0.05	-0.12	0.01	0.125
Total lipids in small LDL (mmol/l)	839	-0.02	-0.07	0.04	0.554	839	-0.06	-0.12	0.00	0.036	839	-0.06	-0.12	0.00	0.064
Phospholipids in small LDL (mmol/l)	839	-0.01	-0.07	0.04	0.662	839	-0.06	-0.11	0.00	0.042	839	-0.06	-0.11	0.00	0.060
Total cholesterol in small LDL (mmol/l)	839	-0.01	-0.07	0.04	0.657	839	-0.06	-0.12	0.00	0.046	839	-0.06	-0.12	0.00	0.053
Cholesterol esters in small LDL (mmol/l)	839	-0.02	-0.08	0.04	0.502	839	-0.06	-0.12	0.00	0.036	839	-0.06	-0.12	0.01	0.077
Free cholesterol in small LDL (mmol/l)	839	0.02	-0.03	0.07	0.492	839	-0.04	-0.09	0.01	0.135	839	-0.08	-0.13	-0.02	0.008
Triglycerides in small LDL (mmol/l)	839	-0.04	-0.11	0.02	0.191	839	-0.08	-0.14	-0.01	0.018	839	-0.04	-0.12	0.03	0.233
Concentration of very large HDL particles (mol/l)	839	0.04	-0.03	0.10	0.235	839	0.05	-0.02	0.11	0.173	839	0.01	-0.05	0.07	0.748
Total lipids in very large HDL (mmol/l)	839	0.03	-0.03	0.10	0.295	839	0.04	-0.02	0.11	0.203	839	0.01	-0.05	0.08	0.696
Phospholipids in very large HDL (mmol/l)	839	0.04	-0.02	0.11	0.152	839	0.05	-0.01	0.12	0.124	839	0.01	-0.06	0.07	0.853
Total cholesterol in very large HDL (mmol/l)	839	0.03	-0.04	0.09	0.462	839	0.04	-0.03	0.11	0.322	839	0.02	-0.05	0.08	0.658
Cholesterol esters in very large HDL (mmol/l)	839	0.02	-0.05	0.09	0.551	839	0.03	-0.04	0.10	0.410	839	0.01	-0.06	0.09	0.676
Free cholesterol in very large HDL (mmol/l)	839	0.04	-0.03	0.10	0.285	839	0.05	-0.02	0.11	0.168	839	0.02	-0.05	0.08	0.629
Triglycerides in very large HDL (mmol/l)	839	-0.04	-0.10	0.03	0.249	839	-0.01	-0.08	0.05	0.681	839	0.06	-0.01	0.14	0.115
Concentration of large HDL particles (mol/l)	839	0.07	0.01	0.13	0.025	839	0.07	0.00	0.13	0.042	839	-0.01	-0.07	0.05	0.768
Total lipids in large HDL (mmol/l)	839	0.07	0.01	0.13	0.022	839	0.07	0.00	0.13	0.040	839	-0.01	-0.07	0.05	0.741
Phospholipids in large HDL (mmol/l)	839	0.06	0.00	0.12	0.038	839	0.06	-0.01	0.12	0.081	839	-0.02	-0.08	0.04	0.574
Total cholesterol in large HDL (mmol/l)	839	0.07	0.02	0.13	0.013	839	0.07	0.01	0.14	0.020	839	-0.01	-0.07	0.06	0.862
Cholesterol esters in large HDL (mmol/l)	839	0.07	0.02	0.13	0.013	839	0.08	0.01	0.14	0.018	839	0.00	-0.06	0.06	0.899
Free cholesterol in large HDL (mmol/l)	839	0.07	0.02	0.13	0.013	839	0.07	0.01	0.13	0.030	839	-0.01	-0.07	0.05	0.729
Triglycerides in large HDL (mmol/l)	839	0.01	-0.05	0.07	0.781	839	0.01	-0.05	0.07	0.717	839	0.02	-0.05	0.09	0.535
Concentration of medium HDL particles (mol/l)	839	0.05	-0.01	0.12	0.100	839	0.02	-0.04	0.09	0.468	839	-0.05	-0.12	0.01	0.127
Total lipids in medium HDL (mmol/l)	839	0.06	-0.01	0.12	0.089	839	0.03	-0.04	0.09	0.443	839	-0.06	-0.12	0.01	0.101
Phospholipids in medium HDL (mmol/l)	839	0.06	-0.01	0.12	0.079	839	0.02	-0.04	0.09	0.462	839	-0.06	-0.12	0.01	0.089
Total cholesterol in medium HDL (mmol/l)	839	0.06	0.00	0.13	0.058	839	0.04	-0.03	0.11	0.242	839	-0.05	-0.12	0.02	0.152
Cholesterol esters in medium HDL (mmol/l)	839	0.06	0.00	0.13	0.052	839	0.04	-0.02	0.11	0.186	839	-0.05	-0.11	0.02	0.188
Free cholesterol in medium HDL (mmol/l)	839	0.05	-0.01	0.12	0.105	839	0.02	-0.05	0.08	0.651	839	-0.06	-0.13	0.00	0.064
Triglycerides in medium HDL (mmol/l)	839	-0.05	-0.11	0.01	0.102	839	-0.07	-0.13	-0.01	0.025	839	-0.02	-0.08	0.05	0.626
Concentration of small HDL particles (mol/l)	839	-0.01	-0.07	0.06	0.849	839	-0.04	-0.10	0.03	0.230	839	-0.06	-0.13	0.01	0.079
Total lipids in small HDL (mmol/l)	839	0.03	-0.03	0.09	0.361	839	-0.02	-0.08	0.04	0.550	839	-0.08	-0.15	-0.02	0.013
Phospholipids in small HDL (mmol/l)	839	-0.02	-0.09	0.04	0.463	839	-0.03	-0.09	0.03	0.349	839	-0.03	-0.10	0.05	0.469
Total cholesterol in small HDL (mmol/l)	839	0.09	0.03	0.15	0.006	839	0.01	-0.05	0.08	0.657	839	-0.12	-0.18	-0.05	4.56E-04
Cholesterol esters in small HDL (mmol/l)	839	0.09	0.03	0.16	0.003	839	0.01	-0.05	0.08	0.707	839	-0.12	-0.19	-0.06	1.98E-04
Free cholesterol in small HDL (mmol/l)	839	0.03	-0.03	0.10	0.277	839	0.02	-0.04	0.08	0.518	839	-0.03	-0.11	0.04	0.365
Triglycerides in small HDL (mmol/l)	839	-0.07	-0.13	-0.01	0.031	839	-0.08	-0.14	-0.02	0.013	839	0.00	-0.07	0.07	0.989
Phospholipids to total lipds ratio in chylomicrons and extremely large VLDL (%)	839	-0.06	-0.12	0.01	0.098	839	-0.09	-0.16	-0.03	0.007	839	-0.04	-0.12	0.04	0.316
Total cholesterol to total lipids ratio in chylomicrons and extremely large VLDL (%)	839	-0.01	-0.08	0.06	0.718	839	-0.03	-0.10	0.03	0.327	839	-0.01	-0.09	0.07	0.760
Cholesterol esters to total lipids ratio in chylomicrons and extremely large VLDL (%)	839	0.01	-0.06	0.08	0.800	839	-0.02	-0.08	0.05	0.649	839	-0.01	-0.09	0.06	0.713
Free cholesterol to total lipids ratio in chylomicrons and extremely large VLDL (%)	839	-0.08	-0.15	-0.01	0.031	839	-0.08	-0.15	-0.01	0.020	839	0.01	-0.07	0.09	0.861

Triglycerides to total lipids ratio in chylomicrons and extremely large VLDL (%)	839	0.02	-0.03	0.07	0.446	839	0.04	-0.01	0.09	0.102	839	0.02	-0.04	0.07	0.545
Phospholipids to total lipds ratio in very large VLDL (%)	839	-0.09	-0.15	-0.02	0.012	839	-0.11	-0.18	-0.04	1.21E-03	839	-0.02	-0.10	0.06	0.595
Total cholesterol to total lipids ratio in very large VLDL (%)	839	0.06	-0.01	0.12	0.104	839	0.02	-0.05	0.09	0.493	839	-0.05	-0.12	0.03	0.212
Cholesterol esters to total lipids ratio in very large VLDL (%)	839	0.07	0.00	0.15	0.052	839	0.05	-0.03	0.13	0.207	839	-0.04	-0.12	0.04	0.326
Free cholesterol to total lipids ratio in very large VLDL (%)	839	0.03	-0.04	0.11	0.381	839	-0.01	-0.09	0.07	0.796	839	-0.06	-0.15	0.02	0.156
Triglycerides to total lipids ratio in very large VLDL (%)	839	-0.03	-0.11	0.04	0.372	839	0.01	-0.07	0.09	0.760	839	0.06	-0.02	0.15	0.153
Phospholipids to total lipds ratio in large VLDL (%)	839	-0.06	-0.14	0.01	0.102	839	-0.06	-0.14	0.01	0.109	839	0.02	-0.07	0.10	0.706
Total cholesterol to total lipids ratio in large VLDL (%)	839	-0.04	-0.11	0.02	0.191	839	-0.05	-0.12	0.02	0.125	839	0.01	-0.06	0.09	0.726
Cholesterol esters to total lipids ratio in large VLDL (%)	839	0.00	-0.03	0.03	0.916	839	-0.01	-0.04	0.02	0.431	839	0.00	-0.04	0.03	0.826
Free cholesterol to total lipids ratio in large VLDL (%)	839	-0.08	-0.15	-0.01	0.030	839	-0.08	-0.16	0.00	0.038	839	0.02	-0.07	0.10	0.698
Triglycerides to total lipids ratio in large VLDL (%)	839	0.01	0.00	0.02	0.141	839	0.01	0.00	0.02	0.141	839	0.00	-0.01	0.01	0.558
Phospholipids to total lipds ratio in medium VLDL (%)	839	0.03	-0.04	0.10	0.358	839	0.02	-0.06	0.10	0.594	839	-0.02	-0.10	0.06	0.604
Total cholesterol to total lipids ratio in medium VLDL (%)	839	0.03	-0.03	0.08	0.382	839	0.00	-0.05	0.06	0.876	839	-0.01	-0.08	0.06	0.791
Cholesterol esters to total lipids ratio in medium VLDL (%)	839	0.04	-0.02	0.10	0.197	839	0.02	-0.05	0.08	0.644	839	-0.01	-0.09	0.06	0.735
Free cholesterol to total lipids ratio in medium VLDL (%)	839	-0.05	-0.11	0.02	0.143	839	-0.04	-0.11	0.02	0.205	839	0.01	-0.06	0.09	0.692
Triglycerides to total lipids ratio in medium VLDL (%)	839	-0.03	-0.09	0.03	0.295	839	-0.01	-0.07	0.05	0.762	839	0.01	-0.06	0.08	0.697
Phospholipids to total lipds ratio in small VLDL (%)	839	0.13	0.06	0.20	2.36E-04	839	0.11	0.04	0.18	1.42E-03	839	-0.05	-0.13	0.03	0.213
Total cholesterol to total lipids ratio in small VLDL (%)	839	0.03	-0.04	0.10	0.385	839	-0.02	-0.09	0.05	0.578	839	-0.06	-0.14	0.01	0.107
Cholesterol esters to total lipids ratio in small VLDL (%)	839	0.01	-0.05	0.08	0.732	839	-0.04	-0.10	0.03	0.290	839	-0.06	-0.14	0.02	0.122
Free cholesterol to total lipids ratio in small VLDL (%)	839	0.13	0.06	0.20	2.94E-04	839	0.10	0.02	0.17	0.011	839	-0.04	-0.12	0.04	0.296
Triglycerides to total lipids ratio in small VLDL (%)	839	-0.06	-0.14	0.01	0.085	839	-0.01	-0.09	0.06	0.736	839	0.07	-0.01	0.15	0.071
Phospholipids to total lipds ratio in very small VLDL (%)	839	0.00	-0.06	0.07	0.894	839	-0.04	-0.10	0.03	0.268	839	-0.05	-0.11	0.02	0.155
Total cholesterol to total lipids ratio in very small VLDL (%)	839	0.04	-0.03	0.10	0.267	839	0.05	-0.02	0.11	0.159	839	0.01	-0.06	0.08	0.803
Cholesterol esters to total lipids ratio in very small VLDL (%)	839	0.00	-0.06	0.07	0.888	839	0.01	-0.05	0.07	0.779	839	-0.01	-0.08	0.06	0.799
Free cholesterol to total lipids ratio in very small VLDL (%)	839	0.10	0.03	0.16	0.003	839	0.12	0.05	0.19	1.01E-03	839	0.05	-0.03	0.13	0.197
Triglycerides to total lipids ratio in very small VLDL (%)	839	-0.05	-0.11	0.02	0.157	839	-0.03	-0.09	0.04	0.428	839	0.03	-0.05	0.10	0.504
Phospholipids to total lipds ratio in IDL (%)	839	0.03	-0.03	0.10	0.333	839	0.03	-0.04	0.09	0.469	839	-0.07	-0.15	0.02	0.111
Total cholesterol to total lipids ratio in IDL (%)	839	-0.01	-0.07	0.05	0.675	839	-0.03	-0.09	0.03	0.371	839	0.01	-0.06	0.08	0.787
Cholesterol esters to total lipids ratio in IDL (%)	839	-0.04	-0.11	0.02	0.191	839	-0.04	-0.11	0.02	0.194	839	0.03	-0.04	0.11	0.415
Free cholesterol to total lipids ratio in IDL (%)	839	0.06	-0.01	0.12	0.087	839	0.03	-0.04	0.10	0.372	839	-0.04	-0.11	0.03	0.290
Triglycerides to total lipids ratio in IDL (%)	839	0.00	-0.06	0.07	0.931	839	0.02	-0.04	0.08	0.492	839	0.01	-0.06	0.08	0.721
Phospholipids to total lipds ratio in large LDL (%)	839	0.01	-0.05	0.06	0.791	839	0.04	-0.02	0.09	0.177	839	0.01	-0.04	0.07	0.621
Total cholesterol to total lipids ratio in large LDL (%)	839	0.01	-0.05	0.07	0.738	839	-0.04	-0.10	0.02	0.184	839	-0.04	-0.10	0.02	0.176
Cholesterol esters to total lipids ratio in large LDL (%)	839	0.00	-0.06	0.05	0.910	839	-0.05	-0.11	0.00	0.060	839	-0.04	-0.09	0.01	0.132
Free cholesterol to total lipids ratio in large LDL (%)	839	0.06	0.00	0.12	0.036	839	0.08	0.03	0.14	0.005	839	0.00	-0.07	0.06	0.948
Triglycerides to total lipids ratio in large LDL (%)	839	0.00	-0.07	0.06	0.982	839	0.03	-0.03	0.09	0.374	839	0.03	-0.05	0.10	0.502
Phospholipids to total lipds ratio in medium LDL (%)	839	0.00	-0.02	0.03	0.716	839	0.02	0.00	0.04	0.104	839	0.01	-0.01	0.03	0.473
Total cholesterol to total lipids ratio in medium LDL (%)	839	-0.01	-0.07	0.06	0.808	839	-0.05	-0.12	0.01	0.098	839	-0.03	-0.09	0.03	0.385
Cholesterol esters to total lipids ratio in medium LDL (%)	839	-0.02	-0.08	0.05	0.591	839	-0.07	-0.13	0.00	0.047	839	-0.03	-0.09	0.03	0.332
Free cholesterol to total lipids ratio in medium LDL (%)	839	0.01	-0.01	0.03	0.322	839	0.02	0.00	0.04	0.023	839	0.01	-0.01	0.03	0.411
Triglycerides to total lipids ratio in medium LDL (%)	839	0.02	-0.05	0.08	0.642	839	0.02	-0.05	0.08	0.621	839	-0.01	-0.09	0.06	0.734
Phospholipids to total lipds ratio in small LDL (%)	839	0.01	-0.03	0.05	0.648	839	0.04	0.00	0.07	0.055	839	0.02	-0.02	0.06	0.262
Total cholesterol to total lipids ratio in small LDL (%)	839	0.00	-0.06	0.07	0.972	839	-0.05	-0.12	0.01	0.121	839	-0.04	-0.11	0.02	0.195
Cholesterol esters to total lipids ratio in small LDL (%)	839	-0.02	-0.08	0.05	0.644	839	-0.07	-0.13	0.00	0.057	839	-0.04	-0.10	0.03	0.256
Free cholesterol to total lipids ratio in small LDL (%)	839	0.02	-0.01	0.06	0.215	839	0.04	0.01	0.08	0.021	839	0.01	-0.02	0.05	0.488
Triglycerides to total lipids ratio in small LDL (%)	839	-0.03	-0.10	0.04	0.376	839	-0.03	-0.09	0.03	0.360	839	0.00	-0.07	0.07	0.962
Phospholipids to total lipds ratio in very large HDL (%)	839	0.07	0.01	0.13	0.032	839	0.07	0.00	0.13	0.061	839	-0.02	-0.08	0.05	0.604
Total cholesterol to total lipids ratio in very large HDL (%)	839	-0.05	-0.11	0.01	0.082	839	-0.05	-0.12	0.01	0.125	839	0.01	-0.05	0.07	0.784
Cholesterol esters to total lipids ratio in very large HDL (%)	839	-0.05	-0.11	0.01	0.097	839	-0.05	-0.12	0.01	0.116	839	0.00	-0.06	0.07	0.933
Free cholesterol to total lipids ratio in very large HDL (%)	839	0.01	-0.06	0.08	0.801	839	0.04	-0.03	0.11	0.315	839	0.05	-0.02	0.12	0.193
Triglycerides to total lipids ratio in very large HDL (%)	839	-0.09	-0.15	-0.02	0.015	839	-0.07	-0.14	-0.01	0.020	839	0.05	-0.02	0.12	0.166
Phospholipids to total lipds ratio in large HDL (%)	839	-0.08	-0.14	-0.01	0.019	839	-0.12	-0.18	-0.05	3.29E-04	839	-0.06	-0.13	0.01	0.081
Total cholesterol to total lipids ratio in large HDL (%)	839	0.09	0.02	0.15	0.007	839	0.11	0.05	0.18	3.40E-04	839	0.03	-0.03	0.10	0.344
Cholesterol esters to total lipids ratio in large HDL (%)	839	0.08	0.02	0.15	0.009	839	0.12	0.06	0.18	1.73E-04	839	0.04	-0.03	0.10	0.256
Free cholesterol to total lipids ratio in large HDL (%)	839	0.07	0.01	0.14	0.026	839	0.07	0.01	0.14	0.025	839	0.01	-0.06	0.07	0.865
Triglycerides to total lipids ratio in large HDL (%)	839	-0.09	-0.15	-0.02	0.009	839	-0.09	-0.15	-0.03	0.005	839	0.03	-0.04	0.10	0.432
Phospholipids to total lipds ratio in medium HDL (%)	839	0.06	-0.01	0.13	0.113	839	0.02	-0.05	0.09	0.591	839	-0.05	-0.13	0.03	0.228

Total cholesterol to total lipids ratio in medium HDL (%)	839	0.01	-0.06	0.07	0.849	839	0.04	-0.03	0.11	0.215	839	0.04	-0.04	0.11	0.328
Cholesterol esters to total lipids ratio in medium HDL (%)	839	0.01	-0.06	0.08	0.748	839	0.05	-0.01	0.12	0.111	839	0.04	-0.03	0.12	0.249
Free cholesterol to total lipids ratio in medium HDL (%)	839	-0.01	-0.08	0.06	0.711	839	-0.04	-0.12	0.04	0.302	839	-0.02	-0.11	0.06	0.559
Triglycerides to total lipids ratio in medium HDL (%)	839	-0.09	-0.15	-0.02	0.008	839	-0.10	-0.16	-0.03	0.004	839	0.00	-0.07	0.07	0.953
Phospholipids to total lipds ratio in small HDL (%)	839	-0.10	-0.16	-0.04	6.83E-04	839	-0.03	-0.09	0.04	0.402	839	0.11	0.04	0.17	2.14E-03
Total cholesterol to total lipids ratio in small HDL (%)	839	0.12	0.06	0.18	1.74E-04	839	0.05	-0.02	0.11	0.162	839	-0.10	-0.17	-0.03	0.003
Cholesterol esters to total lipids ratio in small HDL (%)	839	0.11	0.05	0.17	5.70E-04	839	0.03	-0.03	0.10	0.360	839	-0.11	-0.18	-0.04	1.41E-03
Free cholesterol to total lipids ratio in small HDL (%)	839	0.02	-0.05	0.08	0.617	839	0.10	0.03	0.16	0.004	839	0.12	0.04	0.19	2.19E-03
Triglycerides to total lipids ratio in small HDL (%)	839	-0.09	-0.16	-0.03	0.005	839	-0.09	-0.15	-0.02	0.011	839	0.03	-0.04	0.11	0.360
Mean diameter for VLDL particles (nm)	839	-0.11	-0.18	-0.04	2.42E-03	839	-0.08	-0.16	-0.01	0.025	839	0.06	-0.02	0.14	0.171
Mean diameter for LDL particles (nm)	839	0.04	-0.02	0.10	0.189	839	0.06	0.00	0.12	0.046	839	0.02	-0.06	0.09	0.671
Mean diameter for HDL particles (nm)	839	0.06	0.00	0.12	0.053	839	0.07	0.00	0.14	0.046	839	0.01	-0.05	0.07	0.685
Serum total cholesterol (mmol/l)	839	0.01	-0.04	0.06	0.743	839	-0.04	-0.09	0.02	0.193	839	-0.06	-0.11	0.00	0.038
Total cholesterol in VLDL (mmol/l)	839	-0.07	-0.12	-0.01	0.014	839	-0.09	-0.14	-0.04	8.62E-04	839	0.00	-0.07	0.06	0.918
Remnant cholesterol (non-HDL, non-LDL -cholesterol) (mmol/l)	839	-0.04	-0.08	0.01	0.134	839	-0.08	-0.12	-0.03	1.57E-03	839	-0.04	-0.09	0.02	0.199
Total cholesterol in LDL (mmol/l)	839	-0.01	-0.06	0.05	0.762	839	-0.05	-0.11	0.00	0.058	839	-0.06	-0.12	0.00	0.045
Total cholesterol in HDL (mmol/l)	839	0.07	0.01	0.14	0.015	839	0.06	-0.01	0.12	0.086	839	-0.03	-0.10	0.03	0.273
Total cholesterol in HDL2 (mmol/l)	839	0.08	0.02	0.14	0.008	839	0.06	0.00	0.13	0.055	839	-0.04	-0.10	0.02	0.222
Total cholesterol in HDL3 (mmol/l)	839	0.06	0.00	0.12	0.055	839	0.04	-0.02	0.10	0.197	839	-0.02	-0.09	0.04	0.425
Esterified cholesterol (mmol/l)	839	0.01	-0.04	0.06	0.737	839	-0.04	-0.09	0.02	0.178	839	-0.07	-0.12	-0.01	0.022
Free cholesterol (mmol/l)	838	0.01	-0.05	0.06	0.807	838	-0.03	-0.09	0.02	0.255	838	-0.04	-0.10	0.02	0.176
Serum total triglycerides (mmol/l)	839	-0.10	-0.16	-0.03	2.77E-03	839	-0.09	-0.16	-0.03	0.004	839	0.04	-0.04	0.11	0.353
Triglycerides in VLDL (mmol/l)	839	-0.11	-0.17	-0.04	1.36E-03	839	-0.09	-0.16	-0.03	0.006	839	0.05	-0.02	0.13	0.175
Triglycerides in LDL (mmol/l)	839	-0.02	-0.08	0.05	0.582	839	-0.05	-0.11	0.01	0.106	839	-0.05	-0.12	0.02	0.155
Triglycerides in HDL (mmol/l)	839	-0.06	-0.12	0.01	0.075	839	-0.06	-0.12	0.00	0.042	839	0.01	-0.06	0.08	0.714
Diacylglycerol (mmol/l)	791	0.02	-0.05	0.08	0.653	791	0.03	-0.04	0.11	0.365	791	0.04	-0.04	0.13	0.303
Ratio of diacylglycerol to triglycerides	791	0.05	-0.03	0.12	0.200	791	0.05	-0.03	0.14	0.211	791	0.00	-0.09	0.08	0.973
Total phosphoglycerides (mmol/l)	838	0.01	-0.05	0.07	0.734	838	-0.01	-0.07	0.05	0.797	838	-0.02	-0.09	0.05	0.535
Ratio of triglycerides to phosphoglycerides	838	-0.08	-0.15	-0.01	0.026	838	-0.06	-0.13	0.00	0.055	838	0.08	0.00	0.15	0.041
Phosphatidylcholine and other cholines (mmol/l)	826	0.02	-0.05	0.08	0.620	826	-0.01	-0.07	0.05	0.793	826	-0.01	-0.07	0.06	0.831
Total cholines (mmol/l)	836	0.02	-0.05	0.08	0.631	836	0.00	-0.07	0.06	0.909	836	-0.01	-0.07	0.05	0.763
Apolipoprotein A-I (g/l)	839	0.05	-0.01	0.11	0.096	839	0.02	-0.04	0.09	0.433	839	-0.04	-0.10	0.02	0.200
Apolipoprotein B (g/l)	839	-0.07	-0.12	-0.01	0.016	839	-0.09	-0.15	-0.04	8.09E-04	839	-0.02	-0.08	0.05	0.625
Ratio of apolipoprotein B to apolipoprotein A-I	839	-0.09	-0.15	-0.04	1.49E-03	839	-0.11	-0.17	-0.05	3.43E-04	839	0.00	-0.06	0.07	0.897
Total fatty acids (mmol/l)	839	-0.04	-0.10	0.02	0.212	839	-0.04	-0.10	0.02	0.193	839	0.01	-0.06	0.08	0.786
Estimated description of fatty acid chain length, not actual carbon number	835	-0.01	-0.08	0.06	0.754	835	-0.05	-0.12	0.03	0.213	835	-0.03	-0.11	0.05	0.459
Estimated degree of unsaturation	838	0.05	-0.03	0.12	0.253	838	-0.02	-0.10	0.06	0.609	838	-0.06	-0.14	0.02	0.160
22:6, docosahexaenoic acid (mmol/l)	839	0.01	-0.05	0.07	0.708	839	0.00	-0.07	0.06	0.908	839	-0.06	-0.13	0.01	0.098
18:2, linoleic acid (mmol/l)	836	-0.01	-0.07	0.04	0.630	836	-0.04	-0.10	0.01	0.139	836	-0.01	-0.07	0.06	0.792
Conjugated linoleic acid (mmol/l)	837	-0.02	-0.11	0.07	0.611	837	-0.03	-0.10	0.05	0.501	837	0.06	-0.03	0.14	0.186
Omega-3 fatty acids (mmol/l)	837	-0.04	-0.10	0.02	0.194	837	-0.02	-0.08	0.05	0.594	837	0.00	-0.07	0.06	0.889
Omega-6 fatty acids (mmol/l)	838	-0.01	-0.07	0.04	0.613	838	-0.04	-0.10	0.02	0.161	838	-0.01	-0.07	0.06	0.800
Polyunsaturated fatty acids (mmol/l)	836	-0.02	-0.08	0.04	0.544	836	-0.04	-0.10	0.02	0.215	836	-0.01	-0.07	0.06	0.834
Monounsaturated fatty acids; 16:1, 18:1 (mmol/l)	838	-0.05	-0.11	0.01	0.078	838	-0.07	-0.13	-0.01	0.020	838	0.00	-0.07	0.07	0.921
Saturated fatty acids (mmol/l)	837	-0.03	-0.09	0.04	0.393	837	-0.01	-0.09	0.07	0.778	837	0.03	-0.04	0.10	0.414
Ratio of 22:6 docosahexaenoic acid to total fatty acids (%)	839	0.04	-0.03	0.11	0.243	839	0.02	-0.05	0.10	0.507	839	-0.07	-0.15	0.01	0.075
Ratio of 18:2 linoleic acid to total fatty acids (%)	836	0.03	-0.03	0.10	0.351	836	0.00	-0.07	0.07	0.961	836	-0.01	-0.08	0.06	0.786
Ratio of conjugated linoleic acid to total fatty acids (%)	837	-0.01	-0.10	0.08	0.806	837	-0.02	-0.10	0.06	0.594	837	0.05	-0.03	0.14	0.220
Ratio of omega-3 fatty acids to total fatty acids (%)	837	-0.01	-0.07	0.06	0.795	837	0.02	-0.05	0.09	0.560	837	-0.01	-0.09	0.07	0.779
Ratio of omega-6 fatty acids to total fatty acids (%)	838	0.04	-0.03	0.10	0.304	838	0.01	-0.07	0.08	0.876	838	-0.01	-0.09	0.07	0.817
Ratio of polyunsaturated fatty acids to total fatty acids (%)	836	0.03	-0.04	0.10	0.357	836	0.01	-0.06	0.08	0.747	836	-0.01	-0.09	0.07	0.801
Ratio of monounsaturated fatty acids to total fatty acids (%)	838	-0.04	-0.12	0.03	0.219	838	-0.08	-0.15	0.00	0.052	838	-0.03	-0.11	0.05	0.400
Ratio of saturated fatty acids to total fatty acids (%)	837	0.01	-0.06	0.09	0.737	837	0.08	-0.01	0.17	0.065	837	0.05	-0.02	0.13	0.175
Glucose (mmol/l)	832	-0.06	-0.13	0.01	0.103	832	-0.04	-0.10	0.03	0.268	832	0.02	-0.06	0.10	0.621
Lactate (mmol/l)	838	-0.03	-0.10	0.04	0.418	838	0.01	-0.06	0.09	0.723	838	0.04	-0.04	0.12	0.305
Pyruvate (mmol/l)	836	-0.07	-0.15	0.02	0.118	836	0.00	-0.08	0.07	0.912	836	0.06	-0.02	0.14	0.155
Citrate (mmol/l)	835	0.10	0.02	0.18	0.011	835	0.03	-0.05	0.11	0.459	835	-0.10	-0.18	-0.02	0.018

Alanine (mmol/l)	839	-0.07	-0.15	0.01	0.079	839	0.04	-0.04	0.11	0.368	839	0.14	0.05	0.22	1.53E-03
Glutamine (mmol/l)	839	0.05	-0.02	0.12	0.131	839	0.07	0.00	0.14	0.056	839	-0.02	-0.10	0.05	0.516
Histidine (mmol/l)	789	0.03	-0.05	0.11	0.462	789	0.07	-0.02	0.15	0.126	789	0.07	-0.01	0.15	0.107
Isoleucine (mmol/l)	839	-0.06	-0.14	0.02	0.144	839	-0.01	-0.08	0.07	0.860	839	0.07	-0.01	0.15	0.100
Leucine (mmol/l)	839	0.03	-0.05	0.10	0.501	839	0.04	-0.03	0.11	0.239	839	0.01	-0.06	0.09	0.729
Valine (mmol/l)	839	-0.02	-0.09	0.05	0.605	839	0.01	-0.06	0.08	0.804	839	0.04	-0.05	0.12	0.387
Phenylalanine (mmol/l)	838	0.05	-0.03	0.13	0.199	838	0.05	-0.02	0.13	0.165	838	-0.03	-0.11	0.05	0.471
Tyrosine (mmol/l)	832	0.05	-0.03	0.14	0.229	832	0.02	-0.06	0.10	0.705	832	-0.07	-0.15	0.02	0.118
Acetate (mmol/l)	839	0.05	-0.02	0.12	0.193	839	0.06	-0.01	0.13	0.106	839	0.03	-0.06	0.11	0.563
Acetoacetate (mmol/l)	839	0.06	-0.04	0.16	0.224	839	0.02	-0.06	0.09	0.665	839	-0.08	-0.17	0.00	0.057
3-hydroxybutyrate (mmol/l)	838	-0.01	-0.08	0.05	0.675	838	-0.07	-0.13	-0.01	0.031	838	-0.09	-0.17	-0.01	0.022
Creatinine (mmol/l)	839	-0.09	-0.16	-0.03	0.005	839	-0.03	-0.10	0.04	0.392	839	0.10	0.02	0.18	0.011
Albumin (signal area)	839	-0.12	-0.19	-0.04	0.003	839	-0.07	-0.14	0.01	0.090	839	0.05	-0.04	0.13	0.315
Glycoprotein acetyls, mainly a1-acid glycoprotein (mmol/l)	839	-0.10	-0.16	-0.04	8.64E-04	839	-0.10	-0.16	-0.05	4.14E-04	839	0.01	-0.07	0.08	0.867

Complete case sample

	**CPM at age 15y (per SD higher)** *Adj. for age, sex, ethnicity, maternal education, wear time, wear month, FMI, metabolic trait at 8y*					**MVPA at age 15y (per SD higher)** *Adj. for age, sex, ethnicity, maternal education, wear time, wear month, SED, FMI, metabolic trait at 8y*					**SED at age 15y (per SD higher)** *Adj. for age, sex, ethnicity, maternal education, wear time, wear month, MVPA, FMI, metabolic trait at 8y*				
**Standardised outcome at age 15y**	**N**	**Beta**	**LCL**	**UCL**	**P-value**	**N**	**Beta**	**LCL**	**UCL**	**P-value**	**N**	**Beta**	**LCL**	**UCL**	**P-value**
Systolic blood pressure (mmHg)	520	0.00	-0.08	0.09	0.958	520	0.03	-0.06	0.12	0.539	520	0.02	-0.08	0.12	0.668
Diastolic blood pressure (mmHg)	520	0.05	-0.04	0.14	0.269	520	0.07	-0.01	0.16	0.095	520	0.03	-0.08	0.14	0.560
Concentration of chylomicrons and extremely large VLDL particles (mol/l)	520	-0.07	-0.15	0.00	0.059	520	-0.09	-0.17	-0.02	0.016	520	-0.03	-0.12	0.07	0.594
Total lipids in chylomicrons and extremely large VLDL (mmol/l)	520	-0.07	-0.15	0.00	0.054	520	-0.09	-0.17	-0.02	0.016	520	-0.02	-0.12	0.07	0.613
Phospholipids in chylomicrons and extremely large VLDL (mmol/l)	520	-0.08	-0.15	0.00	0.046	520	-0.10	-0.18	-0.02	0.012	520	-0.03	-0.12	0.07	0.561
Total cholesterol in chylomicrons and extremely large VLDL (mmol/l)	520	-0.05	-0.12	0.02	0.136	520	-0.07	-0.14	0.00	0.047	520	-0.02	-0.11	0.07	0.681
Cholesterol esters in chylomicrons and extremely large VLDL (mmol/l)	520	-0.03	-0.10	0.04	0.372	520	-0.05	-0.11	0.02	0.170	520	-0.01	-0.10	0.08	0.792
Free cholesterol in chylomicrons and extremely large VLDL (mmol/l)	520	-0.07	-0.15	0.00	0.056	520	-0.10	-0.17	-0.02	0.016	520	-0.03	-0.12	0.07	0.572
Triglycerides in chylomicrons and extremely large VLDL (mmol/l)	520	-0.08	-0.15	0.00	0.046	520	-0.10	-0.17	-0.02	0.014	520	-0.02	-0.12	0.07	0.605
Concentration of very large VLDL particles (mol/l)	520	-0.07	-0.14	0.01	0.074	520	-0.08	-0.16	0.00	0.037	520	-0.01	-0.10	0.08	0.795
Total lipids in very large VLDL (mmol/l)	520	-0.07	-0.14	0.01	0.076	520	-0.08	-0.16	0.00	0.038	520	-0.01	-0.10	0.08	0.788
Phospholipids in very large VLDL (mmol/l)	520	-0.07	-0.14	0.00	0.067	520	-0.09	-0.16	-0.01	0.026	520	-0.02	-0.11	0.07	0.695
Total cholesterol in very large VLDL (mmol/l)	520	-0.06	-0.13	0.01	0.103	520	-0.08	-0.15	-0.01	0.035	520	-0.02	-0.11	0.07	0.647
Cholesterol esters in very large VLDL (mmol/l)	520	-0.05	-0.12	0.02	0.155	520	-0.07	-0.14	0.00	0.061	520	-0.02	-0.11	0.07	0.694
Free cholesterol in very large VLDL (mmol/l)	520	-0.07	-0.14	0.01	0.071	520	-0.09	-0.16	-0.01	0.020	520	-0.03	-0.12	0.07	0.592
Triglycerides in very large VLDL (mmol/l)	520	-0.07	-0.14	0.01	0.074	520	-0.08	-0.16	0.00	0.045	520	-0.01	-0.10	0.08	0.857
Concentration of large VLDL particles (mol/l)	520	-0.06	-0.13	0.02	0.130	520	-0.07	-0.15	0.01	0.079	520	-0.01	-0.10	0.08	0.874
Total lipids in large VLDL (mmol/l)	520	-0.05	-0.13	0.02	0.143	520	-0.07	-0.14	0.01	0.082	520	-0.01	-0.10	0.08	0.840
Phospholipids in large VLDL (mmol/l)	520	-0.05	-0.13	0.02	0.144	520	-0.07	-0.15	0.01	0.071	520	-0.01	-0.10	0.08	0.778
Total cholesterol in large VLDL (mmol/l)	520	-0.04	-0.12	0.03	0.243	520	-0.06	-0.14	0.01	0.110	520	-0.02	-0.10	0.07	0.740
Cholesterol esters in large VLDL (mmol/l)	520	-0.03	-0.10	0.04	0.452	520	-0.05	-0.12	0.02	0.192	520	-0.02	-0.11	0.07	0.652
Free cholesterol in large VLDL (mmol/l)	520	-0.06	-0.13	0.02	0.125	520	-0.07	-0.15	0.00	0.063	520	-0.01	-0.10	0.08	0.814
Triglycerides in large VLDL (mmol/l)	520	-0.06	-0.13	0.01	0.116	520	-0.07	-0.15	0.01	0.077	520	-0.01	-0.09	0.08	0.896
Concentration of medium VLDL particles (mol/l)	520	-0.05	-0.12	0.03	0.199	520	-0.07	-0.14	0.01	0.100	520	-0.02	-0.11	0.07	0.723
Total lipids in medium VLDL (mmol/l)	520	-0.04	-0.12	0.03	0.265	520	-0.06	-0.14	0.02	0.120	520	-0.02	-0.11	0.07	0.668
Phospholipids in medium VLDL (mmol/l)	520	-0.04	-0.12	0.03	0.274	520	-0.06	-0.14	0.01	0.113	520	-0.02	-0.11	0.07	0.626
Total cholesterol in medium VLDL (mmol/l)	520	-0.01	-0.08	0.06	0.748	520	-0.04	-0.12	0.03	0.263	520	-0.03	-0.12	0.06	0.477
Cholesterol esters in medium VLDL (mmol/l)	520	0.01	-0.06	0.08	0.714	520	-0.02	-0.09	0.05	0.526	520	-0.04	-0.13	0.05	0.381
Free cholesterol in medium VLDL (mmol/l)	520	-0.04	-0.12	0.03	0.281	520	-0.06	-0.14	0.02	0.117	520	-0.02	-0.11	0.07	0.636
Triglycerides in medium VLDL (mmol/l)	520	-0.06	-0.13	0.02	0.145	520	-0.07	-0.15	0.01	0.089	520	-0.01	-0.10	0.08	0.780
Concentration of small VLDL particles (mol/l)	520	-0.02	-0.10	0.06	0.610	520	-0.06	-0.14	0.02	0.147	520	-0.05	-0.14	0.04	0.276
Total lipids in small VLDL (mmol/l)	520	-0.01	-0.09	0.06	0.753	520	-0.06	-0.14	0.02	0.129	520	-0.06	-0.15	0.02	0.139
Phospholipids in small VLDL (mmol/l)	520	0.01	-0.06	0.08	0.804	520	-0.05	-0.13	0.03	0.229	520	-0.07	-0.16	0.01	0.082

Total cholesterol in small VLDL (mmol/l)	520	0.01	-0.06	0.07	0.851	520	-0.06	-0.12	0.01	0.108	520	-0.09	-0.17	-0.01	0.021
Cholesterol esters in small VLDL (mmol/l)	520	0.00	-0.06	0.07	0.881	520	-0.06	-0.13	0.00	0.068	520	-0.10	-0.17	-0.02	0.011
Free cholesterol in small VLDL (mmol/l)	520	0.01	-0.06	0.08	0.820	520	-0.04	-0.12	0.03	0.254	520	-0.07	-0.15	0.02	0.114
Triglycerides in small VLDL (mmol/l)	520	-0.03	-0.11	0.05	0.420	520	-0.06	-0.15	0.02	0.141	520	-0.04	-0.13	0.06	0.446
Concentration of very small VLDL particles (mol/l)	520	0.04	-0.02	0.10	0.187	520	-0.02	-0.09	0.05	0.556	520	-0.08	-0.16	-0.01	0.032
Total lipids in very small VLDL (mmol/l)	520	0.03	-0.03	0.09	0.381	520	-0.04	-0.10	0.03	0.245	520	-0.09	-0.16	-0.02	0.015
Phospholipids in very small VLDL (mmol/l)	520	0.03	-0.03	0.09	0.379	520	-0.03	-0.10	0.04	0.412	520	-0.08	-0.15	-0.01	0.028
Total cholesterol in very small VLDL (mmol/l)	520	0.03	-0.04	0.10	0.382	520	-0.03	-0.10	0.04	0.407	520	-0.08	-0.16	0.00	0.050
Cholesterol esters in very small VLDL (mmol/l)	520	0.01	-0.05	0.08	0.673	520	-0.04	-0.11	0.02	0.177	520	-0.09	-0.17	-0.01	0.027
Free cholesterol in very small VLDL (mmol/l)	520	0.06	-0.01	0.13	0.113	520	0.01	-0.07	0.09	0.800	520	-0.05	-0.14	0.04	0.282
Triglycerides in very small VLDL (mmol/l)	520	0.00	-0.08	0.08	0.987	520	-0.05	-0.13	0.03	0.227	520	-0.06	-0.15	0.03	0.211
Concentration of IDL particles (mol/l)	520	0.01	-0.06	0.08	0.766	520	-0.03	-0.10	0.05	0.495	520	-0.06	-0.14	0.02	0.160
Total lipids in IDL (mmol/l)	520	0.02	-0.05	0.09	0.533	520	-0.02	-0.09	0.05	0.536	520	-0.07	-0.14	0.01	0.084
Phospholipids in IDL (mmol/l)	520	0.02	-0.06	0.09	0.679	520	-0.02	-0.10	0.05	0.543	520	-0.06	-0.15	0.02	0.131
Total cholesterol in IDL (mmol/l)	520	0.03	-0.04	0.09	0.442	520	-0.02	-0.09	0.05	0.545	520	-0.07	-0.15	0.00	0.066
Cholesterol esters in IDL (mmol/l)	520	0.03	-0.04	0.09	0.420	520	-0.02	-0.09	0.04	0.471	520	-0.08	-0.15	0.00	0.049
Free cholesterol in IDL (mmol/l)	520	0.02	-0.05	0.09	0.517	520	-0.01	-0.09	0.06	0.719	520	-0.06	-0.14	0.02	0.157
Triglycerides in IDL (mmol/l)	520	0.02	-0.06	0.09	0.676	520	-0.03	-0.11	0.05	0.473	520	-0.05	-0.14	0.03	0.243
Concentration of large LDL particles (mol/l)	520	0.01	-0.07	0.09	0.728	520	-0.03	-0.11	0.05	0.450	520	-0.07	-0.15	0.01	0.107
Total lipids in large LDL (mmol/l)	520	0.02	-0.05	0.09	0.594	520	-0.03	-0.10	0.05	0.490	520	-0.07	-0.15	0.01	0.076
Phospholipids in large LDL (mmol/l)	520	0.02	-0.05	0.10	0.536	520	-0.03	-0.11	0.05	0.459	520	-0.08	-0.16	0.00	0.050
Total cholesterol in large LDL (mmol/l)	520	0.02	-0.05	0.09	0.557	520	-0.03	-0.10	0.05	0.517	520	-0.07	-0.15	0.01	0.072
Cholesterol esters in large LDL (mmol/l)	520	0.02	-0.05	0.09	0.586	520	-0.03	-0.10	0.05	0.479	520	-0.07	-0.15	0.00	0.066
Free cholesterol in large LDL (mmol/l)	520	0.03	-0.05	0.10	0.479	520	-0.02	-0.10	0.06	0.634	520	-0.07	-0.15	0.01	0.093
Triglycerides in large LDL (mmol/l)	520	0.01	-0.08	0.09	0.842	520	-0.04	-0.12	0.05	0.377	520	-0.06	-0.15	0.03	0.215
Concentration of medium LDL particles (mol/l)	520	0.01	-0.08	0.09	0.866	520	-0.04	-0.13	0.04	0.343	520	-0.07	-0.16	0.02	0.105
Total lipids in medium LDL (mmol/l)	520	0.02	-0.06	0.09	0.699	520	-0.03	-0.11	0.04	0.398	520	-0.07	-0.16	0.01	0.073
Phospholipids in medium LDL (mmol/l)	520	0.03	-0.04	0.10	0.469	520	-0.04	-0.11	0.04	0.344	520	-0.09	-0.16	-0.01	0.020
Total cholesterol in medium LDL (mmol/l)	520	0.01	-0.06	0.09	0.711	520	-0.03	-0.11	0.05	0.427	520	-0.07	-0.15	0.01	0.084
Cholesterol esters in medium LDL (mmol/l)	520	0.01	-0.07	0.09	0.791	520	-0.03	-0.12	0.05	0.394	520	-0.07	-0.16	0.01	0.095
Free cholesterol in medium LDL (mmol/l)	520	0.03	-0.04	0.10	0.373	520	-0.02	-0.09	0.05	0.588	520	-0.08	-0.15	0.00	0.050
Triglycerides in medium LDL (mmol/l)	520	0.01	-0.08	0.10	0.770	520	-0.05	-0.14	0.04	0.287	520	-0.08	-0.18	0.01	0.092
Concentration of small LDL particles (mol/l)	520	0.00	-0.08	0.09	0.924	520	-0.04	-0.13	0.04	0.314	520	-0.07	-0.16	0.02	0.114
Total lipids in small LDL (mmol/l)	520	0.01	-0.07	0.09	0.753	520	-0.04	-0.12	0.04	0.366	520	-0.07	-0.16	0.01	0.074
Phospholipids in small LDL (mmol/l)	520	0.02	-0.05	0.09	0.619	520	-0.03	-0.11	0.04	0.362	520	-0.08	-0.15	0.00	0.048
Total cholesterol in small LDL (mmol/l)	520	0.01	-0.06	0.09	0.718	520	-0.03	-0.11	0.05	0.405	520	-0.07	-0.16	0.01	0.077
Cholesterol esters in small LDL (mmol/l)	520	0.01	-0.07	0.09	0.842	520	-0.04	-0.12	0.04	0.364	520	-0.07	-0.16	0.01	0.099
Free cholesterol in small LDL (mmol/l)	520	0.04	-0.03	0.11	0.213	520	-0.02	-0.09	0.05	0.607	520	-0.09	-0.16	-0.01	0.022
Triglycerides in small LDL (mmol/l)	520	0.00	-0.09	0.08	0.921	520	-0.07	-0.15	0.02	0.141	520	-0.08	-0.18	0.01	0.097
Concentration of very large HDL particles (mol/l)	520	0.02	-0.07	0.10	0.725	520	0.04	-0.06	0.13	0.464	520	0.04	-0.05	0.13	0.356
Total lipids in very large HDL (mmol/l)	520	0.01	-0.08	0.10	0.794	520	0.03	-0.07	0.13	0.526	520	0.04	-0.05	0.13	0.396
Phospholipids in very large HDL (mmol/l)	520	0.02	-0.06	0.10	0.615	520	0.04	-0.05	0.13	0.371	520	0.04	-0.04	0.12	0.324
Total cholesterol in very large HDL (mmol/l)	520	0.00	-0.09	0.09	0.963	520	0.02	-0.08	0.12	0.739	520	0.03	-0.06	0.12	0.541
Cholesterol esters in very large HDL (mmol/l)	520	0.00	-0.10	0.09	0.954	520	0.01	-0.09	0.11	0.853	520	0.03	-0.07	0.12	0.601
Free cholesterol in very large HDL (mmol/l)	520	0.01	-0.07	0.10	0.753	520	0.03	-0.06	0.13	0.483	520	0.04	-0.05	0.13	0.408
Triglycerides in very large HDL (mmol/l)	520	-0.01	-0.09	0.07	0.810	520	0.00	-0.08	0.09	0.958	520	0.04	-0.06	0.14	0.475
Concentration of large HDL particles (mol/l)	520	0.04	-0.04	0.12	0.307	520	0.06	-0.03	0.15	0.213	520	0.04	-0.05	0.12	0.399
Total lipids in large HDL (mmol/l)	520	0.04	-0.03	0.12	0.292	520	0.06	-0.03	0.15	0.207	520	0.03	-0.05	0.11	0.405
Phospholipids in large HDL (mmol/l)	520	0.04	-0.04	0.11	0.336	520	0.05	-0.04	0.14	0.285	520	0.02	-0.06	0.10	0.545
Total cholesterol in large HDL (mmol/l)	520	0.04	-0.03	0.12	0.262	520	0.06	-0.02	0.15	0.157	520	0.04	-0.04	0.12	0.316
Cholesterol esters in large HDL (mmol/l)	520	0.04	-0.03	0.12	0.265	520	0.06	-0.02	0.15	0.151	520	0.04	-0.04	0.12	0.300
Free cholesterol in large HDL (mmol/l)	520	0.04	-0.03	0.12	0.257	520	0.06	-0.03	0.15	0.185	520	0.04	-0.04	0.12	0.383
Triglycerides in large HDL (mmol/l)	520	0.03	-0.05	0.10	0.510	520	0.02	-0.06	0.11	0.555	520	0.01	-0.09	0.10	0.891
Concentration of medium HDL particles (mol/l)	520	0.06	-0.03	0.14	0.175	520	0.02	-0.06	0.11	0.597	520	-0.05	-0.13	0.04	0.296
Total lipids in medium HDL (mmol/l)	520	0.05	-0.03	0.14	0.211	520	0.02	-0.07	0.11	0.624	520	-0.04	-0.13	0.04	0.318
Phospholipids in medium HDL (mmol/l)	520	0.06	-0.02	0.14	0.166	520	0.02	-0.07	0.11	0.637	520	-0.05	-0.14	0.04	0.253
Total cholesterol in medium HDL (mmol/l)	520	0.04	-0.04	0.13	0.290	520	0.03	-0.06	0.12	0.494	520	-0.02	-0.11	0.06	0.631

Cholesterol esters in medium HDL (mmol/l)	520	0.04	-0.04	0.13	0.305	520	0.04	-0.05	0.12	0.437	520	-0.01	-0.10	0.07	0.751
Free cholesterol in medium HDL (mmol/l)	520	0.05	-0.03	0.13	0.243	520	0.01	-0.08	0.10	0.805	520	-0.05	-0.13	0.03	0.250
Triglycerides in medium HDL (mmol/l)	520	0.01	-0.07	0.08	0.899	520	-0.04	-0.13	0.04	0.332	520	-0.07	-0.16	0.01	0.095
Concentration of small HDL particles (mol/l)	520	0.01	-0.07	0.09	0.832	520	-0.04	-0.13	0.04	0.293	520	-0.09	-0.17	0.00	0.039
Total lipids in small HDL (mmol/l)	520	0.05	-0.03	0.13	0.260	520	-0.02	-0.10	0.06	0.652	520	-0.10	-0.18	-0.02	0.019
Phospholipids in small HDL (mmol/l)	520	-0.02	-0.09	0.06	0.711	520	-0.04	-0.12	0.04	0.355	520	-0.05	-0.14	0.03	0.241
Total cholesterol in small HDL (mmol/l)	520	0.10	0.02	0.18	0.010	520	0.02	-0.06	0.11	0.592	520	-0.10	-0.18	-0.02	0.012
Cholesterol esters in small HDL (mmol/l)	520	0.11	0.03	0.19	0.005	520	0.02	-0.06	0.11	0.579	520	-0.11	-0.19	-0.03	0.009
Free cholesterol in small HDL (mmol/l)	520	0.03	-0.05	0.12	0.414	520	0.01	-0.07	0.09	0.766	520	-0.04	-0.12	0.05	0.421
Triglycerides in small HDL (mmol/l)	520	-0.02	-0.10	0.06	0.544	520	-0.07	-0.15	0.02	0.110	520	-0.05	-0.15	0.04	0.244
Phospholipids to total lipds ratio in chylomicrons and extremely large VLDL (%)	520	-0.02	-0.11	0.07	0.614	520	-0.09	-0.17	0.00	0.054	520	-0.08	-0.18	0.02	0.099
Total cholesterol to total lipids ratio in chylomicrons and extremely large VLDL (%)	520	0.06	-0.03	0.15	0.169	520	0.01	-0.08	0.11	0.776	520	-0.07	-0.18	0.03	0.161
Cholesterol esters to total lipids ratio in chylomicrons and extremely large VLDL (%)	520	0.07	-0.01	0.16	0.099	520	0.04	-0.05	0.13	0.418	520	-0.05	-0.16	0.05	0.296
Free cholesterol to total lipids ratio in chylomicrons and extremely large VLDL (%)	520	-0.01	-0.09	0.08	0.901	520	-0.06	-0.15	0.03	0.176	520	-0.08	-0.18	0.02	0.125
Triglycerides to total lipids ratio in chylomicrons and extremely large VLDL (%)	520	-0.04	-0.10	0.02	0.240	520	0.01	-0.05	0.07	0.814	520	0.07	-0.01	0.14	0.072
Phospholipids to total lipds ratio in very large VLDL (%)	520	-0.01	-0.10	0.07	0.743	520	-0.09	-0.18	0.01	0.065	520	-0.10	-0.20	0.00	0.045
Total cholesterol to total lipids ratio in very large VLDL (%)	520	0.06	-0.03	0.15	0.184	520	0.03	-0.06	0.13	0.461	520	-0.01	-0.12	0.09	0.807
Cholesterol esters to total lipids ratio in very large VLDL (%)	520	0.06	-0.03	0.16	0.213	520	0.05	-0.05	0.15	0.304	520	0.01	-0.10	0.12	0.853
Free cholesterol to total lipids ratio in very large VLDL (%)	520	0.06	-0.04	0.17	0.231	520	0.01	-0.10	0.12	0.858	520	-0.05	-0.17	0.07	0.419
Triglycerides to total lipids ratio in very large VLDL (%)	520	-0.07	-0.17	0.04	0.203	520	-0.01	-0.12	0.10	0.852	520	0.05	-0.07	0.17	0.412
Phospholipids to total lipds ratio in large VLDL (%)	520	0.00	-0.09	0.10	0.964	520	-0.04	-0.14	0.06	0.443	520	-0.06	-0.18	0.05	0.265
Total cholesterol to total lipids ratio in large VLDL (%)	520	0.03	-0.05	0.11	0.489	520	-0.01	-0.11	0.08	0.766	520	-0.04	-0.14	0.05	0.370
Cholesterol esters to total lipids ratio in large VLDL (%)	520	0.03	0.00	0.07	0.081	520	0.01	-0.03	0.05	0.643	520	-0.02	-0.07	0.03	0.387
Free cholesterol to total lipids ratio in large VLDL (%)	520	-0.02	-0.11	0.06	0.577	520	-0.06	-0.16	0.04	0.225	520	-0.05	-0.17	0.06	0.335
Triglycerides to total lipids ratio in large VLDL (%)	520	0.00	-0.01	0.01	0.642	520	0.00	-0.01	0.01	0.739	520	0.01	-0.01	0.02	0.437
Phospholipids to total lipds ratio in medium VLDL (%)	520	0.05	-0.05	0.14	0.331	520	0.05	-0.06	0.16	0.352	520	0.03	-0.09	0.14	0.636
Total cholesterol to total lipids ratio in medium VLDL (%)	520	0.09	0.02	0.16	0.008	520	0.05	-0.02	0.12	0.156	520	-0.04	-0.13	0.04	0.337
Cholesterol esters to total lipids ratio in medium VLDL (%)	520	0.10	0.03	0.18	0.007	520	0.06	-0.01	0.14	0.104	520	-0.04	-0.13	0.06	0.454
Free cholesterol to total lipids ratio in medium VLDL (%)	520	0.01	-0.08	0.09	0.891	520	-0.01	-0.11	0.08	0.753	520	-0.04	-0.13	0.06	0.443
Triglycerides to total lipids ratio in medium VLDL (%)	520	-0.09	-0.16	-0.02	0.008	520	-0.06	-0.13	0.02	0.124	520	0.03	-0.05	0.12	0.459
Phospholipids to total lipds ratio in small VLDL (%)	520	0.08	0.00	0.17	0.052	520	0.09	0.00	0.17	0.043	520	0.01	-0.08	0.10	0.847
Total cholesterol to total lipids ratio in small VLDL (%)	520	0.04	-0.04	0.12	0.357	520	0.01	-0.08	0.10	0.836	520	-0.03	-0.13	0.07	0.546
Cholesterol esters to total lipids ratio in small VLDL (%)	520	0.02	-0.06	0.10	0.611	520	-0.01	-0.09	0.08	0.835	520	-0.03	-0.13	0.06	0.484
Free cholesterol to total lipids ratio in small VLDL (%)	520	0.12	0.04	0.21	0.006	520	0.11	0.01	0.21	0.025	520	0.01	-0.09	0.12	0.812
Triglycerides to total lipids ratio in small VLDL (%)	520	-0.06	-0.15	0.03	0.172	520	-0.04	-0.13	0.06	0.443	520	0.02	-0.08	0.12	0.681
Phospholipids to total lipds ratio in very small VLDL (%)	520	0.03	-0.06	0.11	0.556	520	-0.01	-0.10	0.08	0.830	520	-0.03	-0.12	0.05	0.423
Total cholesterol to total lipids ratio in very small VLDL (%)	520	0.00	-0.09	0.09	0.960	520	0.04	-0.06	0.13	0.423	520	0.05	-0.05	0.14	0.348
Cholesterol esters to total lipids ratio in very small VLDL (%)	520	-0.04	-0.12	0.04	0.376	520	-0.01	-0.10	0.07	0.726	520	0.01	-0.08	0.09	0.903
Free cholesterol to total lipids ratio in very small VLDL (%)	520	0.08	-0.02	0.18	0.103	520	0.13	0.03	0.24	0.015	520	0.10	-0.01	0.22	0.085
Triglycerides to total lipids ratio in very small VLDL (%)	520	-0.02	-0.11	0.06	0.594	520	-0.03	-0.12	0.06	0.487	520	-0.01	-0.11	0.08	0.782
Phospholipids to total lipds ratio in IDL (%)	520	-0.01	-0.10	0.08	0.882	520	-0.02	-0.11	0.08	0.700	520	-0.07	-0.19	0.05	0.273
Total cholesterol to total lipids ratio in IDL (%)	520	0.00	-0.08	0.08	0.989	520	0.00	-0.09	0.08	0.940	520	0.01	-0.09	0.11	0.803
Cholesterol esters to total lipids ratio in IDL (%)	520	-0.01	-0.10	0.07	0.768	520	-0.01	-0.10	0.08	0.839	520	0.02	-0.09	0.12	0.740
Free cholesterol to total lipids ratio in IDL (%)	520	0.02	-0.06	0.10	0.643	520	0.02	-0.07	0.10	0.685	520	0.01	-0.08	0.10	0.791
Triglycerides to total lipids ratio in IDL (%)	520	0.01	-0.08	0.09	0.877	520	0.01	-0.07	0.09	0.814	520	0.01	-0.09	0.10	0.897
Phospholipids to total lipds ratio in large LDL (%)	520	-0.01	-0.09	0.06	0.762	520	0.02	-0.06	0.09	0.703	520	0.01	-0.06	0.08	0.734
Total cholesterol to total lipids ratio in large LDL (%)	520	0.02	-0.05	0.10	0.544	520	-0.02	-0.10	0.06	0.602	520	-0.05	-0.12	0.03	0.243
Cholesterol esters to total lipids ratio in large LDL (%)	520	0.02	-0.05	0.10	0.516	520	-0.03	-0.11	0.05	0.441	520	-0.06	-0.13	0.01	0.071
Free cholesterol to total lipids ratio in large LDL (%)	520	0.00	-0.07	0.07	0.968	520	0.06	-0.02	0.13	0.147	520	0.06	-0.02	0.14	0.121
Triglycerides to total lipids ratio in large LDL (%)	520	-0.01	-0.09	0.07	0.826	520	0.01	-0.07	0.10	0.785	520	0.03	-0.07	0.13	0.595
Phospholipids to total lipds ratio in medium LDL (%)	520	0.00	-0.03	0.03	0.915	520	0.01	-0.02	0.05	0.486	520	0.01	-0.02	0.04	0.568
Total cholesterol to total lipids ratio in medium LDL (%)	520	0.01	-0.08	0.09	0.892	520	-0.03	-0.12	0.06	0.485	520	-0.03	-0.11	0.05	0.479
Cholesterol esters to total lipids ratio in medium LDL (%)	520	0.01	-0.08	0.09	0.871	520	-0.04	-0.13	0.05	0.398	520	-0.04	-0.13	0.04	0.292
Free cholesterol to total lipids ratio in medium LDL (%)	520	0.00	-0.03	0.02	0.875	520	0.01	-0.01	0.04	0.297	520	0.02	-0.01	0.04	0.178
Triglycerides to total lipids ratio in medium LDL (%)	520	0.01	-0.07	0.10	0.740	520	0.00	-0.08	0.09	0.943	520	-0.02	-0.12	0.08	0.710
Phospholipids to total lipds ratio in small LDL (%)	520	-0.01	-0.06	0.04	0.763	520	0.02	-0.03	0.07	0.476	520	0.02	-0.02	0.07	0.319
Total cholesterol to total lipids ratio in small LDL (%)	520	0.02	-0.07	0.10	0.681	520	-0.02	-0.11	0.06	0.575	520	-0.04	-0.12	0.05	0.372

Cholesterol esters to total lipids ratio in small LDL (%)	520	0.01	-0.08	0.10	0.820	520	-0.04	-0.13	0.06	0.442	520	-0.05	-0.13	0.04	0.293
Free cholesterol to total lipids ratio in small LDL (%)	520	0.00	-0.05	0.05	0.988	520	0.03	-0.02	0.08	0.252	520	0.03	-0.02	0.09	0.186
Triglycerides to total lipids ratio in small LDL (%)	520	0.00	-0.09	0.08	0.973	520	-0.03	-0.11	0.05	0.477	520	-0.04	-0.14	0.05	0.372
Phospholipids to total lipds ratio in very large HDL (%)	520	0.03	-0.05	0.12	0.407	520	0.06	-0.04	0.15	0.242	520	0.04	-0.03	0.11	0.308
Total cholesterol to total lipids ratio in very large HDL (%)	520	-0.03	-0.11	0.05	0.433	520	-0.05	-0.15	0.04	0.268	520	-0.04	-0.11	0.03	0.292
Cholesterol esters to total lipids ratio in very large HDL (%)	520	-0.03	-0.11	0.05	0.475	520	-0.05	-0.15	0.04	0.273	520	-0.04	-0.11	0.03	0.253
Free cholesterol to total lipids ratio in very large HDL (%)	520	0.00	-0.09	0.09	1.000	520	0.02	-0.08	0.11	0.741	520	0.03	-0.07	0.13	0.497
Triglycerides to total lipids ratio in very large HDL (%)	520	-0.02	-0.11	0.07	0.689	520	-0.03	-0.10	0.05	0.520	520	0.00	-0.09	0.09	0.958
Phospholipids to total lipds ratio in large HDL (%)	520	-0.04	-0.12	0.05	0.395	520	-0.10	-0.18	-0.01	0.030	520	-0.10	-0.19	-0.01	0.026
Total cholesterol to total lipids ratio in large HDL (%)	520	0.04	-0.05	0.12	0.394	520	0.09	0.00	0.18	0.044	520	0.08	0.00	0.17	0.052
Cholesterol esters to total lipids ratio in large HDL (%)	520	0.04	-0.05	0.12	0.417	520	0.09	0.01	0.18	0.034	520	0.09	0.00	0.17	0.043
Free cholesterol to total lipids ratio in large HDL (%)	520	0.03	-0.05	0.12	0.432	520	0.06	-0.03	0.14	0.205	520	0.05	-0.03	0.14	0.210
Triglycerides to total lipids ratio in large HDL (%)	520	-0.03	-0.11	0.06	0.544	520	-0.05	-0.14	0.03	0.212	520	-0.04	-0.12	0.05	0.422
Phospholipids to total lipds ratio in medium HDL (%)	520	0.08	-0.02	0.17	0.112	520	0.02	-0.07	0.11	0.671	520	-0.07	-0.18	0.04	0.244
Total cholesterol to total lipids ratio in medium HDL (%)	520	-0.05	-0.13	0.04	0.305	520	0.02	-0.06	0.11	0.598	520	0.09	-0.01	0.19	0.083
Cholesterol esters to total lipids ratio in medium HDL (%)	520	-0.05	-0.13	0.04	0.288	520	0.03	-0.05	0.12	0.427	520	0.11	0.00	0.21	0.051
Free cholesterol to total lipids ratio in medium HDL (%)	520	-0.01	-0.10	0.08	0.798	520	-0.05	-0.16	0.07	0.410	520	-0.04	-0.17	0.10	0.577
Triglycerides to total lipids ratio in medium HDL (%)	520	-0.02	-0.11	0.06	0.580	520	-0.06	-0.15	0.03	0.159	520	-0.07	-0.16	0.02	0.132
Phospholipids to total lipds ratio in small HDL (%)	520	-0.12	-0.19	-0.04	1.75E-03	520	-0.04	-0.12	0.04	0.305	520	0.08	0.00	0.17	0.050
Total cholesterol to total lipids ratio in small HDL (%)	520	0.12	0.05	0.20	1.49E-03	520	0.06	-0.02	0.14	0.167	520	-0.07	-0.16	0.01	0.103
Cholesterol esters to total lipids ratio in small HDL (%)	520	0.12	0.05	0.20	1.77E-03	520	0.05	-0.04	0.13	0.279	520	-0.09	-0.17	0.00	0.048
Free cholesterol to total lipids ratio in small HDL (%)	520	-0.02	-0.10	0.05	0.540	520	0.08	-0.01	0.16	0.072	520	0.14	0.04	0.23	0.005
Triglycerides to total lipids ratio in small HDL (%)	520	-0.05	-0.14	0.03	0.215	520	-0.07	-0.16	0.01	0.099	520	-0.02	-0.12	0.07	0.642
Mean diameter for VLDL particles (nm)	520	-0.07	-0.15	0.01	0.088	520	-0.08	-0.17	0.01	0.083	520	-0.02	-0.11	0.08	0.686
Mean diameter for LDL particles (nm)	520	0.01	-0.07	0.09	0.781	520	0.05	-0.02	0.13	0.179	520	0.05	-0.03	0.14	0.221
Mean diameter for HDL particles (nm)	520	0.03	-0.05	0.11	0.477	520	0.06	-0.04	0.16	0.231	520	0.05	-0.03	0.14	0.203
Serum total cholesterol (mmol/l)	520	0.03	-0.04	0.10	0.392	520	-0.02	-0.09	0.06	0.694	520	-0.07	-0.14	0.01	0.080
Total cholesterol in VLDL (mmol/l)	520	-0.01	-0.07	0.06	0.874	520	-0.05	-0.12	0.01	0.108	520	-0.07	-0.14	0.01	0.106
Remnant cholesterol (non-HDL, non-LDL -cholesterol) (mmol/l)	520	0.01	-0.05	0.07	0.707	520	-0.04	-0.11	0.02	0.167	520	-0.08	-0.15	-0.01	0.034
Total cholesterol in LDL (mmol/l)	520	0.02	-0.06	0.09	0.635	520	-0.03	-0.11	0.05	0.468	520	-0.07	-0.15	0.01	0.077
Total cholesterol in HDL (mmol/l)	520	0.05	-0.03	0.13	0.211	520	0.04	-0.05	0.14	0.331	520	0.00	-0.08	0.08	0.968
Total cholesterol in HDL2 (mmol/l)	520	0.05	-0.03	0.13	0.235	520	0.05	-0.04	0.14	0.308	520	0.01	-0.07	0.09	0.852
Total cholesterol in HDL3 (mmol/l)	520	0.05	-0.03	0.13	0.187	520	0.04	-0.05	0.13	0.393	520	-0.01	-0.09	0.07	0.806
Esterified cholesterol (mmol/l)	520	0.02	-0.05	0.10	0.502	520	-0.02	-0.10	0.06	0.605	520	-0.07	-0.15	0.01	0.077
Free cholesterol (mmol/l)	520	0.04	-0.03	0.12	0.220	520	-0.01	-0.09	0.07	0.898	520	-0.06	-0.14	0.02	0.118
Serum total triglycerides (mmol/l)	520	-0.04	-0.12	0.03	0.285	520	-0.07	-0.15	0.01	0.076	520	-0.03	-0.13	0.06	0.469
Triglycerides in VLDL (mmol/l)	520	-0.05	-0.13	0.02	0.177	520	-0.07	-0.15	0.01	0.081	520	-0.02	-0.11	0.07	0.687
Triglycerides in LDL (mmol/l)	520	0.01	-0.08	0.09	0.858	520	-0.05	-0.13	0.04	0.291	520	-0.07	-0.17	0.02	0.140
Triglycerides in HDL (mmol/l)	520	0.00	-0.08	0.07	0.901	520	-0.04	-0.12	0.04	0.340	520	-0.04	-0.13	0.05	0.393
Diacylglycerol (mmol/l)	520	0.05	-0.03	0.13	0.230	520	0.05	-0.04	0.15	0.249	520	0.02	-0.08	0.12	0.730
Ratio of diacylglycerol to triglycerides	520	0.07	-0.03	0.16	0.153	520	0.08	-0.03	0.19	0.165	520	0.01	-0.10	0.12	0.900
Total phosphoglycerides (mmol/l)	520	0.05	-0.03	0.13	0.238	520	0.02	-0.06	0.11	0.591	520	-0.03	-0.12	0.06	0.542
Ratio of triglycerides to phosphoglycerides	520	-0.04	-0.12	0.04	0.276	520	-0.06	-0.13	0.02	0.149	520	0.01	-0.08	0.10	0.858
Phosphatidylcholine and other cholines (mmol/l)	520	0.06	-0.01	0.14	0.111	520	0.01	-0.07	0.10	0.764	520	-0.05	-0.14	0.04	0.291
Total cholines (mmol/l)	520	0.06	-0.02	0.14	0.159	520	0.02	-0.07	0.11	0.659	520	-0.04	-0.13	0.05	0.363
Apolipoprotein A-I (g/l)	520	0.04	-0.04	0.12	0.301	520	0.02	-0.06	0.11	0.602	520	-0.02	-0.09	0.06	0.682
Apolipoprotein B (g/l)	520	-0.01	-0.08	0.06	0.795	520	-0.06	-0.13	0.02	0.122	520	-0.07	-0.15	0.01	0.099
Ratio of apolipoprotein B to apolipoprotein A-I	520	-0.03	-0.10	0.05	0.460	520	-0.07	-0.15	0.01	0.087	520	-0.06	-0.14	0.02	0.152
Total fatty acids (mmol/l)	520	-0.01	-0.08	0.07	0.853	520	-0.04	-0.12	0.03	0.272	520	-0.03	-0.12	0.05	0.431
Estimated description of fatty acid chain length, not actual carbon number	520	0.04	-0.05	0.12	0.414	520	0.01	-0.08	0.09	0.882	520	-0.02	-0.11	0.07	0.627
Estimated degree of unsaturation	520	0.07	-0.03	0.16	0.154	520	0.02	-0.08	0.11	0.738	520	-0.05	-0.15	0.05	0.322
22:6, docosahexaenoic acid (mmol/l)	520	0.01	-0.07	0.09	0.794	520	-0.02	-0.11	0.06	0.598	520	-0.07	-0.17	0.02	0.139
18:2, linoleic acid (mmol/l)	520	0.04	-0.04	0.11	0.314	520	-0.01	-0.09	0.06	0.763	520	-0.02	-0.11	0.06	0.556
Conjugated linoleic acid (mmol/l)	520	0.02	-0.07	0.10	0.690	520	-0.02	-0.10	0.07	0.722	520	-0.03	-0.12	0.06	0.543
Omega-3 fatty acids (mmol/l)	520	-0.06	-0.14	0.02	0.129	520	-0.06	-0.15	0.02	0.145	520	-0.02	-0.12	0.07	0.611
Omega-6 fatty acids (mmol/l)	520	0.03	-0.05	0.10	0.450	520	-0.02	-0.09	0.06	0.687	520	-0.03	-0.11	0.05	0.497
Polyunsaturated fatty acids (mmol/l)	520	0.02	-0.06	0.09	0.656	520	-0.02	-0.10	0.05	0.548	520	-0.03	-0.11	0.05	0.494

Monounsaturated fatty acids; 16:1, 18:1 (mmol/l)	520	-0.01	-0.08	0.06	0.791	520	-0.05	-0.13	0.02	0.184	520	-0.05	-0.14	0.04	0.251
Saturated fatty acids (mmol/l)	520	-0.03	-0.11	0.06	0.545	520	-0.04	-0.13	0.04	0.308	520	-0.02	-0.11	0.07	0.720
Ratio of 22:6 docosahexaenoic acid to total fatty acids (%)	520	0.03	-0.07	0.12	0.595	520	0.00	-0.09	0.10	0.944	520	-0.07	-0.18	0.03	0.178
Ratio of 18:2 linoleic acid to total fatty acids (%)	520	0.08	-0.01	0.16	0.066	520	0.06	-0.03	0.15	0.173	520	0.04	-0.05	0.13	0.356
Ratio of conjugated linoleic acid to total fatty acids (%)	520	0.02	-0.07	0.10	0.670	520	-0.01	-0.10	0.07	0.777	520	-0.02	-0.12	0.07	0.638
Ratio of omega-3 fatty acids to total fatty acids (%)	520	-0.06	-0.15	0.02	0.157	520	-0.04	-0.13	0.06	0.426	520	0.00	-0.11	0.10	0.944
Ratio of omega-6 fatty acids to total fatty acids (%)	520	0.08	-0.01	0.16	0.082	520	0.07	-0.03	0.16	0.158	520	0.05	-0.05	0.14	0.345
Ratio of polyunsaturated fatty acids to total fatty acids (%)	520	0.06	-0.03	0.15	0.173	520	0.06	-0.04	0.15	0.236	520	0.04	-0.05	0.14	0.376
Ratio of monounsaturated fatty acids to total fatty acids (%)	520	-0.01	-0.10	0.08	0.864	520	-0.04	-0.14	0.06	0.410	520	-0.07	-0.17	0.03	0.172
Ratio of saturated fatty acids to total fatty acids (%)	520	-0.06	-0.16	0.04	0.214	520	-0.01	-0.11	0.09	0.798	520	0.03	-0.06	0.12	0.492
Glucose (mmol/l)	520	-0.04	-0.12	0.05	0.398	520	-0.04	-0.12	0.05	0.378	520	0.00	-0.10	0.10	0.957
Lactate (mmol/l)	520	-0.04	-0.14	0.05	0.355	520	0.02	-0.08	0.11	0.752	520	0.08	-0.02	0.19	0.106
Pyruvate (mmol/l)	520	-0.10	-0.20	-0.01	0.034	520	-0.04	-0.13	0.05	0.352	520	0.09	-0.01	0.19	0.090
Citrate (mmol/l)	520	0.12	0.03	0.22	0.011	520	0.05	-0.05	0.16	0.334	520	-0.10	-0.21	0.01	0.064
Alanine (mmol/l)	520	-0.08	-0.18	0.01	0.097	520	0.02	-0.07	0.12	0.609	520	0.17	0.06	0.28	1.76E-03
Glutamine (mmol/l)	520	0.05	-0.02	0.13	0.165	520	0.05	-0.03	0.13	0.234	520	-0.03	-0.12	0.06	0.551
Histidine (mmol/l)	520	0.07	-0.03	0.16	0.172	520	0.11	0.01	0.21	0.037	520	0.08	-0.02	0.18	0.104
Isoleucine (mmol/l)	520	-0.03	-0.12	0.05	0.425	520	0.01	-0.08	0.10	0.801	520	0.06	-0.04	0.16	0.250
Leucine (mmol/l)	520	0.05	-0.03	0.13	0.266	520	0.08	0.00	0.16	0.051	520	0.04	-0.05	0.13	0.393
Valine (mmol/l)	520	0.02	-0.07	0.10	0.693	520	0.06	-0.03	0.15	0.216	520	0.05	-0.05	0.15	0.321
Phenylalanine (mmol/l)	520	0.06	-0.04	0.15	0.254	520	0.08	-0.02	0.17	0.106	520	0.00	-0.11	0.11	0.978
Tyrosine (mmol/l)	520	0.05	-0.04	0.15	0.283	520	0.03	-0.08	0.13	0.636	520	-0.04	-0.14	0.06	0.455
Acetate (mmol/l)	520	0.06	-0.02	0.15	0.158	520	0.11	0.02	0.20	0.015	520	0.08	-0.02	0.19	0.129
Acetoacetate (mmol/l)	520	0.03	-0.04	0.10	0.374	520	0.02	-0.06	0.09	0.657	520	-0.04	-0.12	0.05	0.440
3-hydroxybutyrate (mmol/l)	520	-0.01	-0.09	0.07	0.838	520	-0.06	-0.14	0.02	0.151	520	-0.07	-0.18	0.04	0.213
Creatinine (mmol/l)	520	-0.07	-0.15	0.01	0.101	520	-0.03	-0.12	0.06	0.510	520	0.04	-0.05	0.13	0.355
Albumin (signal area)	520	-0.13	-0.22	-0.04	0.006	520	-0.05	-0.14	0.04	0.294	520	0.09	-0.02	0.20	0.101
Glycoprotein acetyls, mainly a1-acid glycoprotein (mmol/l)	520	-0.10	-0.18	-0.02	0.013	520	-0.13	-0.20	-0.06	3.43E-04	520	-0.03	-0.12	0.06	0.519
