# Supplementary material for: Associations of device-measured physical activity across adolescence with metabolic traits: Prospective cohort study
Source: PLoS Med. 2018 Sep 11;15(9):e1002649. doi: 10.1371/journal.pmed.1002649 (PMC6133272; doi:10.1371/journal.pmed.1002649)
Supplement: S13 Table — ALSPAC, Avon Longitudinal Study of Parents and Children. (PDF) [file pmed.1002649.s013.pdf]

S13 Table Associations of longer-term physical activity (mean of measures at age 12y, 14y, 15y) with metabolic traits at age 15y, with adjustment for metabolic traits at age 8y in ALSPAC

	**Mean of CPM at age 12y, 14y, 15y (per SD higher)**					**Mean of MVPA at age 12y, 14y, 15y (per SD higher)**					**Mean of SED at age 12y, 14y, 15y (per SD higher)**				
	Adj. for age, sex, ethnicity, maternal education, mean wear time, wear month, mean FMI, metabolic trait at 8y					*Adj. for age, sex, ethnicity, maternal education, mean wear time, wear month, mean SED, mean FMI, metabolic trait at 8y*					*Adj. for age, sex, ethnicity, maternal education, mean wear time, wear month, mean MVPA, mean FMI, metabolic trait at 8y*				
**Standardised outcome at age 15y**	**N**	**Beta**	**LCL**	**UCL**	**P-value**	**N**	**Beta**	**LCL**	**UCL**	**P-value**	**N**	**Beta**	**LCL**	**UCL**	**P-value**
Systolic blood pressure (mmHg)	1162	-0.08	-0.16	0.00	0.055	1162	-0.09	-0.18	-0.01	0.030	1162	0.00	-0.10	0.09	0.926
Diastolic blood pressure (mmHg)	1161	-0.12	-0.21	-0.03	0.010	1161	-0.02	-0.11	0.07	0.675	1161	0.07	-0.03	0.16	0.169
Concentration of chylomicrons and extremely large VLDL particles (mol/l)	628	-0.15	-0.25	-0.05	0.005	628	-0.19	-0.30	-0.08	6.20E-04	628	-0.03	-0.16	0.09	0.587
Total lipids in chylomicrons and extremely large VLDL (mmol/l)	628	-0.14	-0.25	-0.04	0.005	628	-0.19	-0.30	-0.08	6.94E-04	628	-0.03	-0.16	0.09	0.613
Phospholipids in chylomicrons and extremely large VLDL (mmol/l)	628	-0.15	-0.25	-0.05	0.004	628	-0.20	-0.31	-0.08	6.01E-04	628	-0.04	-0.16	0.09	0.592
Total cholesterol in chylomicrons and extremely large VLDL (mmol/l)	628	-0.11	-0.21	-0.02	0.023	628	-0.16	-0.26	-0.06	2.14E-03	628	-0.03	-0.14	0.08	0.574
Cholesterol esters in chylomicrons and extremely large VLDL (mmol/l)	628	-0.07	-0.16	0.02	0.138	628	-0.12	-0.22	-0.03	0.012	628	-0.04	-0.14	0.07	0.513
Free cholesterol in chylomicrons and extremely large VLDL (mmol/l)	628	-0.15	-0.25	-0.04	0.005	628	-0.19	-0.30	-0.08	8.04E-04	628	-0.03	-0.16	0.09	0.627
Triglycerides in chylomicrons and extremely large VLDL (mmol/l)	628	-0.15	-0.25	-0.05	0.004	628	-0.20	-0.31	-0.08	5.99E-04	628	-0.03	-0.16	0.09	0.622
Concentration of very large VLDL particles (mol/l)	628	-0.12	-0.22	-0.02	0.017	628	-0.17	-0.28	-0.06	2.18E-03	628	-0.03	-0.15	0.09	0.599
Total lipids in very large VLDL (mmol/l)	628	-0.12	-0.22	-0.02	0.020	628	-0.17	-0.28	-0.06	2.49E-03	628	-0.03	-0.15	0.09	0.591
Phospholipids in very large VLDL (mmol/l)	628	-0.13	-0.23	-0.02	0.015	628	-0.17	-0.28	-0.06	2.07E-03	628	-0.03	-0.16	0.09	0.594
Total cholesterol in very large VLDL (mmol/l)	628	-0.12	-0.22	-0.02	0.016	628	-0.17	-0.28	-0.07	1.51E-03	628	-0.03	-0.15	0.08	0.567
Cholesterol esters in very large VLDL (mmol/l)	628	-0.11	-0.20	-0.01	0.030	628	-0.16	-0.26	-0.06	2.45E-03	628	-0.03	-0.15	0.08	0.547
Free cholesterol in very large VLDL (mmol/l)	628	-0.13	-0.23	-0.03	0.009	628	-0.18	-0.29	-0.07	1.05E-03	628	-0.03	-0.16	0.09	0.577
Triglycerides in very large VLDL (mmol/l)	628	-0.12	-0.22	-0.02	0.024	628	-0.17	-0.28	-0.06	0.003	628	-0.03	-0.15	0.09	0.598
Concentration of large VLDL particles (mol/l)	628	-0.10	-0.20	0.00	0.054	628	-0.15	-0.26	-0.04	0.006	628	-0.03	-0.15	0.08	0.597
Total lipids in large VLDL (mmol/l)	628	-0.10	-0.20	0.00	0.059	628	-0.15	-0.26	-0.04	0.007	628	-0.03	-0.15	0.08	0.579
Phospholipids in large VLDL (mmol/l)	628	-0.10	-0.20	0.00	0.056	628	-0.15	-0.26	-0.04	0.006	628	-0.03	-0.15	0.08	0.562
Total cholesterol in large VLDL (mmol/l)	628	-0.08	-0.18	0.02	0.103	628	-0.14	-0.25	-0.04	0.009	628	-0.04	-0.15	0.07	0.490
Cholesterol esters in large VLDL (mmol/l)	628	-0.06	-0.16	0.04	0.209	628	-0.13	-0.23	-0.02	0.017	628	-0.04	-0.15	0.07	0.430
Free cholesterol in large VLDL (mmol/l)	628	-0.10	-0.20	0.00	0.055	628	-0.15	-0.26	-0.04	0.006	628	-0.04	-0.15	0.08	0.543
Triglycerides in large VLDL (mmol/l)	628	-0.10	-0.20	0.00	0.049	628	-0.15	-0.26	-0.04	0.006	628	-0.03	-0.14	0.09	0.620
Concentration of medium VLDL particles (mol/l)	628	-0.09	-0.19	0.01	0.081	628	-0.15	-0.26	-0.04	0.008	628	-0.04	-0.15	0.08	0.512
Total lipids in medium VLDL (mmol/l)	628	-0.08	-0.18	0.02	0.109	628	-0.14	-0.25	-0.04	0.010	628	-0.04	-0.15	0.07	0.483
Phospholipids in medium VLDL (mmol/l)	628	-0.08	-0.18	0.02	0.110	628	-0.15	-0.26	-0.04	0.010	628	-0.04	-0.16	0.07	0.471
Total cholesterol in medium VLDL (mmol/l)	628	-0.04	-0.14	0.05	0.371	628	-0.11	-0.22	-0.01	0.034	628	-0.05	-0.16	0.06	0.418
Cholesterol esters in medium VLDL (mmol/l)	628	-0.01	-0.11	0.09	0.873	628	-0.08	-0.18	0.02	0.138	628	-0.05	-0.16	0.06	0.389
Free cholesterol in medium VLDL (mmol/l)	628	-0.08	-0.18	0.02	0.108	628	-0.14	-0.25	-0.03	0.011	628	-0.04	-0.15	0.08	0.509
Triglycerides in medium VLDL (mmol/l)	628	-0.10	-0.20	0.00	0.059	628	-0.16	-0.27	-0.04	0.006	628	-0.04	-0.15	0.08	0.531
Concentration of small VLDL particles (mol/l)	628	-0.07	-0.17	0.03	0.162	628	-0.14	-0.25	-0.03	0.015	628	-0.04	-0.16	0.07	0.458
Total lipids in small VLDL (mmol/l)	628	-0.07	-0.16	0.03	0.176	628	-0.14	-0.25	-0.03	0.014	628	-0.05	-0.17	0.07	0.396
Phospholipids in small VLDL (mmol/l)	628	-0.03	-0.13	0.06	0.492	628	-0.11	-0.22	-0.01	0.040	628	-0.06	-0.17	0.05	0.302
Total cholesterol in small VLDL (mmol/l)	628	-0.05	-0.13	0.04	0.299	628	-0.11	-0.21	-0.01	0.025	628	-0.05	-0.16	0.05	0.310
Cholesterol esters in small VLDL (mmol/l)	628	-0.04	-0.13	0.04	0.298	628	-0.10	-0.19	-0.01	0.030	628	-0.05	-0.16	0.05	0.304
Free cholesterol in small VLDL (mmol/l)	628	-0.04	-0.13	0.06	0.439	628	-0.11	-0.21	0.00	0.041	628	-0.05	-0.16	0.06	0.357
Triglycerides in small VLDL (mmol/l)	628	-0.08	-0.19	0.02	0.120	628	-0.14	-0.26	-0.03	0.016	628	-0.03	-0.16	0.09	0.578
Concentration of very small VLDL particles (mol/l)	628	0.00	-0.08	0.08	0.968	628	-0.04	-0.12	0.05	0.399	628	-0.02	-0.11	0.08	0.723
Total lipids in very small VLDL (mmol/l)	628	-0.02	-0.10	0.06	0.699	628	-0.05	-0.14	0.03	0.231	628	-0.02	-0.12	0.07	0.653
Phospholipids in very small VLDL (mmol/l)	628	-0.02	-0.10	0.06	0.606	628	-0.04	-0.13	0.04	0.331	628	-0.01	-0.10	0.09	0.909
Total cholesterol in very small VLDL (mmol/l)	628	0.02	-0.08	0.11	0.738	628	-0.02	-0.11	0.08	0.735	628	-0.03	-0.14	0.08	0.598
Cholesterol esters in very small VLDL (mmol/l)	628	-0.01	-0.10	0.08	0.790	628	-0.04	-0.14	0.05	0.363	628	-0.03	-0.14	0.07	0.531
Free cholesterol in very small VLDL (mmol/l)	628	0.07	-0.03	0.17	0.159	628	0.04	-0.07	0.15	0.440	628	-0.01	-0.13	0.11	0.862
Triglycerides in very small VLDL (mmol/l)	628	-0.06	-0.16	0.04	0.255	628	-0.11	-0.22	0.01	0.064	628	-0.02	-0.14	0.10	0.752
Concentration of IDL particles (mol/l)	628	-0.06	-0.14	0.03	0.222	628	-0.05	-0.15	0.05	0.294	628	0.02	-0.08	0.12	0.726
Total lipids in IDL (mmol/l)	628	-0.03	-0.11	0.05	0.422	628	-0.04	-0.13	0.05	0.409	628	0.01	-0.09	0.10	0.881

Phospholipids in IDL (mmol/l)	628	-0.05	-0.14	0.04	0.304	628	-0.04	-0.15	0.06	0.395	628	0.01	-0.09	0.12	0.812
Total cholesterol in IDL (mmol/l)	628	-0.02	-0.09	0.06	0.672	628	-0.03	-0.12	0.05	0.462	628	-0.01	-0.10	0.08	0.858
Cholesterol esters in IDL (mmol/l)	628	-0.01	-0.09	0.06	0.778	628	-0.04	-0.12	0.04	0.353	628	-0.02	-0.11	0.07	0.624
Free cholesterol in IDL (mmol/l)	628	-0.03	-0.11	0.06	0.535	628	-0.02	-0.11	0.08	0.741	628	0.02	-0.08	0.11	0.698
Triglycerides in IDL (mmol/l)	628	-0.03	-0.13	0.07	0.540	628	-0.05	-0.15	0.06	0.401	628	0.01	-0.10	0.12	0.840
Concentration of large LDL particles (mol/l)	628	-0.05	-0.15	0.04	0.278	628	-0.07	-0.18	0.04	0.228	628	-0.01	-0.12	0.10	0.883
Total lipids in large LDL (mmol/l)	628	-0.04	-0.13	0.04	0.329	628	-0.05	-0.15	0.05	0.302	628	0.00	-0.10	0.10	0.963
Phospholipids in large LDL (mmol/l)	628	-0.04	-0.12	0.05	0.396	628	-0.06	-0.15	0.04	0.268	628	-0.01	-0.11	0.09	0.831
Total cholesterol in large LDL (mmol/l)	628	-0.04	-0.12	0.05	0.369	628	-0.05	-0.14	0.05	0.337	628	0.00	-0.10	0.10	0.990
Cholesterol esters in large LDL (mmol/l)	628	-0.04	-0.13	0.04	0.326	628	-0.05	-0.15	0.04	0.274	628	0.00	-0.10	0.10	0.973
Free cholesterol in large LDL (mmol/l)	628	-0.03	-0.11	0.06	0.530	628	-0.03	-0.12	0.07	0.578	628	0.01	-0.09	0.10	0.896
Triglycerides in large LDL (mmol/l)	628	-0.04	-0.15	0.06	0.435	628	-0.06	-0.17	0.06	0.314	628	0.00	-0.12	0.12	0.937
Concentration of medium LDL particles (mol/l)	628	-0.07	-0.17	0.03	0.199	628	-0.09	-0.21	0.02	0.121	628	-0.02	-0.14	0.10	0.692
Total lipids in medium LDL (mmol/l)	628	-0.05	-0.15	0.04	0.248	628	-0.07	-0.17	0.03	0.193	628	-0.01	-0.11	0.10	0.905
Phospholipids in medium LDL (mmol/l)	628	-0.03	-0.11	0.05	0.485	628	-0.07	-0.16	0.02	0.144	628	-0.03	-0.13	0.07	0.528
Total cholesterol in medium LDL (mmol/l)	628	-0.05	-0.15	0.04	0.242	628	-0.06	-0.17	0.04	0.224	628	0.00	-0.11	0.10	0.955
Cholesterol esters in medium LDL (mmol/l)	628	-0.06	-0.16	0.03	0.192	628	-0.07	-0.18	0.04	0.195	628	0.00	-0.11	0.11	0.988
Free cholesterol in medium LDL (mmol/l)	628	-0.01	-0.09	0.07	0.785	628	-0.04	-0.13	0.05	0.425	628	-0.02	-0.12	0.07	0.651
Triglycerides in medium LDL (mmol/l)	628	-0.04	-0.15	0.08	0.521	628	-0.08	-0.21	0.04	0.200	628	-0.04	-0.18	0.10	0.573
Concentration of small LDL particles (mol/l)	628	-0.07	-0.17	0.03	0.174	628	-0.10	-0.21	0.02	0.095	628	-0.03	-0.14	0.09	0.668
Total lipids in small LDL (mmol/l)	628	-0.06	-0.15	0.03	0.212	628	-0.08	-0.18	0.03	0.158	628	-0.01	-0.12	0.10	0.864
Phospholipids in small LDL (mmol/l)	628	-0.04	-0.12	0.05	0.373	628	-0.08	-0.17	0.02	0.124	628	-0.03	-0.13	0.07	0.580
Total cholesterol in small LDL (mmol/l)	628	-0.06	-0.15	0.04	0.230	628	-0.07	-0.17	0.04	0.216	628	0.00	-0.11	0.10	0.943
Cholesterol esters in small LDL (mmol/l)	628	-0.07	-0.17	0.03	0.172	628	-0.08	-0.19	0.03	0.172	628	-0.01	-0.12	0.11	0.928
Free cholesterol in small LDL (mmol/l)	628	0.02	-0.06	0.10	0.617	628	-0.02	-0.11	0.06	0.617	628	-0.04	-0.13	0.05	0.435
Triglycerides in small LDL (mmol/l)	628	-0.06	-0.17	0.05	0.266	628	-0.12	-0.25	0.00	0.053	628	-0.06	-0.20	0.08	0.438
Concentration of very large HDL particles (mol/l)	628	0.01	-0.09	0.11	0.894	628	0.06	-0.04	0.16	0.251	628	0.08	-0.03	0.18	0.143
Total lipids in very large HDL (mmol/l)	628	0.00	-0.10	0.10	0.991	628	0.05	-0.05	0.16	0.321	628	0.07	-0.03	0.18	0.168
Phospholipids in very large HDL (mmol/l)	628	0.02	-0.08	0.11	0.703	628	0.07	-0.03	0.18	0.145	628	0.08	-0.02	0.18	0.129
Total cholesterol in very large HDL (mmol/l)	628	-0.02	-0.12	0.09	0.766	628	0.03	-0.08	0.14	0.624	628	0.06	-0.05	0.18	0.294
Cholesterol esters in very large HDL (mmol/l)	628	-0.02	-0.13	0.08	0.694	628	0.02	-0.09	0.13	0.741	628	0.06	-0.06	0.17	0.348
Free cholesterol in very large HDL (mmol/l)	628	0.00	-0.10	0.10	0.961	628	0.05	-0.06	0.15	0.386	628	0.07	-0.04	0.18	0.199
Triglycerides in very large HDL (mmol/l)	628	-0.04	-0.14	0.06	0.443	628	-0.01	-0.12	0.10	0.864	628	0.08	-0.04	0.19	0.177
Concentration of large HDL particles (mol/l)	628	0.07	-0.02	0.16	0.115	628	0.11	0.02	0.21	0.020	628	0.06	-0.05	0.16	0.295
Total lipids in large HDL (mmol/l)	628	0.07	-0.02	0.16	0.114	628	0.11	0.02	0.21	0.020	628	0.06	-0.05	0.16	0.285
Phospholipids in large HDL (mmol/l)	628	0.06	-0.03	0.15	0.175	628	0.10	0.00	0.20	0.039	628	0.05	-0.05	0.15	0.340
Total cholesterol in large HDL (mmol/l)	628	0.08	-0.01	0.17	0.077	628	0.12	0.03	0.22	0.012	628	0.06	-0.05	0.16	0.273
Cholesterol esters in large HDL (mmol/l)	628	0.08	-0.01	0.17	0.077	628	0.12	0.03	0.22	0.011	628	0.06	-0.04	0.16	0.265
Free cholesterol in large HDL (mmol/l)	628	0.08	-0.01	0.16	0.079	628	0.12	0.02	0.21	0.015	628	0.05	-0.05	0.16	0.314
Triglycerides in large HDL (mmol/l)	628	0.03	-0.06	0.13	0.526	628	0.05	-0.05	0.15	0.354	628	0.05	-0.06	0.15	0.404
Concentration of medium HDL particles (mol/l)	628	0.12	0.03	0.22	0.011	628	0.06	-0.05	0.17	0.275	628	-0.08	-0.20	0.03	0.156
Total lipids in medium HDL (mmol/l)	628	0.12	0.02	0.21	0.015	628	0.06	-0.04	0.17	0.254	628	-0.08	-0.20	0.04	0.179
Phospholipids in medium HDL (mmol/l)	628	0.12	0.02	0.22	0.014	628	0.06	-0.04	0.17	0.254	628	-0.07	-0.19	0.04	0.214
Total cholesterol in medium HDL (mmol/l)	628	0.12	0.02	0.22	0.016	628	0.08	-0.03	0.18	0.150	628	-0.07	-0.19	0.05	0.236
Cholesterol esters in medium HDL (mmol/l)	628	0.12	0.02	0.22	0.017	628	0.08	-0.02	0.19	0.133	628	-0.07	-0.19	0.05	0.242
Free cholesterol in medium HDL (mmol/l)	628	0.11	0.02	0.20	0.022	628	0.06	-0.05	0.17	0.268	628	-0.07	-0.18	0.05	0.260
Triglycerides in medium HDL (mmol/l)	628	-0.02	-0.12	0.08	0.745	628	-0.09	-0.20	0.02	0.127	628	-0.07	-0.19	0.05	0.260
Concentration of small HDL particles (mol/l)	628	0.03	-0.07	0.13	0.557	628	-0.07	-0.18	0.04	0.236	628	-0.12	-0.24	-0.01	0.036
Total lipids in small HDL (mmol/l)	628	0.08	-0.01	0.18	0.090	628	-0.02	-0.13	0.09	0.720	628	-0.13	-0.24	-0.02	0.026
Phospholipids in small HDL (mmol/l)	628	0.00	-0.09	0.10	0.950	628	-0.06	-0.17	0.05	0.288	628	-0.09	-0.21	0.03	0.140
Total cholesterol in small HDL (mmol/l)	628	0.16	0.06	0.25	1.24E-03	628	0.06	-0.05	0.16	0.291	628	-0.12	-0.23	-0.01	0.037
Cholesterol esters in small HDL (mmol/l)	628	0.16	0.06	0.25	1.29E-03	628	0.06	-0.04	0.16	0.265	628	-0.11	-0.22	0.00	0.050
Free cholesterol in small HDL (mmol/l)	628	0.09	-0.01	0.18	0.070	628	0.03	-0.08	0.13	0.618	628	-0.08	-0.20	0.04	0.173
Triglycerides in small HDL (mmol/l)	628	-0.07	-0.17	0.03	0.173	628	-0.12	-0.23	-0.01	0.030	628	-0.03	-0.15	0.10	0.657
Phospholipids to total lipds ratio in chylomicrons and extremely large VLDL (%)	628	-0.05	-0.15	0.06	0.358	628	-0.11	-0.22	0.00	0.058	628	-0.06	-0.18	0.07	0.367
Total cholesterol to total lipids ratio in chylomicrons and extremely large VLDL (%)	628	0.03	-0.08	0.14	0.549	628	-0.04	-0.16	0.07	0.449	628	-0.07	-0.19	0.04	0.218
Cholesterol esters to total lipids ratio in chylomicrons and extremely large VLDL (%)	628	0.07	-0.04	0.17	0.240	628	-0.01	-0.13	0.11	0.865	628	-0.08	-0.20	0.04	0.196

Free cholesterol to total lipids ratio in chylomicrons and extremely large VLDL (%)	628	-0.07	-0.19	0.04	0.221	628	-0.12	-0.24	0.00	0.044	628	-0.02	-0.15	0.11	0.722
Triglycerides to total lipids ratio in chylomicrons and extremely large VLDL (%)	628	-0.01	-0.09	0.06	0.774	628	0.05	-0.03	0.13	0.210	628	0.06	-0.02	0.14	0.157
Phospholipids to total lipds ratio in very large VLDL (%)	628	-0.10	-0.21	0.00	0.061	628	-0.19	-0.30	-0.07	1.83E-03	628	-0.06	-0.18	0.06	0.301
Total cholesterol to total lipids ratio in very large VLDL (%)	628	0.14	0.01	0.26	0.032	628	0.09	-0.05	0.23	0.223	628	-0.08	-0.21	0.06	0.261
Cholesterol esters to total lipids ratio in very large VLDL (%)	628	0.16	0.03	0.30	0.020	628	0.13	-0.02	0.29	0.098	628	-0.06	-0.21	0.08	0.395
Free cholesterol to total lipids ratio in very large VLDL (%)	628	0.11	-0.03	0.25	0.112	628	0.04	-0.13	0.20	0.666	628	-0.10	-0.25	0.04	0.163
Triglycerides to total lipids ratio in very large VLDL (%)	628	-0.13	-0.26	0.01	0.070	628	-0.04	-0.20	0.12	0.605	628	0.11	-0.03	0.26	0.131
Phospholipids to total lipds ratio in large VLDL (%)	628	-0.11	-0.24	0.01	0.079	628	-0.13	-0.27	0.01	0.075	628	0.03	-0.12	0.17	0.713
Total cholesterol to total lipids ratio in large VLDL (%)	628	-0.03	-0.13	0.08	0.622	628	-0.08	-0.21	0.04	0.190	628	-0.03	-0.15	0.09	0.584
Cholesterol esters to total lipids ratio in large VLDL (%)	628	0.03	-0.02	0.08	0.255	628	0.00	-0.06	0.06	0.919	628	-0.03	-0.09	0.03	0.351
Free cholesterol to total lipids ratio in large VLDL (%)	628	-0.11	-0.23	0.00	0.056	628	-0.15	-0.28	-0.03	0.019	628	-0.01	-0.15	0.13	0.920
Triglycerides to total lipids ratio in large VLDL (%)	628	0.01	-0.01	0.02	0.259	628	0.01	0.00	0.03	0.140	628	0.00	-0.02	0.02	0.937
Phospholipids to total lipds ratio in medium VLDL (%)	628	0.08	-0.04	0.19	0.201	628	0.10	-0.03	0.23	0.144	628	0.03	-0.11	0.16	0.691
Total cholesterol to total lipids ratio in medium VLDL (%)	628	0.09	0.00	0.18	0.055	628	0.06	-0.03	0.16	0.208	628	-0.02	-0.13	0.09	0.761
Cholesterol esters to total lipids ratio in medium VLDL (%)	628	0.12	0.02	0.22	0.016	628	0.10	-0.01	0.20	0.075	628	-0.02	-0.15	0.10	0.706
Free cholesterol to total lipids ratio in medium VLDL (%)	628	-0.06	-0.17	0.05	0.273	628	-0.08	-0.20	0.03	0.155	628	0.01	-0.12	0.13	0.909
Triglycerides to total lipids ratio in medium VLDL (%)	628	-0.10	-0.18	-0.01	0.034	628	-0.07	-0.16	0.01	0.102	628	0.01	-0.10	0.11	0.893
Phospholipids to total lipds ratio in small VLDL (%)	628	0.17	0.06	0.28	2.23E-03	628	0.17	0.05	0.29	0.006	628	-0.02	-0.16	0.12	0.771
Total cholesterol to total lipids ratio in small VLDL (%)	628	0.06	-0.04	0.17	0.249	628	0.07	-0.05	0.19	0.243	628	-0.01	-0.14	0.11	0.835
Cholesterol esters to total lipids ratio in small VLDL (%)	628	0.03	-0.07	0.14	0.544	628	0.04	-0.07	0.16	0.469	628	-0.01	-0.13	0.11	0.856
Free cholesterol to total lipids ratio in small VLDL (%)	628	0.21	0.10	0.32	2.39E-04	628	0.19	0.08	0.30	7.46E-04	628	-0.02	-0.16	0.11	0.727
Triglycerides to total lipids ratio in small VLDL (%)	628	-0.10	-0.22	0.01	0.068	628	-0.11	-0.23	0.02	0.093	628	0.02	-0.11	0.15	0.741
Phospholipids to total lipds ratio in very small VLDL (%)	628	-0.01	-0.12	0.09	0.802	628	-0.02	-0.13	0.09	0.762	628	0.01	-0.09	0.12	0.817
Total cholesterol to total lipids ratio in very small VLDL (%)	628	0.04	-0.07	0.16	0.461	628	0.09	-0.04	0.22	0.174	628	0.03	-0.11	0.17	0.633
Cholesterol esters to total lipids ratio in very small VLDL (%)	628	-0.01	-0.11	0.09	0.783	628	0.02	-0.09	0.12	0.770	628	0.00	-0.11	0.11	0.996
Free cholesterol to total lipids ratio in very small VLDL (%)	628	0.17	0.04	0.30	0.009	628	0.22	0.06	0.38	0.006	628	0.07	-0.11	0.25	0.461
Triglycerides to total lipids ratio in very small VLDL (%)	628	-0.07	-0.18	0.04	0.231	628	-0.09	-0.22	0.04	0.164	628	0.00	-0.14	0.13	0.943
Phospholipids to total lipds ratio in IDL (%)	628	-0.07	-0.21	0.06	0.289	628	-0.05	-0.19	0.08	0.451	628	-0.01	-0.17	0.15	0.932
Total cholesterol to total lipids ratio in IDL (%)	628	0.04	-0.07	0.16	0.426	628	0.01	-0.11	0.13	0.868	628	-0.05	-0.18	0.09	0.484
Cholesterol esters to total lipids ratio in IDL (%)	628	0.03	-0.09	0.15	0.592	628	-0.01	-0.14	0.11	0.819	628	-0.05	-0.20	0.09	0.449
Free cholesterol to total lipids ratio in IDL (%)	628	0.01	-0.10	0.12	0.863	628	0.06	-0.05	0.17	0.267	628	0.04	-0.06	0.15	0.436
Triglycerides to total lipids ratio in IDL (%)	628	-0.02	-0.12	0.08	0.715	628	0.00	-0.11	0.11	0.971	628	0.05	-0.07	0.17	0.433
Phospholipids to total lipds ratio in large LDL (%)	628	0.04	-0.04	0.13	0.327	628	0.01	-0.08	0.10	0.808	628	-0.07	-0.15	0.02	0.144
Total cholesterol to total lipids ratio in large LDL (%)	628	0.02	-0.06	0.10	0.676	628	0.00	-0.09	0.09	0.974	628	-0.02	-0.12	0.07	0.637
Cholesterol esters to total lipids ratio in large LDL (%)	628	-0.01	-0.09	0.07	0.810	628	-0.04	-0.12	0.05	0.381	628	-0.02	-0.11	0.07	0.652
Free cholesterol to total lipids ratio in large LDL (%)	628	0.05	-0.03	0.14	0.221	628	0.11	0.01	0.22	0.027	628	0.03	-0.06	0.13	0.492
Triglycerides to total lipids ratio in large LDL (%)	628	-0.04	-0.14	0.06	0.450	628	0.01	-0.10	0.12	0.882	628	0.08	-0.05	0.21	0.210
Phospholipids to total lipds ratio in medium LDL (%)	628	0.02	-0.02	0.06	0.380	628	0.01	-0.03	0.05	0.664	628	-0.02	-0.06	0.02	0.427
Total cholesterol to total lipids ratio in medium LDL (%)	628	-0.02	-0.11	0.08	0.709	628	-0.01	-0.11	0.09	0.856	628	0.01	-0.09	0.12	0.793
Cholesterol esters to total lipids ratio in medium LDL (%)	628	-0.04	-0.15	0.06	0.406	628	-0.03	-0.14	0.07	0.520	628	0.02	-0.08	0.13	0.648
Free cholesterol to total lipids ratio in medium LDL (%)	628	0.02	-0.01	0.05	0.245	628	0.02	-0.01	0.05	0.237	628	-0.01	-0.04	0.03	0.722
Triglycerides to total lipids ratio in medium LDL (%)	628	-0.03	-0.14	0.08	0.640	628	0.00	-0.12	0.11	0.942	628	0.04	-0.09	0.17	0.504
Phospholipids to total lipds ratio in small LDL (%)	628	0.03	-0.03	0.09	0.377	628	0.02	-0.05	0.08	0.578	628	-0.02	-0.08	0.04	0.546
Total cholesterol to total lipids ratio in small LDL (%)	628	-0.01	-0.11	0.09	0.793	628	0.00	-0.10	0.10	0.988	628	0.02	-0.09	0.12	0.745
Cholesterol esters to total lipids ratio in small LDL (%)	628	-0.05	-0.16	0.06	0.384	628	-0.04	-0.15	0.08	0.528	628	0.02	-0.09	0.14	0.688
Free cholesterol to total lipids ratio in small LDL (%)	628	0.04	-0.02	0.10	0.209	628	0.05	-0.02	0.12	0.155	628	0.00	-0.07	0.07	0.940
Triglycerides to total lipids ratio in small LDL (%)	628	-0.04	-0.15	0.07	0.480	628	-0.06	-0.18	0.05	0.297	628	0.00	-0.13	0.12	0.973
Phospholipids to total lipds ratio in very large HDL (%)	628	0.05	-0.04	0.14	0.306	628	0.11	0.01	0.21	0.028	628	0.07	-0.03	0.16	0.181
Total cholesterol to total lipids ratio in very large HDL (%)	628	-0.04	-0.13	0.05	0.397	628	-0.10	-0.19	0.00	0.055	628	-0.07	-0.16	0.02	0.151
Cholesterol esters to total lipids ratio in very large HDL (%)	628	-0.03	-0.12	0.06	0.520	628	-0.09	-0.19	0.01	0.072	628	-0.07	-0.17	0.02	0.123
Free cholesterol to total lipids ratio in very large HDL (%)	628	-0.05	-0.16	0.07	0.402	628	-0.03	-0.14	0.08	0.591	628	0.04	-0.10	0.17	0.601
Triglycerides to total lipids ratio in very large HDL (%)	628	-0.04	-0.15	0.07	0.444	628	-0.07	-0.18	0.03	0.172	628	0.00	-0.12	0.13	0.961
Phospholipids to total lipds ratio in large HDL (%)	628	-0.08	-0.18	0.03	0.141	628	-0.16	-0.27	-0.05	0.004	628	-0.12	-0.25	0.02	0.088
Total cholesterol to total lipids ratio in large HDL (%)	628	0.08	-0.02	0.18	0.126	628	0.16	0.05	0.26	0.004	628	0.09	-0.03	0.22	0.151
Cholesterol esters to total lipids ratio in large HDL (%)	628	0.08	-0.02	0.18	0.123	628	0.16	0.05	0.26	0.003	628	0.10	-0.03	0.22	0.133
Free cholesterol to total lipids ratio in large HDL (%)	628	0.06	-0.04	0.16	0.246	628	0.11	0.00	0.21	0.047	628	0.06	-0.06	0.18	0.339
Triglycerides to total lipids ratio in large HDL (%)	628	-0.05	-0.16	0.05	0.308	628	-0.11	-0.22	-0.01	0.038	628	-0.04	-0.16	0.08	0.510

Phospholipids to total lipds ratio in medium HDL (%)	628	0.08	-0.04	0.19	0.182	628	0.06	-0.06	0.19	0.309	628	0.01	-0.13	0.15	0.858
Total cholesterol to total lipids ratio in medium HDL (%)	628	-0.02	-0.13	0.09	0.759	628	0.03	-0.09	0.14	0.647	628	0.01	-0.12	0.14	0.825
Cholesterol esters to total lipids ratio in medium HDL (%)	628	-0.01	-0.12	0.10	0.877	628	0.03	-0.08	0.15	0.571	628	0.01	-0.13	0.14	0.938
Free cholesterol to total lipids ratio in medium HDL (%)	628	-0.04	-0.20	0.11	0.586	628	-0.01	-0.14	0.11	0.835	628	0.05	-0.13	0.24	0.577
Triglycerides to total lipids ratio in medium HDL (%)	628	-0.08	-0.18	0.03	0.141	628	-0.14	-0.25	-0.02	0.021	628	-0.05	-0.17	0.08	0.467
Phospholipids to total lipds ratio in small HDL (%)	628	-0.15	-0.24	-0.06	1.70E-03	628	-0.08	-0.18	0.02	0.097	628	0.06	-0.06	0.17	0.334
Total cholesterol to total lipids ratio in small HDL (%)	628	0.17	0.08	0.27	4.41E-04	628	0.12	0.01	0.22	0.030	628	-0.06	-0.18	0.06	0.329
Cholesterol esters to total lipids ratio in small HDL (%)	628	0.16	0.06	0.25	1.23E-03	628	0.09	-0.01	0.20	0.075	628	-0.07	-0.18	0.05	0.270
Free cholesterol to total lipids ratio in small HDL (%)	628	0.03	-0.08	0.13	0.615	628	0.11	0.00	0.21	0.056	628	0.08	-0.05	0.21	0.239
Triglycerides to total lipids ratio in small HDL (%)	628	-0.13	-0.23	-0.02	0.020	628	-0.14	-0.25	-0.02	0.021	628	0.03	-0.10	0.16	0.679
Mean diameter for VLDL particles (nm)	628	-0.12	-0.23	-0.02	0.026	628	-0.17	-0.29	-0.06	0.004	628	-0.04	-0.16	0.09	0.578
Mean diameter for LDL particles (nm)	628	0.06	-0.03	0.15	0.200	628	0.14	0.03	0.24	0.011	628	0.09	-0.03	0.21	0.158
Mean diameter for HDL particles (nm)	628	0.04	-0.05	0.13	0.400	628	0.10	0.00	0.21	0.043	628	0.08	-0.02	0.19	0.105
Serum total cholesterol (mmol/l)	628	-0.01	-0.09	0.07	0.754	628	-0.03	-0.12	0.06	0.523	628	0.00	-0.10	0.09	0.916
Total cholesterol in VLDL (mmol/l)	628	-0.05	-0.14	0.04	0.260	628	-0.12	-0.22	-0.03	0.014	628	-0.05	-0.16	0.05	0.312
Remnant cholesterol (non-HDL, non-LDL -cholesterol) (mmol/l)	628	-0.04	-0.12	0.04	0.329	628	-0.09	-0.18	0.00	0.039	628	-0.04	-0.14	0.06	0.403
Total cholesterol in LDL (mmol/l)	628	-0.05	-0.14	0.04	0.293	628	-0.06	-0.15	0.04	0.275	628	0.00	-0.10	0.10	0.989
Total cholesterol in HDL (mmol/l)	628	0.09	0.00	0.17	0.057	628	0.10	0.00	0.19	0.049	628	0.02	-0.09	0.12	0.767
Total cholesterol in HDL2 (mmol/l)	628	0.09	0.00	0.18	0.057	628	0.11	0.01	0.21	0.032	628	0.02	-0.09	0.13	0.727
Total cholesterol in HDL3 (mmol/l)	628	0.08	-0.01	0.16	0.070	628	0.07	-0.02	0.16	0.138	628	0.01	-0.10	0.11	0.915
Esterified cholesterol (mmol/l)	628	-0.02	-0.10	0.06	0.626	628	-0.03	-0.12	0.06	0.492	628	0.00	-0.09	0.09	0.980
Free cholesterol (mmol/l)	628	0.01	-0.07	0.09	0.855	628	-0.02	-0.12	0.07	0.605	628	-0.02	-0.11	0.08	0.695
Serum total triglycerides (mmol/l)	628	-0.09	-0.20	0.01	0.066	628	-0.15	-0.27	-0.04	0.009	628	-0.03	-0.15	0.09	0.611
Triglycerides in VLDL (mmol/l)	628	-0.10	-0.20	0.00	0.054	628	-0.16	-0.27	-0.04	0.006	628	-0.03	-0.15	0.08	0.580
Triglycerides in LDL (mmol/l)	628	-0.04	-0.15	0.06	0.428	628	-0.08	-0.20	0.04	0.201	628	-0.02	-0.15	0.11	0.758
Triglycerides in HDL (mmol/l)	628	-0.04	-0.13	0.06	0.474	628	-0.07	-0.18	0.03	0.172	628	-0.01	-0.13	0.11	0.855
Diacylglycerol (mmol/l)	594	0.02	-0.09	0.13	0.711	594	0.04	-0.07	0.15	0.505	594	0.05	-0.07	0.18	0.415
Ratio of diacylglycerol to triglycerides	594	0.02	-0.10	0.15	0.700	594	0.07	-0.06	0.20	0.283	594	0.08	-0.06	0.23	0.273
Total phosphoglycerides (mmol/l)	628	0.05	-0.05	0.15	0.307	628	0.00	-0.10	0.10	0.941	628	-0.03	-0.14	0.08	0.555
Ratio of triglycerides to phosphoglycerides	628	-0.11	-0.22	0.00	0.060	628	-0.13	-0.24	-0.02	0.019	628	0.01	-0.10	0.12	0.850
Phosphatidylcholine and other cholines (mmol/l)	616	0.05	-0.05	0.14	0.333	616	0.02	-0.08	0.13	0.638	616	-0.01	-0.12	0.10	0.821
Total cholines (mmol/l)	626	0.04	-0.05	0.14	0.378	626	0.00	-0.10	0.10	0.983	626	-0.02	-0.13	0.08	0.650
Apolipoprotein A-I (g/l)	628	0.05	-0.03	0.13	0.232	628	0.04	-0.05	0.14	0.338	628	0.01	-0.09	0.10	0.920
Apolipoprotein B (g/l)	628	-0.08	-0.17	0.01	0.072	628	-0.13	-0.24	-0.03	0.014	628	-0.03	-0.14	0.08	0.582
Ratio of apolipoprotein B to apolipoprotein A-I	628	-0.11	-0.20	-0.01	0.025	628	-0.16	-0.27	-0.05	0.003	628	-0.03	-0.15	0.08	0.554
Total fatty acids (mmol/l)	628	-0.02	-0.11	0.07	0.637	628	-0.07	-0.17	0.04	0.209	628	-0.02	-0.13	0.08	0.653
Estimated description of fatty acid chain length, not actual carbon number	625	-0.03	-0.15	0.09	0.648	625	0.00	-0.13	0.13	0.959	625	0.05	-0.07	0.18	0.399
Estimated degree of unsaturation	627	0.00	-0.12	0.12	0.988	627	0.02	-0.11	0.16	0.726	627	0.03	-0.10	0.17	0.640
22:6, docosahexaenoic acid (mmol/l)	628	0.02	-0.08	0.13	0.651	628	0.00	-0.11	0.11	0.992	628	-0.03	-0.15	0.09	0.616
18:2, linoleic acid (mmol/l)	626	-0.04	-0.13	0.05	0.361	626	-0.07	-0.17	0.03	0.169	626	0.00	-0.10	0.10	0.972
Conjugated linoleic acid (mmol/l)	627	-0.12	-0.25	0.01	0.072	627	-0.09	-0.22	0.04	0.159	627	0.06	-0.09	0.22	0.406
Omega-3 fatty acids (mmol/l)	626	-0.08	-0.19	0.03	0.158	626	-0.07	-0.19	0.04	0.220	626	0.00	-0.13	0.13	0.991
Omega-6 fatty acids (mmol/l)	628	-0.04	-0.13	0.05	0.421	628	-0.06	-0.16	0.04	0.244	628	0.01	-0.09	0.11	0.817
Polyunsaturated fatty acids (mmol/l)	626	-0.04	-0.13	0.05	0.381	626	-0.06	-0.16	0.04	0.264	626	0.01	-0.09	0.12	0.800
Monounsaturated fatty acids; 16:1, 18:1 (mmol/l)	627	-0.01	-0.10	0.09	0.868	627	-0.08	-0.18	0.03	0.152	627	-0.05	-0.16	0.06	0.383
Saturated fatty acids (mmol/l)	626	0.00	-0.10	0.10	0.962	626	-0.04	-0.17	0.09	0.581	626	-0.03	-0.15	0.08	0.555
Ratio of 22:6 docosahexaenoic acid to total fatty acids (%)	628	0.06	-0.06	0.19	0.302	628	0.06	-0.07	0.19	0.378	628	-0.02	-0.16	0.11	0.713
Ratio of 18:2 linoleic acid to total fatty acids (%)	626	-0.02	-0.12	0.09	0.768	626	0.01	-0.11	0.14	0.824	626	0.04	-0.08	0.16	0.524
Ratio of conjugated linoleic acid to total fatty acids (%)	627	-0.13	-0.27	0.00	0.051	627	-0.09	-0.22	0.03	0.150	627	0.08	-0.07	0.24	0.305
Ratio of omega-3 fatty acids to total fatty acids (%)	626	-0.07	-0.19	0.05	0.284	626	-0.01	-0.15	0.12	0.827	626	0.03	-0.11	0.17	0.675
Ratio of omega-6 fatty acids to total fatty acids (%)	628	-0.01	-0.12	0.10	0.925	628	0.04	-0.09	0.16	0.585	628	0.07	-0.06	0.19	0.309
Ratio of polyunsaturated fatty acids to total fatty acids (%)	626	-0.01	-0.12	0.10	0.829	626	0.04	-0.09	0.16	0.567	626	0.07	-0.06	0.19	0.315
Ratio of monounsaturated fatty acids to total fatty acids (%)	627	0.01	-0.11	0.13	0.837	627	-0.03	-0.17	0.10	0.634	627	-0.04	-0.17	0.09	0.574
Ratio of saturated fatty acids to total fatty acids (%)	626	0.00	-0.13	0.13	0.992	626	0.01	-0.15	0.17	0.896	626	-0.03	-0.16	0.11	0.709
Glucose (mmol/l)	622	-0.05	-0.16	0.05	0.311	622	-0.09	-0.19	0.02	0.109	622	-0.04	-0.17	0.09	0.533
Lactate (mmol/l)	627	-0.05	-0.17	0.07	0.405	627	-0.05	-0.17	0.06	0.376	627	0.02	-0.12	0.16	0.777
Pyruvate (mmol/l)	626	-0.14	-0.26	-0.03	0.013	626	-0.12	-0.24	0.00	0.047	626	0.06	-0.07	0.19	0.350

Citrate (mmol/l)	624	0.14	0.02	0.27	0.022	624	0.05	-0.08	0.18	0.445	624	-0.11	-0.25	0.02	0.108
Alanine (mmol/l)	628	-0.15	-0.27	-0.04	0.010	628	-0.08	-0.20	0.03	0.147	628	0.12	-0.02	0.26	0.093
Glutamine (mmol/l)	628	0.07	-0.03	0.16	0.175	628	0.03	-0.07	0.13	0.577	628	-0.05	-0.16	0.07	0.400
Histidine (mmol/l)	592	0.08	-0.04	0.19	0.192	592	0.14	0.02	0.27	0.022	592	0.10	-0.04	0.23	0.153
Isoleucine (mmol/l)	628	-0.04	-0.15	0.06	0.411	628	-0.04	-0.15	0.07	0.494	628	0.04	-0.08	0.17	0.501
Leucine (mmol/l)	628	0.05	-0.05	0.15	0.305	628	0.06	-0.05	0.16	0.275	628	0.03	-0.08	0.15	0.562
Valine (mmol/l)	628	0.02	-0.08	0.12	0.718	628	-0.01	-0.13	0.11	0.903	628	-0.01	-0.14	0.11	0.850
Phenylalanine (mmol/l)	627	0.09	-0.03	0.21	0.126	627	0.13	0.01	0.25	0.030	627	0.06	-0.07	0.19	0.375
Tyrosine (mmol/l)	623	0.04	-0.08	0.16	0.498	623	0.01	-0.12	0.13	0.922	623	-0.02	-0.16	0.12	0.802
Acetate (mmol/l)	628	0.09	-0.02	0.20	0.102	628	0.11	-0.01	0.23	0.076	628	0.05	-0.08	0.18	0.484
Acetoacetate (mmol/l)	628	0.01	-0.09	0.12	0.848	628	0.02	-0.09	0.12	0.730	628	0.04	-0.10	0.18	0.566
3-hydroxybutyrate (mmol/l)	627	-0.03	-0.14	0.08	0.559	627	-0.07	-0.18	0.04	0.223	627	-0.04	-0.17	0.08	0.483
Creatinine (mmol/l)	628	-0.08	-0.18	0.01	0.092	628	-0.02	-0.13	0.09	0.705	628	0.09	-0.03	0.20	0.135
Albumin (signal area)	628	-0.16	-0.29	-0.04	0.011	628	-0.09	-0.22	0.04	0.186	628	0.06	-0.07	0.20	0.360
Glycoprotein acetyls, mainly a1-acid glycoprotein (mmol/l)	628	-0.12	-0.22	-0.02	0.024	628	-0.15	-0.25	-0.05	0.003	628	-0.01	-0.12	0.10	0.869

Complete case sample

	**Mean of CPM at age 12y, 14y, 15y (per SD higher)** *Adj. for age, sex, ethnicity, maternal education, mean wear time, wear month, mean FMI, metabolic trait at 8y*					**Mean of MVPA at age 12y, 14y, 15y (per SD higher)** *Adj. for age, sex, ethnicity, maternal education, mean wear time, wear month, mean FMI, metabolic trait at 8y*					**Mean of SED at age 12y, 14y, 15y (per SD higher)** *Adj. for age, sex, ethnicity, maternal education, mean wear time, wear month, mean FMI, metabolic trait at 8y*				
**Standardised outcome at age 15y**	**N**	**Beta**	**LCL**	**UCL**	**P-value**	**N**	**Beta**	**LCL**	**UCL**	**P-value**	**N**	**Beta**	**LCL**	**UCL**	**P-value**
Systolic blood pressure (mmHg)	520	-0.07	-0.19	0.05	0.275	520	-0.10	-0.23	0.03	0.147	520	-0.04	-0.18	0.10	0.610
Diastolic blood pressure (mmHg)	520	0.03	-0.10	0.15	0.685	520	0.07	-0.06	0.21	0.277	520	0.01	-0.14	0.17	0.852
Concentration of chylomicrons and extremely large VLDL particles (mol/l)	520	-0.06	-0.17	0.06	0.316	520	-0.13	-0.25	-0.01	0.030	520	-0.10	-0.23	0.03	0.139
Total lipids in chylomicrons and extremely large VLDL (mmol/l)	520	-0.05	-0.17	0.06	0.354	520	-0.12	-0.24	-0.01	0.038	520	-0.10	-0.23	0.03	0.146
Phospholipids in chylomicrons and extremely large VLDL (mmol/l)	520	-0.06	-0.18	0.06	0.304	520	-0.13	-0.26	-0.01	0.030	520	-0.10	-0.24	0.03	0.135
Total cholesterol in chylomicrons and extremely large VLDL (mmol/l)	520	-0.03	-0.13	0.08	0.627	520	-0.09	-0.20	0.02	0.101	520	-0.08	-0.20	0.04	0.179
Cholesterol esters in chylomicrons and extremely large VLDL (mmol/l)	520	0.01	-0.10	0.11	0.899	520	-0.05	-0.15	0.05	0.336	520	-0.07	-0.18	0.05	0.256
Free cholesterol in chylomicrons and extremely large VLDL (mmol/l)	520	-0.06	-0.17	0.06	0.318	520	-0.13	-0.25	-0.01	0.037	520	-0.10	-0.23	0.04	0.160
Triglycerides in chylomicrons and extremely large VLDL (mmol/l)	520	-0.06	-0.17	0.06	0.321	520	-0.13	-0.25	-0.01	0.033	520	-0.10	-0.23	0.03	0.144
Concentration of very large VLDL particles (mol/l)	520	-0.03	-0.15	0.08	0.554	520	-0.10	-0.22	0.01	0.088	520	-0.09	-0.22	0.04	0.168
Total lipids in very large VLDL (mmol/l)	520	-0.03	-0.14	0.08	0.598	520	-0.10	-0.21	0.02	0.099	520	-0.09	-0.21	0.04	0.168
Phospholipids in very large VLDL (mmol/l)	520	-0.04	-0.15	0.07	0.475	520	-0.11	-0.23	0.01	0.073	520	-0.09	-0.22	0.04	0.171
Total cholesterol in very large VLDL (mmol/l)	520	-0.03	-0.14	0.08	0.576	520	-0.10	-0.21	0.01	0.076	520	-0.09	-0.21	0.03	0.150
Cholesterol esters in very large VLDL (mmol/l)	520	-0.01	-0.12	0.09	0.783	520	-0.08	-0.19	0.02	0.123	520	-0.09	-0.20	0.03	0.156
Free cholesterol in very large VLDL (mmol/l)	520	-0.05	-0.16	0.06	0.407	520	-0.12	-0.23	0.00	0.048	520	-0.10	-0.23	0.03	0.148
Triglycerides in very large VLDL (mmol/l)	520	-0.03	-0.14	0.09	0.642	520	-0.09	-0.21	0.02	0.117	520	-0.09	-0.21	0.04	0.176
Concentration of large VLDL particles (mol/l)	520	-0.01	-0.12	0.10	0.902	520	-0.08	-0.19	0.04	0.199	520	-0.08	-0.20	0.04	0.190
Total lipids in large VLDL (mmol/l)	520	0.00	-0.11	0.11	0.946	520	-0.07	-0.19	0.04	0.210	520	-0.08	-0.20	0.04	0.182
Phospholipids in large VLDL (mmol/l)	520	-0.01	-0.11	0.10	0.921	520	-0.08	-0.19	0.04	0.188	520	-0.09	-0.21	0.04	0.167
Total cholesterol in large VLDL (mmol/l)	520	0.01	-0.10	0.12	0.828	520	-0.06	-0.18	0.05	0.261	520	-0.09	-0.21	0.03	0.140
Cholesterol esters in large VLDL (mmol/l)	520	0.03	-0.07	0.14	0.543	520	-0.05	-0.15	0.06	0.406	520	-0.09	-0.21	0.03	0.129
Free cholesterol in large VLDL (mmol/l)	520	-0.01	-0.12	0.10	0.887	520	-0.08	-0.19	0.03	0.172	520	-0.09	-0.21	0.03	0.158
Triglycerides in large VLDL (mmol/l)	520	-0.01	-0.12	0.10	0.868	520	-0.07	-0.19	0.04	0.202	520	-0.08	-0.20	0.04	0.208
Concentration of medium VLDL particles (mol/l)	520	0.01	-0.10	0.12	0.911	520	-0.07	-0.19	0.04	0.223	520	-0.09	-0.21	0.03	0.143
Total lipids in medium VLDL (mmol/l)	520	0.02	-0.09	0.12	0.783	520	-0.06	-0.18	0.05	0.266	520	-0.09	-0.21	0.03	0.134
Phospholipids in medium VLDL (mmol/l)	520	0.02	-0.09	0.13	0.761	520	-0.07	-0.18	0.05	0.251	520	-0.10	-0.22	0.02	0.113
Total cholesterol in medium VLDL (mmol/l)	520	0.05	-0.05	0.16	0.310	520	-0.03	-0.14	0.08	0.571	520	-0.09	-0.21	0.02	0.120
Cholesterol esters in medium VLDL (mmol/l)	520	0.09	-0.02	0.19	0.109	520	0.00	-0.10	0.11	0.956	520	-0.09	-0.21	0.03	0.158
Free cholesterol in medium VLDL (mmol/l)	520	0.02	-0.09	0.12	0.784	520	-0.07	-0.18	0.05	0.254	520	-0.10	-0.22	0.03	0.124
Triglycerides in medium VLDL (mmol/l)	520	0.00	-0.11	0.10	0.932	520	-0.08	-0.19	0.04	0.191	520	-0.09	-0.21	0.04	0.168
Concentration of small VLDL particles (mol/l)	520	0.03	-0.08	0.14	0.587	520	-0.07	-0.19	0.05	0.254	520	-0.11	-0.24	0.01	0.078

Total lipids in small VLDL (mmol/l)	520	0.03	-0.07	0.14	0.527	520	-0.07	-0.18	0.05	0.235	520	-0.12	-0.24	0.00	0.059
Phospholipids in small VLDL (mmol/l)	520	0.07	-0.04	0.17	0.215	520	-0.04	-0.16	0.07	0.452	520	-0.12	-0.24	0.00	0.048
Total cholesterol in small VLDL (mmol/l)	520	0.04	-0.06	0.13	0.448	520	-0.06	-0.16	0.04	0.256	520	-0.11	-0.23	0.00	0.055
Cholesterol esters in small VLDL (mmol/l)	520	0.02	-0.07	0.12	0.597	520	-0.06	-0.16	0.03	0.205	520	-0.11	-0.22	0.01	0.068
Free cholesterol in small VLDL (mmol/l)	520	0.06	-0.04	0.16	0.235	520	-0.04	-0.15	0.07	0.447	520	-0.12	-0.24	0.00	0.053
Triglycerides in small VLDL (mmol/l)	520	0.02	-0.10	0.13	0.787	520	-0.07	-0.20	0.05	0.253	520	-0.10	-0.23	0.03	0.132
Concentration of very small VLDL particles (mol/l)	520	0.06	-0.03	0.15	0.159	520	0.00	-0.09	0.09	0.978	520	-0.06	-0.17	0.05	0.287
Total lipids in very small VLDL (mmol/l)	520	0.04	-0.04	0.13	0.330	520	-0.02	-0.11	0.07	0.696	520	-0.06	-0.17	0.04	0.243
Phospholipids in very small VLDL (mmol/l)	520	0.02	-0.06	0.11	0.603	520	-0.02	-0.11	0.07	0.659	520	-0.04	-0.14	0.06	0.423
Total cholesterol in very small VLDL (mmol/l)	520	0.07	-0.04	0.17	0.203	520	0.01	-0.10	0.11	0.900	520	-0.06	-0.17	0.06	0.318
Cholesterol esters in very small VLDL (mmol/l)	520	0.04	-0.06	0.13	0.474	520	-0.02	-0.12	0.07	0.627	520	-0.07	-0.18	0.04	0.232
Free cholesterol in very small VLDL (mmol/l)	520	0.12	0.01	0.23	0.032	520	0.07	-0.05	0.19	0.241	520	-0.03	-0.17	0.10	0.620
Triglycerides in very small VLDL (mmol/l)	520	0.02	-0.09	0.13	0.700	520	-0.06	-0.19	0.06	0.326	520	-0.09	-0.22	0.04	0.170
Concentration of IDL particles (mol/l)	520	-0.02	-0.12	0.08	0.711	520	-0.04	-0.15	0.07	0.450	520	-0.03	-0.14	0.09	0.638
Total lipids in IDL (mmol/l)	520	0.00	-0.09	0.09	0.944	520	-0.02	-0.12	0.07	0.627	520	-0.03	-0.13	0.08	0.577
Phospholipids in IDL (mmol/l)	520	-0.02	-0.13	0.08	0.693	520	-0.04	-0.16	0.07	0.463	520	-0.03	-0.14	0.09	0.643
Total cholesterol in IDL (mmol/l)	520	0.02	-0.06	0.11	0.619	520	-0.02	-0.11	0.08	0.745	520	-0.04	-0.14	0.06	0.436
Cholesterol esters in IDL (mmol/l)	520	0.03	-0.05	0.12	0.420	520	-0.02	-0.11	0.07	0.740	520	-0.06	-0.16	0.05	0.293
Free cholesterol in IDL (mmol/l)	520	-0.01	-0.10	0.09	0.901	520	-0.02	-0.12	0.09	0.735	520	-0.01	-0.12	0.09	0.809
Triglycerides in IDL (mmol/l)	520	0.00	-0.11	0.11	0.945	520	-0.04	-0.16	0.08	0.519	520	-0.03	-0.16	0.09	0.597
Concentration of large LDL particles (mol/l)	520	-0.02	-0.13	0.09	0.716	520	-0.06	-0.18	0.06	0.328	520	-0.05	-0.17	0.07	0.391
Total lipids in large LDL (mmol/l)	520	-0.01	-0.11	0.09	0.843	520	-0.04	-0.15	0.07	0.454	520	-0.04	-0.15	0.07	0.515
Phospholipids in large LDL (mmol/l)	520	0.00	-0.10	0.10	0.969	520	-0.04	-0.15	0.06	0.424	520	-0.05	-0.16	0.06	0.384
Total cholesterol in large LDL (mmol/l)	520	-0.01	-0.10	0.09	0.908	520	-0.04	-0.14	0.07	0.491	520	-0.04	-0.15	0.07	0.502
Cholesterol esters in large LDL (mmol/l)	520	-0.01	-0.10	0.09	0.903	520	-0.04	-0.14	0.06	0.453	520	-0.04	-0.15	0.07	0.462
Free cholesterol in large LDL (mmol/l)	520	0.00	-0.10	0.09	0.933	520	-0.03	-0.13	0.08	0.604	520	-0.03	-0.14	0.08	0.613
Triglycerides in large LDL (mmol/l)	520	-0.03	-0.14	0.09	0.676	520	-0.06	-0.19	0.07	0.374	520	-0.04	-0.17	0.10	0.593
Concentration of medium LDL particles (mol/l)	520	-0.03	-0.15	0.09	0.640	520	-0.08	-0.21	0.05	0.214	520	-0.08	-0.21	0.06	0.259
Total lipids in medium LDL (mmol/l)	520	-0.02	-0.12	0.09	0.757	520	-0.06	-0.17	0.06	0.331	520	-0.05	-0.17	0.06	0.386
Phospholipids in medium LDL (mmol/l)	520	0.02	-0.07	0.11	0.612	520	-0.04	-0.14	0.06	0.442	520	-0.07	-0.18	0.03	0.176
Total cholesterol in medium LDL (mmol/l)	520	-0.02	-0.12	0.09	0.719	520	-0.06	-0.17	0.06	0.340	520	-0.05	-0.16	0.07	0.422
Cholesterol esters in medium LDL (mmol/l)	520	-0.03	-0.14	0.08	0.610	520	-0.06	-0.18	0.06	0.295	520	-0.05	-0.17	0.07	0.445
Free cholesterol in medium LDL (mmol/l)	520	0.03	-0.06	0.12	0.541	520	-0.02	-0.12	0.08	0.655	520	-0.06	-0.17	0.04	0.256
Triglycerides in medium LDL (mmol/l)	520	-0.03	-0.16	0.10	0.678	520	-0.09	-0.23	0.05	0.211	520	-0.08	-0.23	0.07	0.285
Concentration of small LDL particles (mol/l)	520	-0.02	-0.14	0.09	0.697	520	-0.08	-0.21	0.05	0.215	520	-0.08	-0.21	0.05	0.215
Total lipids in small LDL (mmol/l)	520	-0.02	-0.12	0.09	0.757	520	-0.06	-0.18	0.06	0.306	520	-0.06	-0.18	0.06	0.334
Phospholipids in small LDL (mmol/l)	520	0.02	-0.08	0.11	0.705	520	-0.04	-0.15	0.06	0.422	520	-0.07	-0.18	0.03	0.173
Total cholesterol in small LDL (mmol/l)	520	-0.02	-0.13	0.09	0.708	520	-0.06	-0.17	0.06	0.325	520	-0.05	-0.17	0.07	0.397
Cholesterol esters in small LDL (mmol/l)	520	-0.03	-0.15	0.08	0.555	520	-0.07	-0.20	0.05	0.249	520	-0.06	-0.18	0.07	0.397
Free cholesterol in small LDL (mmol/l)	520	0.07	-0.02	0.15	0.135	520	0.00	-0.09	0.10	0.969	520	-0.07	-0.17	0.03	0.165
Triglycerides in small LDL (mmol/l)	520	-0.02	-0.14	0.11	0.768	520	-0.11	-0.25	0.04	0.139	520	-0.11	-0.27	0.04	0.136
Concentration of very large HDL particles (mol/l)	520	-0.02	-0.13	0.10	0.788	520	0.05	-0.07	0.17	0.386	520	0.09	-0.02	0.20	0.114
Total lipids in very large HDL (mmol/l)	520	-0.02	-0.13	0.10	0.770	520	0.05	-0.07	0.16	0.455	520	0.08	-0.03	0.19	0.168
Phospholipids in very large HDL (mmol/l)	520	-0.01	-0.12	0.09	0.818	520	0.06	-0.05	0.17	0.299	520	0.10	-0.01	0.21	0.077
Total cholesterol in very large HDL (mmol/l)	520	-0.02	-0.14	0.10	0.729	520	0.02	-0.10	0.15	0.713	520	0.05	-0.07	0.17	0.409
Cholesterol esters in very large HDL (mmol/l)	520	-0.02	-0.15	0.10	0.710	520	0.02	-0.11	0.15	0.803	520	0.04	-0.08	0.16	0.512
Free cholesterol in very large HDL (mmol/l)	520	-0.02	-0.14	0.10	0.779	520	0.04	-0.08	0.16	0.519	520	0.07	-0.04	0.19	0.218
Triglycerides in very large HDL (mmol/l)	520	0.00	-0.11	0.11	0.958	520	0.04	-0.08	0.16	0.523	520	0.05	-0.07	0.18	0.393
Concentration of large HDL particles (mol/l)	520	0.04	-0.06	0.13	0.480	520	0.10	0.00	0.21	0.048	520	0.10	-0.01	0.20	0.087
Total lipids in large HDL (mmol/l)	520	0.03	-0.06	0.13	0.479	520	0.10	0.00	0.20	0.051	520	0.09	-0.01	0.20	0.088
Phospholipids in large HDL (mmol/l)	520	0.03	-0.07	0.12	0.577	520	0.09	-0.01	0.19	0.085	520	0.08	-0.02	0.19	0.122
Total cholesterol in large HDL (mmol/l)	520	0.04	-0.06	0.14	0.423	520	0.11	0.01	0.21	0.039	520	0.10	-0.01	0.21	0.078
Cholesterol esters in large HDL (mmol/l)	520	0.04	-0.06	0.14	0.425	520	0.11	0.01	0.21	0.036	520	0.10	-0.01	0.21	0.074
Free cholesterol in large HDL (mmol/l)	520	0.04	-0.06	0.14	0.417	520	0.10	0.00	0.21	0.050	520	0.09	-0.02	0.20	0.100
Triglycerides in large HDL (mmol/l)	520	0.06	-0.04	0.17	0.249	520	0.09	-0.02	0.21	0.108	520	0.05	-0.07	0.17	0.406
Concentration of medium HDL particles (mol/l)	520	0.12	0.02	0.23	0.024	520	0.09	-0.03	0.20	0.139	520	-0.05	-0.17	0.08	0.453
Total lipids in medium HDL (mmol/l)	520	0.11	0.01	0.22	0.036	520	0.08	-0.03	0.20	0.152	520	-0.04	-0.17	0.08	0.486

Phospholipids in medium HDL (mmol/l)	520	0.12	0.01	0.22	0.034	520	0.08	-0.03	0.20	0.148	520	-0.04	-0.16	0.09	0.543
Total cholesterol in medium HDL (mmol/l)	520	0.10	-0.01	0.21	0.062	520	0.09	-0.03	0.20	0.132	520	-0.03	-0.15	0.09	0.632
Cholesterol esters in medium HDL (mmol/l)	520	0.10	-0.01	0.21	0.071	520	0.09	-0.03	0.20	0.128	520	-0.03	-0.15	0.10	0.642
Free cholesterol in medium HDL (mmol/l)	520	0.11	0.00	0.21	0.047	520	0.08	-0.04	0.20	0.176	520	-0.03	-0.15	0.09	0.612
Triglycerides in medium HDL (mmol/l)	520	0.05	-0.06	0.16	0.335	520	-0.03	-0.15	0.10	0.693	520	-0.10	-0.23	0.03	0.123
Concentration of small HDL particles (mol/l)	520	0.06	-0.05	0.17	0.316	520	-0.04	-0.16	0.09	0.572	520	-0.13	-0.25	-0.01	0.040
Total lipids in small HDL (mmol/l)	520	0.10	-0.01	0.21	0.079	520	0.00	-0.11	0.12	0.957	520	-0.12	-0.24	0.00	0.048
Phospholipids in small HDL (mmol/l)	520	0.03	-0.08	0.14	0.613	520	-0.02	-0.15	0.10	0.695	520	-0.09	-0.21	0.03	0.155
Total cholesterol in small HDL (mmol/l)	520	0.14	0.04	0.25	0.008	520	0.05	-0.06	0.16	0.386	520	-0.10	-0.21	0.02	0.108
Cholesterol esters in small HDL (mmol/l)	520	0.14	0.03	0.24	0.010	520	0.04	-0.07	0.15	0.438	520	-0.09	-0.21	0.03	0.131
Free cholesterol in small HDL (mmol/l)	520	0.10	-0.01	0.20	0.072	520	0.06	-0.06	0.17	0.338	520	-0.06	-0.19	0.06	0.315
Triglycerides in small HDL (mmol/l)	520	-0.01	-0.12	0.11	0.906	520	-0.07	-0.20	0.05	0.265	520	-0.07	-0.21	0.06	0.282
Phospholipids to total lipds ratio in chylomicrons and extremely large VLDL (%)	520	0.00	-0.11	0.12	0.989	520	-0.07	-0.19	0.06	0.290	520	-0.08	-0.20	0.04	0.198
Total cholesterol to total lipids ratio in chylomicrons and extremely large VLDL (%)	520	0.11	-0.02	0.23	0.097	520	0.02	-0.12	0.15	0.821	520	-0.10	-0.23	0.03	0.133
Cholesterol esters to total lipids ratio in chylomicrons and extremely large VLDL (%)	520	0.12	-0.01	0.25	0.070	520	0.04	-0.09	0.17	0.554	520	-0.09	-0.22	0.05	0.201
Free cholesterol to total lipids ratio in chylomicrons and extremely large VLDL (%)	520	0.03	-0.09	0.15	0.645	520	-0.06	-0.19	0.07	0.363	520	-0.10	-0.23	0.04	0.161
Triglycerides to total lipids ratio in chylomicrons and extremely large VLDL (%)	520	-0.07	-0.15	0.02	0.112	520	0.00	-0.09	0.09	0.953	520	0.08	-0.01	0.17	0.078
Phospholipids to total lipds ratio in very large VLDL (%)	520	0.01	-0.11	0.13	0.892	520	-0.12	-0.24	0.01	0.074	520	-0.14	-0.26	-0.01	0.032
Total cholesterol to total lipids ratio in very large VLDL (%)	520	0.09	-0.05	0.23	0.202	520	0.06	-0.11	0.23	0.475	520	-0.04	-0.19	0.10	0.552
Cholesterol esters to total lipids ratio in very large VLDL (%)	520	0.10	-0.05	0.24	0.211	520	0.09	-0.10	0.27	0.344	520	-0.02	-0.17	0.14	0.813
Free cholesterol to total lipids ratio in very large VLDL (%)	520	0.10	-0.06	0.26	0.242	520	0.03	-0.16	0.21	0.781	520	-0.09	-0.25	0.07	0.277
Triglycerides to total lipids ratio in very large VLDL (%)	520	-0.11	-0.27	0.05	0.167	520	-0.03	-0.22	0.16	0.752	520	0.10	-0.06	0.26	0.209
Phospholipids to total lipds ratio in large VLDL (%)	520	0.00	-0.14	0.14	0.997	520	-0.07	-0.22	0.09	0.383	520	-0.06	-0.21	0.09	0.416
Total cholesterol to total lipids ratio in large VLDL (%)	520	0.07	-0.05	0.19	0.234	520	-0.04	-0.17	0.10	0.600	520	-0.11	-0.23	0.02	0.092
Cholesterol esters to total lipids ratio in large VLDL (%)	520	0.06	0.00	0.11	0.050	520	0.00	-0.06	0.06	0.982	520	-0.05	-0.12	0.01	0.117
Free cholesterol to total lipids ratio in large VLDL (%)	520	0.00	-0.13	0.12	0.965	520	-0.09	-0.23	0.05	0.203	520	-0.09	-0.24	0.06	0.223
Triglycerides to total lipids ratio in large VLDL (%)	520	-0.01	-0.02	0.01	0.491	520	0.01	-0.01	0.02	0.593	520	0.01	-0.01	0.03	0.258
Phospholipids to total lipds ratio in medium VLDL (%)	520	0.07	-0.06	0.21	0.298	520	0.08	-0.07	0.23	0.292	520	0.01	-0.14	0.15	0.906
Total cholesterol to total lipids ratio in medium VLDL (%)	520	0.15	0.05	0.25	0.004	520	0.09	-0.01	0.19	0.082	520	-0.05	-0.16	0.07	0.431
Cholesterol esters to total lipids ratio in medium VLDL (%)	520	0.17	0.05	0.28	0.004	520	0.11	0.00	0.23	0.055	520	-0.04	-0.17	0.09	0.537
Free cholesterol to total lipids ratio in medium VLDL (%)	520	0.03	-0.09	0.15	0.627	520	-0.04	-0.17	0.09	0.538	520	-0.08	-0.21	0.05	0.249
Triglycerides to total lipids ratio in medium VLDL (%)	520	-0.15	-0.25	-0.05	2.93E-03	520	-0.10	-0.20	0.00	0.041	520	0.03	-0.08	0.15	0.548
Phospholipids to total lipds ratio in small VLDL (%)	520	0.09	-0.03	0.21	0.150	520	0.14	0.01	0.26	0.036	520	0.07	-0.07	0.20	0.326
Total cholesterol to total lipids ratio in small VLDL (%)	520	0.04	-0.07	0.16	0.466	520	0.03	-0.10	0.17	0.616	520	-0.02	-0.15	0.12	0.816
Cholesterol esters to total lipids ratio in small VLDL (%)	520	0.02	-0.10	0.13	0.775	520	0.01	-0.12	0.14	0.913	520	-0.02	-0.15	0.11	0.791
Free cholesterol to total lipids ratio in small VLDL (%)	520	0.18	0.06	0.31	0.005	520	0.17	0.05	0.30	0.005	520	0.00	-0.15	0.15	0.994
Triglycerides to total lipids ratio in small VLDL (%)	520	-0.07	-0.19	0.06	0.302	520	-0.07	-0.21	0.08	0.372	520	0.00	-0.14	0.14	0.976
Phospholipids to total lipds ratio in very small VLDL (%)	520	0.00	-0.12	0.11	0.948	520	-0.02	-0.15	0.10	0.717	520	-0.01	-0.13	0.11	0.848
Total cholesterol to total lipids ratio in very small VLDL (%)	520	0.01	-0.12	0.14	0.882	520	0.07	-0.08	0.22	0.354	520	0.08	-0.07	0.23	0.305
Cholesterol esters to total lipids ratio in very small VLDL (%)	520	-0.05	-0.15	0.06	0.409	520	-0.02	-0.14	0.10	0.798	520	0.02	-0.10	0.14	0.698
Free cholesterol to total lipids ratio in very small VLDL (%)	520	0.14	-0.01	0.30	0.070	520	0.23	0.04	0.42	0.017	520	0.13	-0.07	0.33	0.194
Triglycerides to total lipids ratio in very small VLDL (%)	520	-0.03	-0.16	0.09	0.596	520	-0.06	-0.21	0.09	0.425	520	-0.04	-0.18	0.11	0.632
Phospholipids to total lipds ratio in IDL (%)	520	-0.14	-0.28	0.01	0.077	520	-0.10	-0.25	0.06	0.222	520	0.02	-0.16	0.19	0.857
Total cholesterol to total lipids ratio in IDL (%)	520	0.08	-0.05	0.20	0.239	520	0.02	-0.11	0.16	0.722	520	-0.05	-0.20	0.09	0.478
Cholesterol esters to total lipids ratio in IDL (%)	520	0.08	-0.05	0.22	0.222	520	0.02	-0.12	0.16	0.775	520	-0.07	-0.22	0.09	0.390
Free cholesterol to total lipids ratio in IDL (%)	520	-0.04	-0.16	0.08	0.544	520	0.01	-0.11	0.13	0.888	520	0.05	-0.07	0.17	0.384
Triglycerides to total lipids ratio in IDL (%)	520	-0.02	-0.14	0.10	0.722	520	0.01	-0.11	0.14	0.851	520	0.04	-0.09	0.18	0.531
Phospholipids to total lipds ratio in large LDL (%)	520	0.01	-0.09	0.12	0.839	520	-0.01	-0.11	0.10	0.898	520	-0.04	-0.14	0.06	0.450
Total cholesterol to total lipids ratio in large LDL (%)	520	0.04	-0.06	0.14	0.435	520	0.00	-0.10	0.10	0.970	520	-0.04	-0.15	0.07	0.472
Cholesterol esters to total lipids ratio in large LDL (%)	520	0.03	-0.06	0.13	0.477	520	-0.01	-0.11	0.08	0.775	520	-0.05	-0.15	0.06	0.378
Free cholesterol to total lipids ratio in large LDL (%)	520	-0.03	-0.12	0.07	0.599	520	0.05	-0.06	0.16	0.358	520	0.08	-0.02	0.19	0.116
Triglycerides to total lipids ratio in large LDL (%)	520	-0.05	-0.17	0.07	0.399	520	0.02	-0.11	0.14	0.780	520	0.08	-0.06	0.22	0.240
Phospholipids to total lipds ratio in medium LDL (%)	520	0.01	-0.04	0.06	0.686	520	0.01	-0.04	0.06	0.676	520	0.00	-0.05	0.04	0.862
Total cholesterol to total lipids ratio in medium LDL (%)	520	0.00	-0.12	0.11	0.990	520	-0.02	-0.14	0.10	0.752	520	-0.01	-0.13	0.11	0.866
Cholesterol esters to total lipids ratio in medium LDL (%)	520	-0.01	-0.14	0.11	0.844	520	-0.03	-0.15	0.09	0.647	520	-0.01	-0.13	0.12	0.924
Free cholesterol to total lipids ratio in medium LDL (%)	520	0.00	-0.03	0.04	0.794	520	0.01	-0.02	0.05	0.528	520	0.00	-0.03	0.04	0.792
Triglycerides to total lipids ratio in medium LDL (%)	520	-0.04	-0.16	0.09	0.534	520	0.00	-0.13	0.13	0.971	520	0.04	-0.10	0.18	0.564

Phospholipids to total lipds ratio in small LDL (%)	520	0.01	-0.06	0.09	0.758	520	0.02	-0.06	0.09	0.646	520	0.00	-0.07	0.08	0.979
Total cholesterol to total lipids ratio in small LDL (%)	520	0.00	-0.12	0.12	0.975	520	-0.02	-0.13	0.10	0.802	520	-0.01	-0.13	0.12	0.924
Cholesterol esters to total lipids ratio in small LDL (%)	520	-0.02	-0.15	0.11	0.724	520	-0.04	-0.17	0.09	0.566	520	-0.01	-0.14	0.12	0.875
Free cholesterol to total lipids ratio in small LDL (%)	520	0.02	-0.06	0.09	0.660	520	0.04	-0.04	0.12	0.331	520	0.03	-0.05	0.11	0.476
Triglycerides to total lipids ratio in small LDL (%)	520	-0.01	-0.13	0.11	0.882	520	-0.03	-0.16	0.10	0.655	520	-0.03	-0.17	0.11	0.684
Phospholipids to total lipds ratio in very large HDL (%)	520	-0.01	-0.11	0.09	0.818	520	0.08	-0.03	0.20	0.146	520	0.12	0.01	0.22	0.029
Total cholesterol to total lipids ratio in very large HDL (%)	520	0.01	-0.09	0.11	0.868	520	-0.08	-0.20	0.03	0.136	520	-0.12	-0.22	-0.02	0.021
Cholesterol esters to total lipids ratio in very large HDL (%)	520	0.01	-0.09	0.11	0.786	520	-0.08	-0.19	0.03	0.167	520	-0.12	-0.22	-0.02	0.021
Free cholesterol to total lipids ratio in very large HDL (%)	520	-0.05	-0.18	0.09	0.488	520	-0.04	-0.17	0.09	0.571	520	0.02	-0.12	0.17	0.785
Triglycerides to total lipids ratio in very large HDL (%)	520	0.03	-0.10	0.16	0.625	520	0.00	-0.11	0.12	0.932	520	-0.02	-0.15	0.12	0.813
Phospholipids to total lipds ratio in large HDL (%)	520	-0.03	-0.15	0.09	0.575	520	-0.15	-0.28	-0.02	0.026	520	-0.16	-0.31	-0.01	0.031
Total cholesterol to total lipids ratio in large HDL (%)	520	0.02	-0.10	0.14	0.714	520	0.13	0.01	0.25	0.041	520	0.14	0.00	0.28	0.051
Cholesterol esters to total lipids ratio in large HDL (%)	520	0.02	-0.10	0.14	0.719	520	0.14	0.01	0.26	0.030	520	0.15	0.01	0.29	0.042
Free cholesterol to total lipids ratio in large HDL (%)	520	0.02	-0.10	0.13	0.765	520	0.07	-0.05	0.20	0.242	520	0.09	-0.04	0.22	0.194
Triglycerides to total lipids ratio in large HDL (%)	520	0.03	-0.09	0.15	0.672	520	-0.05	-0.17	0.07	0.394	520	-0.08	-0.21	0.05	0.256
Phospholipids to total lipds ratio in medium HDL (%)	520	0.08	-0.05	0.21	0.214	520	0.09	-0.05	0.23	0.223	520	0.03	-0.13	0.18	0.734
Total cholesterol to total lipids ratio in medium HDL (%)	520	-0.06	-0.19	0.06	0.303	520	-0.02	-0.16	0.11	0.726	520	0.03	-0.11	0.17	0.673
Cholesterol esters to total lipids ratio in medium HDL (%)	520	-0.06	-0.19	0.07	0.356	520	-0.02	-0.15	0.11	0.761	520	0.02	-0.13	0.17	0.768
Free cholesterol to total lipids ratio in medium HDL (%)	520	-0.04	-0.21	0.14	0.671	520	-0.02	-0.17	0.13	0.789	520	0.05	-0.16	0.25	0.634
Triglycerides to total lipids ratio in medium HDL (%)	520	-0.01	-0.12	0.11	0.923	520	-0.09	-0.22	0.04	0.197	520	-0.09	-0.23	0.04	0.178
Phospholipids to total lipds ratio in small HDL (%)	520	-0.13	-0.23	-0.03	0.014	520	-0.07	-0.18	0.04	0.233	520	0.04	-0.08	0.16	0.541
Total cholesterol to total lipids ratio in small HDL (%)	520	0.14	0.03	0.24	0.012	520	0.08	-0.03	0.20	0.149	520	-0.03	-0.16	0.09	0.612
Cholesterol esters to total lipids ratio in small HDL (%)	520	0.13	0.02	0.23	0.019	520	0.06	-0.05	0.18	0.280	520	-0.04	-0.17	0.08	0.485
Free cholesterol to total lipids ratio in small HDL (%)	520	0.02	-0.10	0.14	0.697	520	0.12	0.00	0.25	0.046	520	0.10	-0.04	0.24	0.172
Triglycerides to total lipids ratio in small HDL (%)	520	-0.06	-0.18	0.06	0.321	520	-0.09	-0.22	0.04	0.181	520	-0.03	-0.16	0.11	0.708
Mean diameter for VLDL particles (nm)	520	-0.04	-0.16	0.09	0.572	520	-0.10	-0.23	0.03	0.127	520	-0.09	-0.22	0.05	0.204
Mean diameter for LDL particles (nm)	520	0.01	-0.09	0.12	0.817	520	0.09	-0.03	0.21	0.131	520	0.11	-0.01	0.24	0.065
Mean diameter for HDL particles (nm)	520	0.01	-0.10	0.11	0.901	520	0.10	-0.02	0.21	0.108	520	0.12	0.01	0.23	0.040
Serum total cholesterol (mmol/l)	520	0.02	-0.06	0.11	0.591	520	-0.01	-0.10	0.09	0.895	520	-0.03	-0.14	0.07	0.516
Total cholesterol in VLDL (mmol/l)	520	0.04	-0.05	0.14	0.359	520	-0.05	-0.15	0.05	0.314	520	-0.11	-0.22	0.01	0.062
Remnant cholesterol (non-HDL, non-LDL -cholesterol) (mmol/l)	520	0.04	-0.05	0.12	0.413	520	-0.04	-0.13	0.05	0.385	520	-0.09	-0.20	0.02	0.110
Total cholesterol in LDL (mmol/l)	520	-0.01	-0.11	0.09	0.801	520	-0.05	-0.16	0.06	0.406	520	-0.04	-0.16	0.07	0.460
Total cholesterol in HDL (mmol/l)	520	0.06	-0.04	0.15	0.222	520	0.09	-0.01	0.19	0.080	520	0.05	-0.06	0.15	0.404
Total cholesterol in HDL2 (mmol/l)	520	0.05	-0.05	0.14	0.345	520	0.09	-0.02	0.19	0.094	520	0.06	-0.06	0.17	0.324
Total cholesterol in HDL3 (mmol/l)	520	0.08	-0.01	0.17	0.098	520	0.09	-0.01	0.19	0.084	520	0.02	-0.08	0.13	0.682
Esterified cholesterol (mmol/l)	520	0.02	-0.07	0.11	0.704	520	-0.01	-0.11	0.09	0.872	520	-0.03	-0.13	0.07	0.577
Free cholesterol (mmol/l)	520	0.04	-0.05	0.13	0.357	520	-0.01	-0.11	0.10	0.912	520	-0.05	-0.15	0.05	0.338
Serum total triglycerides (mmol/l)	520	0.00	-0.11	0.11	0.966	520	-0.08	-0.21	0.04	0.178	520	-0.10	-0.22	0.03	0.144
Triglycerides in VLDL (mmol/l)	520	0.00	-0.11	0.11	0.941	520	-0.08	-0.20	0.04	0.181	520	-0.09	-0.21	0.03	0.154
Triglycerides in LDL (mmol/l)	520	-0.03	-0.15	0.10	0.691	520	-0.08	-0.21	0.06	0.258	520	-0.07	-0.21	0.08	0.364
Triglycerides in HDL (mmol/l)	520	0.03	-0.07	0.14	0.541	520	-0.01	-0.13	0.11	0.859	520	-0.05	-0.18	0.08	0.456
Diacylglycerol (mmol/l)	520	0.05	-0.07	0.17	0.386	520	0.06	-0.07	0.18	0.369	520	0.03	-0.11	0.17	0.691
Ratio of diacylglycerol to triglycerides	520	0.03	-0.11	0.17	0.669	520	0.06	-0.09	0.20	0.440	520	0.05	-0.11	0.21	0.560
Total phosphoglycerides (mmol/l)	520	0.08	-0.03	0.19	0.160	520	0.03	-0.08	0.13	0.598	520	-0.05	-0.18	0.07	0.420
Ratio of triglycerides to phosphoglycerides	520	-0.01	-0.13	0.11	0.867	520	-0.06	-0.17	0.06	0.337	520	-0.03	-0.15	0.09	0.661
Phosphatidylcholine and other cholines (mmol/l)	520	0.08	-0.02	0.18	0.114	520	0.04	-0.06	0.15	0.430	520	-0.03	-0.15	0.08	0.590
Total cholines (mmol/l)	520	0.08	-0.03	0.18	0.162	520	0.03	-0.08	0.13	0.626	520	-0.04	-0.16	0.08	0.493
Apolipoprotein A-I (g/l)	520	0.05	-0.04	0.14	0.256	520	0.06	-0.04	0.16	0.221	520	0.02	-0.08	0.12	0.740
Apolipoprotein B (g/l)	520	0.00	-0.10	0.10	0.949	520	-0.08	-0.19	0.03	0.160	520	-0.10	-0.22	0.02	0.103
Ratio of apolipoprotein B to apolipoprotein A-I	520	-0.02	-0.12	0.09	0.719	520	-0.11	-0.22	0.00	0.060	520	-0.11	-0.23	0.02	0.092
Total fatty acids (mmol/l)	520	0.02	-0.08	0.12	0.656	520	-0.05	-0.16	0.05	0.333	520	-0.08	-0.19	0.03	0.161
Estimated description of fatty acid chain length, not actual carbon number	520	0.02	-0.10	0.15	0.739	520	0.04	-0.09	0.17	0.574	520	0.04	-0.09	0.17	0.541
Estimated degree of unsaturation	520	0.02	-0.11	0.15	0.760	520	0.02	-0.11	0.15	0.766	520	0.02	-0.12	0.16	0.778
22:6, docosahexaenoic acid (mmol/l)	520	0.00	-0.11	0.12	0.950	520	-0.03	-0.15	0.09	0.585	520	-0.06	-0.19	0.07	0.376
18:2, linoleic acid (mmol/l)	520	0.04	-0.06	0.14	0.467	520	-0.03	-0.14	0.08	0.572	520	-0.06	-0.17	0.05	0.310
Conjugated linoleic acid (mmol/l)	520	0.00	-0.12	0.13	0.974	520	-0.02	-0.14	0.10	0.760	520	0.01	-0.14	0.16	0.896
Omega-3 fatty acids (mmol/l)	520	-0.06	-0.18	0.05	0.253	520	-0.09	-0.22	0.03	0.133	520	-0.05	-0.18	0.09	0.482

Omega-6 fatty acids (mmol/l)	520	0.03	-0.07	0.13	0.562	520	-0.03	-0.13	0.08	0.626	520	-0.04	-0.15	0.07	0.498
Polyunsaturated fatty acids (mmol/l)	520	0.02	-0.08	0.12	0.751	520	-0.04	-0.14	0.07	0.480	520	-0.04	-0.15	0.07	0.488
Monounsaturated fatty acids; 16:1, 18:1 (mmol/l)	520	0.06	-0.05	0.16	0.271	520	-0.03	-0.14	0.08	0.630	520	-0.10	-0.22	0.02	0.092
Saturated fatty acids (mmol/l)	520	0.00	-0.11	0.11	0.966	520	-0.06	-0.18	0.05	0.278	520	-0.08	-0.20	0.04	0.201
Ratio of 22:6 docosahexaenoic acid to total fatty acids (%)	520	0.01	-0.13	0.15	0.860	520	0.01	-0.14	0.15	0.927	520	-0.03	-0.17	0.12	0.707
Ratio of 18:2 linoleic acid to total fatty acids (%)	520	0.03	-0.09	0.14	0.630	520	0.03	-0.10	0.17	0.617	520	0.03	-0.09	0.16	0.600
Ratio of conjugated linoleic acid to total fatty acids (%)	520	-0.02	-0.15	0.11	0.791	520	-0.02	-0.15	0.10	0.723	520	0.03	-0.12	0.19	0.662
Ratio of omega-3 fatty acids to total fatty acids (%)	520	-0.09	-0.22	0.04	0.188	520	-0.07	-0.22	0.08	0.363	520	0.01	-0.15	0.16	0.940
Ratio of omega-6 fatty acids to total fatty acids (%)	520	0.03	-0.09	0.15	0.663	520	0.05	-0.09	0.18	0.508	520	0.06	-0.07	0.20	0.351
Ratio of polyunsaturated fatty acids to total fatty acids (%)	520	0.01	-0.12	0.13	0.928	520	0.03	-0.11	0.17	0.687	520	0.06	-0.08	0.20	0.373
Ratio of monounsaturated fatty acids to total fatty acids (%)	520	0.07	-0.06	0.21	0.286	520	0.03	-0.12	0.17	0.711	520	-0.06	-0.20	0.07	0.341
Ratio of saturated fatty acids to total fatty acids (%)	520	-0.10	-0.24	0.04	0.149	520	-0.06	-0.21	0.09	0.432	520	0.02	-0.11	0.15	0.794
Glucose (mmol/l)	520	-0.04	-0.16	0.08	0.483	520	-0.12	-0.24	-0.01	0.038	520	-0.08	-0.22	0.06	0.275
Lactate (mmol/l)	520	-0.06	-0.19	0.07	0.370	520	-0.02	-0.15	0.11	0.805	520	0.07	-0.09	0.22	0.398
Pyruvate (mmol/l)	520	-0.15	-0.28	-0.02	0.028	520	-0.10	-0.23	0.04	0.165	520	0.08	-0.06	0.23	0.242
Citrate (mmol/l)	520	0.17	0.03	0.30	0.020	520	0.07	-0.08	0.21	0.365	520	-0.16	-0.31	0.00	0.044
Alanine (mmol/l)	520	-0.16	-0.28	-0.03	0.017	520	-0.06	-0.19	0.06	0.339	520	0.14	0.00	0.29	0.057
Glutamine (mmol/l)	520	0.07	-0.03	0.18	0.175	520	0.03	-0.08	0.15	0.577	520	-0.05	-0.17	0.08	0.471
Histidine (mmol/l)	520	0.06	-0.07	0.19	0.341	520	0.16	0.02	0.30	0.023	520	0.11	-0.03	0.25	0.135
Isoleucine (mmol/l)	520	0.00	-0.12	0.13	0.939	520	0.00	-0.13	0.12	0.974	520	0.01	-0.13	0.15	0.874
Leucine (mmol/l)	520	0.09	-0.03	0.20	0.158	520	0.09	-0.03	0.21	0.152	520	0.01	-0.12	0.14	0.907
Valine (mmol/l)	520	0.04	-0.07	0.16	0.480	520	0.02	-0.11	0.16	0.716	520	-0.01	-0.15	0.13	0.878
Phenylalanine (mmol/l)	520	0.11	-0.02	0.24	0.103	520	0.13	0.00	0.26	0.058	520	0.04	-0.11	0.19	0.569
Tyrosine (mmol/l)	520	0.06	-0.08	0.20	0.383	520	0.03	-0.12	0.18	0.690	520	-0.01	-0.16	0.15	0.938
Acetate (mmol/l)	520	0.12	0.00	0.24	0.046	520	0.13	-0.01	0.26	0.062	520	0.03	-0.11	0.17	0.638
Acetoacetate (mmol/l)	520	0.02	-0.08	0.13	0.657	520	0.03	-0.08	0.14	0.587	520	-0.01	-0.14	0.11	0.866
3-hydroxybutyrate (mmol/l)	520	-0.05	-0.17	0.08	0.454	520	-0.07	-0.20	0.05	0.256	520	-0.03	-0.17	0.11	0.643
Creatinine (mmol/l)	520	-0.03	-0.13	0.07	0.527	520	0.01	-0.11	0.13	0.865	520	0.04	-0.08	0.15	0.542
Albumin (signal area)	520	-0.15	-0.27	-0.02	0.025	520	-0.08	-0.21	0.06	0.264	520	0.06	-0.08	0.20	0.379
Glycoprotein acetyls, mainly a1-acid glycoprotein (mmol/l)	520	-0.09	-0.20	0.03	0.139	520	-0.15	-0.25	-0.05	0.004	520	-0.03	-0.15	0.09	0.621
