# Supplementary material for: Associations of device-measured physical activity across adolescence with metabolic traits: Prospective cohort study
Source: PLoS Med. 2018 Sep 11;15(9):e1002649. doi: 10.1371/journal.pmed.1002649 (PMC6133272; doi:10.1371/journal.pmed.1002649)
Supplement: S14 Table — ALSPAC, Avon Longitudinal Study of Parents and Children. (PDF) [file pmed.1002649.s014.pdf]

S14 Table Associations of change in physical activity (change in measures from age 12y-15y) with metabolic traits at age 15y, with adjustment for metabolic traits at age 8y in ALSPAC

	**Change in CPM from age 12y-15y (per SD-unit increase)** *Adj. for age, sex, ethnicity, maternal education, wear time change, wear month, FMI change, metabolic trait at 8y*					**Change in MVPA from age 12y-15y (per SD-unit increase)** *Adj. for age, sex, ethnicity, maternal education, wear time change, wear month, SED change, FMI change, metabolic trait at 8y*					**Change in SED from age 12y-15y (per SD-unit increase)** *Adj. for age, sex, ethnicity, maternal education, wear time change, wear month, MVPA change, FMI change, metabolic trait at 8y*				
**Standardised outcome at age 15y**	**N**	**Beta**	**LCL**	**UCL**	**P-value**	**N**	**Beta**	**LCL**	**UCL**	**P-value**	**N**	**Beta**	**LCL**	**UCL**	**P-value**
Systolic blood pressure (mmHg)	1393	0.01	-0.04	0.05	0.826	1393	0.02	-0.02	0.07	0.347	1393	0.00	-0.05	0.05	0.945
Diastolic blood pressure (mmHg)	1392	0.04	-0.01	0.09	0.137	1392	0.07	0.02	0.13	0.008	1392	0.05	0.00	0.10	0.066
Concentration of chylomicrons and extremely large VLDL particles (mol/l)	735	-0.03	-0.08	0.03	0.312	735	-0.03	-0.09	0.03	0.291	735	0.01	-0.06	0.08	0.794
Total lipids in chylomicrons and extremely large VLDL (mmol/l)	735	-0.03	-0.09	0.03	0.305	735	-0.03	-0.09	0.03	0.306	735	0.01	-0.06	0.08	0.773
Phospholipids in chylomicrons and extremely large VLDL (mmol/l)	735	-0.03	-0.09	0.03	0.283	735	-0.03	-0.09	0.03	0.284	735	0.01	-0.06	0.07	0.832
Total cholesterol in chylomicrons and extremely large VLDL (mmol/l)	735	-0.03	-0.08	0.03	0.358	735	-0.03	-0.09	0.03	0.285	735	0.01	-0.06	0.08	0.721
Cholesterol esters in chylomicrons and extremely large VLDL (mmol/l)	735	-0.02	-0.08	0.04	0.452	735	-0.03	-0.09	0.03	0.302	735	0.01	-0.05	0.08	0.682
Free cholesterol in chylomicrons and extremely large VLDL (mmol/l)	735	-0.03	-0.09	0.03	0.298	735	-0.03	-0.09	0.03	0.277	735	0.01	-0.06	0.08	0.841
Triglycerides in chylomicrons and extremely large VLDL (mmol/l)	735	-0.03	-0.09	0.03	0.300	735	-0.03	-0.09	0.03	0.316	735	0.01	-0.06	0.08	0.782
Concentration of very large VLDL particles (mol/l)	735	-0.03	-0.09	0.02	0.241	735	-0.03	-0.08	0.03	0.383	735	0.03	-0.04	0.10	0.436
Total lipids in very large VLDL (mmol/l)	735	-0.03	-0.09	0.02	0.238	735	-0.03	-0.09	0.03	0.375	735	0.03	-0.04	0.10	0.449
Phospholipids in very large VLDL (mmol/l)	735	-0.03	-0.09	0.02	0.225	735	-0.03	-0.09	0.02	0.262	735	0.02	-0.05	0.08	0.659
Total cholesterol in very large VLDL (mmol/l)	735	-0.03	-0.09	0.03	0.291	735	-0.03	-0.09	0.03	0.295	735	0.02	-0.05	0.08	0.642
Cholesterol esters in very large VLDL (mmol/l)	735	-0.03	-0.09	0.03	0.311	735	-0.03	-0.08	0.03	0.355	735	0.02	-0.05	0.09	0.513
Free cholesterol in very large VLDL (mmol/l)	735	-0.03	-0.09	0.03	0.277	735	-0.03	-0.09	0.02	0.242	735	0.01	-0.06	0.08	0.810
Triglycerides in very large VLDL (mmol/l)	735	-0.04	-0.09	0.02	0.230	735	-0.02	-0.08	0.04	0.440	735	0.03	-0.04	0.10	0.357
Concentration of large VLDL particles (mol/l)	735	-0.03	-0.09	0.03	0.300	735	-0.02	-0.08	0.04	0.541	735	0.04	-0.03	0.11	0.294
Total lipids in large VLDL (mmol/l)	735	-0.03	-0.09	0.03	0.287	735	-0.02	-0.08	0.04	0.504	735	0.04	-0.03	0.11	0.308
Phospholipids in large VLDL (mmol/l)	735	-0.03	-0.09	0.03	0.279	735	-0.02	-0.08	0.04	0.453	735	0.03	-0.04	0.10	0.350
Total cholesterol in large VLDL (mmol/l)	735	-0.03	-0.09	0.03	0.273	735	-0.02	-0.08	0.03	0.425	735	0.04	-0.03	0.10	0.313
Cholesterol esters in large VLDL (mmol/l)	735	-0.03	-0.09	0.03	0.323	735	-0.02	-0.08	0.04	0.455	735	0.04	-0.03	0.11	0.306
Free cholesterol in large VLDL (mmol/l)	735	-0.04	-0.10	0.02	0.235	735	-0.03	-0.08	0.03	0.396	735	0.03	-0.04	0.10	0.344
Triglycerides in large VLDL (mmol/l)	735	-0.03	-0.09	0.03	0.298	735	-0.02	-0.08	0.04	0.554	735	0.04	-0.03	0.11	0.299
Concentration of medium VLDL particles (mol/l)	735	-0.04	-0.10	0.03	0.256	735	-0.02	-0.08	0.04	0.505	735	0.04	-0.03	0.11	0.249
Total lipids in medium VLDL (mmol/l)	735	-0.03	-0.10	0.03	0.268	735	-0.02	-0.08	0.04	0.473	735	0.04	-0.03	0.11	0.274
Phospholipids in medium VLDL (mmol/l)	735	-0.04	-0.10	0.02	0.243	735	-0.02	-0.08	0.04	0.435	735	0.04	-0.03	0.11	0.265
Total cholesterol in medium VLDL (mmol/l)	735	-0.03	-0.09	0.03	0.323	735	-0.03	-0.09	0.03	0.324	735	0.03	-0.04	0.10	0.424
Cholesterol esters in medium VLDL (mmol/l)	735	-0.02	-0.08	0.04	0.423	735	-0.03	-0.09	0.02	0.234	735	0.01	-0.05	0.08	0.693
Free cholesterol in medium VLDL (mmol/l)	735	-0.04	-0.10	0.03	0.251	735	-0.02	-0.08	0.04	0.441	735	0.04	-0.03	0.11	0.260
Triglycerides in medium VLDL (mmol/l)	735	-0.03	-0.10	0.03	0.268	735	-0.02	-0.08	0.04	0.575	735	0.04	-0.03	0.11	0.246
Concentration of small VLDL particles (mol/l)	735	-0.03	-0.09	0.03	0.358	735	-0.03	-0.08	0.03	0.401	735	0.03	-0.04	0.09	0.420
Total lipids in small VLDL (mmol/l)	735	-0.02	-0.08	0.04	0.435	735	-0.03	-0.09	0.03	0.305	735	0.01	-0.05	0.08	0.728
Phospholipids in small VLDL (mmol/l)	735	-0.01	-0.07	0.05	0.662	735	-0.02	-0.08	0.03	0.401	735	0.01	-0.06	0.07	0.873
Total cholesterol in small VLDL (mmol/l)	735	-0.01	-0.06	0.04	0.713	735	-0.04	-0.09	0.01	0.085	735	-0.03	-0.10	0.03	0.284
Cholesterol esters in small VLDL (mmol/l)	735	-0.01	-0.06	0.04	0.778	735	-0.05	-0.10	-0.01	0.029	735	-0.06	-0.12	0.01	0.079
Free cholesterol in small VLDL (mmol/l)	735	-0.02	-0.07	0.04	0.591	735	-0.02	-0.08	0.03	0.369	735	0.01	-0.05	0.08	0.757
Triglycerides in small VLDL (mmol/l)	735	-0.03	-0.10	0.03	0.319	735	-0.02	-0.08	0.04	0.535	735	0.04	-0.03	0.11	0.264
Concentration of very small VLDL particles (mol/l)	735	0.00	-0.05	0.06	0.858	735	-0.04	-0.09	0.01	0.125	735	-0.05	-0.11	0.02	0.169
Total lipids in very small VLDL (mmol/l)	735	0.00	-0.05	0.06	0.899	735	-0.05	-0.10	0.00	0.039	735	-0.07	-0.13	0.00	0.040
Phospholipids in very small VLDL (mmol/l)	735	0.01	-0.05	0.06	0.764	735	-0.05	-0.10	0.00	0.064	735	-0.07	-0.13	0.00	0.037
Total cholesterol in very small VLDL (mmol/l)	735	0.00	-0.05	0.06	0.945	735	-0.05	-0.11	0.00	0.039	735	-0.07	-0.14	0.00	0.036
Cholesterol esters in very small VLDL (mmol/l)	735	0.00	-0.05	0.06	0.928	735	-0.05	-0.11	0.00	0.034	735	-0.08	-0.15	-0.01	0.017
Free cholesterol in very small VLDL (mmol/l)	735	0.00	-0.06	0.05	0.968	735	-0.05	-0.10	0.01	0.091	735	-0.04	-0.11	0.03	0.263
Triglycerides in very small VLDL (mmol/l)	735	-0.02	-0.08	0.05	0.591	735	-0.02	-0.08	0.03	0.427	735	0.02	-0.05	0.08	0.606
Concentration of IDL particles (mol/l)	735	0.01	-0.05	0.06	0.829	735	-0.04	-0.09	0.02	0.181	735	-0.05	-0.12	0.01	0.107
Total lipids in IDL (mmol/l)	735	0.01	-0.04	0.07	0.633	735	-0.04	-0.09	0.01	0.136	735	-0.07	-0.13	0.00	0.035

Phospholipids in IDL (mmol/l)	735	0.01	-0.04	0.07	0.626	735	-0.04	-0.10	0.02	0.193	735	-0.07	-0.14	0.00	0.046
Total cholesterol in IDL (mmol/l)	735	0.01	-0.04	0.07	0.601	735	-0.04	-0.09	0.01	0.111	735	-0.07	-0.14	-0.01	0.025
Cholesterol esters in IDL (mmol/l)	735	0.01	-0.04	0.07	0.639	735	-0.04	-0.09	0.01	0.094	735	-0.07	-0.13	-0.01	0.030
Free cholesterol in IDL (mmol/l)	735	0.02	-0.04	0.08	0.572	735	-0.04	-0.09	0.02	0.186	735	-0.07	-0.14	-0.01	0.032
Triglycerides in IDL (mmol/l)	735	0.00	-0.07	0.06	0.960	735	-0.02	-0.08	0.04	0.454	735	-0.01	-0.08	0.06	0.756
Concentration of large LDL particles (mol/l)	735	0.02	-0.04	0.07	0.606	735	-0.03	-0.09	0.03	0.273	735	-0.06	-0.13	0.01	0.079
Total lipids in large LDL (mmol/l)	735	0.02	-0.04	0.08	0.507	735	-0.04	-0.09	0.02	0.219	735	-0.07	-0.14	0.00	0.035
Phospholipids in large LDL (mmol/l)	735	0.02	-0.03	0.08	0.441	735	-0.04	-0.09	0.02	0.211	735	-0.08	-0.14	-0.01	0.024
Total cholesterol in large LDL (mmol/l)	735	0.02	-0.04	0.08	0.473	735	-0.04	-0.09	0.02	0.206	735	-0.07	-0.14	-0.01	0.026
Cholesterol esters in large LDL (mmol/l)	735	0.02	-0.04	0.08	0.482	735	-0.04	-0.09	0.02	0.201	735	-0.07	-0.14	-0.01	0.027
Free cholesterol in large LDL (mmol/l)	735	0.02	-0.04	0.08	0.453	735	-0.04	-0.09	0.02	0.228	735	-0.08	-0.15	-0.01	0.025
Triglycerides in large LDL (mmol/l)	735	0.00	-0.06	0.07	0.967	735	-0.02	-0.09	0.04	0.456	735	-0.02	-0.09	0.05	0.519
Concentration of medium LDL particles (mol/l)	735	0.01	-0.05	0.07	0.663	735	-0.03	-0.09	0.03	0.312	735	-0.05	-0.12	0.01	0.122
Total lipids in medium LDL (mmol/l)	735	0.02	-0.04	0.08	0.537	735	-0.03	-0.09	0.02	0.243	735	-0.07	-0.13	0.00	0.050
Phospholipids in medium LDL (mmol/l)	735	0.02	-0.03	0.08	0.380	735	-0.03	-0.08	0.02	0.261	735	-0.07	-0.13	-0.01	0.031
Total cholesterol in medium LDL (mmol/l)	735	0.02	-0.04	0.08	0.548	735	-0.04	-0.09	0.02	0.217	735	-0.07	-0.14	0.00	0.042
Cholesterol esters in medium LDL (mmol/l)	735	0.02	-0.04	0.07	0.603	735	-0.04	-0.10	0.02	0.195	735	-0.07	-0.14	0.00	0.047
Free cholesterol in medium LDL (mmol/l)	735	0.03	-0.03	0.08	0.367	735	-0.03	-0.08	0.03	0.365	735	-0.06	-0.13	0.00	0.050
Triglycerides in medium LDL (mmol/l)	735	0.01	-0.05	0.08	0.744	735	-0.02	-0.08	0.05	0.585	735	-0.03	-0.11	0.04	0.381
Concentration of small LDL particles (mol/l)	735	0.01	-0.04	0.07	0.648	735	-0.03	-0.09	0.03	0.390	735	-0.05	-0.11	0.02	0.152
Total lipids in small LDL (mmol/l)	735	0.02	-0.04	0.08	0.518	735	-0.03	-0.09	0.03	0.282	735	-0.06	-0.13	0.00	0.055
Phospholipids in small LDL (mmol/l)	735	0.02	-0.03	0.08	0.433	735	-0.02	-0.08	0.03	0.412	735	-0.05	-0.11	0.01	0.075
Total cholesterol in small LDL (mmol/l)	735	0.02	-0.04	0.08	0.498	735	-0.03	-0.09	0.02	0.240	735	-0.07	-0.14	0.00	0.038
Cholesterol esters in small LDL (mmol/l)	735	0.02	-0.04	0.08	0.579	735	-0.04	-0.10	0.02	0.231	735	-0.07	-0.14	0.00	0.052
Free cholesterol in small LDL (mmol/l)	735	0.03	-0.03	0.09	0.293	735	-0.02	-0.08	0.03	0.366	735	-0.07	-0.13	-0.01	0.031
Triglycerides in small LDL (mmol/l)	735	0.00	-0.06	0.06	0.950	735	-0.02	-0.09	0.04	0.455	735	-0.02	-0.09	0.05	0.623
Concentration of very large HDL particles (mol/l)	735	0.01	-0.05	0.08	0.671	735	0.01	-0.06	0.08	0.771	735	0.00	-0.07	0.06	0.935
Total lipids in very large HDL (mmol/l)	735	0.01	-0.05	0.08	0.672	735	0.01	-0.06	0.08	0.719	735	0.00	-0.06	0.06	0.997
Phospholipids in very large HDL (mmol/l)	735	0.01	-0.05	0.08	0.654	735	0.01	-0.06	0.08	0.796	735	-0.01	-0.07	0.06	0.849
Total cholesterol in very large HDL (mmol/l)	735	0.02	-0.05	0.09	0.655	735	0.02	-0.06	0.09	0.651	735	0.00	-0.06	0.07	0.957
Cholesterol esters in very large HDL (mmol/l)	735	0.01	-0.06	0.08	0.692	735	0.02	-0.06	0.09	0.694	735	0.00	-0.07	0.07	0.983
Free cholesterol in very large HDL (mmol/l)	735	0.02	-0.05	0.09	0.582	735	0.02	-0.05	0.09	0.566	735	0.00	-0.06	0.07	0.913
Triglycerides in very large HDL (mmol/l)	735	-0.04	-0.10	0.03	0.311	735	-0.03	-0.10	0.04	0.393	735	0.03	-0.04	0.10	0.397
Concentration of large HDL particles (mol/l)	735	0.02	-0.04	0.08	0.532	735	0.01	-0.06	0.08	0.820	735	-0.02	-0.08	0.05	0.618
Total lipids in large HDL (mmol/l)	735	0.02	-0.04	0.08	0.511	735	0.01	-0.06	0.08	0.773	735	-0.02	-0.08	0.05	0.622
Phospholipids in large HDL (mmol/l)	735	0.02	-0.04	0.08	0.513	735	0.00	-0.06	0.07	0.929	735	-0.03	-0.09	0.04	0.426
Total cholesterol in large HDL (mmol/l)	735	0.02	-0.04	0.09	0.485	735	0.02	-0.05	0.08	0.613	735	-0.01	-0.07	0.06	0.823
Cholesterol esters in large HDL (mmol/l)	735	0.02	-0.04	0.08	0.490	735	0.02	-0.05	0.08	0.599	735	-0.01	-0.07	0.06	0.863
Free cholesterol in large HDL (mmol/l)	735	0.02	-0.04	0.09	0.472	735	0.01	-0.05	0.08	0.666	735	-0.01	-0.07	0.05	0.682
Triglycerides in large HDL (mmol/l)	735	-0.03	-0.09	0.03	0.347	735	-0.05	-0.11	0.02	0.153	735	-0.02	-0.09	0.05	0.634
Concentration of medium HDL particles (mol/l)	735	0.02	-0.04	0.09	0.453	735	-0.01	-0.07	0.05	0.791	735	-0.04	-0.11	0.03	0.272
Total lipids in medium HDL (mmol/l)	735	0.02	-0.04	0.09	0.456	735	-0.01	-0.07	0.06	0.803	735	-0.04	-0.11	0.03	0.234
Phospholipids in medium HDL (mmol/l)	735	0.03	-0.04	0.09	0.379	735	-0.01	-0.07	0.05	0.790	735	-0.04	-0.11	0.02	0.212
Total cholesterol in medium HDL (mmol/l)	735	0.02	-0.04	0.09	0.479	735	0.00	-0.06	0.07	0.990	735	-0.04	-0.11	0.03	0.310
Cholesterol esters in medium HDL (mmol/l)	735	0.02	-0.04	0.09	0.488	735	0.00	-0.06	0.07	0.882	735	-0.03	-0.11	0.05	0.428
Free cholesterol in medium HDL (mmol/l)	735	0.02	-0.04	0.09	0.467	735	-0.02	-0.08	0.05	0.555	735	-0.06	-0.13	0.00	0.059
Triglycerides in medium HDL (mmol/l)	735	-0.02	-0.09	0.04	0.476	735	-0.05	-0.11	0.02	0.151	735	-0.02	-0.08	0.05	0.631
Concentration of small HDL particles (mol/l)	735	0.02	-0.04	0.08	0.546	735	-0.01	-0.07	0.05	0.779	735	-0.04	-0.11	0.03	0.240
Total lipids in small HDL (mmol/l)	735	0.03	-0.03	0.10	0.297	735	-0.01	-0.07	0.05	0.859	735	-0.05	-0.11	0.02	0.150
Phospholipids in small HDL (mmol/l)	735	0.01	-0.06	0.07	0.827	735	0.00	-0.06	0.06	0.886	735	-0.02	-0.09	0.05	0.550
Total cholesterol in small HDL (mmol/l)	735	0.06	-0.01	0.12	0.077	735	0.00	-0.06	0.06	0.971	735	-0.06	-0.13	0.01	0.077
Cholesterol esters in small HDL (mmol/l)	735	0.06	0.00	0.12	0.060	735	0.00	-0.06	0.06	0.959	735	-0.07	-0.13	0.00	0.047
Free cholesterol in small HDL (mmol/l)	735	0.02	-0.04	0.09	0.503	735	0.01	-0.05	0.07	0.680	735	-0.01	-0.08	0.06	0.835
Triglycerides in small HDL (mmol/l)	735	-0.02	-0.09	0.05	0.600	735	-0.03	-0.09	0.03	0.374	735	0.01	-0.06	0.08	0.834
Phospholipids to total lipds ratio in chylomicrons and extremely large VLDL (%)	735	0.00	-0.07	0.06	0.888	735	-0.03	-0.10	0.03	0.322	735	-0.04	-0.11	0.04	0.327
Total cholesterol to total lipids ratio in chylomicrons and extremely large VLDL (%)	735	0.01	-0.06	0.08	0.786	735	-0.01	-0.07	0.06	0.808	735	-0.01	-0.08	0.07	0.882
Cholesterol esters to total lipids ratio in chylomicrons and extremely large VLDL (%)	735	0.00	-0.06	0.07	0.906	735	-0.01	-0.08	0.05	0.715	735	0.00	-0.08	0.07	0.981

Free cholesterol to total lipids ratio in chylomicrons and extremely large VLDL (%)	735	0.00	-0.07	0.07	0.993	735	-0.01	-0.07	0.06	0.845	735	0.00	-0.08	0.08	0.936
Triglycerides to total lipids ratio in chylomicrons and extremely large VLDL (%)	735	-0.01	-0.05	0.04	0.748	735	0.01	-0.04	0.05	0.675	735	0.01	-0.04	0.06	0.634
Phospholipids to total lipds ratio in very large VLDL (%)	735	0.01	-0.06	0.07	0.868	735	0.00	-0.07	0.07	0.988	735	0.00	-0.08	0.07	0.926
Total cholesterol to total lipids ratio in very large VLDL (%)	735	-0.03	-0.10	0.04	0.365	735	-0.03	-0.11	0.04	0.362	735	0.02	-0.04	0.09	0.499
Cholesterol esters to total lipids ratio in very large VLDL (%)	735	-0.04	-0.12	0.04	0.304	735	-0.04	-0.12	0.04	0.294	735	0.03	-0.05	0.10	0.490
Free cholesterol to total lipids ratio in very large VLDL (%)	735	-0.02	-0.09	0.05	0.578	735	-0.03	-0.11	0.06	0.549	735	0.02	-0.06	0.10	0.637
Triglycerides to total lipids ratio in very large VLDL (%)	735	0.04	-0.04	0.11	0.350	735	0.04	-0.04	0.13	0.319	735	-0.03	-0.11	0.06	0.539
Phospholipids to total lipds ratio in large VLDL (%)	735	0.04	-0.03	0.12	0.284	735	0.04	-0.04	0.11	0.357	735	-0.01	-0.09	0.06	0.727
Total cholesterol to total lipids ratio in large VLDL (%)	735	0.00	-0.06	0.07	0.918	735	0.01	-0.06	0.08	0.756	735	0.03	-0.04	0.11	0.417
Cholesterol esters to total lipids ratio in large VLDL (%)	735	-0.01	-0.04	0.03	0.716	735	0.00	-0.03	0.03	0.845	735	0.02	-0.02	0.06	0.320
Free cholesterol to total lipids ratio in large VLDL (%)	735	0.02	-0.06	0.09	0.651	735	0.02	-0.05	0.09	0.568	735	0.00	-0.07	0.08	0.925
Triglycerides to total lipids ratio in large VLDL (%)	735	0.00	-0.01	0.01	0.583	735	0.00	-0.01	0.01	0.430	735	0.00	-0.01	0.01	0.512
Phospholipids to total lipds ratio in medium VLDL (%)	735	0.00	-0.07	0.07	0.983	735	0.01	-0.08	0.11	0.778	735	0.04	-0.07	0.14	0.480
Total cholesterol to total lipids ratio in medium VLDL (%)	735	0.01	-0.05	0.07	0.763	735	-0.01	-0.07	0.04	0.674	735	0.00	-0.08	0.07	0.917
Cholesterol esters to total lipids ratio in medium VLDL (%)	735	0.00	-0.06	0.06	0.886	735	-0.02	-0.08	0.04	0.462	735	-0.01	-0.08	0.07	0.832
Free cholesterol to total lipids ratio in medium VLDL (%)	735	0.01	-0.06	0.07	0.789	735	0.03	-0.04	0.10	0.431	735	0.04	-0.04	0.12	0.293
Triglycerides to total lipids ratio in medium VLDL (%)	735	-0.01	-0.07	0.05	0.749	735	0.01	-0.05	0.07	0.769	735	0.00	-0.08	0.08	0.991
Phospholipids to total lipds ratio in small VLDL (%)	735	0.05	-0.01	0.11	0.116	735	0.03	-0.03	0.09	0.318	735	-0.04	-0.11	0.04	0.311
Total cholesterol to total lipids ratio in small VLDL (%)	735	0.00	-0.07	0.07	0.947	735	-0.05	-0.12	0.02	0.149	735	-0.06	-0.14	0.02	0.153
Cholesterol esters to total lipids ratio in small VLDL (%)	735	-0.01	-0.08	0.06	0.815	735	-0.06	-0.13	0.01	0.091	735	-0.06	-0.14	0.01	0.100
Free cholesterol to total lipids ratio in small VLDL (%)	735	0.04	-0.03	0.10	0.277	735	0.03	-0.05	0.10	0.495	735	0.02	-0.09	0.13	0.774
Triglycerides to total lipids ratio in small VLDL (%)	735	-0.01	-0.08	0.06	0.774	735	0.03	-0.04	0.11	0.337	735	0.06	-0.02	0.14	0.154
Phospholipids to total lipds ratio in very small VLDL (%)	735	0.01	-0.05	0.08	0.701	735	-0.03	-0.09	0.04	0.378	735	-0.04	-0.10	0.03	0.275
Total cholesterol to total lipids ratio in very small VLDL (%)	735	-0.01	-0.08	0.06	0.813	735	-0.01	-0.07	0.06	0.807	735	-0.02	-0.10	0.05	0.515
Cholesterol esters to total lipids ratio in very small VLDL (%)	735	-0.01	-0.08	0.06	0.796	735	-0.01	-0.08	0.05	0.678	735	-0.04	-0.11	0.03	0.260
Free cholesterol to total lipids ratio in very small VLDL (%)	735	0.00	-0.05	0.05	0.966	735	0.00	-0.05	0.06	0.854	735	0.03	-0.04	0.10	0.393
Triglycerides to total lipids ratio in very small VLDL (%)	735	0.00	-0.06	0.07	0.962	735	0.03	-0.03	0.09	0.391	735	0.05	-0.02	0.11	0.196
Phospholipids to total lipds ratio in IDL (%)	735	0.04	-0.02	0.09	0.179	735	0.01	-0.05	0.07	0.702	735	-0.09	-0.20	0.03	0.138
Total cholesterol to total lipids ratio in IDL (%)	735	-0.02	-0.08	0.04	0.511	735	-0.02	-0.08	0.04	0.434	735	0.00	-0.07	0.08	0.918
Cholesterol esters to total lipids ratio in IDL (%)	735	-0.03	-0.09	0.03	0.319	735	-0.02	-0.08	0.04	0.600	735	0.03	-0.05	0.11	0.480
Free cholesterol to total lipids ratio in IDL (%)	735	0.02	-0.04	0.09	0.519	735	-0.02	-0.08	0.05	0.602	735	-0.06	-0.13	0.02	0.130
Triglycerides to total lipids ratio in IDL (%)	735	0.01	-0.06	0.07	0.836	735	0.02	-0.03	0.08	0.420	735	0.04	-0.03	0.10	0.311
Phospholipids to total lipds ratio in large LDL (%)	735	0.00	-0.06	0.05	0.872	735	0.04	-0.02	0.09	0.172	735	0.03	-0.02	0.09	0.238
Total cholesterol to total lipids ratio in large LDL (%)	735	0.01	-0.05	0.07	0.756	735	-0.04	-0.09	0.02	0.178	735	-0.05	-0.11	0.01	0.083
Cholesterol esters to total lipids ratio in large LDL (%)	735	0.01	-0.04	0.07	0.626	735	-0.04	-0.09	0.01	0.150	735	-0.06	-0.11	0.00	0.045
Free cholesterol to total lipids ratio in large LDL (%)	735	0.00	-0.05	0.06	0.885	735	0.03	-0.03	0.08	0.317	735	0.00	-0.06	0.06	0.969
Triglycerides to total lipids ratio in large LDL (%)	735	0.00	-0.07	0.06	0.955	735	0.02	-0.04	0.08	0.611	735	0.03	-0.04	0.10	0.426
Phospholipids to total lipds ratio in medium LDL (%)	735	0.00	-0.02	0.02	0.991	735	0.02	0.00	0.04	0.122	735	0.02	-0.01	0.04	0.218
Total cholesterol to total lipids ratio in medium LDL (%)	735	0.00	-0.07	0.06	0.887	735	-0.05	-0.11	0.01	0.096	735	-0.05	-0.12	0.02	0.151
Cholesterol esters to total lipids ratio in medium LDL (%)	735	0.00	-0.06	0.06	0.995	735	-0.06	-0.12	0.01	0.075	735	-0.06	-0.13	0.01	0.108
Free cholesterol to total lipids ratio in medium LDL (%)	735	0.00	-0.02	0.02	0.911	735	0.02	0.00	0.03	0.090	735	0.01	-0.01	0.04	0.163
Triglycerides to total lipids ratio in medium LDL (%)	735	0.02	-0.04	0.08	0.538	735	0.01	-0.05	0.07	0.816	735	-0.01	-0.08	0.07	0.866
Phospholipids to total lipds ratio in small LDL (%)	735	0.00	-0.04	0.03	0.833	735	0.03	-0.01	0.07	0.102	735	0.03	-0.01	0.08	0.114
Total cholesterol to total lipids ratio in small LDL (%)	735	0.01	-0.06	0.07	0.854	735	-0.05	-0.11	0.01	0.106	735	-0.06	-0.13	0.01	0.081
Cholesterol esters to total lipids ratio in small LDL (%)	735	0.01	-0.06	0.07	0.846	735	-0.05	-0.12	0.01	0.098	735	-0.06	-0.14	0.01	0.090
Free cholesterol to total lipids ratio in small LDL (%)	735	0.00	-0.03	0.03	0.916	735	0.03	-0.01	0.06	0.157	735	0.02	-0.02	0.06	0.248
Triglycerides to total lipids ratio in small LDL (%)	735	0.00	-0.06	0.07	0.967	735	0.00	-0.06	0.06	0.991	735	0.01	-0.06	0.09	0.698
Phospholipids to total lipds ratio in very large HDL (%)	735	0.01	-0.05	0.07	0.722	735	-0.01	-0.08	0.05	0.690	735	-0.03	-0.09	0.03	0.332
Total cholesterol to total lipids ratio in very large HDL (%)	735	0.00	-0.06	0.06	0.997	735	0.02	-0.04	0.09	0.481	735	0.02	-0.04	0.08	0.442
Cholesterol esters to total lipids ratio in very large HDL (%)	735	0.00	-0.07	0.06	0.903	735	0.02	-0.05	0.08	0.611	735	0.02	-0.04	0.08	0.495
Free cholesterol to total lipids ratio in very large HDL (%)	735	0.04	-0.03	0.10	0.280	735	0.05	-0.02	0.12	0.185	735	0.02	-0.07	0.10	0.661
Triglycerides to total lipids ratio in very large HDL (%)	735	-0.06	-0.13	0.01	0.080	735	-0.05	-0.11	0.02	0.136	735	0.05	-0.03	0.12	0.206
Phospholipids to total lipds ratio in large HDL (%)	735	-0.02	-0.08	0.04	0.558	735	-0.05	-0.11	0.00	0.055	735	-0.05	-0.12	0.01	0.115
Total cholesterol to total lipids ratio in large HDL (%)	735	0.03	-0.03	0.09	0.321	735	0.06	0.00	0.11	0.050	735	0.03	-0.03	0.10	0.340
Cholesterol esters to total lipids ratio in large HDL (%)	735	0.03	-0.03	0.09	0.364	735	0.06	0.00	0.11	0.038	735	0.04	-0.03	0.11	0.242
Free cholesterol to total lipids ratio in large HDL (%)	735	0.03	-0.03	0.10	0.317	735	0.04	-0.02	0.10	0.232	735	0.00	-0.06	0.06	0.977
Triglycerides to total lipids ratio in large HDL (%)	735	-0.05	-0.11	0.01	0.129	735	-0.05	-0.11	0.02	0.145	735	0.02	-0.06	0.09	0.665

Phospholipids to total lipds ratio in medium HDL (%)	735	0.04	-0.02	0.11	0.208	735	-0.01	-0.08	0.05	0.694	735	-0.05	-0.13	0.03	0.218
Total cholesterol to total lipids ratio in medium HDL (%)	735	-0.01	-0.08	0.06	0.743	735	0.04	-0.03	0.10	0.248	735	0.04	-0.04	0.12	0.310
Cholesterol esters to total lipids ratio in medium HDL (%)	735	-0.01	-0.08	0.06	0.694	735	0.05	-0.02	0.12	0.135	735	0.06	-0.03	0.16	0.199
Free cholesterol to total lipids ratio in medium HDL (%)	735	0.01	-0.07	0.09	0.815	735	-0.05	-0.13	0.04	0.278	735	-0.11	-0.29	0.08	0.253
Triglycerides to total lipids ratio in medium HDL (%)	735	-0.04	-0.10	0.03	0.307	735	-0.05	-0.11	0.02	0.176	735	0.00	-0.07	0.07	0.927
Phospholipids to total lipds ratio in small HDL (%)	735	-0.06	-0.12	0.00	0.063	735	0.00	-0.06	0.06	0.950	735	0.06	-0.01	0.13	0.082
Total cholesterol to total lipids ratio in small HDL (%)	735	0.06	0.00	0.12	0.054	735	0.01	-0.06	0.07	0.848	735	-0.06	-0.13	0.01	0.107
Cholesterol esters to total lipids ratio in small HDL (%)	735	0.06	0.00	0.12	0.052	735	0.00	-0.06	0.06	0.990	735	-0.07	-0.14	0.00	0.052
Free cholesterol to total lipids ratio in small HDL (%)	735	-0.03	-0.10	0.03	0.313	735	0.05	-0.01	0.11	0.117	735	0.11	0.03	0.19	0.010
Triglycerides to total lipids ratio in small HDL (%)	735	-0.03	-0.10	0.04	0.355	735	-0.03	-0.10	0.03	0.354	735	0.02	-0.05	0.09	0.538
Mean diameter for VLDL particles (nm)	735	-0.03	-0.10	0.04	0.428	735	0.00	-0.07	0.07	0.965	735	0.04	-0.03	0.11	0.303
Mean diameter for LDL particles (nm)	735	-0.03	-0.08	0.03	0.348	735	-0.01	-0.06	0.05	0.780	735	0.02	-0.05	0.08	0.603
Mean diameter for HDL particles (nm)	735	0.01	-0.05	0.08	0.714	735	0.01	-0.06	0.08	0.840	735	-0.01	-0.07	0.06	0.859
Serum total cholesterol (mmol/l)	735	0.02	-0.03	0.08	0.417	735	-0.03	-0.09	0.03	0.289	735	-0.07	-0.13	-0.01	0.028
Total cholesterol in VLDL (mmol/l)	735	-0.02	-0.07	0.03	0.487	735	-0.04	-0.09	0.01	0.133	735	-0.01	-0.07	0.05	0.757
Remnant cholesterol (non-HDL, non-LDL -cholesterol) (mmol/l)	735	0.00	-0.05	0.05	0.947	735	-0.04	-0.09	0.00	0.073	735	-0.04	-0.10	0.01	0.139
Total cholesterol in LDL (mmol/l)	735	0.02	-0.04	0.08	0.499	735	-0.04	-0.09	0.02	0.216	735	-0.07	-0.14	-0.01	0.032
Total cholesterol in HDL (mmol/l)	735	0.04	-0.03	0.10	0.288	735	0.01	-0.06	0.08	0.744	735	-0.03	-0.09	0.03	0.343
Total cholesterol in HDL2 (mmol/l)	735	0.04	-0.03	0.10	0.247	735	0.01	-0.05	0.08	0.695	735	-0.04	-0.10	0.03	0.271
Total cholesterol in HDL3 (mmol/l)	735	0.03	-0.04	0.09	0.408	735	0.01	-0.06	0.07	0.833	735	-0.02	-0.08	0.04	0.566
Esterified cholesterol (mmol/l)	735	0.03	-0.03	0.09	0.288	735	-0.03	-0.09	0.03	0.283	735	-0.08	-0.14	-0.02	0.008
Free cholesterol (mmol/l)	735	0.00	-0.05	0.06	0.873	735	-0.03	-0.09	0.03	0.342	735	-0.03	-0.09	0.03	0.343
Serum total triglycerides (mmol/l)	735	-0.03	-0.09	0.03	0.332	735	-0.03	-0.09	0.03	0.398	735	0.03	-0.04	0.10	0.429
Triglycerides in VLDL (mmol/l)	735	-0.03	-0.09	0.03	0.287	735	-0.02	-0.08	0.04	0.517	735	0.04	-0.03	0.11	0.290
Triglycerides in LDL (mmol/l)	735	0.00	-0.06	0.07	0.904	735	-0.02	-0.09	0.04	0.492	735	-0.03	-0.10	0.05	0.485
Triglycerides in HDL (mmol/l)	735	-0.03	-0.10	0.03	0.349	735	-0.04	-0.10	0.01	0.141	735	0.00	-0.07	0.07	0.946
Diacylglycerol (mmol/l)	698	0.05	-0.02	0.12	0.131	698	0.05	-0.03	0.12	0.210	698	0.01	-0.07	0.08	0.840
Ratio of diacylglycerol to triglycerides	698	0.09	0.02	0.17	0.018	698	0.07	-0.02	0.16	0.113	698	-0.03	-0.10	0.05	0.478
Total phosphoglycerides (mmol/l)	735	0.01	-0.05	0.08	0.698	735	0.01	-0.06	0.07	0.814	735	0.01	-0.06	0.08	0.816
Ratio of triglycerides to phosphoglycerides	735	-0.03	-0.09	0.04	0.401	735	-0.02	-0.08	0.04	0.542	735	0.03	-0.04	0.10	0.416
Phosphatidylcholine and other cholines (mmol/l)	723	-0.02	-0.09	0.04	0.533	723	-0.04	-0.11	0.03	0.241	723	0.00	-0.07	0.07	0.963
Total cholines (mmol/l)	733	0.01	-0.05	0.07	0.789	733	0.00	-0.07	0.07	0.976	733	0.00	-0.07	0.07	0.910
Apolipoprotein A-I (g/l)	735	0.03	-0.03	0.09	0.360	735	0.00	-0.07	0.07	0.998	735	-0.03	-0.09	0.03	0.266
Apolipoprotein B (g/l)	735	-0.01	-0.07	0.04	0.624	735	-0.04	-0.09	0.01	0.161	735	-0.02	-0.08	0.05	0.609
Ratio of apolipoprotein B to apolipoprotein A-I	735	-0.03	-0.08	0.03	0.398	735	-0.03	-0.09	0.02	0.226	735	0.00	-0.06	0.07	0.963
Total fatty acids (mmol/l)	735	0.00	-0.06	0.05	0.918	735	0.00	-0.06	0.05	0.890	735	0.02	-0.04	0.09	0.478
Estimated description of fatty acid chain length, not actual carbon number	732	0.00	-0.06	0.06	0.989	732	-0.05	-0.11	0.02	0.142	732	-0.06	-0.14	0.01	0.104
Estimated degree of unsaturation	734	0.01	-0.06	0.08	0.758	734	-0.07	-0.14	0.00	0.038	734	-0.11	-0.18	-0.03	0.007
22:6, docosahexaenoic acid (mmol/l)	735	0.01	-0.05	0.07	0.669	735	-0.02	-0.09	0.04	0.459	735	-0.07	-0.13	-0.01	0.031
18:2, linoleic acid (mmol/l)	733	0.00	-0.05	0.06	0.885	733	-0.02	-0.08	0.04	0.501	733	0.00	-0.07	0.06	0.951
Conjugated linoleic acid (mmol/l)	734	0.03	-0.05	0.11	0.459	734	0.02	-0.06	0.09	0.671	734	-0.03	-0.10	0.04	0.392
Omega-3 fatty acids (mmol/l)	733	0.01	-0.05	0.07	0.764	733	0.00	-0.08	0.07	0.903	733	-0.05	-0.11	0.02	0.150
Omega-6 fatty acids (mmol/l)	735	0.01	-0.04	0.07	0.659	735	-0.02	-0.07	0.04	0.601	735	-0.01	-0.08	0.05	0.663
Polyunsaturated fatty acids (mmol/l)	733	0.01	-0.05	0.07	0.709	733	-0.02	-0.07	0.04	0.593	733	-0.02	-0.08	0.05	0.568
Monounsaturated fatty acids; 16:1, 18:1 (mmol/l)	734	-0.03	-0.09	0.04	0.408	734	-0.02	-0.08	0.04	0.452	734	0.04	-0.03	0.10	0.294
Saturated fatty acids (mmol/l)	733	0.00	-0.06	0.06	0.974	733	0.01	-0.05	0.07	0.726	733	0.04	-0.03	0.10	0.302
Ratio of 22:6 docosahexaenoic acid to total fatty acids (%)	735	0.02	-0.05	0.08	0.649	735	-0.03	-0.10	0.03	0.327	735	-0.10	-0.17	-0.03	0.006
Ratio of 18:2 linoleic acid to total fatty acids (%)	733	0.01	-0.07	0.08	0.870	733	-0.03	-0.10	0.05	0.484	733	-0.02	-0.10	0.05	0.509
Ratio of conjugated linoleic acid to total fatty acids (%)	734	0.04	-0.04	0.12	0.302	734	0.03	-0.05	0.10	0.462	734	-0.03	-0.10	0.04	0.362
Ratio of omega-3 fatty acids to total fatty acids (%)	733	0.02	-0.05	0.09	0.545	733	0.00	-0.08	0.07	0.976	733	-0.08	-0.16	-0.01	0.020
Ratio of omega-6 fatty acids to total fatty acids (%)	735	0.02	-0.06	0.10	0.598	735	-0.02	-0.09	0.06	0.654	735	-0.04	-0.12	0.03	0.285
Ratio of polyunsaturated fatty acids to total fatty acids (%)	733	0.02	-0.06	0.09	0.659	733	-0.02	-0.10	0.05	0.560	733	-0.06	-0.13	0.02	0.152
Ratio of monounsaturated fatty acids to total fatty acids (%)	734	-0.03	-0.11	0.04	0.380	734	-0.03	-0.11	0.04	0.396	734	0.02	-0.05	0.10	0.548
Ratio of saturated fatty acids to total fatty acids (%)	733	0.02	-0.05	0.09	0.635	733	0.06	-0.01	0.13	0.111	733	0.03	-0.05	0.10	0.493
Glucose (mmol/l)	728	-0.02	-0.08	0.05	0.599	728	0.00	-0.07	0.07	0.960	728	0.01	-0.07	0.09	0.794
Lactate (mmol/l)	734	0.00	-0.08	0.08	0.959	734	0.02	-0.06	0.10	0.604	734	-0.01	-0.09	0.07	0.763
Pyruvate (mmol/l)	733	0.04	-0.04	0.12	0.361	733	0.06	-0.02	0.14	0.139	733	-0.02	-0.10	0.06	0.622

Citrate (mmol/l)	731	0.05	-0.03	0.12	0.208	731	0.04	-0.04	0.12	0.363	731	0.02	-0.06	0.09	0.690
Alanine (mmol/l)	735	-0.02	-0.10	0.06	0.660	735	0.06	-0.01	0.14	0.107	735	0.07	-0.01	0.15	0.096
Glutamine (mmol/l)	735	-0.01	-0.08	0.05	0.693	735	0.03	-0.04	0.10	0.361	735	0.02	-0.04	0.09	0.501
Histidine (mmol/l)	691	0.01	-0.06	0.09	0.714	691	0.02	-0.05	0.10	0.524	691	0.03	-0.05	0.11	0.519
Isoleucine (mmol/l)	735	-0.02	-0.10	0.05	0.563	735	0.01	-0.07	0.08	0.869	735	0.03	-0.05	0.10	0.485
Leucine (mmol/l)	735	0.02	-0.05	0.10	0.564	735	0.03	-0.04	0.10	0.435	735	0.00	-0.06	0.07	0.886
Valine (mmol/l)	735	0.00	-0.07	0.07	0.989	735	0.01	-0.06	0.08	0.803	735	0.00	-0.08	0.07	0.963
Phenylalanine (mmol/l)	734	0.03	-0.04	0.09	0.424	734	0.00	-0.07	0.07	0.994	734	-0.05	-0.12	0.02	0.191
Tyrosine (mmol/l)	728	-0.02	-0.10	0.06	0.691	728	-0.04	-0.12	0.04	0.291	728	-0.06	-0.14	0.02	0.115
Acetate (mmol/l)	735	0.00	-0.07	0.07	0.972	735	0.04	-0.03	0.10	0.286	735	0.05	-0.03	0.14	0.185
Acetoacetate (mmol/l)	735	0.09	-0.06	0.25	0.218	735	0.07	-0.06	0.21	0.306	735	-0.04	-0.12	0.05	0.384
3-hydroxybutyrate (mmol/l)	734	0.03	-0.04	0.09	0.415	734	0.00	-0.06	0.06	0.976	734	-0.03	-0.11	0.05	0.423
Creatinine (mmol/l)	735	-0.04	-0.10	0.03	0.260	735	0.01	-0.05	0.08	0.686	735	0.09	0.02	0.15	0.012
Albumin (signal area)	735	-0.03	-0.10	0.04	0.373	735	0.01	-0.06	0.08	0.795	735	0.02	-0.06	0.10	0.583
Glycoprotein acetyls, mainly a1-acid glycoprotein (mmol/l)	735	-0.03	-0.09	0.04	0.420	735	-0.05	-0.12	0.01	0.099	735	-0.05	-0.13	0.02	0.184

Complete case sample

	**Change in CPM from age 12y-15y (per SD-unit increase)**					**Change in MVPA from age 12y-15y (per SD-unit increase)**					**Change in SED from age 12y-15y (per SD-unit increase)**				
	Adj. for age, sex, ethnicity, maternal education, wear time change, wear month, FMI change, metabolic trait at 8y					*Adj. for age, sex, ethnicity, maternal education, wear time change, wear month, SED change, FMI change, metabolic trait at 8y*					*Adj. for age, sex, ethnicity, maternal education, wear time change, wear month, MVPA change, FMI change, metabolic trait at 8y*				
**Standardised outcome at age 15y**	**N**	**Beta**	**LCL**	**UCL**	**P-value**	**N**	**Beta**	**LCL**	**UCL**	**P-value**	**N**	**Beta**	**LCL**	**UCL**	**P-value**
Systolic blood pressure (mmHg)	520	0.02	-0.06	0.10	0.603	520	0.08	0.00	0.17	0.042	520	0.06	-0.02	0.15	0.148
Diastolic blood pressure (mmHg)	520	0.03	-0.05	0.11	0.499	520	0.06	-0.03	0.14	0.195	520	0.06	-0.03	0.15	0.165
Concentration of chylomicrons and extremely large VLDL particles (mol/l)	520	-0.02	-0.08	0.05	0.628	520	0.00	-0.07	0.06	0.917	520	0.03	-0.04	0.10	0.431
Total lipids in chylomicrons and extremely large VLDL (mmol/l)	520	-0.02	-0.08	0.05	0.575	520	-0.01	-0.07	0.06	0.830	520	0.03	-0.05	0.10	0.501
Phospholipids in chylomicrons and extremely large VLDL (mmol/l)	520	-0.02	-0.09	0.04	0.533	520	-0.01	-0.07	0.05	0.773	520	0.02	-0.05	0.10	0.509
Total cholesterol in chylomicrons and extremely large VLDL (mmol/l)	520	-0.01	-0.07	0.06	0.792	520	0.00	-0.06	0.06	0.972	520	0.02	-0.05	0.10	0.568
Cholesterol esters in chylomicrons and extremely large VLDL (mmol/l)	520	0.00	-0.06	0.06	0.997	520	0.00	-0.06	0.07	0.907	520	0.02	-0.06	0.09	0.645
Free cholesterol in chylomicrons and extremely large VLDL (mmol/l)	520	-0.02	-0.08	0.05	0.601	520	-0.01	-0.07	0.06	0.798	520	0.02	-0.05	0.10	0.570
Triglycerides in chylomicrons and extremely large VLDL (mmol/l)	520	-0.02	-0.09	0.04	0.540	520	-0.01	-0.07	0.06	0.804	520	0.03	-0.05	0.10	0.494
Concentration of very large VLDL particles (mol/l)	520	-0.02	-0.09	0.04	0.462	520	0.00	-0.07	0.06	0.933	520	0.04	-0.03	0.12	0.259
Total lipids in very large VLDL (mmol/l)	520	-0.03	-0.09	0.04	0.451	520	0.00	-0.07	0.06	0.898	520	0.04	-0.03	0.12	0.290
Phospholipids in very large VLDL (mmol/l)	520	-0.02	-0.09	0.04	0.487	520	-0.01	-0.07	0.06	0.834	520	0.03	-0.04	0.11	0.393
Total cholesterol in very large VLDL (mmol/l)	520	-0.01	-0.08	0.05	0.652	520	0.00	-0.07	0.06	0.938	520	0.03	-0.05	0.10	0.474
Cholesterol esters in very large VLDL (mmol/l)	520	-0.01	-0.08	0.05	0.685	520	0.00	-0.06	0.06	0.997	520	0.03	-0.05	0.10	0.451
Free cholesterol in very large VLDL (mmol/l)	520	-0.02	-0.08	0.05	0.620	520	-0.01	-0.07	0.06	0.857	520	0.02	-0.05	0.10	0.518
Triglycerides in very large VLDL (mmol/l)	520	-0.03	-0.10	0.04	0.393	520	0.00	-0.07	0.06	0.902	520	0.05	-0.03	0.12	0.230
Concentration of large VLDL particles (mol/l)	520	-0.03	-0.09	0.04	0.465	520	0.00	-0.07	0.06	0.947	520	0.04	-0.03	0.12	0.258
Total lipids in large VLDL (mmol/l)	520	-0.03	-0.09	0.04	0.473	520	0.00	-0.07	0.06	0.932	520	0.04	-0.04	0.12	0.282
Phospholipids in large VLDL (mmol/l)	520	-0.02	-0.09	0.05	0.500	520	0.00	-0.07	0.06	0.931	520	0.04	-0.04	0.12	0.303
Total cholesterol in large VLDL (mmol/l)	520	-0.02	-0.09	0.05	0.574	520	0.00	-0.06	0.07	0.990	520	0.04	-0.04	0.12	0.300
Cholesterol esters in large VLDL (mmol/l)	520	-0.01	-0.08	0.06	0.710	520	0.00	-0.06	0.07	0.942	520	0.03	-0.04	0.11	0.390
Free cholesterol in large VLDL (mmol/l)	520	-0.03	-0.09	0.04	0.460	520	0.00	-0.07	0.06	0.942	520	0.04	-0.03	0.12	0.249
Triglycerides in large VLDL (mmol/l)	520	-0.03	-0.10	0.04	0.433	520	0.00	-0.07	0.06	0.903	520	0.04	-0.03	0.12	0.275
Concentration of medium VLDL particles (mol/l)	520	-0.02	-0.09	0.04	0.491	520	0.00	-0.07	0.06	0.940	520	0.04	-0.04	0.12	0.313
Total lipids in medium VLDL (mmol/l)	520	-0.02	-0.09	0.05	0.556	520	0.00	-0.07	0.06	0.954	520	0.04	-0.04	0.11	0.369
Phospholipids in medium VLDL (mmol/l)	520	-0.02	-0.09	0.05	0.561	520	0.00	-0.07	0.07	0.996	520	0.04	-0.04	0.12	0.321
Total cholesterol in medium VLDL (mmol/l)	520	0.00	-0.07	0.06	0.936	520	0.00	-0.06	0.07	0.924	520	0.02	-0.06	0.10	0.620
Cholesterol esters in medium VLDL (mmol/l)	520	0.01	-0.05	0.08	0.716	520	0.00	-0.06	0.07	0.907	520	0.00	-0.08	0.08	0.991
Free cholesterol in medium VLDL (mmol/l)	520	-0.02	-0.09	0.05	0.566	520	0.00	-0.07	0.07	0.996	520	0.04	-0.04	0.12	0.310
Triglycerides in medium VLDL (mmol/l)	520	-0.03	-0.10	0.04	0.422	520	-0.01	-0.07	0.06	0.876	520	0.04	-0.04	0.12	0.322
Concentration of small VLDL particles (mol/l)	520	-0.01	-0.07	0.06	0.857	520	0.00	-0.06	0.06	0.981	520	0.02	-0.05	0.10	0.557

Total lipids in small VLDL (mmol/l)	520	0.00	-0.06	0.07	0.914	520	0.00	-0.06	0.06	0.968	520	0.00	-0.07	0.08	0.925
Phospholipids in small VLDL (mmol/l)	520	0.01	-0.05	0.08	0.739	520	0.00	-0.06	0.06	0.989	520	0.00	-0.08	0.07	0.927
Total cholesterol in small VLDL (mmol/l)	520	0.03	-0.03	0.09	0.320	520	-0.01	-0.06	0.04	0.773	520	-0.05	-0.12	0.02	0.199
Cholesterol esters in small VLDL (mmol/l)	520	0.03	-0.02	0.09	0.231	520	-0.02	-0.07	0.03	0.508	520	-0.07	-0.14	0.00	0.050
Free cholesterol in small VLDL (mmol/l)	520	0.01	-0.05	0.08	0.714	520	0.01	-0.05	0.06	0.846	520	0.01	-0.07	0.08	0.888
Triglycerides in small VLDL (mmol/l)	520	-0.02	-0.09	0.05	0.606	520	0.00	-0.07	0.06	0.932	520	0.03	-0.05	0.11	0.407
Concentration of very small VLDL particles (mol/l)	520	0.04	-0.01	0.10	0.129	520	0.00	-0.05	0.05	0.954	520	-0.06	-0.13	0.02	0.119
Total lipids in very small VLDL (mmol/l)	520	0.04	-0.01	0.10	0.142	520	-0.02	-0.07	0.03	0.538	520	-0.08	-0.15	-0.01	0.023
Phospholipids in very small VLDL (mmol/l)	520	0.05	-0.01	0.11	0.113	520	0.00	-0.06	0.05	0.868	520	-0.07	-0.14	0.00	0.051
Total cholesterol in very small VLDL (mmol/l)	520	0.03	-0.03	0.10	0.289	520	-0.03	-0.09	0.03	0.285	520	-0.09	-0.17	-0.02	0.019
Cholesterol esters in very small VLDL (mmol/l)	520	0.03	-0.03	0.10	0.314	520	-0.03	-0.09	0.02	0.234	520	-0.10	-0.18	-0.03	0.009
Free cholesterol in very small VLDL (mmol/l)	520	0.03	-0.03	0.10	0.303	520	-0.02	-0.08	0.04	0.577	520	-0.06	-0.14	0.03	0.186
Triglycerides in very small VLDL (mmol/l)	520	0.01	-0.06	0.08	0.764	520	0.01	-0.05	0.07	0.807	520	0.01	-0.06	0.09	0.733
Concentration of IDL particles (mol/l)	520	0.04	-0.02	0.11	0.190	520	0.01	-0.06	0.07	0.806	520	-0.05	-0.12	0.03	0.236
Total lipids in IDL (mmol/l)	520	0.05	-0.01	0.12	0.127	520	0.00	-0.06	0.07	0.920	520	-0.06	-0.14	0.01	0.097
Phospholipids in IDL (mmol/l)	520	0.05	-0.02	0.12	0.136	520	0.01	-0.06	0.08	0.813	520	-0.06	-0.14	0.02	0.158
Total cholesterol in IDL (mmol/l)	520	0.05	-0.02	0.11	0.140	520	0.00	-0.06	0.06	0.961	520	-0.07	-0.14	0.01	0.077
Cholesterol esters in IDL (mmol/l)	520	0.05	-0.02	0.11	0.155	520	0.00	-0.06	0.05	0.908	520	-0.07	-0.14	0.01	0.082
Free cholesterol in IDL (mmol/l)	520	0.05	-0.02	0.12	0.137	520	0.00	-0.06	0.07	0.910	520	-0.07	-0.15	0.01	0.110
Triglycerides in IDL (mmol/l)	520	0.03	-0.05	0.10	0.441	520	0.01	-0.06	0.08	0.767	520	-0.01	-0.09	0.07	0.776
Concentration of large LDL particles (mol/l)	520	0.05	-0.02	0.12	0.147	520	0.01	-0.06	0.08	0.707	520	-0.04	-0.12	0.03	0.266
Total lipids in large LDL (mmol/l)	520	0.05	-0.01	0.12	0.115	520	0.01	-0.06	0.08	0.826	520	-0.06	-0.14	0.02	0.126
Phospholipids in large LDL (mmol/l)	520	0.06	-0.01	0.13	0.092	520	0.01	-0.06	0.08	0.797	520	-0.06	-0.14	0.01	0.106
Total cholesterol in large LDL (mmol/l)	520	0.05	-0.01	0.12	0.113	520	0.01	-0.06	0.07	0.859	520	-0.06	-0.14	0.01	0.106
Cholesterol esters in large LDL (mmol/l)	520	0.05	-0.01	0.12	0.113	520	0.01	-0.06	0.07	0.853	520	-0.06	-0.14	0.01	0.107
Free cholesterol in large LDL (mmol/l)	520	0.06	-0.01	0.13	0.117	520	0.01	-0.06	0.07	0.880	520	-0.07	-0.15	0.01	0.107
Triglycerides in large LDL (mmol/l)	520	0.03	-0.05	0.11	0.518	520	0.00	-0.07	0.08	0.925	520	-0.02	-0.10	0.06	0.634
Concentration of medium LDL particles (mol/l)	520	0.05	-0.02	0.12	0.161	520	0.02	-0.05	0.09	0.621	520	-0.03	-0.11	0.04	0.396
Total lipids in medium LDL (mmol/l)	520	0.05	-0.02	0.12	0.127	520	0.01	-0.06	0.08	0.776	520	-0.05	-0.13	0.02	0.178
Phospholipids in medium LDL (mmol/l)	520	0.05	-0.01	0.12	0.126	520	0.00	-0.06	0.07	0.886	520	-0.05	-0.12	0.02	0.130
Total cholesterol in medium LDL (mmol/l)	520	0.05	-0.02	0.12	0.125	520	0.01	-0.06	0.08	0.789	520	-0.06	-0.14	0.02	0.158
Cholesterol esters in medium LDL (mmol/l)	520	0.05	-0.02	0.12	0.124	520	0.01	-0.06	0.08	0.786	520	-0.06	-0.14	0.02	0.164
Free cholesterol in medium LDL (mmol/l)	520	0.05	-0.02	0.12	0.160	520	0.01	-0.06	0.07	0.839	520	-0.05	-0.12	0.03	0.222
Triglycerides in medium LDL (mmol/l)	520	0.03	-0.05	0.11	0.439	520	0.01	-0.07	0.09	0.886	520	-0.03	-0.12	0.06	0.564
Concentration of small LDL particles (mol/l)	520	0.04	-0.03	0.11	0.242	520	0.01	-0.06	0.09	0.697	520	-0.03	-0.10	0.05	0.495
Total lipids in small LDL (mmol/l)	520	0.05	-0.02	0.12	0.151	520	0.01	-0.06	0.08	0.798	520	-0.05	-0.13	0.03	0.204
Phospholipids in small LDL (mmol/l)	520	0.04	-0.03	0.11	0.224	520	0.01	-0.06	0.08	0.835	520	-0.04	-0.11	0.03	0.289
Total cholesterol in small LDL (mmol/l)	520	0.05	-0.02	0.12	0.132	520	0.01	-0.06	0.08	0.840	520	-0.06	-0.14	0.02	0.151
Cholesterol esters in small LDL (mmol/l)	520	0.05	-0.02	0.13	0.137	520	0.01	-0.06	0.08	0.789	520	-0.06	-0.14	0.03	0.194
Free cholesterol in small LDL (mmol/l)	520	0.05	-0.02	0.11	0.192	520	-0.01	-0.07	0.06	0.863	520	-0.06	-0.13	0.01	0.107
Triglycerides in small LDL (mmol/l)	520	0.02	-0.06	0.10	0.588	520	0.01	-0.07	0.08	0.884	520	-0.01	-0.09	0.07	0.854
Concentration of very large HDL particles (mol/l)	520	0.01	-0.08	0.09	0.876	520	0.01	-0.08	0.10	0.892	520	0.00	-0.07	0.08	0.905
Total lipids in very large HDL (mmol/l)	520	0.00	-0.08	0.09	0.965	520	0.00	-0.09	0.10	0.942	520	0.01	-0.07	0.09	0.855
Phospholipids in very large HDL (mmol/l)	520	0.01	-0.07	0.09	0.816	520	0.01	-0.08	0.10	0.836	520	0.00	-0.07	0.08	0.922
Total cholesterol in very large HDL (mmol/l)	520	-0.01	-0.09	0.08	0.874	520	0.00	-0.10	0.09	0.922	520	0.01	-0.07	0.09	0.827
Cholesterol esters in very large HDL (mmol/l)	520	-0.01	-0.10	0.07	0.795	520	-0.01	-0.11	0.08	0.814	520	0.01	-0.08	0.09	0.873
Free cholesterol in very large HDL (mmol/l)	520	0.00	-0.08	0.09	0.927	520	0.01	-0.08	0.10	0.803	520	0.01	-0.07	0.10	0.721
Triglycerides in very large HDL (mmol/l)	520	-0.01	-0.10	0.08	0.792	520	0.00	-0.08	0.08	0.958	520	0.02	-0.07	0.10	0.680
Concentration of large HDL particles (mol/l)	520	0.02	-0.06	0.10	0.693	520	0.01	-0.08	0.09	0.836	520	-0.01	-0.08	0.07	0.868
Total lipids in large HDL (mmol/l)	520	0.01	-0.07	0.09	0.728	520	0.01	-0.07	0.09	0.824	520	0.00	-0.08	0.07	0.924
Phospholipids in large HDL (mmol/l)	520	0.02	-0.06	0.10	0.645	520	0.01	-0.08	0.09	0.839	520	-0.01	-0.09	0.07	0.782
Total cholesterol in large HDL (mmol/l)	520	0.01	-0.07	0.09	0.800	520	0.01	-0.07	0.09	0.800	520	0.00	-0.07	0.08	0.926
Cholesterol esters in large HDL (mmol/l)	520	0.01	-0.07	0.09	0.806	520	0.01	-0.07	0.09	0.798	520	0.00	-0.07	0.08	0.907
Free cholesterol in large HDL (mmol/l)	520	0.01	-0.07	0.09	0.777	520	0.01	-0.07	0.09	0.810	520	0.00	-0.08	0.08	0.999
Triglycerides in large HDL (mmol/l)	520	0.01	-0.07	0.09	0.800	520	0.00	-0.08	0.07	0.912	520	-0.04	-0.12	0.05	0.397
Concentration of medium HDL particles (mol/l)	520	0.03	-0.05	0.11	0.429	520	0.01	-0.07	0.08	0.894	520	-0.03	-0.11	0.06	0.535
Total lipids in medium HDL (mmol/l)	520	0.03	-0.05	0.11	0.491	520	0.00	-0.07	0.08	0.919	520	-0.02	-0.11	0.06	0.583

Phospholipids in medium HDL (mmol/l)	520	0.03	-0.05	0.11	0.428	520	0.00	-0.08	0.08	0.978	520	-0.03	-0.12	0.05	0.405
Total cholesterol in medium HDL (mmol/l)	520	0.02	-0.06	0.10	0.648	520	0.01	-0.07	0.09	0.793	520	0.00	-0.09	0.08	0.943
Cholesterol esters in medium HDL (mmol/l)	520	0.02	-0.06	0.09	0.697	520	0.01	-0.06	0.09	0.730	520	0.01	-0.09	0.10	0.890
Free cholesterol in medium HDL (mmol/l)	520	0.03	-0.05	0.11	0.461	520	0.00	-0.08	0.08	0.928	520	-0.04	-0.12	0.03	0.274
Triglycerides in medium HDL (mmol/l)	520	0.02	-0.05	0.09	0.631	520	-0.01	-0.07	0.06	0.854	520	-0.03	-0.11	0.05	0.459
Concentration of small HDL particles (mol/l)	520	0.02	-0.06	0.09	0.649	520	-0.01	-0.08	0.06	0.858	520	-0.02	-0.10	0.05	0.532
Total lipids in small HDL (mmol/l)	520	0.04	-0.04	0.11	0.320	520	0.00	-0.07	0.07	0.957	520	-0.04	-0.11	0.04	0.339
Phospholipids in small HDL (mmol/l)	520	0.00	-0.08	0.07	0.962	520	-0.01	-0.08	0.06	0.826	520	-0.01	-0.08	0.07	0.878
Total cholesterol in small HDL (mmol/l)	520	0.07	0.00	0.15	0.066	520	0.02	-0.06	0.09	0.661	520	-0.05	-0.13	0.03	0.190
Cholesterol esters in small HDL (mmol/l)	520	0.08	0.01	0.15	0.036	520	0.02	-0.06	0.09	0.640	520	-0.06	-0.14	0.02	0.117
Free cholesterol in small HDL (mmol/l)	520	0.01	-0.07	0.09	0.834	520	0.00	-0.07	0.08	0.895	520	0.01	-0.07	0.09	0.868
Triglycerides in small HDL (mmol/l)	520	0.00	-0.08	0.08	0.978	520	-0.01	-0.08	0.05	0.693	520	-0.01	-0.09	0.08	0.902
Phospholipids to total lipids ratio in chylomicrons and extremely large VLDL (%)	520	0.00	-0.08	0.08	0.999	520	-0.03	-0.11	0.05	0.413	520	-0.03	-0.12	0.05	0.441
Total cholesterol to total lipids ratio in chylomicrons and extremely large VLDL (%)	520	0.06	-0.02	0.14	0.142	520	0.04	-0.04	0.11	0.340	520	-0.02	-0.12	0.07	0.594
Cholesterol esters to total lipids ratio in chylomicrons and extremely large VLDL (%)	520	0.05	-0.02	0.13	0.171	520	0.04	-0.03	0.12	0.279	520	-0.01	-0.10	0.08	0.840
Free cholesterol to total lipids ratio in chylomicrons and extremely large VLDL (%)	520	0.02	-0.06	0.11	0.556	520	0.00	-0.08	0.08	1.000	520	-0.03	-0.12	0.07	0.554
Triglycerides to total lipids ratio in chylomicrons and extremely large VLDL (%)	520	-0.04	-0.09	0.01	0.144	520	-0.02	-0.07	0.03	0.478	520	0.03	-0.04	0.09	0.424
Phospholipids to total lipds ratio in very large VLDL (%)	520	0.03	-0.05	0.11	0.430	520	0.02	-0.07	0.10	0.700	520	-0.02	-0.11	0.07	0.671
Total cholesterol to total lipids ratio in very large VLDL (%)	520	-0.01	-0.09	0.08	0.845	520	0.00	-0.10	0.09	0.950	520	0.04	-0.04	0.13	0.310
Cholesterol esters to total lipids ratio in very large VLDL (%)	520	-0.02	-0.12	0.07	0.668	520	-0.02	-0.12	0.09	0.769	520	0.05	-0.04	0.13	0.312
Free cholesterol to total lipids ratio in very large VLDL (%)	520	0.01	-0.09	0.10	0.873	520	0.01	-0.11	0.13	0.834	520	0.05	-0.07	0.16	0.418
Triglycerides to total lipids ratio in very large VLDL (%)	520	0.00	-0.10	0.09	0.937	520	0.00	-0.11	0.11	0.957	520	-0.05	-0.15	0.06	0.401
Phospholipids to total lipds ratio in large VLDL (%)	520	0.05	-0.04	0.15	0.256	520	0.03	-0.07	0.13	0.556	520	-0.05	-0.14	0.05	0.327
Total cholesterol to total lipids ratio in large VLDL (%)	520	0.03	-0.05	0.11	0.490	520	0.03	-0.05	0.11	0.503	520	0.03	-0.06	0.11	0.563
Cholesterol esters to total lipids ratio in large VLDL (%)	520	0.01	-0.03	0.05	0.611	520	0.02	-0.03	0.06	0.463	520	0.02	-0.02	0.07	0.309
Free cholesterol to total lipids ratio in large VLDL (%)	520	0.03	-0.06	0.11	0.530	520	0.02	-0.06	0.11	0.618	520	-0.02	-0.11	0.08	0.744
Triglycerides to total lipids ratio in large VLDL (%)	520	-0.01	-0.02	0.01	0.375	520	0.00	-0.02	0.01	0.443	520	0.00	-0.01	0.01	0.834
Phospholipids to total lipds ratio in medium VLDL (%)	520	0.01	-0.08	0.09	0.836	520	0.05	-0.07	0.17	0.418	520	0.09	-0.05	0.23	0.195
Total cholesterol to total lipids ratio in medium VLDL (%)	520	0.06	-0.01	0.12	0.075	520	0.03	-0.04	0.09	0.374	520	-0.02	-0.11	0.07	0.653
Cholesterol esters to total lipids ratio in medium VLDL (%)	520	0.06	-0.01	0.13	0.087	520	0.03	-0.04	0.09	0.451	520	-0.02	-0.11	0.06	0.604
Free cholesterol to total lipids ratio in medium VLDL (%)	520	0.02	-0.06	0.10	0.635	520	0.04	-0.05	0.13	0.357	520	0.04	-0.05	0.13	0.405
Triglycerides to total lipids ratio in medium VLDL (%)	520	-0.05	-0.12	0.01	0.100	520	-0.03	-0.11	0.04	0.343	520	0.01	-0.09	0.10	0.914
Phospholipids to total lipds ratio in small VLDL (%)	520	0.02	-0.05	0.09	0.517	520	0.01	-0.06	0.08	0.762	520	-0.02	-0.10	0.06	0.573
Total cholesterol to total lipids ratio in small VLDL (%)	520	0.03	-0.05	0.12	0.466	520	-0.02	-0.09	0.06	0.684	520	-0.05	-0.14	0.05	0.318
Cholesterol esters to total lipids ratio in small VLDL (%)	520	0.03	-0.06	0.11	0.521	520	-0.03	-0.10	0.05	0.517	520	-0.06	-0.15	0.03	0.185
Free cholesterol to total lipids ratio in small VLDL (%)	520	0.03	-0.05	0.12	0.417	520	0.05	-0.04	0.14	0.308	520	0.05	-0.09	0.20	0.459
Triglycerides to total lipids ratio in small VLDL (%)	520	-0.03	-0.12	0.05	0.433	520	0.01	-0.08	0.09	0.896	520	0.04	-0.05	0.13	0.417
Phospholipids to total lipds ratio in very small VLDL (%)	520	0.05	-0.03	0.12	0.253	520	0.02	-0.07	0.10	0.702	520	-0.02	-0.10	0.06	0.641
Total cholesterol to total lipids ratio in very small VLDL (%)	520	-0.02	-0.11	0.06	0.587	520	-0.03	-0.10	0.05	0.512	520	-0.03	-0.11	0.06	0.512
Cholesterol esters to total lipids ratio in very small VLDL (%)	520	-0.03	-0.11	0.06	0.568	520	-0.04	-0.12	0.04	0.350	520	-0.05	-0.13	0.04	0.281
Free cholesterol to total lipids ratio in very small VLDL (%)	520	0.00	-0.06	0.06	0.931	520	0.01	-0.05	0.07	0.712	520	0.03	-0.06	0.11	0.509
Triglycerides to total lipids ratio in very small VLDL (%)	520	-0.01	-0.09	0.07	0.875	520	0.02	-0.05	0.08	0.663	520	0.04	-0.04	0.12	0.314
Phospholipids to total lipds ratio in IDL (%)	520	0.03	-0.04	0.10	0.406	520	-0.01	-0.08	0.07	0.840	520	-0.10	-0.25	0.05	0.205
Total cholesterol to total lipids ratio in IDL (%)	520	-0.01	-0.08	0.07	0.847	520	0.00	-0.07	0.06	0.889	520	0.01	-0.08	0.10	0.777
Cholesterol esters to total lipids ratio in IDL (%)	520	-0.02	-0.10	0.05	0.521	520	0.00	-0.08	0.07	0.959	520	0.04	-0.06	0.14	0.450
Free cholesterol to total lipids ratio in IDL (%)	520	0.04	-0.04	0.11	0.319	520	0.00	-0.08	0.07	0.989	520	-0.05	-0.14	0.04	0.278
Triglycerides to total lipids ratio in IDL (%)	520	-0.01	-0.09	0.07	0.876	520	0.01	-0.06	0.08	0.819	520	0.03	-0.05	0.11	0.518
Phospholipids to total lipds ratio in large LDL (%)	520	-0.04	-0.10	0.03	0.271	520	0.01	-0.06	0.08	0.803	520	0.04	-0.02	0.10	0.227
Total cholesterol to total lipids ratio in large LDL (%)	520	0.03	-0.04	0.10	0.331	520	-0.01	-0.08	0.06	0.694	520	-0.05	-0.12	0.02	0.140
Cholesterol esters to total lipids ratio in large LDL (%)	520	0.04	-0.02	0.11	0.214	520	-0.01	-0.08	0.06	0.747	520	-0.06	-0.12	0.00	0.065
Free cholesterol to total lipids ratio in large LDL (%)	520	-0.02	-0.09	0.04	0.456	520	0.00	-0.06	0.06	0.982	520	0.01	-0.06	0.08	0.872
Triglycerides to total lipids ratio in large LDL (%)	520	-0.02	-0.10	0.06	0.653	520	0.00	-0.07	0.07	0.941	520	0.02	-0.06	0.10	0.642
Phospholipids to total lipds ratio in medium LDL (%)	520	-0.02	-0.04	0.01	0.252	520	0.00	-0.03	0.03	0.788	520	0.02	-0.01	0.05	0.234
Total cholesterol to total lipids ratio in medium LDL (%)	520	0.03	-0.04	0.11	0.362	520	-0.02	-0.09	0.06	0.674	520	-0.06	-0.13	0.02	0.163
Cholesterol esters to total lipids ratio in medium LDL (%)	520	0.05	-0.03	0.12	0.212	520	-0.01	-0.09	0.06	0.721	520	-0.07	-0.15	0.02	0.111
Free cholesterol to total lipids ratio in medium LDL (%)	520	-0.01	-0.04	0.01	0.152	520	0.00	-0.02	0.03	0.792	520	0.02	-0.01	0.04	0.151
Triglycerides to total lipids ratio in medium LDL (%)	520	0.02	-0.06	0.10	0.683	520	0.00	-0.07	0.07	0.964	520	-0.01	-0.11	0.08	0.754

Phospholipids to total lipds ratio in small LDL (%)	520	-0.03	-0.07	0.01	0.149	520	0.00	-0.04	0.05	0.855	520	0.04	-0.01	0.09	0.143
Total cholesterol to total lipids ratio in small LDL (%)	520	0.05	-0.03	0.12	0.231	520	-0.01	-0.09	0.06	0.704	520	-0.07	-0.15	0.02	0.111
Cholesterol esters to total lipids ratio in small LDL (%)	520	0.05	-0.02	0.13	0.159	520	-0.01	-0.09	0.07	0.850	520	-0.07	-0.16	0.02	0.133
Free cholesterol to total lipids ratio in small LDL (%)	520	-0.03	-0.07	0.01	0.176	520	0.00	-0.04	0.04	0.995	520	0.03	-0.02	0.07	0.294
Triglycerides to total lipids ratio in small LDL (%)	520	0.00	-0.07	0.08	0.920	520	0.00	-0.07	0.07	0.998	520	0.00	-0.08	0.09	0.911
Phospholipids to total lipds ratio in very large HDL (%)	520	0.03	-0.05	0.11	0.416	520	0.01	-0.07	0.09	0.833	520	-0.03	-0.10	0.04	0.437
Total cholesterol to total lipids ratio in very large HDL (%)	520	-0.03	-0.11	0.05	0.479	520	-0.01	-0.09	0.07	0.872	520	0.03	-0.05	0.10	0.466
Cholesterol esters to total lipids ratio in very large HDL (%)	520	-0.03	-0.11	0.05	0.476	520	-0.01	-0.09	0.07	0.798	520	0.02	-0.05	0.10	0.547
Free cholesterol to total lipids ratio in very large HDL (%)	520	0.02	-0.06	0.09	0.655	520	0.04	-0.04	0.12	0.349	520	0.03	-0.08	0.14	0.573
Triglycerides to total lipids ratio in very large HDL (%)	520	-0.03	-0.10	0.04	0.405	520	-0.01	-0.07	0.05	0.697	520	0.03	-0.05	0.11	0.470
Phospholipids to total lipds ratio in large HDL (%)	520	0.01	-0.05	0.08	0.677	520	-0.01	-0.07	0.05	0.670	520	-0.04	-0.11	0.04	0.346
Total cholesterol to total lipids ratio in large HDL (%)	520	0.00	-0.07	0.06	0.887	520	0.01	-0.05	0.07	0.669	520	0.03	-0.05	0.11	0.509
Cholesterol esters to total lipids ratio in large HDL (%)	520	-0.01	-0.08	0.06	0.795	520	0.01	-0.05	0.07	0.711	520	0.03	-0.05	0.11	0.453
Free cholesterol to total lipids ratio in large HDL (%)	520	0.01	-0.06	0.08	0.785	520	0.02	-0.05	0.09	0.577	520	0.01	-0.07	0.09	0.798
Triglycerides to total lipids ratio in large HDL (%)	520	-0.02	-0.09	0.05	0.549	520	-0.02	-0.09	0.05	0.588	520	0.00	-0.09	0.08	0.947
Phospholipids to total lipds ratio in medium HDL (%)	520	0.04	-0.03	0.12	0.264	520	-0.03	-0.10	0.04	0.397	520	-0.09	-0.18	0.00	0.061
Total cholesterol to total lipids ratio in medium HDL (%)	520	-0.04	-0.12	0.04	0.318	520	0.03	-0.04	0.10	0.381	520	0.09	-0.01	0.18	0.075
Cholesterol esters to total lipids ratio in medium HDL (%)	520	-0.05	-0.12	0.03	0.236	520	0.04	-0.03	0.11	0.276	520	0.12	-0.01	0.24	0.073
Free cholesterol to total lipids ratio in medium HDL (%)	520	0.03	-0.08	0.13	0.630	520	-0.03	-0.13	0.07	0.550	520	-0.12	-0.37	0.14	0.381
Triglycerides to total lipids ratio in medium HDL (%)	520	0.01	-0.07	0.08	0.829	520	-0.01	-0.07	0.06	0.883	520	-0.03	-0.11	0.06	0.545
Phospholipids to total lipds ratio in small HDL (%)	520	-0.08	-0.16	-0.01	0.026	520	-0.02	-0.09	0.06	0.631	520	0.07	-0.01	0.15	0.104
Total cholesterol to total lipids ratio in small HDL (%)	520	0.08	0.01	0.15	0.034	520	0.02	-0.05	0.10	0.541	520	-0.06	-0.14	0.03	0.175
Cholesterol esters to total lipids ratio in small HDL (%)	520	0.08	0.01	0.16	0.021	520	0.02	-0.05	0.09	0.579	520	-0.07	-0.15	0.01	0.091
Free cholesterol to total lipids ratio in small HDL (%)	520	-0.08	-0.15	-0.01	0.029	520	0.01	-0.05	0.08	0.739	520	0.12	0.02	0.22	0.021
Triglycerides to total lipids ratio in small HDL (%)	520	-0.02	-0.09	0.06	0.674	520	-0.02	-0.09	0.05	0.633	520	0.00	-0.08	0.09	0.915
Mean diameter for VLDL particles (nm)	520	-0.03	-0.11	0.05	0.487	520	-0.01	-0.08	0.07	0.898	520	0.03	-0.06	0.11	0.514
Mean diameter for LDL particles (nm)	520	-0.03	-0.09	0.04	0.457	520	-0.02	-0.08	0.05	0.599	520	-0.01	-0.09	0.07	0.884
Mean diameter for HDL particles (nm)	520	0.01	-0.07	0.09	0.849	520	0.00	-0.09	0.09	0.968	520	-0.01	-0.08	0.07	0.887
Serum total cholesterol (mmol/l)	520	0.05	-0.02	0.12	0.152	520	0.00	-0.07	0.07	0.910	520	-0.06	-0.13	0.01	0.108
Total cholesterol in VLDL (mmol/l)	520	0.01	-0.04	0.07	0.644	520	-0.01	-0.06	0.05	0.833	520	-0.02	-0.09	0.05	0.559
Remnant cholesterol (non-HDL, non-LDL -cholesterol) (mmol/l)	520	0.03	-0.02	0.09	0.218	520	0.00	-0.05	0.05	0.912	520	-0.05	-0.12	0.02	0.141
Total cholesterol in LDL (mmol/l)	520	0.05	-0.01	0.12	0.119	520	0.01	-0.06	0.07	0.830	520	-0.06	-0.14	0.02	0.128
Total cholesterol in HDL (mmol/l)	520	0.02	-0.06	0.10	0.591	520	0.00	-0.08	0.09	0.914	520	-0.01	-0.09	0.06	0.721
Total cholesterol in HDL2 (mmol/l)	520	0.02	-0.06	0.10	0.569	520	0.00	-0.08	0.09	0.922	520	-0.02	-0.09	0.06	0.678
Total cholesterol in HDL3 (mmol/l)	520	0.02	-0.06	0.10	0.634	520	0.01	-0.08	0.09	0.873	520	-0.01	-0.08	0.07	0.838
Esterified cholesterol (mmol/l)	520	0.05	-0.02	0.12	0.135	520	0.00	-0.07	0.07	0.974	520	-0.07	-0.14	0.00	0.061
Free cholesterol (mmol/l)	520	0.04	-0.03	0.11	0.241	520	0.01	-0.06	0.09	0.765	520	-0.03	-0.10	0.04	0.439
Serum total triglycerides (mmol/l)	520	-0.02	-0.09	0.06	0.668	520	0.00	-0.07	0.06	0.919	520	0.03	-0.05	0.11	0.451
Triglycerides in VLDL (mmol/l)	520	-0.02	-0.09	0.05	0.491	520	0.00	-0.07	0.06	0.913	520	0.04	-0.04	0.12	0.319
Triglycerides in LDL (mmol/l)	520	0.03	-0.05	0.11	0.500	520	0.00	-0.07	0.08	0.899	520	-0.02	-0.11	0.07	0.652
Triglycerides in HDL (mmol/l)	520	0.01	-0.07	0.08	0.889	520	-0.01	-0.08	0.06	0.791	520	-0.01	-0.10	0.07	0.731
Diacylglycerol (mmol/l)	520	0.07	-0.02	0.16	0.114	520	0.06	-0.03	0.15	0.198	520	-0.01	-0.10	0.07	0.787
Ratio of diacylglycerol to triglycerides	520	0.11	0.01	0.21	0.030	520	0.08	-0.03	0.19	0.158	520	-0.05	-0.14	0.04	0.285
Total phosphoglycerides (mmol/l)	520	0.04	-0.05	0.13	0.355	520	0.05	-0.04	0.14	0.255	520	0.02	-0.07	0.11	0.656
Ratio of triglycerides to phosphoglycerides	520	-0.03	-0.10	0.04	0.439	520	-0.01	-0.08	0.06	0.725	520	0.04	-0.04	0.12	0.334
Phosphatidylcholine and other cholines (mmol/l)	520	0.02	-0.06	0.10	0.618	520	-0.01	-0.09	0.08	0.869	520	-0.02	-0.10	0.07	0.722
Total cholines (mmol/l)	520	0.05	-0.04	0.13	0.264	520	0.04	-0.05	0.13	0.392	520	0.00	-0.09	0.08	0.924
Apolipoprotein A-I (g/l)	520	0.03	-0.05	0.11	0.504	520	0.01	-0.08	0.09	0.851	520	-0.01	-0.09	0.06	0.699
Apolipoprotein B (g/l)	520	0.02	-0.04	0.08	0.439	520	0.01	-0.05	0.06	0.773	520	-0.01	-0.08	0.05	0.686
Ratio of apolipoprotein B to apolipoprotein A-I	520	0.01	-0.05	0.07	0.727	520	0.01	-0.05	0.06	0.818	520	0.00	-0.07	0.07	0.896
Total fatty acids (mmol/l)	520	0.02	-0.06	0.09	0.663	520	0.03	-0.04	0.10	0.424	520	0.04	-0.04	0.11	0.333
Estimated description of fatty acid chain length, not actual carbon number	520	0.00	-0.07	0.07	0.903	520	-0.05	-0.12	0.02	0.151	520	-0.08	-0.15	0.00	0.051
Estimated degree of unsaturation	520	0.04	-0.05	0.13	0.346	520	-0.05	-0.12	0.03	0.203	520	-0.11	-0.19	-0.02	0.015
22:6, docosahexaenoic acid (mmol/l)	520	0.04	-0.03	0.12	0.293	520	0.01	-0.06	0.08	0.781	520	-0.06	-0.13	0.02	0.157
18:2, linoleic acid (mmol/l)	520	0.04	-0.03	0.11	0.223	520	0.03	-0.04	0.10	0.443	520	0.02	-0.06	0.10	0.584
Conjugated linoleic acid (mmol/l)	520	0.03	-0.06	0.11	0.564	520	0.02	-0.06	0.10	0.696	520	-0.02	-0.10	0.05	0.556
Omega-3 fatty acids (mmol/l)	520	0.00	-0.07	0.08	0.920	520	0.01	-0.07	0.09	0.835	520	-0.02	-0.09	0.06	0.674

Omega-6 fatty acids (mmol/l)	520	0.05	-0.02	0.12	0.191	520	0.03	-0.04	0.10	0.438	520	0.00	-0.07	0.08	0.912
Polyunsaturated fatty acids (mmol/l)	520	0.04	-0.03	0.11	0.236	520	0.03	-0.05	0.10	0.471	520	0.00	-0.07	0.08	0.961
Monounsaturated fatty acids; 16:1, 18:1 (mmol/l)	520	-0.02	-0.09	0.06	0.687	520	-0.01	-0.08	0.06	0.811	520	0.03	-0.05	0.11	0.523
Saturated fatty acids (mmol/l)	520	0.01	-0.06	0.09	0.728	520	0.05	-0.03	0.13	0.230	520	0.06	-0.02	0.14	0.129
Ratio of 22:6 docosahexaenoic acid to total fatty acids (%)	520	0.04	-0.05	0.12	0.386	520	-0.01	-0.09	0.07	0.796	520	-0.10	-0.19	-0.01	0.029
Ratio of 18:2 linoleic acid to total fatty acids (%)	520	0.05	-0.04	0.13	0.259	520	0.01	-0.07	0.09	0.792	520	0.00	-0.09	0.08	0.982
Ratio of conjugated linoleic acid to total fatty acids (%)	520	0.04	-0.05	0.13	0.389	520	0.03	-0.06	0.11	0.534	520	-0.02	-0.10	0.05	0.534
Ratio of omega-3 fatty acids to total fatty acids (%)	520	-0.01	-0.09	0.07	0.850	520	-0.01	-0.10	0.07	0.754	520	-0.06	-0.14	0.03	0.195
Ratio of omega-6 fatty acids to total fatty acids (%)	520	0.06	-0.03	0.15	0.195	520	0.01	-0.07	0.10	0.749	520	-0.03	-0.11	0.06	0.543
Ratio of polyunsaturated fatty acids to total fatty acids (%)	520	0.06	-0.04	0.15	0.232	520	0.01	-0.07	0.09	0.808	520	-0.04	-0.13	0.05	0.396
Ratio of monounsaturated fatty acids to total fatty acids (%)	520	-0.05	-0.14	0.04	0.274	520	-0.06	-0.14	0.03	0.186	520	-0.01	-0.10	0.07	0.742
Ratio of saturated fatty acids to total fatty acids (%)	520	0.00	-0.08	0.09	0.947	520	0.06	-0.02	0.14	0.157	520	0.05	-0.04	0.14	0.302
Glucose (mmol/l)	520	-0.01	-0.09	0.06	0.721	520	0.01	-0.08	0.10	0.812	520	0.04	-0.05	0.13	0.427
Lactate (mmol/l)	520	0.00	-0.08	0.08	0.958	520	0.06	-0.02	0.14	0.165	520	0.07	-0.02	0.16	0.119
Pyruvate (mmol/l)	520	-0.01	-0.09	0.07	0.849	520	0.05	-0.03	0.13	0.233	520	0.06	-0.03	0.15	0.174
Citrate (mmol/l)	520	0.04	-0.06	0.14	0.436	520	0.02	-0.08	0.13	0.651	520	0.02	-0.08	0.11	0.710
Alanine (mmol/l)	520	0.00	-0.09	0.09	0.993	520	0.11	0.02	0.20	0.017	520	0.12	0.03	0.22	0.014
Glutamine (mmol/l)	520	-0.01	-0.09	0.06	0.695	520	0.02	-0.05	0.10	0.496	520	0.03	-0.05	0.10	0.493
Histidine (mmol/l)	520	0.06	-0.03	0.15	0.226	520	0.07	-0.02	0.16	0.142	520	0.02	-0.07	0.12	0.607
Isoleucine (mmol/l)	520	-0.02	-0.09	0.05	0.589	520	0.03	-0.04	0.10	0.387	520	0.05	-0.03	0.14	0.219
Leucine (mmol/l)	520	0.02	-0.04	0.09	0.491	520	0.07	0.00	0.13	0.058	520	0.04	-0.03	0.12	0.282
Valine (mmol/l)	520	0.01	-0.06	0.09	0.759	520	0.06	-0.02	0.14	0.124	520	0.05	-0.04	0.13	0.280
Phenylalanine (mmol/l)	520	0.01	-0.07	0.09	0.842	520	0.01	-0.08	0.10	0.821	520	-0.04	-0.12	0.04	0.382
Tyrosine (mmol/l)	520	0.04	-0.05	0.13	0.371	520	0.00	-0.09	0.09	0.979	520	-0.08	-0.18	0.01	0.076
Acetate (mmol/l)	520	0.03	-0.05	0.11	0.494	520	0.06	-0.01	0.14	0.097	520	0.05	-0.04	0.14	0.299
Acetoacetate (mmol/l)	520	0.02	-0.05	0.09	0.502	520	0.01	-0.06	0.09	0.734	520	0.00	-0.08	0.07	0.899
3-hydroxybutyrate (mmol/l)	520	0.02	-0.06	0.10	0.676	520	-0.01	-0.08	0.07	0.826	520	-0.02	-0.11	0.07	0.632
Creatinine (mmol/l)	520	-0.04	-0.11	0.04	0.322	520	0.01	-0.07	0.08	0.881	520	0.06	-0.01	0.13	0.111
Albumin (signal area)	520	-0.01	-0.09	0.07	0.776	520	0.05	-0.03	0.13	0.196	520	0.07	-0.02	0.16	0.145
Glycoprotein acetyls, mainly a1-acid glycoprotein (mmol/l)	520	0.01	-0.07	0.08	0.850	520	-0.03	-0.10	0.04	0.430	520	-0.06	-0.14	0.03	0.214
