# Supplementary material for: Associations of device-measured physical activity across adolescence with metabolic traits: Prospective cohort study
Source: PLoS Med. 2018 Sep 11;15(9):e1002649. doi: 10.1371/journal.pmed.1002649 (PMC6133272; doi:10.1371/journal.pmed.1002649)
Supplement: S15 Table — ALSPAC, Avon Longitudinal Study of Parents and Children. (PDF) [file pmed.1002649.s015.pdf]

S15 Table Associations of physical activity measures with adiposity at age 15y in ALSPAC

Standardised exposure (SD-units)	Adjustment	Standardised outcome at age 15y<br>Fat mass index (kg/m^2)				
		N	Beta	LCL	UCL	P-value
**Current activity**						
CPM at age 15y	Age, sex, ethnicity, maternal education, smoking, alcohol, wear time, wear month	1800	-0.12	-0.17	-0.08	2.96E-07
CPM at age 15y	Age, sex, ethnicity, maternal education, smoking, alcohol, wear time, wear month, FMI at 10y	1613	-0.04	-0.07	-0.01	0.008
MVPA at age 15y	Age, sex, ethnicity, maternal education, smoking, alcohol, wear time, wear month	1800	-0.11	-0.15	-0.06	1.72E-06
MVPA at age 15y	Age, sex, ethnicity, maternal education, smoking, alcohol, wear time, wear month, SED at 15y	1800	-0.11	-0.15	-0.06	4.82E-06
MVPA at age 15y	Age, sex, ethnicity, maternal education, smoking, alcohol, wear time, wear month, SED at 15y, FMI at 10y	1613	-0.06	-0.09	-0.03	2.28E-04
SED at age 15y	Age, sex, ethnicity, maternal education, smoking, alcohol, wear time, wear month	1800	0.03	-0.01	0.08	0.155
SED at age 15y	Age, sex, ethnicity, maternal education, smoking, alcohol, wear time, wear month, MVPA at 15y	1800	0.01	-0.04	0.05	0.825
SED at age 15y	Age, sex, ethnicity, maternal education, smoking, alcohol, wear time, wear month, MVPA at 15y, FMI at 10y	1613	-0.02	-0.05	0.02	0.287
**Longer-term activity**						
Mean of CPM at age 12y, 14y, 15y	Age, sex, ethnicity, maternal education, smoking, alcohol, mean wear time, wear month	1292	-0.22	-0.28	-0.15	3.32E-10
Mean of CPM at age 12y, 14y, 15y	Age, sex, ethnicity, maternal education, smoking, alcohol, mean wear time, wear month, FMI at 10y	1192	-0.05	-0.10	-0.01	0.031
Mean of MVPA at age 12y, 14y, 15y	Age, sex, ethnicity, maternal education, smoking, alcohol, mean wear time, wear month	1292	-0.20	-0.27	-0.14	1.96E-09
Mean of MVPA at age 12y, 14y, 15y	Age, sex, ethnicity, maternal education, smoking, alcohol, mean wear time, wear month, mean SED	1292	-0.18	-0.25	-0.11	4.09E-07
Mean of MVPA at age 12y, 14y, 15y	Age, sex, ethnicity, maternal education, smoking, alcohol, mean wear time, wear month, mean SED, FMI at 10y	1192	-0.11	-0.16	-0.06	1.77E-05
Mean of SED at age 12y, 14y, 15y	Age, sex, ethnicity, maternal education, smoking, alcohol, mean wear time, wear month	1292	0.12	0.06	0.19	3.26E-04
Mean of SED at age 12y, 14y, 15y	Age, sex, ethnicity, maternal education, smoking, alcohol, mean wear time, wear month, mean MVPA	1292	0.06	-0.01	0.13	0.109
Mean of SED at age 12y, 14y, 15y	Age, sex, ethnicity, maternal education, smoking, alcohol, mean wear time, wear month, mean MVPA, FMI at 10y	1192	-0.05	-0.10	0.00	0.052
**Change in activity**						
Change in CPM from age 12y-15y	Age, sex, ethnicity, maternal education, smoking, alcohol, change in wear time, wear month	1555	0.05	0.00	0.09	0.033
Change in CPM from age 12y-15y	Age, sex, ethnicity, maternal education, smoking, alcohol, change in wear time, wear month, FMI at 10y	1423	-0.03	-0.06	0.00	0.042
Change in MVPA from age 12y-15y	Age, sex, ethnicity, maternal education, smoking, alcohol, change in wear time, wear month	1555	0.06	0.01	0.10	0.010
Change in MVPA from age 12y-15y	Age, sex, ethnicity, maternal education, smoking, alcohol, change in wear time, wear month, change in SED	1555	0.04	0.00	0.08	0.068
Change in MVPA from age 12y-15y	Age, sex, ethnicity, maternal education, smoking, alcohol, change in wear time, wear month, change in SED, FMI at 10y	1423	-0.02	-0.05	0.01	0.273
Change in SED from age 12y-15y	Age, sex, ethnicity, maternal education, smoking, alcohol, change in wear time, wear month	1555	-0.07	-0.11	-0.02	0.003
Change in SED from age 12y-15y	Age, sex, ethnicity, maternal education, smoking, alcohol, change in wear time, wear month, change in MVPA	1555	-0.06	-0.10	-0.01	0.020
Change in SED from age 12y-15y	Age, sex, ethnicity, maternal education, smoking, alcohol, change in wear time, wear month, change in MVPA, FMI at 10y	1423	0.01	-0.03	0.04	0.662

Complete case sample

Standardised exposure	Adjustment	Standardised outcome at age 15y<br>Fat mass index (kg/m^2)				
		N	Beta	LCL	UCL	P-value
**Current activity**						
CPM at age 15y	Age, sex, ethnicity, maternal education, smoking, alcohol, wear time, wear month	1192	-0.10	-0.15	-0.04	5.91E-04
CPM at age 15y	Age, sex, ethnicity, maternal education, smoking, alcohol, wear time, wear month, FMI at 10y	1192	-0.04	-0.08	-0.002	0.038
MVPA at age 15y	Age, sex, ethnicity, maternal education, smoking, alcohol, wear time, wear month	1192	-0.06	-0.12	-0.01	0.017
MVPA at age 15y	Age, sex, ethnicity, maternal education, smoking, alcohol, wear time, wear month, SED at 15y	1192	-0.06	-0.11	0.00	0.040
MVPA at age 15y	Age, sex, ethnicity, maternal education, smoking, alcohol, wear time, wear month, SED at 15y, FMI at 10y	1192	-0.05	-0.08	-0.01	0.011
SED at age 15y	Age, sex, ethnicity, maternal education, smoking, alcohol, wear time, wear month	1192	0.04	-0.01	0.10	0.123
SED at age 15y	Age, sex, ethnicity, maternal education, smoking, alcohol, wear time, wear month, MVPA at 15y	1192	0.03	-0.03	0.08	0.348
SED at age 15y	Age, sex, ethnicity, maternal education, smoking, alcohol, wear time, wear month, MVPA at 15y, FMI at 10y	1192	-0.01	-0.05	0.03	0.720
**Longer-term activity**						
Mean of CPM at age 12y, 14y, 15y	Age, sex, ethnicity, maternal education, smoking, alcohol, mean wear time, wear month	1192	-0.23	-0.30	-0.16	2.09E-10
Mean of CPM at age 12y, 14y, 15y	Age, sex, ethnicity, maternal education, smoking, alcohol, mean wear time, wear month, FMI at 10y	1192	-0.05	-0.10	-0.01	0.031
Mean of MVPA at age 12y, 14y, 15y	Age, sex, ethnicity, maternal education, smoking, alcohol, mean wear time, wear month	1192	-0.20	-0.27	-0.13	8.03E-09
Mean of MVPA at age 12y, 14y, 15y	Age, sex, ethnicity, maternal education, smoking, alcohol, mean wear time, wear month, mean SED	1192	-0.18	-0.25	-0.11	2.04E-06
Mean of MVPA at age 12y, 14y, 15y	Age, sex, ethnicity, maternal education, smoking, alcohol, mean wear time, wear month, mean SED, FMI at 10y	1192	-0.11	-0.16	-0.06	1.77E-05
Mean of SED at age 12y, 14y, 15y	Age, sex, ethnicity, maternal education, smoking, alcohol, mean wear time, wear month	1192	0.13	0.06	0.20	1.71E-04
Mean of SED at age 12y, 14y, 15y	Age, sex, ethnicity, maternal education, smoking, alcohol, mean wear time, wear month, mean MVPA	1192	0.07	0.00	0.14	0.064
Mean of SED at age 12y, 14y, 15y	Age, sex, ethnicity, maternal education, smoking, alcohol, mean wear time, wear month, mean MVPA, FMI at 10y	1192	-0.05	-0.10	0.00	0.052
**Change in activity**						
Change in CPM from age 12y-15y	Age, sex, ethnicity, maternal education, smoking, alcohol, change in wear time, wear month	1192	0.05	0.00	0.10	0.031
Change in CPM from age 12y-15y	Age, sex, ethnicity, maternal education, smoking, alcohol, change in wear time, wear month, FMI at 10y	1192	-0.03	-0.06	0.00	0.094
Change in MVPA from age 12y-15y	Age, sex, ethnicity, maternal education, smoking, alcohol, change in wear time, wear month	1192	0.07	0.03	0.12	2.00E-03
Change in MVPA from age 12y-15y	Age, sex, ethnicity, maternal education, smoking, alcohol, change in wear time, wear month, change in SED	1192	0.07	0.02	0.11	0.008
Change in MVPA from age 12y-15y	Age, sex, ethnicity, maternal education, smoking, alcohol, change in wear time, wear month, change in SED, FMI at 10y	1192	-0.01	-0.04	0.02	0.551
Change in SED from age 12y-15y	Age, sex, ethnicity, maternal education, smoking, alcohol, change in wear time, wear month	1192	-0.05	-0.10	0.00	0.043
Change in SED from age 12y-15y	Age, sex, ethnicity, maternal education, smoking, alcohol, change in wear time, wear month, change in MVPA	1192	-0.03	-0.08	0.02	0.231
Change in SED from age 12y-15y	Age, sex, ethnicity, maternal education, smoking, alcohol, change in wear time, wear month, change in MVPA, FMI at 10y	1192	0.01	-0.02	0.05	0.403
