# Supplementary material for: Associations of device-measured physical activity across adolescence with metabolic traits: Prospective cohort study
Source: PLoS Med. 2018 Sep 11;15(9):e1002649. doi: 10.1371/journal.pmed.1002649 (PMC6133272; doi:10.1371/journal.pmed.1002649)
Supplement: S1 Protocol — (PDF) [file pmed.1002649.s016.pdf]

# Submission information

# Objectively assessed habitual physical activity and future metabolic functioning (B2853)

Submission date/time: 02/03/2017 10:35:19

## *Principal Applicant Details*

**Principal Applicant Title:** Dr

**Your name:** Joshua Bell

**Position Held (note: If you are a PhD student, the proposal should be submitted by your supervisor with you listed as a co-applicant):** Research Associate, Integrative Cancer Epidemiology Programme

**Is this a student project?:**

**What type of student project is this?:**

**Please specify what type of student project this is...:**

**Affiliation:** MRC IEU at the University of Bristol

**Are you a 'direct access' user of ALSPAC data, or a member of the Integrative Epidemiology Unit (IEU)?:** Yes

**Address line 1:** Oakfield House

**Address line 2:**

**City:** Bristol

**Country:** United Kingdom

**Postcode:** BS8 2BN

**Email:** j.bell@bristol.ac.uk

**Telephone:**

## *Co-applicant details*

**How many co-applicants?:** 4

## Co-applicant 1

**Name and title:** Prof George Davey Smith
**Position Held and Affiliation:** Professor and Director of MRC IEU, University of Bristol
**Email:** kz.davey-smith@bristol.ac.uk
**Role in Project:** Line manager / Project supervisor

## Co-applicant 2

**Name and title:** Dr David Carslake
**Position Held and Affiliation:** Senior Research Associate, MRC IEU, University of Bristol
**Email:** david.carslake@bristol.ac.uk
**Role in Project:** Line manager / Project supervisor
**Data use: please tick this box if the co-applicant will use the data. If this field is empty, the co-applicant will not use the data:** Yes

## Co-applicant 3

**Name and title:** Dr Rebecca Richmond
**Position Held and Affiliation:** Senior Research Associate, MRC IEU, University of Bristol
**Email:** rebecca.richmond@bristol.ac.uk
**Role in Project:** Collaborator
**Data use: please tick this box if the co-applicant will use the data. If this field is empty, the co-applicant will not use the data:** Yes

## Co-applicant 4

**Name and title:** Prof Mark Hamer
**Position Held and Affiliation:** Professor and Chair in Exercise as Medicine, National Centre for Sport and Exercise Medicine, Loughborough University
**Email:** m.hamer@lboro.ac.uk
**Role in Project:** Collaborator

## Co-applicant 5

## Co-applicant 6

# Co-applicant 7

# Co-applicant 8

# Co-applicant 9

# Co-applicant 10

# *PROJECT DETAILS*

**Title of project:** Objectively assessed habitual physical activity and future metabolic functioning

**Proposed Project Start Date:** 2017-03-10

**Proposed Project Finish Date:** 2018-03-10

**Is your project currently funded?:** Yes

**Current project funding:** CRUK Integrative Cancer Epidemiology Programme, IEU, University of Bristol

**Are you seeking funding for this project?:** No

----------------Potential funding fields----------------
**Have you previously had a project with us?:** Yes

**Data buddy name (if one has been assigned to you):**

**Where did you find out about ALSPAC?:** Word of mouth

**Please specify where you heard about ALSPAC?:**

**Research dissemination:** Peer review journal article, Conference

**Other plans for disseminating research findings:**

----------------Invoice details----------------
- **Name:** George Davey Smith

- **Position Held:** Professor and Director of MRC IEU, University of Bristol

- **Address:** Oakfield House

- **Email:** kz.davey-smith@bristol.ac.uk

- **Telephone number:**

# *Description of Project*

**Project summary for laypersons:** Physical activity is widely linked with better metabolic functioning, but whether these links reflect distinct causal effects is unclear. In the absence of robust genetic instruments for physical activity, we aim to examine habitual levels of physical activity across adolescence in relation to future metabolic profiles in young adulthood. To do this, we aim to use 3 repeated measures of objectively assessed physical activity (total, light, moderate-to-vigorous, and sedentary time), along with 2 repeated measures of objectively assessed fitness and lean body mass, and to relate these to a wide array of blood-based metabolic markers at follow-up (and to change in these from historical assessments) in the form of clinically relevant traits including fasting insulin and over 80 metabolites including branched chain amino acids. The ALSPAC cohort of children is an ideal resource for carrying out this study given availability of repeated objective measures and the young age of participants which allows associations to be estimated with minimal bias due to subclinical disease.

**Aim(s) and objective(s):** Aims

1) To examine whether habitually high levels of total physical activity, light physical activity, and moderate-to-vigorous physical activity, along with habitually low levels of sedentary time, relate favourably to future metabolic functioning

2) To compare the relative importance of each physical activity domain for future metabolism

3) To examine whether patterns of associations between habitual physical activity and metabolic outcomes mimic known causal effects of BMI on those same metabolic outcomes

4) To examine whether persistently high levels of fitness and lean body mass relate favourably to future metabolic functioning

5) To examine these exposures in relation to change in metabolic traits and metabolites

Objectives

1) To test associations of higher total physical activity, higher light physical activity, higher moderate-to-vigorous physical activity, and lower sedentary time, based on 3 measures during adolescence (at age 11, 13, and 15), in the form of an average across all repeated assessments and of high vs. low tertiles, with metabolic traits and metabolites at age 17

2) To compare the existence, magnitude, and pattern of association between each physical activity domain with each metabolic outcome

3) To compare the existence, magnitude, and pattern of physical activity-metabolic outcome associations with those of body mass index-metabolic outcome associations known from published Mendelian randomisation studies

4) To test associations of higher fitness and higher regional and total lean body mass based on 2 measures in adolescence (age 13 and 15), in the form of an average across all repeated assessments and of high vs. low tertiles, with metabolic traits and metabolites at age 17

5) To test associations of these exposures with change in metabolic traits and metabolites from baseline (age 15) and pre-baseline (age 7) to follow-up (age 17).

**Methods (including an overview of statistical methods):** Individual-level clinic and questionnaire data on ALSPAC children will be used for all analyses.

Habitual physical activity will be derived from objective assessments of physical activity on 3 occasions (at age 11, 13, and 15) in two ways; first, by using an average level across all 3 occasions to represent a single habitual level as a continuous variable; and second, by using data-derived thresholds (i.e. upper vs. lower tertile on all 3 occasions) as a categorical variable for persistent exposure. This will be done for all physical activity domains (total, light, moderate-to-vigorous, and sedentary time), as well as for objectively-assessed fitness level and lean body mass.

Associations of these exposures with metabolic outcomes will be examined using linear regression models, outcome variables being log transformed when necessary and standardised (mean=0, standard deviation=1) to allow comparability of effect sizes. Models would be adjusted for age and sex at a minimum, with subsequent adjustments for socioeconomic factors, health behaviours, puberty timing, and body mass index. Correction of significance thresholds will be made for multiple testing, likely on the basis of principle component analyses given the correlated nature of metabolic outcomes.

Analyses will be repeated for these exposures in relation to change in each metabolic trait and metabolite from baseline (age 15) to follow-up (age 17), additionally incorporating a pre-baseline (historical) assessment from age 7. Linear mixed modelling may be used in replace of basic linear modelling to examine such changes.

Patterns of association will be compared visually and quantitatively to those observed in published Mendelian randomisation studies of body mass index and metabolic outcomes (i.e. Holmes et al., Causal Effects of Body Mass Index on Cardiometabolic Traits and Events: A Mendelian Randomization Analysis, Am J Hum Genet, 2014; Wurtz et al., Metabolic Signatures of Adiposity in Young Adults: Mendelian Randomization Analysis and Effects of Weight Change, PLoS Med, 2014) in order to estimate the likelihood of any observed associations of physical activity with metabolic outcomes reflecting causal effects of body mass index.

Such observational analyses are planned in light of robust genetic variants for

habitual physical activity being unavailable to date for use within a Mendelian randomisation framework. If, during the course of this study, such variants were to become available in literature, these variants will be used as instruments for habitual physical activity as a complementary analysis. This will require additional use of imputed genotype data on ALSPAC children; variants will be specified if needed.

**Exposures, outcomes and confounders to be considered (justifying particular types of data as necessary):** Exposures

At age 11, 13, and 15: Accelerometer (Actigraph)-derived total physical activity (total counts per minute or day), moderate-to-vigorous physical activity (minutes per day), light physical activity (minutes per day), and sedentary time (minutes per day)

At age 13 and 15: Bicycle test-derived fitness level (heart rate and blood pressure at repeated intervals during and after testing, and timing of early completion); Dual-energy x-ray absorptiometry-(DEXA) derived whole body and regional lean mass in gram units (total body, arms, legs, trunk, android, gynoid)

Outcomes

At age 17 (follow-up), 15 (baseline for change) and 7 (pre-baseline for change): Clinic measures where available of resting systolic and diastolic blood pressure, fasting blood glucose, fasting insulin, derived insulin sensitivity, HDL cholesterol, total cholesterol, LDL cholesterol, triglycerides, C-reactive protein, interleukin-6, and all available (approximately 80) NMR-derived blood-based metabolite concentrations

Covariates

At age 15 (main baseline), 13, 11, and 7 (historical): Age in months; sex; ethnicity; mother’s education level; birth weight; body mass index; puberty timing (age at menarche or voice breaking); smoking status; alcohol consumption

**Reasons for using ALSPAC:** The ALSPAC resource is uniquely suited for examining links between habitual physical activity and metabolic functioning, given the availability of repeated objective measures of different domains of physical activity and of fitness and lean body mass (a rarity in physical activity research), in addition to a wide array of blood-based markers of metabolic functioning in the form of both clinically relevant metabolic traits and detailed nuclear magnetic resonance-derived metabolites. Further advantages of using ALSPAC include the ability to examine change in metabolic outcomes from baseline and historical pre-baseline assessments, as well as the young age of participants with exposure and outcome assessments made between adolescence and young adulthood, allowing for minimal bias in associations due to subclinical disease. Together, this allows for a strong study design and a significant contribution to the observational epidemiology of physical activity and health.

**What do you think the likely impact of your research will be?:** The likely output of this research will be at least 1 publication in a general medical or epidemiology journal, the impact of which may be theoretical advancement in active research fields of basic biology, health behaviour and clinical disease epidemiology, and dissemination to public health practice.

**Subject classification (please select one):** Epidemiology

**Other subject - please specify:**

----------------Diseases/conditions (click to expand/collapse)----------------
- **Please tick all appropriate diseases/conditions:** Diabetes, Hypertension, Obesity, Other (please specify)

- **Other disease/condition - please specify:** Metabolic functioning

----------------Techniques (click to expand/collapse)----------------
- **Please tick all appropriate techniques:** Metabolomics, NMR, Statistical methods

- **Other techniques - please specify:**

----------------Keywords (click to expand/collapse)----------------
- **Please tick all appropriate keywords:** Biological samples -e.g. blood, cell lines, saliva, etc., Biomarkers - e.g. cotinine, fatty acids, haemoglobin, etc., Blood pressure, BMI, Cardiovascular, Cohort studies - attrition, bias, participant engagement, ethics, Childhood - childcare, childhood adversity, Metabolic - metabolism, Physical - activity, fitness, function

- **Methods - please specify:**

- **Other keyword(s) (please specify):**

# *Types of existing data required*

**Does your project involve analysing existing data?:** Yes
**Existing questionnaire data:** Yes

**Existing clinic data:** Yes

**Existing data from biological samples (not genetic):** Yes

----------------Existing genome-wide SNP genotype data----------------
- **Existing genetics data:** No

----------------Existing Methylation Data----------------

- **Methylation data:** No

----------------Linkage data----------------
- **Third party data (e.g. data provided by education/health organisations):** No

----------------Address Data----------------
- **Do you require any data linked to addresses?:** No

----------------Text data----------------
- **Text data:** No

# *NEW data or sample collection*

**Are you requesting the collection of new data and/or biosamples?:** No
----------------New data/biosamples----------------
- **Please name the person responsible for handling any incidental findings from the data / sample collection:**

- **Please provide details of what data you want to collect:**

- **How will your data be collected?:**

# *Do you want new genotyping carried out by LGC?*

**SNP genotyping:** No

----------------LGC genotyping info----------------

# *Do you want new genotyping or methylation data generated on the Illumina platform?*

**Do you require Illumina arrays to be run in the ALSPAC Laboratory?:** No

----------------Illumina array info----------------

# *Do you require DNA samples for other analysis?*

**Do you require DNA samples to analyse elsewhere?:** No

----------------Dna samples info----------------

**- Laboratory name:**

**- Laboratory address:**

**- Contact person:**

**- Laboratory email address:**

**- Laboratory phone number:**

**- DNA is supplied as standard aliquots, 250ng at 10ng/microliter. Is your request for a non-standard quantity of DNA?:**

**- Justification for non-standard aliquots:**

**- Quantity of DNA required:**

**- Minimum concentration required:**

**- Details of samples required:**

**- Will you perform SNP and/or micro-satellite analysis?:**

**- SNPs list:**

**- Will you perform sequencing analysis?:**

**- Sequencing details:**

**- Will you perform structural DNA work (including copy number variation)?:**

**- Genotyping details:**

**- If you wish to perform another form of genotyping/analysis on our samples, please give details here.:**

**- Cell-line DNA:**

**- If you are not happy to receive cell-line DNA, please provide an explanation for your requirements.:**

**- Laboratory protocols:**

**- Laboratory protocols - confirmation:**

**- I understand that all genotypes will be returned to ALSPAC and be made**

**available to other researchers:** Yes

## *Other biological samples*

**Will the project require access to biological samples other than DNA?:** No
**1. Please confirm that...:** ...you are familiar with the latest version of the ALSPAC access policy
**2. Please confirm that...:** ...you understand it is your responsibility to ensure that all members of your team working on this project complete confidentiality forms and that you inform ALSPAC of any changes to the team
**3. Please confirm that...:** ...you understand that data and samples from the ALSPAC resource cannot be used for commercial gain
**4. Please confirm that...:** ...you understand that you and your team must not pass on any data or samples awarded, or any derived variables or genotypes generated by this application to a third party (i.e. to anybody who is not included in the list of applicants on this form)
**5. Please confirm that...:** ...you aware that any third party seeking to use data, samples, or derived variables or genotypes arising from this application must approach ALSPAC to obtain access permission of their own
**6. Please confirm that...:** ...you understand that if a problem arises involving any misuse of the ALSPAC data or samples provided for this project that violates any of the terms and conditions specified by the Materials Transfer Agreement (MTA), Data Transfer Agreement (DTA) or confidentiality form that you (as principal applicant) have signed, you will be held responsible. This might result in you being excluded from using the ALSPAC resource in the future.
**7. I understand that...:** ...costs will be determined after the proposal has been approved and that I will not receive any data or samples until I have settled my invoice or provided a purchase order number.
**8. I understand that...:** ...all genotypes and/or data generated from biological samples will be returned to ALSPAC and be made available to other researchers.
**9. I declare that...:** ...I have no conflict of interest in relation to this research.
**If you do have a conflict on interest, please declare it here:**
**Date:** 2017-03-02
**Print name (this will serve as your signature):** Joshua Bell
